# Dissecting Causal Relationships Between Gut Microbiota, Plasma Metabolites and Bladder Cancer: A Two‐Step Mendelian Randomization Study

**DOI:** 10.1002/hsr2.71206

**Published:** 2025-09-09

**Authors:** Kai Che, Dong Qian, Shuxia Cui, Fei Xie

**Affiliations:** ^1^ Department of Urology The Affiliated Hospital of Qingdao University Qingdao China; ^2^ Department of Clinical Medicine Qingdao university Qingdao China; ^3^ Department of Clinical Pharmacy Qilu Hospital of Shandong University (Qingdao) Qingdao China; ^4^ Department of Critical Care Medicine The Affiliated Hospital of Qingdao University Qingdao China

**Keywords:** bladder cancer, gut microbiota, Mendelian randomization, plasma metabolites

## Abstract

**Background and Aims:**

Previous studies have shown that gut microbiota is associated with bladder cancer. However, the causal relationships and potential mediating factors between the gut microbiota (GM) and bladder cancer (BCa) have not been well defined.

**Methods:**

To investigate this, we utilized summary statistics from genome‐wide association studies of gut microbiota (Dutch Microbiome Project, *n* = 7738), plasma metabolites (Canadian Longitudinal Study of Aging follows, *n* = 8096), and BCa (FinnGen Biobank R9, 2053 cases and 287,137 controls). We then conducted bidirectional Mendelian randomization (MR) analyses to explore the causal relationships between GM and BCa, and employed a two‐step MR approach to identify potential mediating metabolites.

**Results:**

Our study revealed that three taxa, Species Bacteroides dorei, Genus Streptococcus Species and Bacteroides salyersiae, were associated with BCa, while no association was found between BCa on these three taxa. Additionally, we identified 5 plasma metabolites that were simultaneously associated with BCa and the above three taxa. Mediation analysis showed that the associations between Species Bacteroides salyersiae and BCa were mediated by N‐methylproline and X‐19299, accounting for 11.6% and 28.56% of the total effect, respectively. Furthermore, Species Bacteroides dorei, Genus Streptococcus Species and Bacteroides salyersiae potentially affected BCa through 2,3‐dihydroxypyridine, N‐palmitoyl‐sphinganine and N‐methylproline, respectively.

**Conclusions:**

Overall, our study dissected the causal relationships between GM and BCa, potentially mediated by plasma metabolites. Our study identified potential targets for treatment of bladder cancer.

## 1 Background

Bladder cancer (BCa), one of the most common cancers of genitourinary system, is the tenth most common cancer worldwide [1]. It was estimated by GLOBOCAN that there were 573,000 new BCa cases and 213,000 deaths worldwide in 2020 [2]. Urothelial carcinoma is the main subtype of BCa, and approximately 25% of BCa cases are muscle invasive BCa (MIBC), which requires systemic chemotherapy, immunotherapy, and radical treatment [3]. Risk factors for BCa include Tobacco smoking, occupational exposures (aromatic amines and polycyclic aromatic hydrocarbons), medications (cyclophosphamide), and Schistosoma infection [4]. Recent studies have proposed that gut microbiota may influence cancer development and treatment response in genitourinary malignancies [5]. It is crucial to dissect the causal relationships between BCa and gut microbiota and the underlying mechanism.

Gut microbiota (GM) have been shown to play an important role in the development and progression of various cancers, including colorectal cancer(CRC) [6, 7], gastric cancer [8], liver cancer [9], lung cancer [10], prostate cancer(PCa) [11] and bladder cancer [12]. A progressive trend for an increased abundance of Fusobacterium nucleatum was observed in CRC, from highly dysplastic adenomas to late cancer stages [12]. And Fusobacterium nucleatum could promote proliferation of cancer cells to activate the development of CRC [7]. Matsushita et al. reported that Rikenellaceae, Alistipes, and Lachnospira were considerably increased in men with high Gleason prostate cancer [11]. He et al. demonstrated that among BCa patients, Prevotella, Clostridium cluster XI were significantly reduced [13]. However, the mechanism through which GM affect BCa remain unclear.

Interestingly, some investigations had reported that GM could affect the development and progression of cancers by producing a variety of metabolites, including bile acids (BAs), short‐chain fatty acids (SCFAs), Trimethylamine n‐oxide (TMAO), and amino acids [14, 15]. Deoxycholic Acid (DCA), one of the main secondary BAs produced by Clostridium, acts as a tumor promoter in CRC [16]. Utilizing gnotobiotic mouse models, Donohoe et al. proposed that fiber have a potent tumor‐suppressive effect in a microbiota‐ and butyrate‐dependent manner [17]. Lukas et al. found that intestinal B. pseudolongum enhanced cancer immunotherapy response through production of the metabolite inosine [18]. Wang et al. observed the correlation between BCa and Butyricicoccus pullicaecorum which could produce butyrate to mediate anticancer effects on BCa cells by regulating cell cycle, cell growth, apoptosis, and gene expression [19]. Therefore, we sought to clarify these associations between GM, metabolites, and BCa.

Mendelian randomization (MR) is a method that utilizes genetic variants as instrumental variables (IVs) to explore causality between exposure and outcome. It can minimize confounding and reverse causation, which are inherent limitations of conventional observational studies. In our study, we aimed to explore the causal relationships between GM and BCa and whether it can be mediated by plasma metabolites. Initially, a two‐sample MR analysis was performed to investigate the causal effects between GM and BCa. Subsequently, plasma metabolites that have causal relationships with GM and BCawere identified. Finally, the proportion of GM's effect on BCa mediated by plasma metabolites were calculated to assess whether GM could affect BCa via plasma metabolites. The results of our study could potentially provide valuable insights into the complex interactions between GM, plasma metabolites, and BCa, pointing to directions for further research and potential therapeutic interventions.

## 2 Methods

### 2.1 Study Design

**Figure 1:**
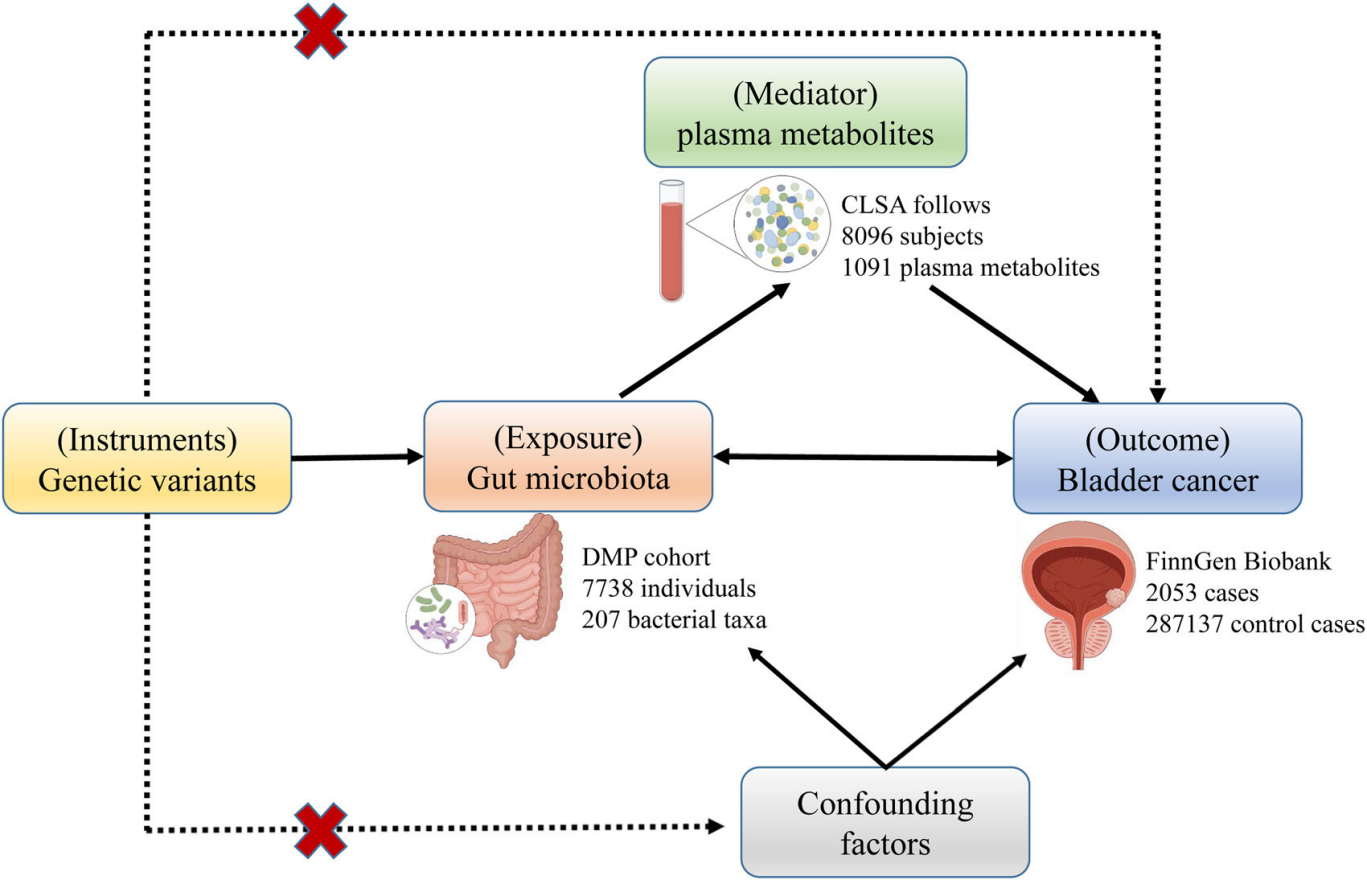
The study design. A two‐step Mendelian randomization study of GM on BCa mediated by plasma metabolites. GM: Gut microbiota; BCa: Bladder cancer.

The study design was depicted in Figure 1. We initially investigated the causal relationships between gut microbiota and BCa. Bidirectional MR was conducted to distinguish direct effects from reverse causality. Subsequently, we identified the plasma metabolites associated with GM and BCa simultaneously. Finally, to identify potential mediating metabolites, we utilized a two‐step MR approach to explore the causal associations between gut microbiota and BCa mediated by plasma metabolites. Additionally, Figure 1 illustrates that the genetic variants used as IVs termed single nucleotide polymorphisms (SNPs) should fulfill three criteria [1]: strongly predict the exposures [2], correlate with outcomes solely through exposure, and [3] remain independent of any confounders of expose‐outcome associations.

### 2.2 Data Sources

The summary statistics for gut microbiota, plasma metabolites, and bladder cancer were obtained from previous genome‐wide association studies (GWAS). Gut microbiota data were obtained from a GWAS involving 7738 European individuals from the Dutch Microbiome Project (DMP), which aims to evaluate the impact of different exposures and life‐styles on gut microbiota composition [20]. This project encompassed 207 taxonomies, including 5 phyla, 10 classes, 13 orders, 26 families, 48 genera, and 105 species. Notably, 205 bacterial pathways were excluded in our analysis.

Plasma metabolites data were extracted from the Canadian Longitudinal Study of Aging (CLSA) follows [21]. This metabolomics study focused on 8096 subjects of European ancestries within the CLSA cohort who were genotyped genome‐wide and had their circulating plasma metabolites measured. The study conducted genome‐wide association studies of 1091 plasma metabolites and 309 metabolite ratios. However, only 1091 individual plasma metabolites were included, and metabolite ratios were not incorporated into our analysis.

Data for bladder cancer were derived from FinnGen Biobank R9 [22], comprising 2053 BCa cases and 287,137 control cases. Individuals with excess heterozygosity (±4 SD), genotype missingness ≥ 5%, ambiguous sex and non‐Finnish ancestry were excluded. Bladder cancer cases were diagnosed according to ICD‐O‐3, with controls excluding all cancer diagnoses.

### 2.3 Genetic Instrumental Variables (IVs) Selection

SNPs exhibiting a genome‐wide significant association (*p* < 5 × 10^−8^) from the GWAS data for each exposure were selected as potential IVs. Due to the limited number of available IVs, we adjusted the significance threshold to *p* < 1 × 10^−5^ for gut microbiota, following the approach used in previous MR study [23, 24, 25]. For plasma metabolites, a more stringent threshold of *p* < 5 × 10^−8^ was applied to select genetic predictive factors. Subsequently, all those genetic variants were clumped with the threshold: R^2^ < 0.001 within 10000 kb clumping distance. We utilized PhenoScanner V2 (http://www.phenoscanner.medschl.cam.ac.uk/) to search for potential confounders and bypasses (e.g. age, sex, race, and other disease) associated with obtained SNPs. Finally, we calculated the F statistics for all SNPs to prevent the impact of weak IVs. Therefore, we excluded IVs with F < 10 when performing MR analysis.

### 2.4 Statistical Analysis

Five methods (“MR Egger”, “Weighted median”, “Inverse variance weighted (IVW)”, “Simple mode” and “Weighted mode”) were employed in our MR analysis [26, 27, 28, 29], IVW was the primary method for causal estimation due to its precision and robustness. *p* < 0.05 was considered to have a significant association between exposure and outcome. Cochran's Q statistic, based on IVW and MR Egger methods, was utilized to assess the degree of heterogeneity. We also utilized MR‐Egger intercept test to detect the pleiotropy. Leave‐one‐out analyses and funnel plots ruled out the possibility of potential outliers and horizontal pleiotropy. In addition, the Bayesian weighted Mendelian randomization (BWMR) was conducted to validate the results of MR study.

Two‐step MR analysis was performed to determine the mediation effect of plasma metabolites on the associations between gut microbiota and BCa. The proportion of the total effect mediated by plasma metabolites was estimated by dividing the indirect effect by the total effect (β1 × β2/β3), which β1 represents the effect of gut microbiota on plasma metabolites, β2 indicates the effect of plasma metabolites on BCa, and β3 refers to the effect of gut microbiota on BCa. The proportion of the mediation effect was not calculated if the total and direct effect were not in the same direction.

All analyses were performed using the R platform (version 4.2.1). The ‘TwoSampleMR’, ‘ggplot2’, and ‘foreach’ packages were used for statistical analyses and data visualizations.

## 3 Results

### 3.1 MR Analysis Between Gut Microbiota and BCa

**Figure 2:**
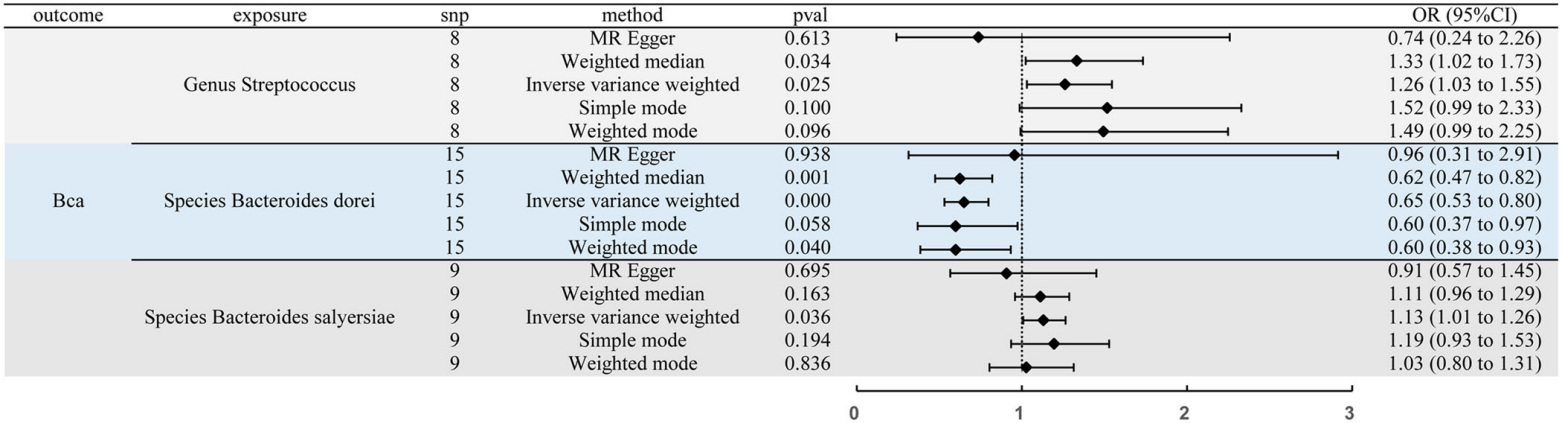
MR analysis showed the causality of 3 genera on BCa were significant (Genus Streptococcus, Species Bacteroides dorei, Species Bacteroides salyersiae). OR: odds ratio; CI: Confidence Interval; BCa: Bladder cancer.

In our study, we utilized bi‐direction Mendelian Randomization analysis to investigate the causal relationships between GM and BCa. As shown in Figure 2, both Genus Streptococcus (OR (odds ratio) = 1.26, 95% CI (confidence interval): 1.03–1.55, *p*‐value = 0.025) and Species Bacteroides salyersiae showed positive associations with BCa (OR = 1.13, 95% CI: 1.01–1.26, *p*‐value = 0.036), whereas Species Bacteroides dorei exhibited a protective effect (OR = 0.65, 95% CI: 0.53–0.80, *p*‐value < 0.001) on BCa. In addition, Genus Dorea displayed a positive relationship with BCa, with ORs of 1.42 (95% CI: 1.00–2.02, *p*‐value = 0.048) (Table S1). Importantly, no evidence of heterogeneity or pleiotropy in our MR analysis was indicated (Figure S1–S2, Table S1), enhancing the reliability of our findings.

To strengthen the reliability of our analysis, we conducted Bayesian weighted Mendelian Randomization (BWMR). The results were congruent with the associations identified in our initial analysis, with Genus Streptococcus and Species Bacteroides salyersiae showing positive associations with Bca, and Species Bacteroides dorei exhibiting a negative association with BCa (Table 1). Notably, Genus Dorea was excluded for further analysis because of its nonsignificant association with BCa in BWMR. Moreover, reverse MR analysis revealed no significant influence of BCa on the investigated gut microbiota (Table S2). These findings emphasize the potential impact of gut microbiota diversification on BCa development.

**Table 1:** Mendelian randomization analysis on the causal effect of GM on BCa using BWMR approach. Odds ratio (OR), 95% confidence interval (CI), Bayesian weighted Mendelian Randomization (BWMR), gut microbiota (GM), bladder cancer (BCa).

Exposure	Outcome	Method	OR	95% CI	*p* values
Genus Streptococcus	BCa	BWMR	1.329	1.088–1.624	0.005
Genus Dorea		BWMR	1.318	0.936–1.854	0.114
Species Bacteroides dorei		BWMR	0.641	0.516–0.796	< 0.001
Species Bacteroides salyersiae		BWMR	1.135	1.006–1.280	0.040

### 3.2 MR Analysis of Significant Gut Microbiota on Plasma Metabolites

To elucidate the mediating effects of GM on BCa through plasma metabolites, we employed a two‐step MR approach. Initially, we aimed to elucidate the associations between investigated gut microbiota and plasma metabolites. Our analysis identified 28, 52 and 38 metabolites using the IVW method to be associated with Genus Streptococcus (Table 2), Species Bacteroides dorei (Table 3) and Species Bacteroides salyersiae (Table 4), respectively. Of note, N, N‐dimethylalanine levels was identified as the most significant risk factor for Genus Streptococcus (OR = 1.13, 95% CI: 1.05–1.23, *p*‐value = 0.002), Maleate levels was the the most significant protective factor for Species Bacteroides dorei (OR = 0.84, 95% CI: 0.76–0.92, *p*‐value < 0.001), and Dihydroferulic acid sulfate levels was the the most significant risk factor for Species Bacteroides salyersiae (OR = 1.13, 95% CI: 1.05–1.22, *p*‐value = 0.002. The detailed results of other MR methods and sensitivity analysis are shown in Table S3, and no heterogeneity or pleiotropy was disclosed in our findings (Figure S3–S8, Table S3).

**Table 2:** Mendelian randomization analysis on the causal effect of plasma metabolite on Genus Streptococcus. Number of single‐nucleotide polymorphism (nsnp), Odds ratio (OR), 95% confidence interval (CI), Inverse variance weighted (IVW).

Outcome	Exposure	Method	nsnp	OR	95% CI	pval
Genus Streptococcus	Glucuronate levels	IVW	11	0.924	0.855–0.999	0.046
	3‐indoxyl sulfate levels	IVW	11	0.913	0.845–0.986	0.021
	Galactonate levels	IVW	11	0.917	0.846–0.995	0.037
	Alpha‐hydroxyisovalerate levels	IVW	11	1.083	1.005–1.167	0.036
	N4‐acetylcytidine levels	IVW	11	0.923	0.852–1.000	0.049
	Cysteine‐glutathione disulfide levels	IVW	11	0.903	0.819–0.996	0.041
	6‐oxopiperidine‐2‐carboxylate levels	IVW	11	1.090	1.007–1.180	0.032
	Eugenol sulfate levels	IVW	11	1.111	1.021–1.209	0.015
	6‐hydroxyindole sulfate levels	IVW	11	0.900	0.833–0.973	0.008
	N‐palmitoyl‐sphinganine (d18:0/16:0) levels	IVW	11	1.099	1.016–1.188	0.018
	1‐stearoyl‐2‐linoleoyl‐GPI (18:0/18:2) levels	IVW	11	1.097	1.015–1.186	0.020
	2‐hydroxyarachidate levels	IVW	11	1.114	1.019–1.217	0.018
	N,N‐dimethylalanine levels	IVW	11	1.134	1.048–1.227	0.002
	Pipecolate levels	IVW	11	1.096	1.013–1.186	0.023
	4‐hydroxyphenylacetate levels	IVW	11	0.901	0.834–0.974	0.009
	5,6‐dihydrothymine levels	IVW	11	0.921	0.852–0.995	0.037
	Methylsuccinate levels	IVW	11	0.888	0.804–0.980	0.018
	1‐methylnicotinamide levels	IVW	11	0.875	0.793–0.965	0.008
	Trans‐urocanate levels	IVW	11	0.920	0.850–0.995	0.036
	Salicylate levels	IVW	11	0.917	0.843–0.998	0.044
	Valine levels	IVW	11	1.093	1.012–1.180	0.024
	X‐12261 levels	IVW	11	0.861	0.754–0.984	0.028
	X‐12714 levels	IVW	11	0.914	0.840–0.995	0.038
	X‐13553 levels	IVW	11	0.929	0.865–0.999	0.046
	X‐23641 levels	IVW	11	1.095	1.007–1.007	0.034
	X‐25519 levels	IVW	11	1.092	1.009–1.181	0.028
	3‐methylcytidine levels	IVW	11	1.085	1.008–1.168	0.031
	N2‐acetyl,N6,N6‐dimethyllysine levels	IVW	11	0.909	0.828–0.999	0.048

**Table 3:** Mendelian randomization analysis on the causal effect of plasma metabolite on Species Bacteroides dorei. Number of single‐nucleotide polymorphism (nsnp), Odds ratio (OR), 95% confidence interval (CI), Inverse variance weighted (IVW).

Outcome	Exposure	Method	nsnp	OR	95% CI	pval
Species Bacteroides dorei	Quinate levels	IVW	15	0.887	0.787–0.999	0.048
	Gentisate levels	IVW	15	0.892	0.809–0.984	0.023
	Maleate levels	IVW	15	0.837	0.758–0.923	< 0.001
	2,3‐dihydroxypyridine levels	IVW	15	0.887	0.794–0.991	0.035
	Epiandrosterone sulfate levels	IVW	15	0.889	0.807–0.980	0.017
	N‐methyl‐2‐pyridone‐5‐carboxamide levels	IVW	15	0.883	0.791–0.985	0.026
	3‐(3‐hydroxyphenyl)propionate levels	IVW	15	1.116	1.005–1.240	0.041
	O‐cresol sulfate levels	IVW	15	0.861	0.776–0.956	0.005
	Glycosyl‐N‐stearoyl‐sphingosine (d18:1/18:0) levels	IVW	15	0.892	0.811–0.981	0.019
	5alpha‐androstan‐3alpha,17alpha‐diol monosulfate levels	IVW	15	0.878	0.784–0.984	0.025
	5alpha‐androstan‐3beta,17alpha‐diol disulfate levels	IVW	15	0.897	0.809–0.994	0.037
	5alpha‐androstan‐3beta,17beta‐diol monosulfate (2) levels	IVW	15	0.873	0.791–0.964	0.007
	5alpha‐androstan‐3alpha,17beta‐diol monosulfate(1) levels	IVW	15	0.870	0.789–0.960	0.005
	Imidazole propionate levels	IVW	15	0.907	0.823–1.000	0.050
	3‐methyl catechol sulfate (1)levels	IVW	15	0.857	0.777–0.946	0.002
	3‐acetylphenol sulfate levels	IVW	15	0.885	0.794–0.985	0.026
	S‐allylcysteine levels	IVW	15	0.880	0.791–0.979	0.019
	4‐vinylguaiacol sulfate levels	IVW	15	0.888	0.801–0.984	0.023
	3‐hydroxypyridine sulfate levels	IVW	15	0.845	0.767–0.931	0.001
	Citraconate/glutaconate levels	IVW	15	0.864	0.783–0.954	0.004
	1,2,3‐benzenetriol sulfate (2) levels	IVW	15	0.887	0.800–0.983	0.022
	Dopamine 3‐o‐sulfate levels	IVW	15	1.106	1.003–1.220	0.044
	Gamma‐glutamylcitrulline levels	IVW	15	1.140	1.032–1.259	0.010
	Glucuronide of piperine metabolite C17H21NO3 (3) levels	IVW	15	0.882	0.789–0.985	0.026
	6‐bromotryptophan levels	IVW	15	0.887	0.796–0.988	0.029
	3‐hydroxypyridine glucuronide levels	IVW	15	0.861	0.766–0.968	0.012
	4‐ethylcatechol sulfate levels	IVW	15	0.844	0.765–0.931	0.001
	3‐hydroxy‐2‐methylpyridine sulfate levels	IVW	15	0.817	0.728–0.918	0.001
	5‐hydroxy‐2‐methylpyridine sulfate levels	IVW	15	0.851	0.761–0.952	0.005
	4‐acetylcatechol sulfate (1) levels	IVW	15	0.893	0.804–0.993	0.036
	N‐acetylvaline levels	IVW	15	0.902	0.821–0.991	0.032
	Urate levels	IVW	15	0.900	0.823–0.984	0.021
	9,10‐DiHOME levels	IVW	15	1.124	1.014–1.246	0.026
	Inosine levels	IVW	15	1.198	1.055–1.360	0.005
	Nicotinamide levels	IVW	15	0.879	0.781–0.990	0.034
	Retinol (Vitamin A) levels	IVW	15	0.853	0.774–0.941	0.001
	1‐methylnicotinamide levels	IVW	15	0.888	0.805–0.979	0.017
	X‐11444 levels	IVW	15	0.886	0.808–0.972	0.011
	X‐12730 levels	IVW	15	0.827	0.744–0.919	0.000
	X‐13553 levels	IVW	15	1.107	1.012–1.212	0.027
	X‐12844 levels	IVW	15	0.902	0.817–0.996	0.042
	X‐16964 levels	IVW	15	0.896	0.811–0.991	0.032
	X‐17346 levels	IVW	15	0.887	0.794–0.991	0.034
	X‐18779 levels	IVW	15	1.122	1.017–1.237	0.021
	X‐23655 levels	IVW	15	0.874	0.784–0.975	0.015
	X‐23678 levels	IVW	15	0.875	0.769–0.996	0.043
	X‐24546 levels	IVW	15	0.886	0.799–0.983	0.023
	X‐24970 levels	IVW	15	1.152	1.044–1.271	0.005
	X‐24588 levels	IVW	15	0.900	0.820–0.988	0.027
	X‐25419 levels	IVW	15	0.897	0.805–0.999	0.048
	Androsterone sulfate levels	IVW	15	0.902	0.820–0.991	0.032
	Ethylmalonate levels	IVW	15	0.904	0.820–0.996	0.041

**Table 4:** Mendelian randomization analysis on the causal effect of plasma metabolite on Species Bacteroides salyersiae. Number of single‐nucleotide polymorphism (nsnp), Odds ratio (OR), 95% confidence interval (CI), Inverse variance weighted (IVW).

Outcome	Exposure	Method	nsnp	OR	or_lci95	pval
Species Bacteroides salyersiae	Carnitine levels	IVW	8	0.916	0.862–0.973	0.004
	4‐acetylphenol sulfate levels	IVW	8	1.115	1.009–1.232	0.033
	Stachydrine levels	IVW	8	1.073	1.010–1.140	0.023
	4‐hydroxyhippurate levels	IVW	8	1.078	1.017–1.144	0.012
	Hydroquinone sulfate levels	IVW	8	1.077	1.011–1.147	0.021
	Alpha‐hydroxycaproate levels	IVW	8	0.907	0.839–0.980	0.014
	N‐methylproline levels	IVW	8	1.064	1.001–1.131	0.047
	5alpha‐androstan‐3alpha,17beta‐diol monosulfate (1) levels	IVW	8	0.929	0.873–0.988	0.020
	2‐hydroxyhippurate levels	IVW	8	1.081	1.013–1.154	0.018
	Dihydroferulate levels	IVW	8	1.088	1.015–1.166	0.017
	2‐hydroxydecanoate levels	IVW	8	1.075	1.012–1.142	0.019
	3‐(3‐hydroxyphenyl)propionate sulfate levels	IVW	8	1.098	1.021–1.182	0.012
	Sphingomyelin (d18:2/14:0, d18:1/14:1) levels	IVW	8	1.051	1.000–1.104	0.050
	Methyl‐4‐hydroxybenzoate sulfate levels	IVW	8	1.064	1.003–1.128	0.040
	1‐stearoyl‐2‐docosahexaenoyl‐gpc (18:0/22:6) levels	IVW	8	0.923	0.870–0.978	0.007
	1‐oleoyl‐2‐docosahexaenoyl‐GPC (18:1/22:6) levels	IVW	8	0.943	0.890–1.000	0.048
	Ceramide (d18:1/14:0, d16:1/16:0) levels	IVW	8	1.068	1.001–1.139	0.047
	Heneicosapentaenoate (21:5n3) levels	IVW	8	0.924	0.854–0.999	0.048
	Docosahexaenoylcarnitine (C22:6) levels	IVW	8	0.908	0.833–0.990	0.028
	N,N,N‐trimethyl‐5‐aminovalerate levels	IVW	8	0.922	0.864–0.983	0.013
	Dihydroferulic acid sulfate levels	IVW	8	1.131	1.047–1.221	0.002
	Butyrate/isobutyrate (4:0) levels	IVW	8	1.073	1.005–1.146	0.034
	Gamma‐glutamylhistidine levels	IVW	8	1.066	1.003–1.134	0.041
	Anthranilate levels	IVW	8	1.079	1.004–1.160	0.039
	Histidine levels	IVW	8	1.089	1.015–1.167	0.017
	Arachidate (20:0) levels	IVW	8	1.067	1.003–1.134	0.038
	X‐07765 levels	IVW	8	0.935	0.879–0.993	0.030
	X‐11444 levels	IVW	8	0.939	0.887–0.994	0.030
	X‐14939 levels	IVW	8	0.932	0.874–0.993	0.031
	X‐18901 levels	IVW	8	1.063	1.001–1.129	0.048
	X‐18913 levels	IVW	8	1.076	1.013–1.143	0.018
	X‐19299 levels	IVW	8	0.892	0.831–0.958	0.002
	X‐21742 levels	IVW	8	1.112	1.033–1.196	0.005
	X‐24757 levels	IVW	8	1.103	1.028–1.184	0.006
	X‐24795 levels	IVW	8	1.107	1.012–1.212	0.027
	X‐24947 levels	IVW	8	0.919	0.865–0.977	0.007
	X‐25828 levels	IVW	8	0.937	0.880–0.998	0.042
	Androsterone sulfate levels	IVW	8	0.939	0.886–0.996	0.035

### 3.3 MR Analysis of Significant Plasma Metabolites on BCa

Subsequently, we investigated the causal relationships between 148 plasma metabolites and BCa. Our findings demonstrated that N‐palmitoyl‐sphinganine levels (OR = 0.83, 95% CI: 0.70–0.98, *p*‐value = 0.032), 2,3‐dihydroxypyridine levels (OR = 0.77, 95% CI: 0.64–0.94, *p*‐value = 0.010), Anthranilate levels (OR = 0.88, 95% CI: 0.78–0.99, *p*‐value = 0.041), and X‐19299 levels (OR = 0.73, 95% CI: 0.57–0.94, *p*‐value = 0.016) were negatively associated with BCa, while N‐methylproline levels (OR = 1.25, 95% CI: 1.02–1.53, *p*‐value = 0.025) were positively associated with BCa (Table S4). Additionally, no heterogeneity or pleiotropy were observed in our findings (Figure S9–S10, Table S4).

### 3.4 Mediation Effect of Gut Microbiota on BCa

**Figure 3:**
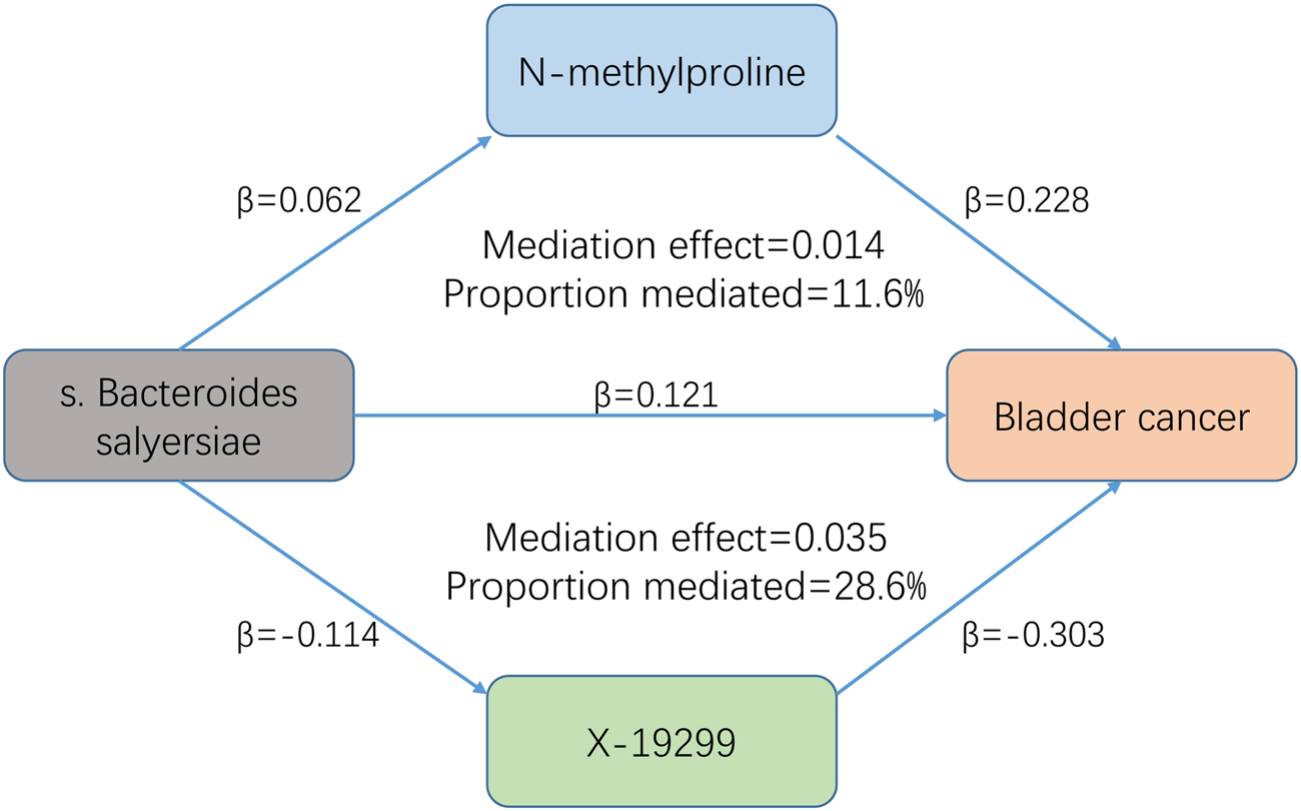
Mediation effect of Species Bacteroides salyersiae on BCa via N‐methylproline and X19299.

Our mediation analysis observed that the Genus Streptococcus and Species Bacteroides dorei mediated their effects on BCa through N‐palmitoyl‐sphinganine levels and 2,3‐dihydroxypyridine levels, with mediation effects of −0.017 and 0.030, respectively. The mediation effect of N‐methylproline levels from Species Bacteroides salyersiae on BCa was 0.014, accounting for 11.6% of the total effect, while that of X‐19299 levels was 0.035, accounting for 28.6% of the total effect (Figure 3). Furthermore, Species Bacteroides salyersiae could affect BCa by Anthranilate levels (mediation effect = −0.009) (Table 5).

**Table 5:** Mediation effect of GM on BCa via plasma metabolites. β1 representing the effect of gut microbiota on the plasma metabolites, β2 indicating the effect of plasma metabolites on BCa, and β3 referring to the effect of gut microbiota on BCa, gut microbiota (GM), bladder cancer (BCa).

Outcome		Exposure	Mediator	Total effect (β3)	β1		β2	Mediation effect	Mediation proportion
Bca	Genus Streptococcus		N‐palmitoyl‐sphinganine	0.232		0.094	−0.181	−0.017	
	Species Bacteroides dorei		2,3‐dihydroxypyridine	−0.430		−0.120	−0.253	0.030	
	Species Bacteroides salyersiae		N‐methylproline	0.121		0.062	0.228	0.014	11.60%
			Anthranilate	0.121		0.076	−0.124	−0.009	
			X‐19299	0.121		−0.114	−0.303	0.035	28.60%

## 4 Discussion

In recent years, the role of gut microbiota in cancers has attracted significant attention. GM have been shown to influence tumor through various mechanisms, including regulation of host immune response, influence of metabolites, and regulation of inflammatory response. However, existing research on the impact of GM in cancers has predominantly focused on intestinal tract cancers, leaving the effect of GM in extraintestinal cancers and their underlying mechanisms unexplored. This study pioneered the utilization of a two‐step MR approach to elucidate the potential causal relationships between GM and BCa, as well as the mediation effect of plasma metabolites. These findings contribute to a better understanding of the pathophysiological mechanism of tumor development and provide new insights into future tumor treatment and prevention strategies.

Our study identified a negative association between Species Bacteroides dorei and BCa, indicating a potential protective effect against BCa. Species Bacteroides dorei, characterized as gram‐negative rods, anaerobic, nonmotile and non‐spore‐forming, play a vital role in maintaining normal intestinal physiology and function and was the most significant gut microbiota taxa associated with BCa in our study [30]. Previous studies have linked Bacteroides dorei to various diseases, including liver cirrhosis [31], atherosclerosis [32], type 1 diabetes [33], polycystic ovary syndrome [34] and tumors, especially tumor immunotherapy [35, 36, 37]. The relative abundance of Bacteroides dorei was found to be significantly higher in primary colorectal tumors [35], with major pathological response to neoadjuvant therapy for young‐onset rectal cancers associated with Bacteroides dorei [38]. In addition, Bacteroides dorei could predict the overall survival of immune checkpoint inhibitor (ICI)‐treated non‐small cell lung cancer (NSCLC) patients [36], and was associated with immune‐related adverse events in patients with advanced stage melanoma treated with ICI [37]. Our study revealed that Species Bacteroides dorei could potentially influence BCa through 2,3‐dihydroxypyridine(2,3‐DHP). 2,3‐DHP is a metabolite of mimosine, which has been identified as toxic to animals [39]. It dampens thyroid function, leading to a significant increase in serum thyrotropin (TSH) levels and a decrease in serum triiodothyronine (T3) levels in rats. Additionally, 2,3‐DHP inhibits Catechol‐O‐methyltransferase (COMT) activity [39, 40]. Despite these known effects, the role of 2,3‐DHP in tumors remains unexplored and warrants further investigation.

The Genus Streptococcus was positively correlated with an increased risk of BCa. It comprises a wide variety of pathogenic and commensal gram‐positive bacteria, some of which have been considered to play controversial roles in the development and progression of tumors. Studies have reported an increased abundance of Streptococcus in diverse cancers, including colorectal cancer [41], gastric cancer [42], pancreatic ductal adenocarcinoma [43], breast cancer [44], multiple myeloma [45], esophageal squamous cell carcinoma and lung cancer [46]. However, conflicting findings have been observed, with a reduction of Genus Streptococcus in oral squamous cell carcinoma (OSCC) groups compared to normal controls [47]. Streptococcus has been associated with positive responses to anti‐PD‐1/PD‐L1 therapy across different gastrointestinal cancer types, facilitating recruitment of tumor‐infiltrating CD8^+^T cells [48]. Streptococcus thermophilus has demonstrated tumor‐suppressive effects through the production of galactose, modulating oxidative phosphorylation and the Hippo signaling pathway [49]. Moreover, Jian et al. proposed that Streptococcus is significantly correlated with the host metabolome, suggesting strong metabolic interactions between microbes and the host. Our study suggests that Genus Streptococcus might affect BCa through N‐palmitoyl‐sphinganine, a ceramide present in human hair, which is associated with ectopic fat accumulation and granulocyte differentiation [50, 51, 52]. While the role of N‐palmitoyl‐sphinganine in tumors remains unclear, certain strains of Streptococcus may possess inherent antitumor properties, while others can trigger the host immune response against tumors [53]. This highlights the potential of Streptococcus as both targeted and immunostimulatory approaches for cancer treatment.

Species Bacteroides salyersiae was another risk factor for BCa in our MR analysis. Belonging to the Genus Bacteroides, Bacteroides salyersiae, along with Bacteroides dorei, were prominent constituents of the human gut microbiota, playing crucial roles in the metabolism of complex carbohydrates [54]. Previous studies have identified that Species Bacteroides salyersiae is involved in Alzheimer's disease [55], diffuse large‐B cell lymphoma [56] and immunotherapy resistance in renal cell carcinoma [57]. Using Mendelian randomization, Xie et al. reported a negative relationship between Bacteroides salyersiae and PCa [58], which is in contrast to our results. This might stem from the inherent heterogeneity across different tumors. Our analysis identified three metabolites that mediated the interaction between GM and BCa: X‐19299, N‐methylproline and Anthranilate. X‐19299, the most recently identified metabolite in Chen's study, accounts for over a quarter of the causal association between GM and BCa [21], underscoring its significance in the promotion of BCa by Bacteroides salyersiae. N‐methylproline could be released by GM consuming proline betaine, which has been proven to have cardiovascular activity [59]. And anthranilate, derived from the catabolism of kynurenine by kynureninase, was upregulated in correlation with the prostate‐specific antigen (PSA) level of prostate cancer patients [60] and was supported to promote tumor immune infiltration [61]. Further exploration of Bacteroides salyersiae and its metabolites holds promise for the development of novel treatment strategies for BCa.

However, this study has several limitations. First, our analysis was primarily performed in European populations, and it is necessary to validate the conclusions in other populations. Second, we collected BCa cases from public database with a sample size of 2053, which might introduce bias into the results. Third, we observed that the effects of Species Bacteroides dorei, Genus Streptococcus and Species Bacteroides salyersiae on BCa were mediated by 2,3‐dihydroxypyridine, N‐palmitoyl‐sphinganine and anthranilate, respectively. However, the mediation effect of these metabolites on BCa was the opposite of the total effect of GM on BCa, suggesting the presence of other mediators that require further investigation. Fourth, our analysis inherits the inherent limitation of metabolite profiling in the underlying GWAS datasets. The single‐timepoint measurements cannot capture intra‐individual metabolic variation over time, and may be influenced by acute physiological states (e.g., fasting status, circadian rhythms, or transient environmental exposures). While this limitation is partially mitigated by the large sample sizes of the source GWAS, future studies incorporating longitudinal metabolite data or repeated measurements may better account for temporal biological variation. Finally, although the mediation effect in our study have undergone rigorous steps and screening through MR, which accounts for approximately 11.6%–28.6% of the total effect. Considering that biological processes are complex, extensive research is still needed to prove the specific mechanisms discussed above.

## 5 Conclusion

In conclusion, our study illustrates causal relationships between GM, plasma metabolites, and BCa. Specifically, Species Bacteroides salyersiae was demonstrated to increase the risk of BCa, mediated by N‐methylproline and X‐19299 with mediation proportions of 11.6% and 28.5%, respectively. Moreover, Species Bacteroides dorei, Genus Streptococcus Species and Bacteroides salyersiae were found to affect BCa through 2,3‐dihydroxypyridine, N‐palmitoyl‐sphinganine and N‐methylproline, respectively. These findings provide genetic evidence supporting the associations between gut microbiota, plasma metabolites, and BCa, thus providing potential novel treatment strategies for BCa.

## Author Contributions

**Kai Che:** writing – original draft, writing – review and editing, conceptualization, methodology, investigation. **Dong Qian:** writing – review and editing, conceptualization, methodology, software, data curation, formal analysis, project administration. **Shuxia Cui:** investigation, validation, writing – review and editing, visualization. **Fei Xie:** conceptualization, formal analysis, software, supervision, writing – review and editing, funding acquisition, resources.

## Ethics Statement

This analysis of publicly available data does not require ethical approval.

## Conflicts of Interest

No conflict of interest exists in the submission of this manuscript, and the manuscript is approved by all authors for publication.

## Transparency Statement

The lead author Fei Xie affirms that this manuscript is an honest, accurate, and transparent account of the study being reported; that no important aspects of the study have been omitted; and that any discrepancies from the study as planned (and, if relevant, registered) have been explained.

## Supporting information


**Figure S1:** Funnel plots for MR causal effects of gut microbiota on Bca.

**Figure S2:** Leave‐one‐out analysis for MR causal effects of gut microbiota on Bca.

**Figure S3:** Funnel plots for MR causal effects of plasma metabolites on g_Streptococcus.

**Figure S4:** Leave‐one‐out analysis for MR causal effects of plasma metabolite on g_Streptococcus.

**Figure S5:** Funnel plots for MR causal effects of plasma metabolites on s_Bacteroides_dorei.

**Figure S6:** Leave‐one‐out analysis for MR causal effects of plasma metabolites on s_Bacteroides_dorei.

**Figure S7:** Funnel plots for MR causal effects of plasma metabolites on s_Bacteroides_salyersiae.

**Figure S8:** Leave‐one‐out analysis for MR causal effects of plasma metabolites on s_Bacteroides_salyersiae.

**Figure S9:** Funnel plots for MR causal effects of significantly plasma metabolites on Bca.

**Figure S10:** Leave‐one‐out analysis for MR causal effects of significantly plasma metabolites on Bca.

supplementary table.

supmat.

## Data Availability

The data that support the findings of this study are openly available in [Dutch Microbiome Project] at [10.1038/s41588‐021‐00992‐y], reference number [20], in [Canadian Longitudinal Study of Aging (CLSA) follows] at [10.1038/s41588‐022‐01270‐1], reference number [21] and in [FinnGen Biobank] at [10.1038/s41586‐022‐05473‐8], reference number [22]. The corresponding author, Fei Xie, had full access to all of the data in this study and takes complete responsibility for the integrity of the data and the accuracy of the data analysis. These data were derived from the following resources available in the public domain: the Dutch Microbiome Project (https://dutchmicrobiomeproject.molgeniscloud.org/), the Canadian Longitudinal Study of Aging (CLSA) follows (https://www.ebi.ac.uk/gwas/studies/GCST90199621-902010209), and FinnGen Biobank (https://www.finngen.fi).
