# Supplementary material for: Dissecting Causal Relationships Between Gut Microbiota, Plasma Metabolites and Bladder Cancer: A Two‐Step Mendelian Randomization Study
Source: Health Sci Rep. 2025 Sep 9;8(9):e71206. doi: 10.1002/hsr2.71206 (PMC12420358; doi:10.1002/hsr2.71206)
Supplement: Supplementary file 1 — Figure S1: Funnel plots for MR causal effects of gut microbiota on Bca. [file HSR2-8-e71206-s010.pdf]

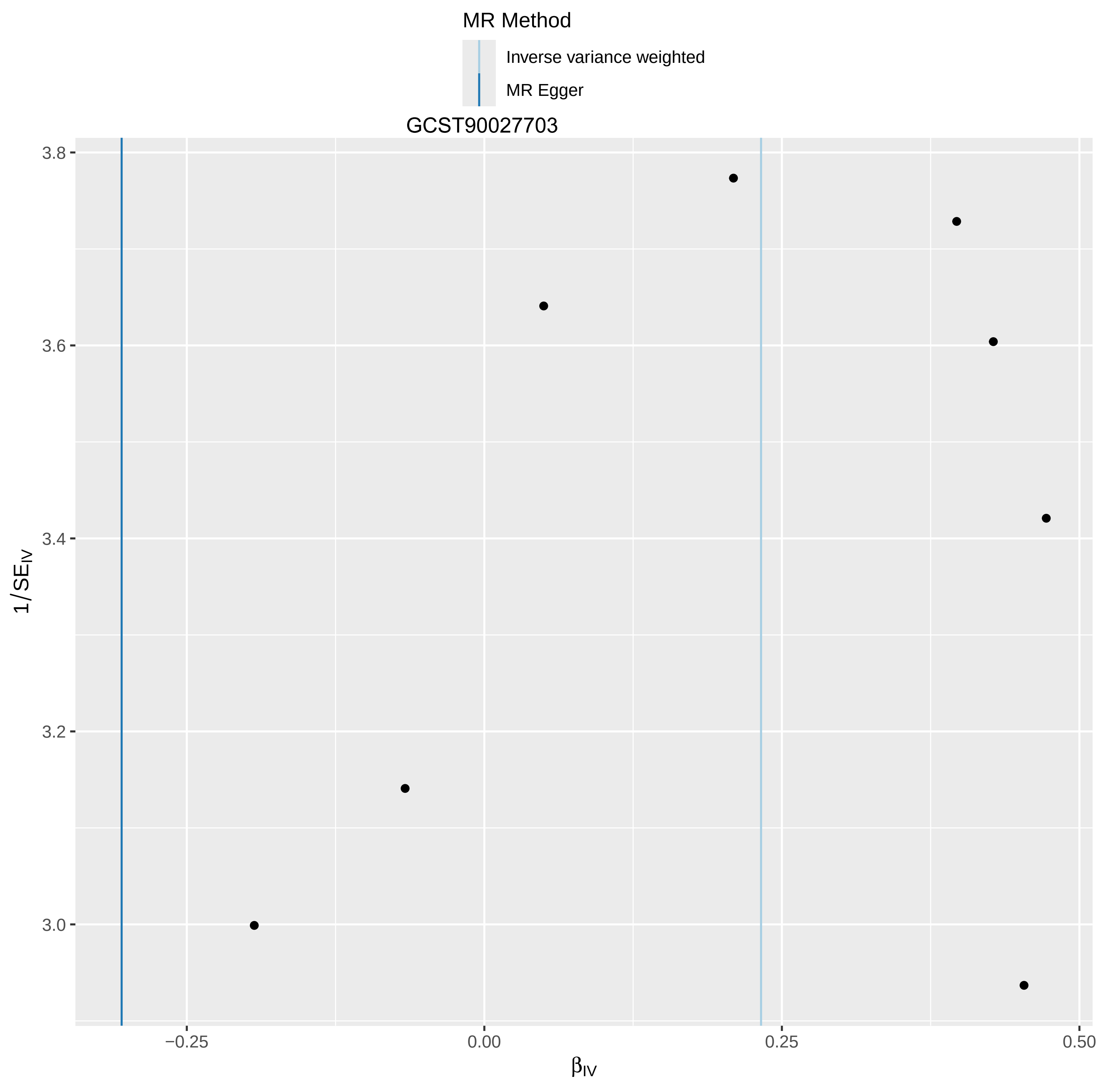
MR Method
Inverse variance weighted
MR Egger
GCST90027703
3.8
3.6
3.4
3.2
3.0
1/SE_IV
−0.25
0.00
0.25
0.50
β_IV

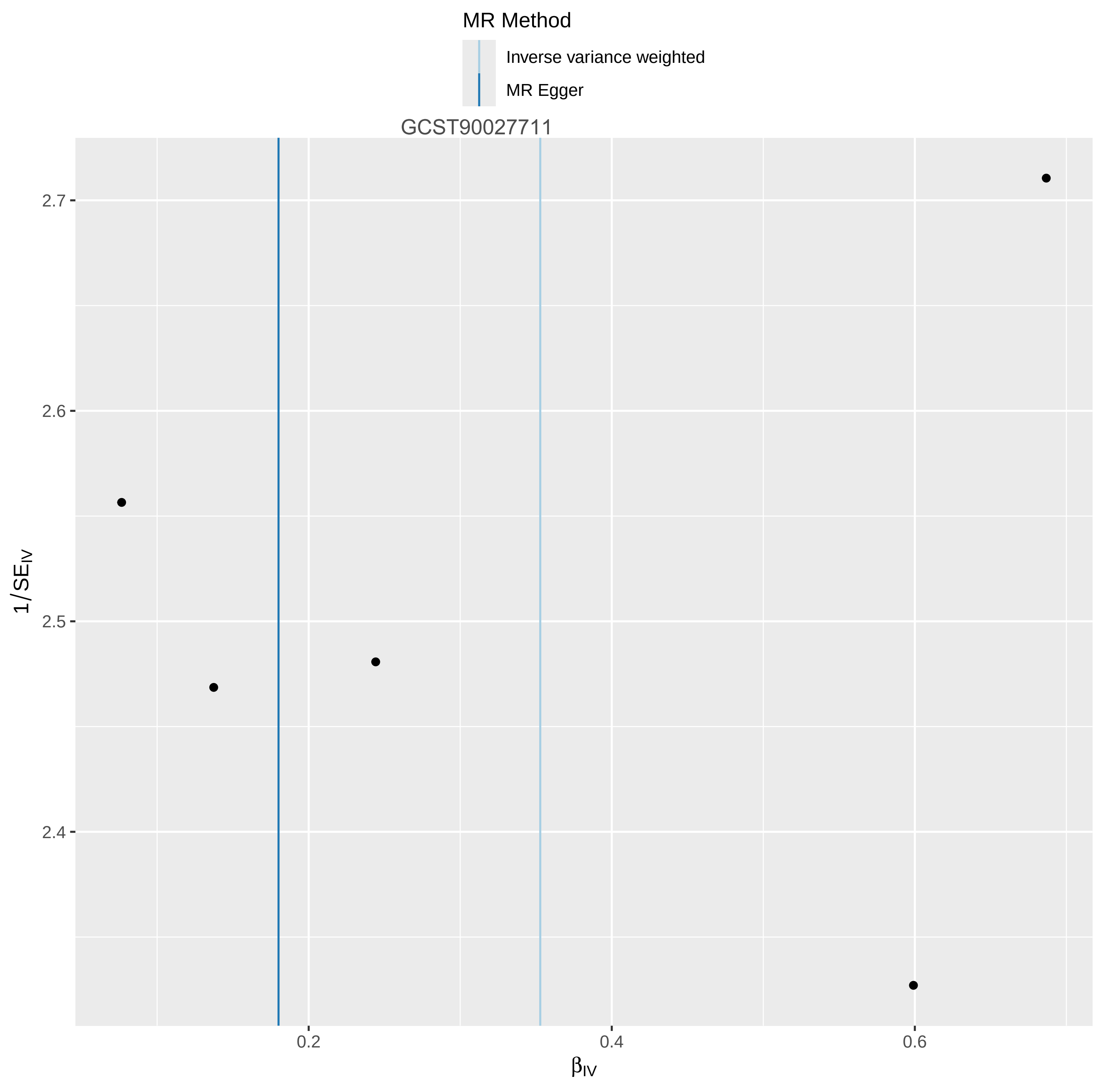
MR Method
Inverse variance weighted
MR Egger
GCST90027711
2.7
2.6
2.5
2.4
$1/SE_{IV}$
0.2
0.4
0.6
β_{IV}

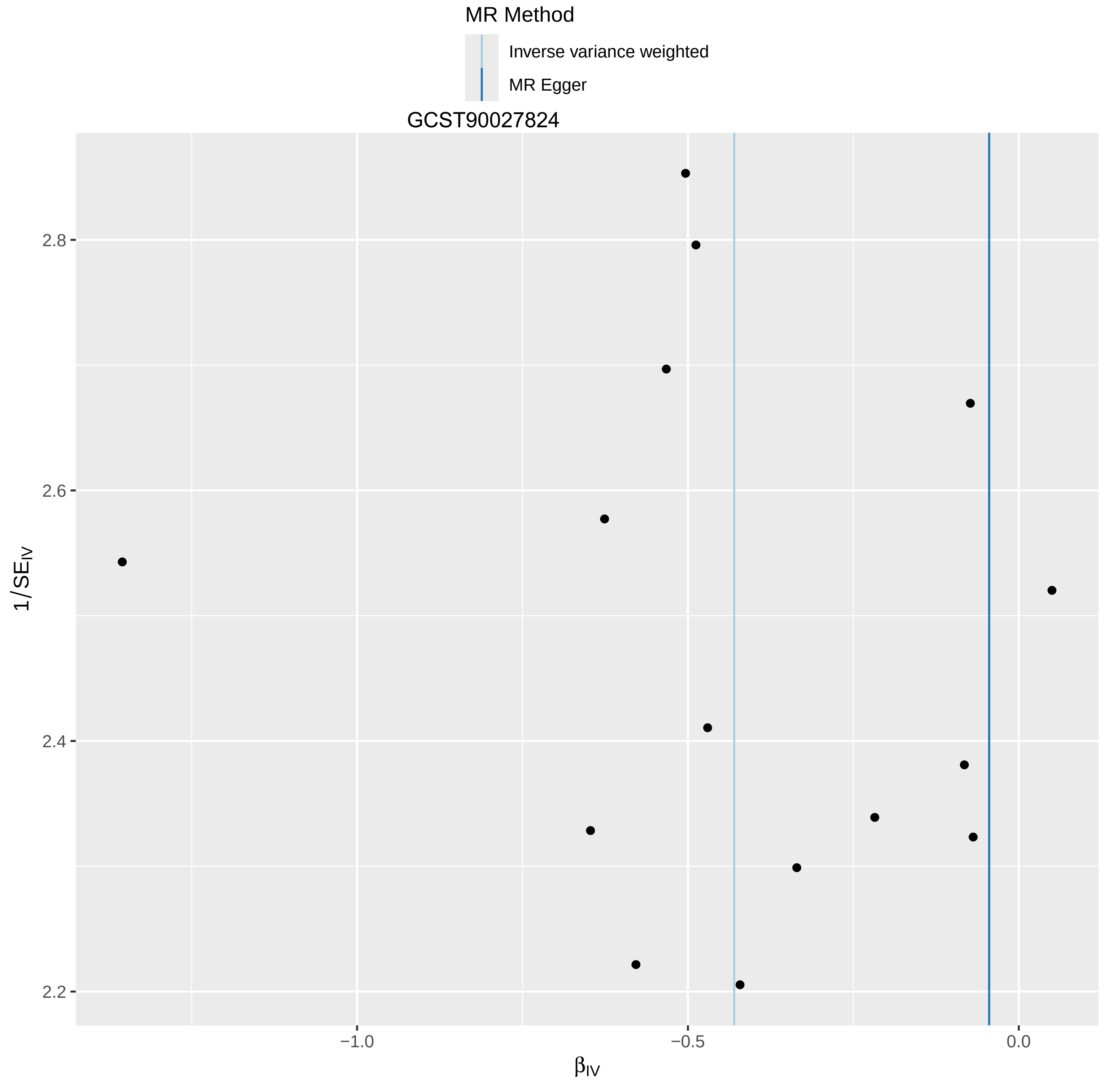

MR Method
Inverse variance weighted
MR Egger
GCST90027824
1/SE_IV
2.2
2.4
2.6
2.8
−1.0
−0.5
0.0
β_IV

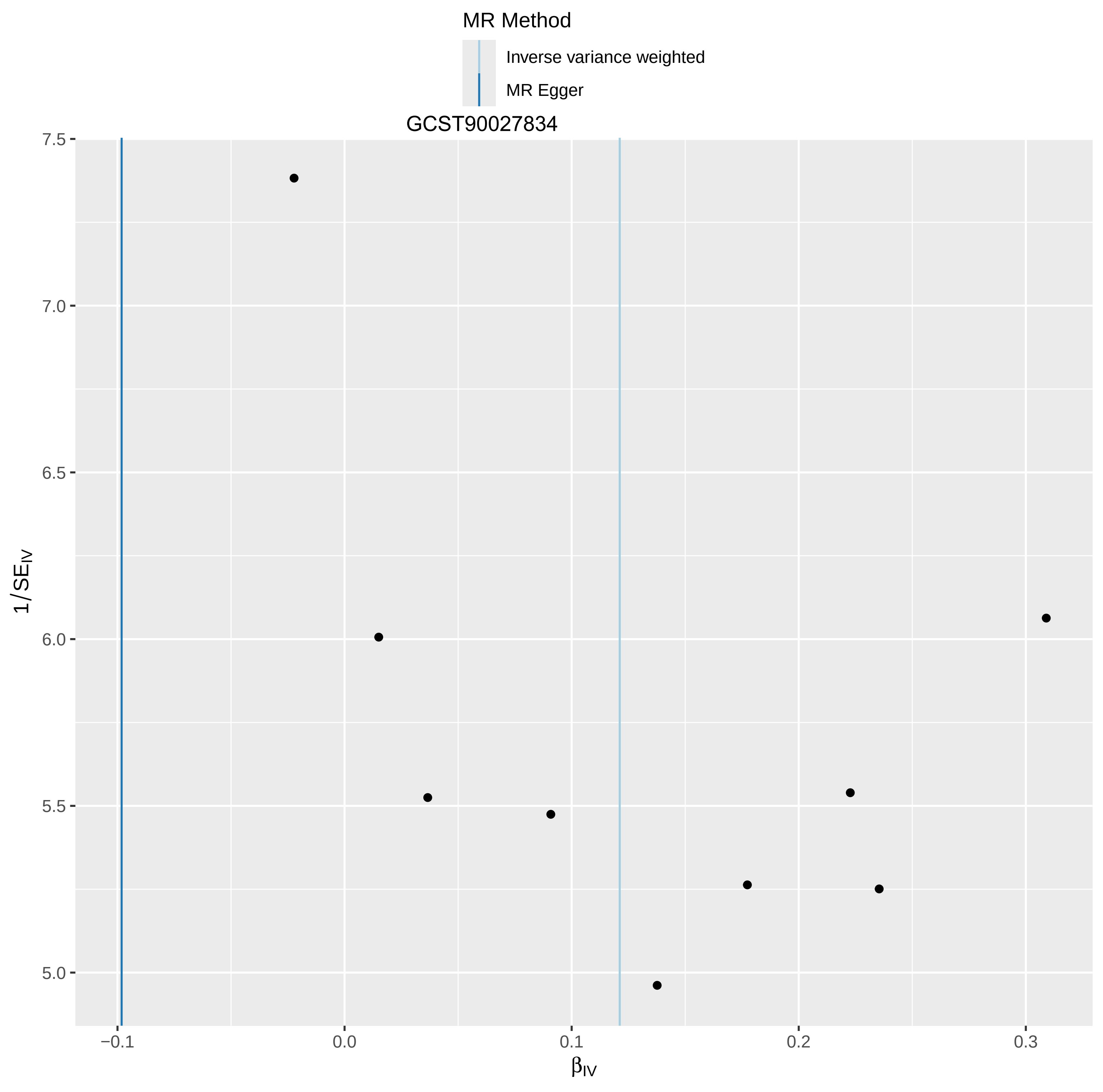

MR Method
Inverse variance weighted
MR Egger
GCST90027834
7.5
7.0
6.5
6.0
5.5
5.0
1/SE$_{IV}$
−0.1
0.0
0.1
0.2
0.3
β$_{IV}$
