# Supplementary figures and images for: Dissecting Causal Relationships Between Gut Microbiota, Plasma Metabolites and Bladder Cancer: A Two‐Step Mendelian Randomization Study
Source: Health Sci Rep. 2025 Sep 9;8(9):e71206. doi: 10.1002/hsr2.71206 (PMC12420358; doi:10.1002/hsr2.71206)

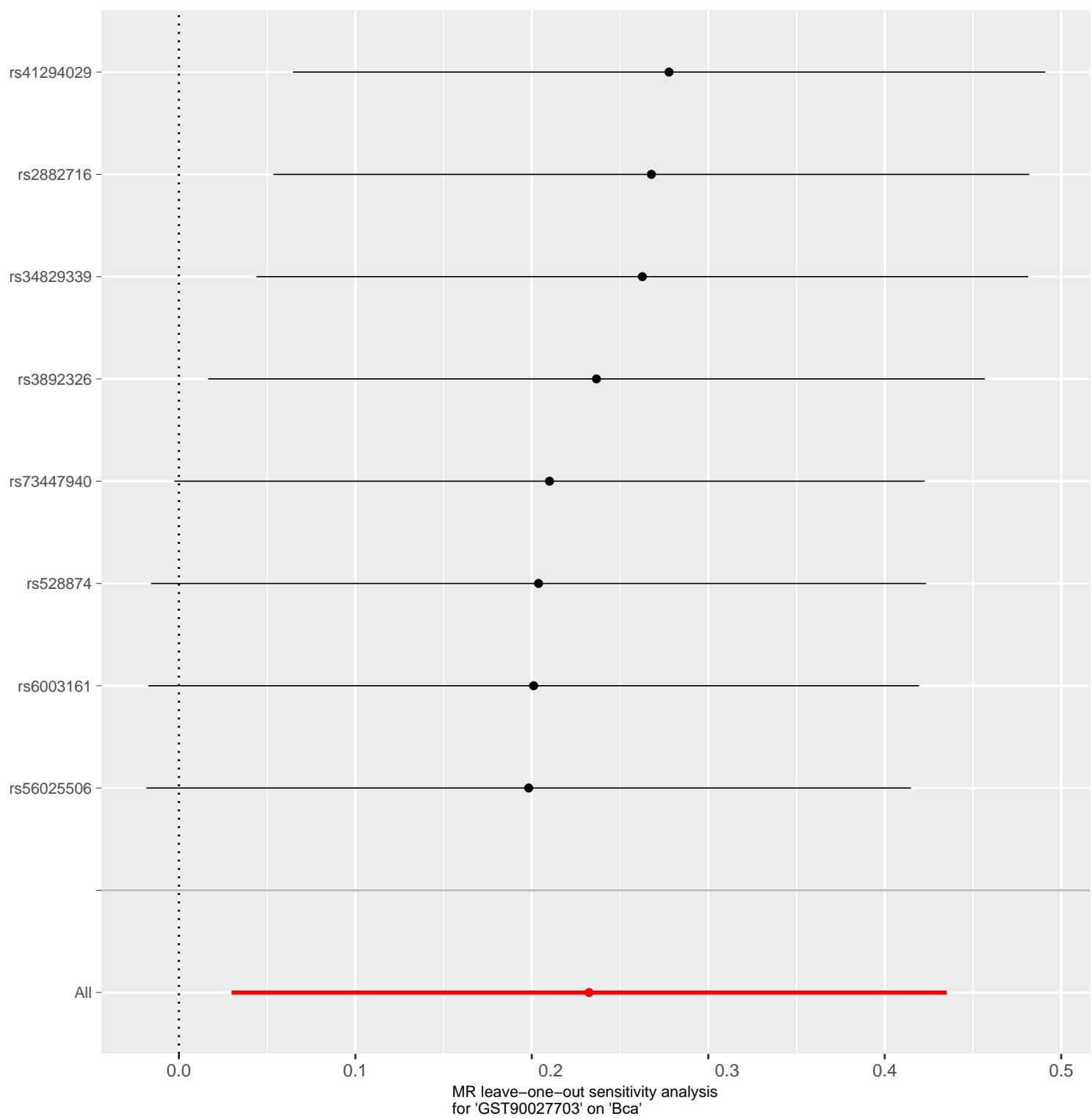

rs7479452

rs74448283

rs56398621

rs12541342

rs404634

All

0.0

0.2

0.4

0.6

0.8

MR leave-one-out sensitivity analysis  
for 'GST90027711' on 'Bca'

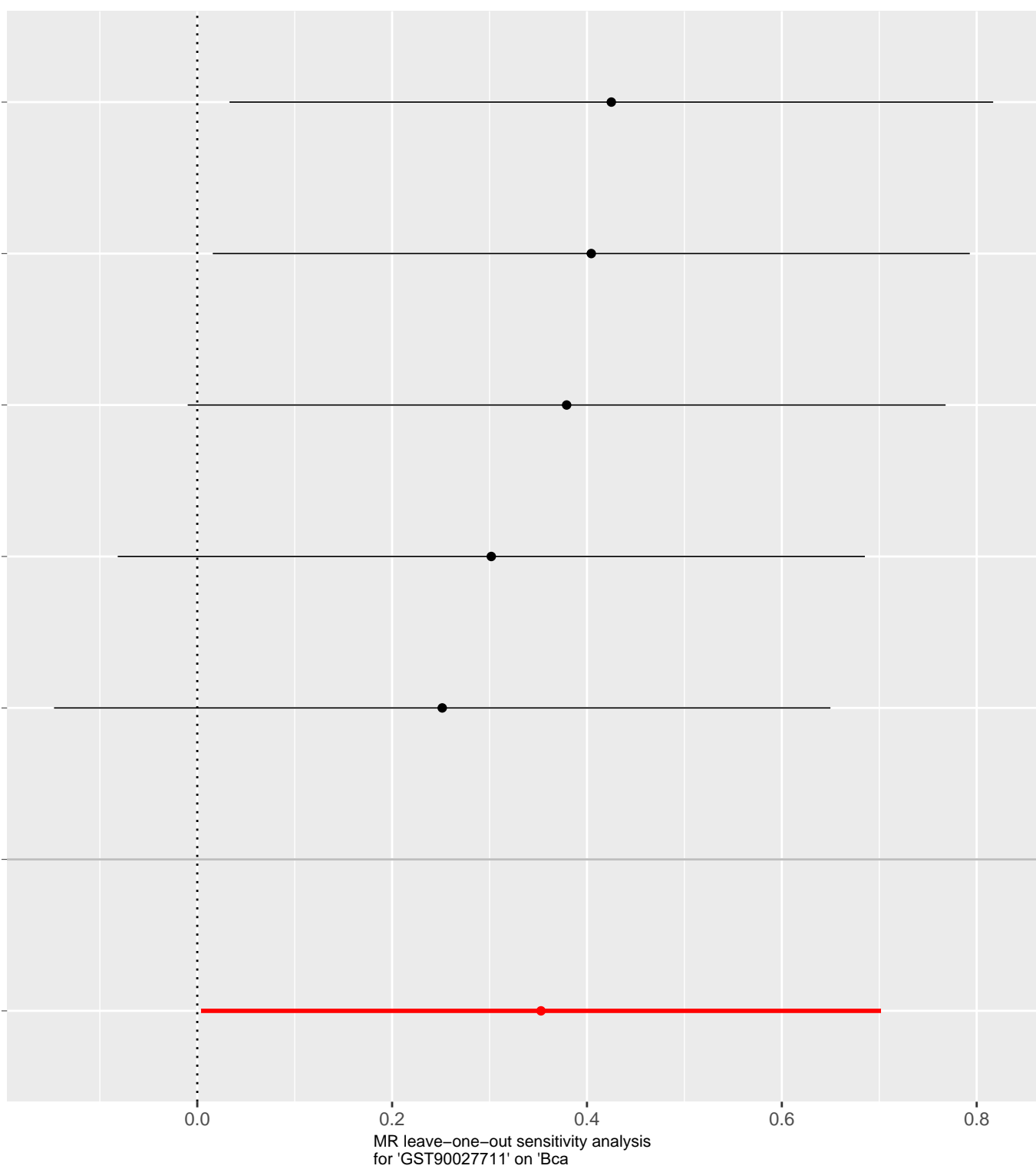

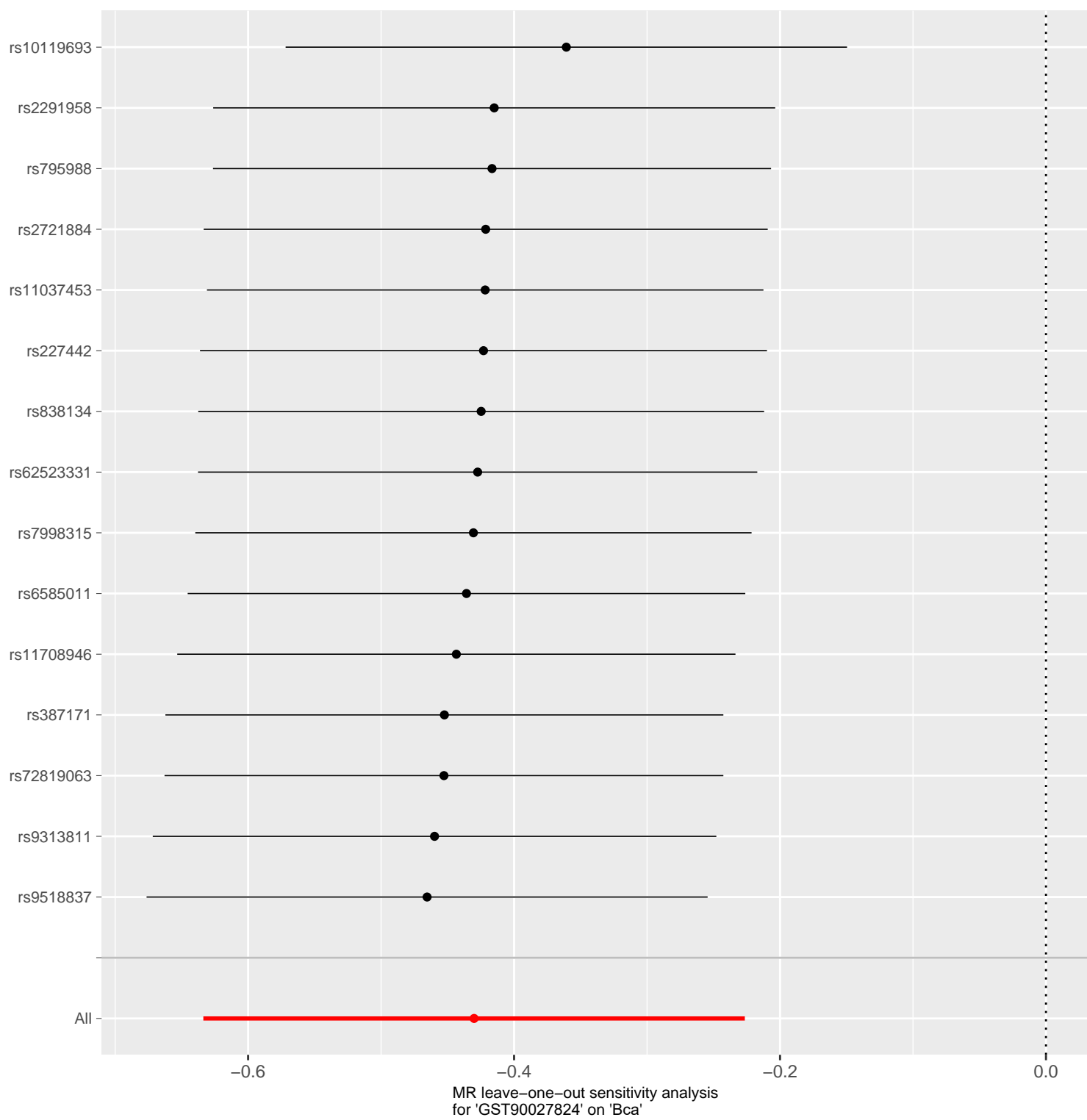

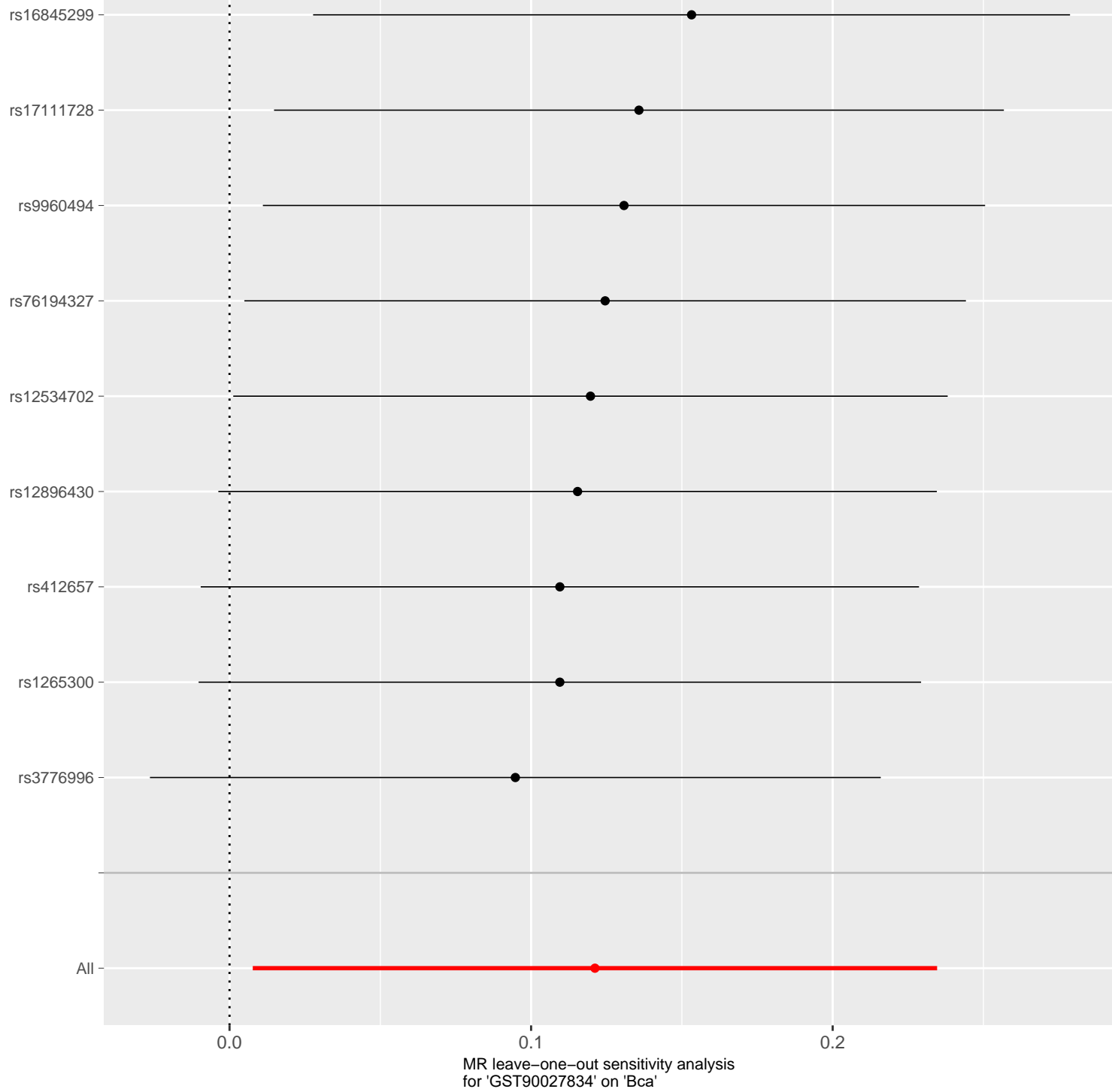

Supplement: Supplementary file 2 — Figure S2: Leave‐one‐out analysis for MR causal effects of gut microbiota on Bca. [file HSR2-8-e71206-s012.pdf]

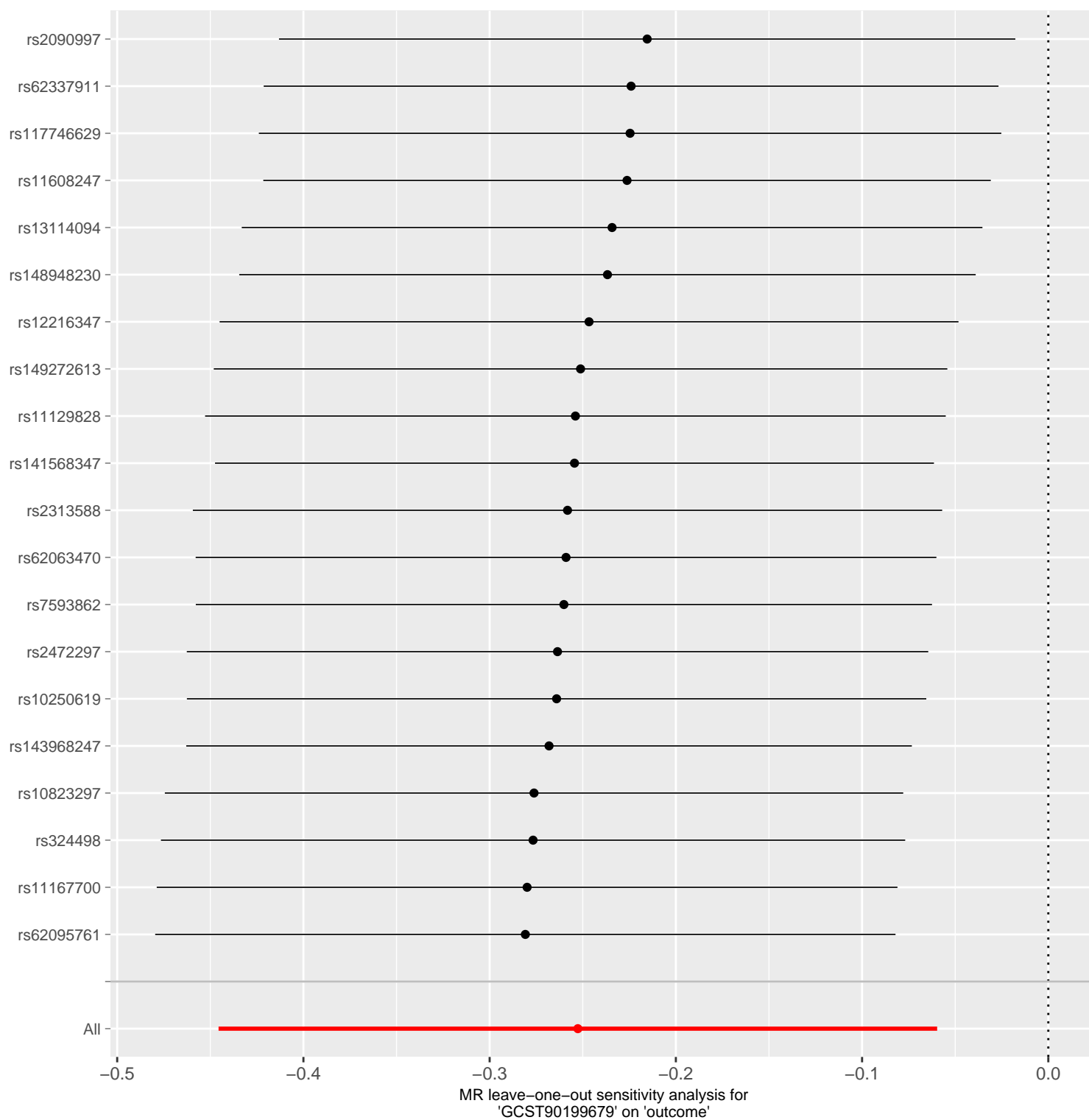

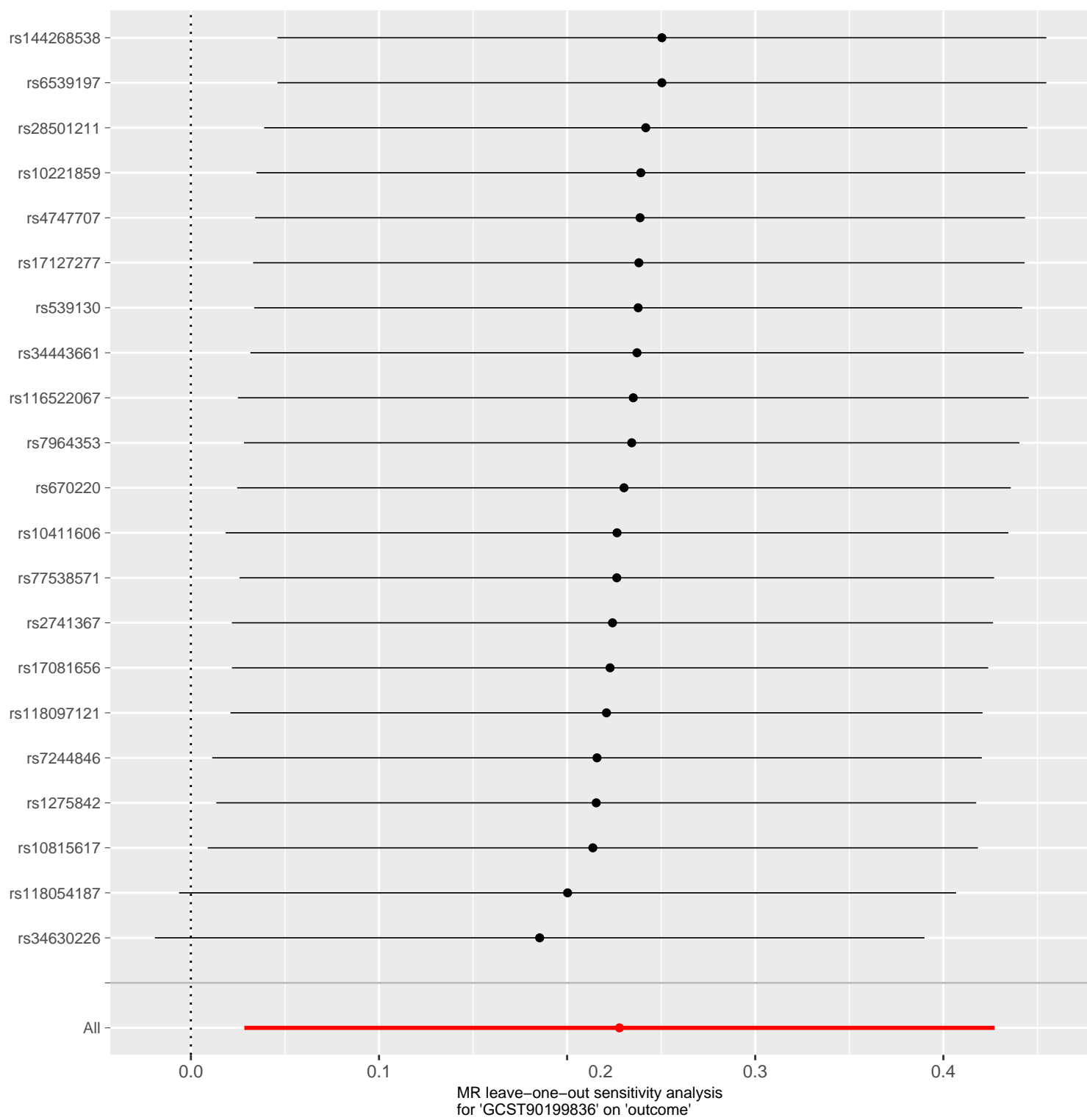

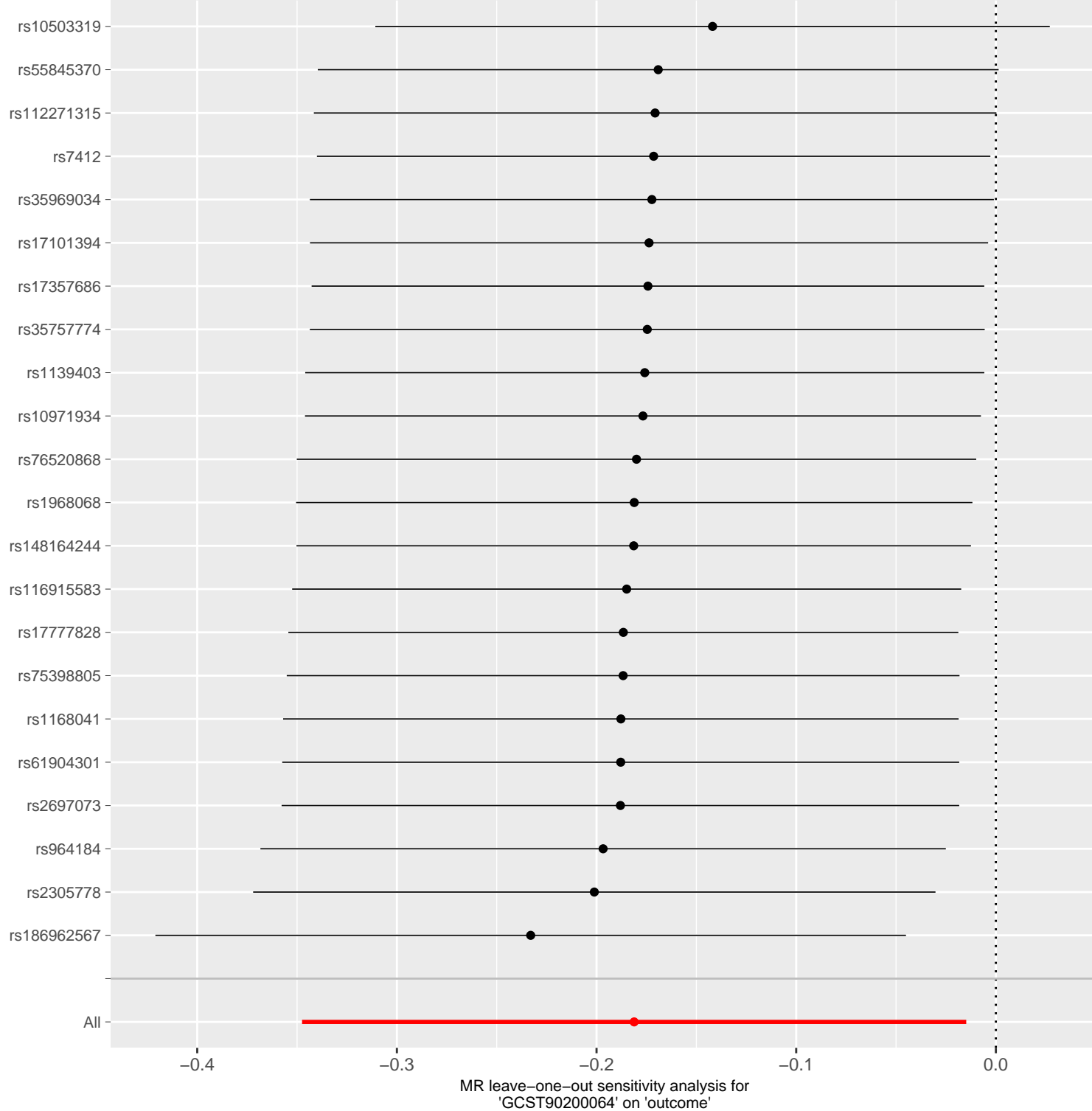

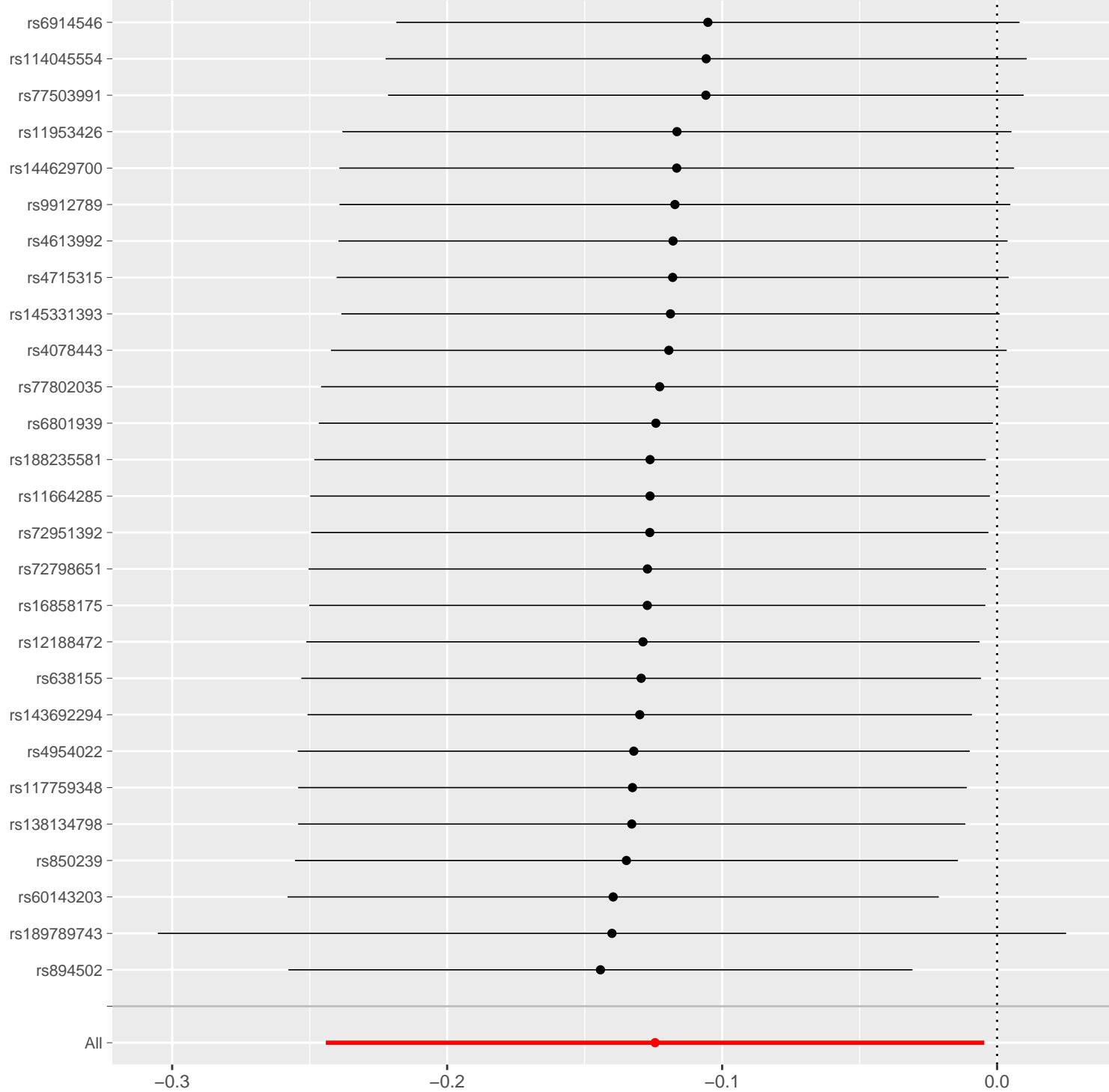

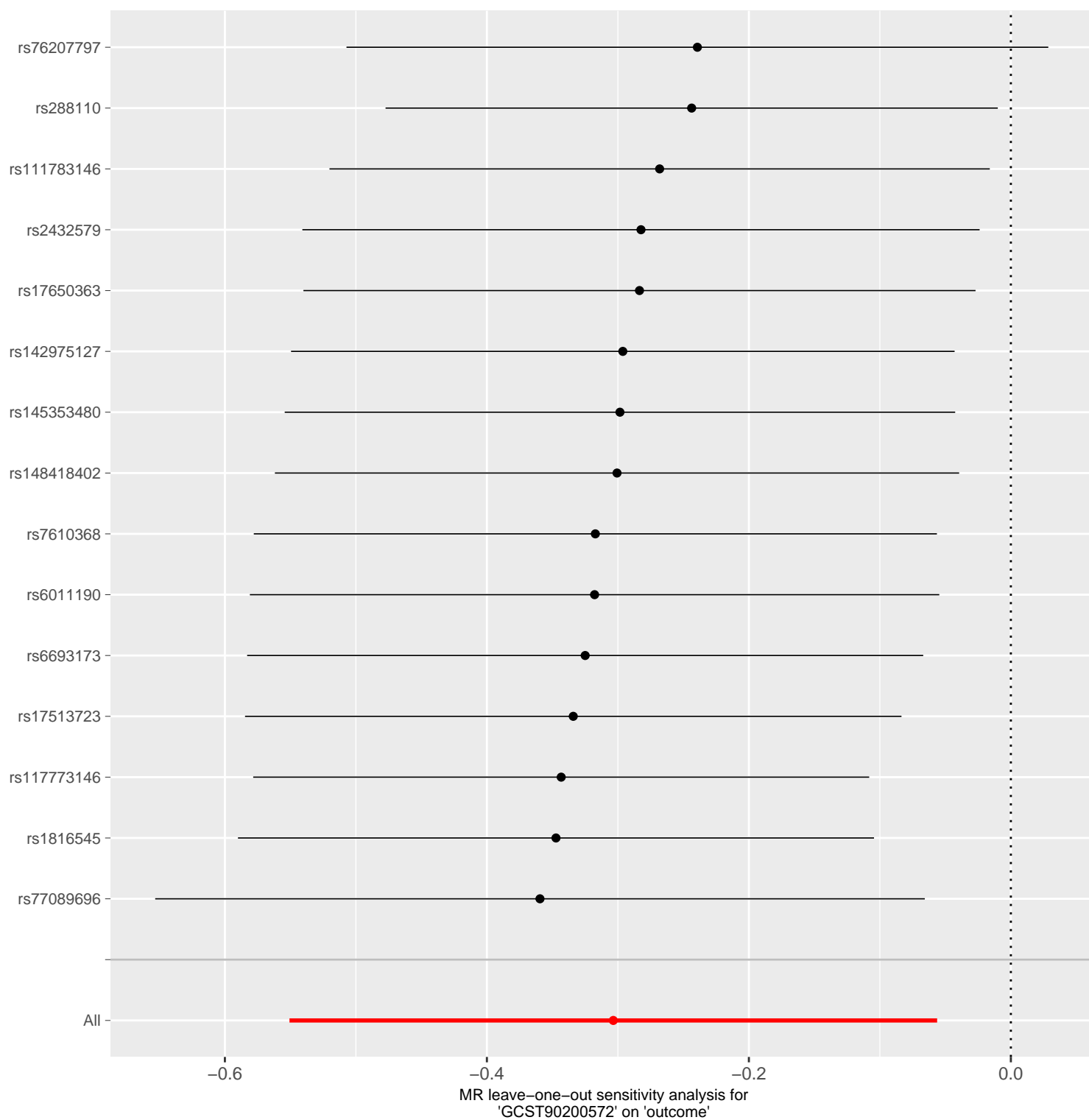

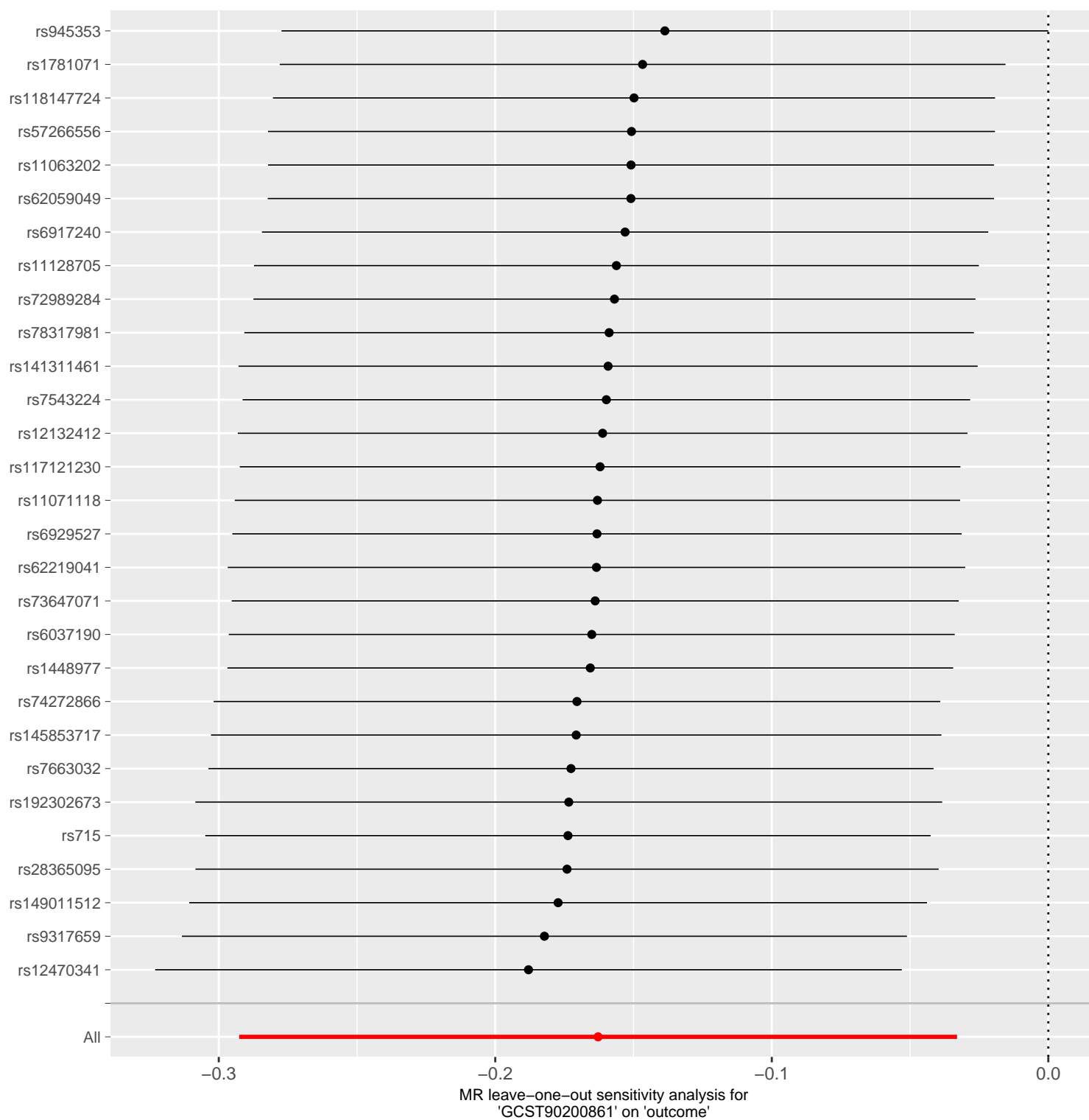

Supplement: Supplementary file 10 — Figure S10: Leave‐one‐out analysis for MR causal effects of significantly plasma metabolites on Bca. [file HSR2-8-e71206-s004.pdf]
