# Supplementary material for: Dissecting Causal Relationships Between Gut Microbiota, Plasma Metabolites and Bladder Cancer: A Two‐Step Mendelian Randomization Study
Source: Health Sci Rep. 2025 Sep 9;8(9):e71206. doi: 10.1002/hsr2.71206 (PMC12420358; doi:10.1002/hsr2.71206)
Supplement: Supplementary file 3 — Figure S3: Funnel plots for MR causal effects of plasma metabolites on g_Streptococcus. [file HSR2-8-e71206-s011.pdf]

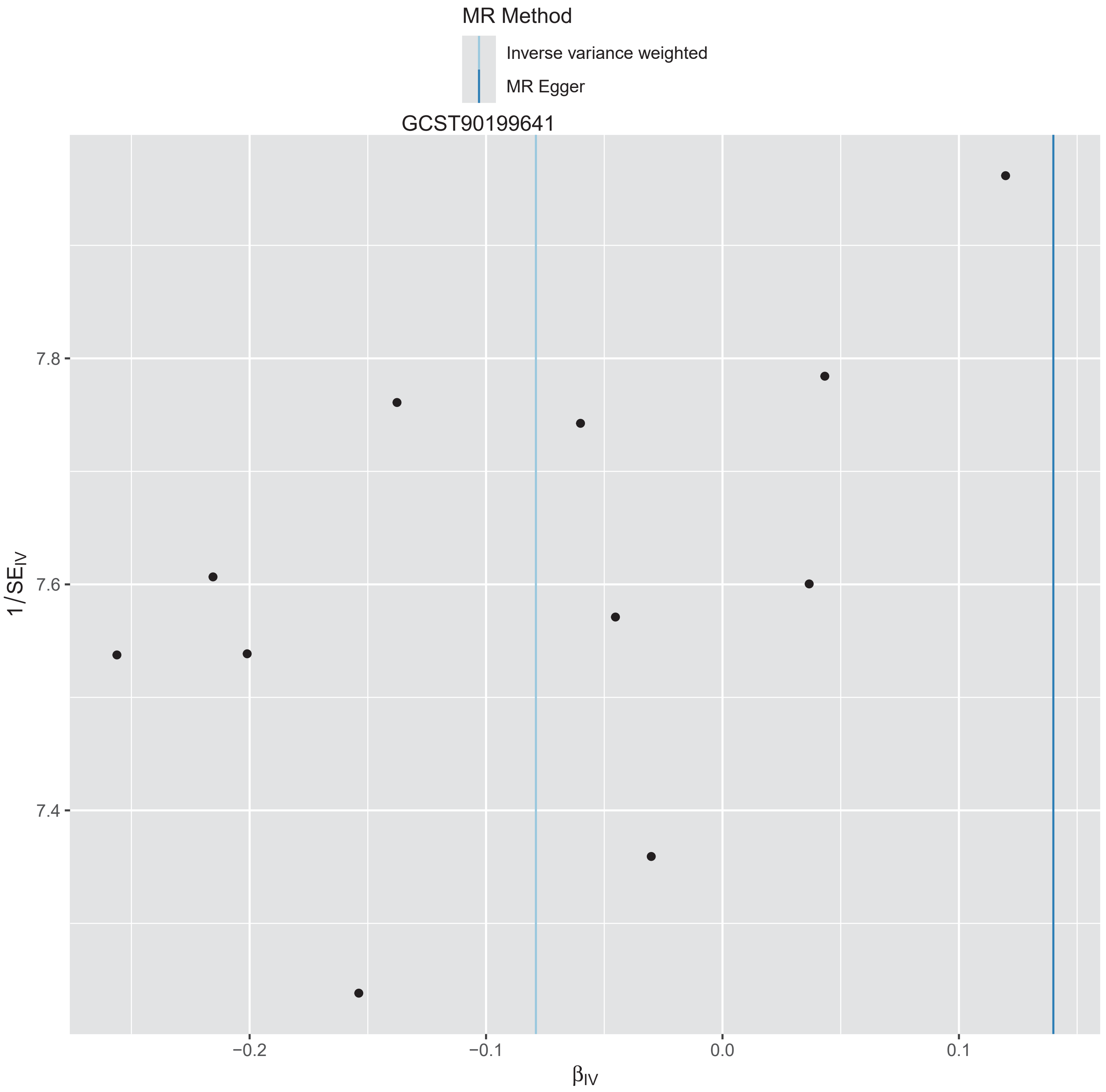

MR Method
Inverse variance weighted
MR Egger
GCST90199641
7.8
7.6
7.4
$1/SE_{IV}$
−0.2
−0.1
0.0
0.1
β_{IV}

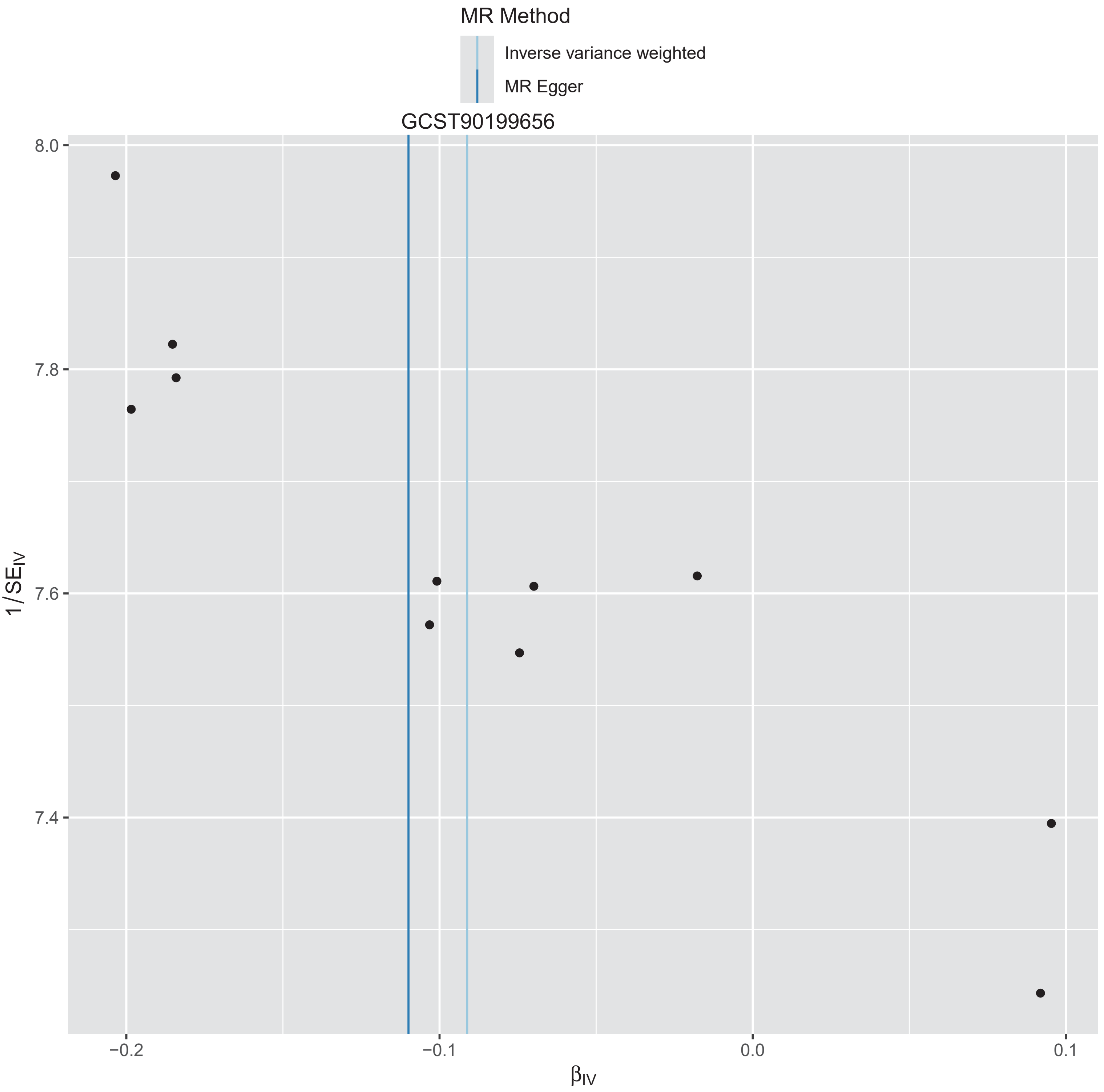
MR Method
Inverse variance weighted
MR Egger
GCST90199656
8.0
7.8
7.6
7.4
1/SE_IV
−0.2
−0.1
0.0
0.1
β_IV

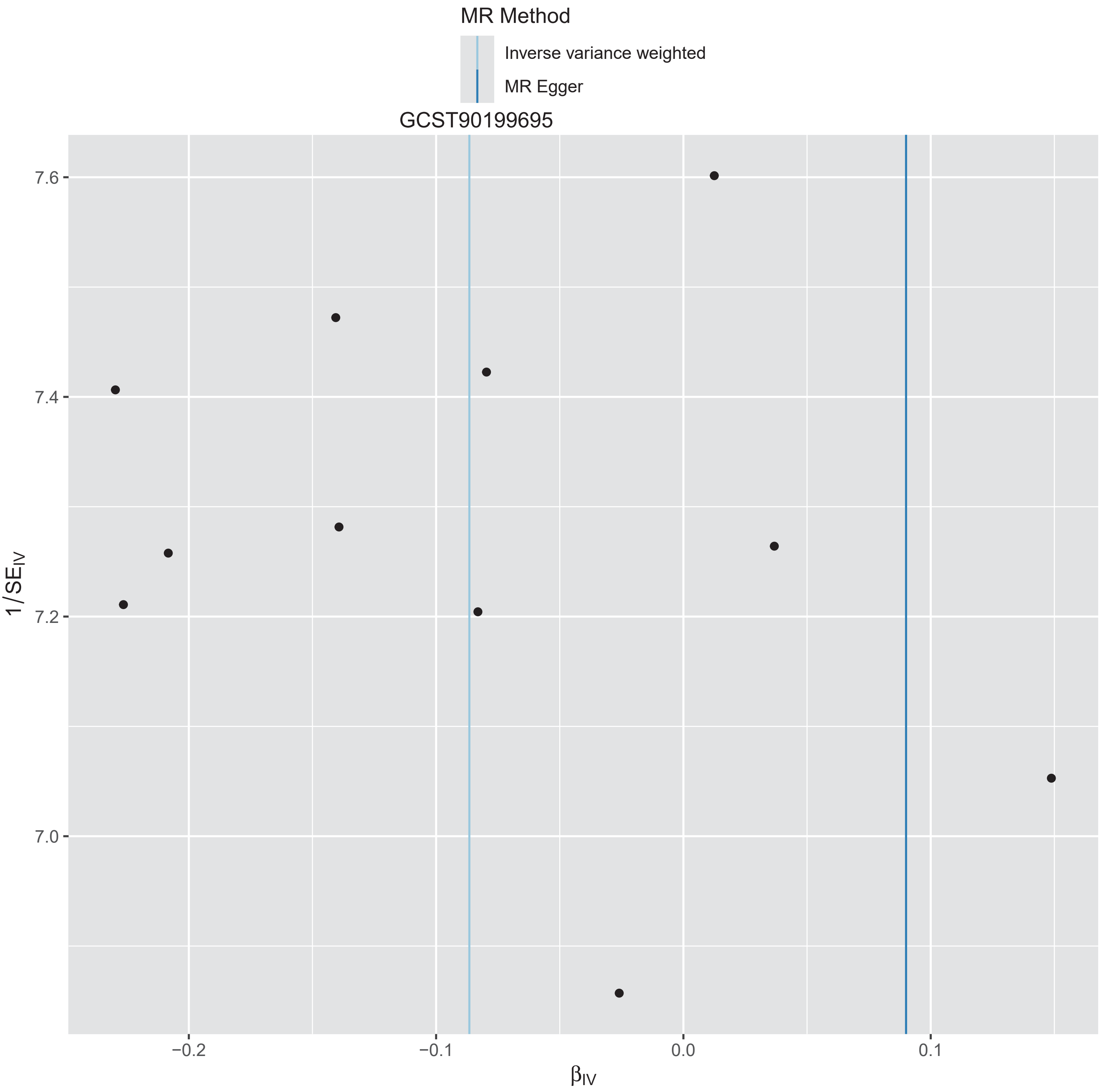
MR Method
Inverse variance weighted
MR Egger
GCST90199695
7.6
7.4
7.2
7.0
1/SE_IV
−0.2
−0.1
0.0
0.1
β_IV

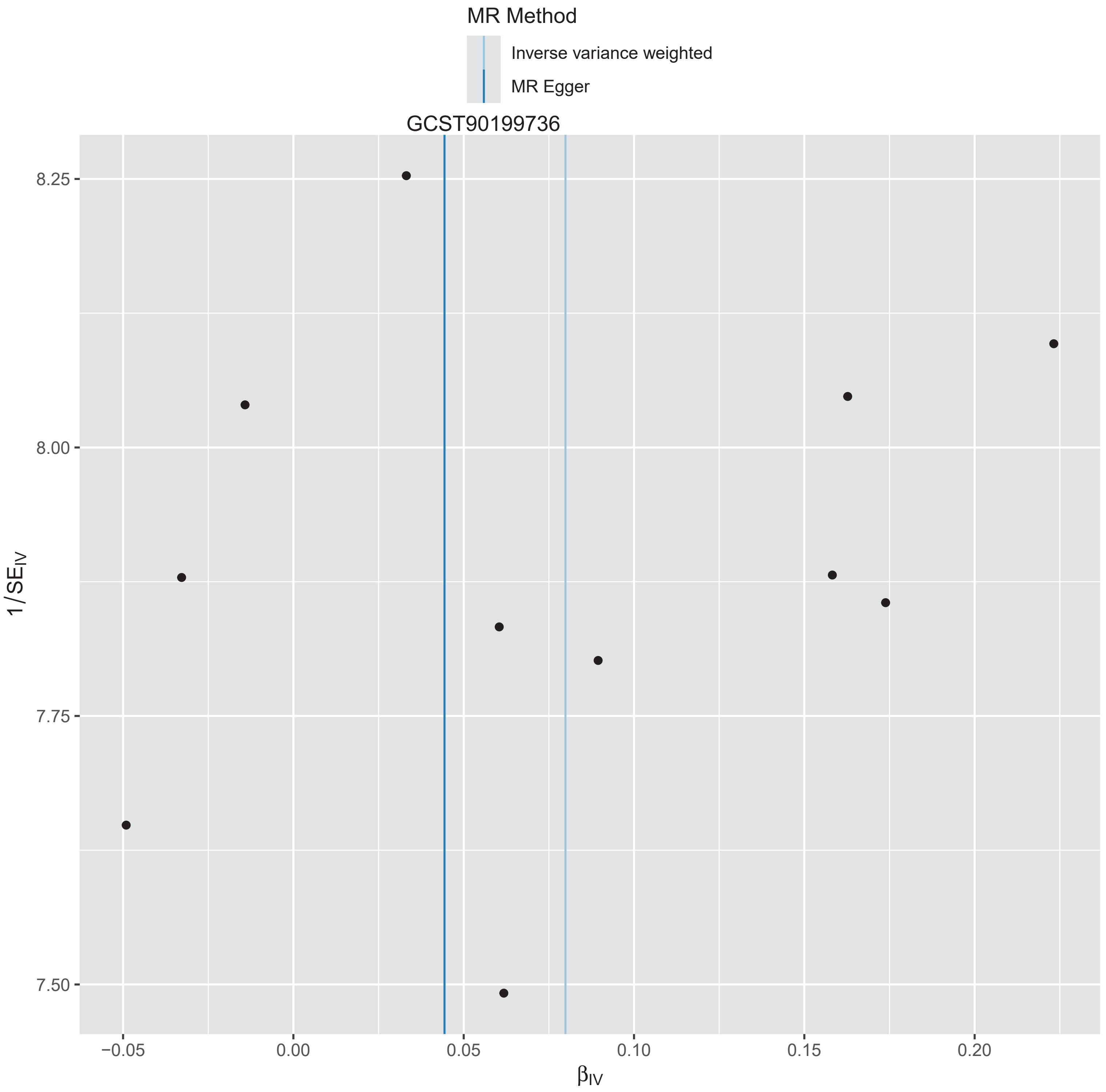
MR Method
Inverse variance weighted
MR Egger
GCST90199736
8.25
8.00
7.75
7.50
1/SE_IV
−0.05
0.00
0.05
0.10
0.15
0.20
β_IV

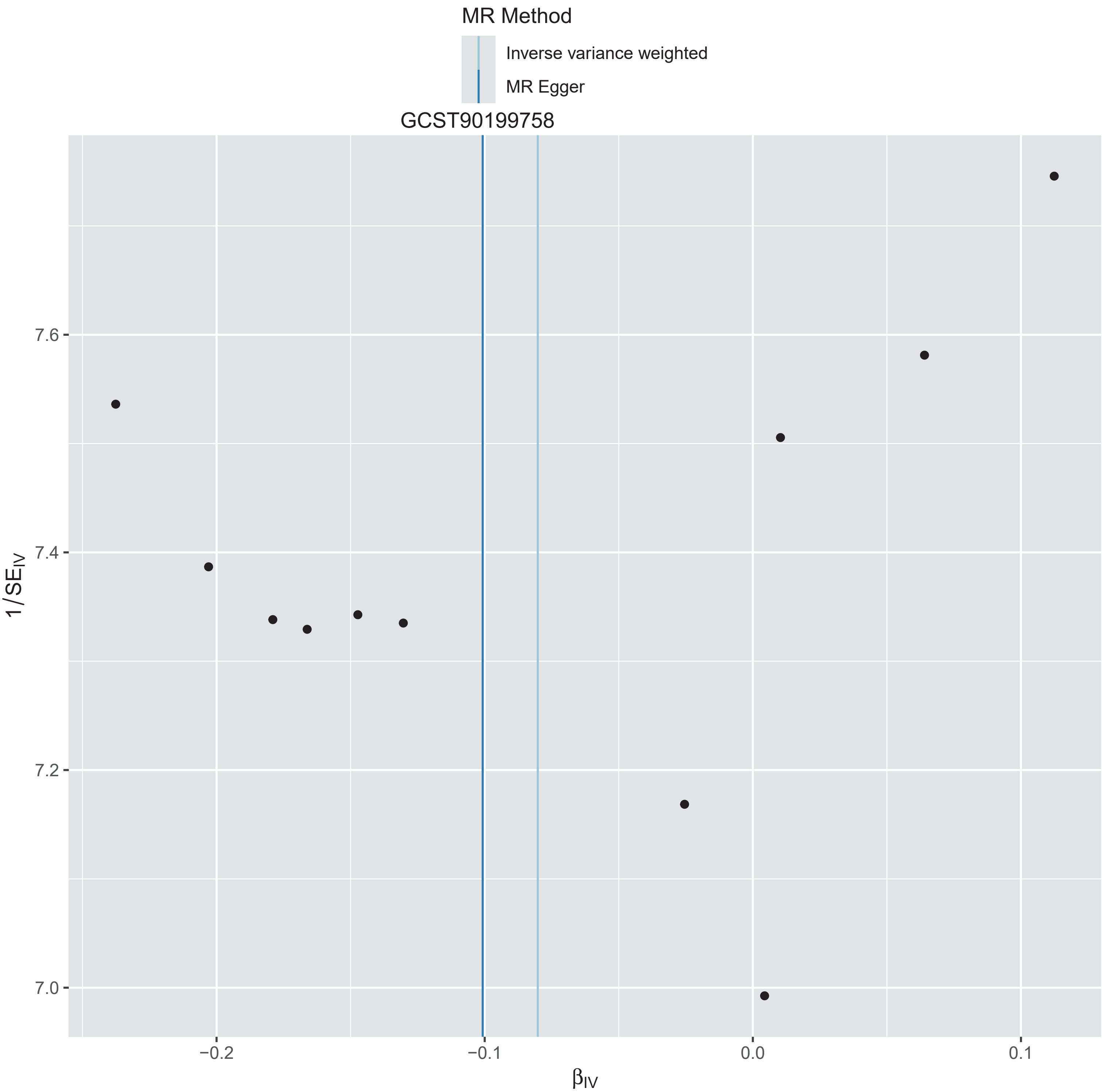

MR Method
Inverse variance weighted
MR Egger
GCST90199758
1/SE$_{IV}$
7.6
7.4
7.2
7.0
−0.2
−0.1
0.0
0.1
β$_{IV}$

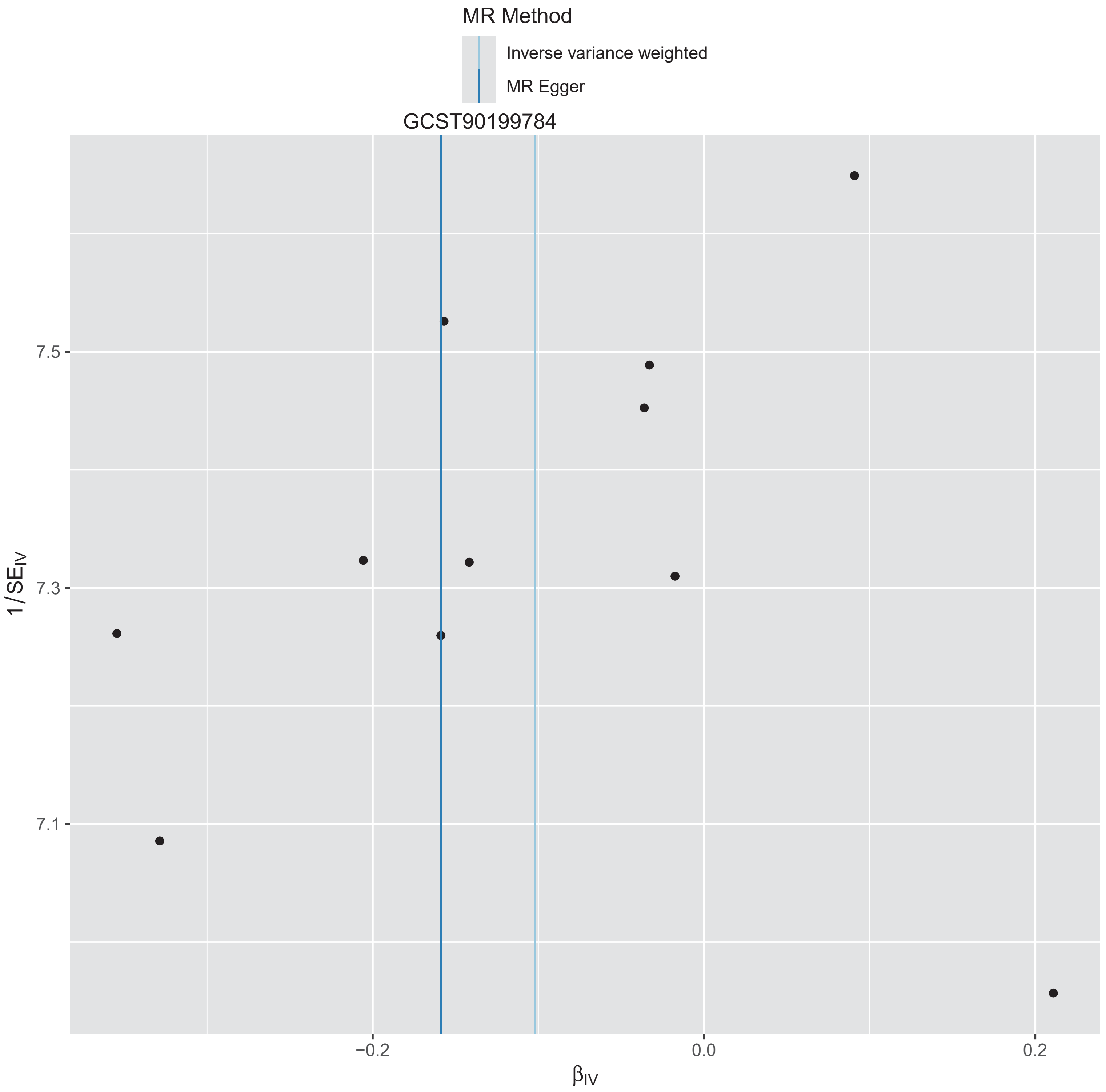

MR Method
Inverse variance weighted
MR Egger
GCST90199784
7.5
7.3
7.1
1/SE$_{IV}$
−0.2
0.0
0.2
β$_{IV}$

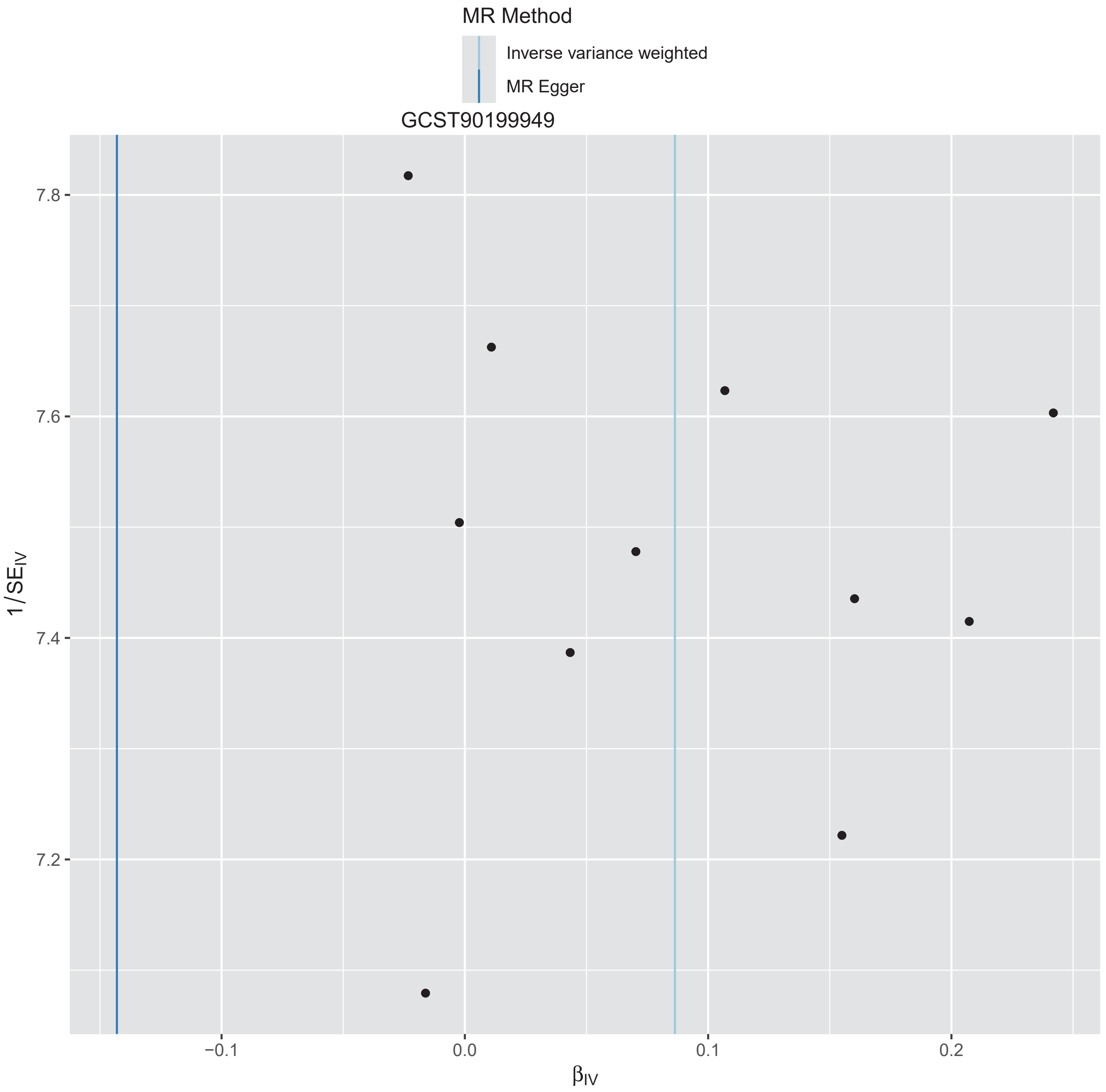
MR Method
Inverse variance weighted
MR Egger
GCST90199949
7.8
7.6
7.4
7.2
1/SE$_{IV}$
−0.1
0.0
0.1
0.2
β_{IV}

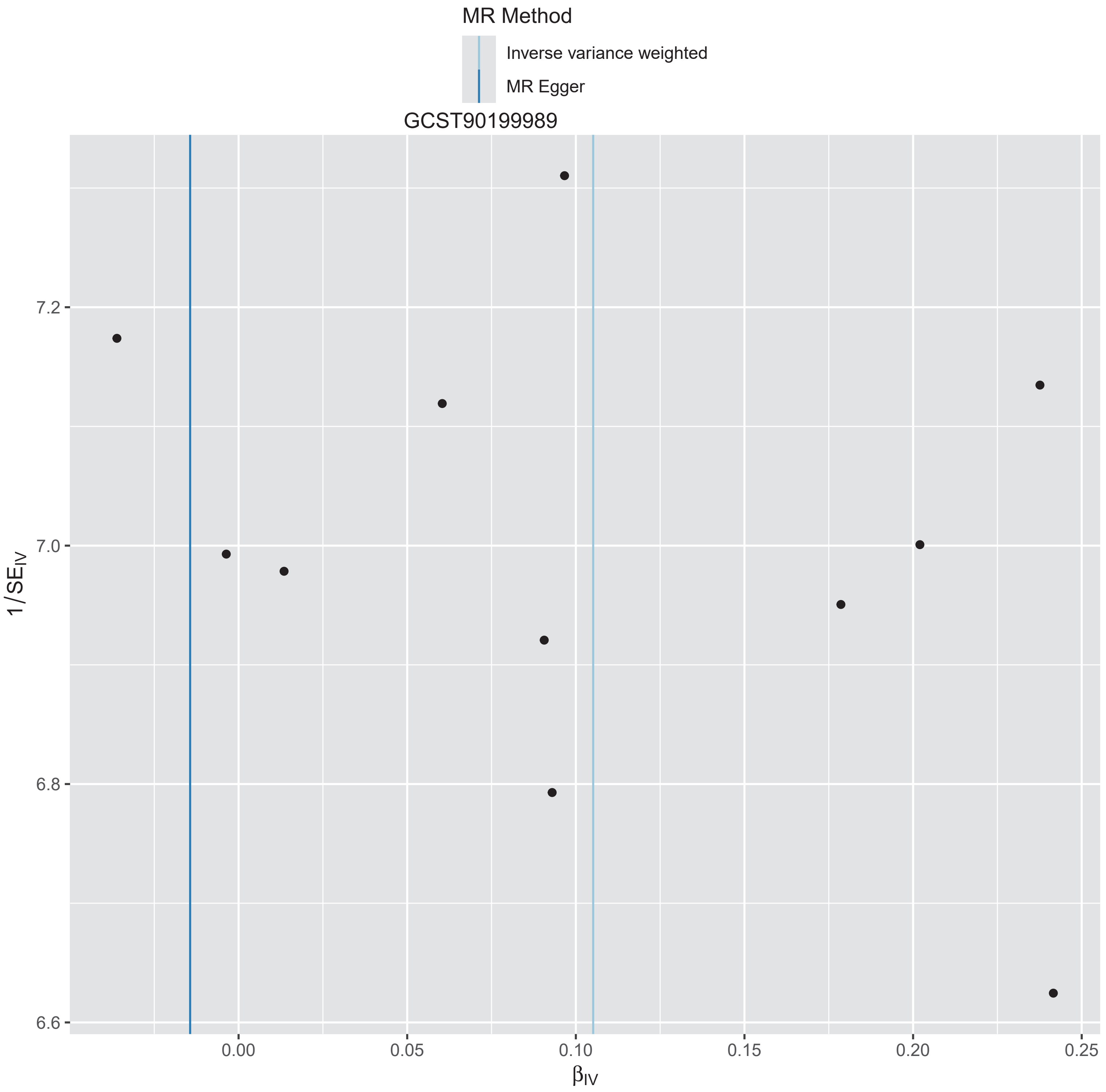

MR Method
Inverse variance weighted
MR Egger
GCST90199989
7.2
7.0
6.8
6.6
1/SE_IV
0.00
0.05
0.10
0.15
0.20
0.25
β_IV

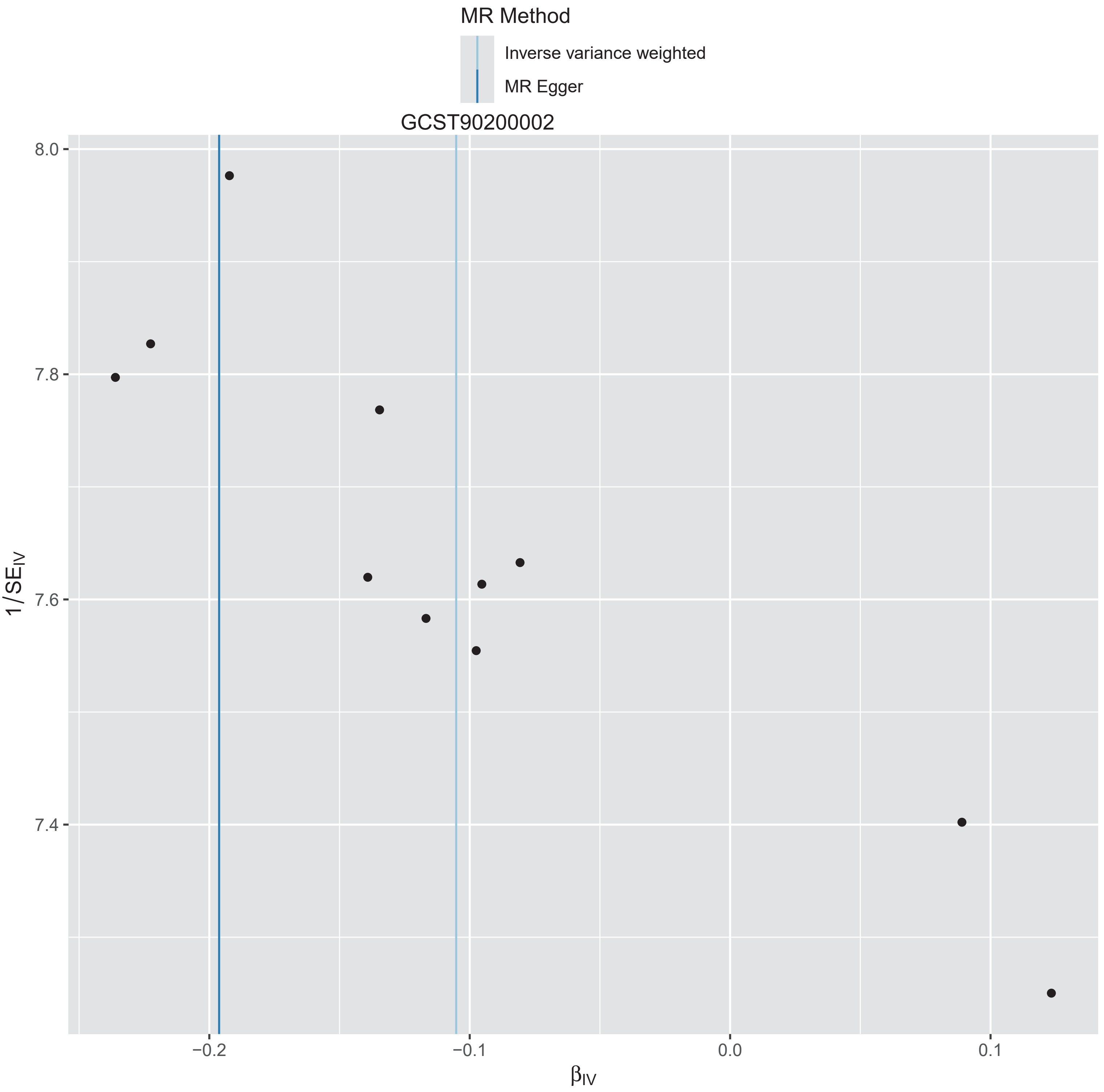

MR Method
Inverse variance weighted
MR Egger
GCST90200002
8.0
7.8
7.6
7.4
1/SE_IV
−0.2
−0.1
0.0
0.1
β_IV

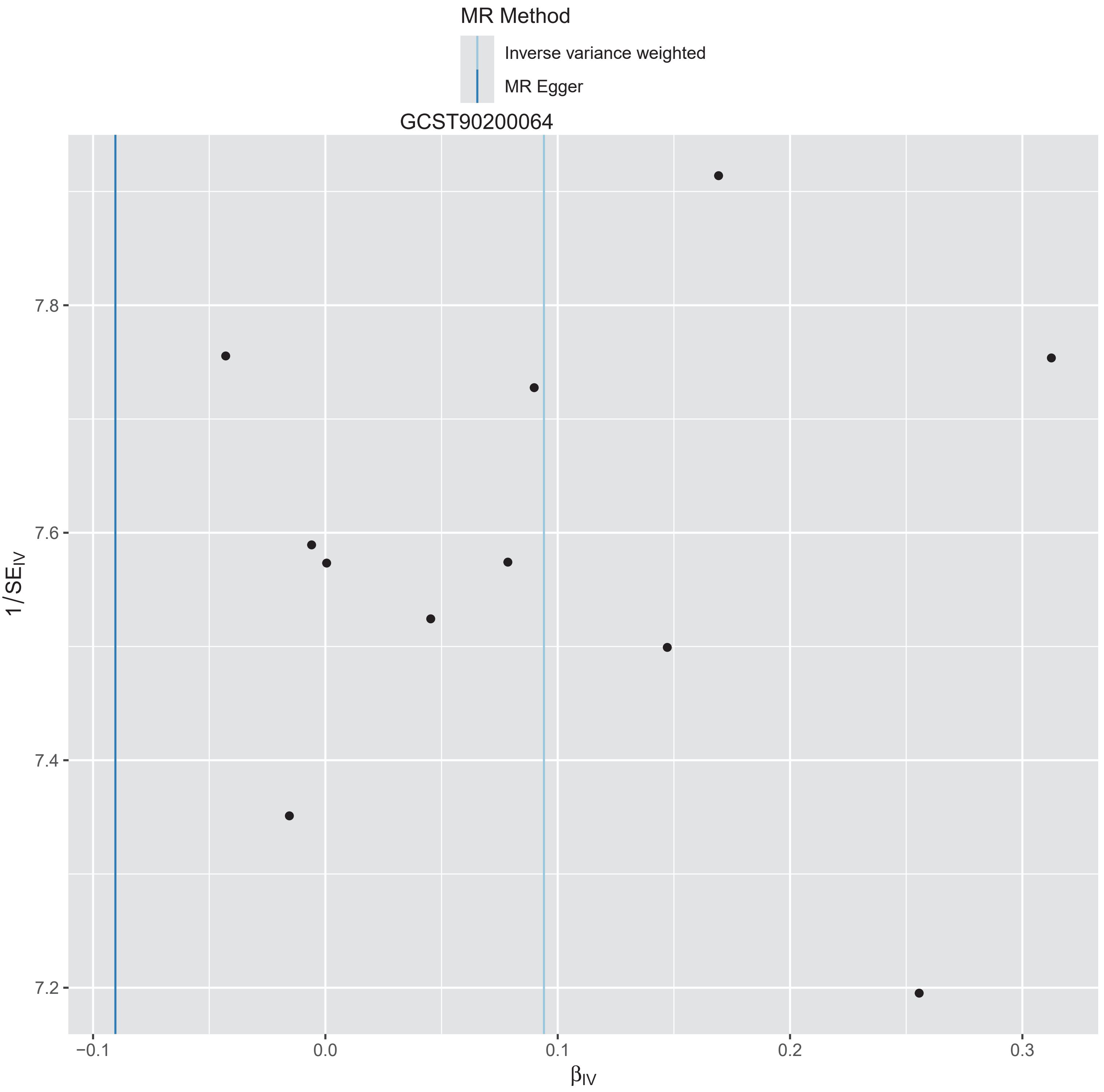
MR Method
Inverse variance weighted
MR Egger
GCST90200064
7.8
7.6
7.4
7.2
1/SE_IV
−0.1
0.0
0.1
0.2
0.3
β_IV

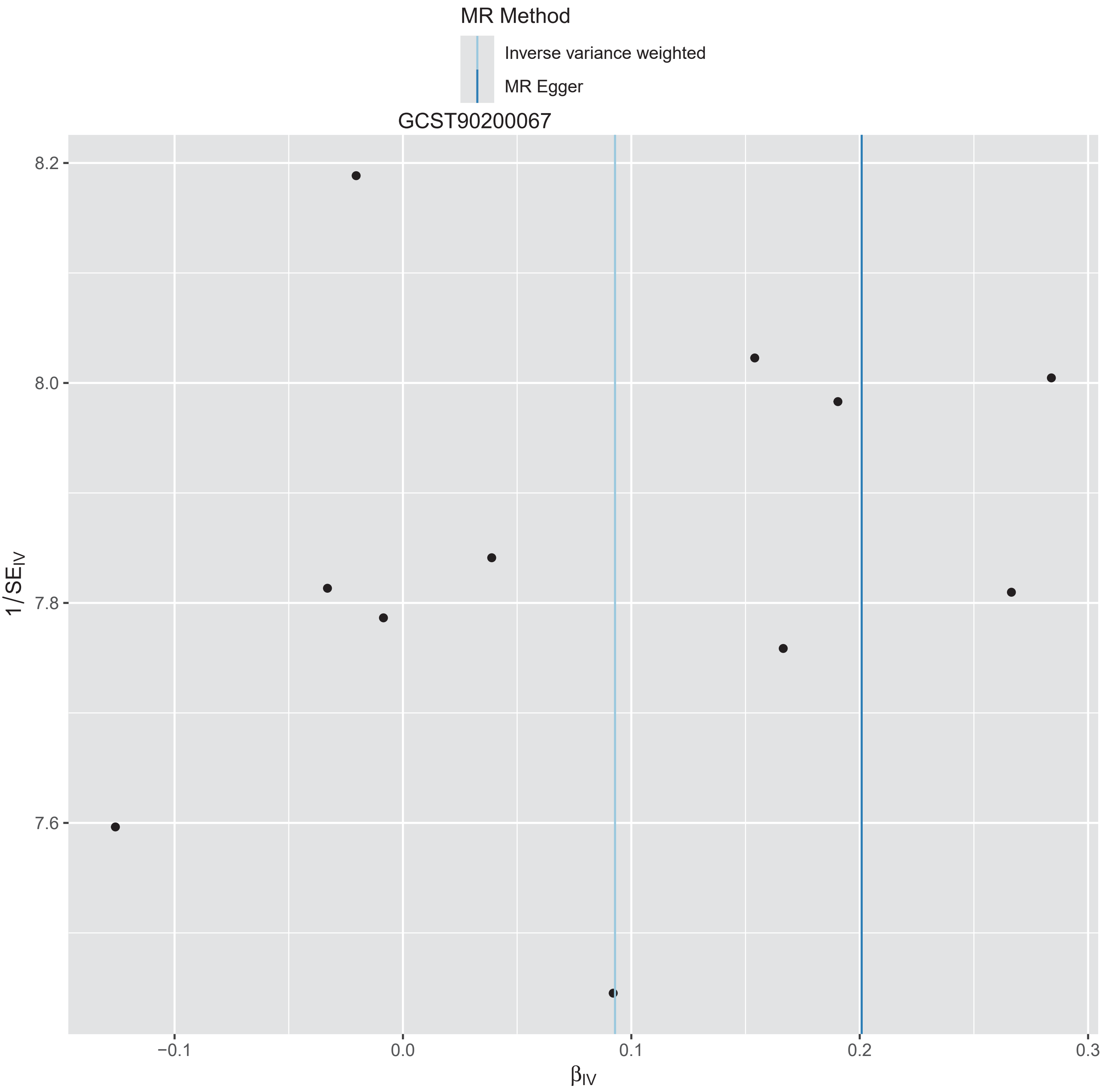
MR Method
Inverse variance weighted
MR Egger
GCST90200067
8.2
8.0
7.8
7.6
1/SE_IV
−0.1
0.0
0.1
0.2
0.3
β_IV

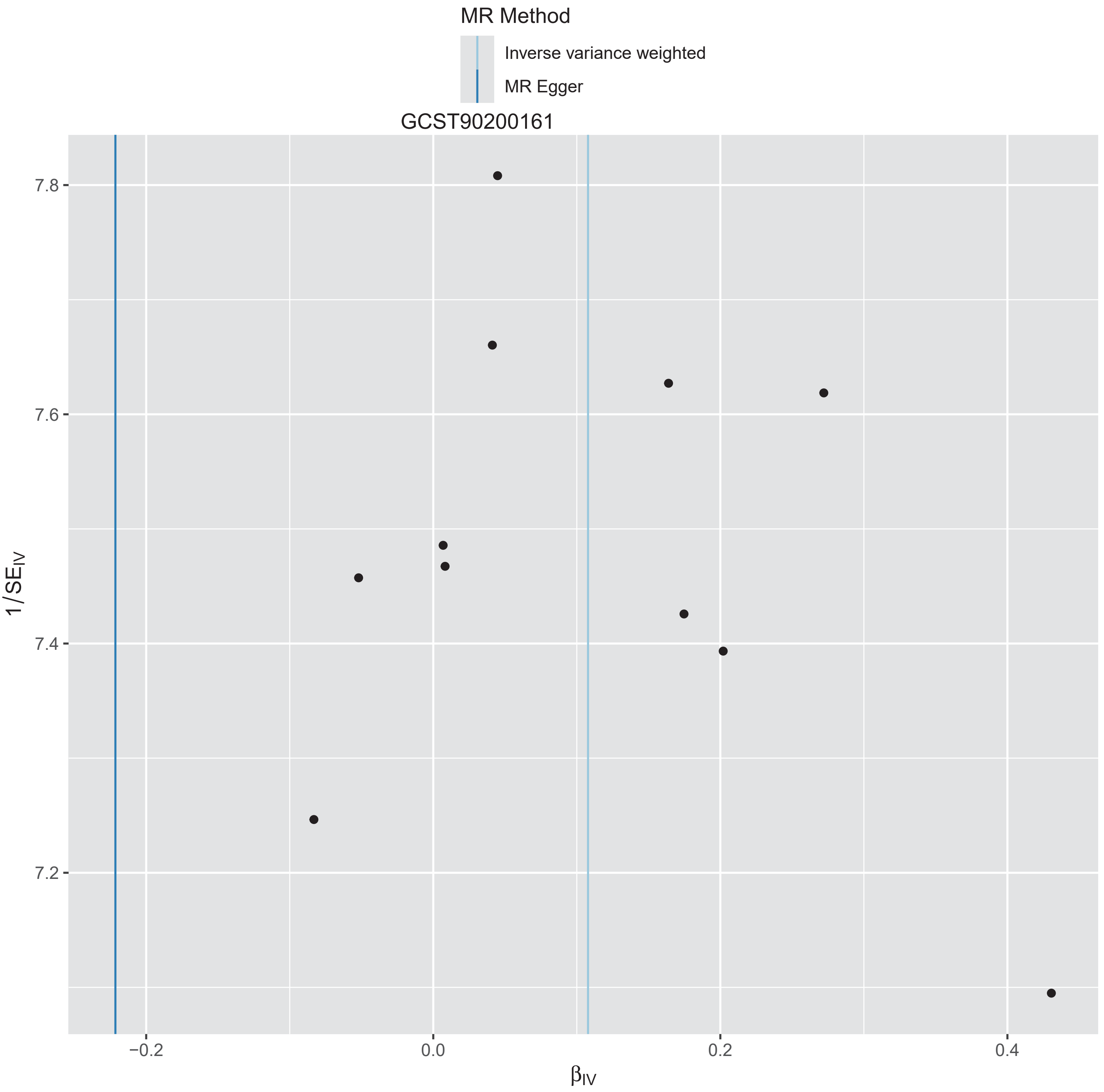

MR Method
Inverse variance weighted
MR Egger
GCST90200161
7.8
7.6
7.4
7.2
1/SE$_{IV}$
−0.2
0.0
0.2
0.4
β$_{IV}$

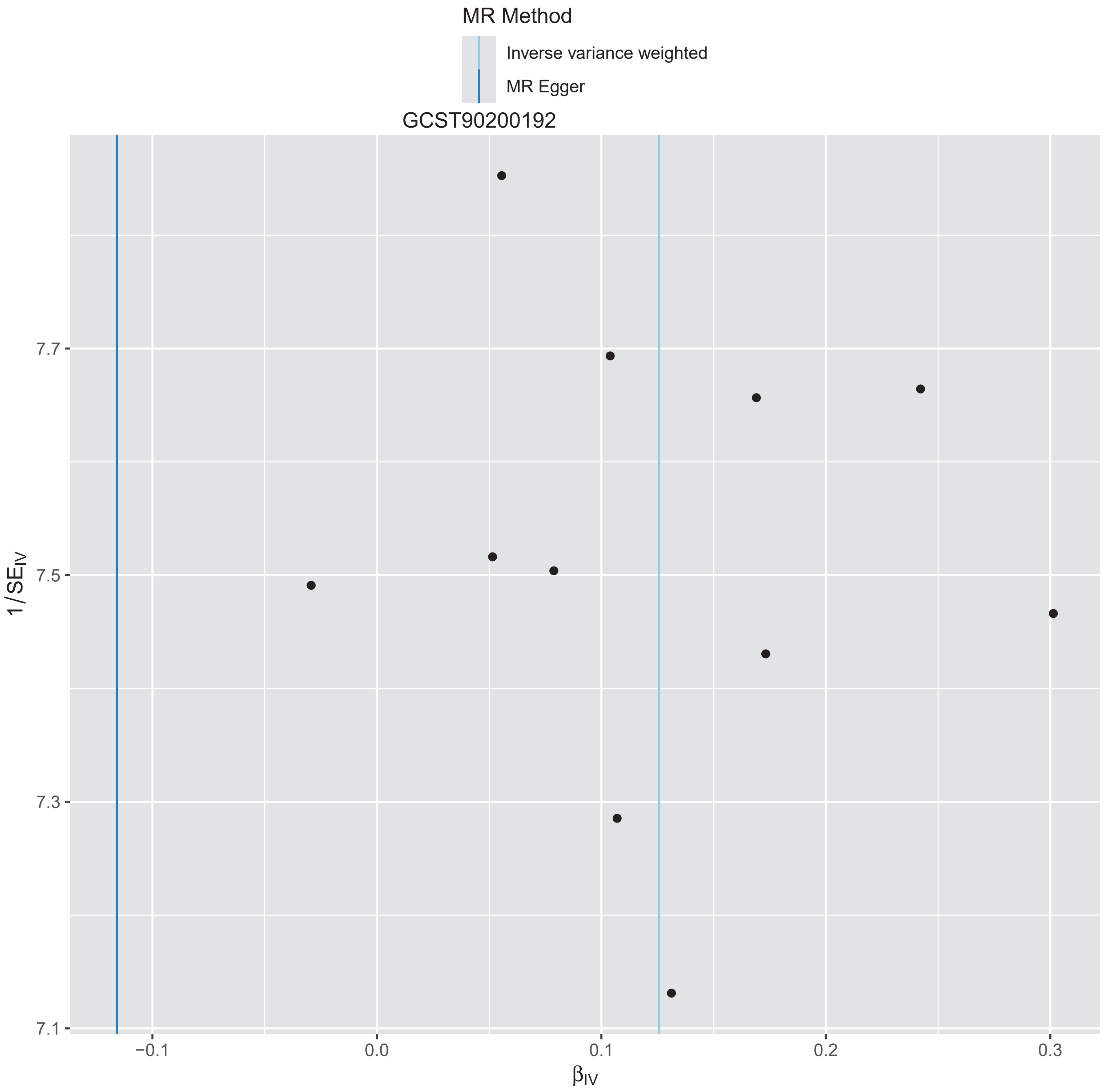

MR Method
Inverse variance weighted
MR Egger
GCST90200192
1/SE_IV
7.7
7.5
7.3
7.1
−0.1
0.0
0.1
0.2
0.3
β_IV

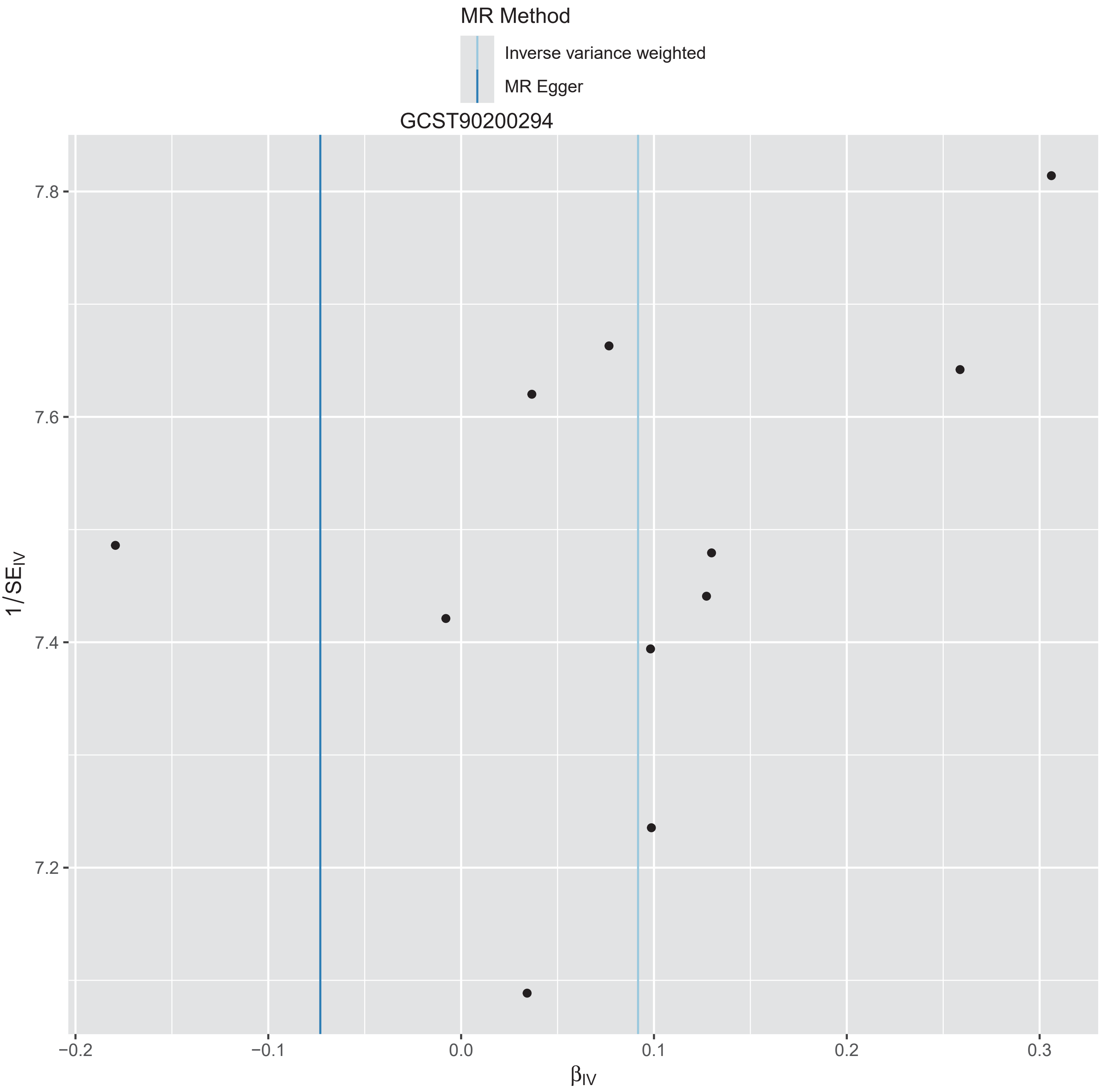

MR Method
Inverse variance weighted
MR Egger
GCST90200294
7.8
7.6
7.4
7.2
1/SE_IV
−0.2
−0.1
0.0
0.1
0.2
0.3
β_IV

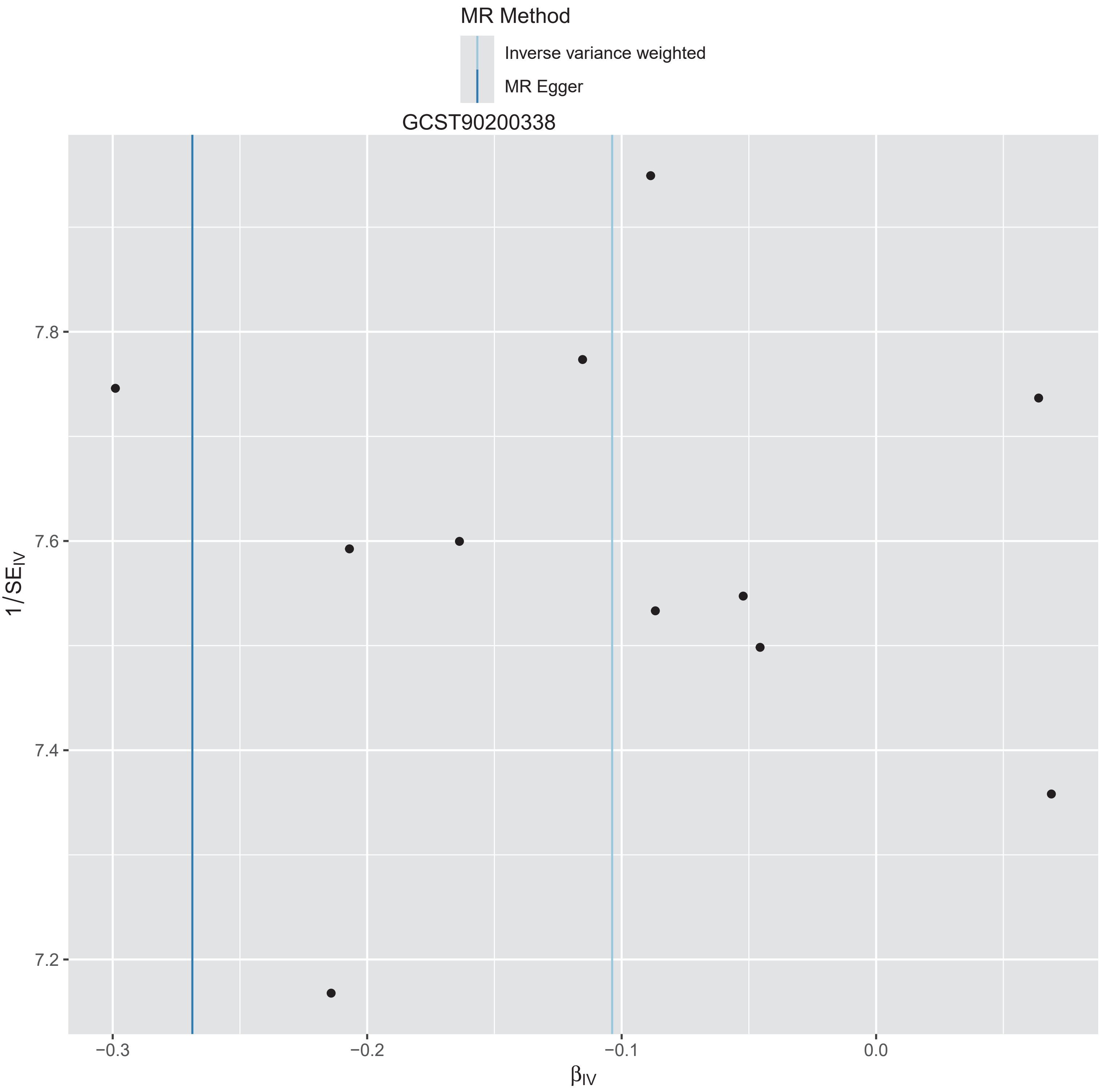
MR Method
Inverse variance weighted
MR Egger
GCST90200338
7.8
7.6
7.4
7.2
1/SE$_{IV}$
−0.3
−0.2
−0.1
0.0
β_{IV}

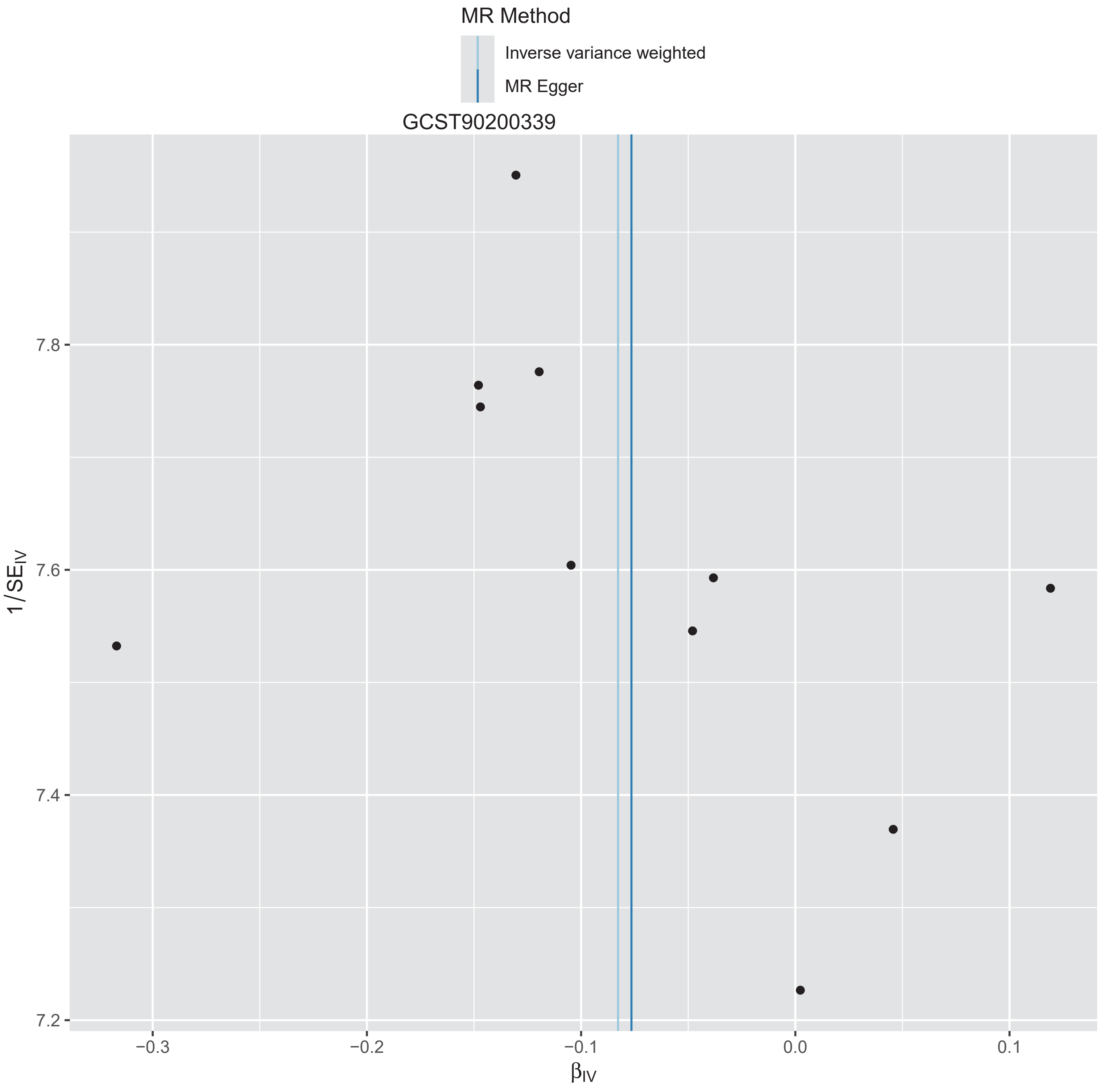

MR Method
Inverse variance weighted
MR Egger
GCST90200339
7.8
7.6
7.4
7.2
1/SE_IV
−0.3
−0.2
−0.1
0.0
0.1
β_IV

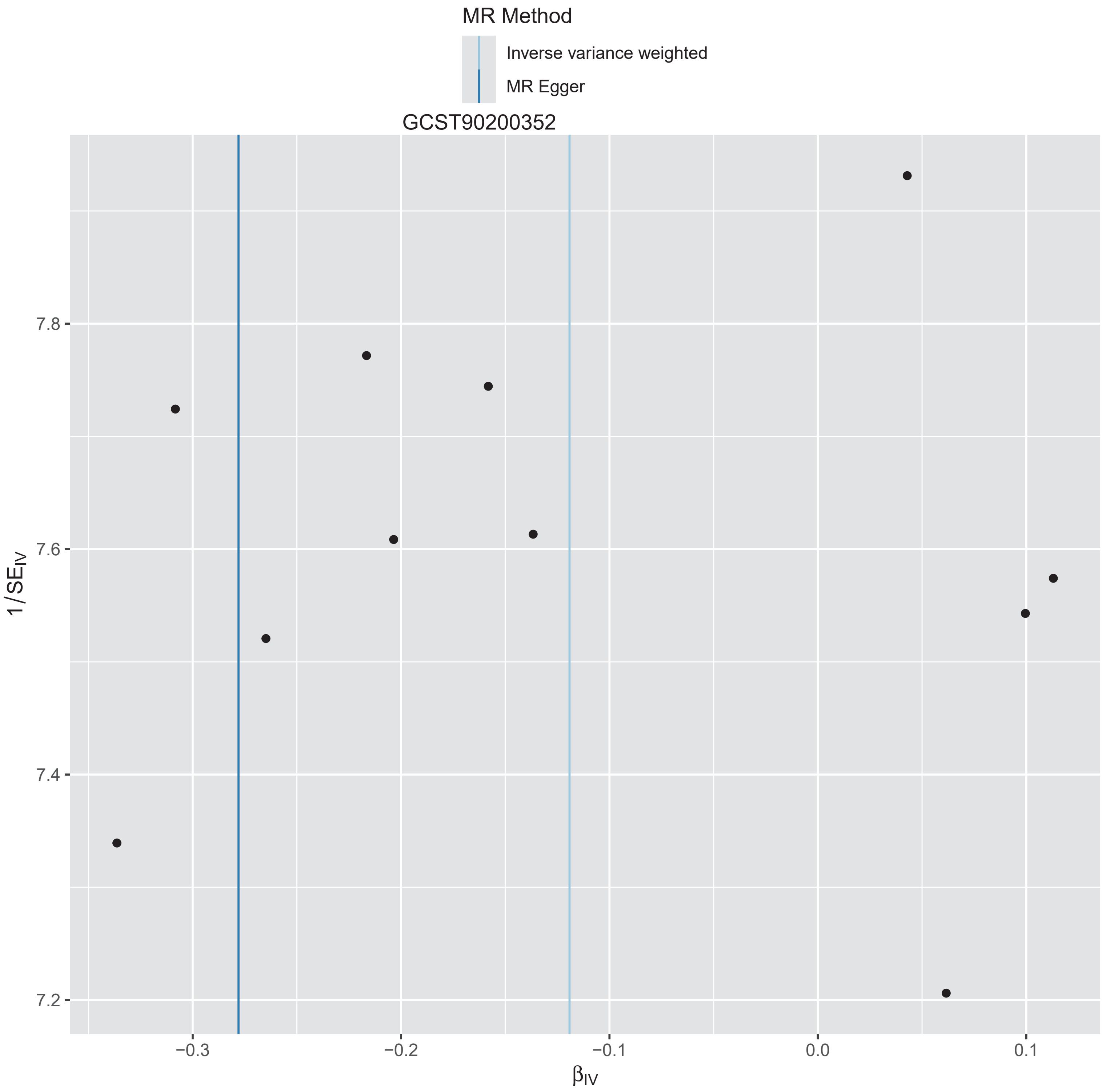
MR Method
Inverse variance weighted
MR Egger
GCST90200352
7.8
7.6
7.4
7.2
1/SE_IV
−0.3
−0.2
−0.1
0.0
0.1
β_IV

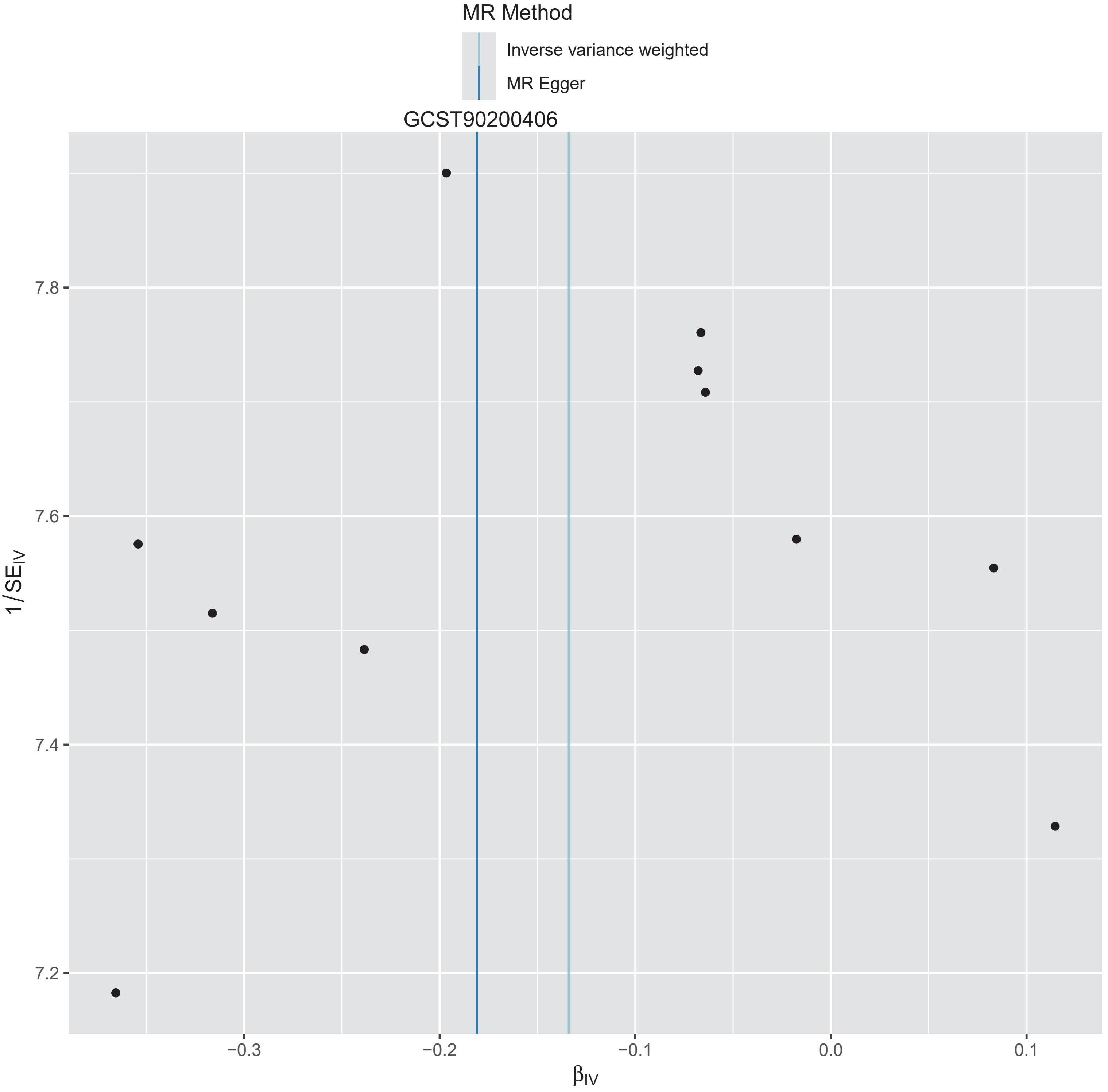
MR Method
Inverse variance weighted
MR Egger
GCST90200406
7.8
7.6
7.4
7.2
1/SE_IV
−0.3
−0.2
−0.1
0.0
0.1
β_IV

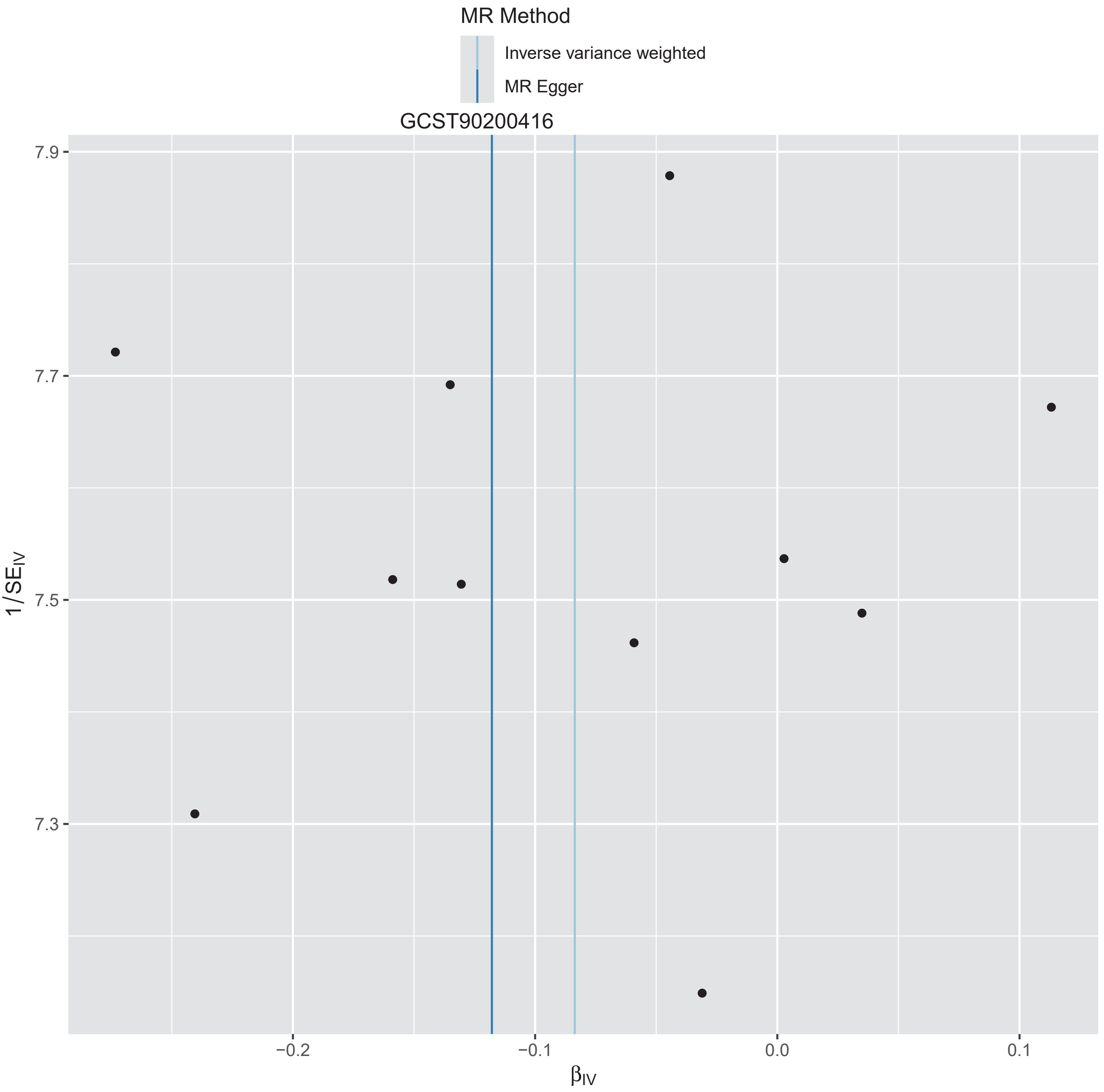

MR Method
Inverse variance weighted
MR Egger
GCST90200416
7.9
7.7
7.5
7.3
1/SE_IV
−0.2
−0.1
0.0
0.1
β_IV

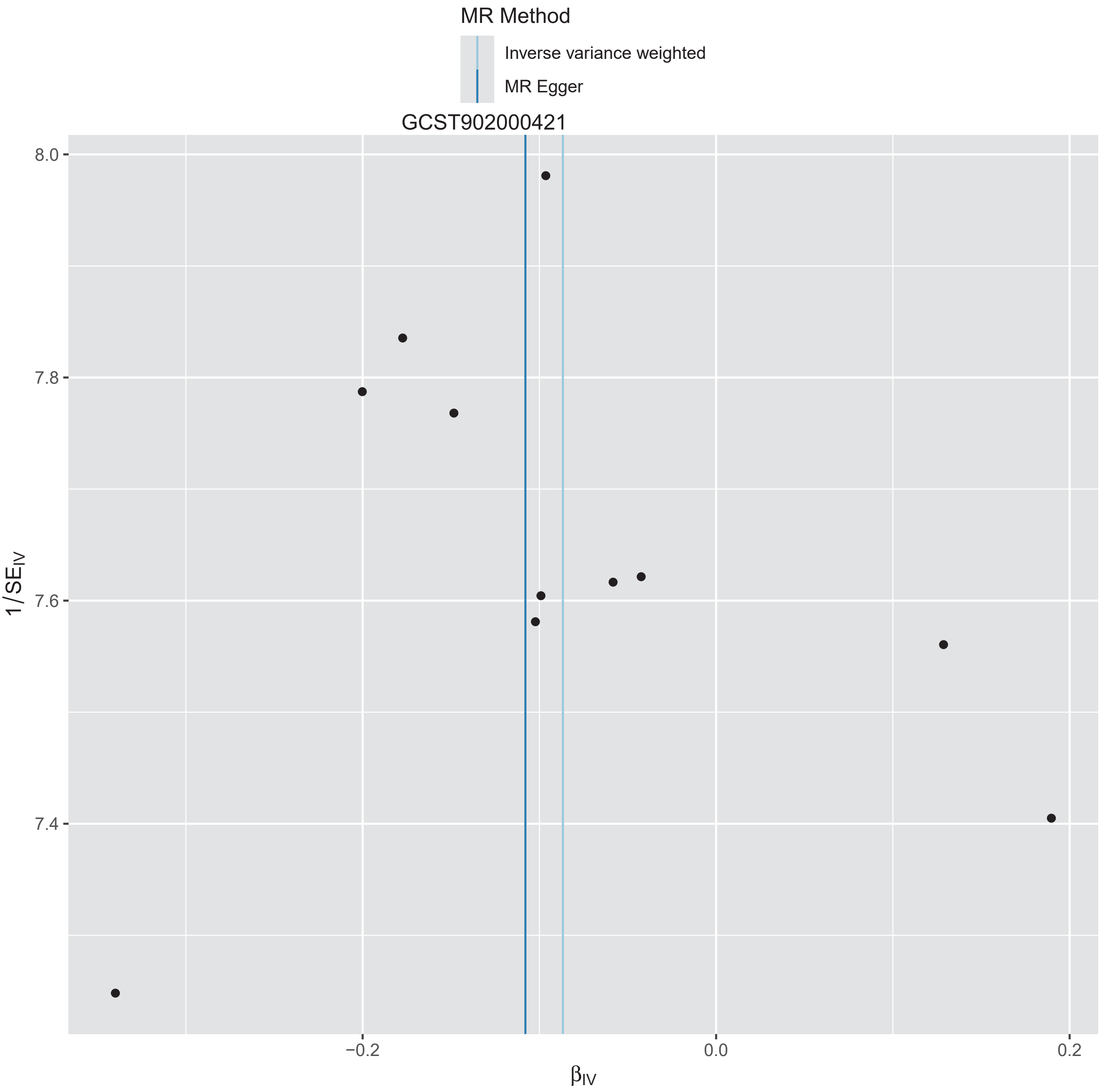

MR Method
Inverse variance weighted
MR Egger
GCST902000421
8.0
7.8
7.6
7.4
1/SE_IV
−0.2
0.0
0.2
β_IV

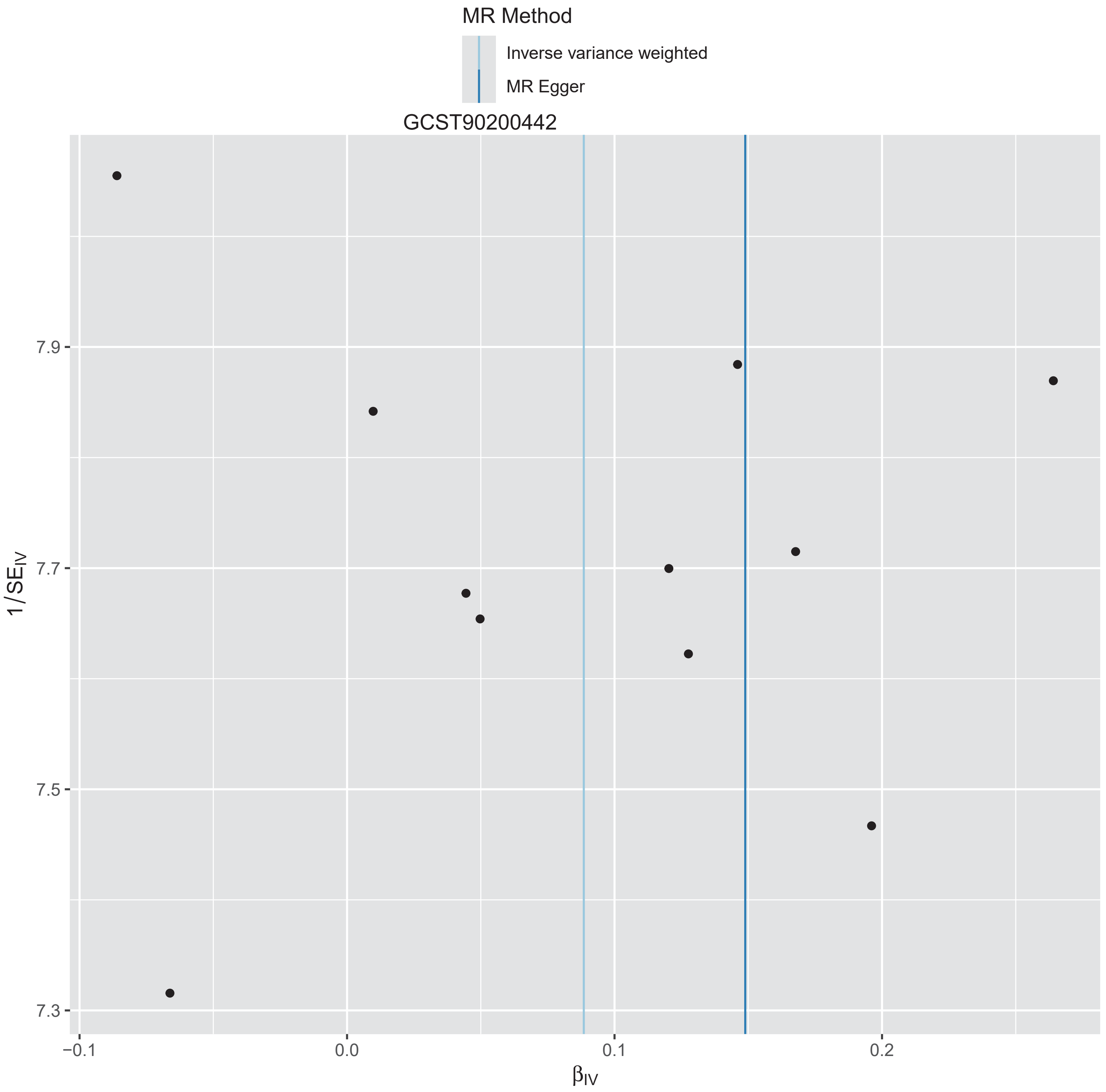

MR Method
Inverse variance weighted
MR Egger
GCST90200442
7.9
7.7
7.5
7.3
1/SE_IV
−0.1
0.0
0.1
0.2
β_IV

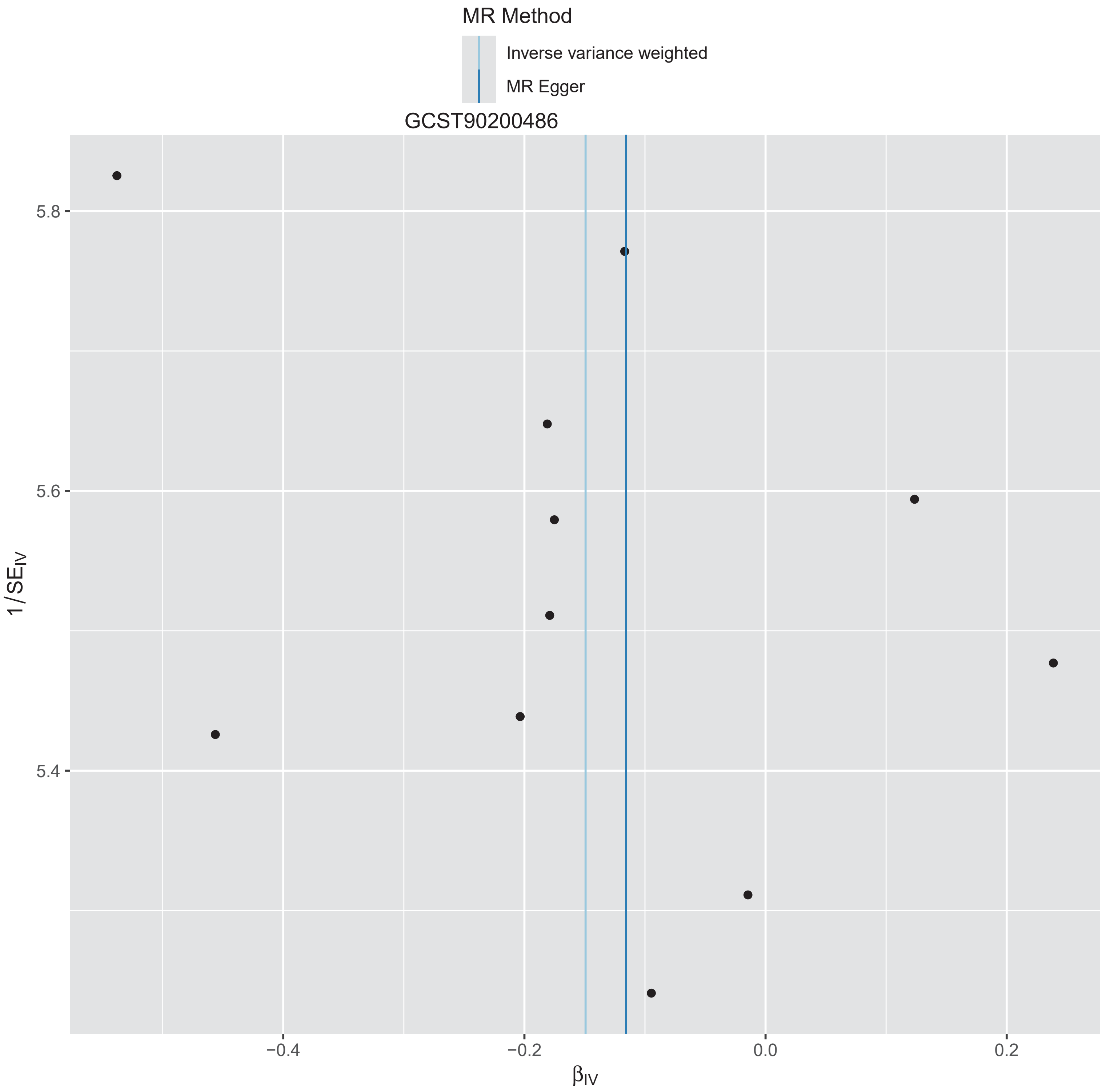

MR Method
Inverse variance weighted
MR Egger
GCST90200486
5.8
5.6
5.4
1/SE_IV
−0.4
−0.2
0.0
0.2
β_IV

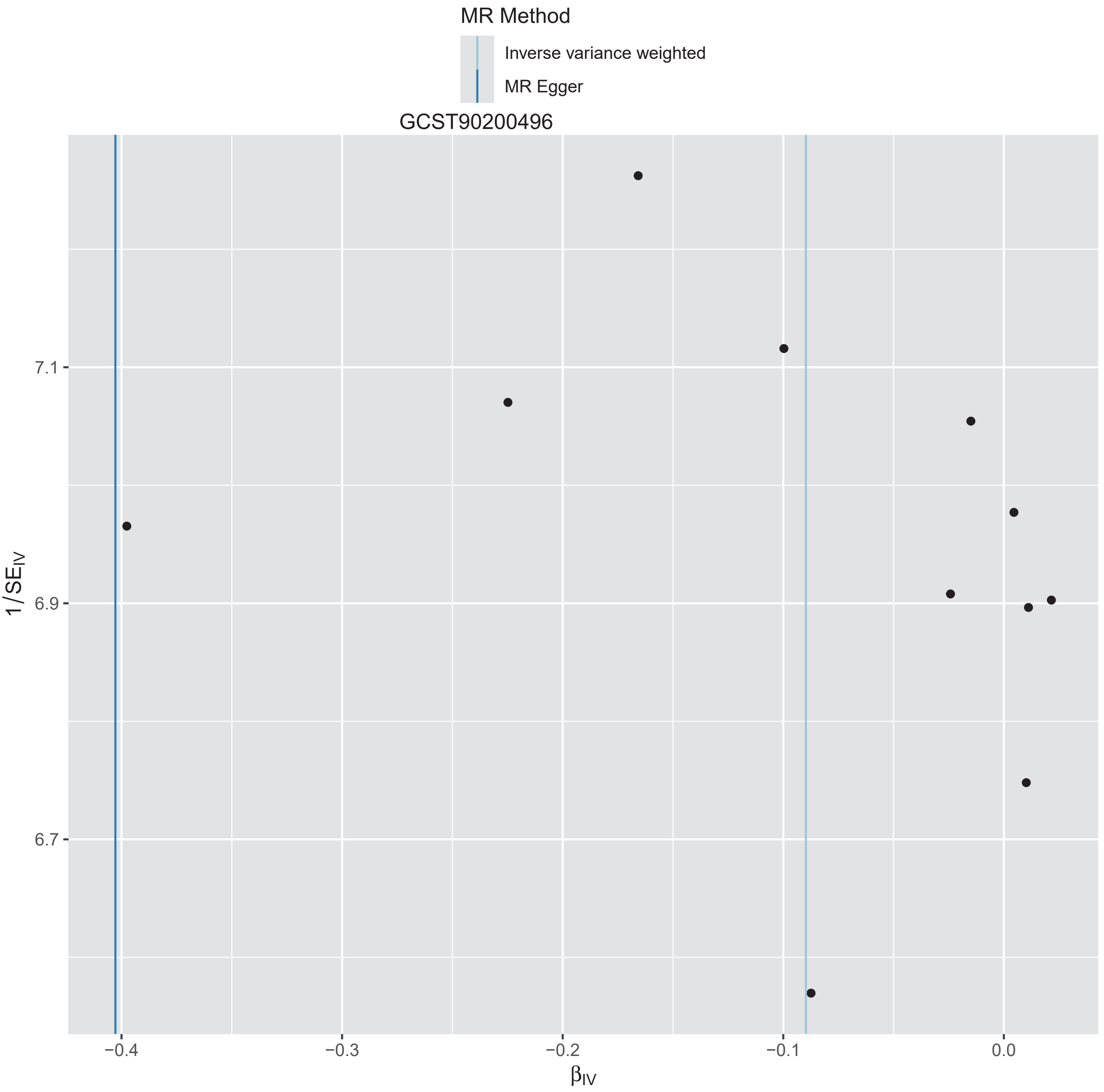
MR Method
Inverse variance weighted
MR Egger
GCST90200496
1/SE_IV
7.1
6.9
6.7
−0.4
−0.3
−0.2
−0.1
0.0
β_IV

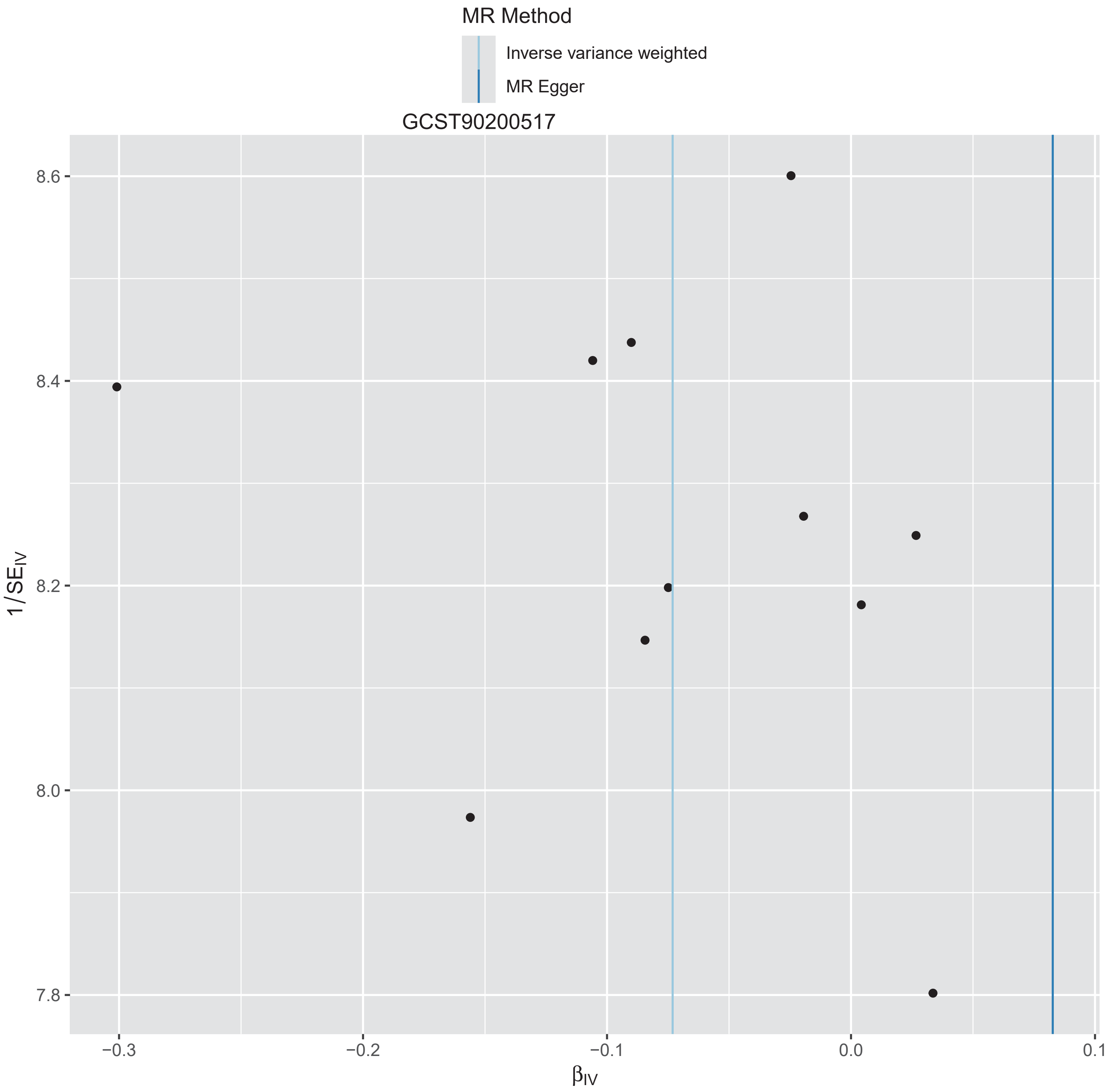
MR Method
Inverse variance weighted
MR Egger
GCST90200517
8.6
8.4
8.2
8.0
7.8
1/SE_IV
−0.3
−0.2
−0.1
0.0
0.1
β_IV

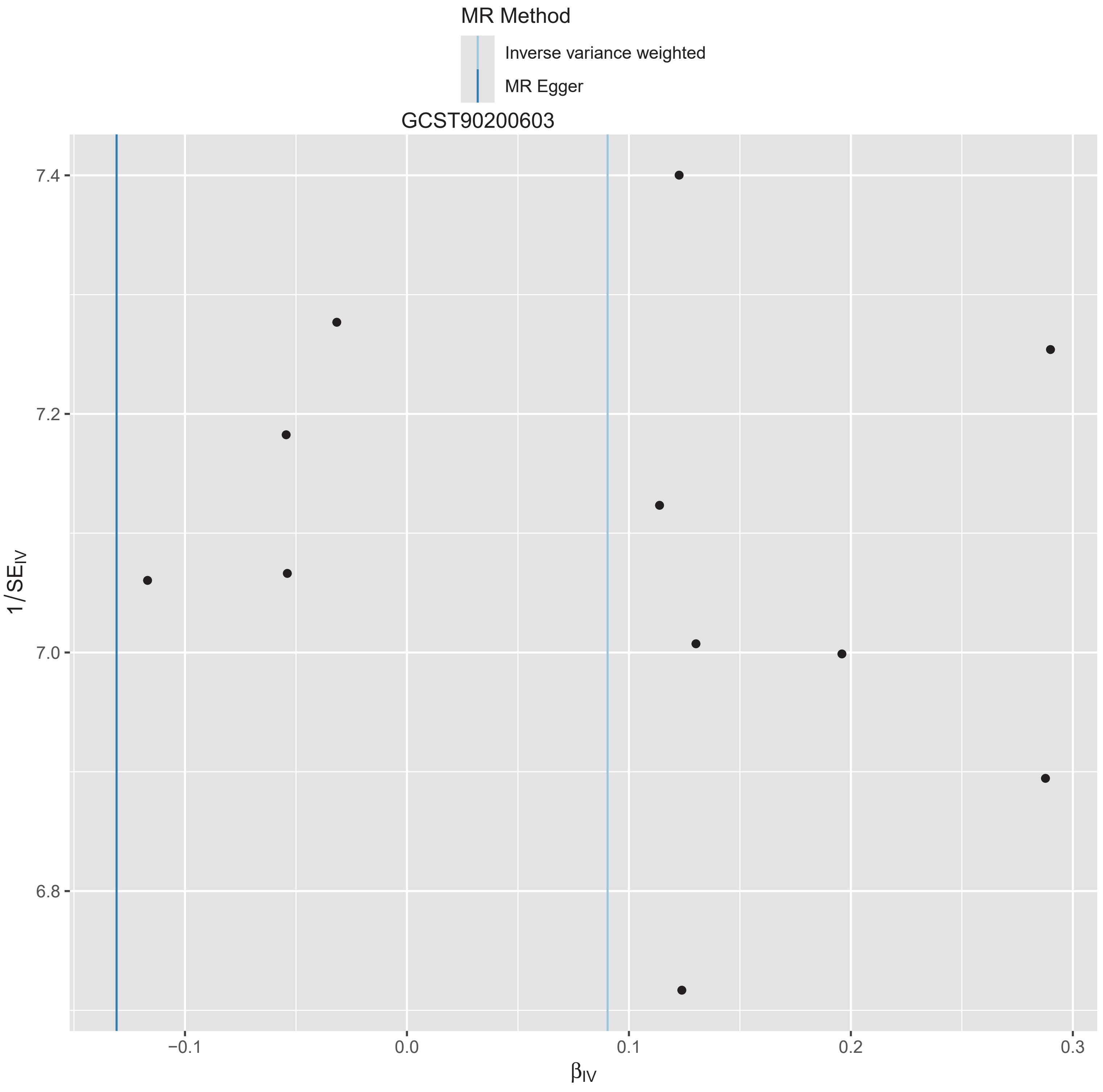
MR Method
Inverse variance weighted
MR Egger
GCST90200603
7.4
7.2
7.0
6.8
1/SE_IV
−0.1
0.0
0.1
0.2
0.3
β_IV

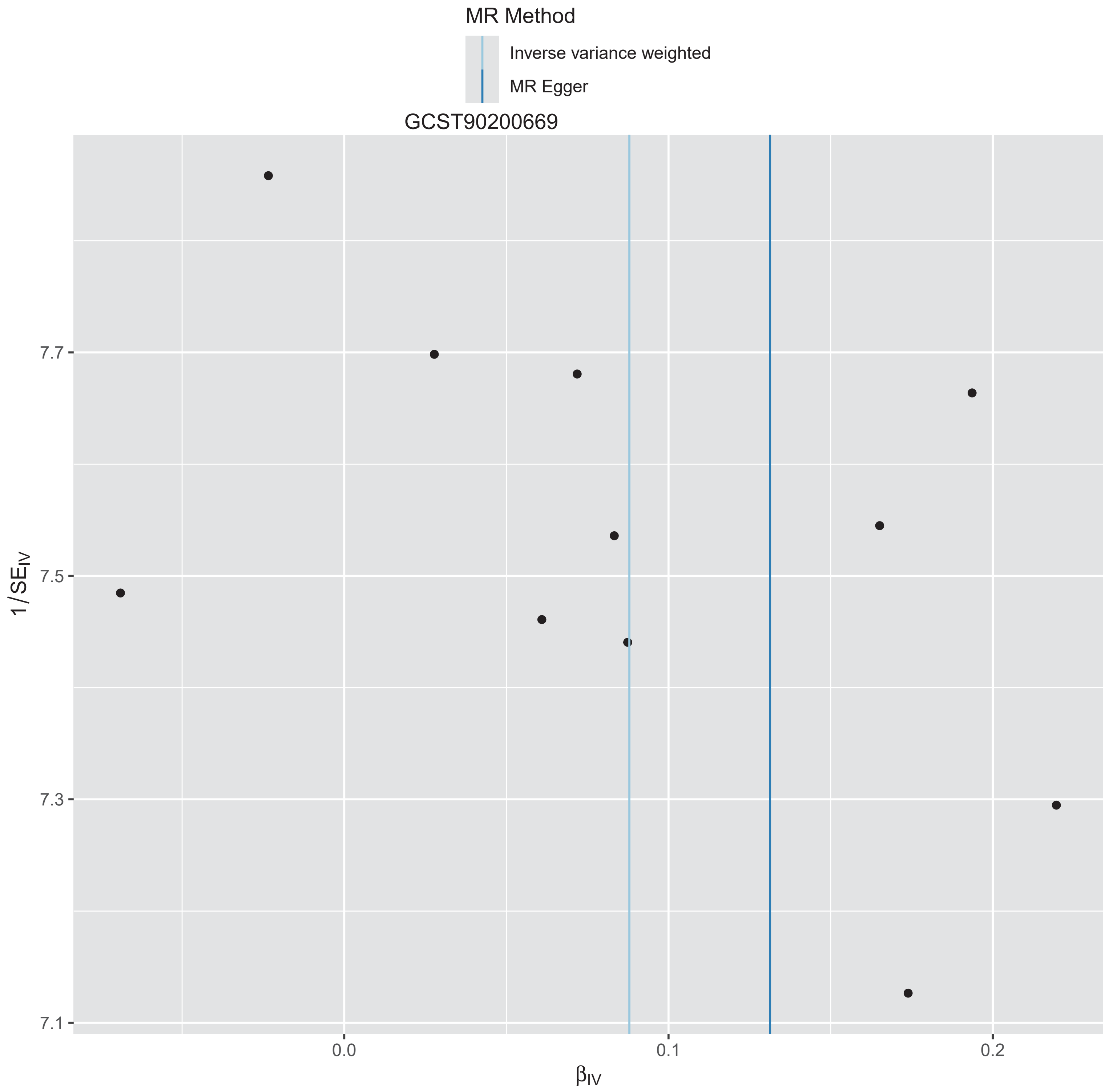
MR Method
Inverse variance weighted
MR Egger
GCST90200669
1/SE$_{IV}$
7.7
7.5
7.3
7.1
0.0
0.1
0.2
β$_{IV}$

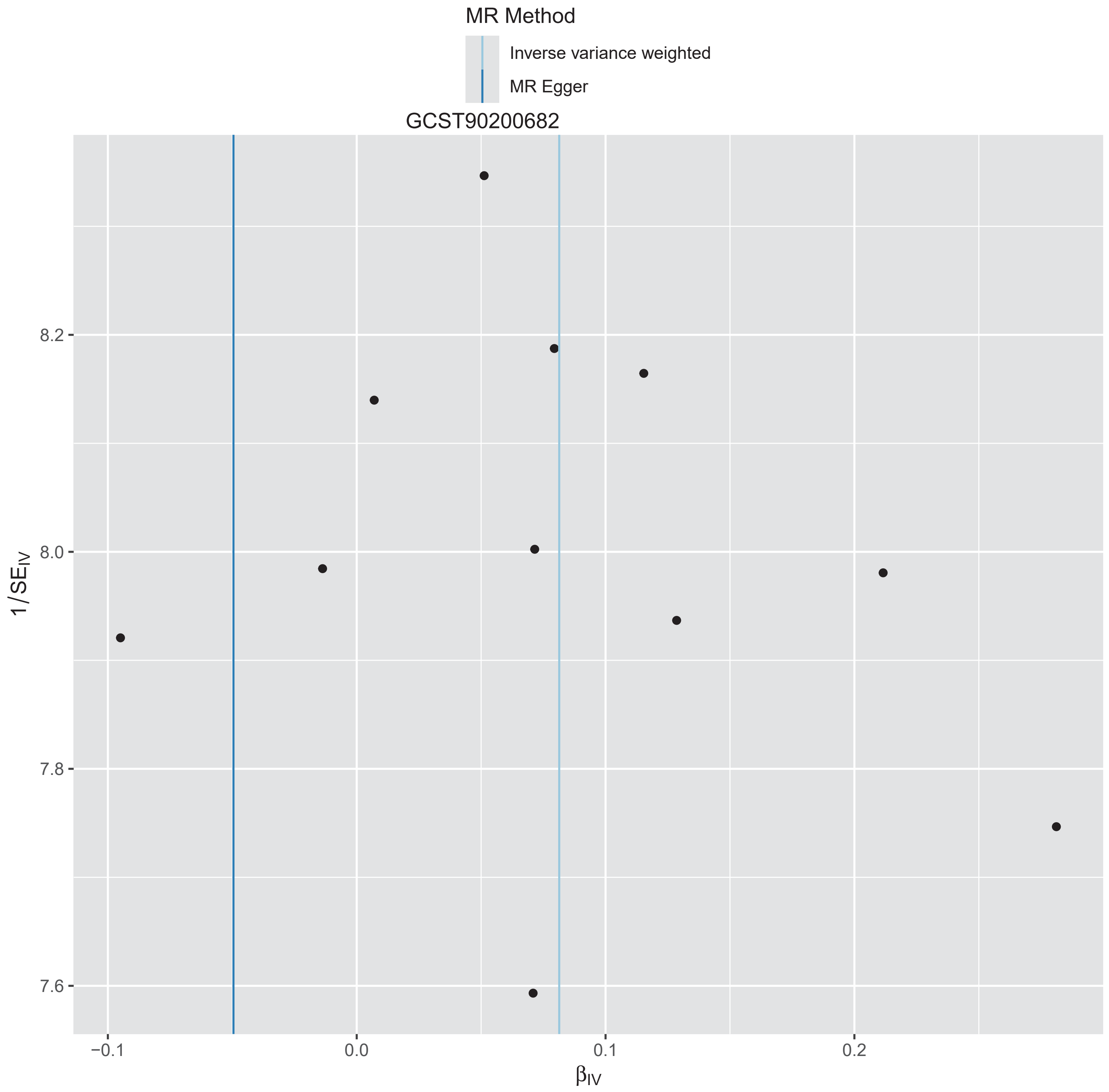

MR Method
Inverse variance weighted
MR Egger
GCST90200682
8.2
8.0
7.8
7.6
1/SE_IV
−0.1
0.0
0.1
0.2
β_IV

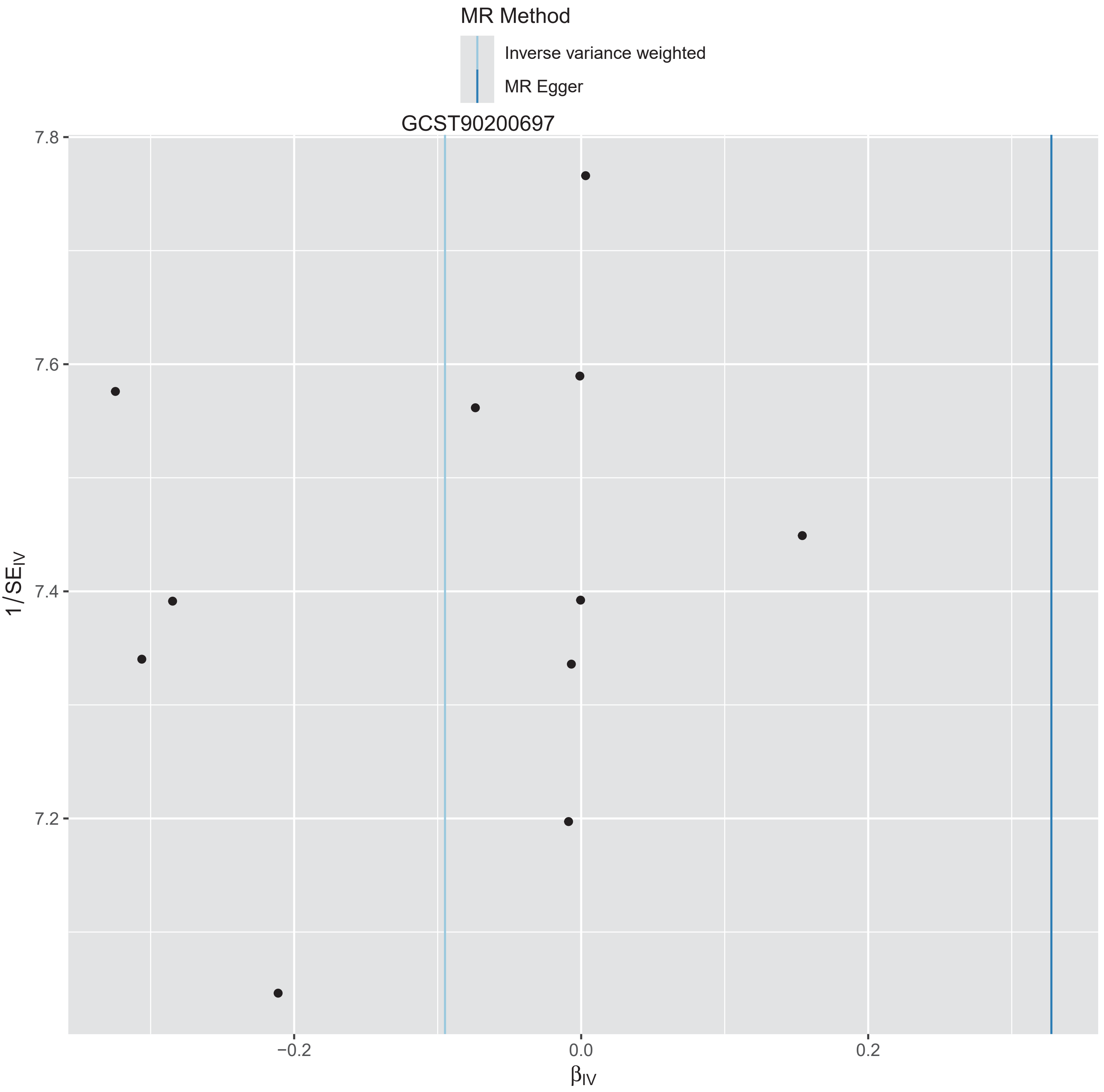
MR Method
Inverse variance weighted
MR Egger
GCST90200697
7.8
7.6
7.4
7.2
1/SE_IV
−0.2
0.0
0.2
β_IV
