# Supplementary material for: Dissecting Causal Relationships Between Gut Microbiota, Plasma Metabolites and Bladder Cancer: A Two‐Step Mendelian Randomization Study
Source: Health Sci Rep. 2025 Sep 9;8(9):e71206. doi: 10.1002/hsr2.71206 (PMC12420358; doi:10.1002/hsr2.71206)
Supplement: Supplementary file 4 — Figure S4: Leave‐one‐out analysis for MR causal effects of plasma metabolite on g_Streptococcus. [file HSR2-8-e71206-s007.pdf]

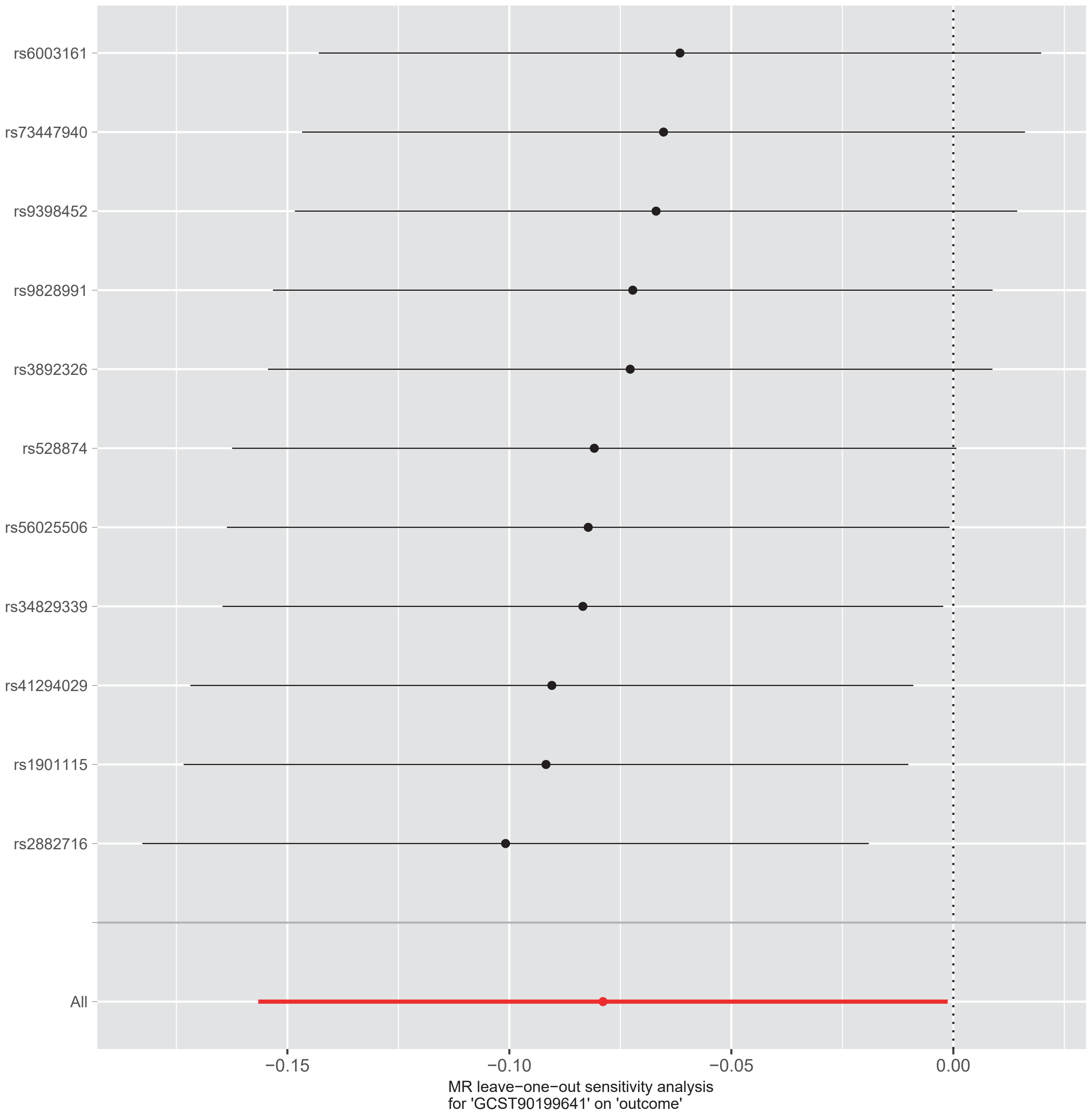

rs6003161
rs73447940
rs9398452
rs9828991
rs3892326
rs528874
rs56025506
rs34829339
rs41294029
rs1901115
rs2882716
All
−0.15
−0.10
−0.05
0.00
MR leave−one−out sensitivity analysis
for 'GCST90199641' on 'outcome'

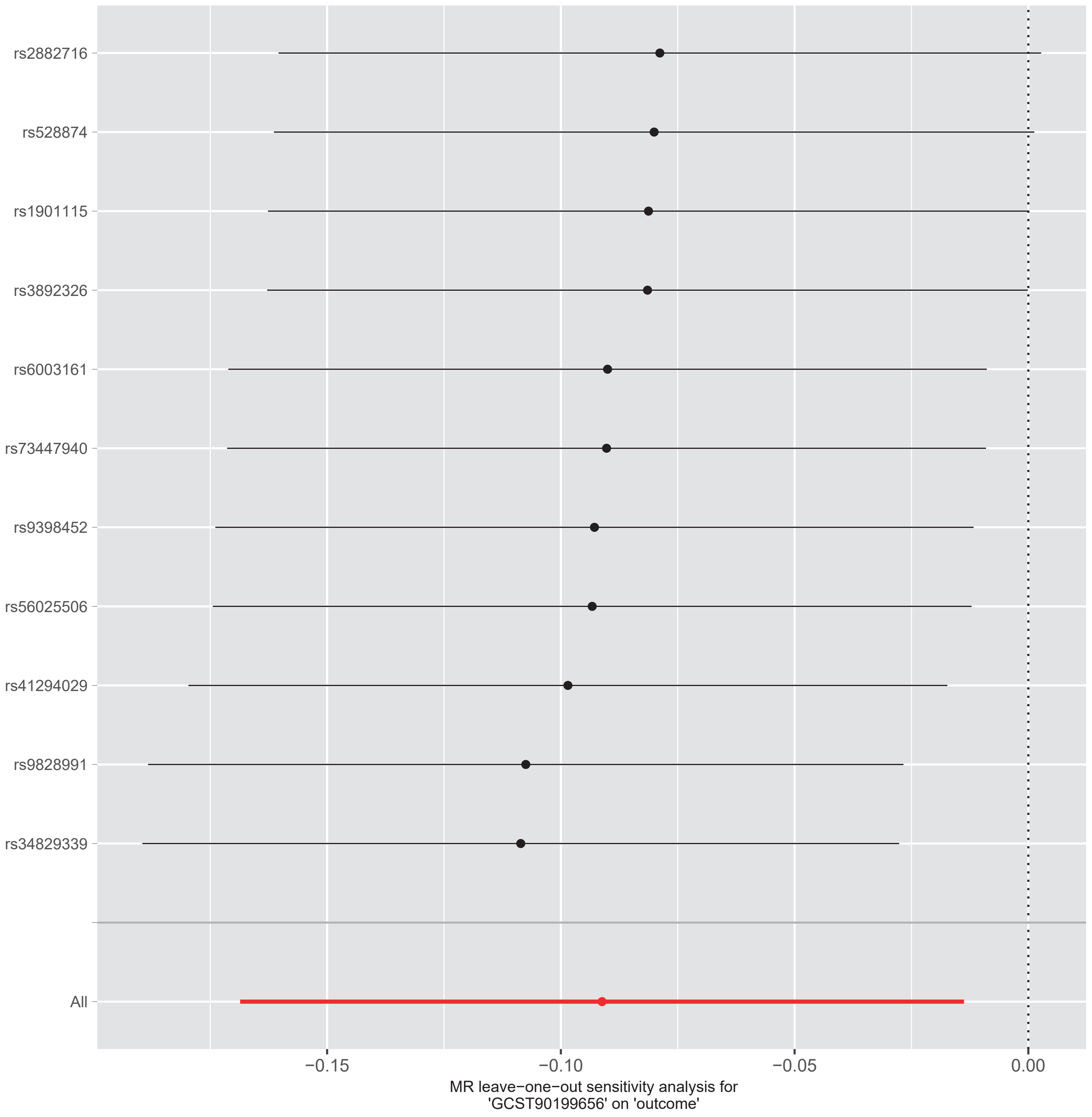
rs2882716
rs528874
rs1901115
rs3892326
rs6003161
rs73447940
rs9398452
rs56025506
rs41294029
rs9828991
rs34829339
All
−0.15
−0.10
−0.05
0.00
MR leave−one−out sensitivity analysis for
'GCST90199656' on 'outcome'

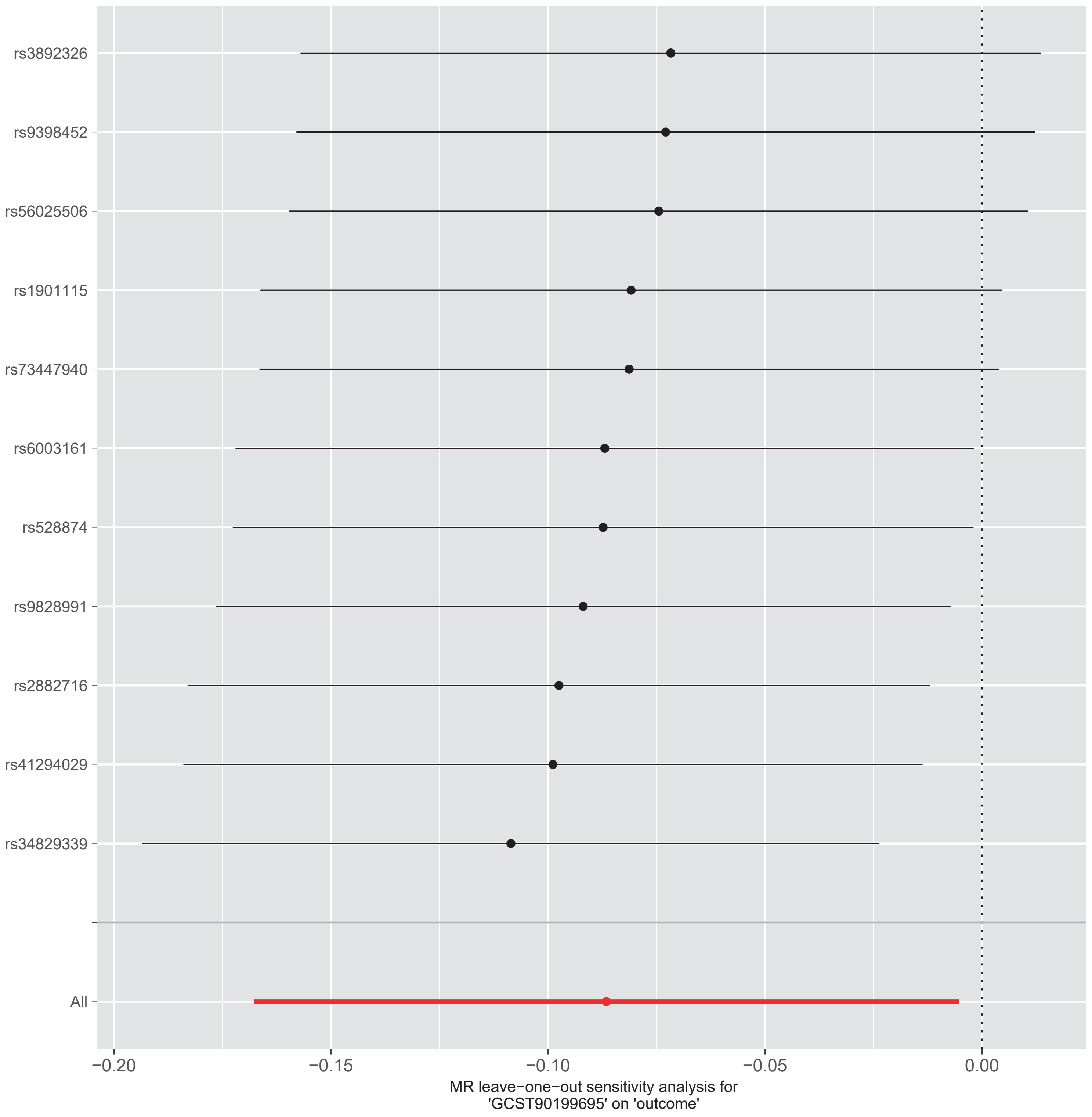
rs3892326
rs9398452
rs56025506
rs1901115
rs73447940
rs6003161
rs528874
rs9828991
rs2882716
rs41294029
rs34829339
All
−0.20
−0.15
−0.10
−0.05
0.00
MR leave−one−out sensitivity analysis for
'GCST90199695' on 'outcome'

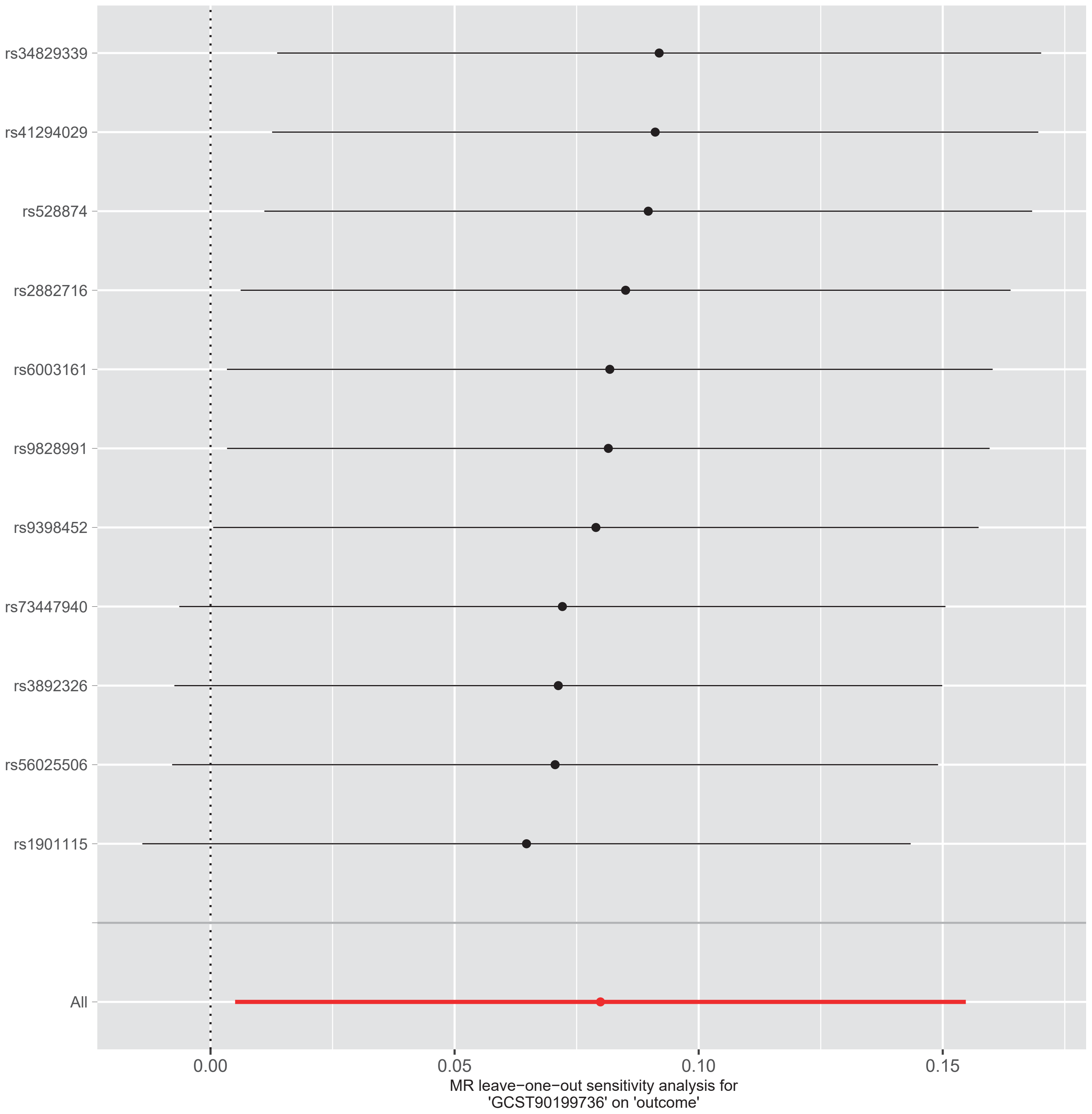
rs34829339
rs41294029
rs528874
rs2882716
rs6003161
rs9828991
rs9398452
rs73447940
rs3892326
rs56025506
rs1901115
All
0.00
0.05
0.10
0.15
MR leave−one−out sensitivity analysis for
'GCST90199736' on 'outcome'

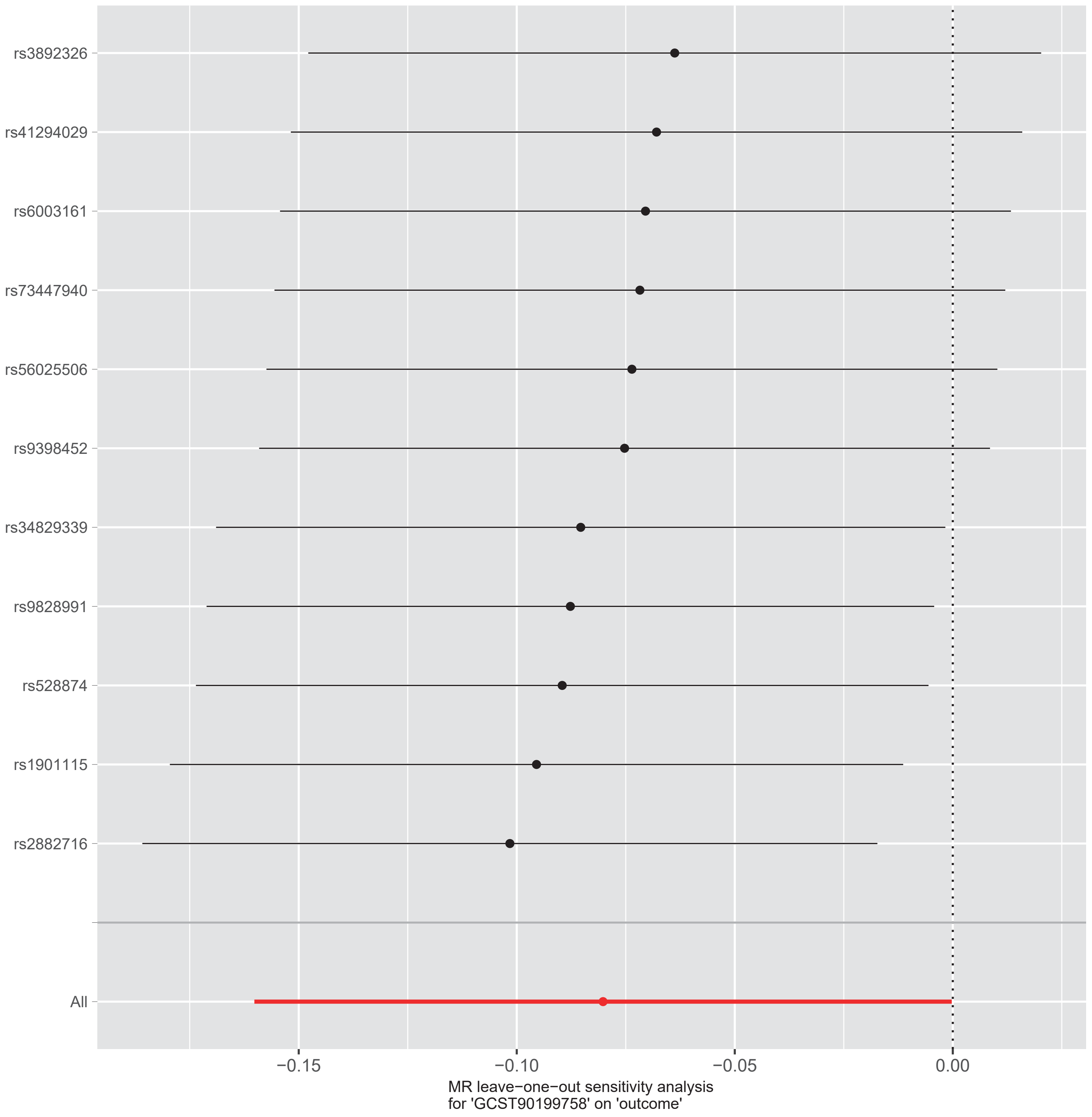
rs3892326
rs41294029
rs6003161
rs73447940
rs56025506
rs9398452
rs34829339
rs9828991
rs528874
rs1901115
rs2882716
All
−0.15
−0.10
−0.05
0.00
MR leave−one−out sensitivity analysis
for 'GCST90199758' on 'outcome'

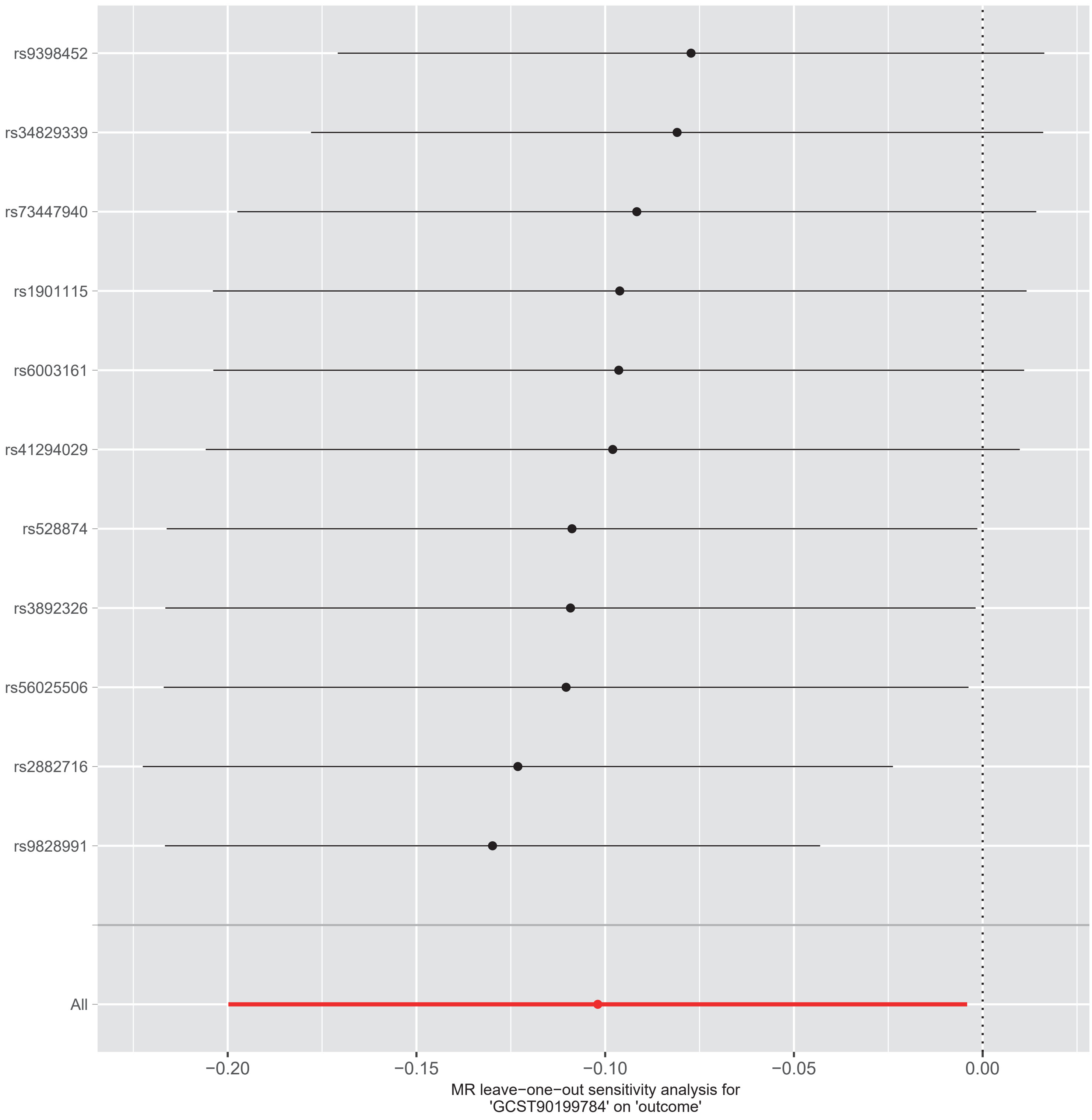
rs9398452
rs34829339
rs73447940
rs1901115
rs6003161
rs41294029
rs528874
rs3892326
rs56025506
rs2882716
rs9828991
All
−0.20
−0.15
−0.10
−0.05
0.00
MR leave−one−out sensitivity analysis for
'GCST90199784' on 'outcome'

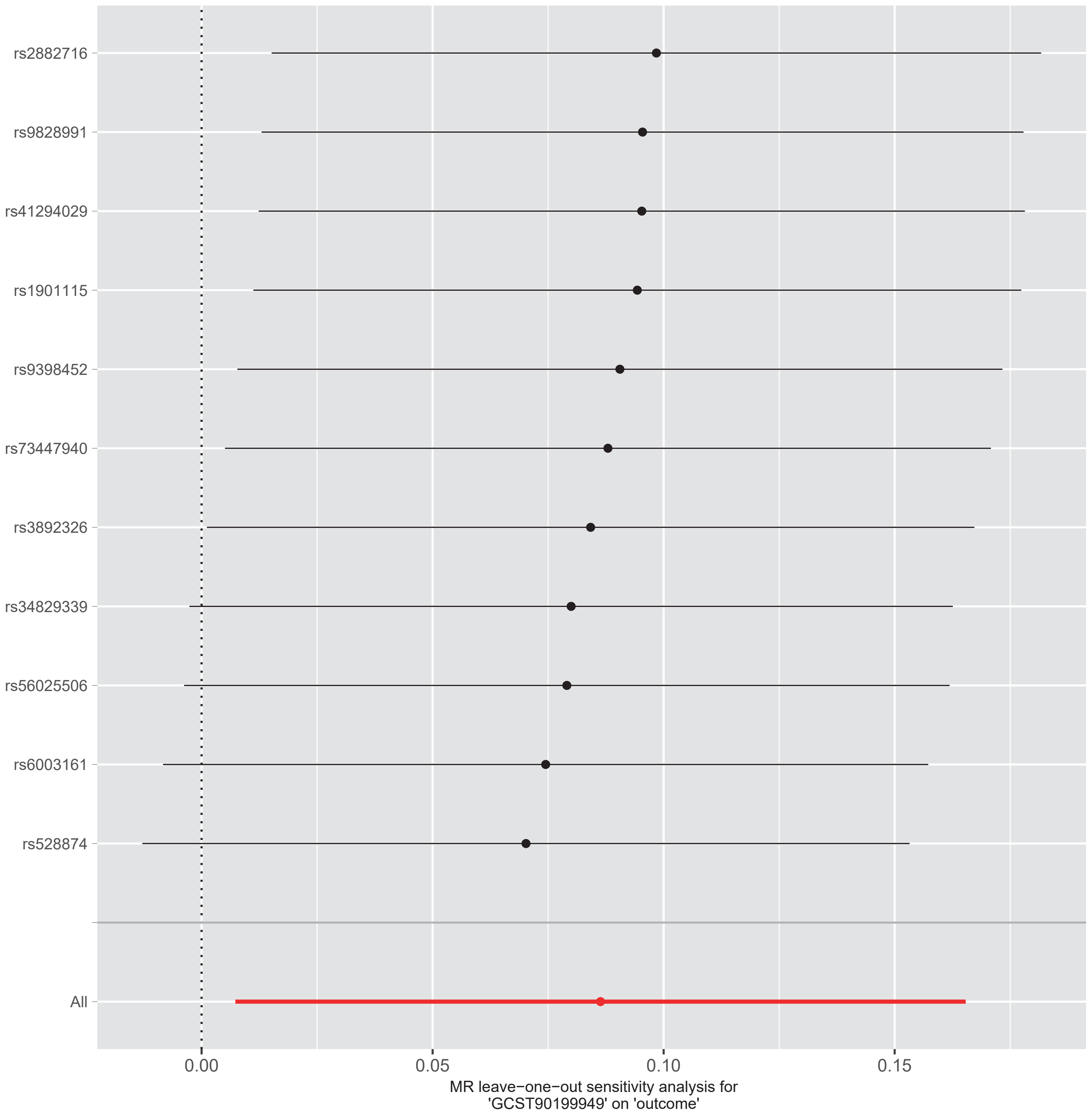
rs2882716
rs9828991
rs41294029
rs1901115
rs9398452
rs73447940
rs3892326
rs34829339
rs56025506
rs6003161
rs528874
All
0.00
0.05
0.10
0.15
MR leave−one−out sensitivity analysis for
'GCST90199949' on 'outcome'

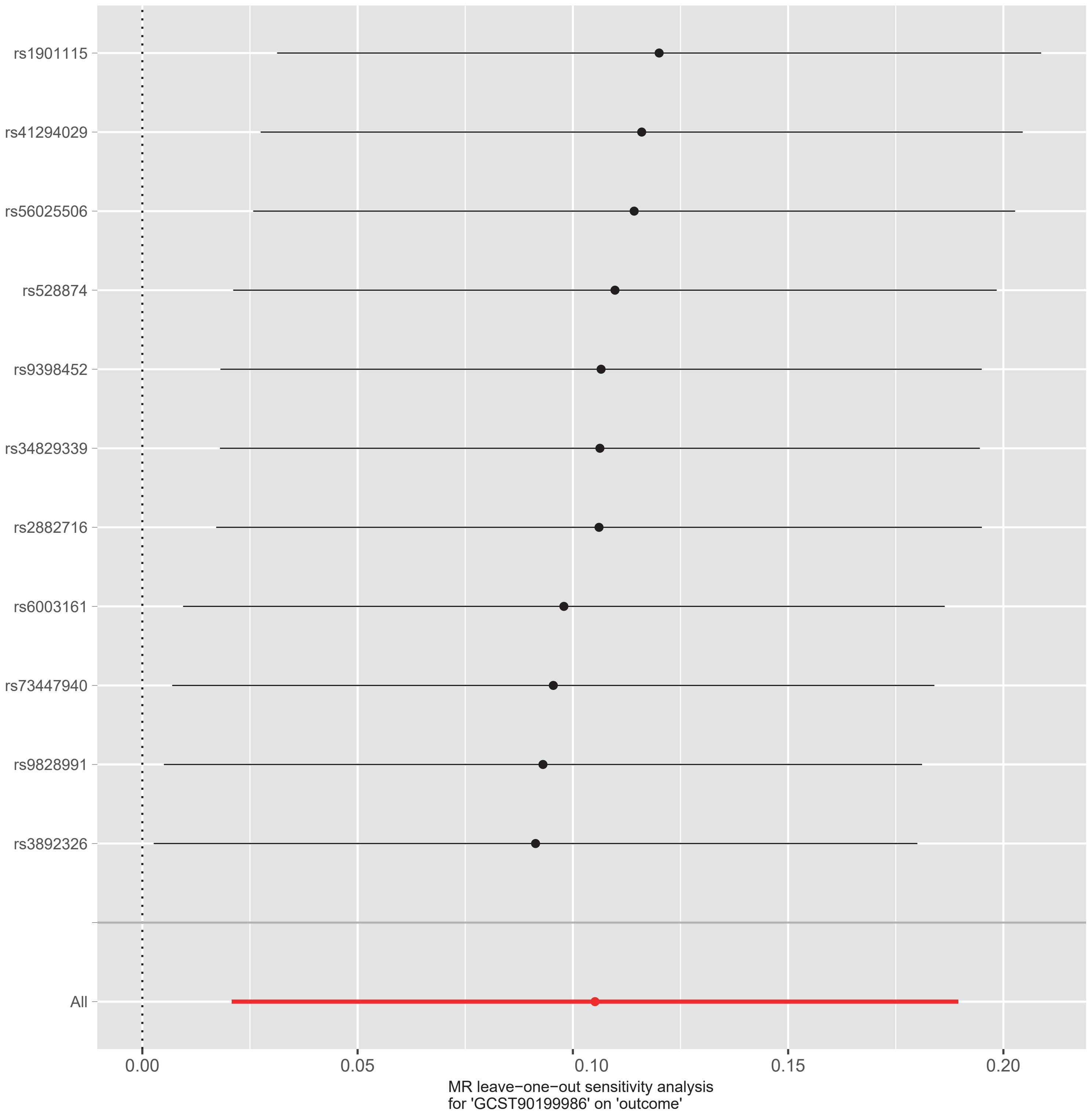

rs1901115
rs41294029
rs56025506
rs528874
rs9398452
rs34829339
rs2882716
rs6003161
rs73447940
rs9828991
rs3892326
All
0.00
0.05
0.10
0.15
0.20
MR leave−one−out sensitivity analysis
for 'GCST90199986' on 'outcome'

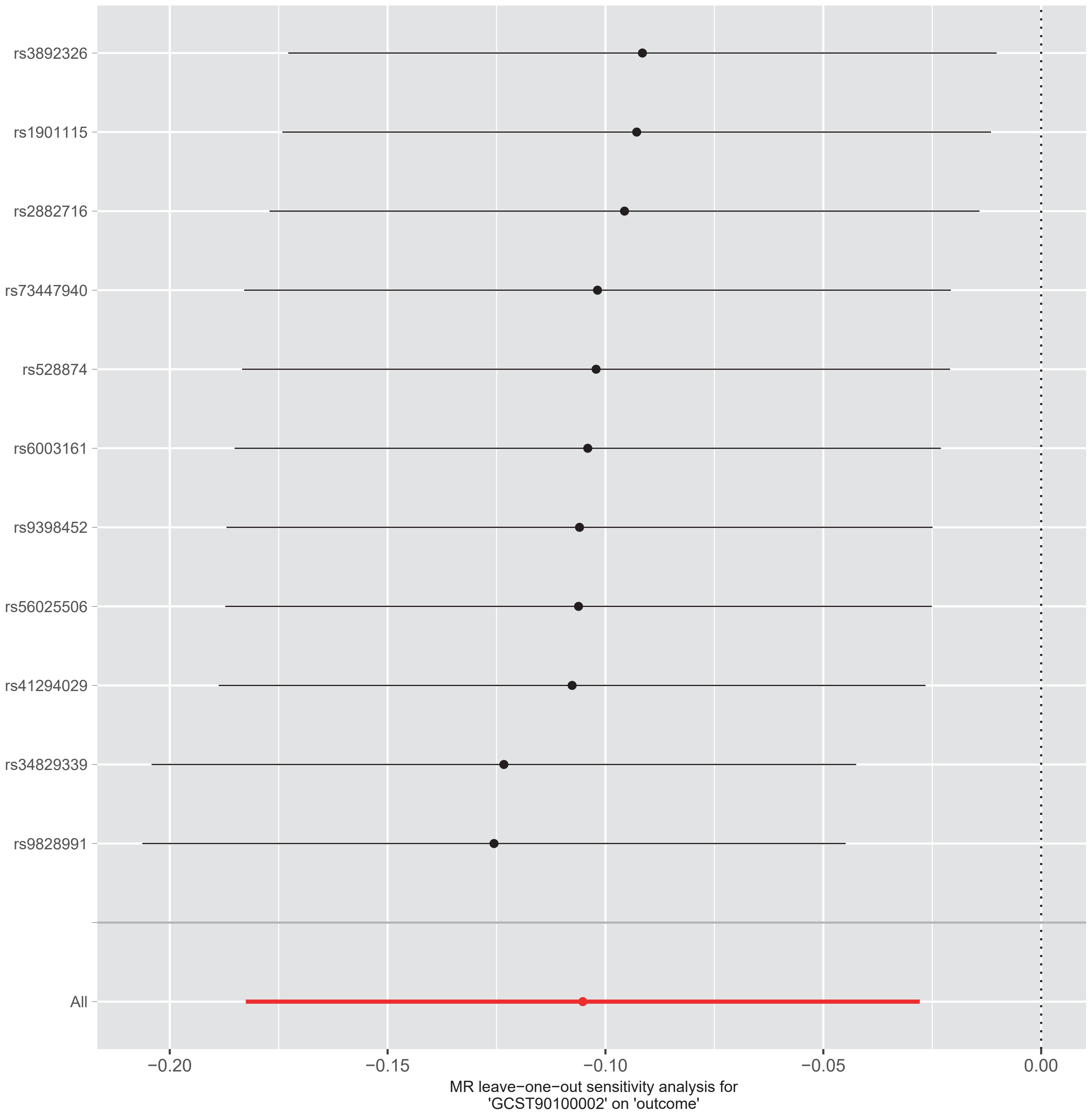
rs3892326
rs1901115
rs2882716
rs73447940
rs528874
rs6003161
rs9398452
rs56025506
rs41294029
rs34829339
rs9828991
All
−0.20
−0.15
−0.10
−0.05
0.00
MR leave−one−out sensitivity analysis for
'GCST90100002' on 'outcome'

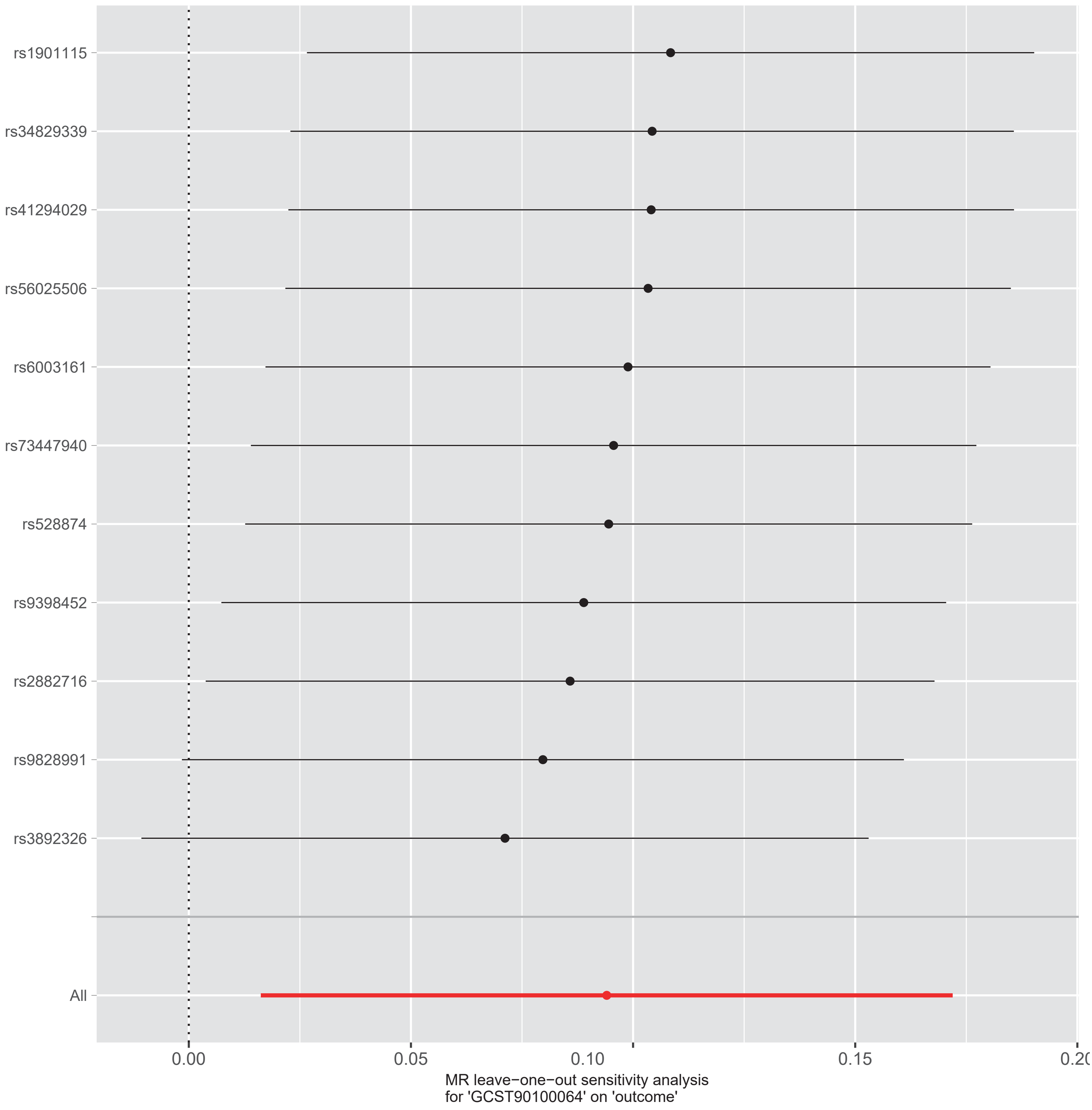
rs1901115
rs34829339
rs41294029
rs56025506
rs6003161
rs73447940
rs528874
rs9398452
rs2882716
rs9828991
rs3892326
All
0.00
0.05
0.10
0.15
MR leave−one−out sensitivity analysis
for 'GCST90100064' on 'outcome'

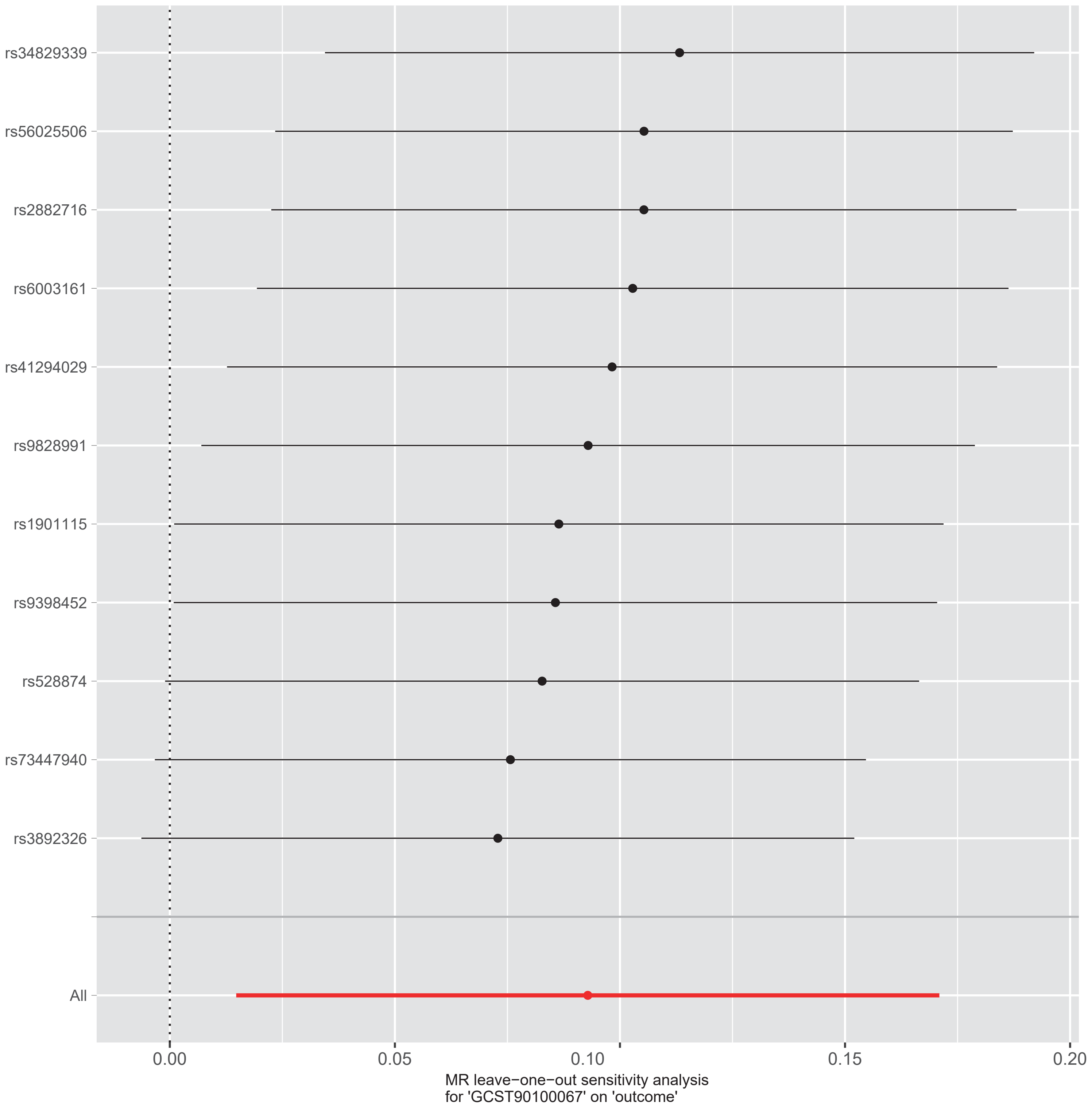
rs34829339
rs56025506
rs2882716
rs6003161
rs41294029
rs9828991
rs1901115
rs9398452
rs528874
rs73447940
rs3892326
All
0.00
0.05
0.10
0.15
0.20
MR leave−one−out sensitivity analysis
for 'GCST90100067' on 'outcome'

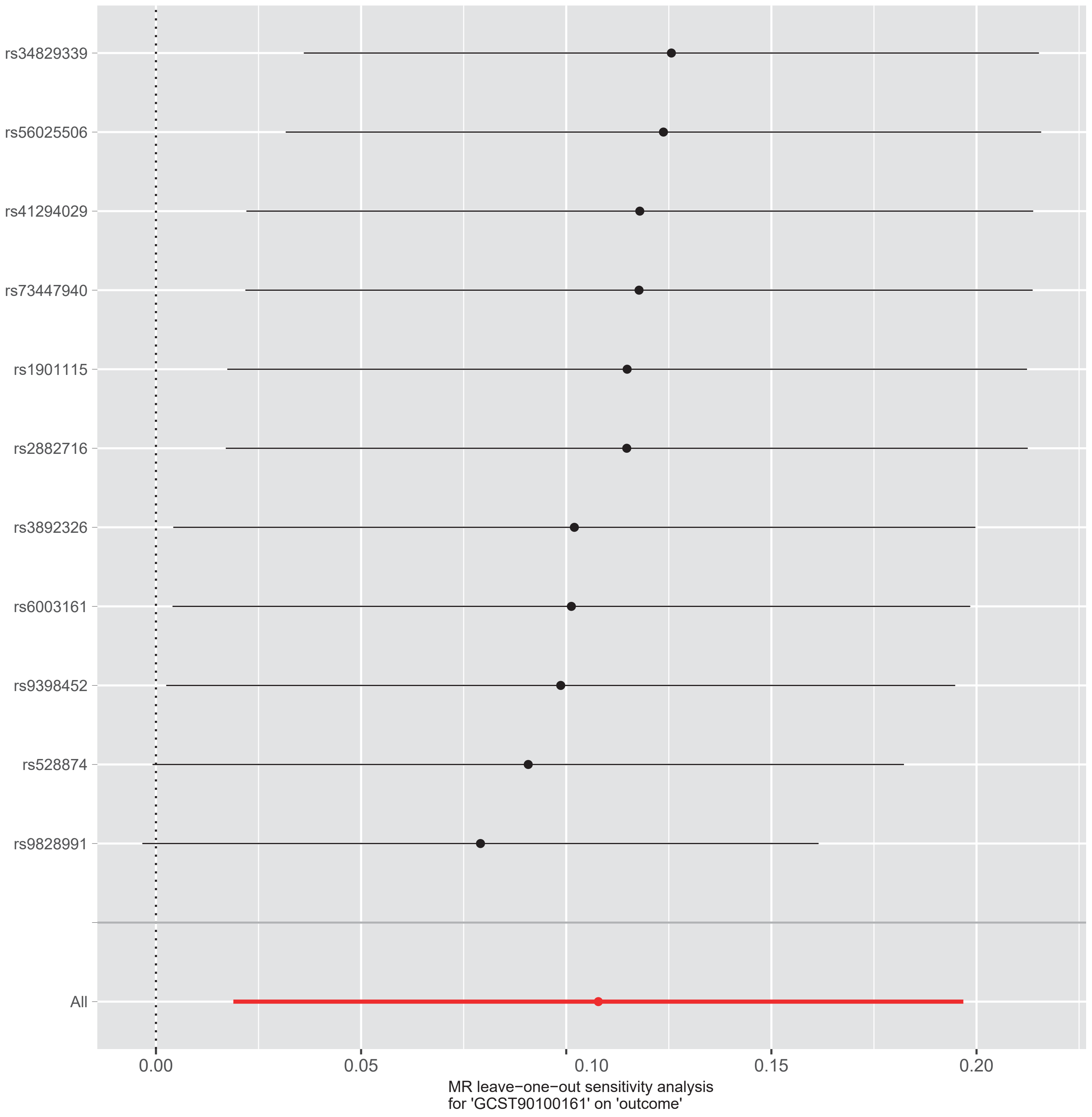
rs34829339
rs56025506
rs41294029
rs73447940
rs1901115
rs2882716
rs3892326
rs6003161
rs9398452
rs528874
rs9828991
All
0.00
0.05
0.10
0.15
0.20
MR leave−one−out sensitivity analysis
for 'GCST90100161' on 'outcome'

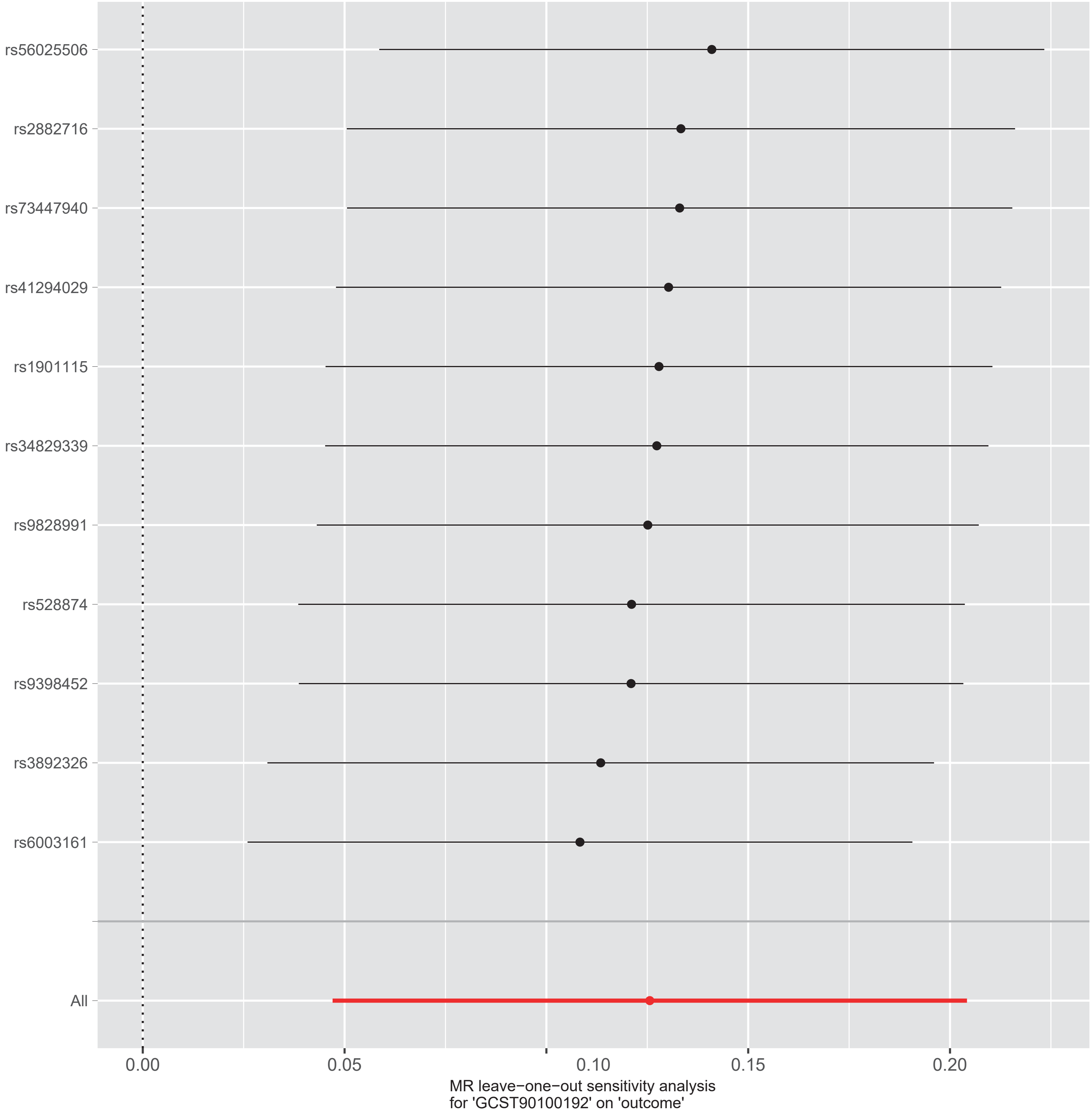

rs56025506
rs2882716
rs73447940
rs41294029
rs1901115
rs34829339
rs9828991
rs528874
rs9398452
rs3892326
rs6003161
All
0.00
0.05
0.10
0.15
0.20
MR leave−one−out sensitivity analysis
for 'GCST90100192' on 'outcome'

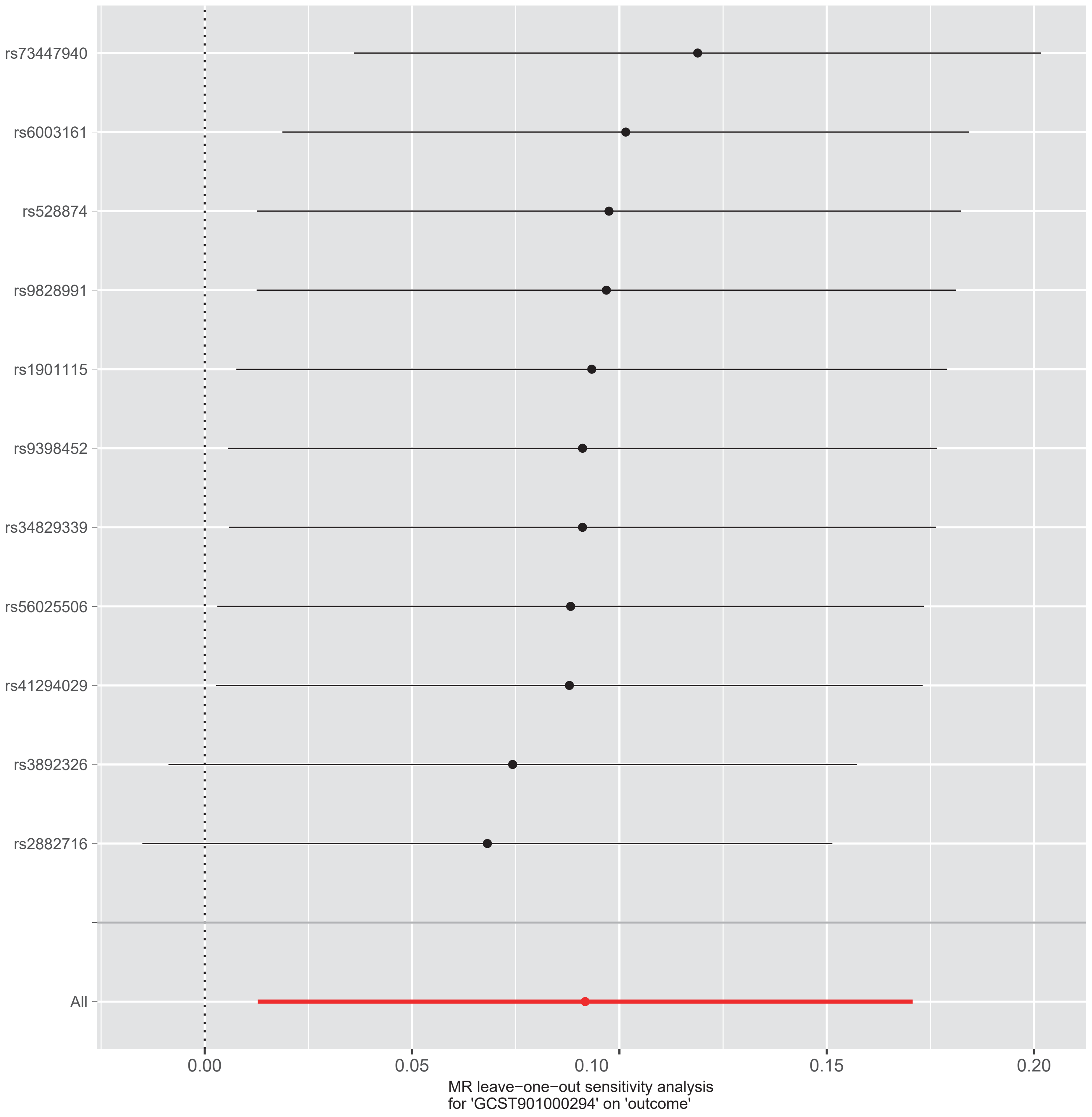

rs73447940
rs6003161
rs528874
rs9828991
rs1901115
rs9398452
rs34829339
rs56025506
rs41294029
rs3892326
rs2882716
All
0.00
0.05
0.10
0.15
0.20
MR leave−one−out sensitivity analysis
for 'GCST901000294' on 'outcome'

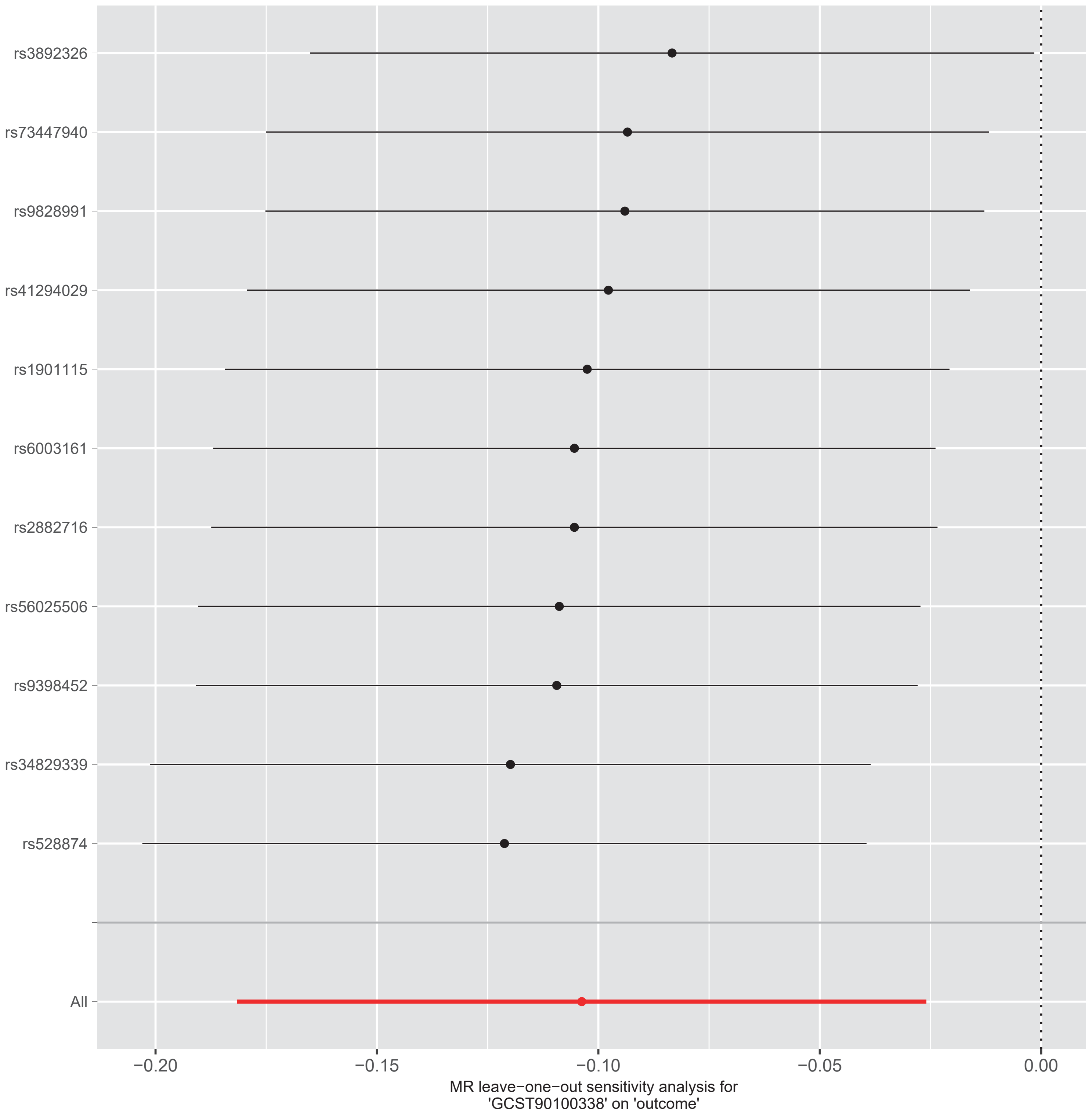
rs3892326
rs73447940
rs9828991
rs41294029
rs1901115
rs6003161
rs2882716
rs56025506
rs9398452
rs34829339
rs528874
All
−0.20
−0.15
−0.10
−0.05
0.00
MR leave−one−out sensitivity analysis for
'GCST90100338' on 'outcome'

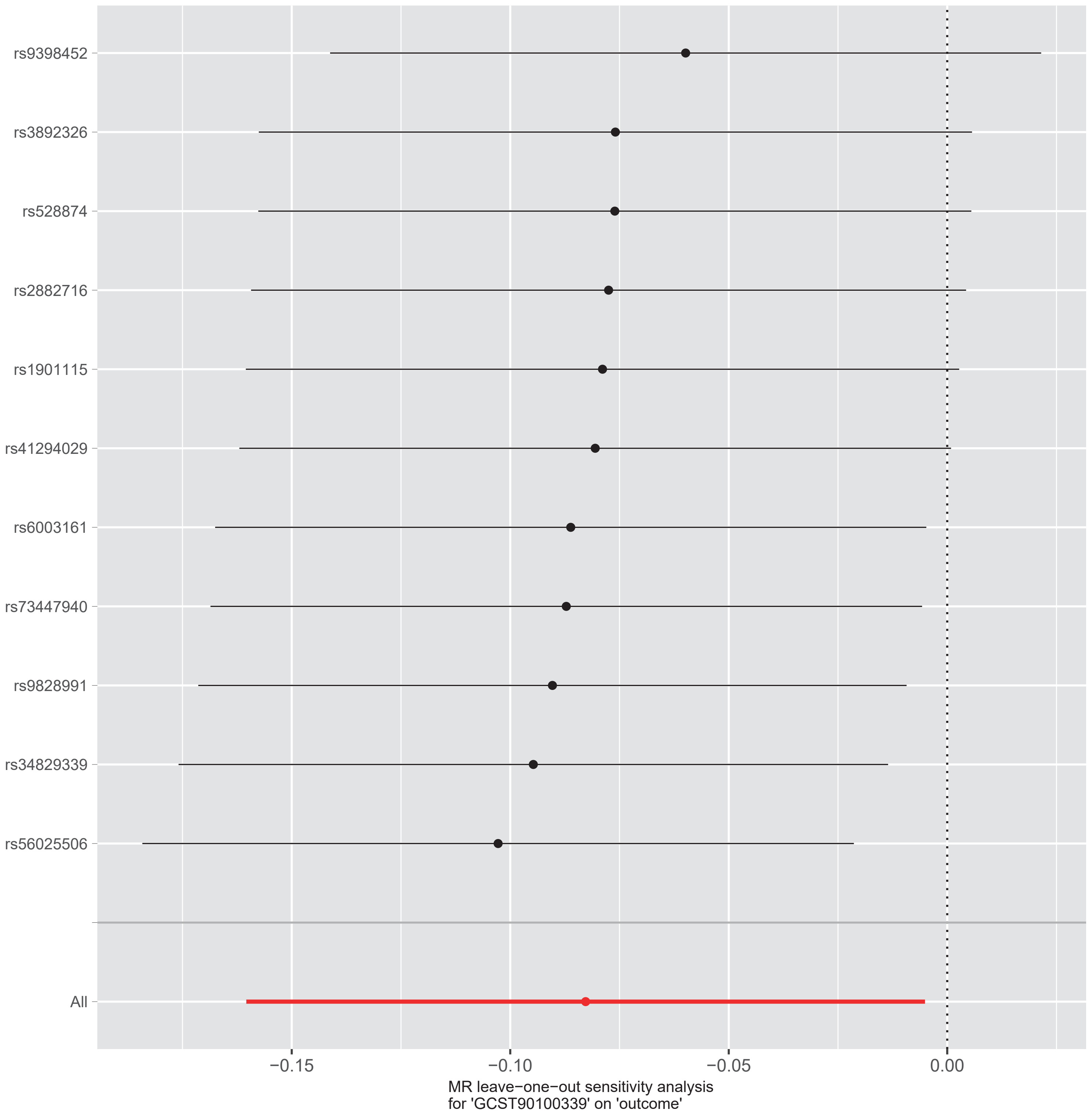
rs9398452
rs3892326
rs528874
rs2882716
rs1901115
rs41294029
rs6003161
rs73447940
rs9828991
rs34829339
rs56025506
All
−0.15
−0.10
−0.05
0.00
MR leave−one−out sensitivity analysis
for 'GCST90100339' on 'outcome'

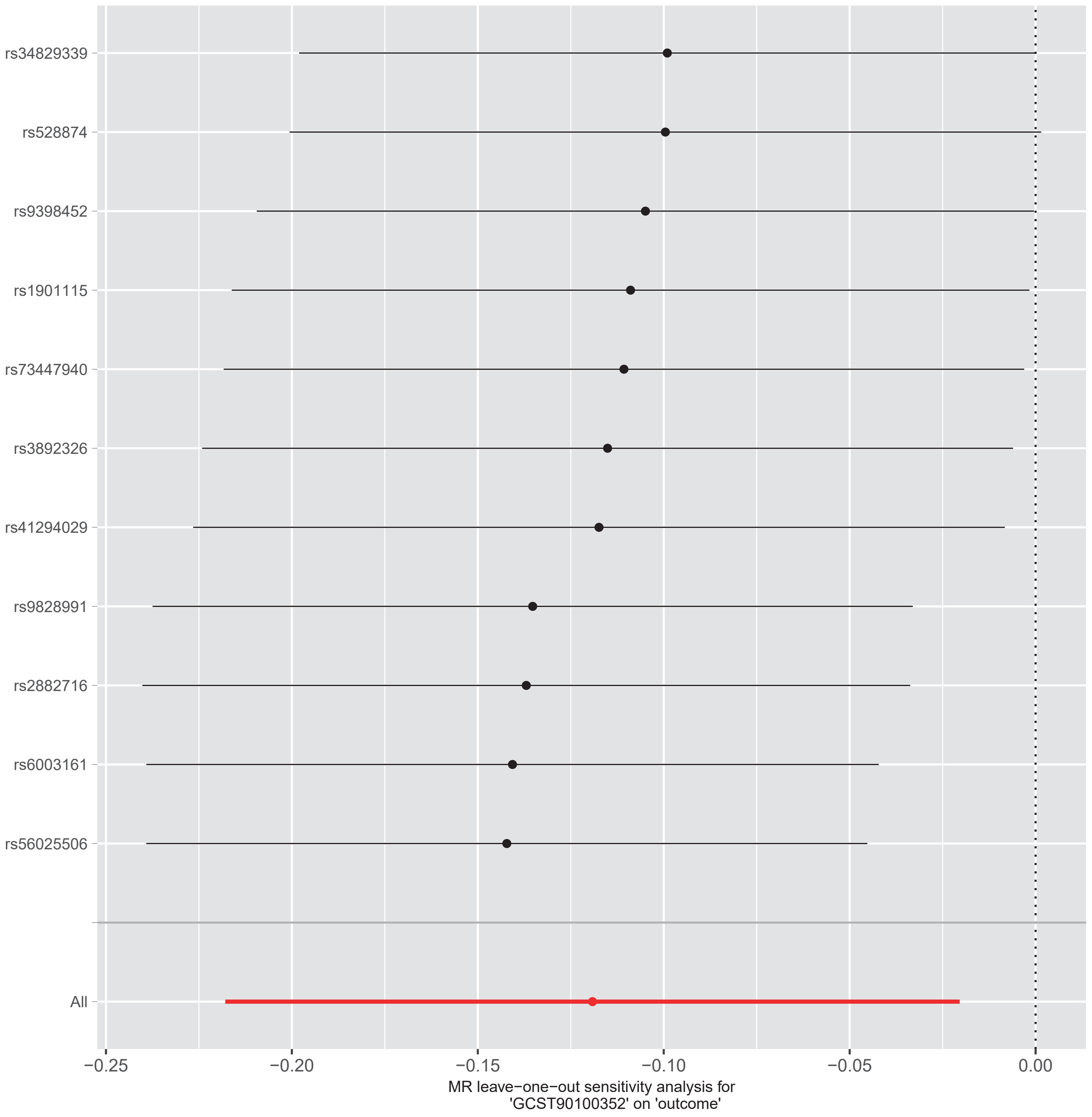
rs34829339
rs528874
rs9398452
rs1901115
rs73447940
rs3892326
rs41294029
rs9828991
rs2882716
rs6003161
rs56025506
All
−0.25
−0.20
−0.15
−0.10
−0.05
0.00
MR leave−one−out sensitivity analysis for
'GCST90100352' on 'outcome'

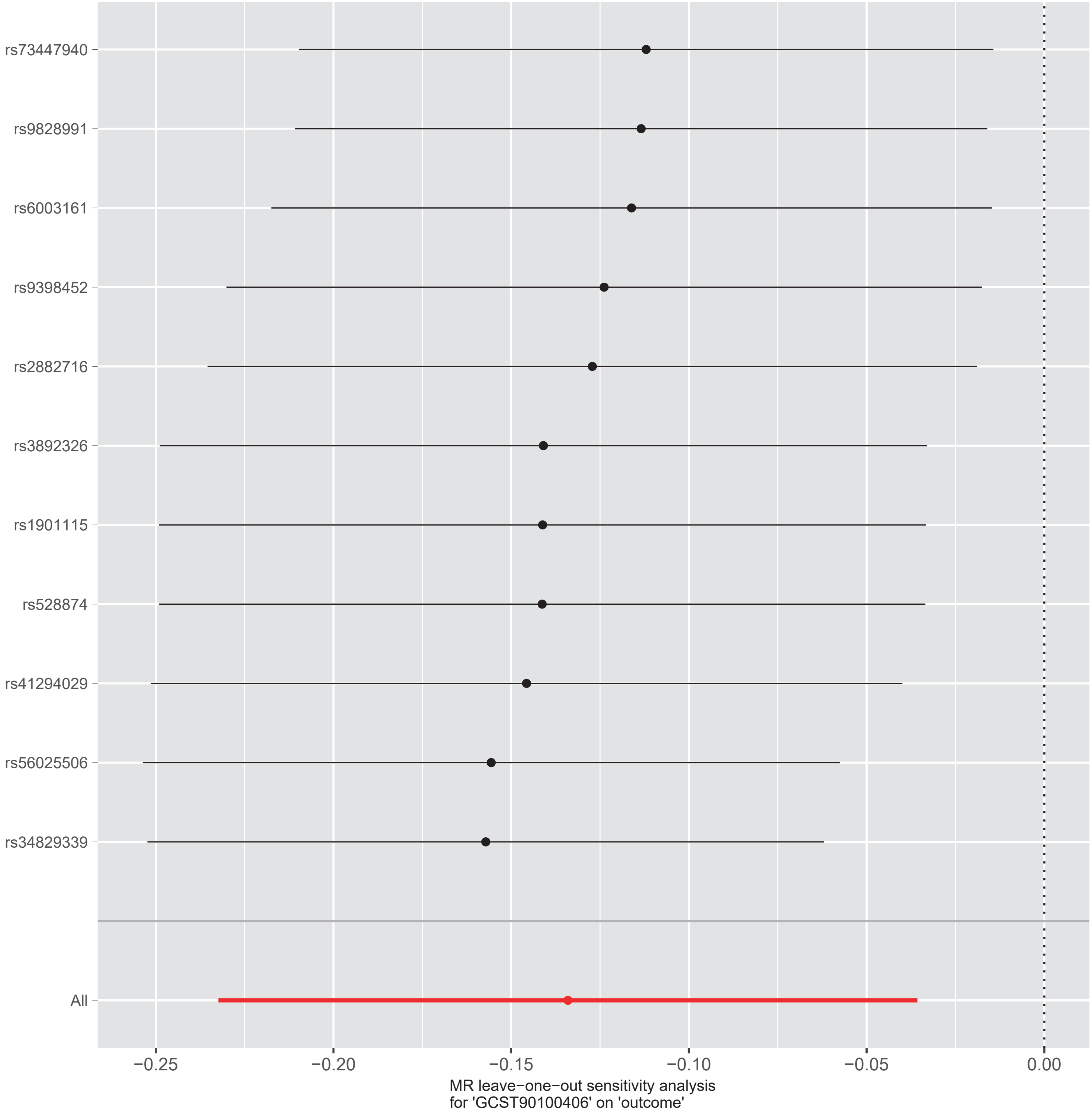

rs73447940
rs9828991
rs6003161
rs9398452
rs2882716
rs3892326
rs1901115
rs528874
rs41294029
rs56025506
rs34829339
All
−0.25
−0.20
−0.15
−0.10
−0.05
0.00
MR leave−one−out sensitivity analysis
for 'GCST90100406' on 'outcome'

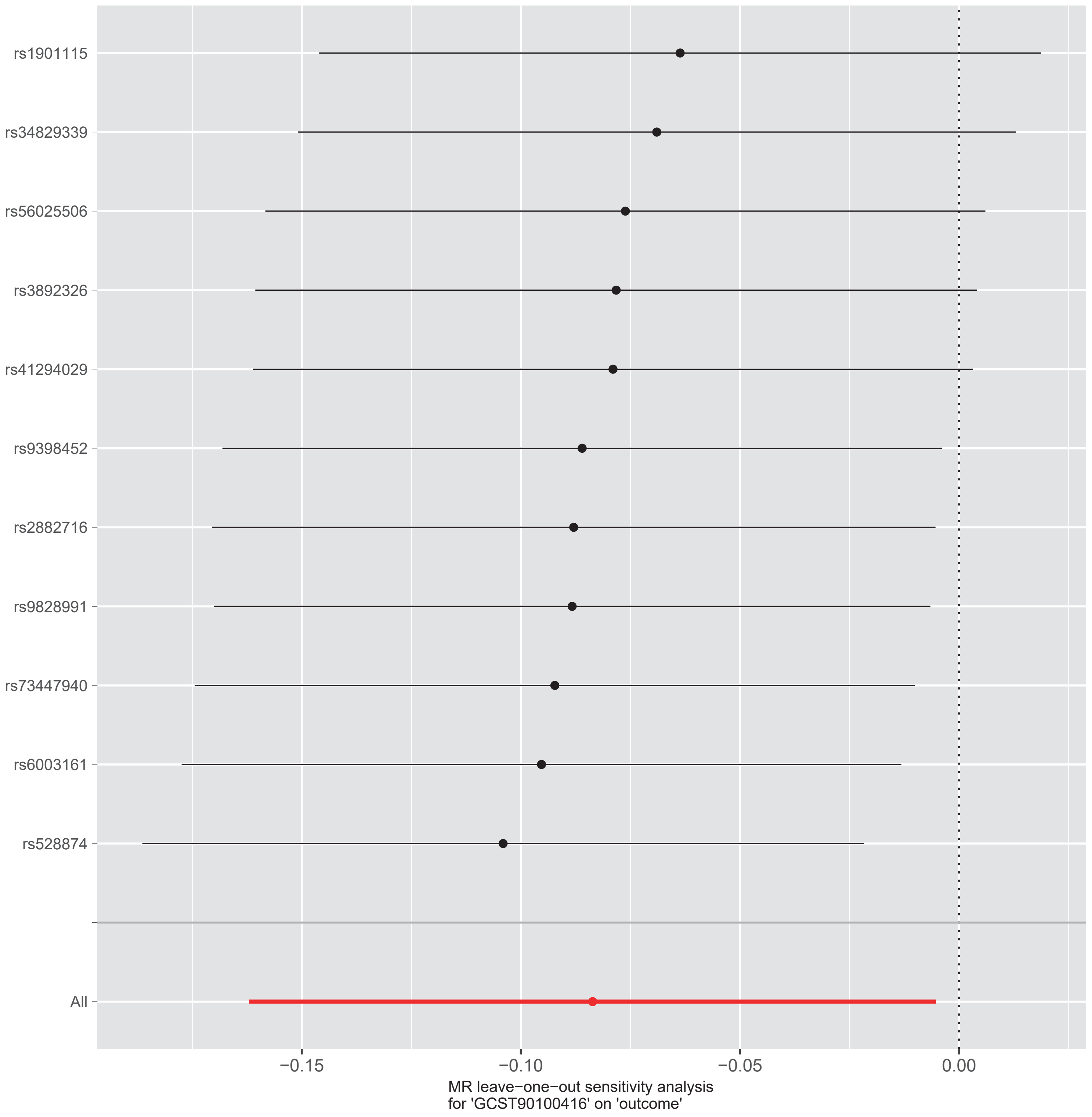
rs1901115
rs34829339
rs56025506
rs3892326
rs41294029
rs9398452
rs2882716
rs9828991
rs73447940
rs6003161
rs528874
All
−0.15
−0.10
−0.05
0.00
MR leave−one−out sensitivity analysis
for 'GCST90100416' on 'outcome'

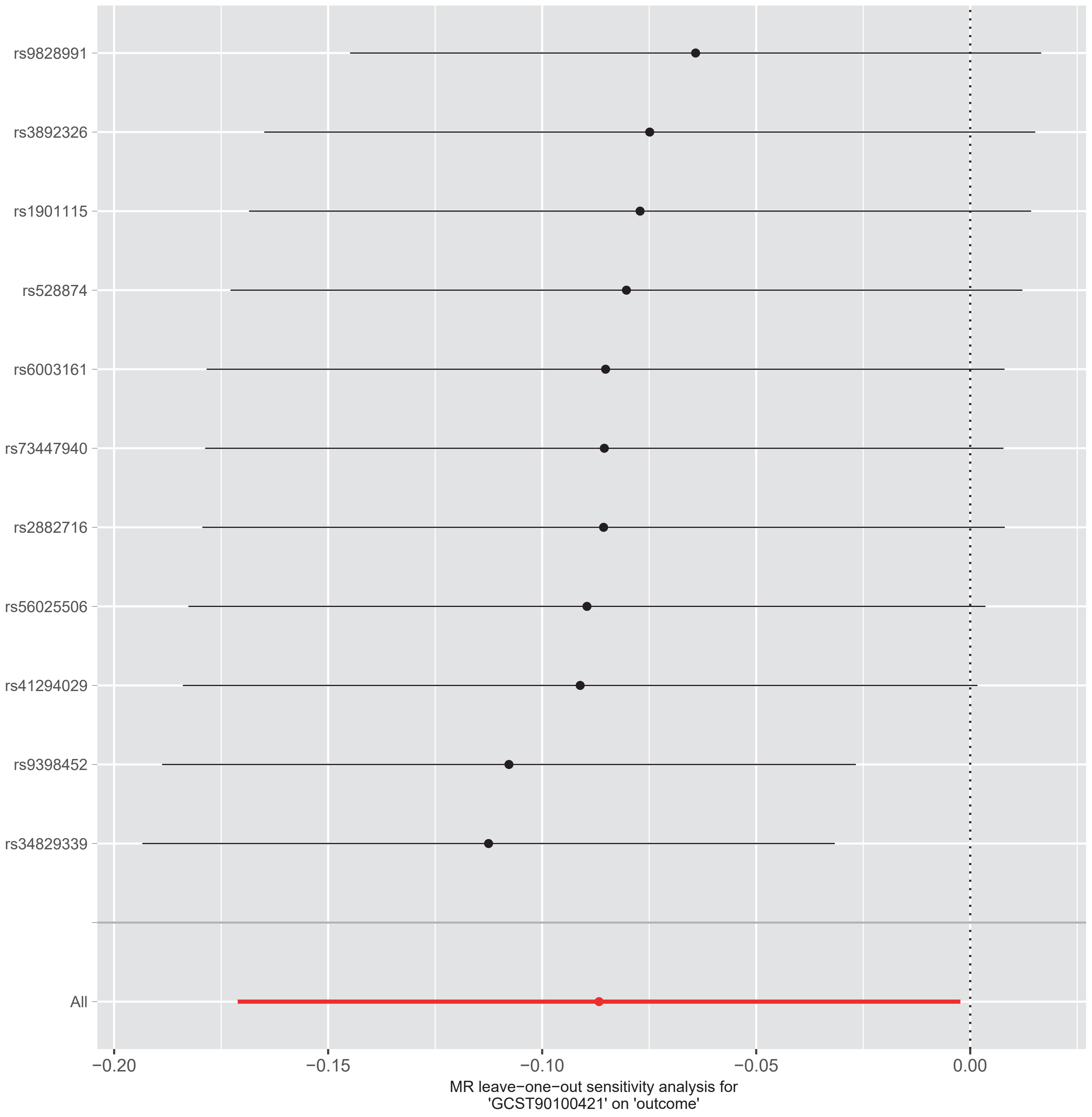
rs9828991
rs3892326
rs1901115
rs528874
rs6003161
rs73447940
rs2882716
rs56025506
rs41294029
rs9398452
rs34829339
All
−0.20
−0.15
−0.10
−0.05
0.00
MR leave−one−out sensitivity analysis for
'GCST90100421' on 'outcome'

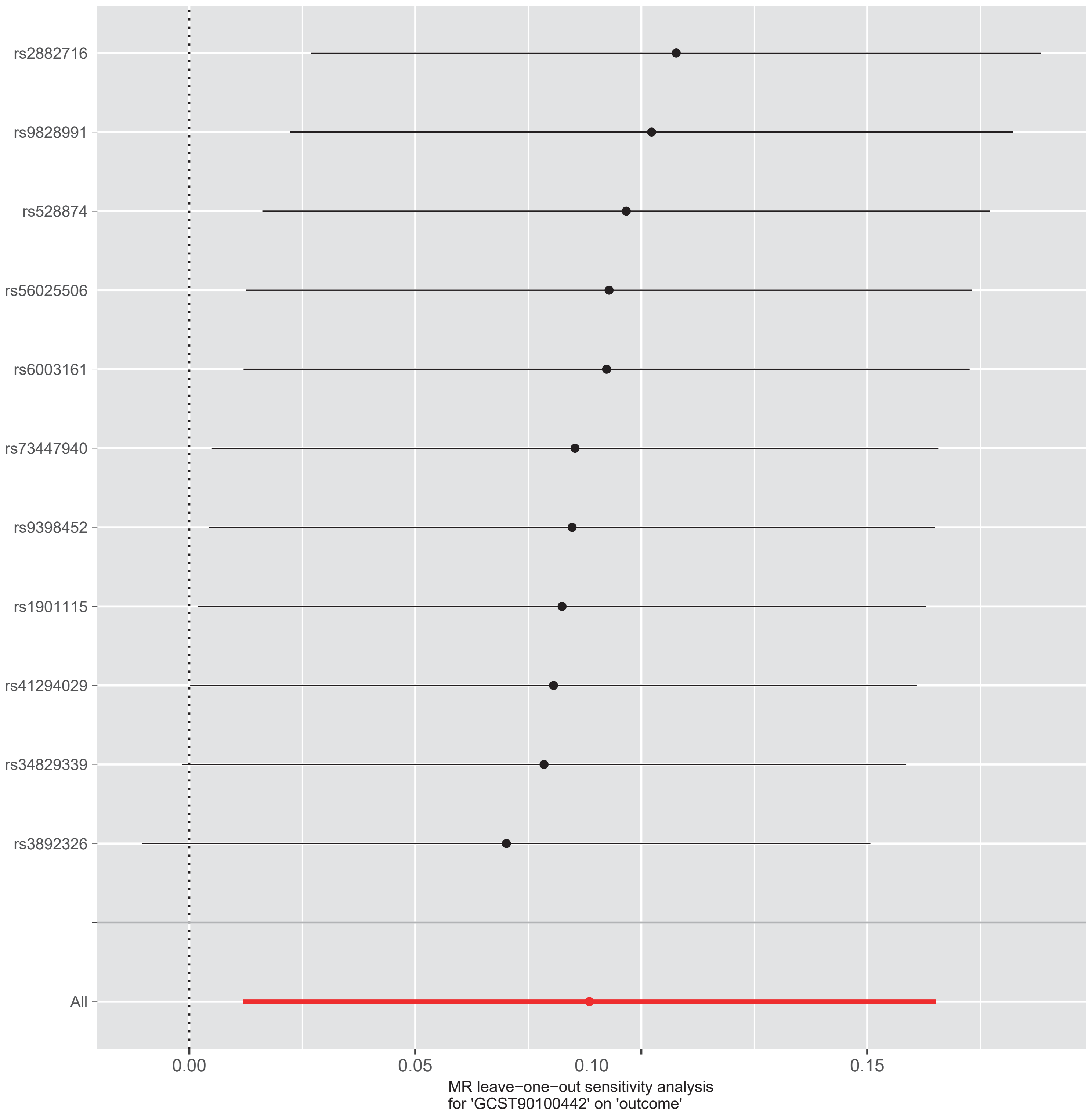
rs2882716
rs9828991
rs528874
rs56025506
rs6003161
rs73447940
rs9398452
rs1901115
rs41294029
rs34829339
rs3892326
All
0.00
0.05
0.10
0.15
MR leave−one−out sensitivity analysis
for 'GCST90100442' on 'outcome'

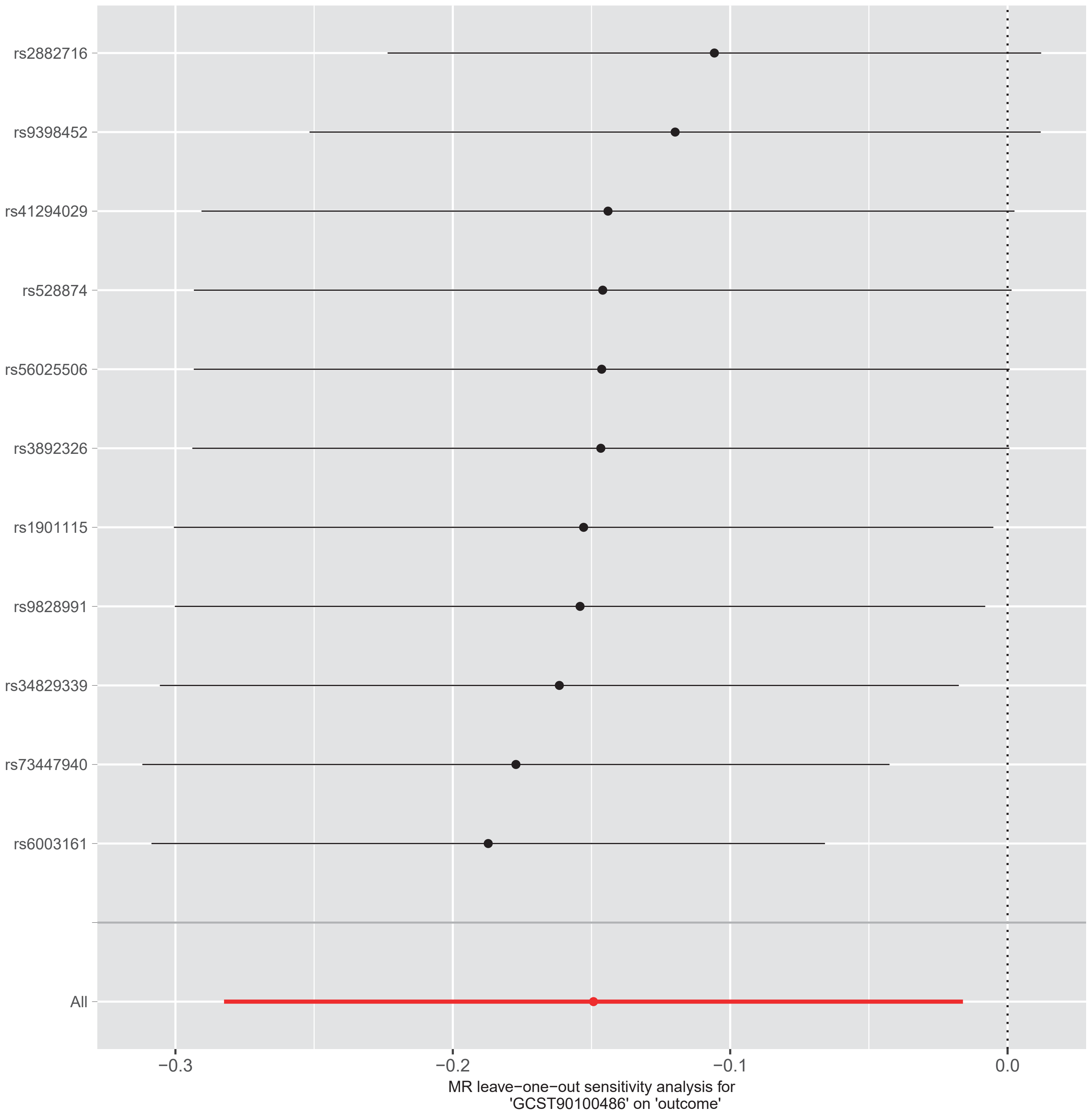
rs2882716
rs9398452
rs41294029
rs528874
rs56025506
rs3892326
rs1901115
rs9828991
rs34829339
rs73447940
rs6003161
All
−0.3
−0.2
−0.1
0.0
MR leave−one−out sensitivity analysis for
'GCST90100486' on 'outcome'

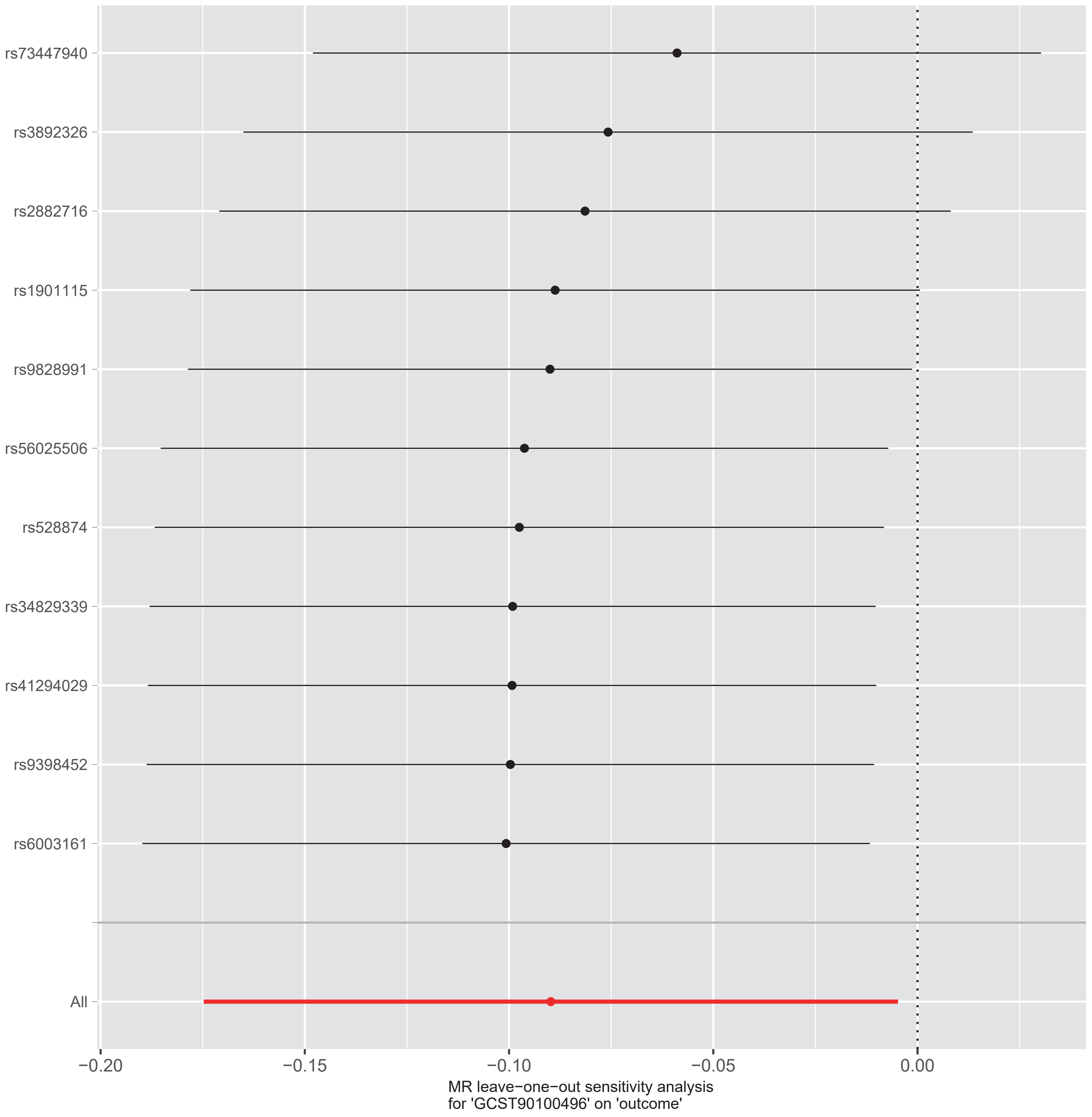

rs73447940
rs3892326
rs2882716
rs1901115
rs9828991
rs56025506
rs528874
rs34829339
rs41294029
rs9398452
rs6003161
All
−0.20
−0.15
−0.10
−0.05
0.00
MR leave−one−out sensitivity analysis
for 'GCST90100496' on 'outcome'

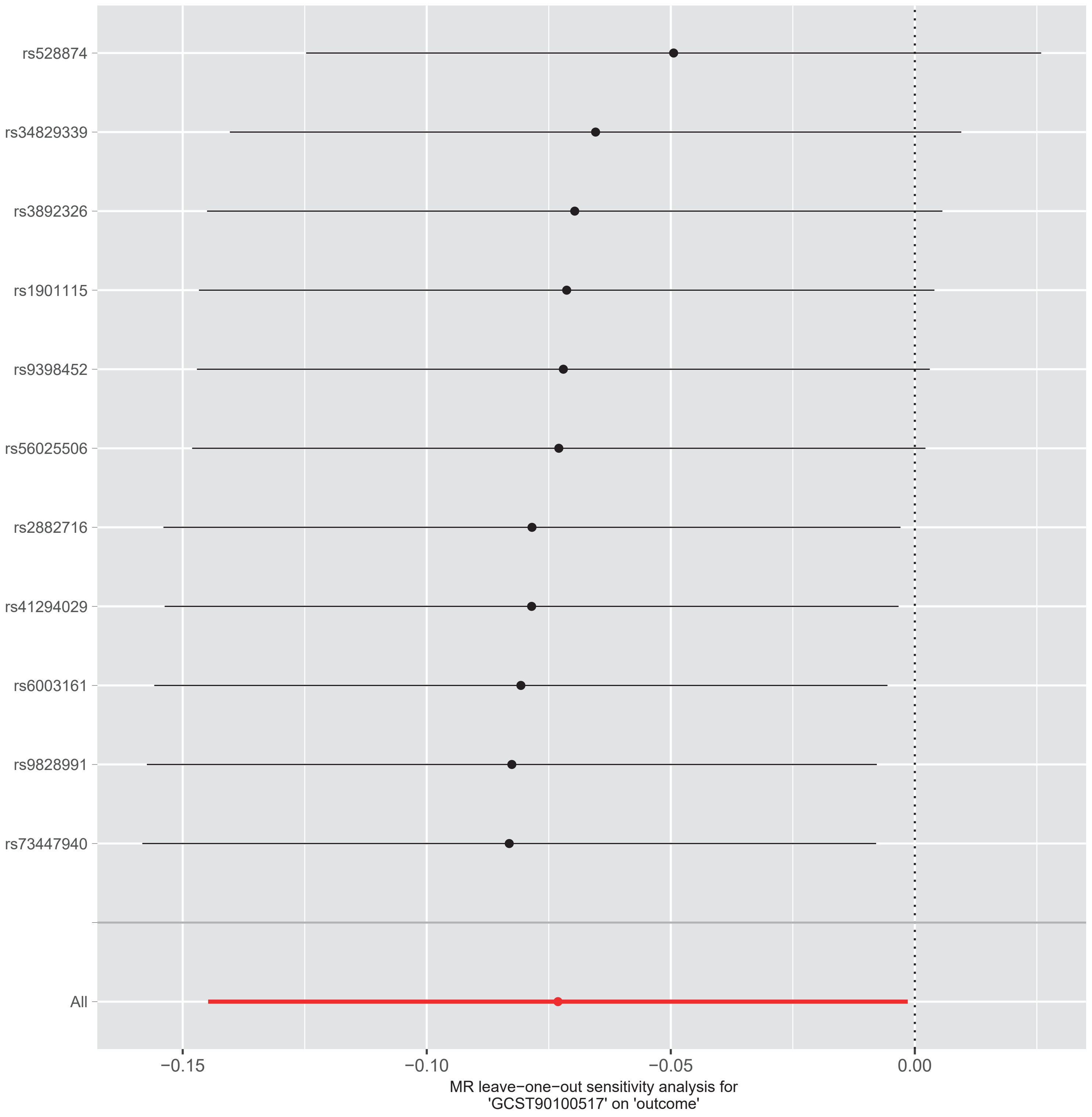
rs528874
rs34829339
rs3892326
rs1901115
rs9398452
rs56025506
rs2882716
rs41294029
rs6003161
rs9828991
rs73447940
All
−0.15
−0.10
−0.05
0.00
MR leave−one−out sensitivity analysis for
'GCST90100517' on 'outcome'

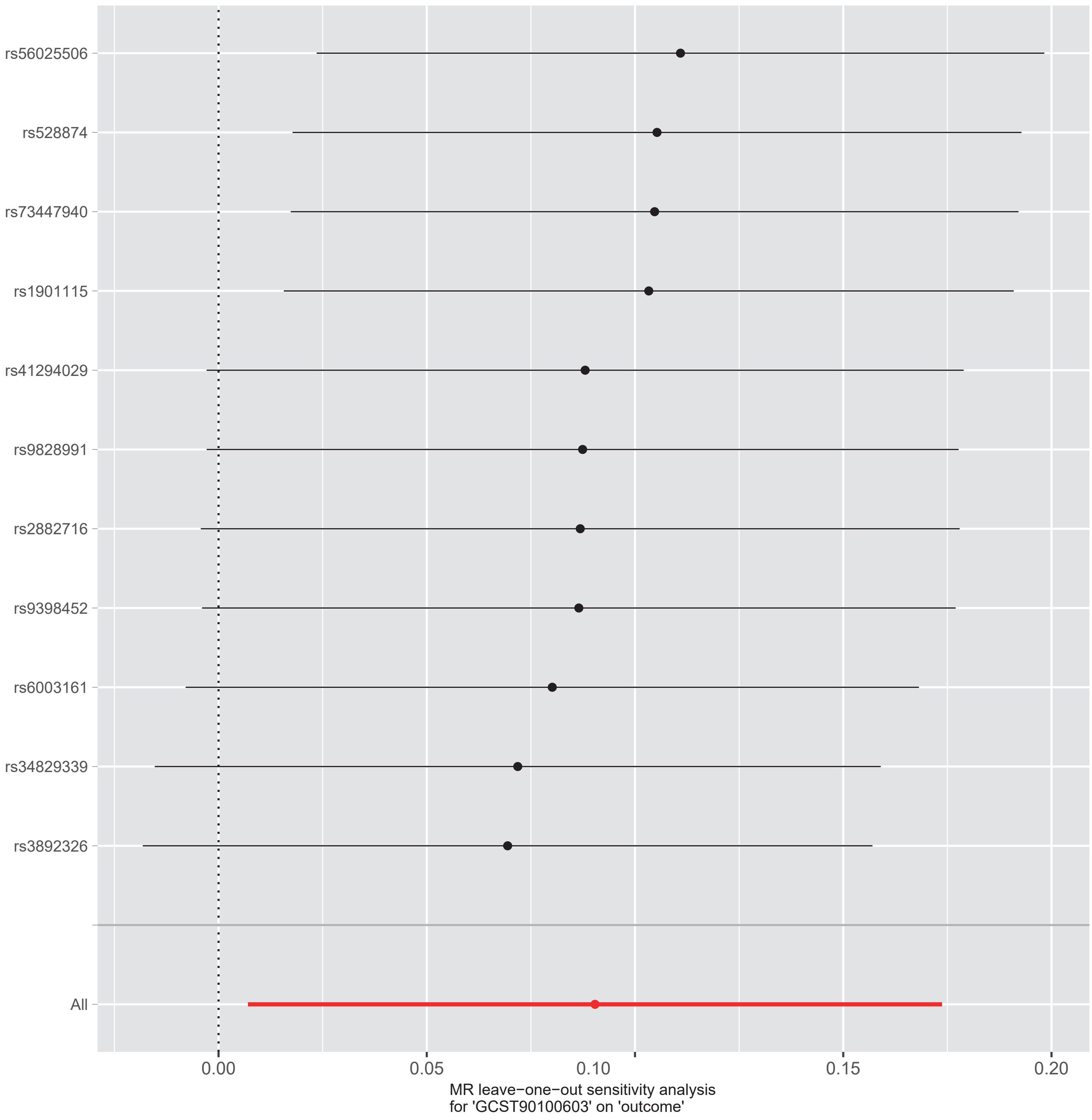
rs56025506
rs528874
rs73447940
rs1901115
rs41294029
rs9828991
rs2882716
rs9398452
rs6003161
rs34829339
rs3892326
All
0.00
0.05
0.10
0.15
0.20
MR leave−one−out sensitivity analysis
for 'GCST90100603' on 'outcome'

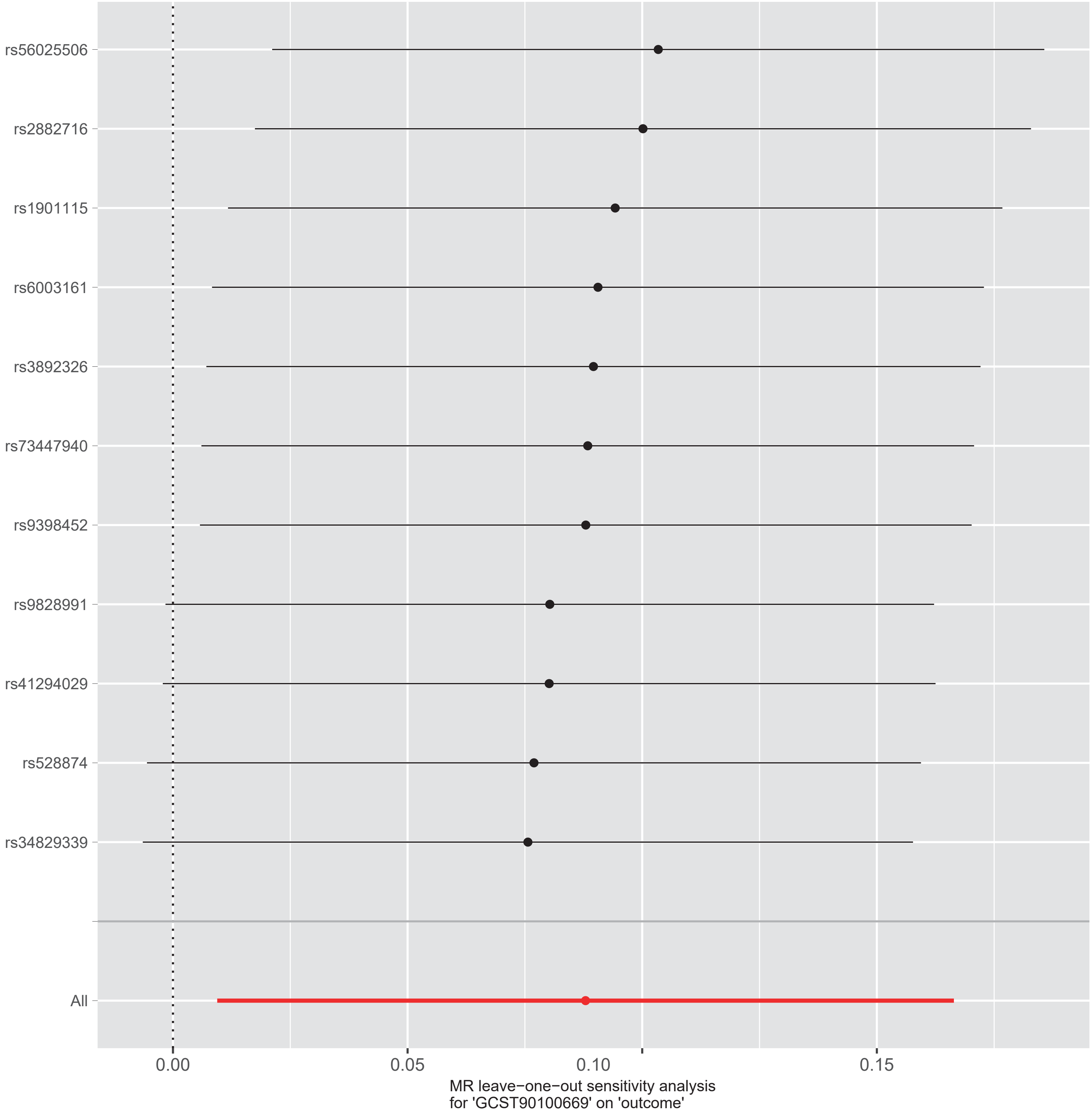
rs56025506
rs2882716
rs1901115
rs6003161
rs3892326
rs73447940
rs9398452
rs9828991
rs41294029
rs528874
rs34829339
All
0.00
0.05
0.10
0.15
MR leave−one−out sensitivity analysis
for 'GCST90100669' on 'outcome'

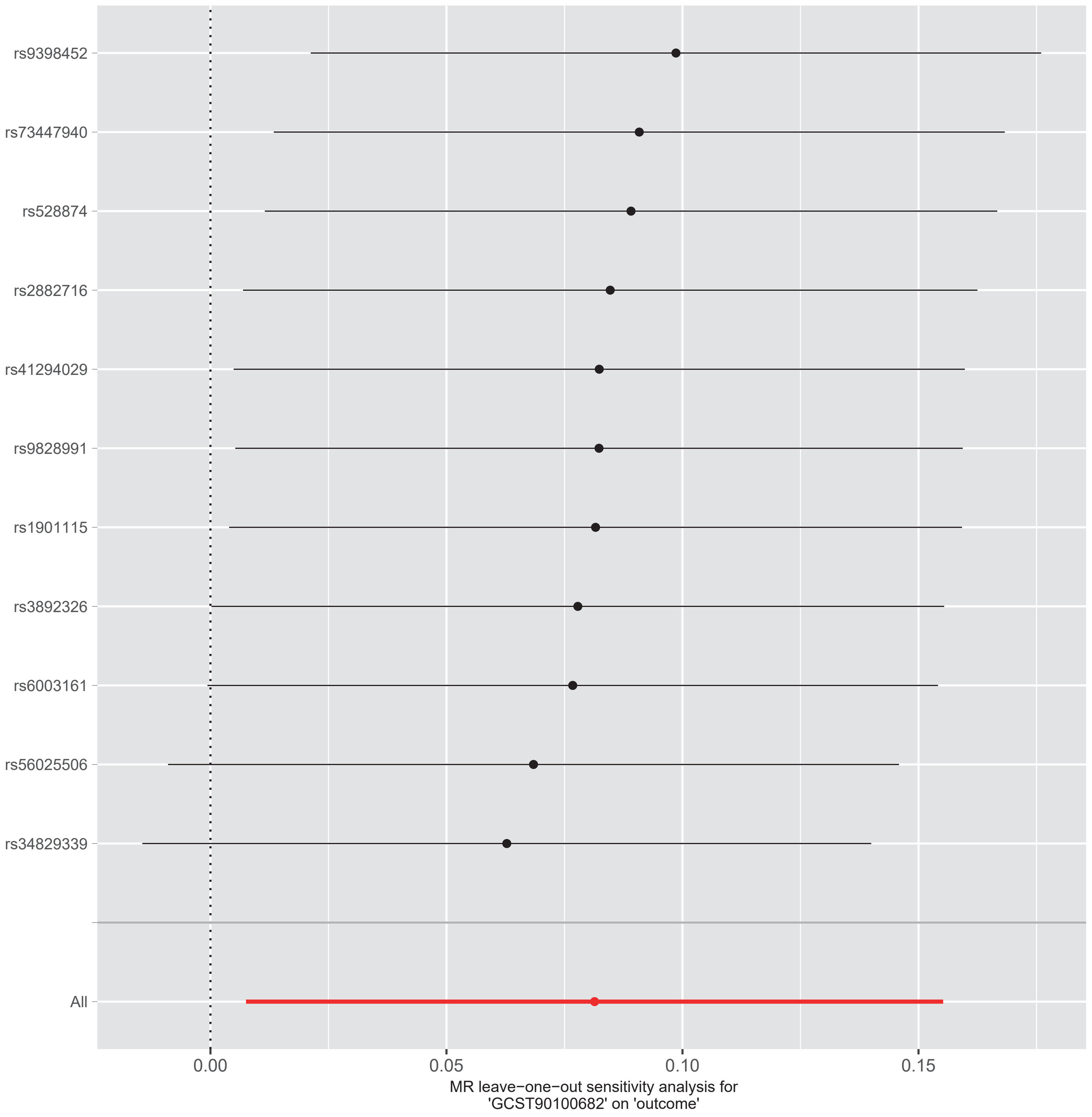
rs9398452
rs73447940
rs528874
rs2882716
rs41294029
rs9828991
rs1901115
rs3892326
rs6003161
rs56025506
rs34829339
All
0.00
0.05
0.10
0.15
MR leave−one−out sensitivity analysis for
'GCST90100682' on 'outcome'

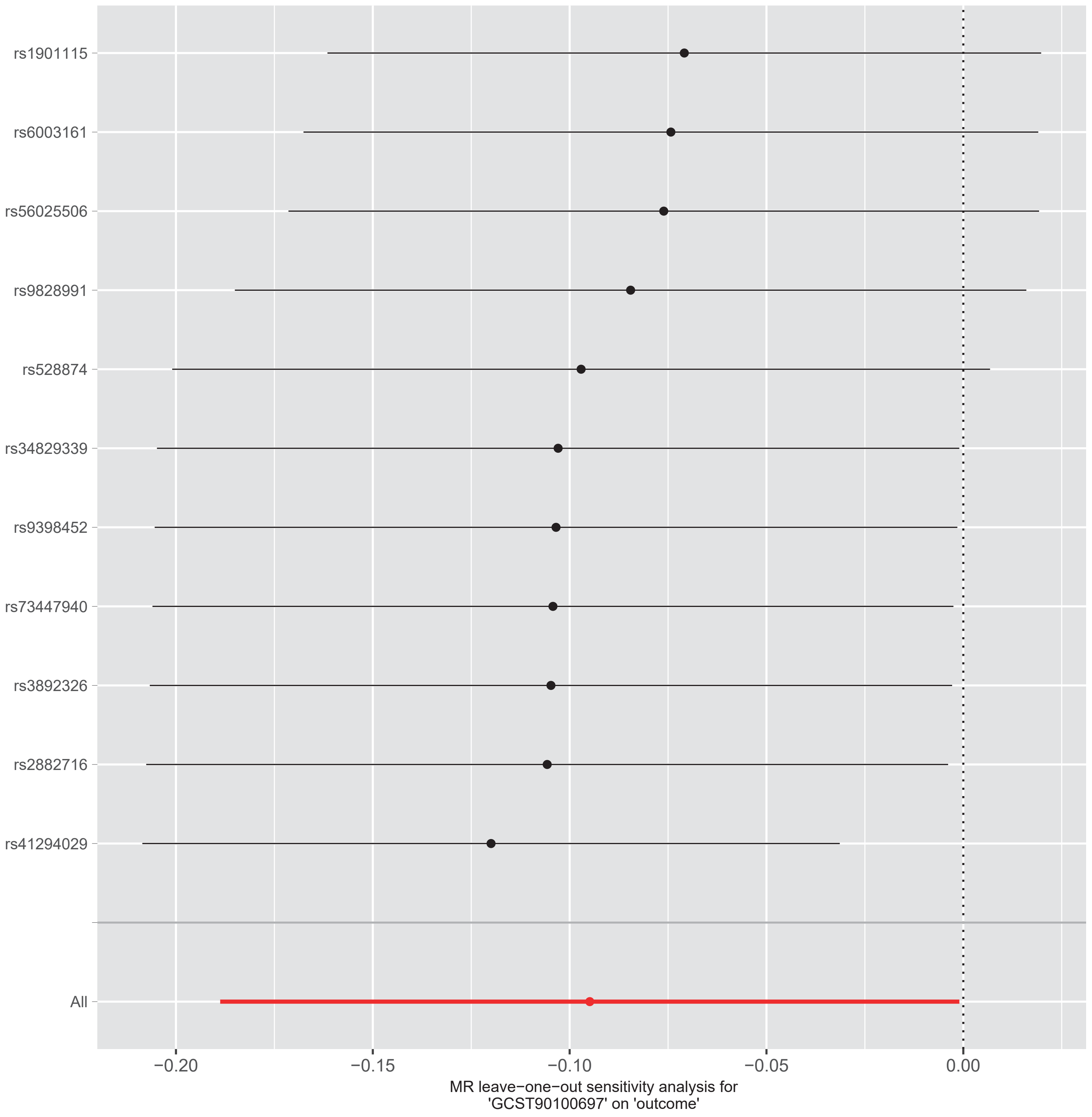
rs1901115
rs6003161
rs56025506
rs9828991
rs528874
rs34829339
rs9398452
rs73447940
rs3892326
rs2882716
rs41294029
All
−0.20
−0.15
−0.10
−0.05
0.00
MR leave−one−out sensitivity analysis for
'GCST90100697' on 'outcome'
