# Supplementary material for: Dissecting Causal Relationships Between Gut Microbiota, Plasma Metabolites and Bladder Cancer: A Two‐Step Mendelian Randomization Study
Source: Health Sci Rep. 2025 Sep 9;8(9):e71206. doi: 10.1002/hsr2.71206 (PMC12420358; doi:10.1002/hsr2.71206)
Supplement: Supplementary file 5 — Figure S5: Funnel plots for MR causal effects of plasma metabolites on s_Bacteroides_dorei. [file HSR2-8-e71206-s002.pdf]

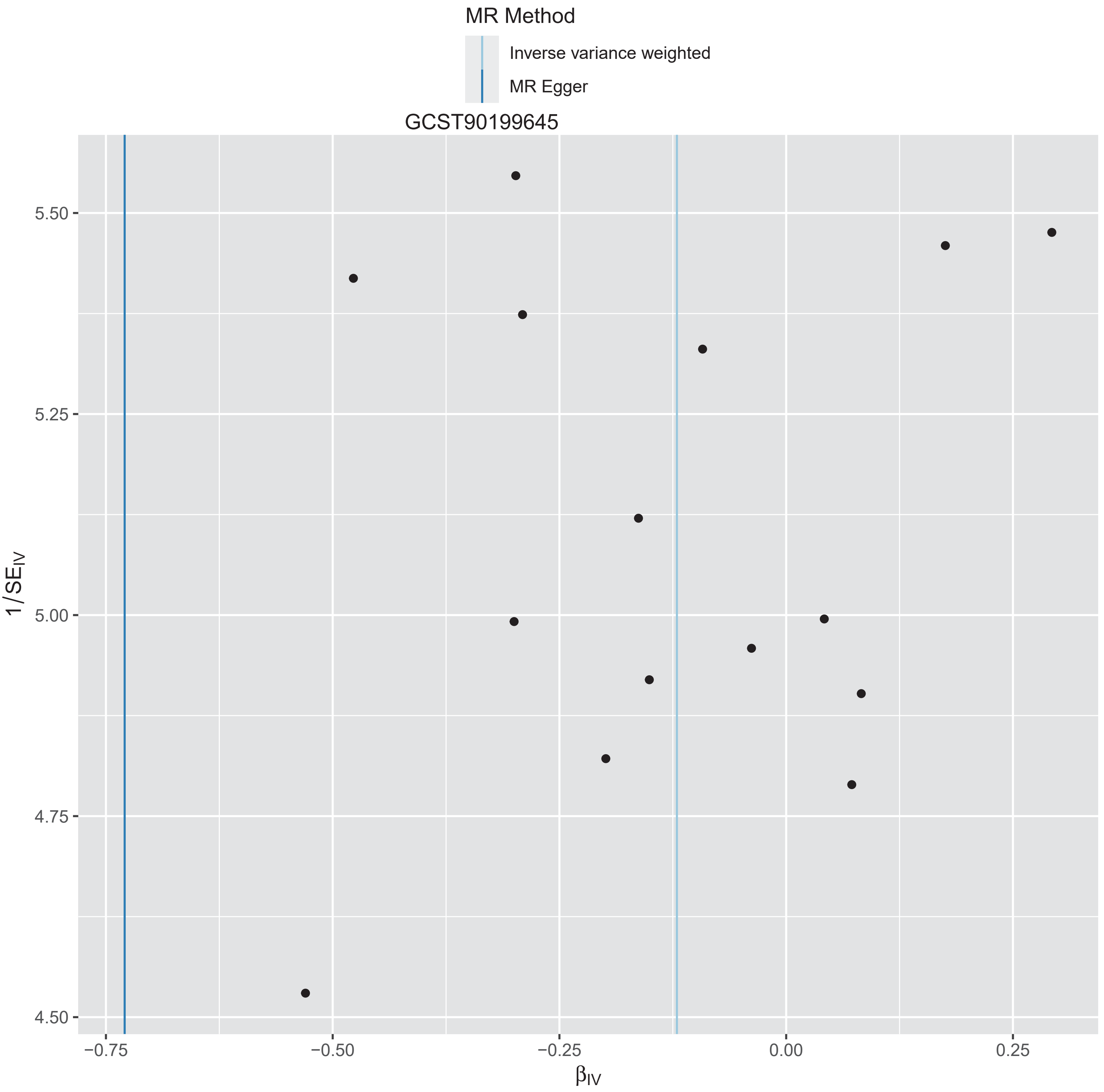
MR Method
Inverse variance weighted
MR Egger
GCST90199645
5.50
5.25
5.00
4.75
4.50
1/SE_IV
−0.75
−0.50
−0.25
0.00
0.25
β_IV

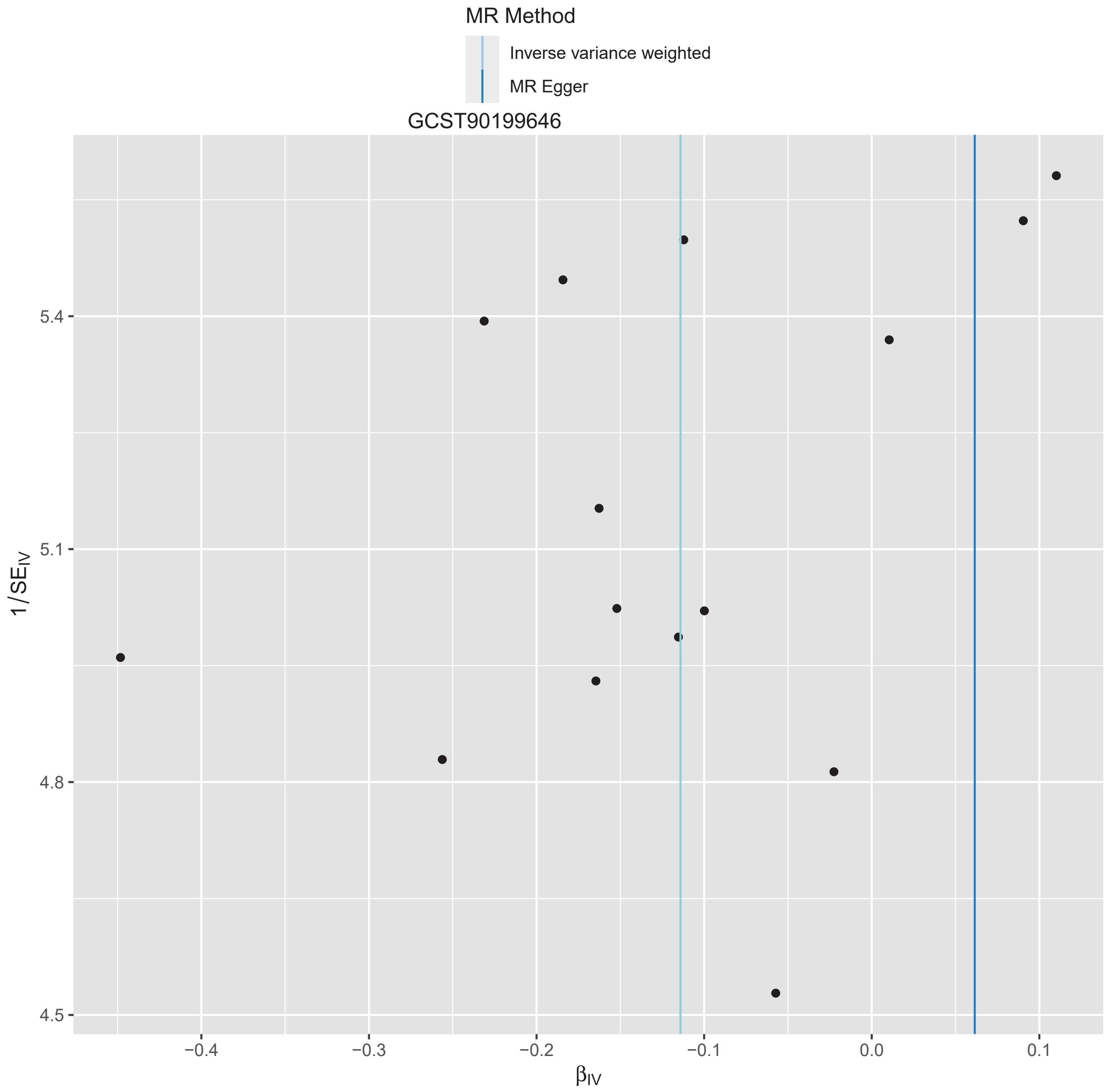
MR Method
Inverse variance weighted
MR Egger
GCST90199646
5.4
5.1
4.8
4.5
1/SE$_{IV}$
−0.4
−0.3
−0.2
−0.1
0.0
0.1
β$_{IV}$

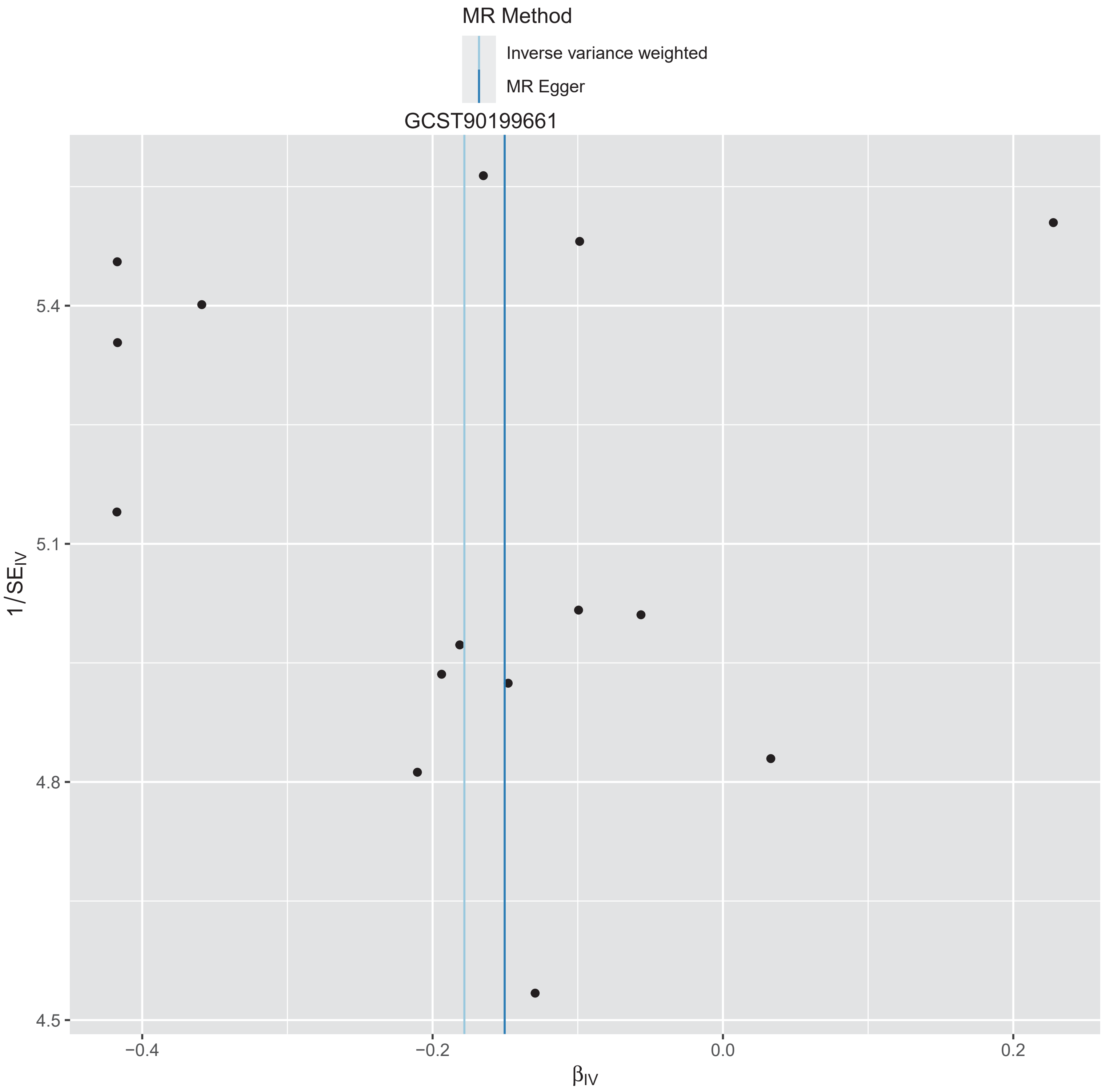
MR Method
Inverse variance weighted
MR Egger
GCST90199661
5.4
5.1
4.8
4.5
1/SE$_{IV}$
−0.4
−0.2
0.0
0.2
β$_{IV}$

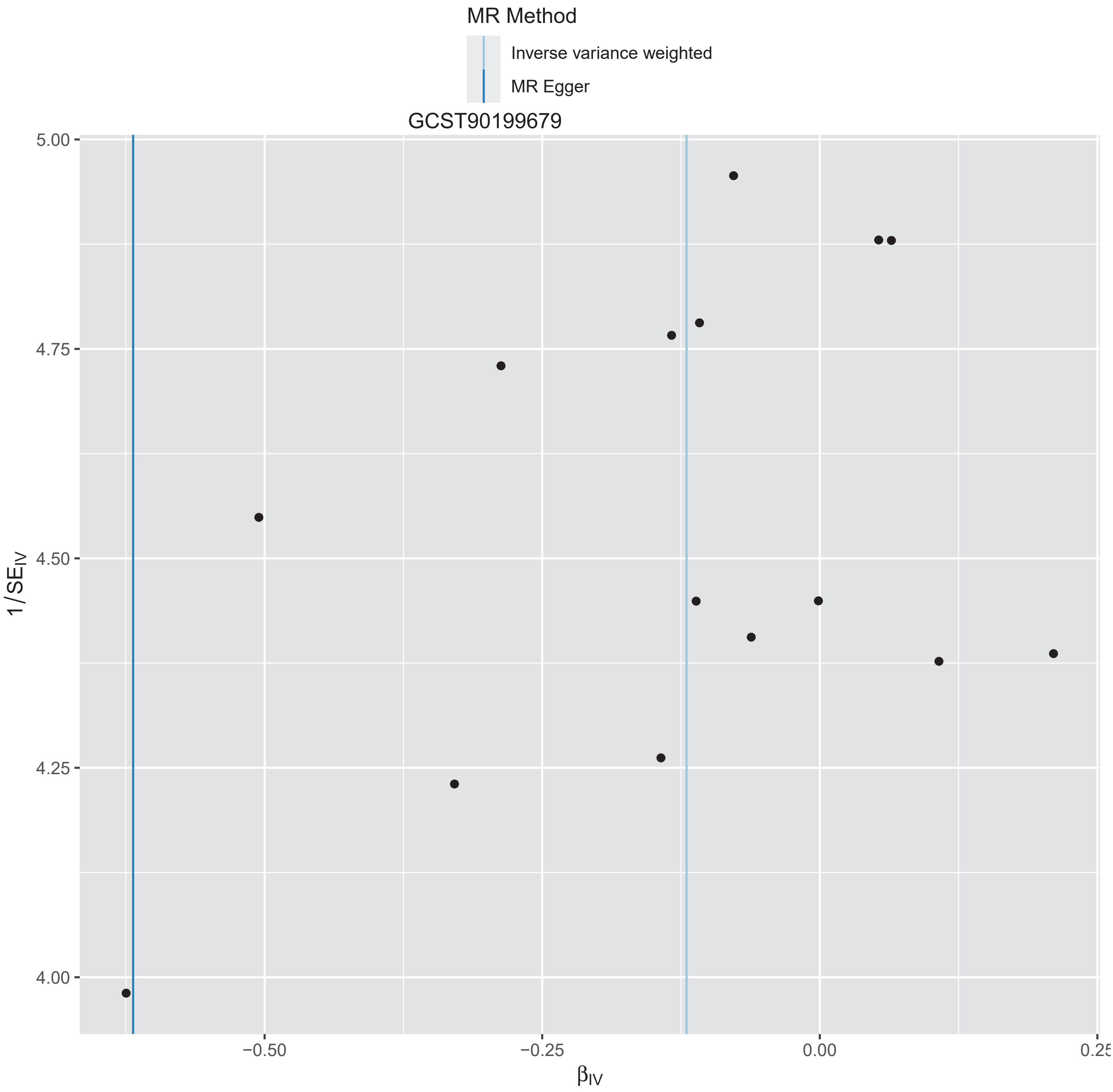

MR Method
Inverse variance weighted
MR Egger
GCST90199679
5.00
4.75
4.50
4.25
4.00
1/SE$_{IV}$
−0.50
−0.25
0.00
0.25
β$_{IV}$

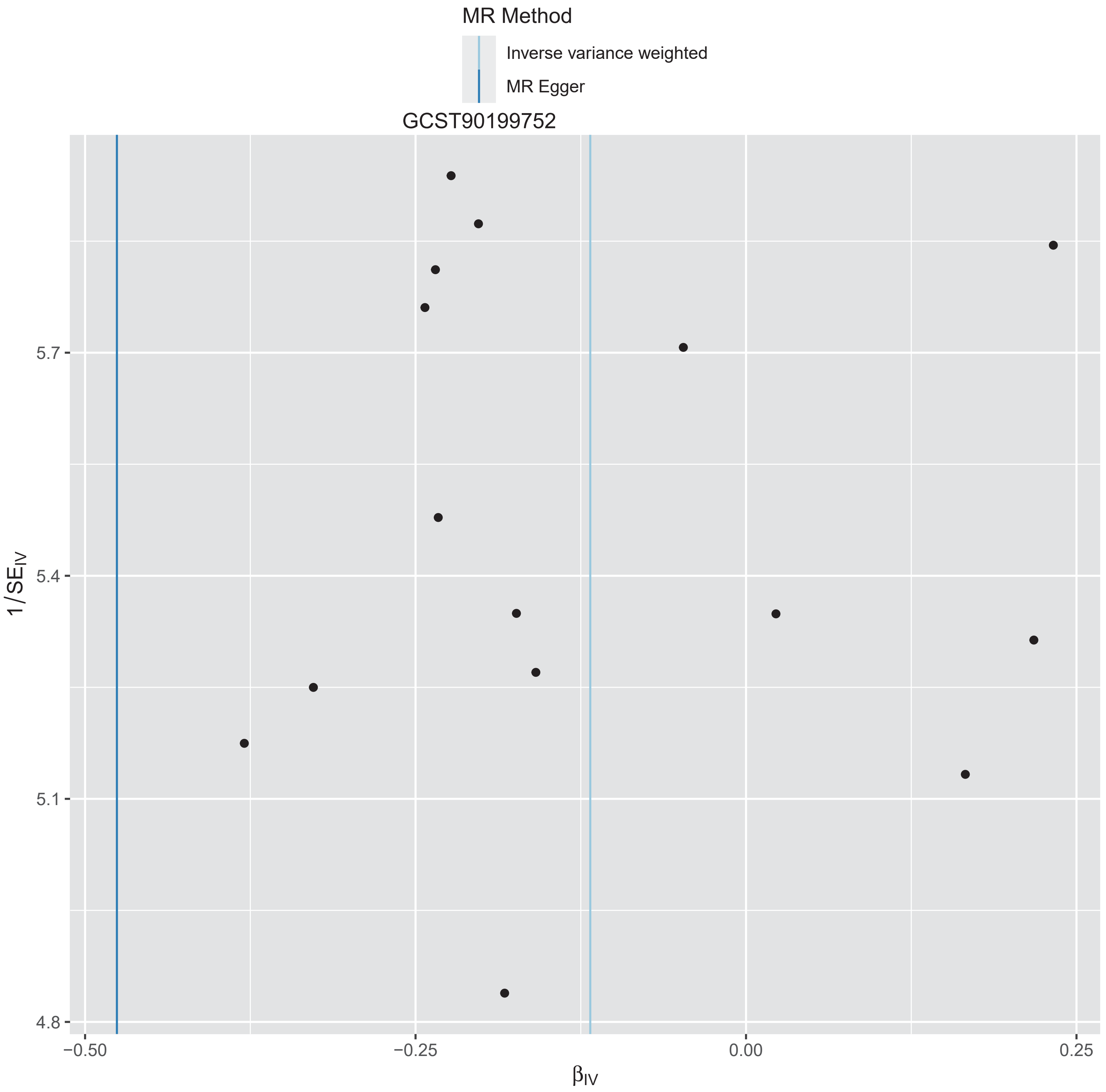

MR Method
Inverse variance weighted
MR Egger
GCST90199752
1/SE_IV
5.7
5.4
5.1
4.8
−0.50
−0.25
0.00
0.25
β_IV

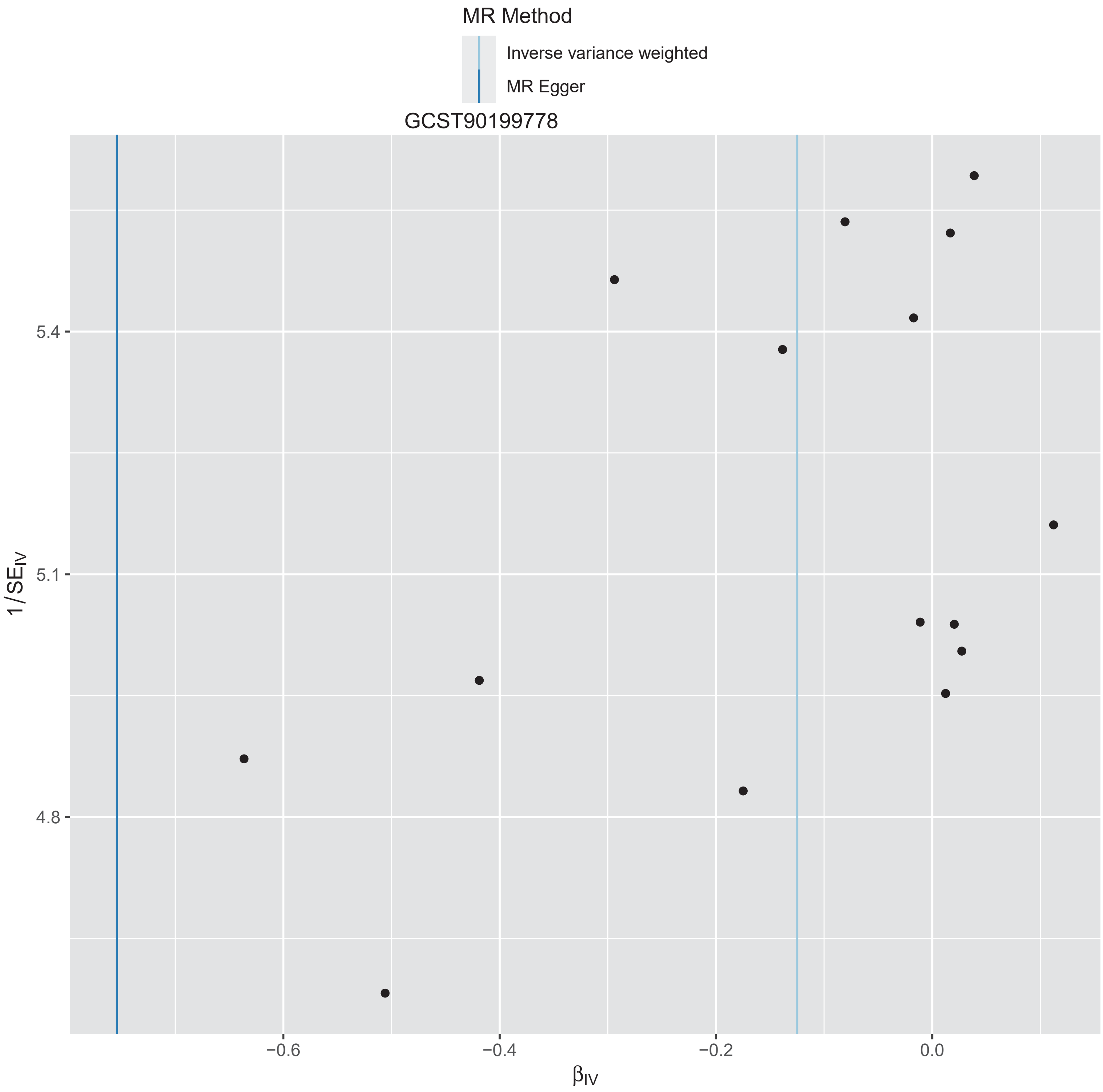

MR Method
Inverse variance weighted
MR Egger
GCST90199778
1/SE$_{IV}$
5.4
5.1
4.8
−0.6
−0.4
−0.2
0.0
β$_{IV}$

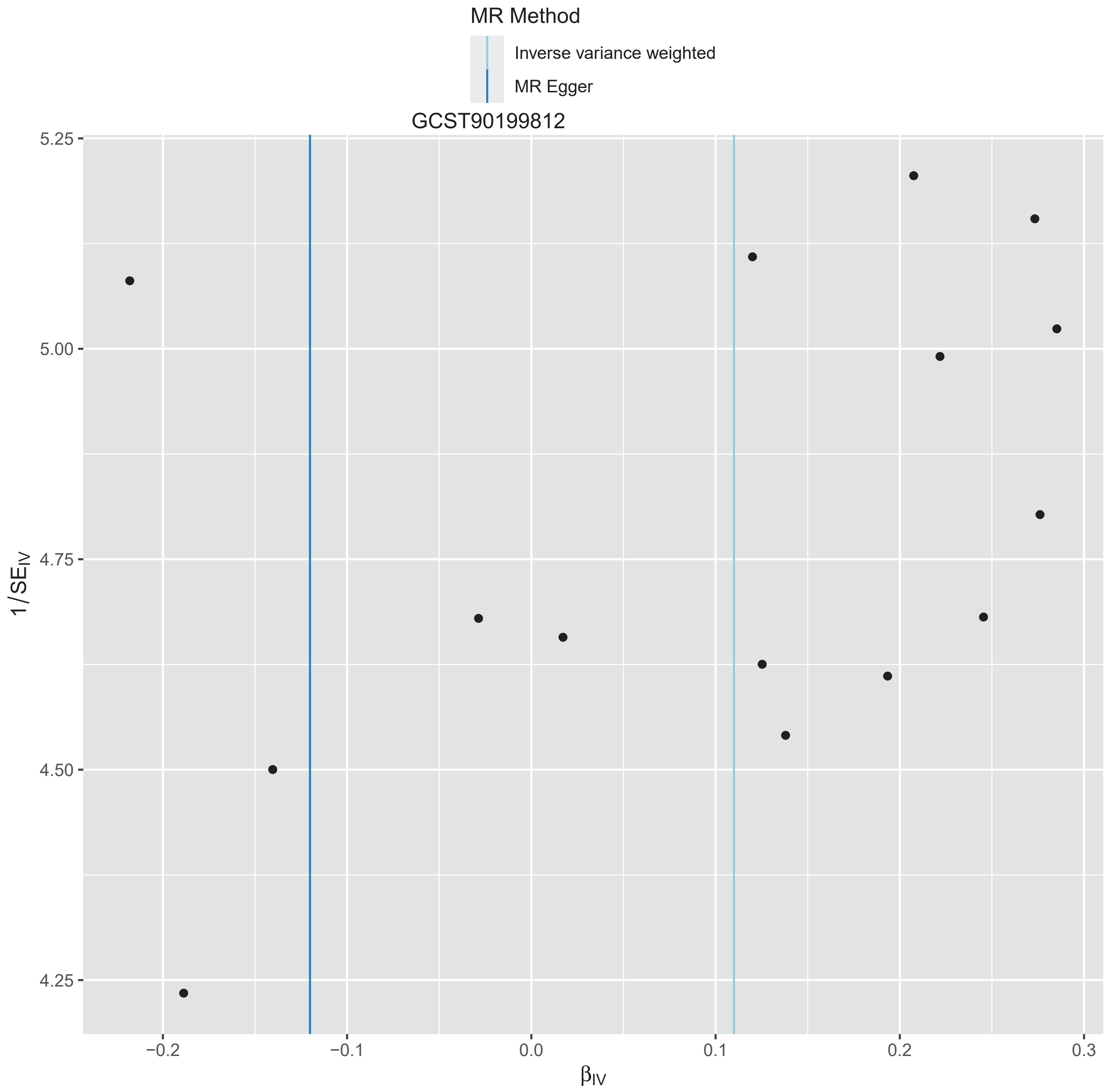

MR Method
Inverse variance weighted
MR Egger
GCST90199812
5.25
5.00
4.75
4.50
4.25
1/SE_IV
−0.2
−0.1
0.0
0.1
0.2
0.3
β_IV

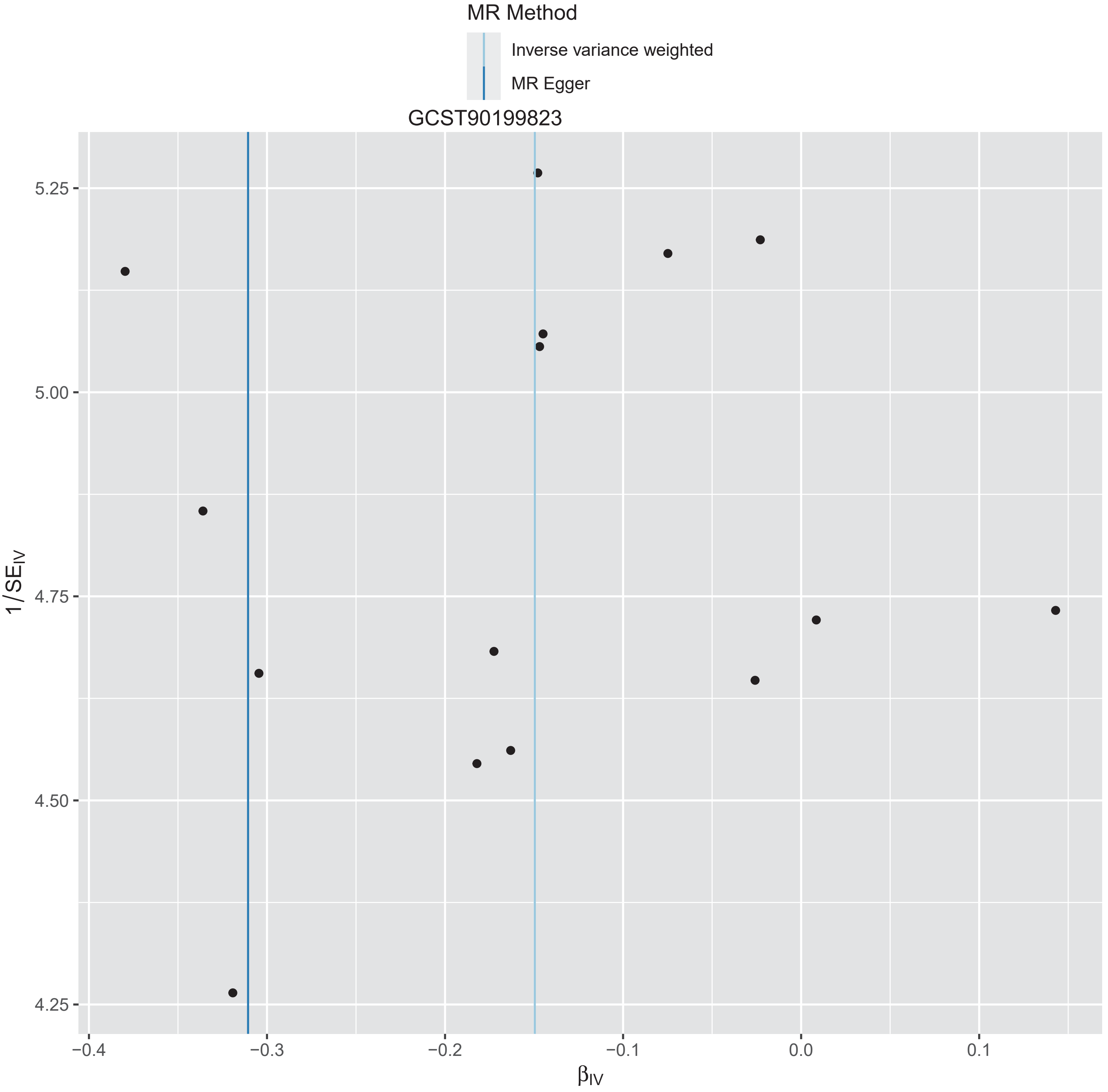
MR Method
Inverse variance weighted
MR Egger
GCST90199823
5.25
5.00
4.75
4.50
4.25
1/SE$_{IV}$
−0.4
−0.3
−0.2
−0.1
0.0
0.1
β$_{IV}$

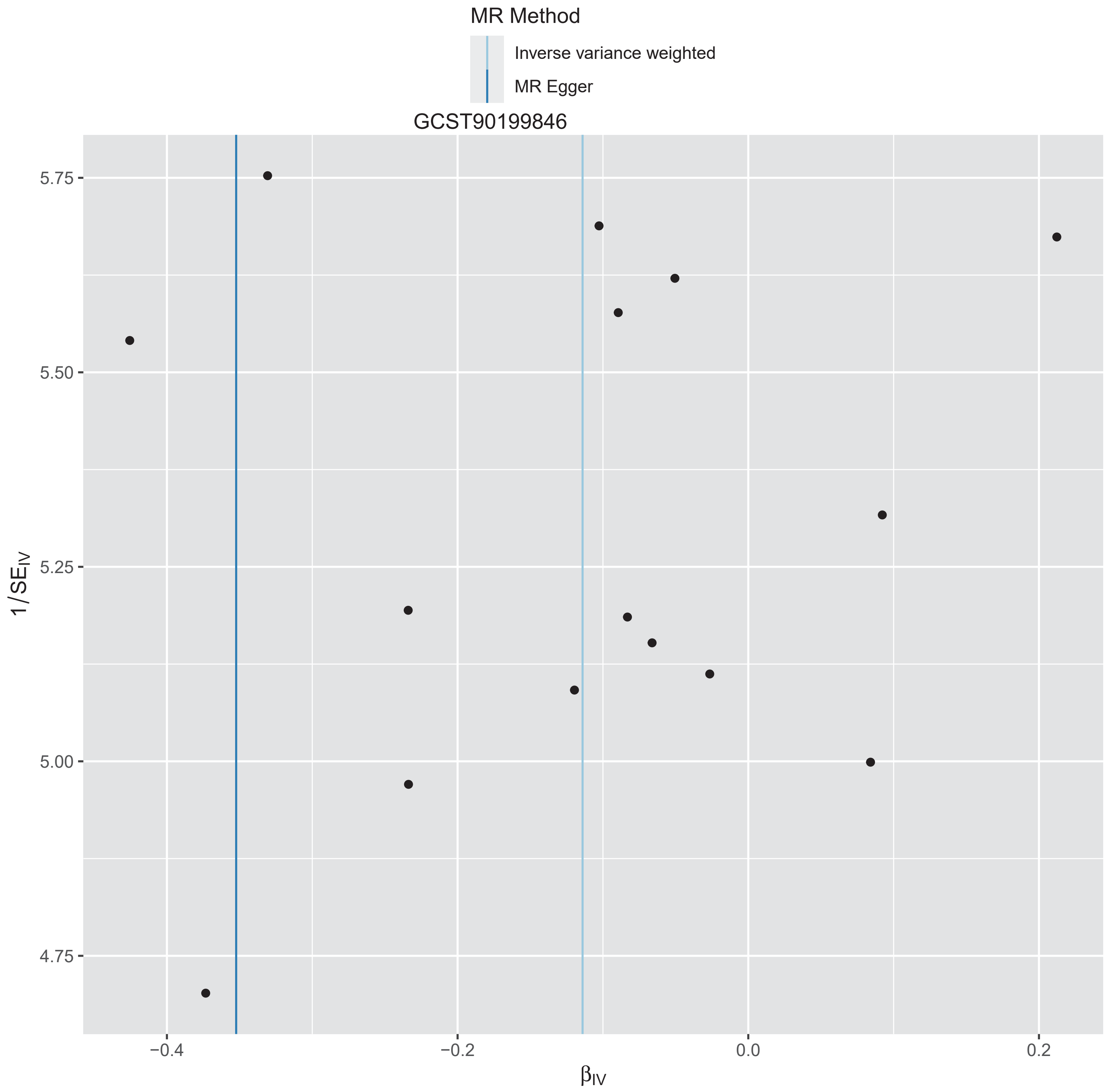
MR Method
Inverse variance weighted
MR Egger
GCST90199846
1/SE_IV
β_IV
5.75
5.50
5.25
5.00
4.75
−0.4
−0.2
0.0
0.2

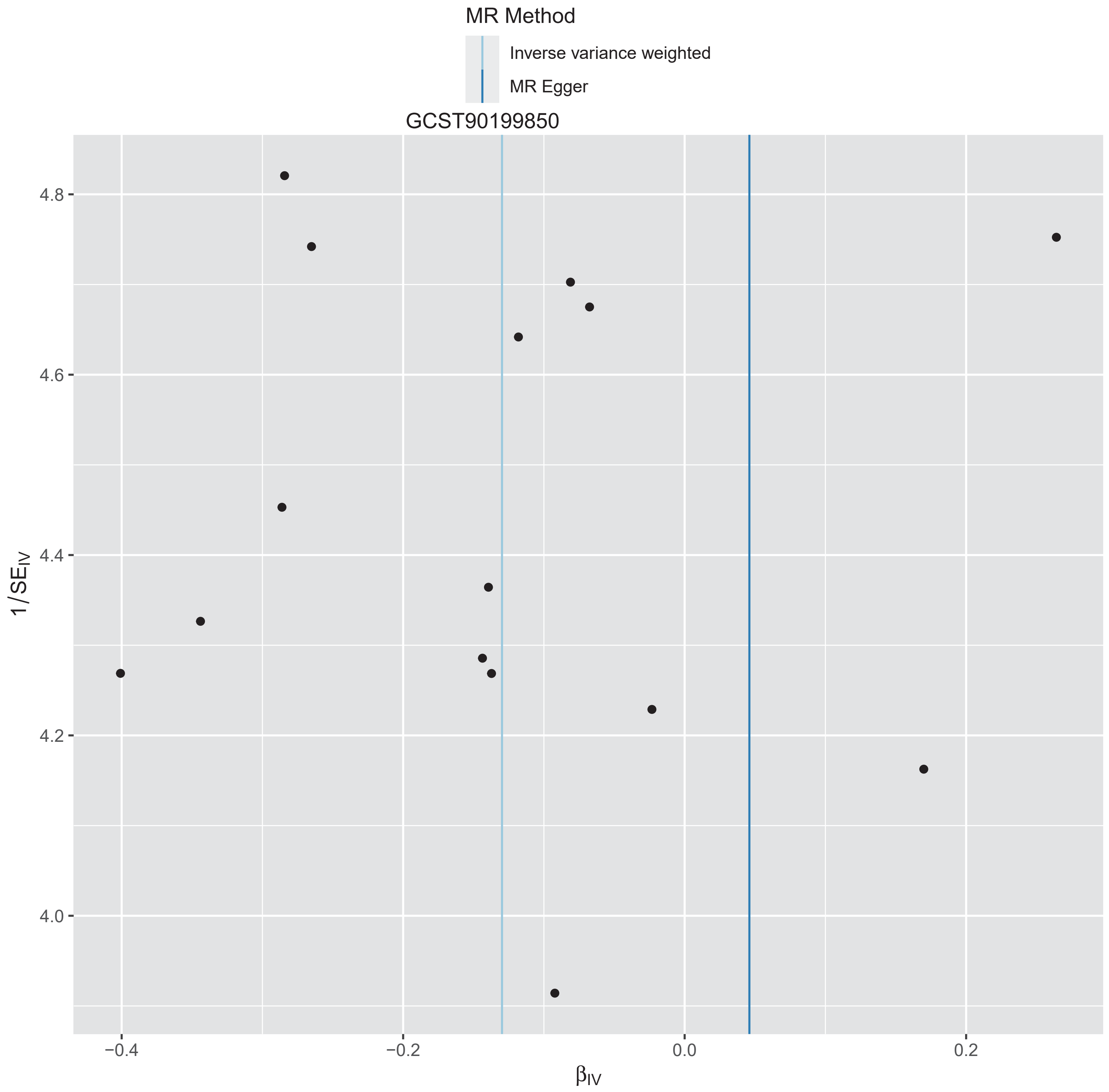

MR Method
Inverse variance weighted
MR Egger
GCST90199850
4.8
4.6
4.4
4.2
4.0
1/SE$_{IV}$
−0.4
−0.2
0.0
0.2
β$_{IV}$

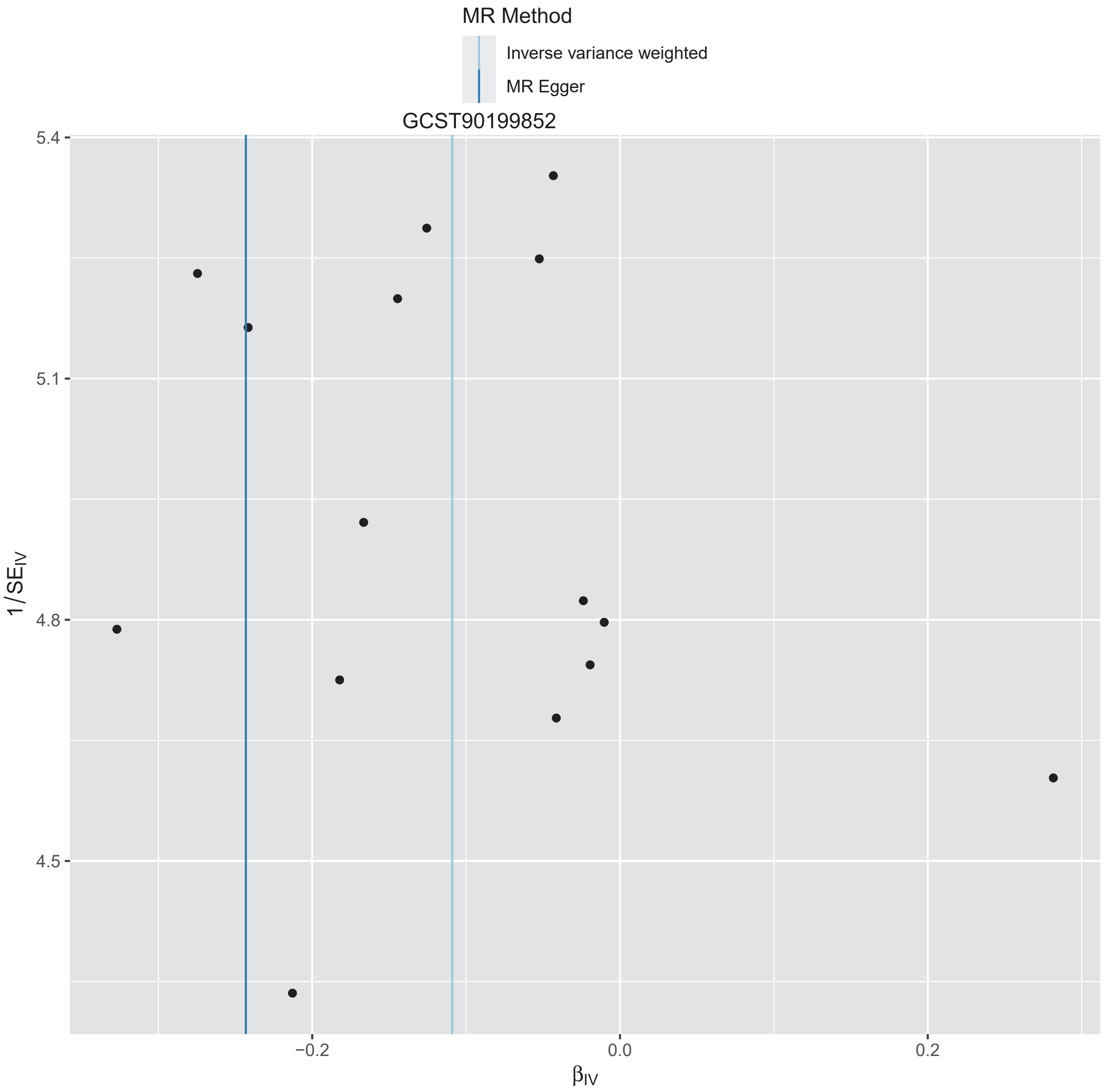

MR Method
Inverse variance weighted
MR Egger
GCST90199852
5.4
5.1
4.8
4.5
1/SE$_{IV}$
−0.2
0.0
0.2
β$_{IV}$

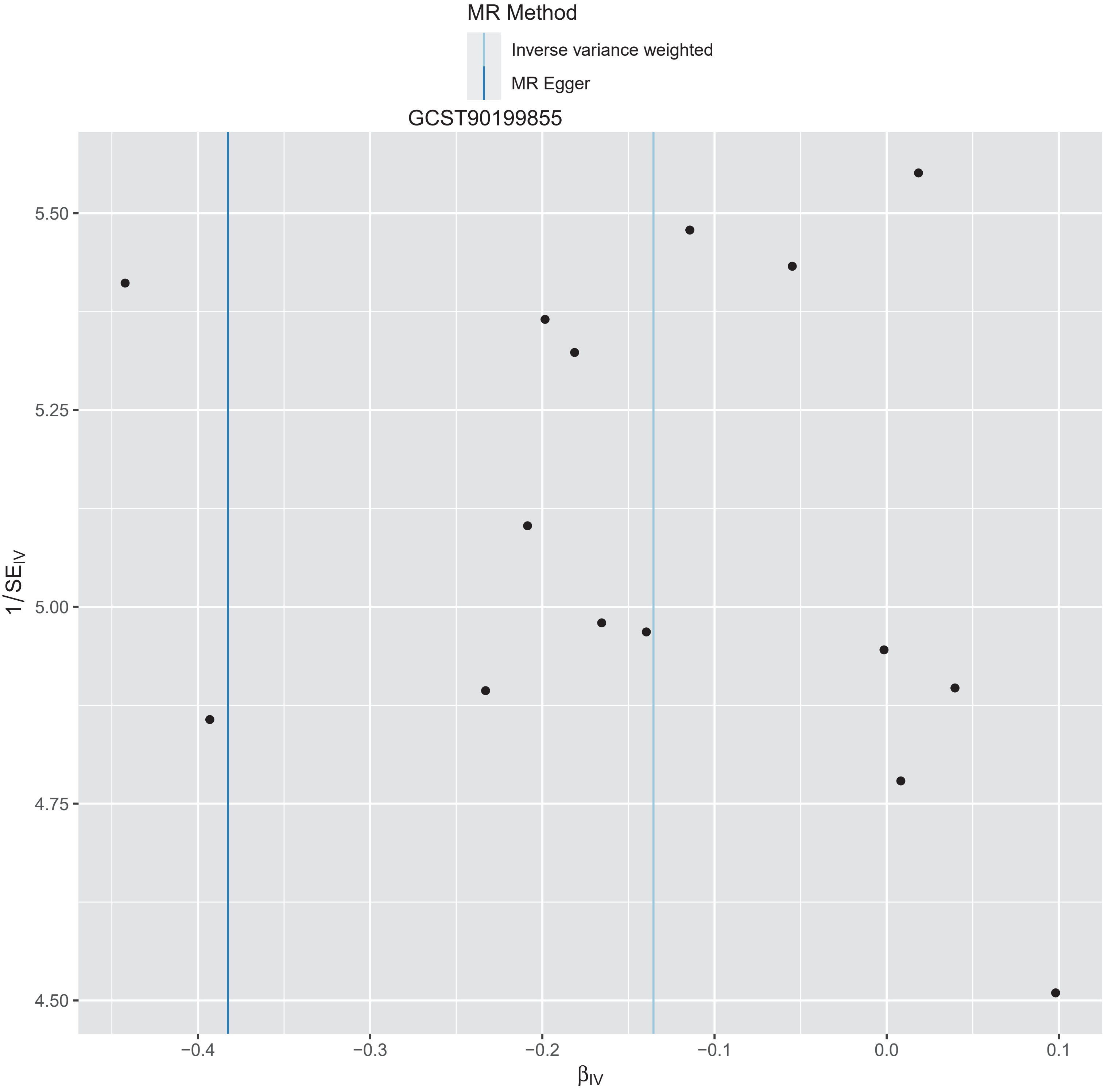

MR Method
Inverse variance weighted
MR Egger
GCST90199855
5.50
5.25
5.00
4.75
4.50
1/SEIV
−0.4
−0.3
−0.2
−0.1
0.0
0.1
βIV

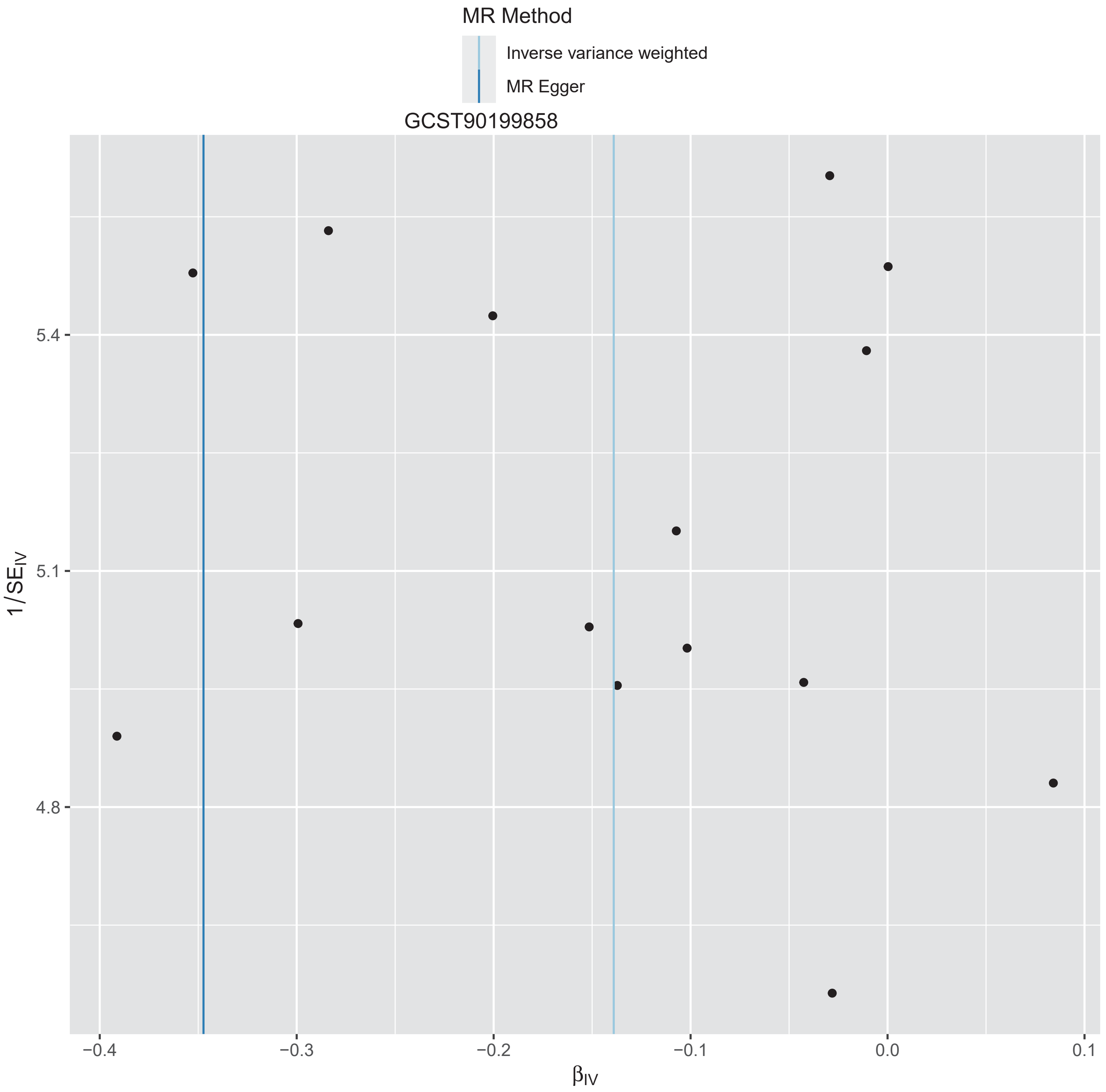
MR Method
Inverse variance weighted
MR Egger
GCST90199858
5.4
5.1
4.8
1/SE_IV
−0.4
−0.3
−0.2
−0.1
0.0
0.1
β_IV

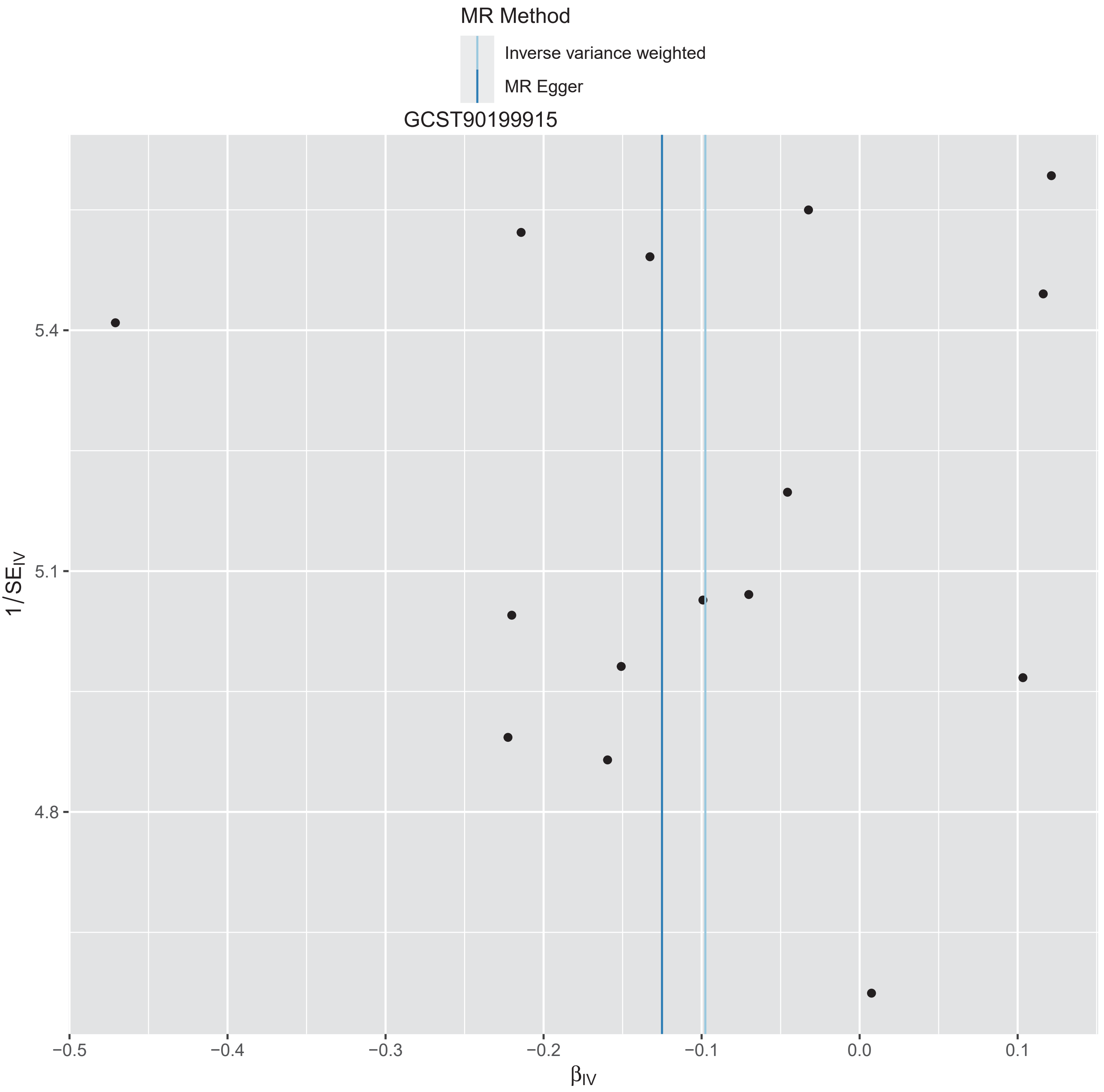
MR Method
Inverse variance weighted
MR Egger
GCST90199915
5.4
5.1
4.8
1/SE_IV
−0.5
−0.4
−0.3
−0.2
−0.1
0.0
0.1
β_IV

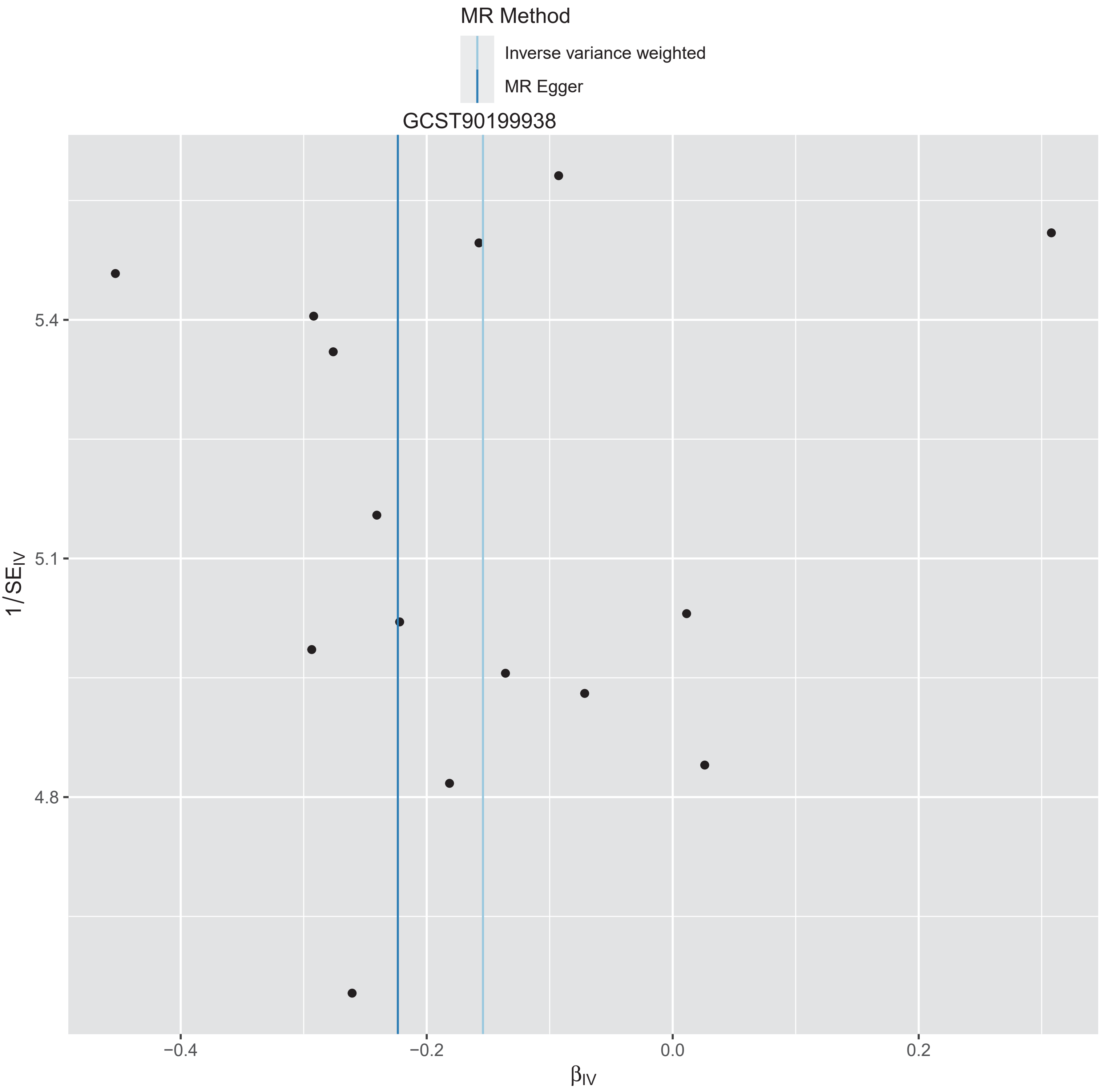
MR Method
Inverse variance weighted
MR Egger
GCST90199938
5.4
5.1
4.8
1/SE$_{IV}$
−0.4
−0.2
0.0
0.2
β$_{IV}$

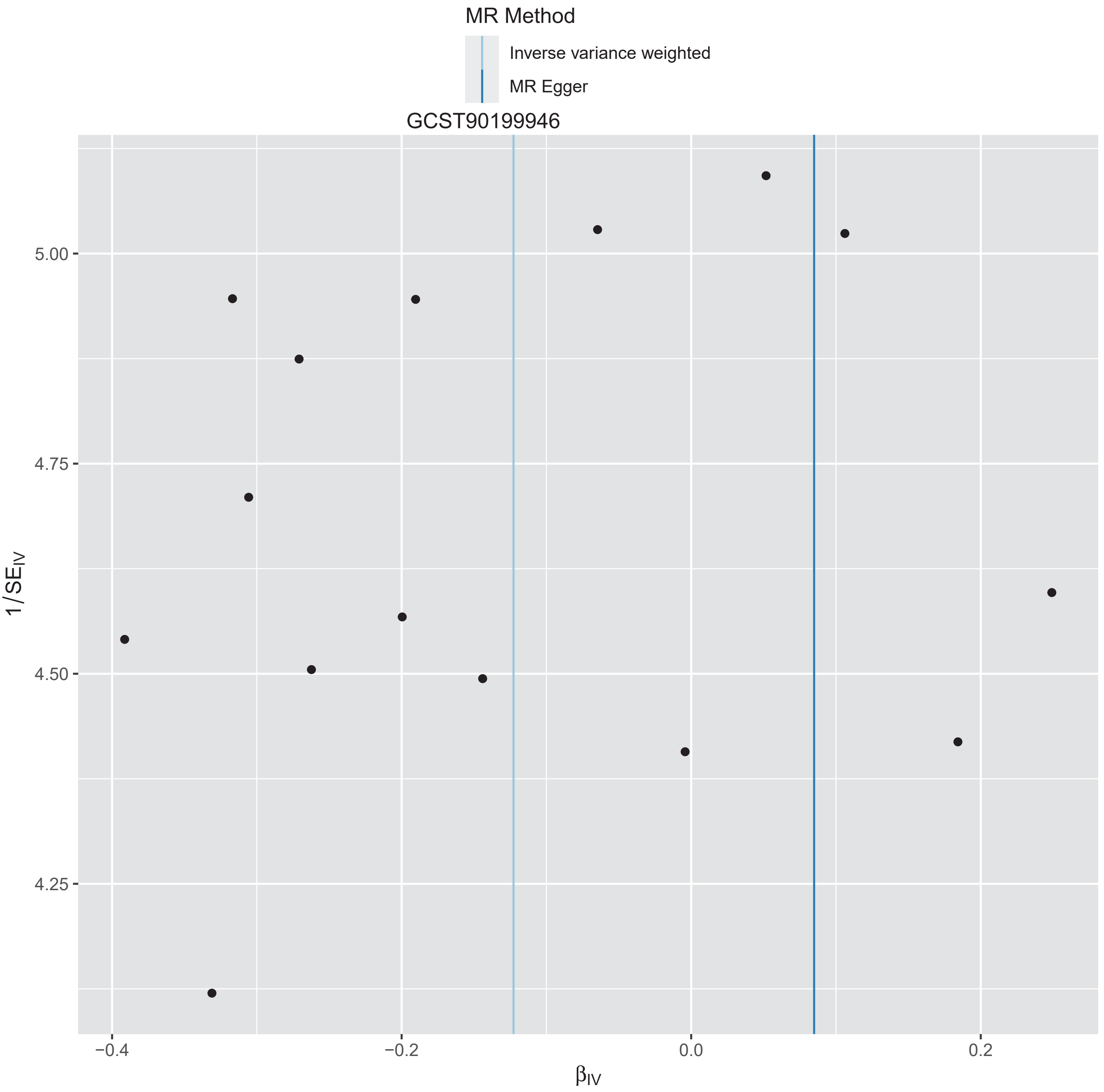
MR Method
Inverse variance weighted
MR Egger
GCST90199946
5.00
4.75
4.50
4.25
1/SE_IV
−0.4
−0.2
0.0
0.2
β_IV

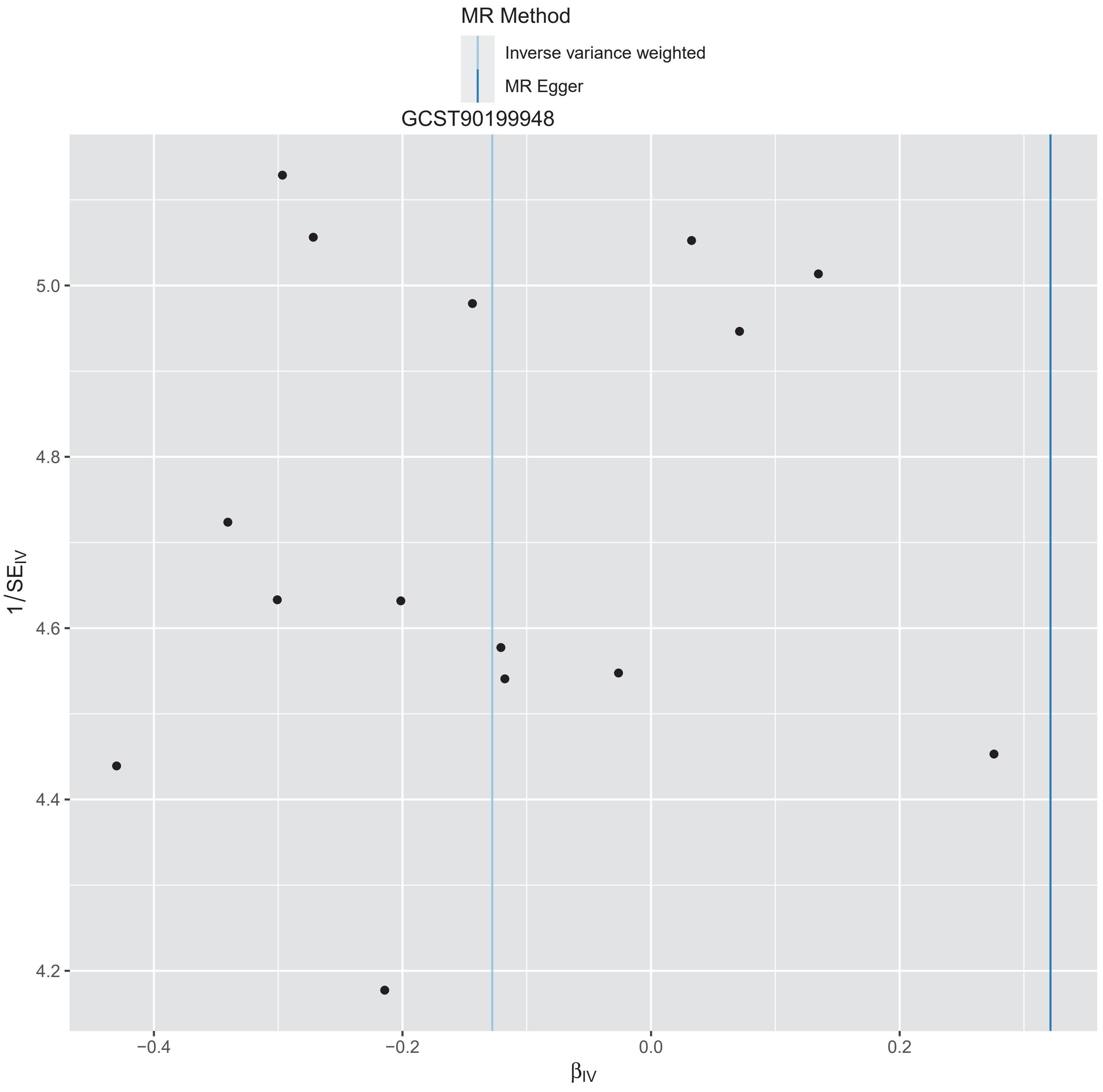
MR Method
Inverse variance weighted
MR Egger
GCST90199948
5.0
4.8
4.6
4.4
4.2
1/SE_IV
−0.4
−0.2
0.0
0.2
β_IV

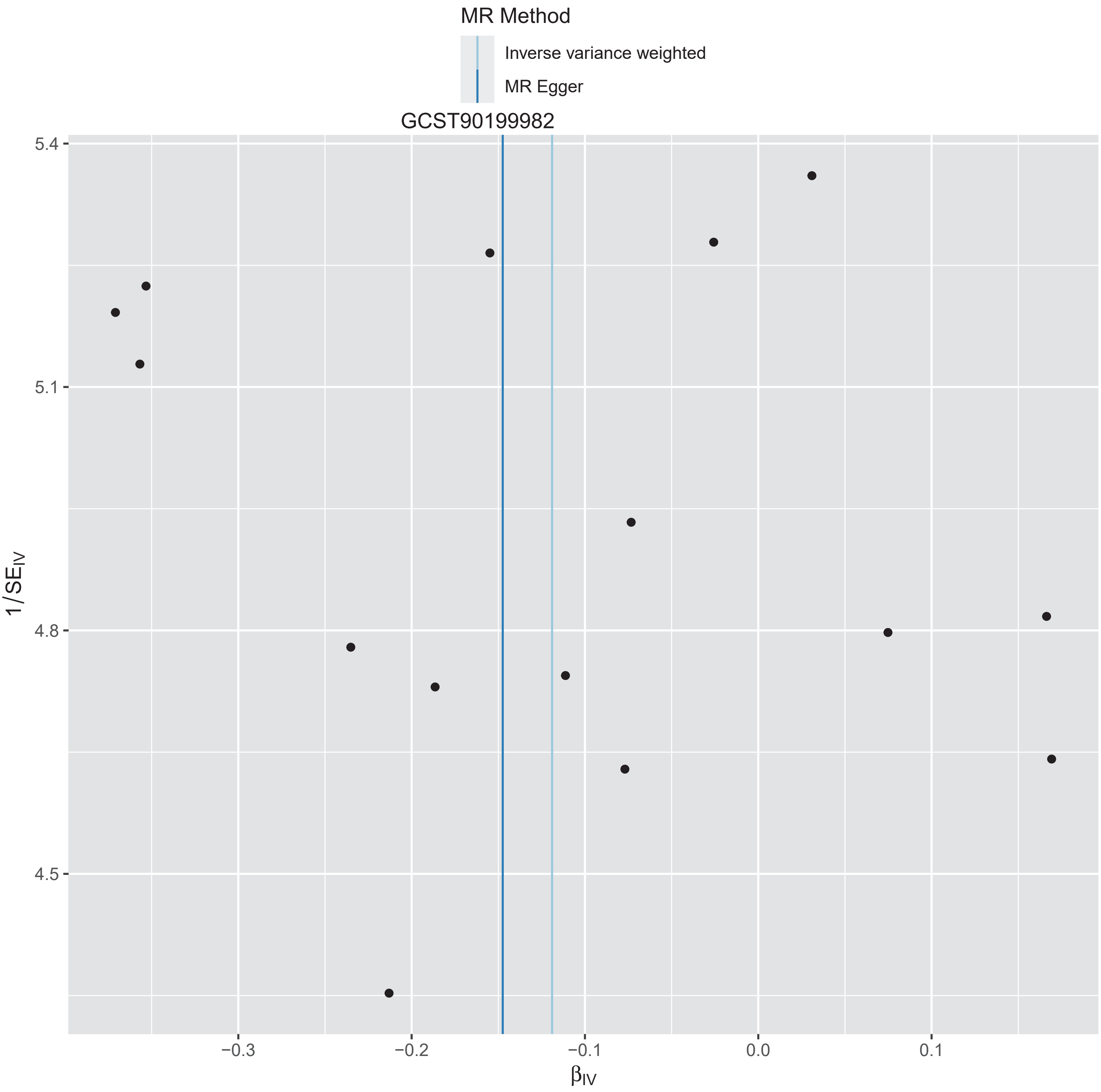

MR Method
Inverse variance weighted
MR Egger
GCST90199982
5.4
5.1
4.8
4.5
1/SE_IV
−0.3
−0.2
−0.1
0.0
0.1
β_IV

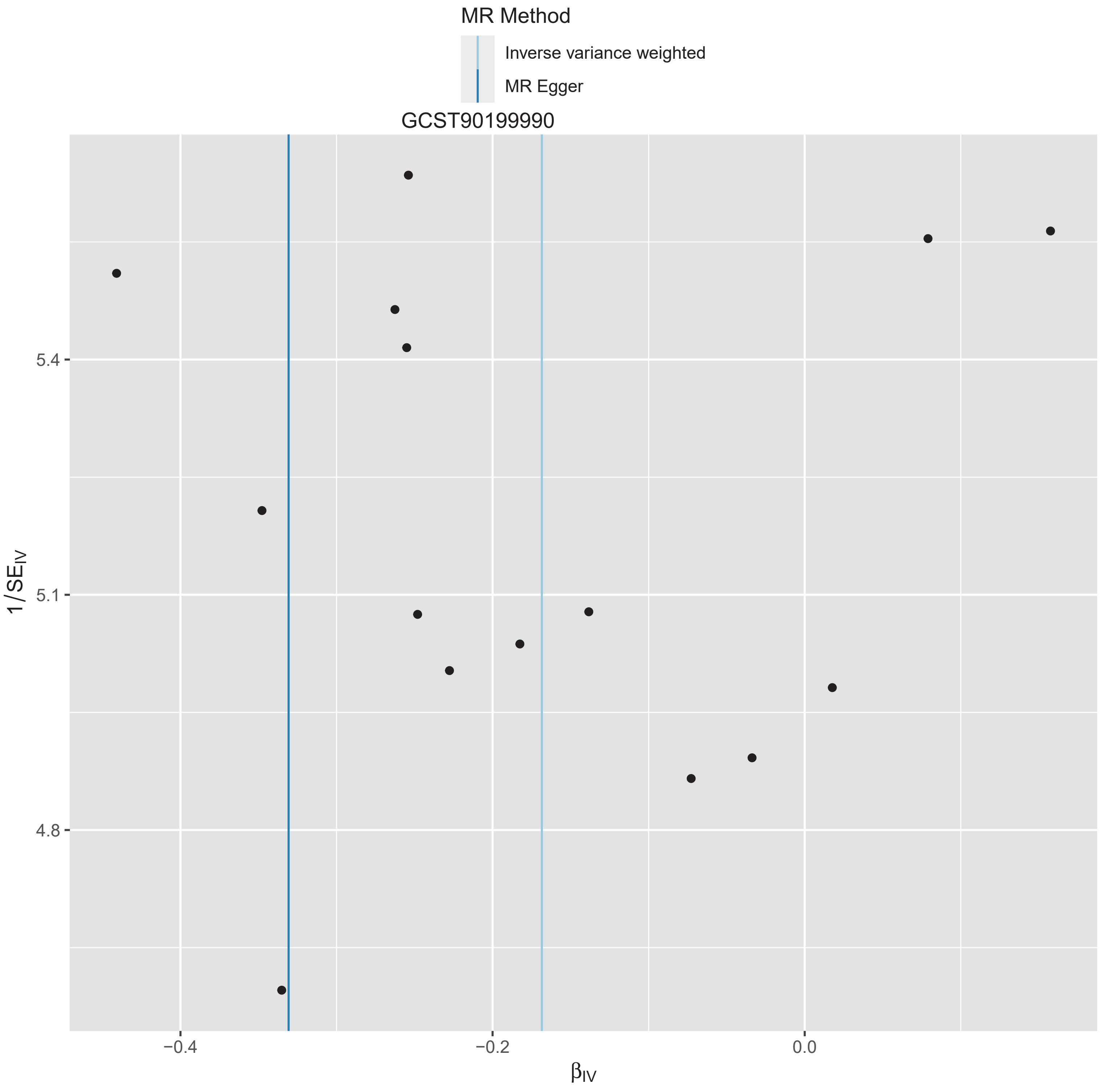
MR Method
Inverse variance weighted
MR Egger
GCST90199990
1/SE$_{IV}$
5.4
5.1
4.8
−0.4
−0.2
0.0
β$_{IV}$

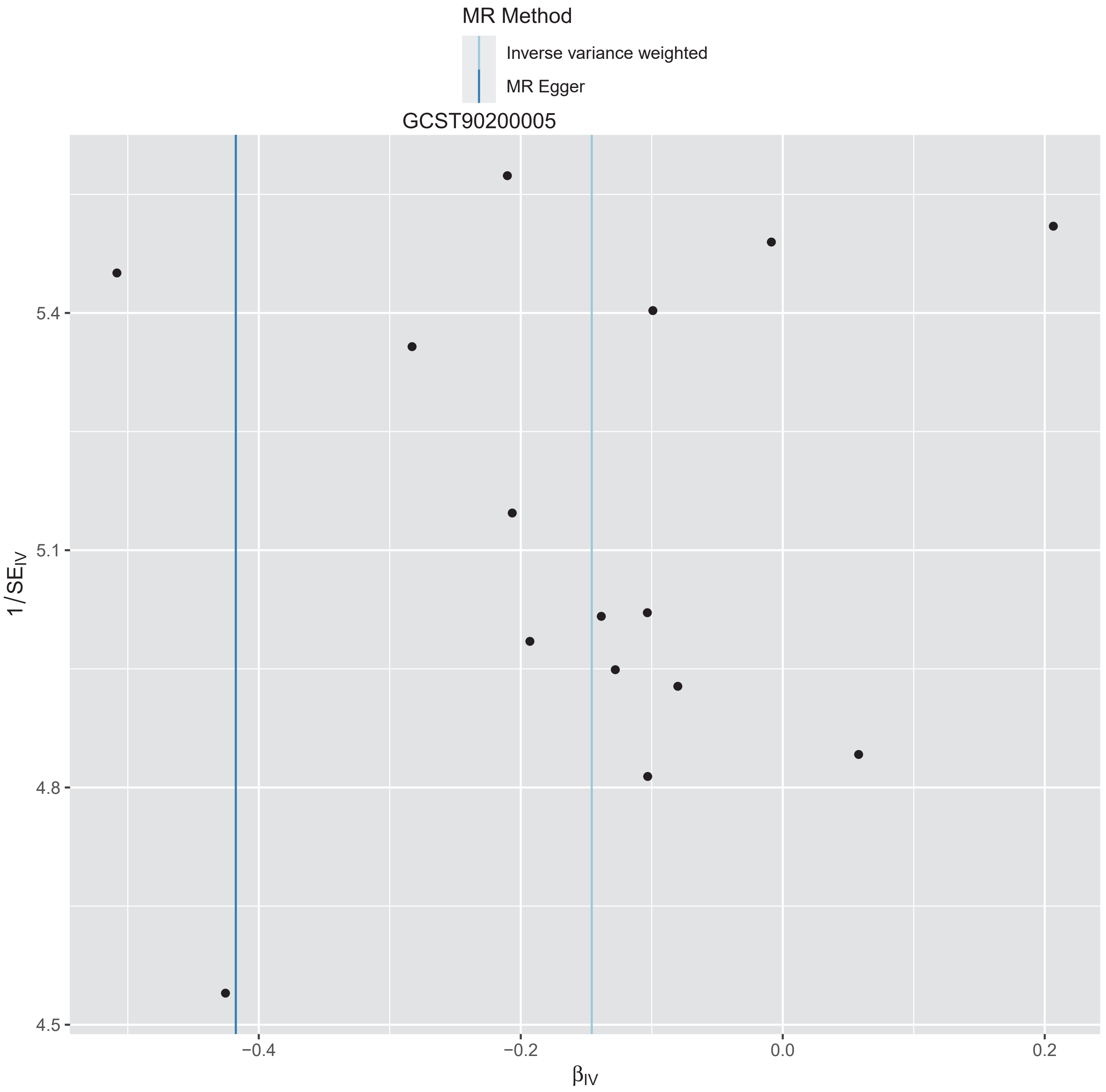
MR Method
Inverse variance weighted
MR Egger
GCST90200005
1/SE_IV
5.4
5.1
4.8
4.5
−0.4
−0.2
0.0
0.2
β_IV

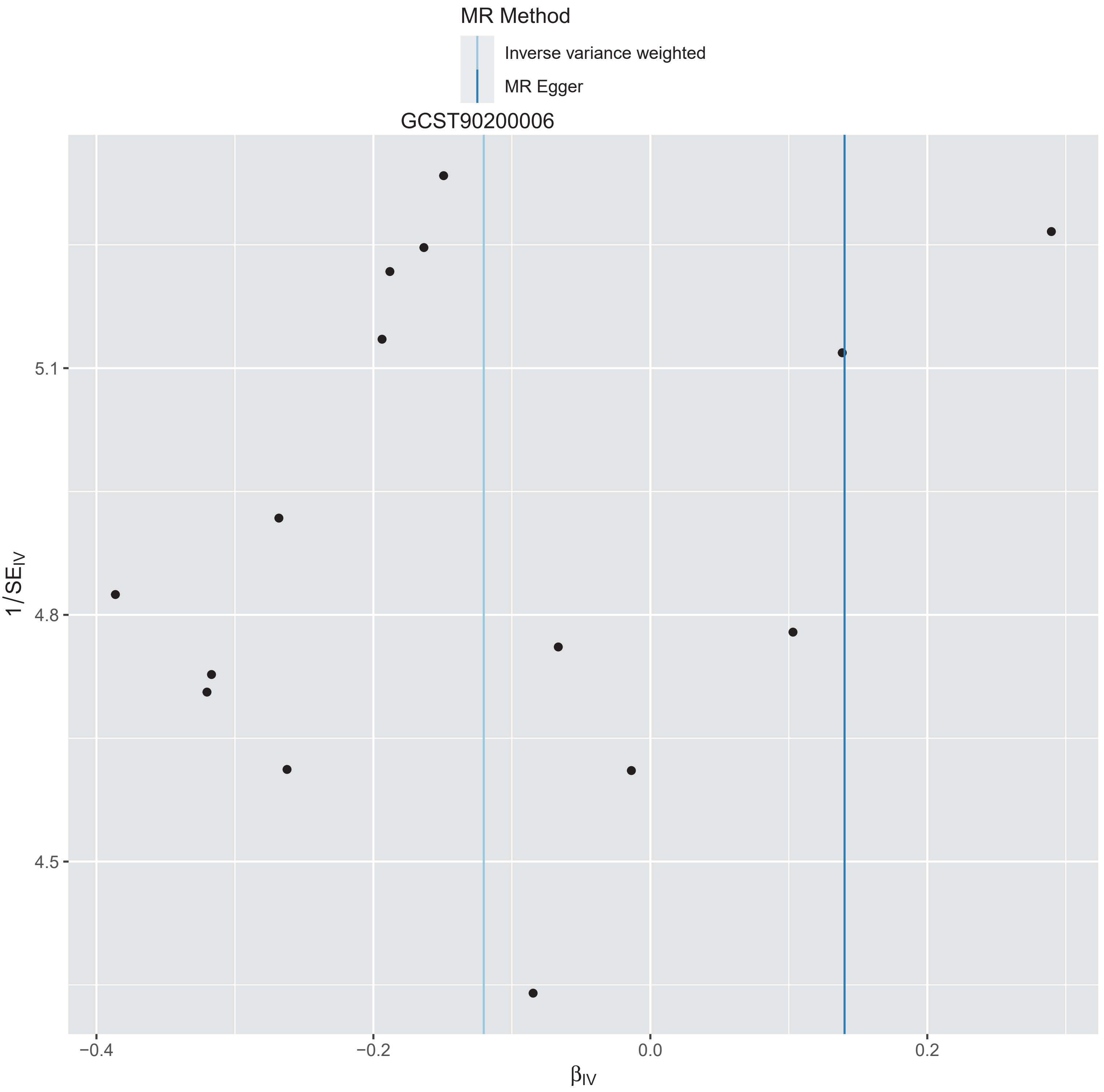

MR Method
Inverse variance weighted
MR Egger
GCST90200006
5.1
4.8
4.5
1/SE$_{IV}$
−0.4
−0.2
0.0
0.2
β$_{IV}$

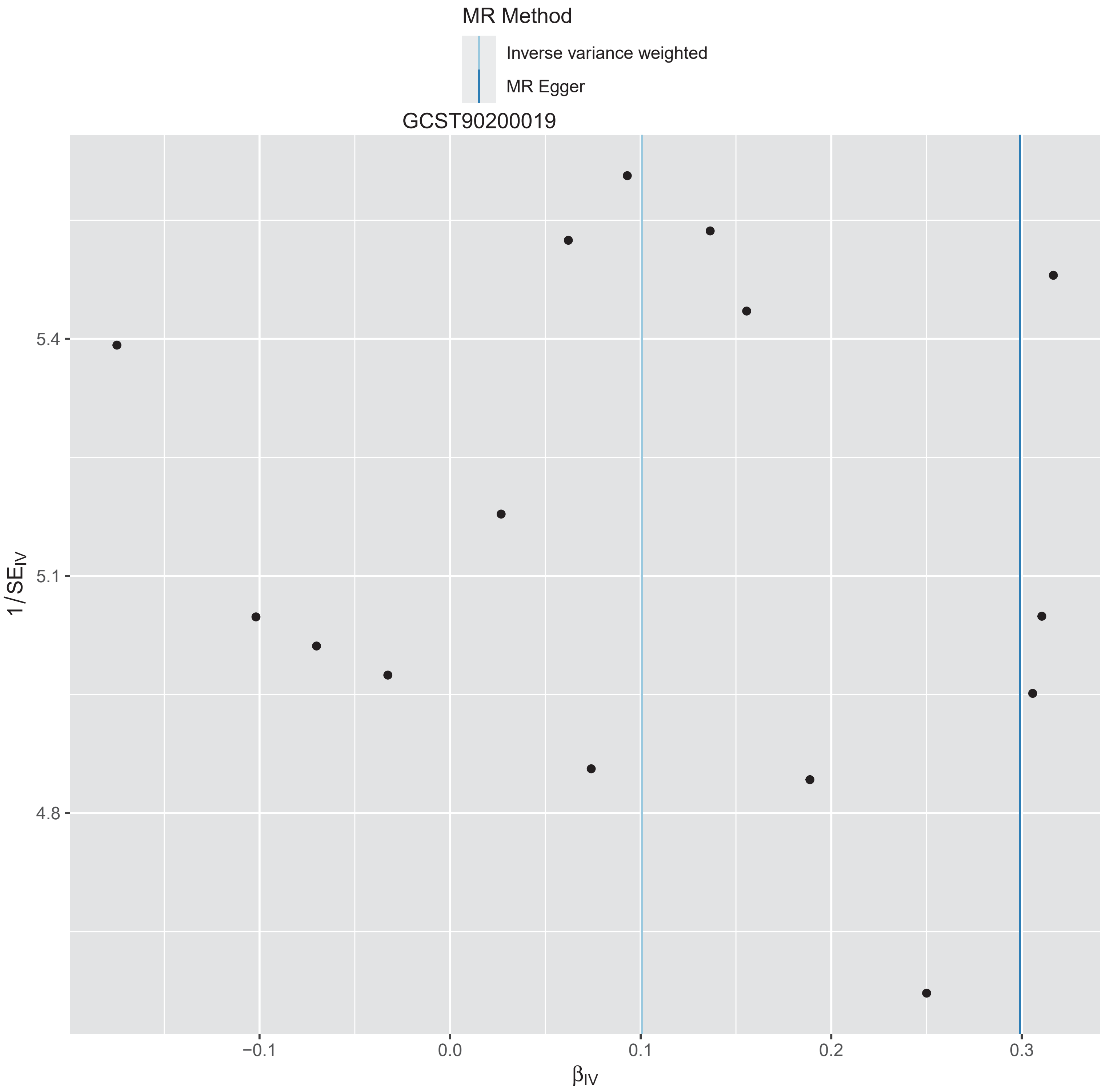
MR Method
Inverse variance weighted
MR Egger
GCST90200019
5.4
5.1
4.8
1/SE_IV
−0.1
0.0
0.1
0.2
0.3
β_IV

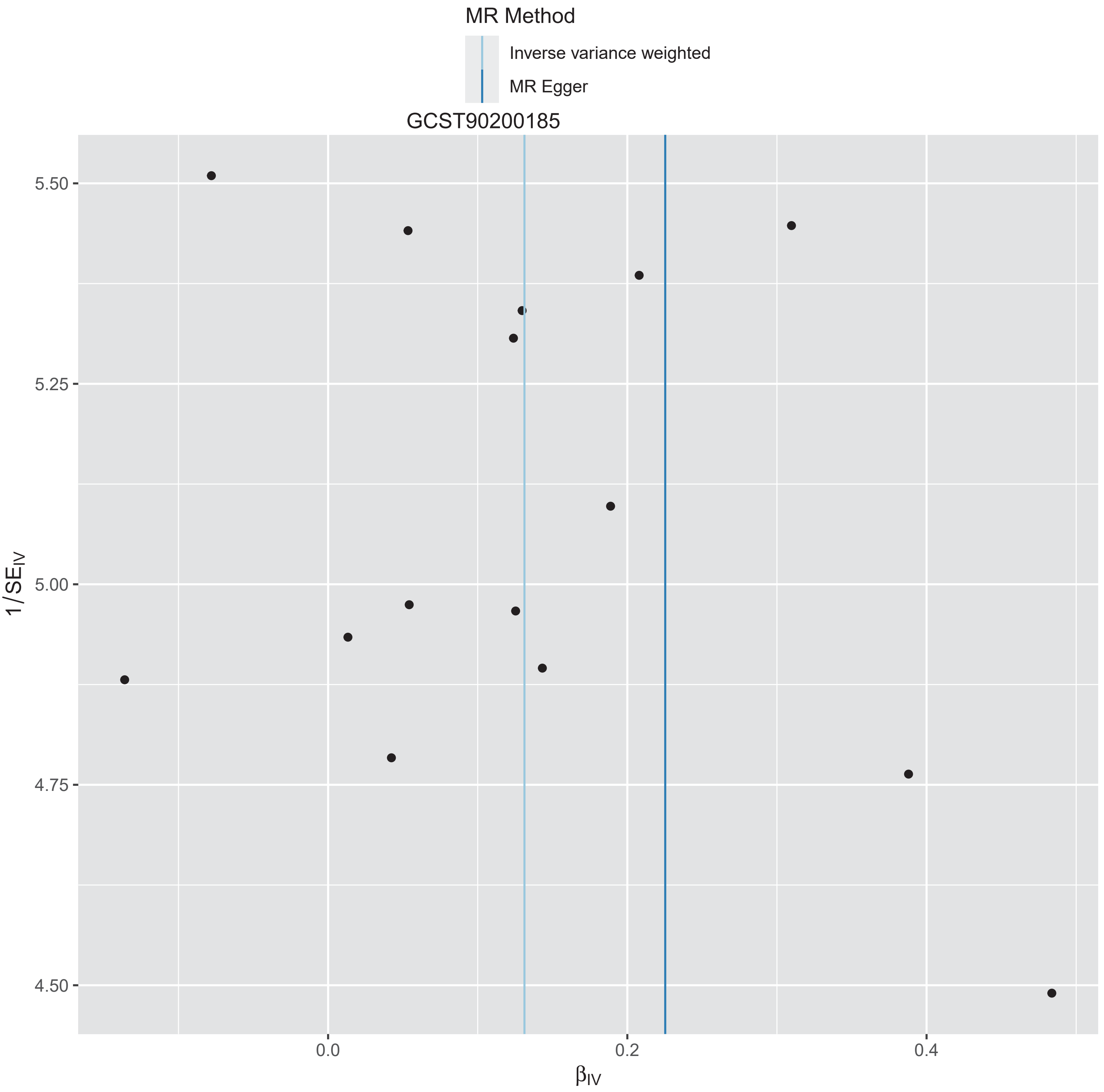

MR Method
Inverse variance weighted
MR Egger
GCST90200185
5.50
5.25
5.00
4.75
4.50
1/SE$_{IV}$
0.0
0.2
0.4
β$_{IV}$

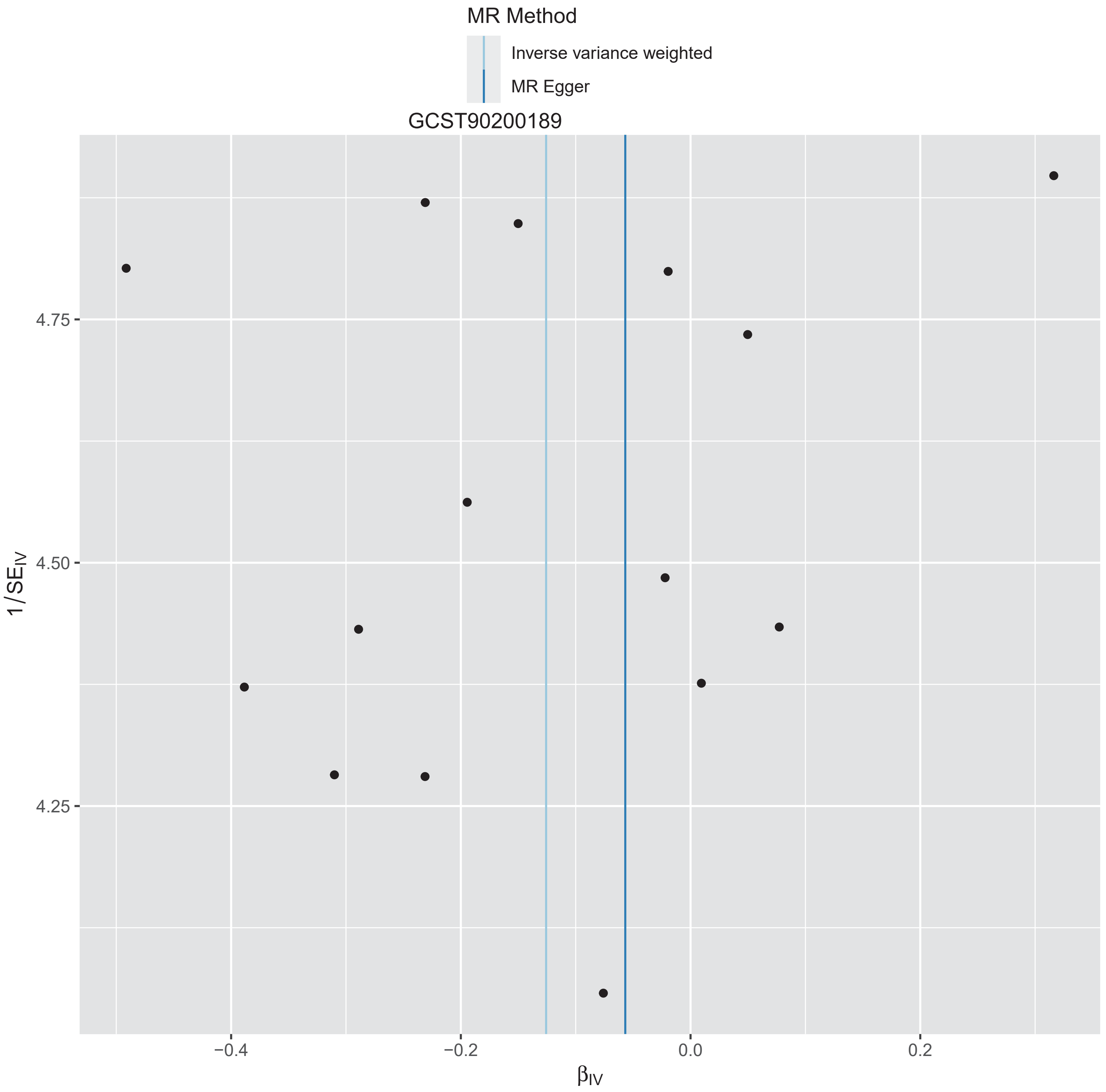
MR Method
Inverse variance weighted
MR Egger
GCST90200189
4.75
4.50
4.25
1/SE_IV
−0.4
−0.2
0.0
0.2
β_IV

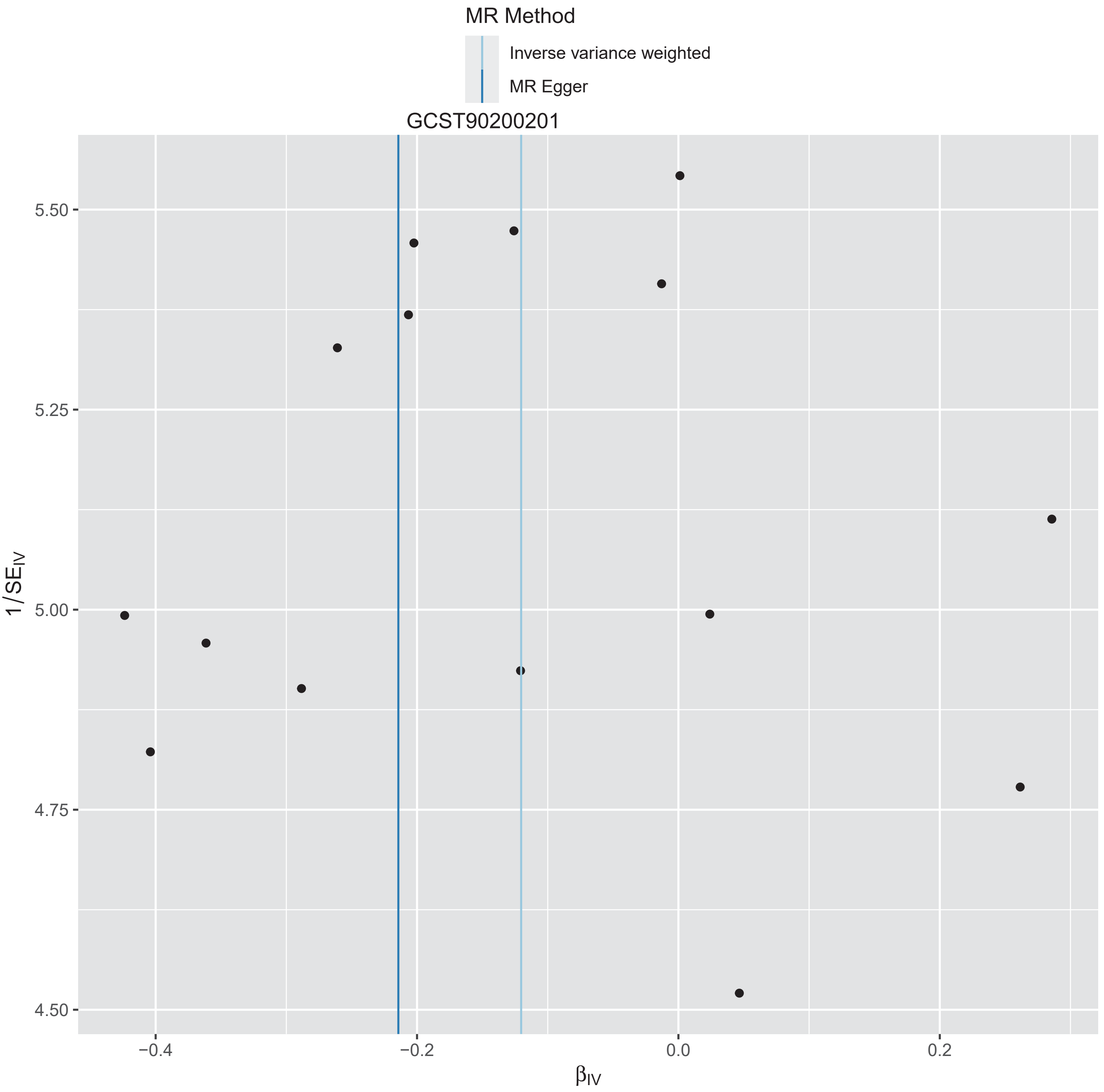
MR Method
Inverse variance weighted
MR Egger
GCST90200201
5.50
5.25
5.00
4.75
4.50
1/SE_IV
−0.4
−0.2
0.0
0.2
β_IV

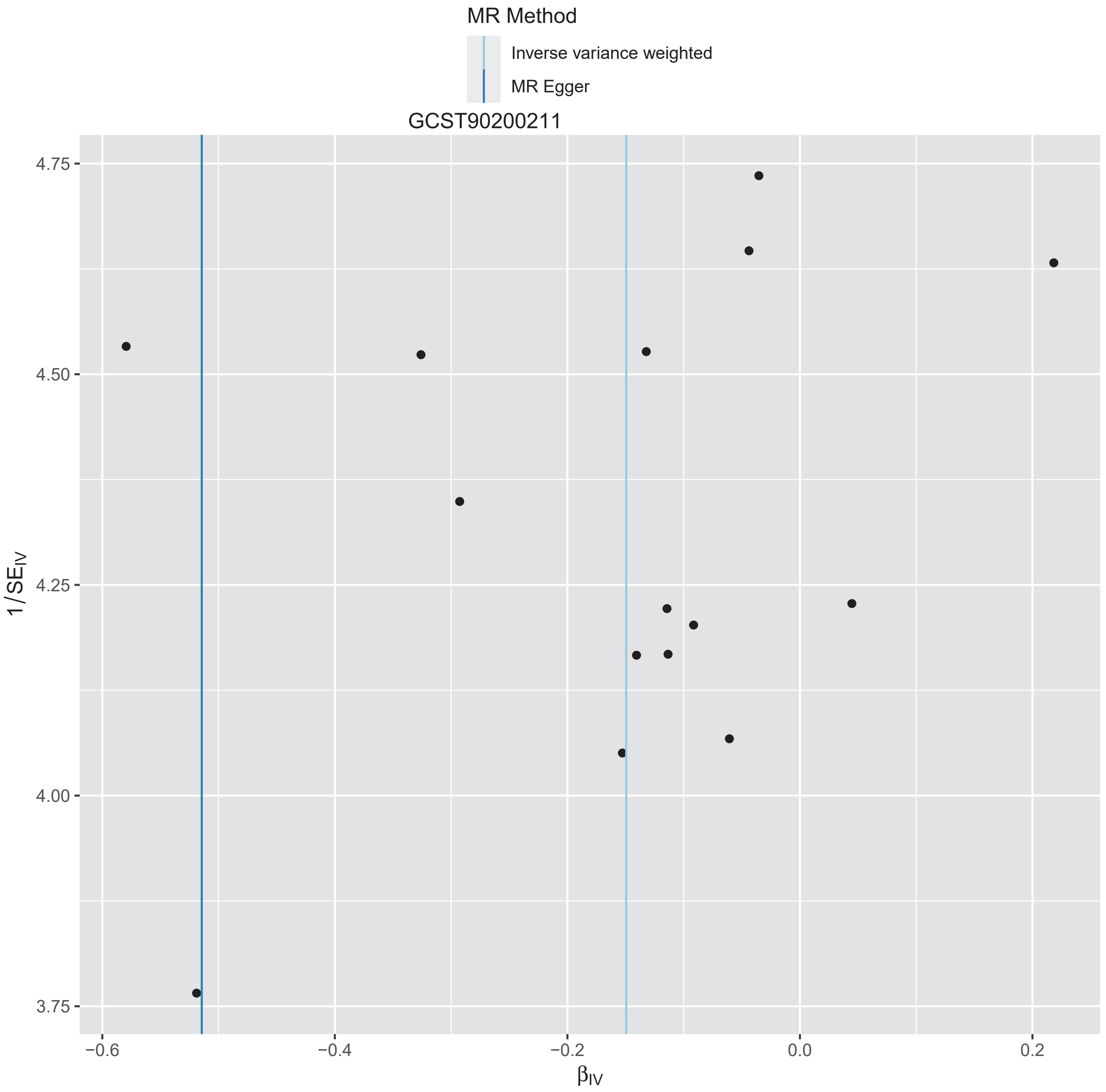
MR Method
Inverse variance weighted
MR Egger
GCST90200211
4.75
4.50
4.25
4.00
3.75
1/SE_IV
−0.6
−0.4
−0.2
0.0
0.2
β_IV

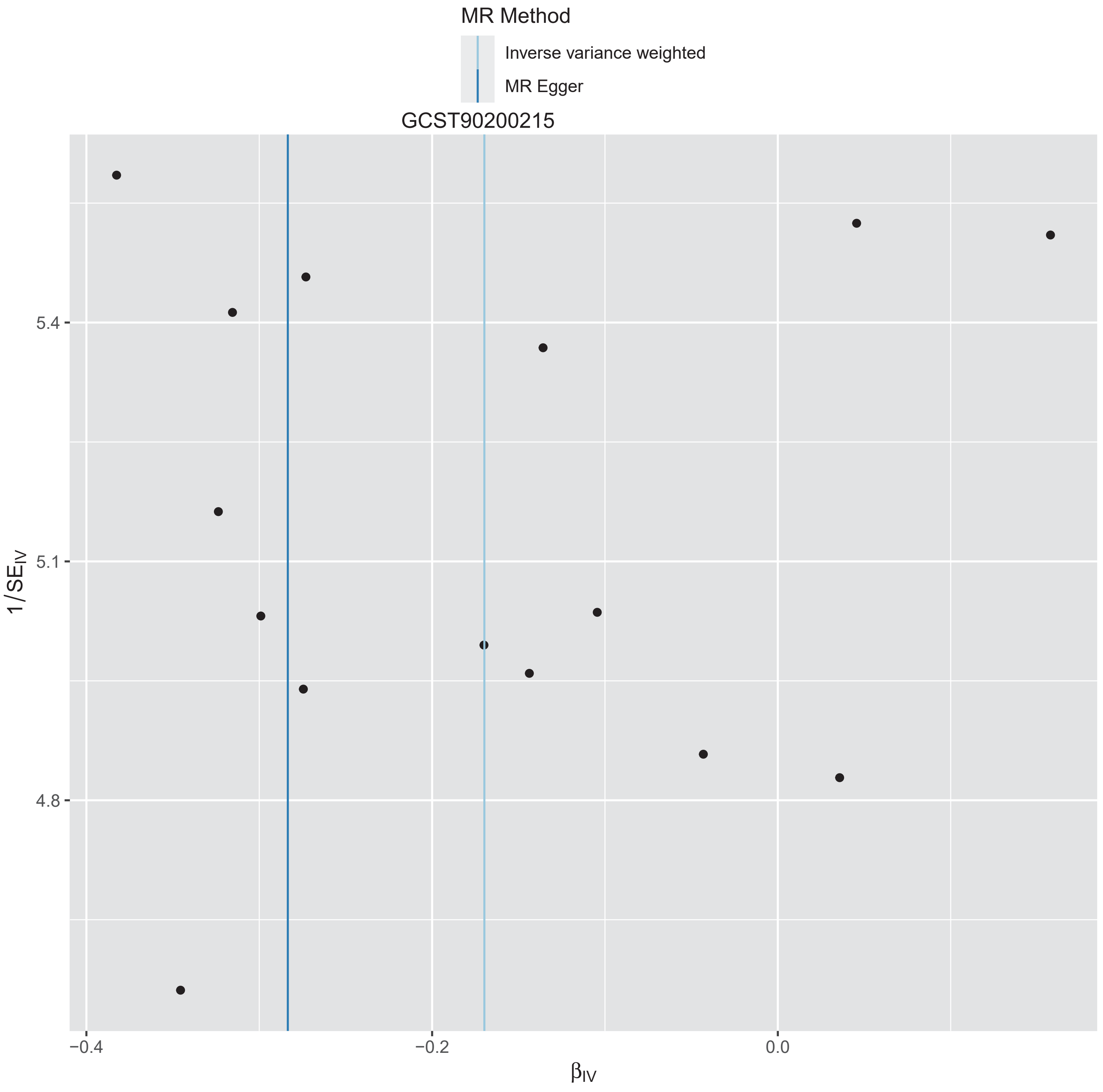
MR Method
Inverse variance weighted
MR Egger
GCST90200215
5.4
5.1
4.8
1/SE$_{IV}$
−0.4
−0.2
0.0
β$_{IV}$

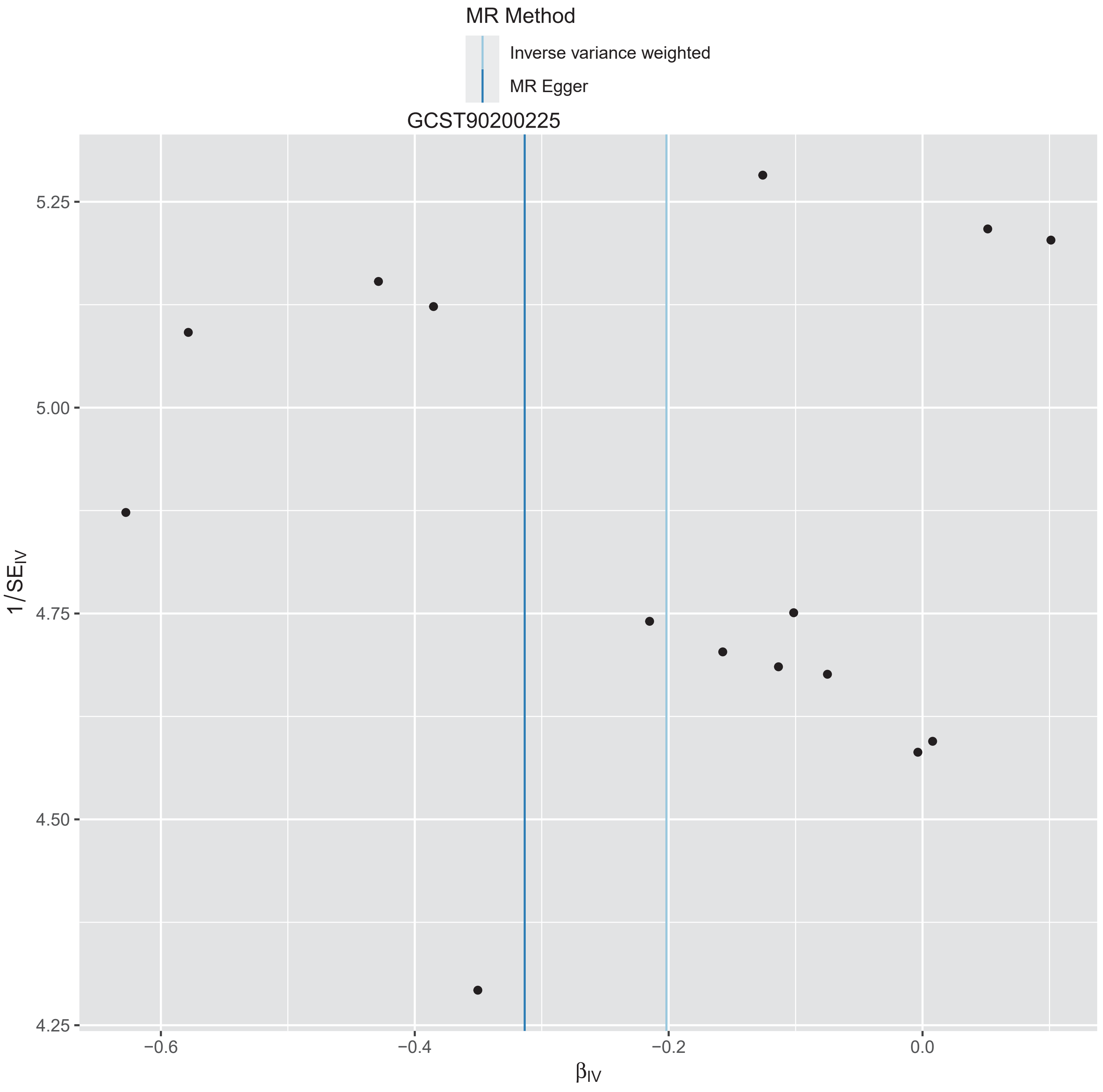

MR Method
Inverse variance weighted
MR Egger
GCST90200225
5.25
5.00
4.75
4.50
4.25
1/SE$_{IV}$
−0.6
−0.4
−0.2
0.0
β$_{IV}$

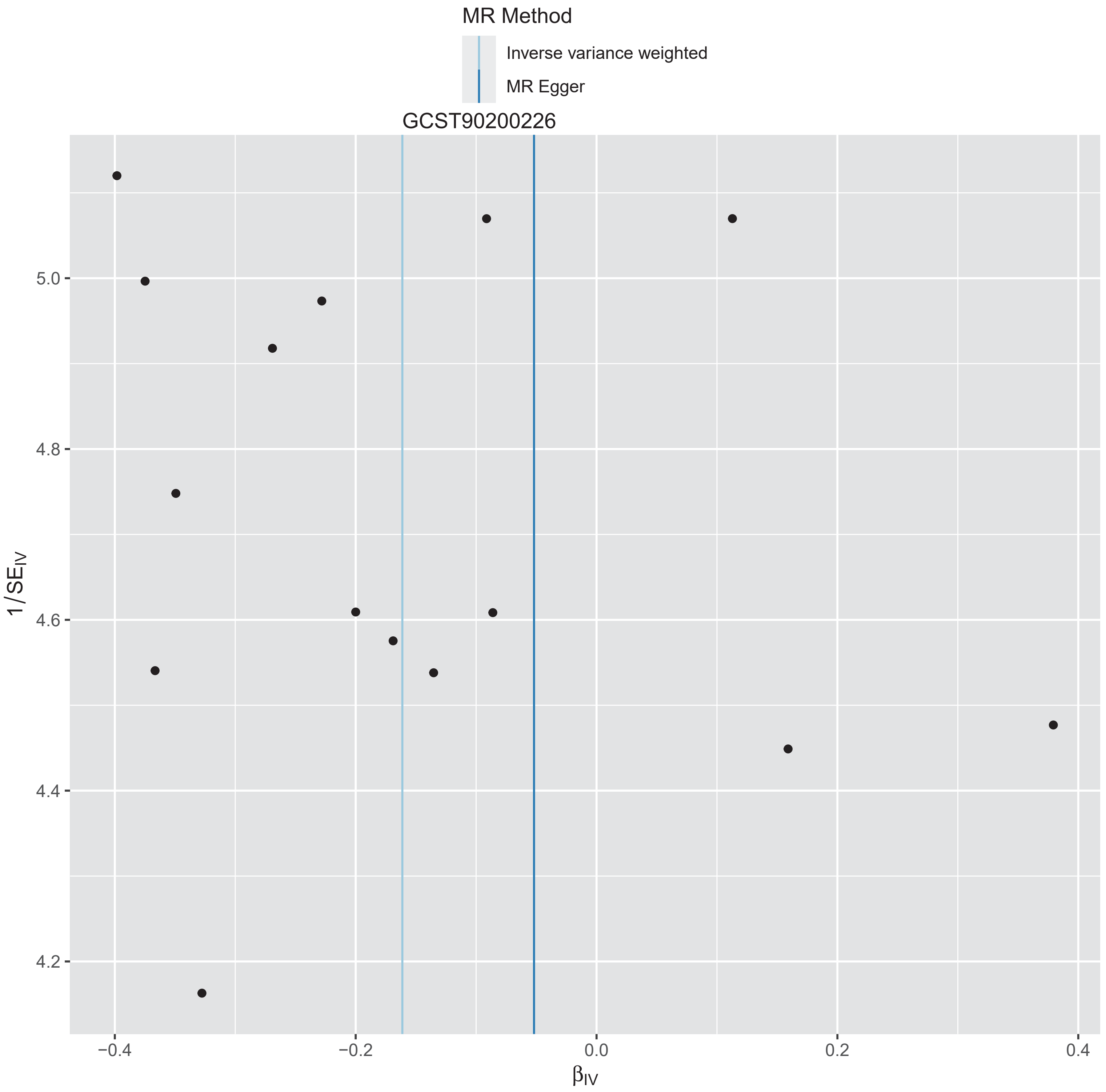

MR Method
Inverse variance weighted
MR Egger
GCST90200226
5.0
4.8
4.6
4.4
4.2
1/SE$_{IV}$
−0.4
−0.2
0.0
0.2
0.4
β$_{IV}$

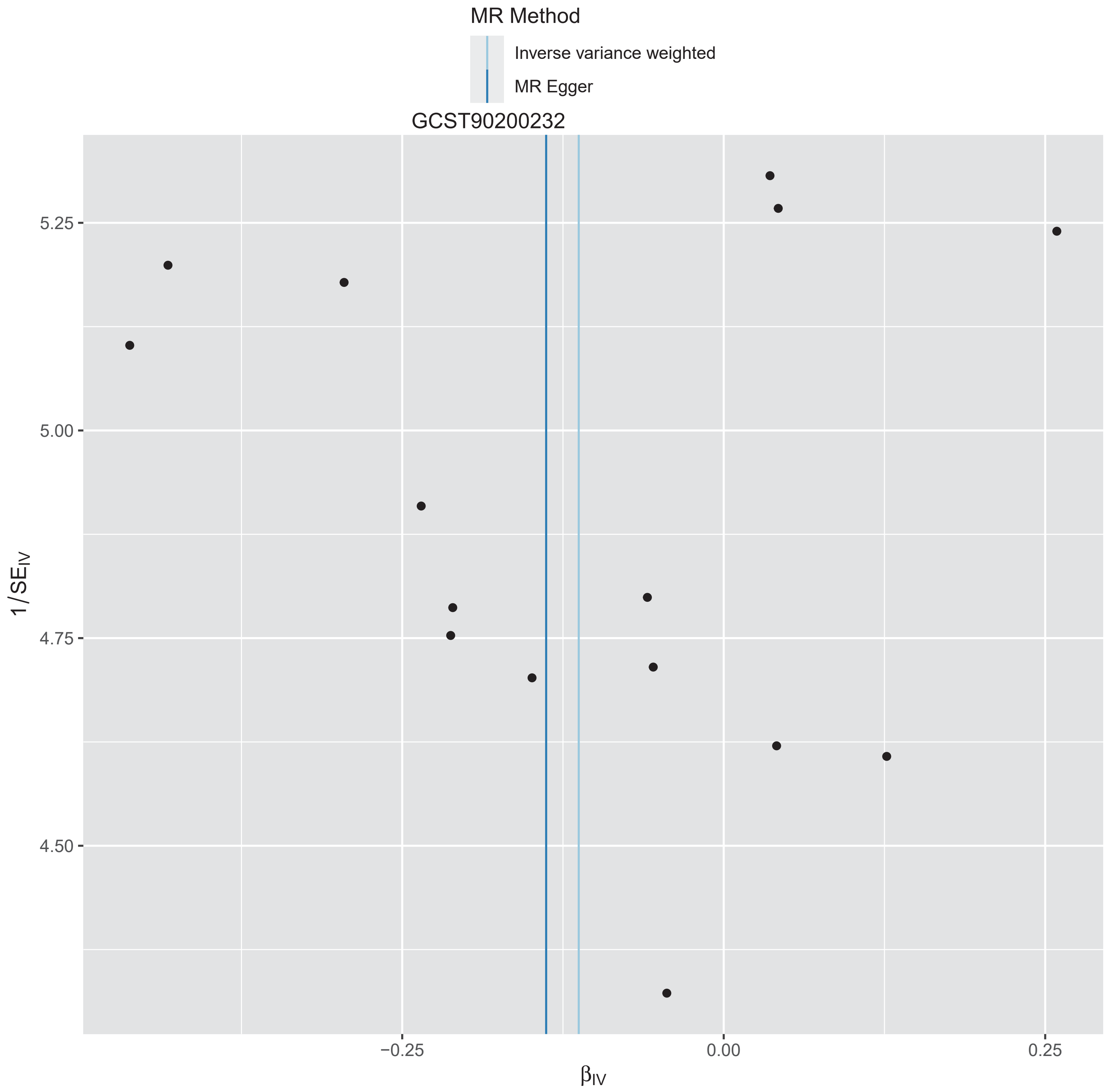

MR Method
Inverse variance weighted
MR Egger
GCST90200232
5.25
5.00
4.75
4.50
1/SE$_{IV}$
−0.25
0.00
0.25
β$_{IV}$

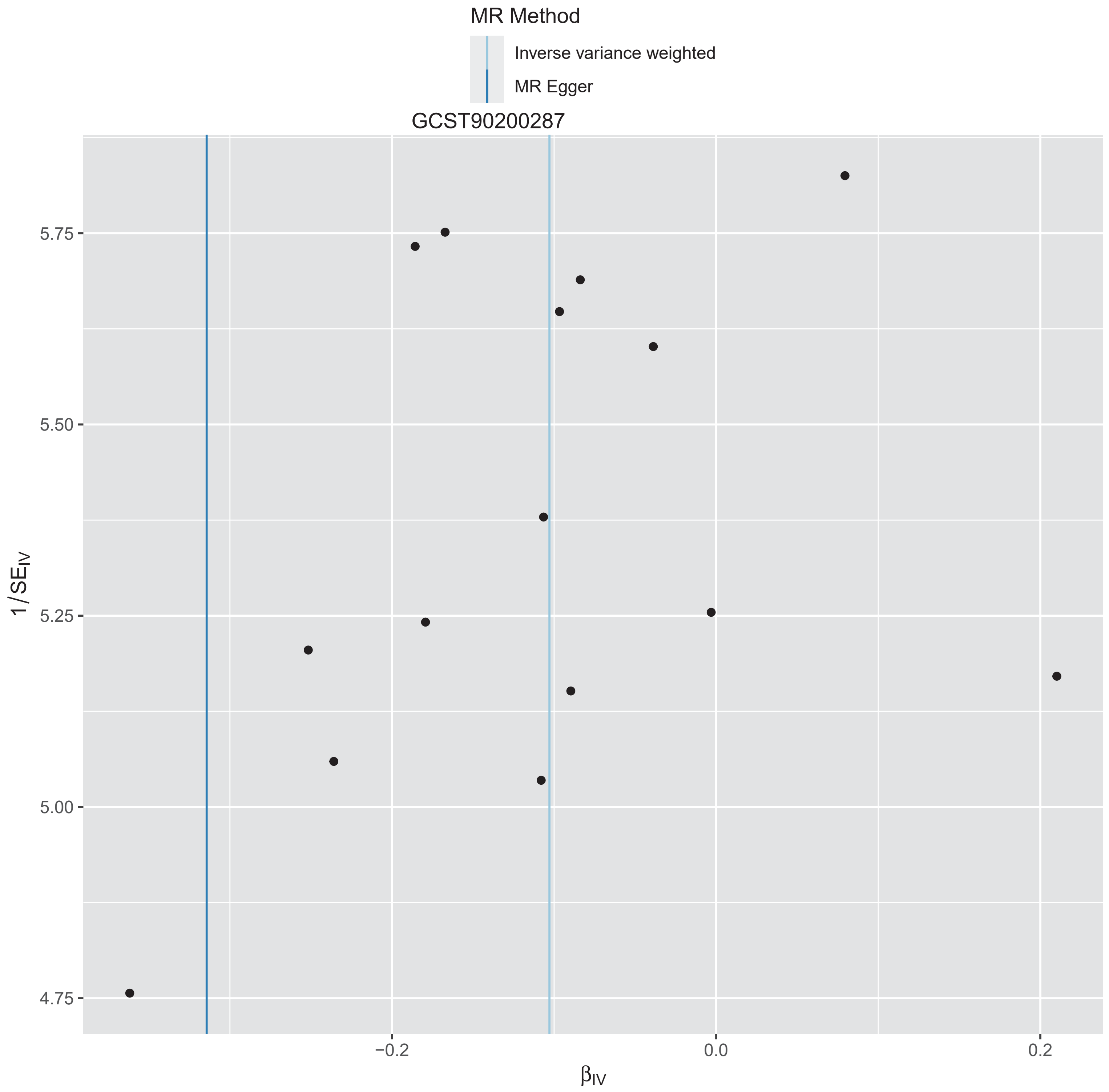
MR Method
Inverse variance weighted
MR Egger
GCST90200287
5.75
5.50
5.25
5.00
4.75
1/SE_IV
−0.2
0.0
0.2
β_IV

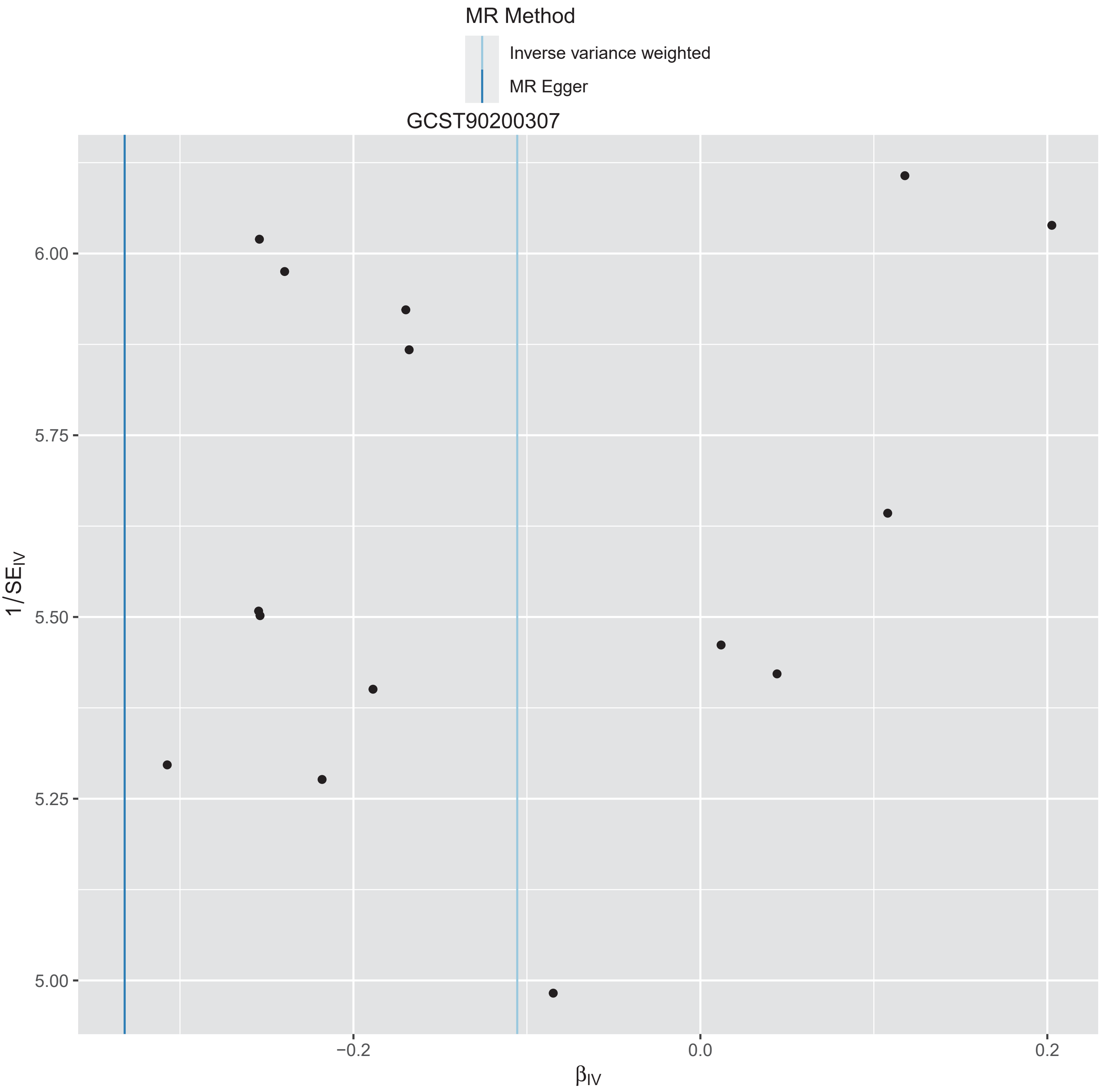
MR Method
Inverse variance weighted
MR Egger
GCST90200307
6.00
5.75
5.50
5.25
5.00
1/SE$_{IV}$
−0.2
0.0
0.2
β$_{IV}$

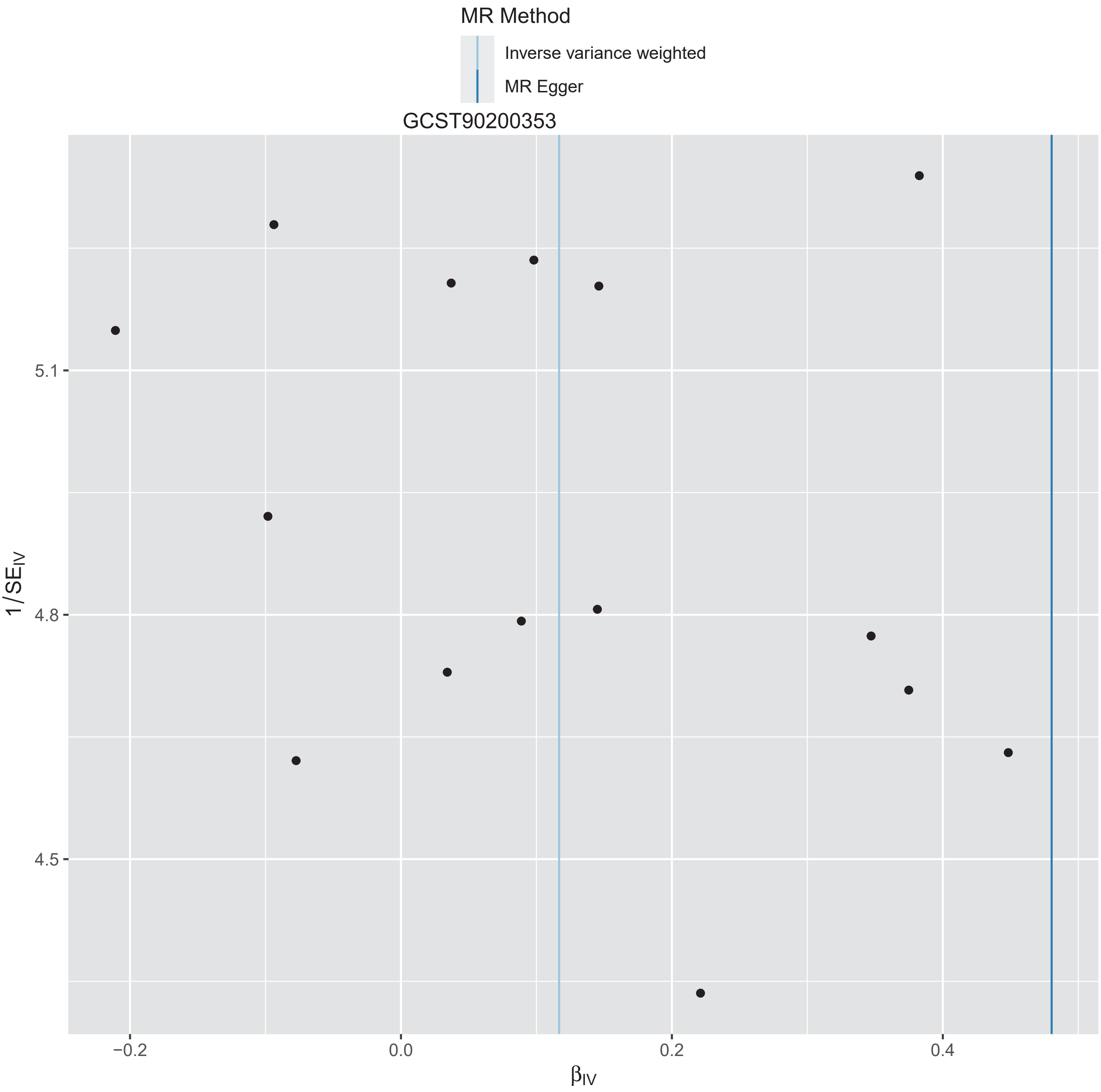
MR Method
Inverse variance weighted
MR Egger
GCST90200353
1/SE_IV
5.1
4.8
4.5
−0.2
0.0
0.2
0.4
β_IV

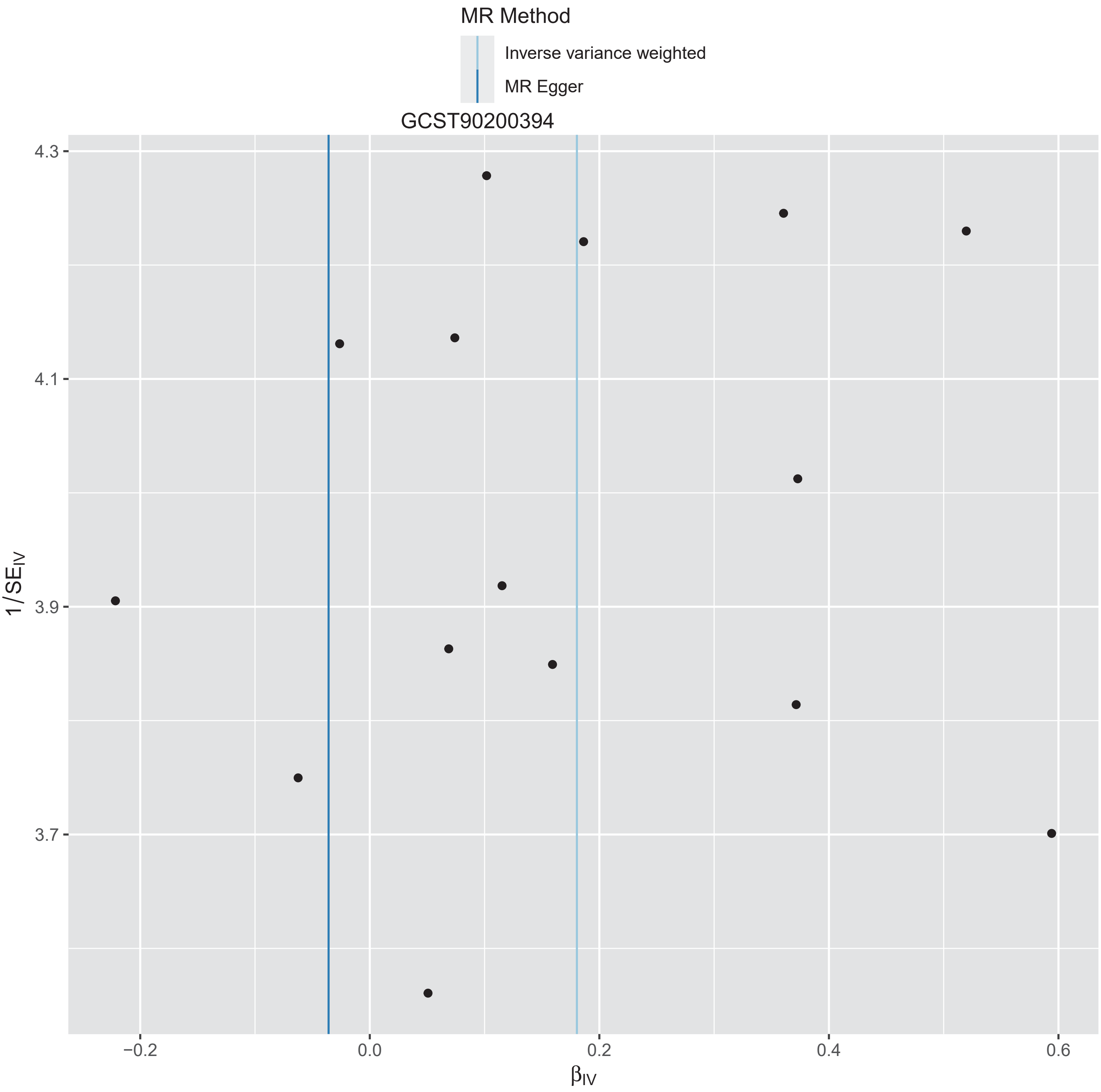
MR Method
Inverse variance weighted
MR Egger
GCST90200394
4.3
4.1
3.9
3.7
1/SE_{IV}
−0.2
0.0
0.2
0.4
0.6
β_{IV}

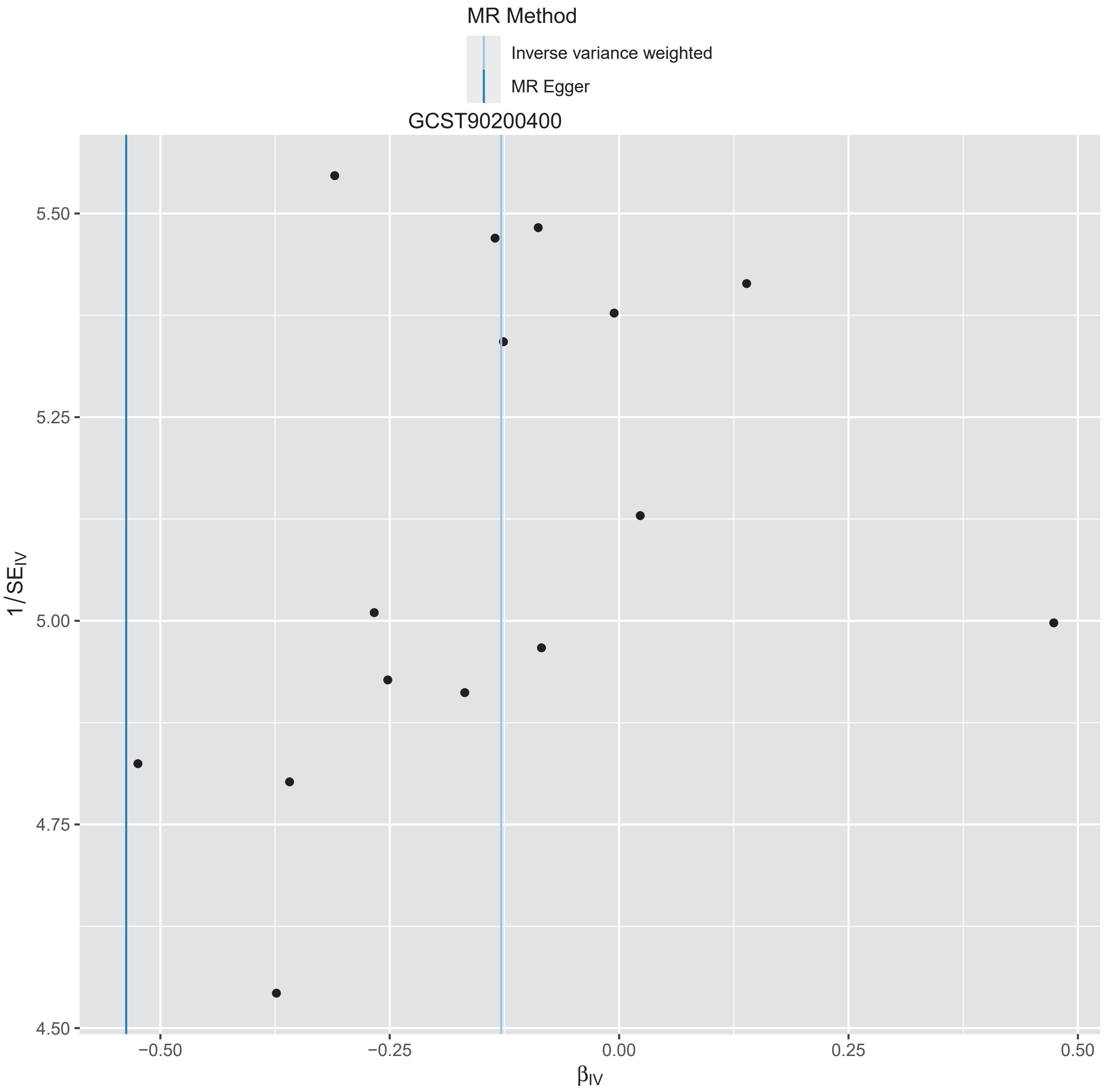
MR Method
Inverse variance weighted
MR Egger
GCST90200400
5.50
5.25
5.00
4.75
4.50
1/SE$_{IV}$
−0.50
−0.25
0.00
0.25
0.50
β$_{IV}$

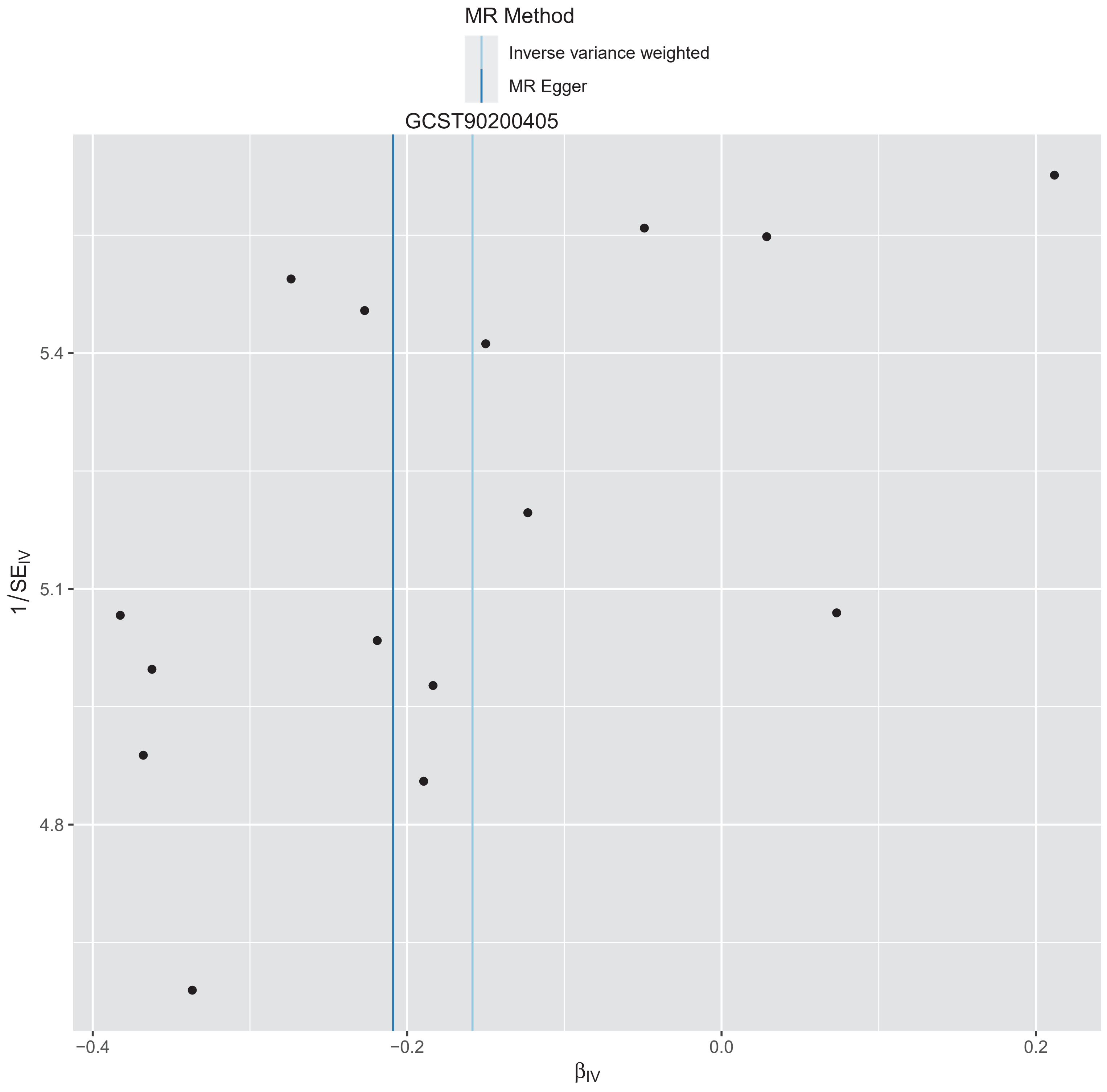
MR Method
Inverse variance weighted
MR Egger
GCST90200405
5.4
5.1
4.8
1/SE$_{IV}$
−0.4
−0.2
0.0
0.2
β$_{IV}$

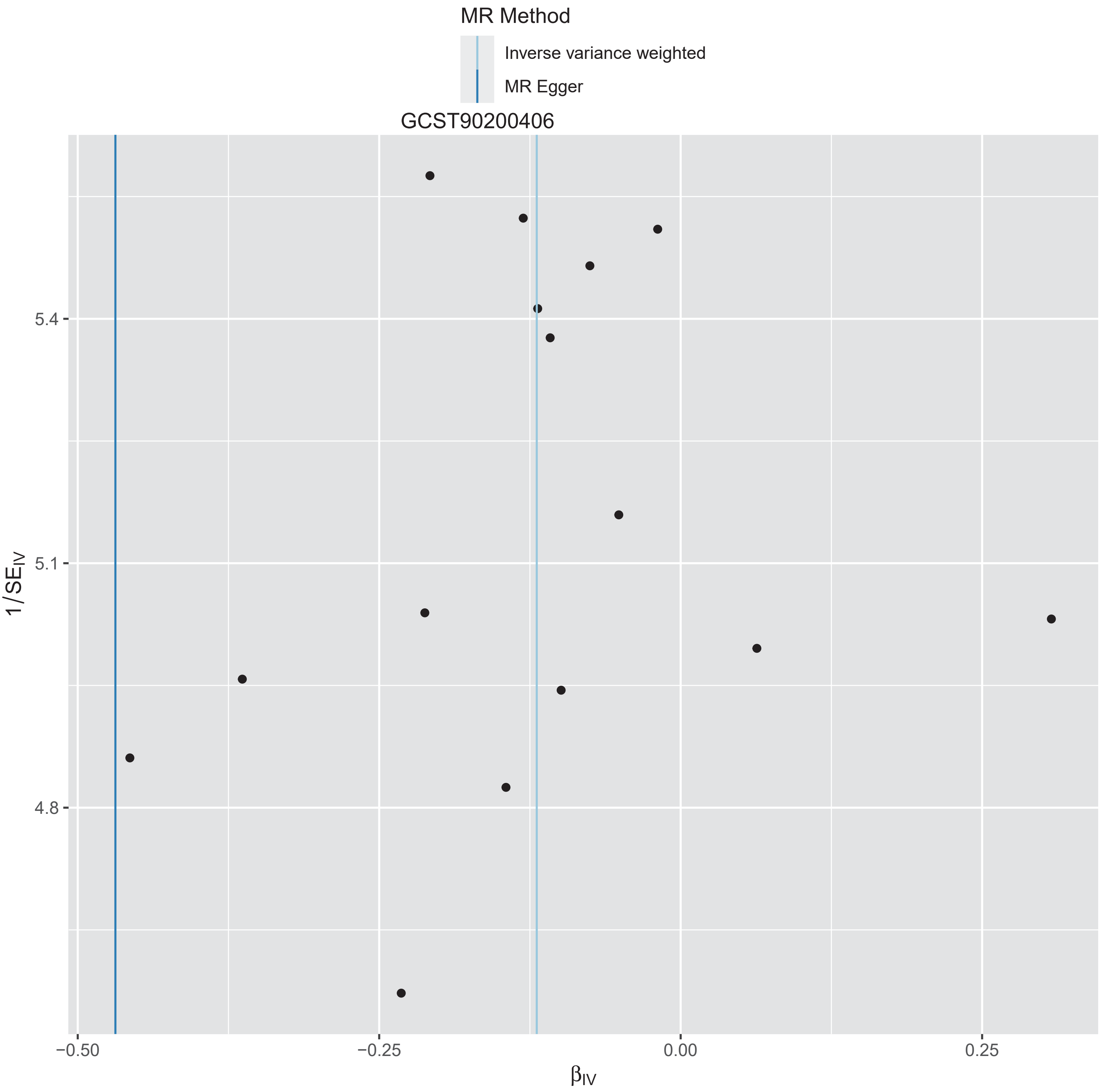
MR Method
Inverse variance weighted
MR Egger
GCST90200406
5.4
5.1
4.8
1/SE$_{IV}$
−0.50
−0.25
0.00
0.25
β$_{IV}$

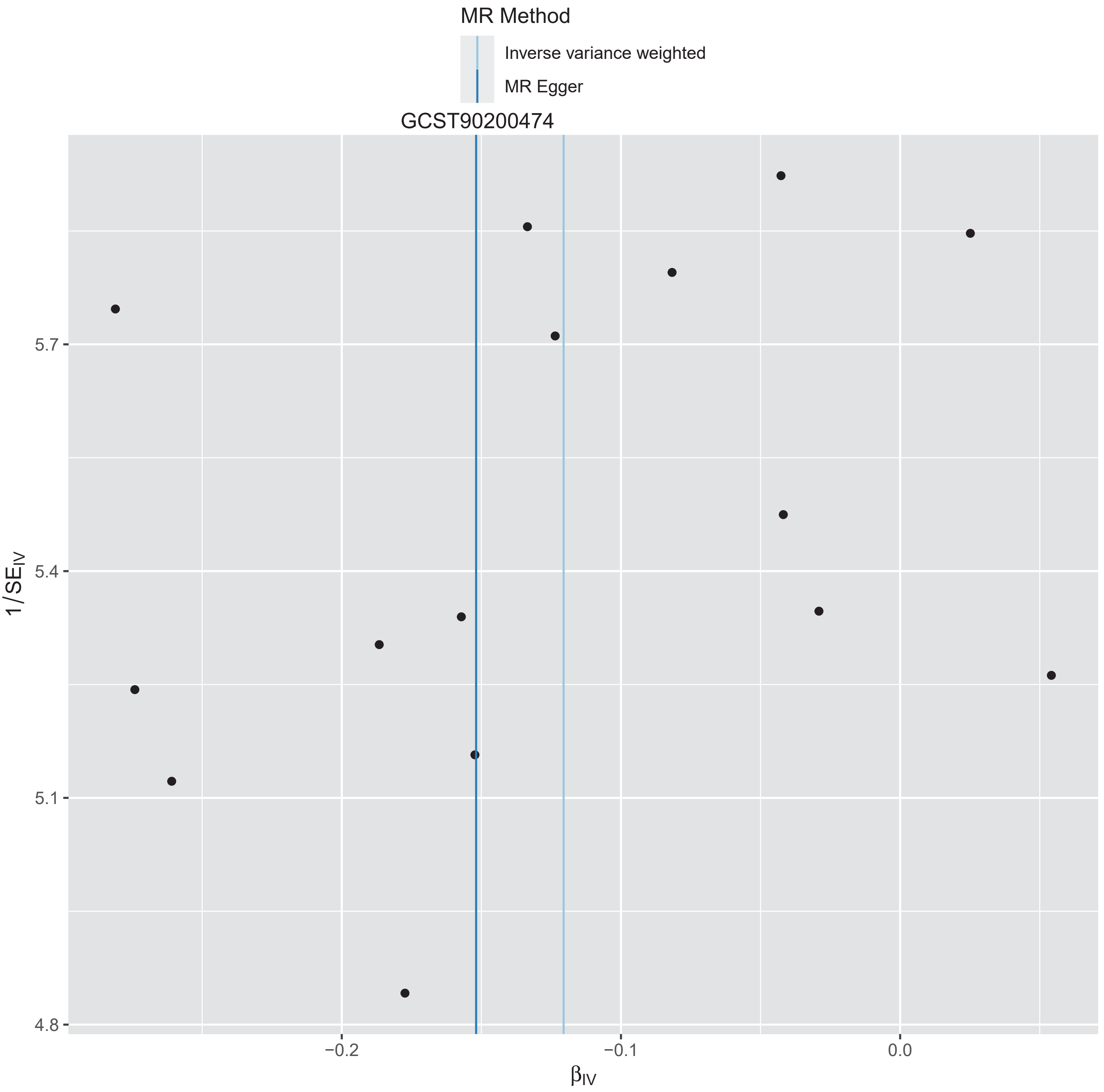

MR Method
Inverse variance weighted
MR Egger
GCST90200474
1/SE$_{IV}$
5.7
5.4
5.1
4.8
−0.2
−0.1
0.0
β$_{IV}$

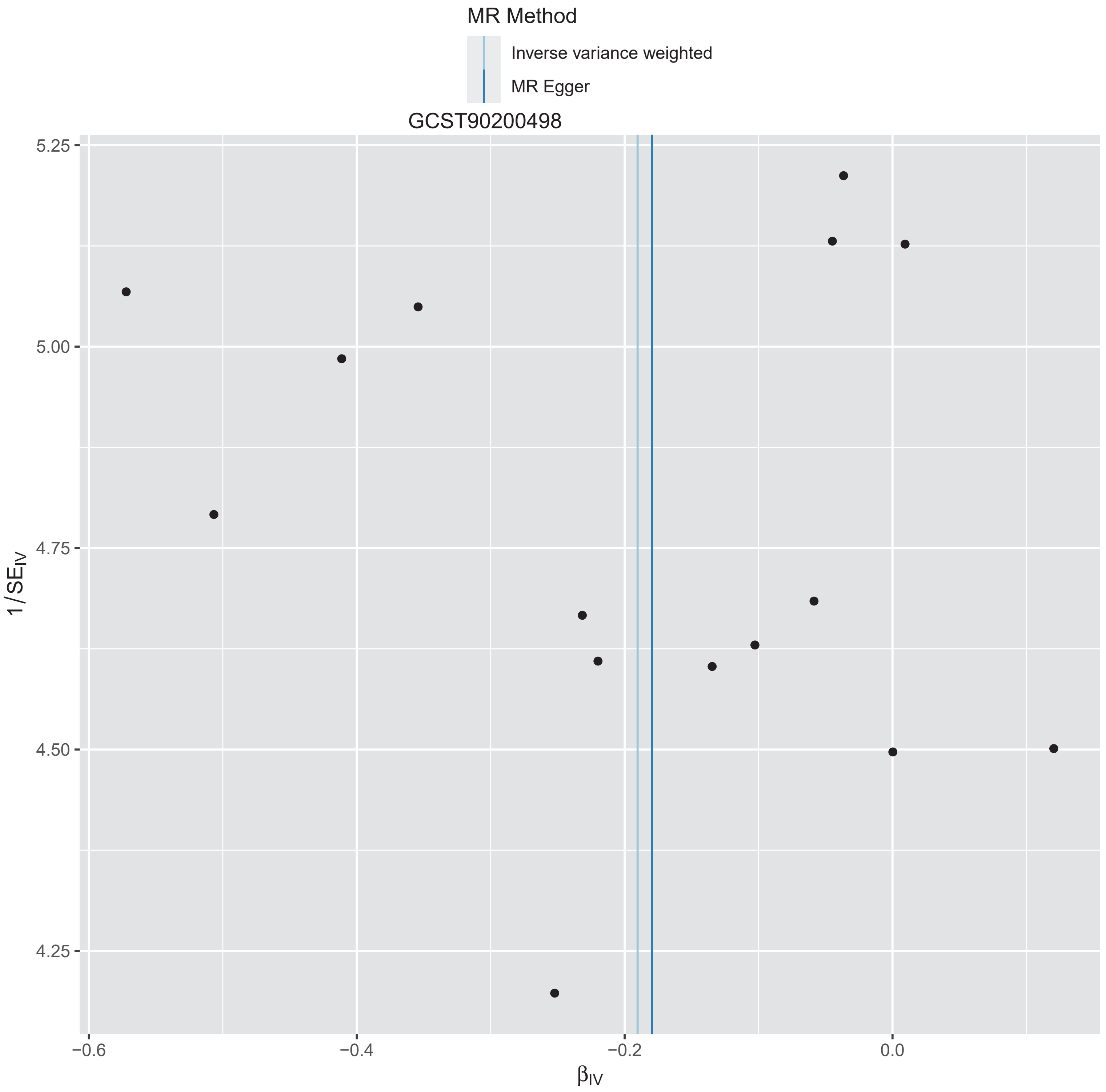

MR Method
Inverse variance weighted
MR Egger
GCST90200498
5.25
5.00
4.75
4.50
4.25
1/SE$_{IV}$
−0.6
−0.4
−0.2
0.0
β$_{IV}$

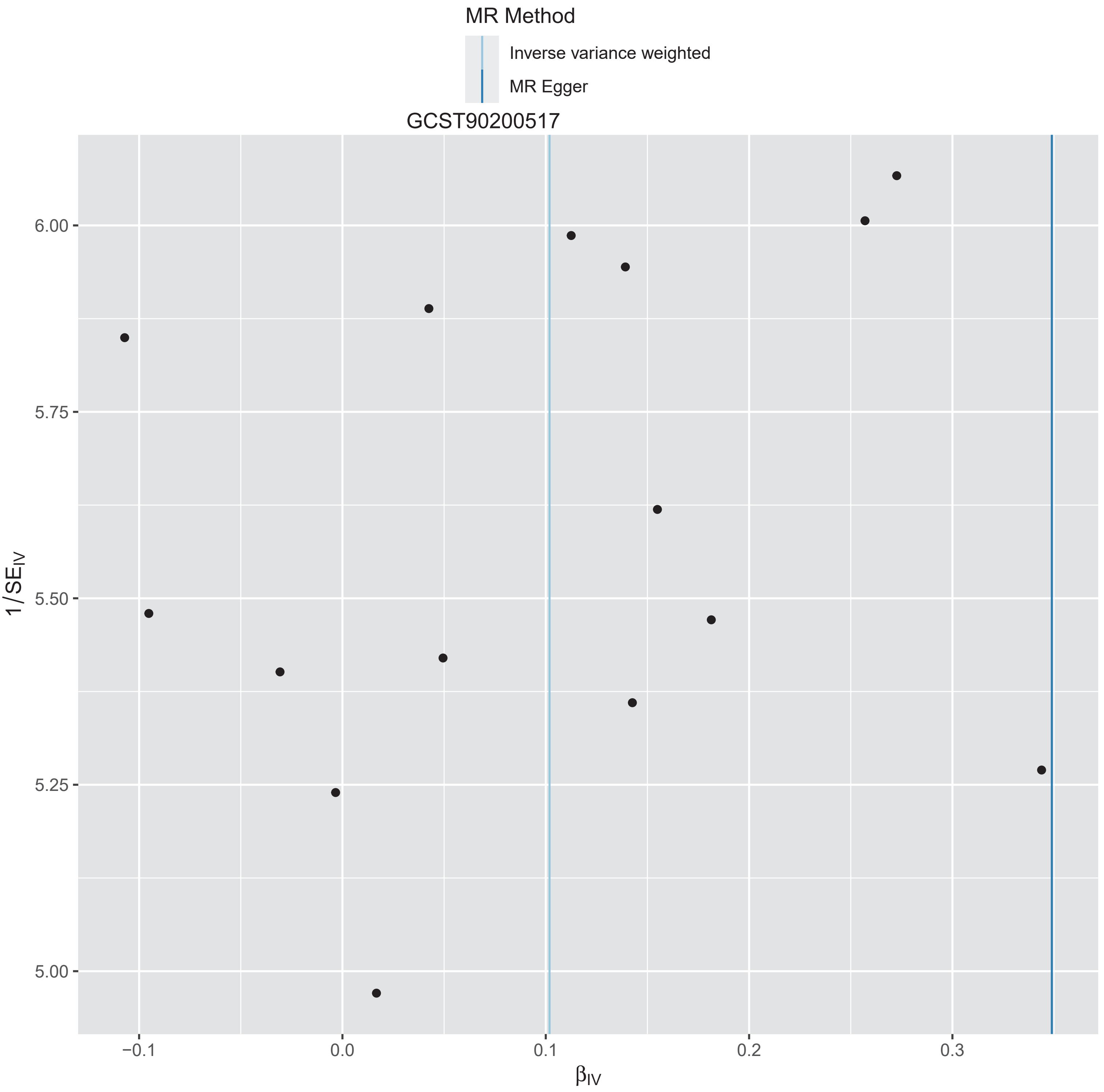
MR Method
Inverse variance weighted
MR Egger
GCST90200517
6.00
5.75
5.50
5.25
5.00
1/SE$_{IV}$
−0.1
0.0
0.1
0.2
0.3
β$_{IV}$

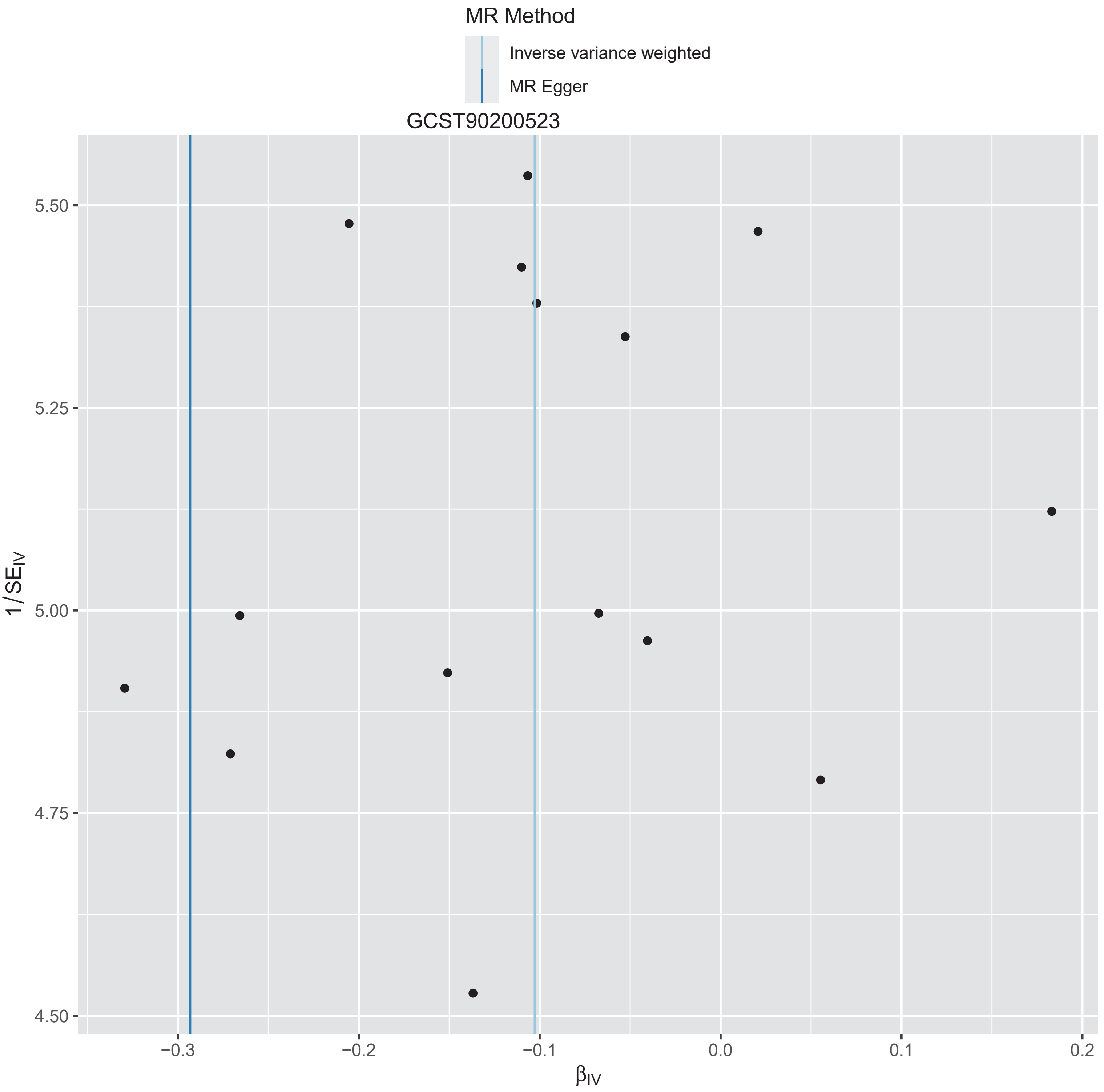
MR Method
Inverse variance weighted
MR Egger
GCST90200523
5.50
5.25
5.00
4.75
4.50
1/SE_IV
−0.3
−0.2
−0.1
0.0
0.1
0.2
β_IV

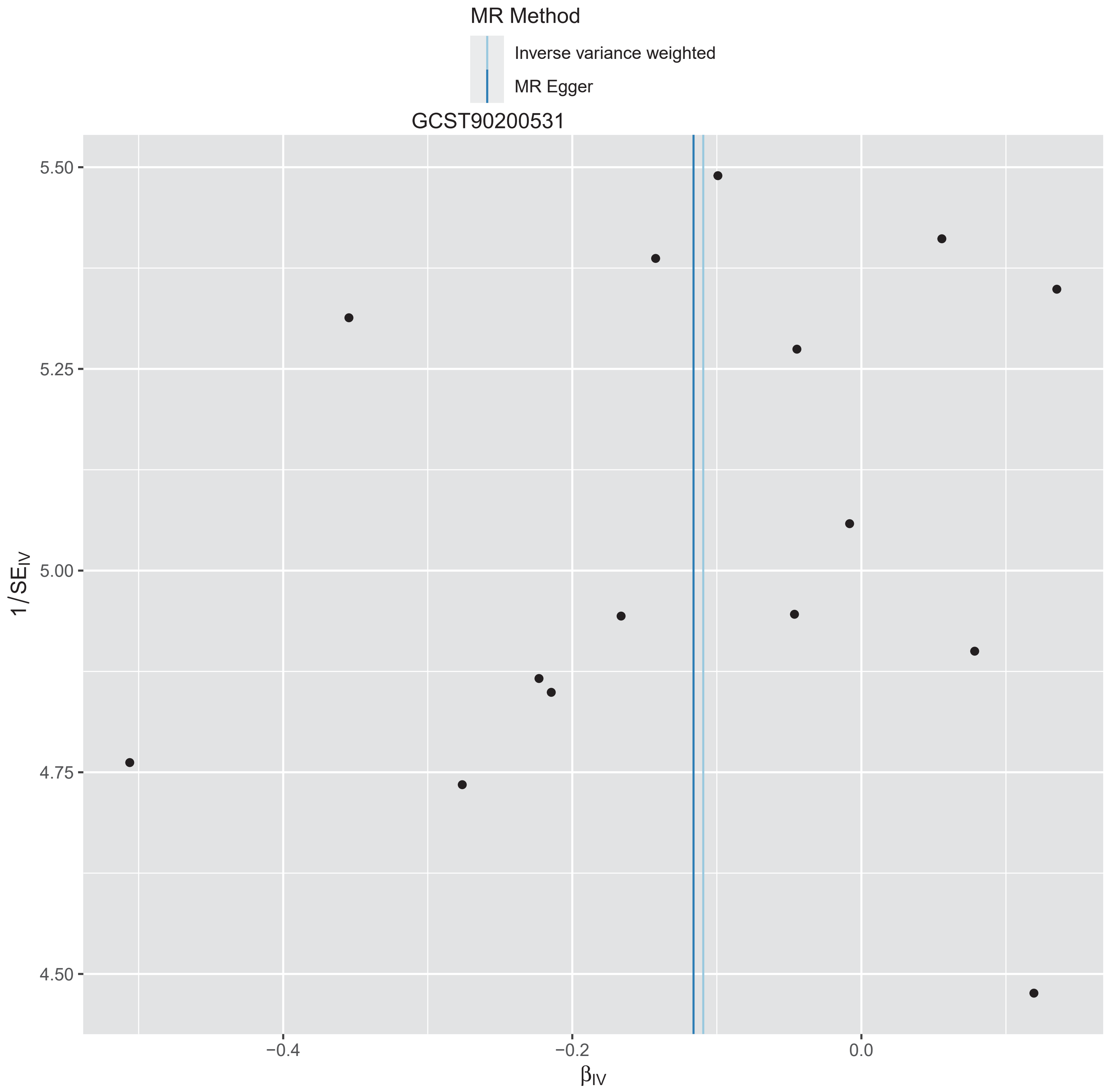
MR Method
Inverse variance weighted
MR Egger
GCST90200531
5.50
5.25
5.00
4.75
4.50
1/SE_IV
−0.4
−0.2
0.0
β_IV

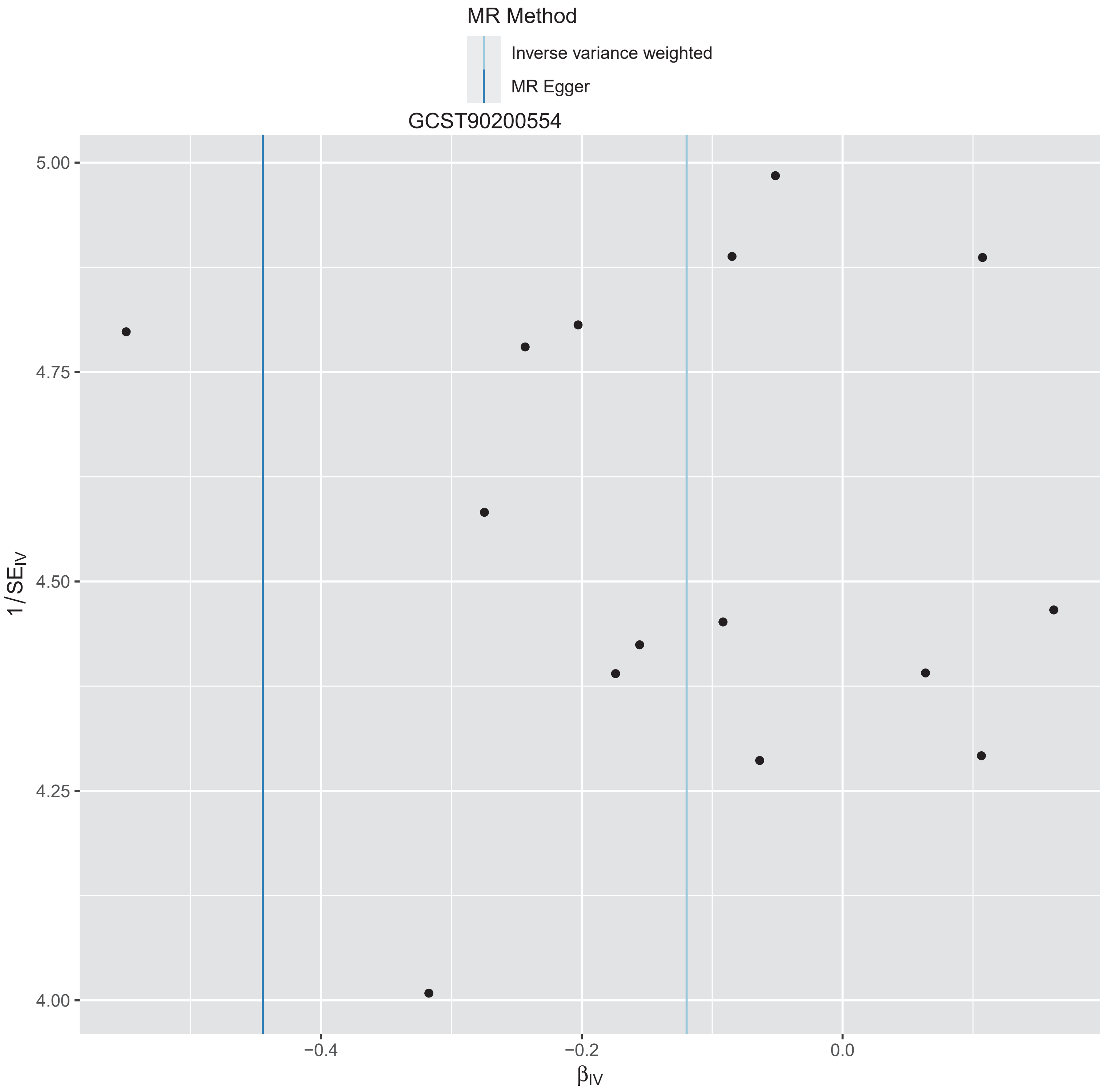

MR Method
Inverse variance weighted
MR Egger
GCST90200554
5.00
4.75
4.50
4.25
4.00
1/SE$_{IV}$
−0.4
−0.2
0.0
β$_{IV}$

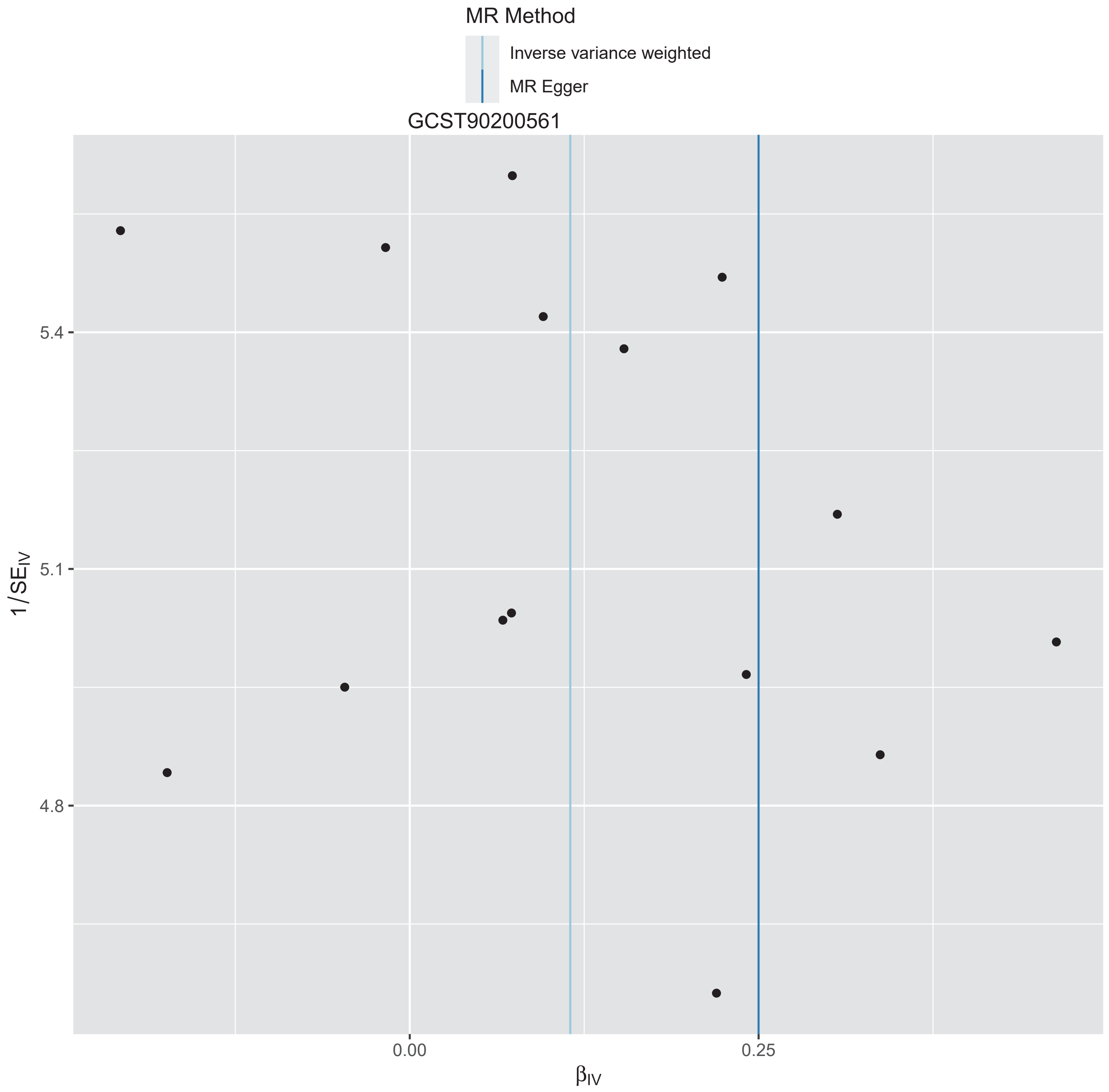

MR Method
Inverse variance weighted
MR Egger
GCST90200561
5.4
5.1
4.8
1/SE_IV
0.00
0.25
β_IV

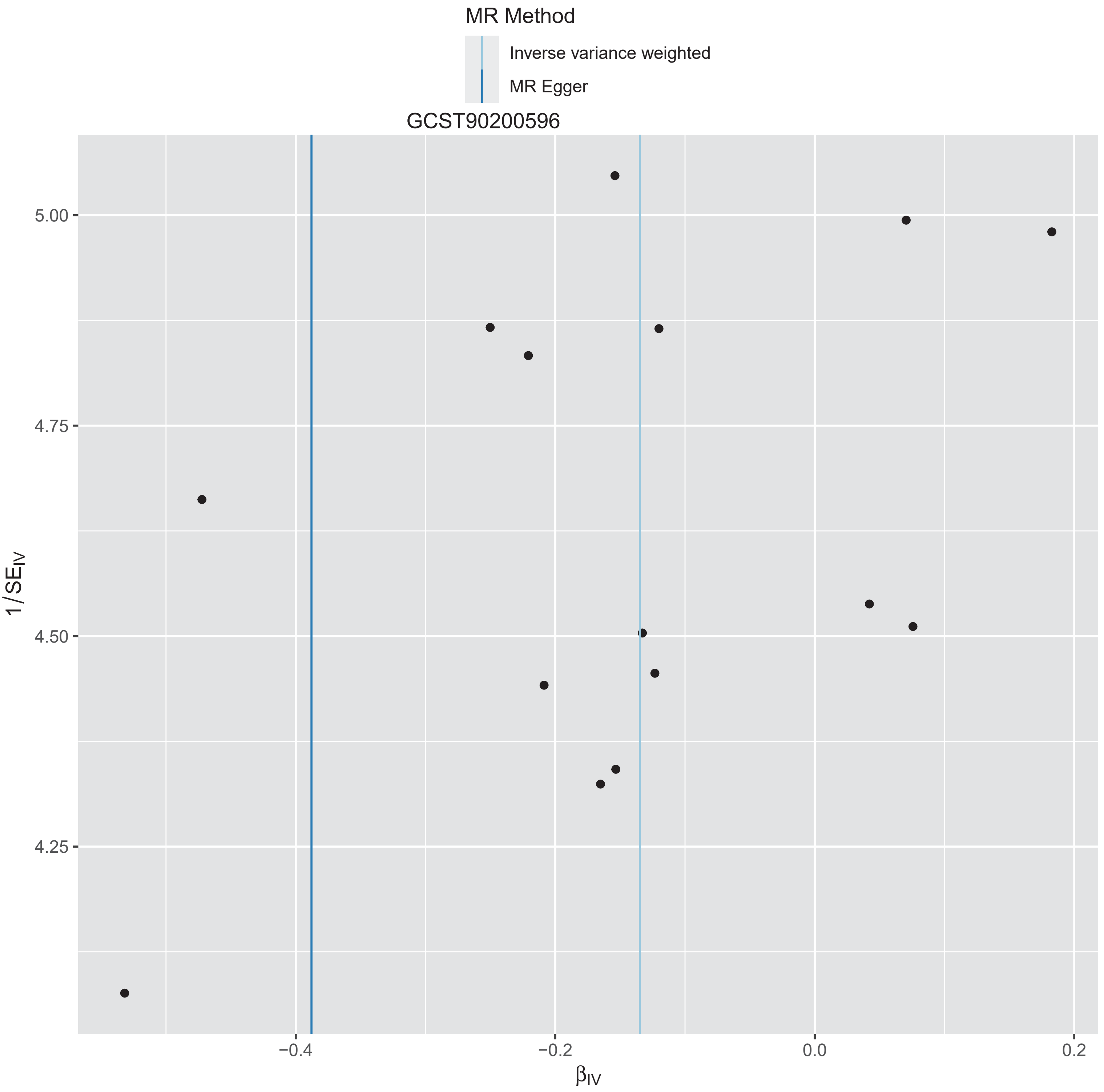

MR Method
Inverse variance weighted
MR Egger
GCST90200596
5.00
4.75
4.50
4.25
1/SE$_{IV}$
−0.4
−0.2
0.0
0.2
β$_{IV}$

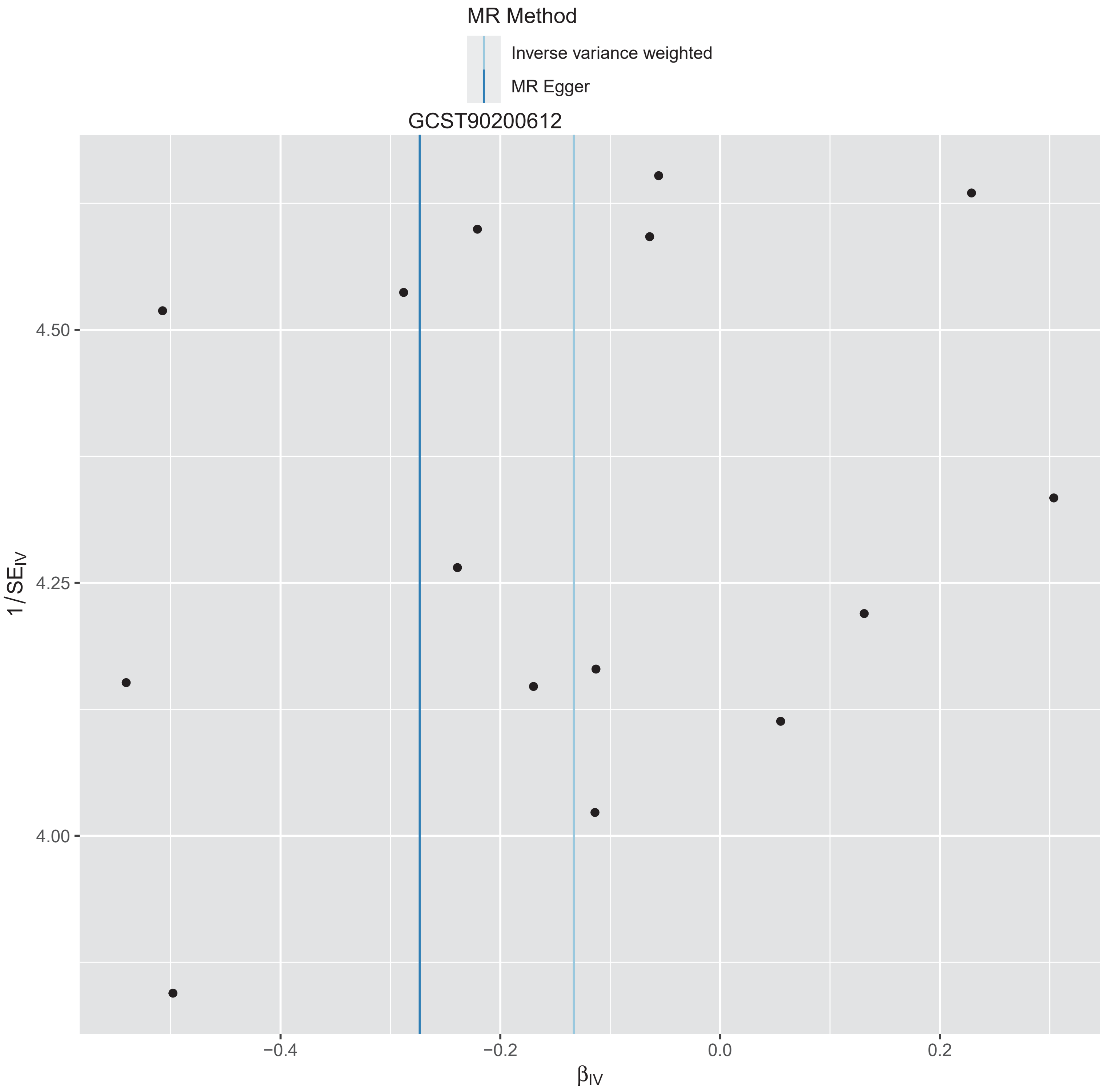

MR Method
Inverse variance weighted
MR Egger
GCST90200612
4.50
4.25
4.00
1/SE$_{IV}$
−0.4
−0.2
0.0
0.2
β$_{IV}$

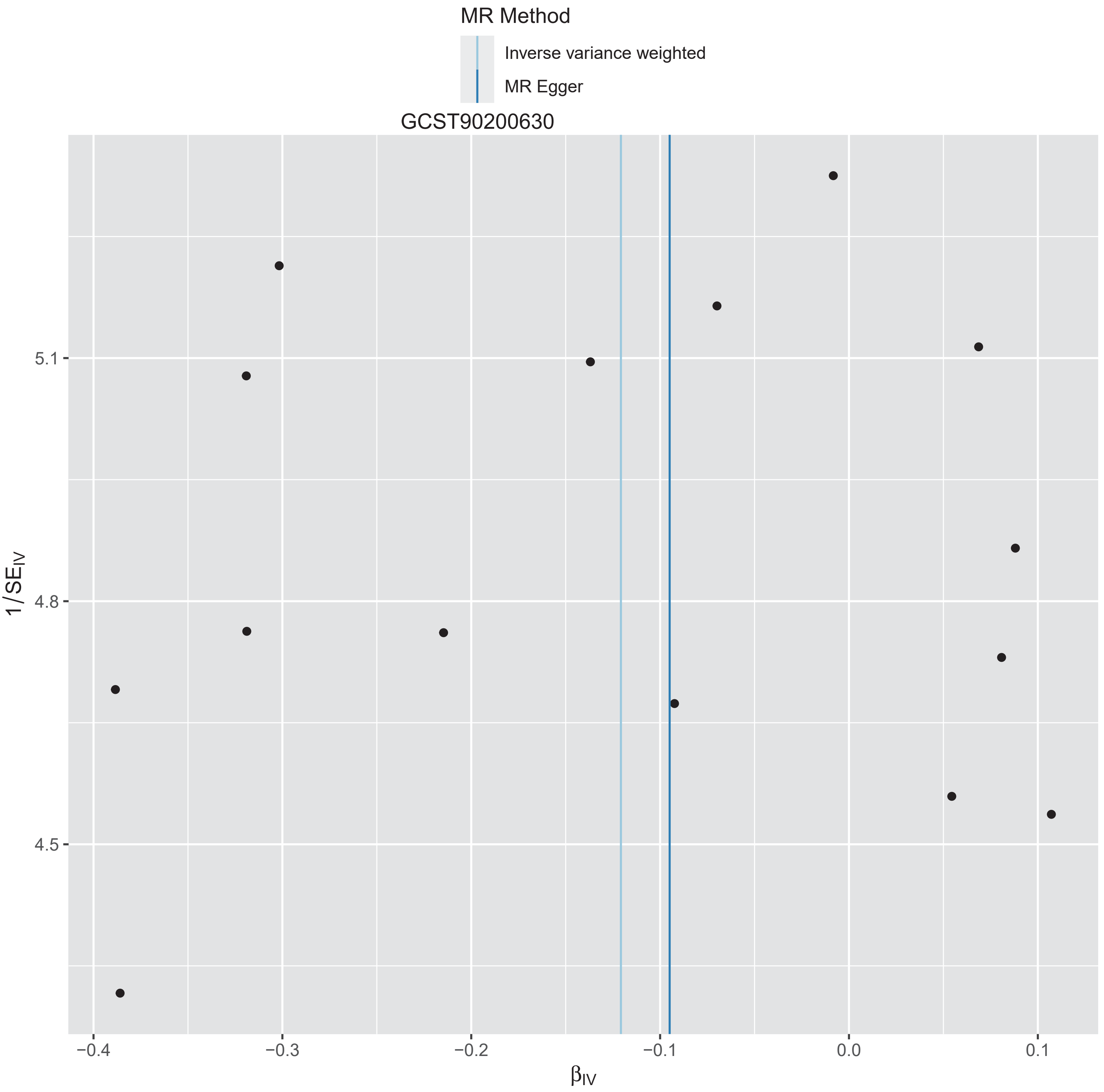
MR Method
Inverse variance weighted
MR Egger
GCST90200630
5.1
4.8
4.5
1/SE$_{IV}$
−0.4
−0.3
−0.2
−0.1
0.0
0.1
β$_{IV}$

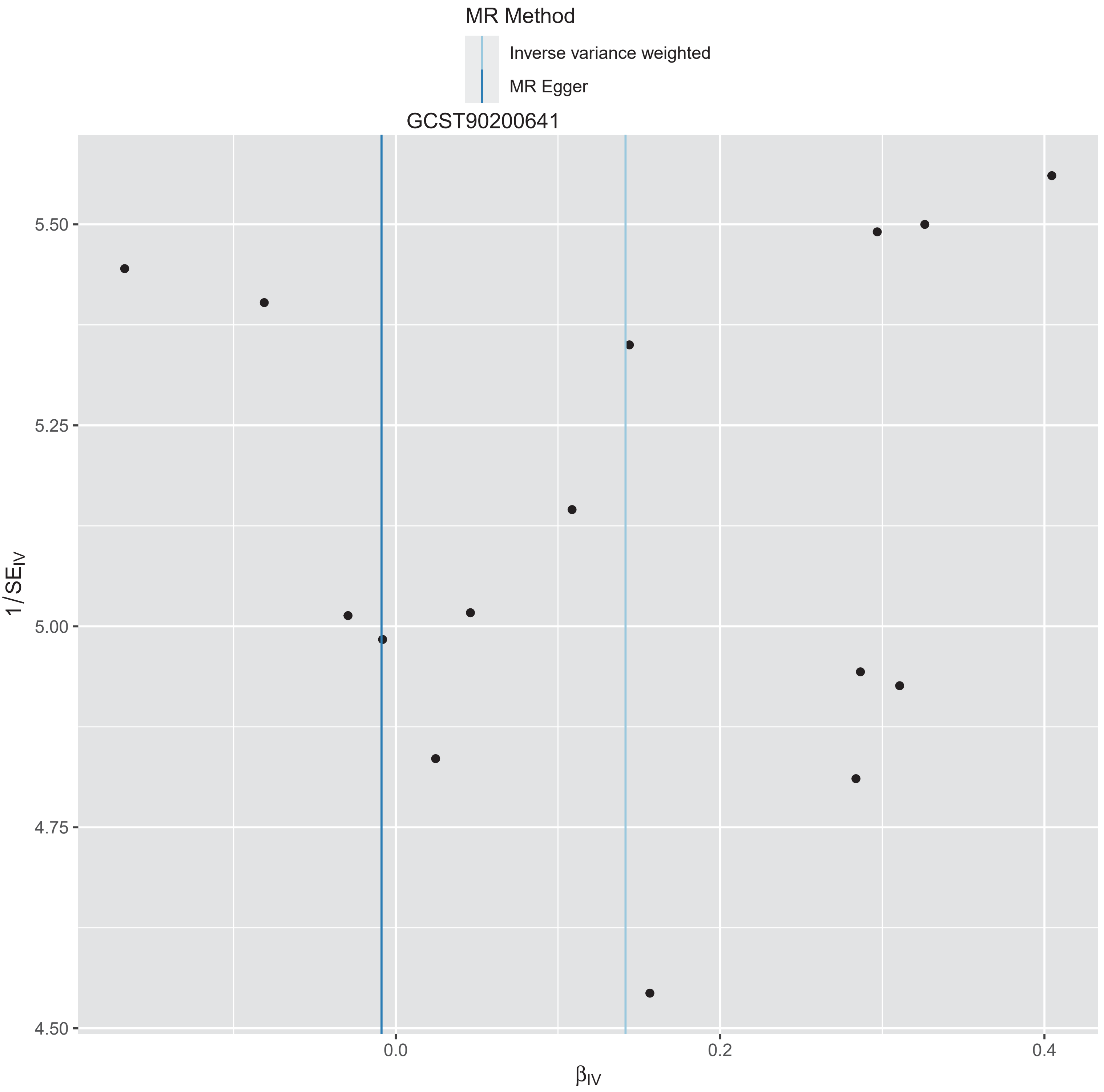

MR Method
Inverse variance weighted
MR Egger
GCST90200641
5.50
5.25
5.00
4.75
4.50
1/SE_IV
0.0
0.2
0.4
β_IV

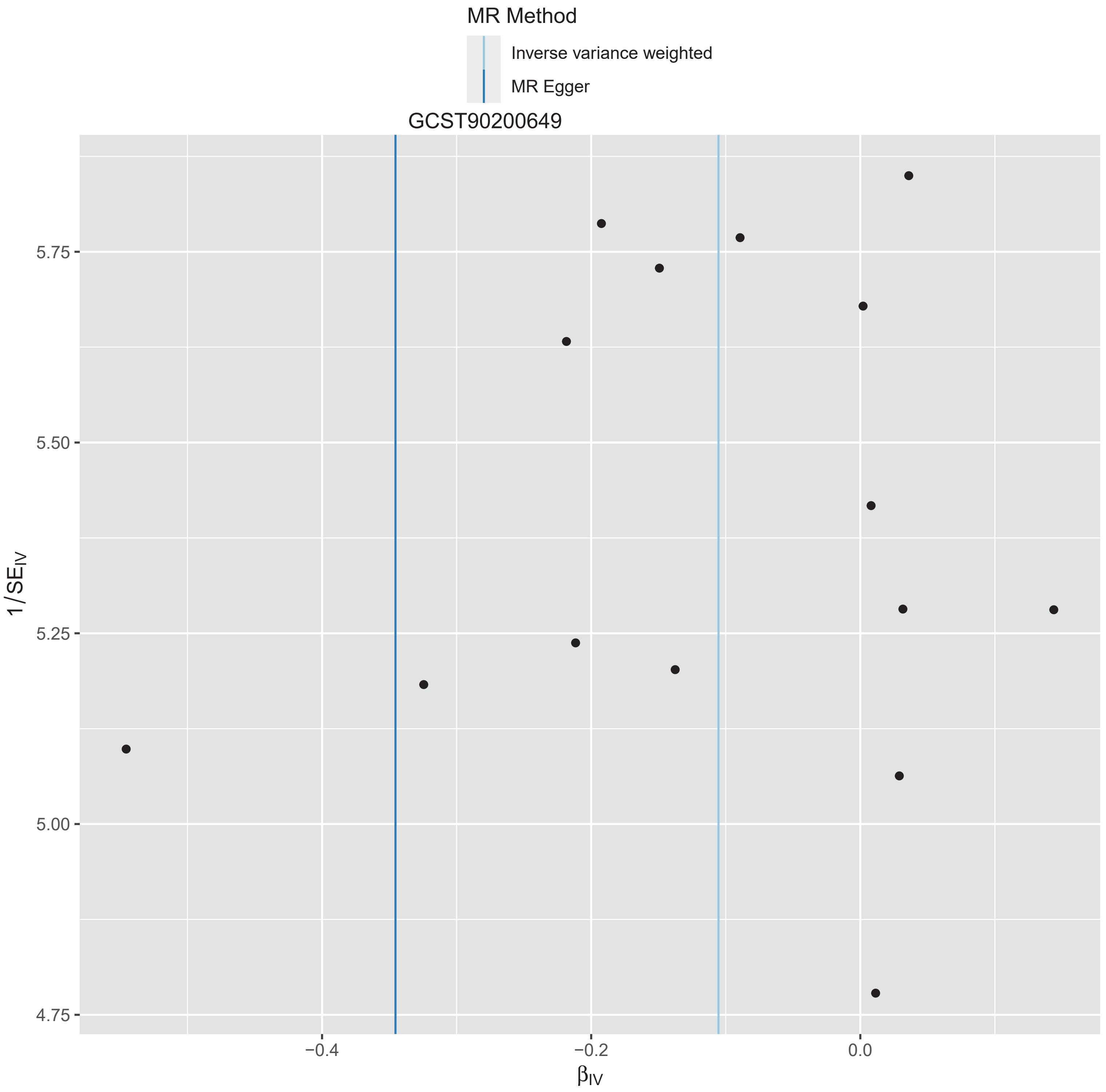

MR Method
Inverse variance weighted
MR Egger
GCST90200649
5.75
5.50
5.25
5.00
4.75
1/SE_IV
−0.4
−0.2
0.0
β_IV

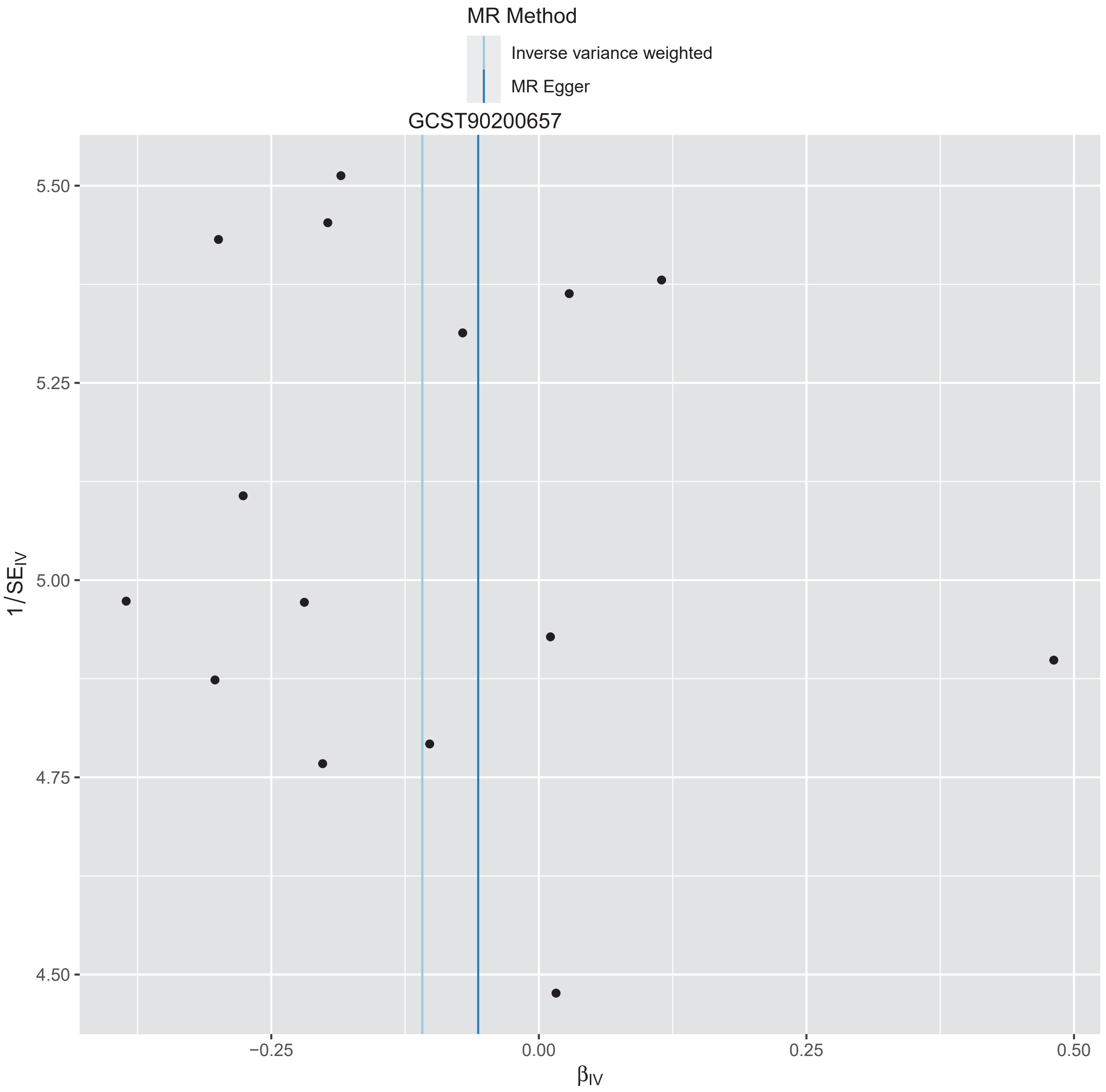

MR Method
Inverse variance weighted
MR Egger
GCST90200657
5.50
5.25
5.00
4.75
4.50
1/SE$_{IV}$
−0.25
0.00
0.25
0.50
β$_{IV}$

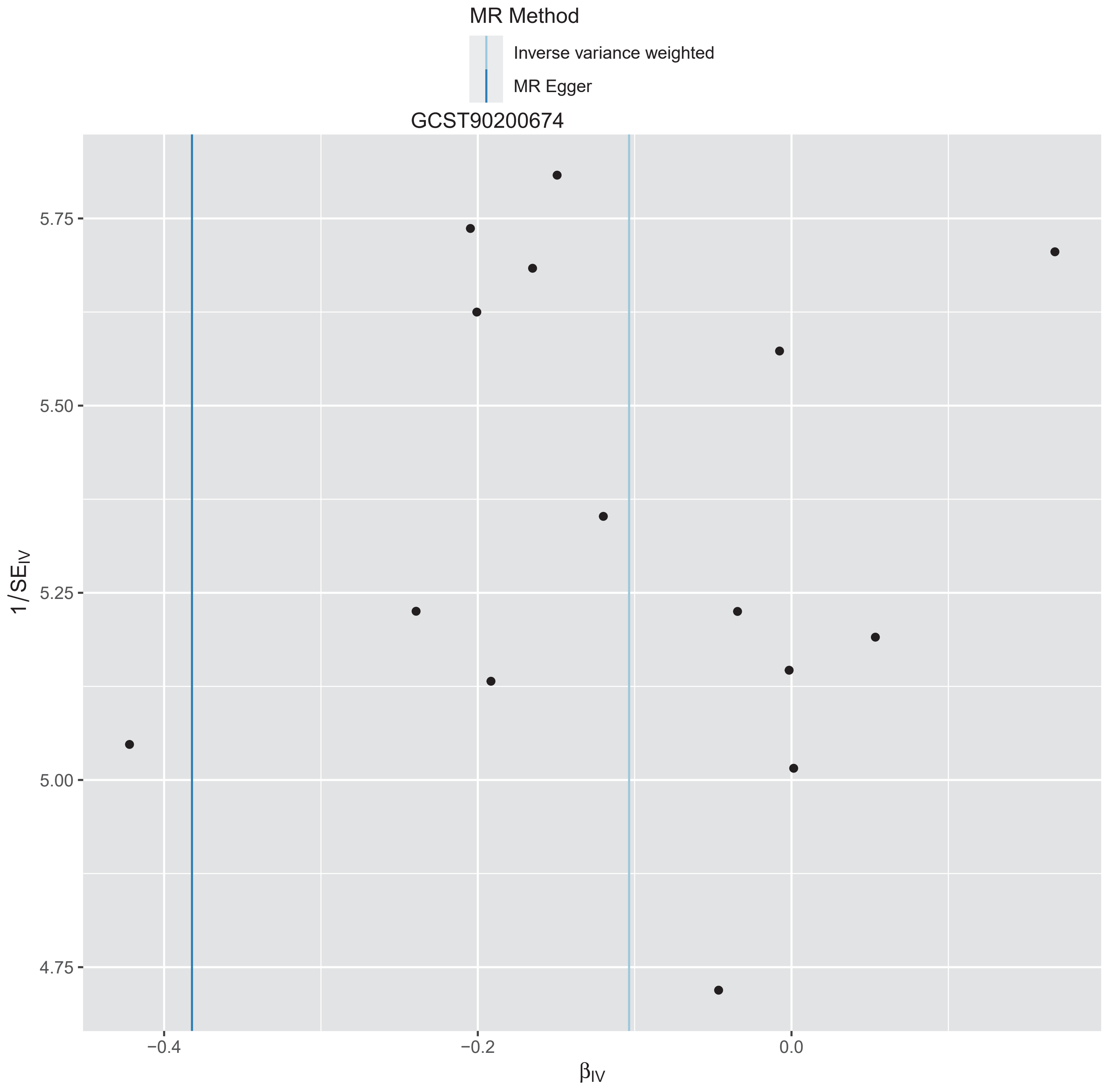

MR Method
Inverse variance weighted
MR Egger
GCST90200674
5.75
5.50
5.25
5.00
4.75
1/SE$_{IV}$
−0.4
−0.2
0.0
β_{IV}

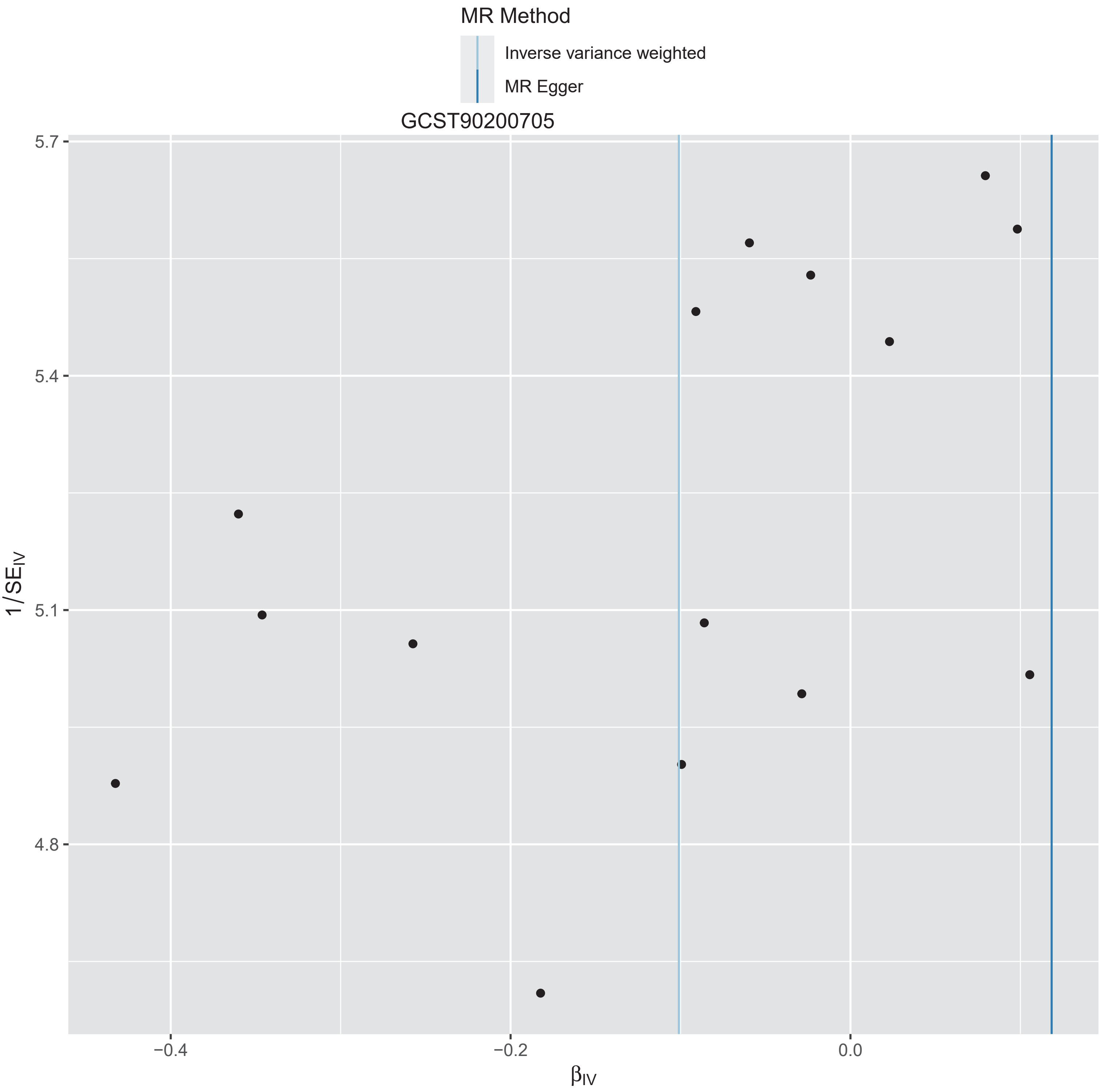
MR Method
Inverse variance weighted
MR Egger
GCST90200705
5.7
5.4
5.1
4.8
1/SE$_{IV}$
−0.4
−0.2
0.0
β$_{IV}$
