# Supplementary material for: Dissecting Causal Relationships Between Gut Microbiota, Plasma Metabolites and Bladder Cancer: A Two‐Step Mendelian Randomization Study
Source: Health Sci Rep. 2025 Sep 9;8(9):e71206. doi: 10.1002/hsr2.71206 (PMC12420358; doi:10.1002/hsr2.71206)
Supplement: Supplementary file 6 — Figure S6: Leave‐one‐out analysis for MR causal effects of plasma metabolites on s_Bacteroides_dorei. [file HSR2-8-e71206-s009.pdf]

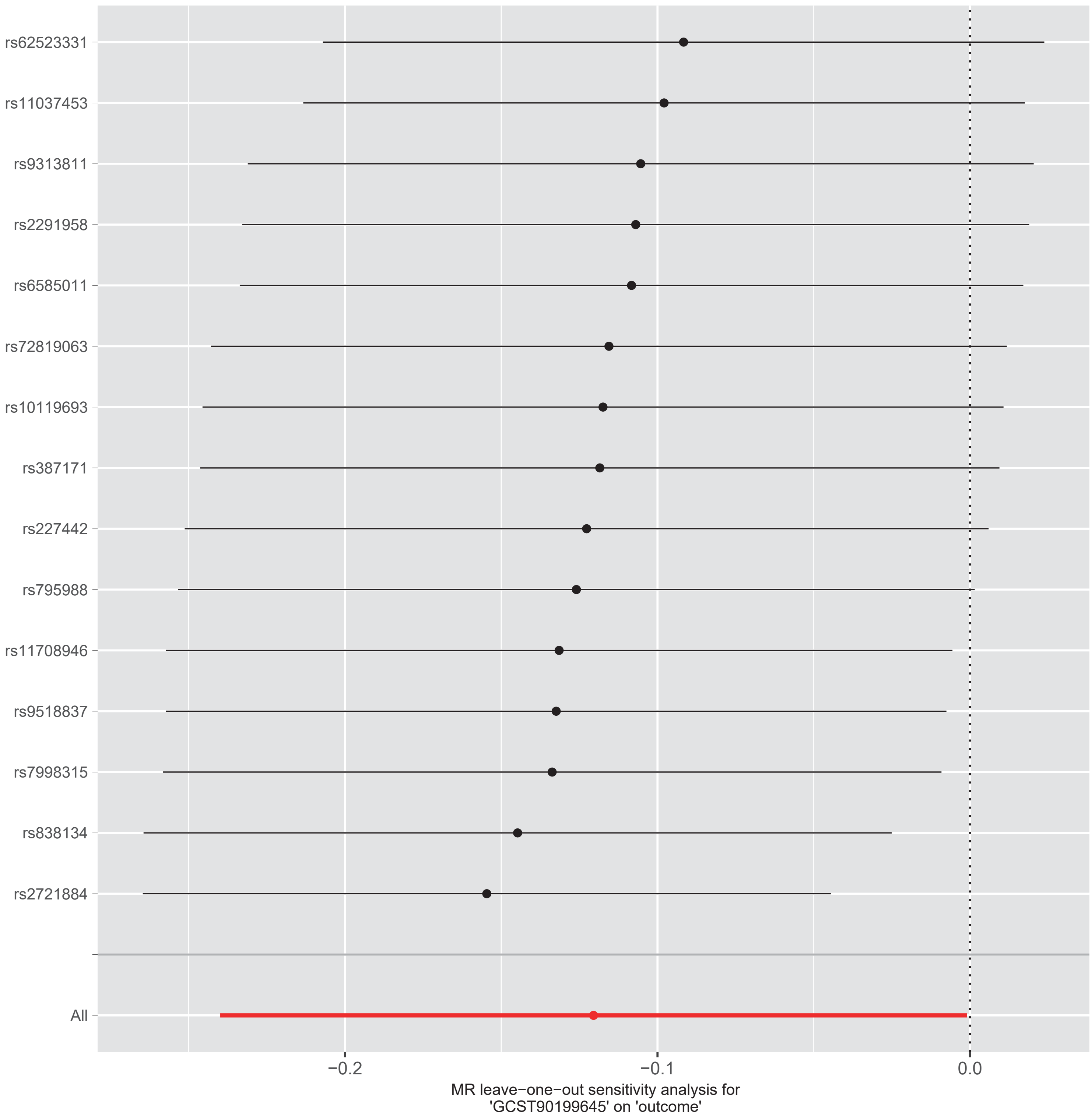
rs62523331
rs11037453
rs9313811
rs2291958
rs6585011
rs72819063
rs10119693
rs387171
rs227442
rs795988
rs11708946
rs9518837
rs7998315
rs838134
rs2721884
All
−0.2
−0.1
0.0
MR leave−one−out sensitivity analysis for
'GCST90199645' on 'outcome'

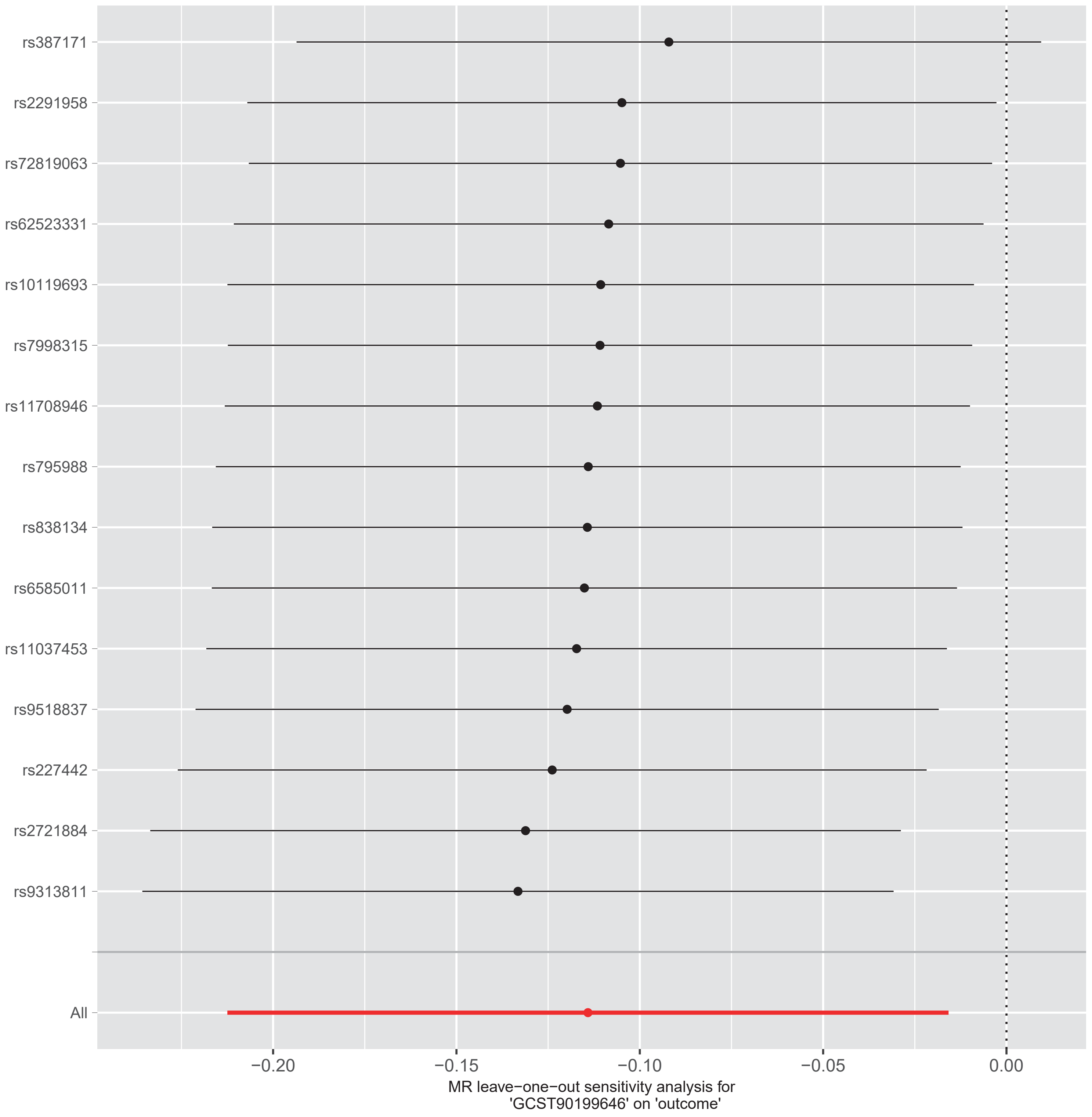
rs387171
rs2291958
rs72819063
rs62523331
rs10119693
rs7998315
rs11708946
rs795988
rs838134
rs6585011
rs11037453
rs9518837
rs227442
rs2721884
rs9313811
All
−0.20
−0.15
−0.10
−0.05
0.00
MR leave−one−out sensitivity analysis for
'GCST90199646' on 'outcome'

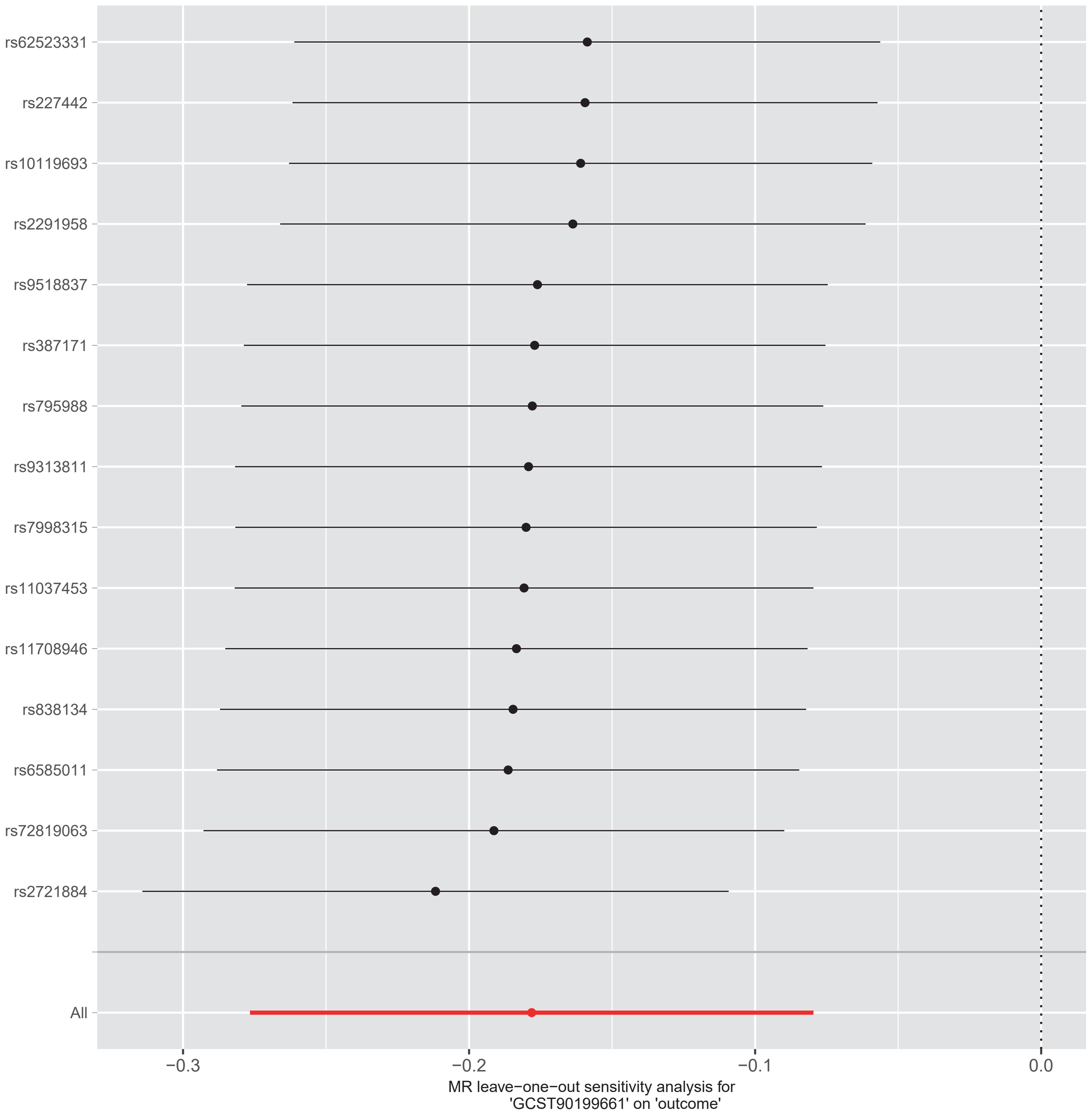
rs62523331
rs227442
rs10119693
rs2291958
rs9518837
rs387171
rs795988
rs9313811
rs7998315
rs11037453
rs11708946
rs838134
rs6585011
rs72819063
rs2721884
All
−0.3
−0.2
−0.1
0.0
MR leave−one−out sensitivity analysis for
'GCST90199661' on 'outcome'

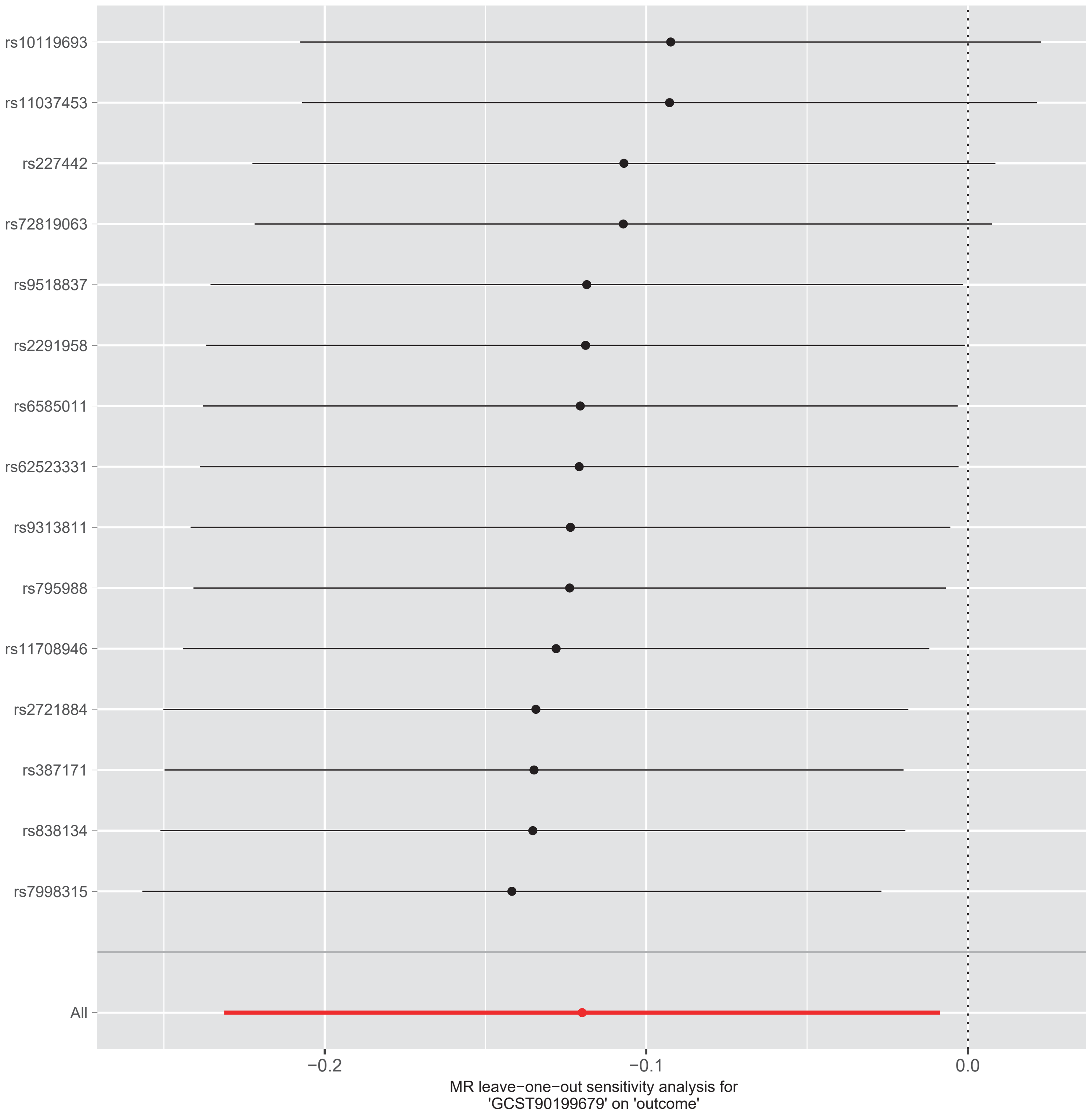
rs10119693
rs11037453
rs227442
rs72819063
rs9518837
rs2291958
rs6585011
rs62523331
rs9313811
rs795988
rs11708946
rs2721884
rs387171
rs838134
rs7998315
All
−0.2
−0.1
0.0
MR leave−one−out sensitivity analysis for
'GCST90199679' on 'outcome'

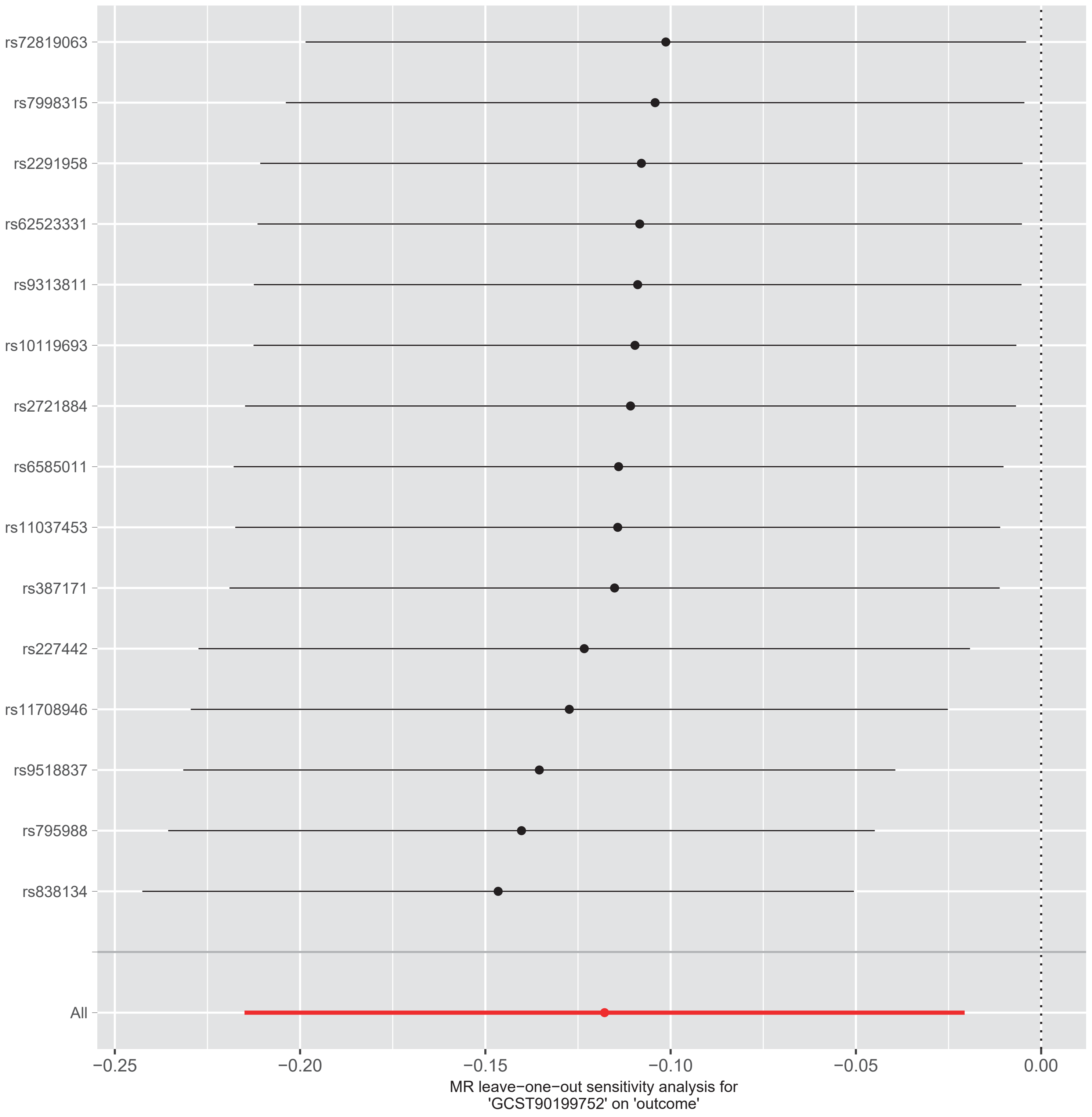
rs72819063
rs7998315
rs2291958
rs62523331
rs9313811
rs10119693
rs2721884
rs6585011
rs11037453
rs387171
rs227442
rs11708946
rs9518837
rs795988
rs838134
All
−0.25
−0.20
−0.15
−0.10
−0.05
0.00
MR leave−one−out sensitivity analysis for
'GCST90199752' on 'outcome'

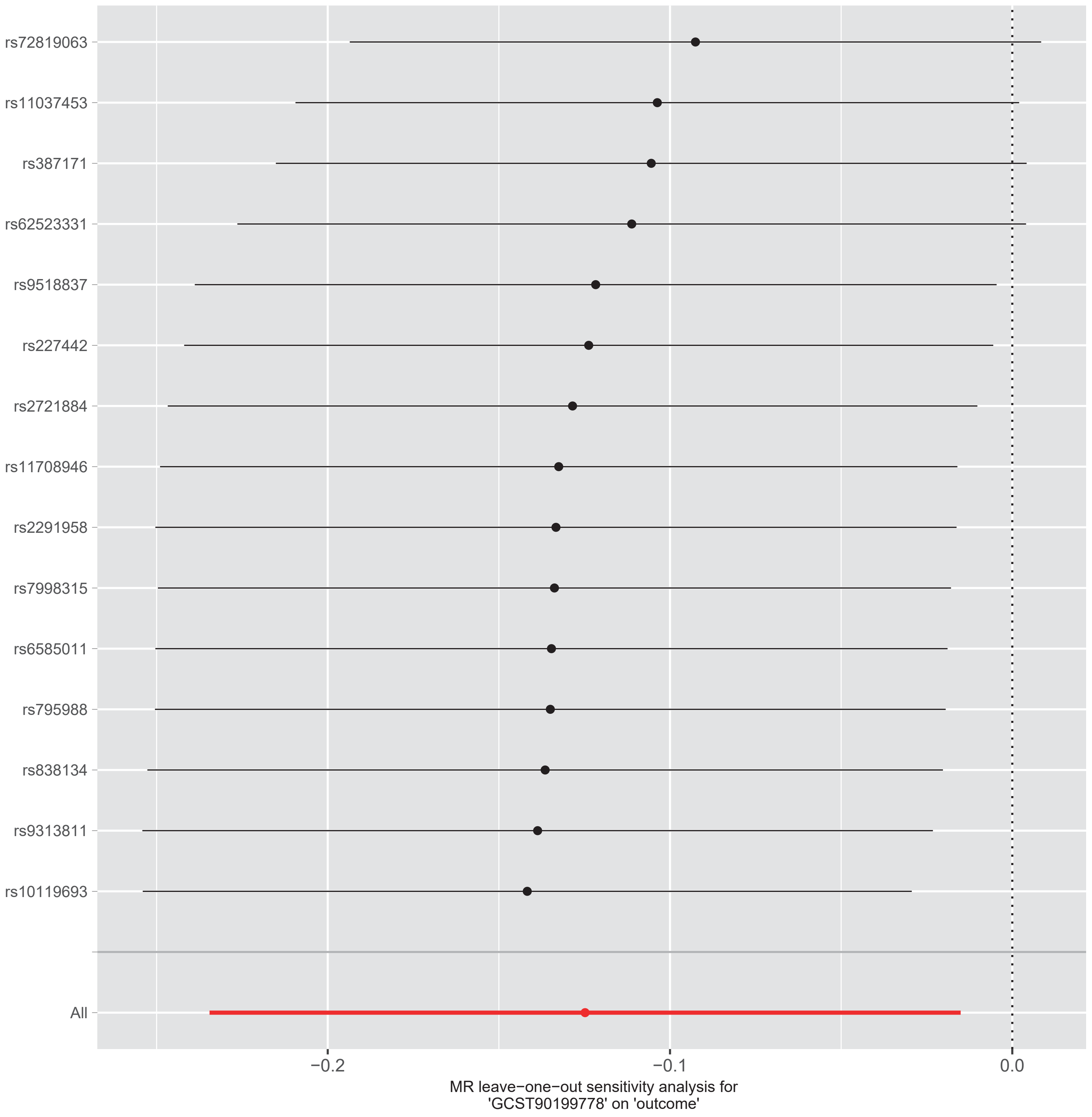
rs72819063
rs11037453
rs387171
rs62523331
rs9518837
rs227442
rs2721884
rs11708946
rs2291958
rs7998315
rs6585011
rs795988
rs838134
rs9313811
rs10119693
All
−0.2
−0.1
0.0
MR leave−one−out sensitivity analysis for
'GCST90199778' on 'outcome'

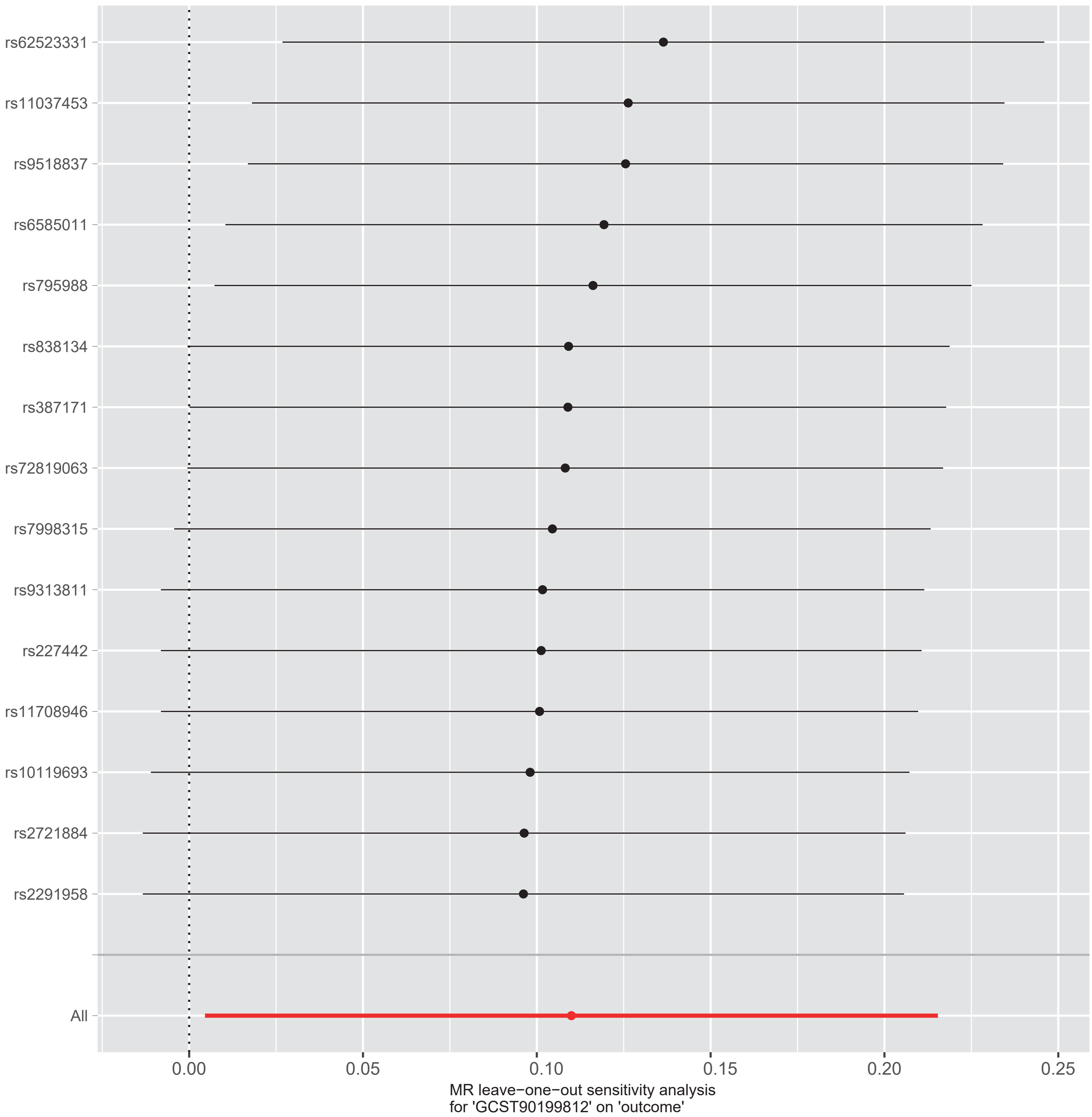
rs62523331
rs11037453
rs9518837
rs6585011
rs795988
rs838134
rs387171
rs72819063
rs7998315
rs9313811
rs227442
rs11708946
rs10119693
rs2721884
rs2291958
All
0.00
0.05
0.10
0.15
0.20
0.25
MR leave−one−out sensitivity analysis
for 'GCST90199812' on 'outcome'

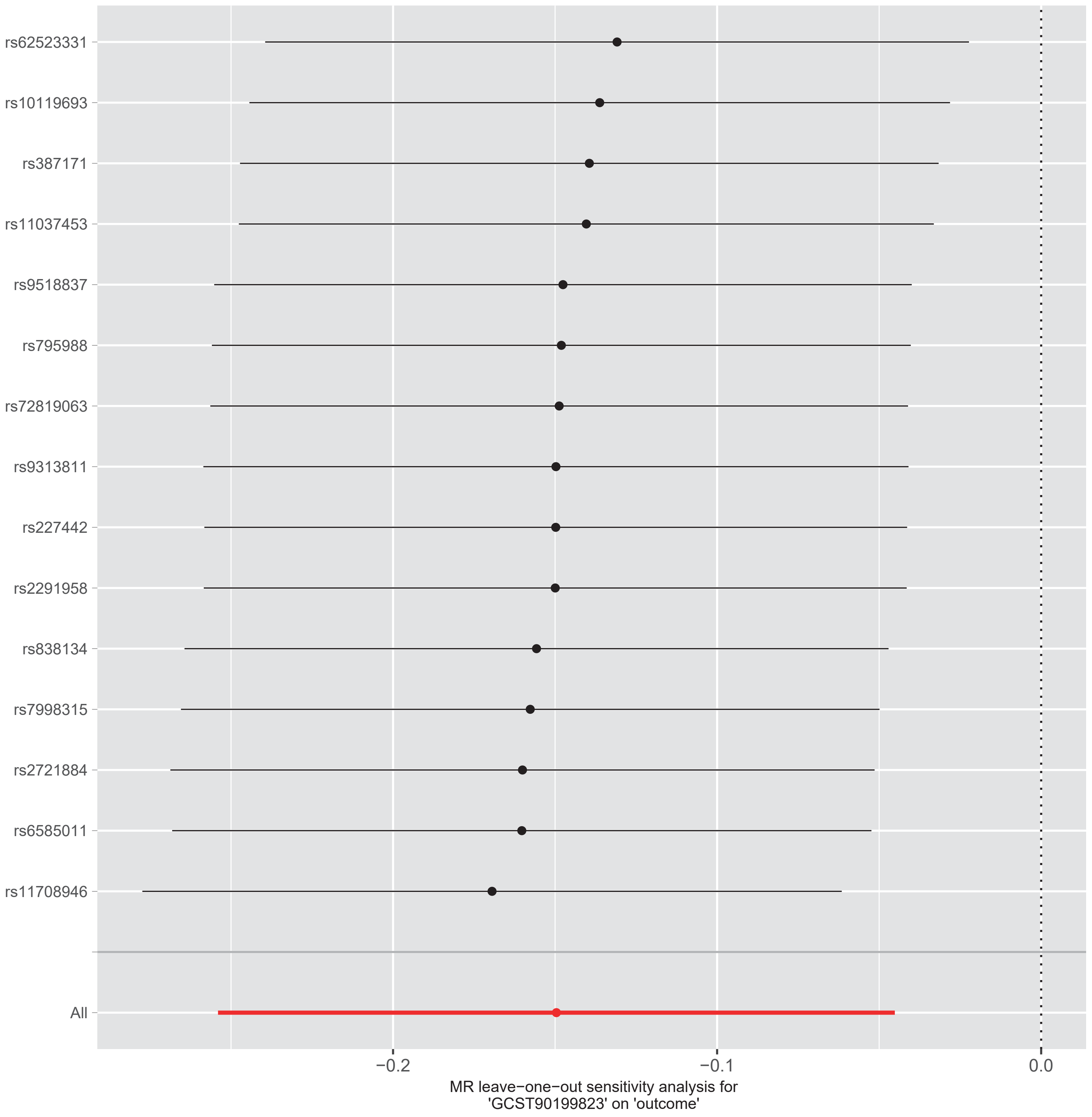
rs62523331
rs10119693
rs387171
rs11037453
rs9518837
rs795988
rs72819063
rs9313811
rs227442
rs2291958
rs838134
rs7998315
rs2721884
rs6585011
rs11708946
All
−0.2
−0.1
0.0
MR leave−one−out sensitivity analysis for
'GCST90199823' on 'outcome'

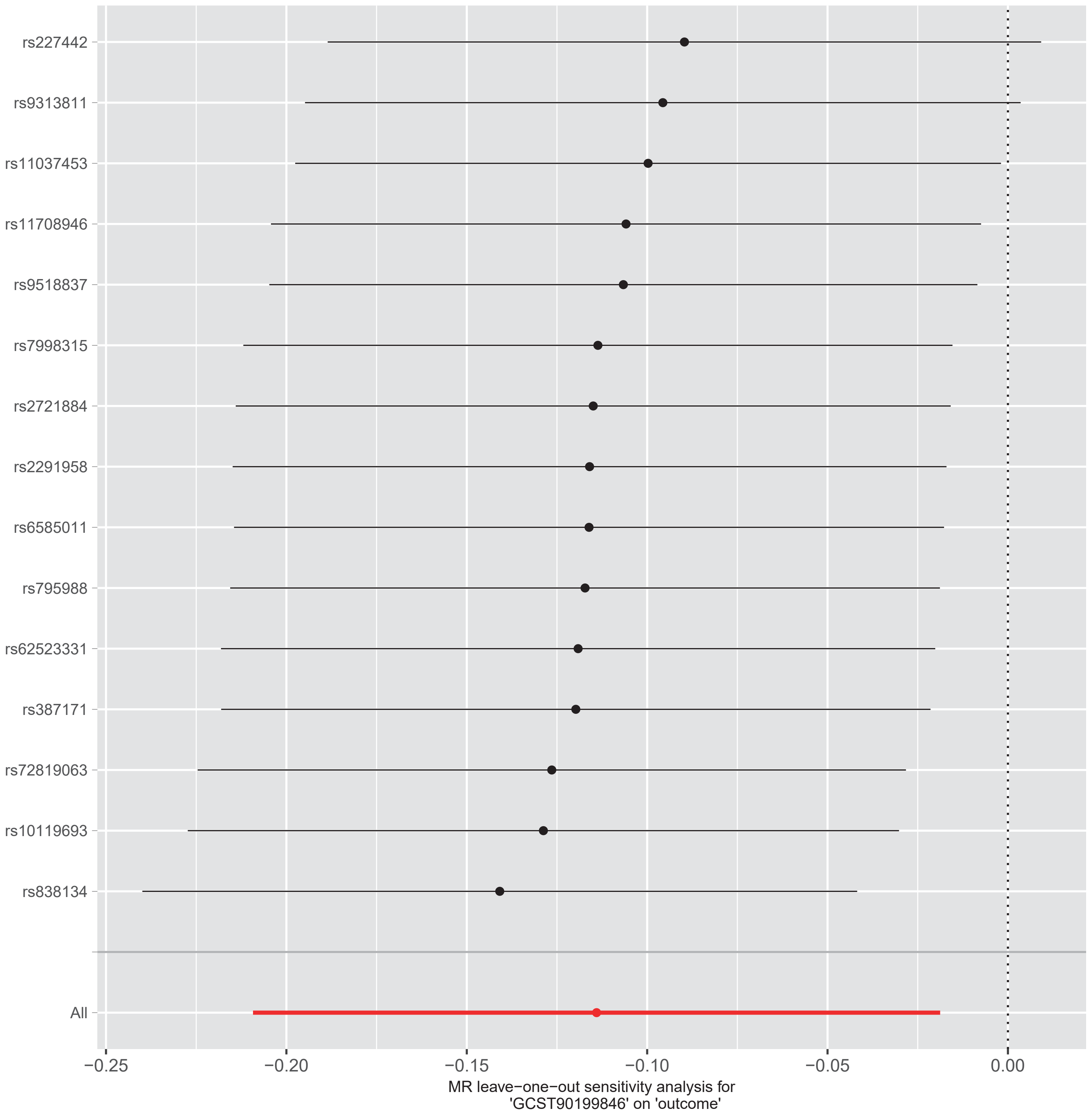

rs227442
rs9313811
rs11037453
rs11708946
rs9518837
rs7998315
rs2721884
rs2291958
rs6585011
rs795988
rs62523331
rs387171
rs72819063
rs10119693
rs838134
All
−0.25
−0.20
−0.15
−0.10
−0.05
0.00
MR leave−one−out sensitivity analysis for
'GCST90199846' on 'outcome'

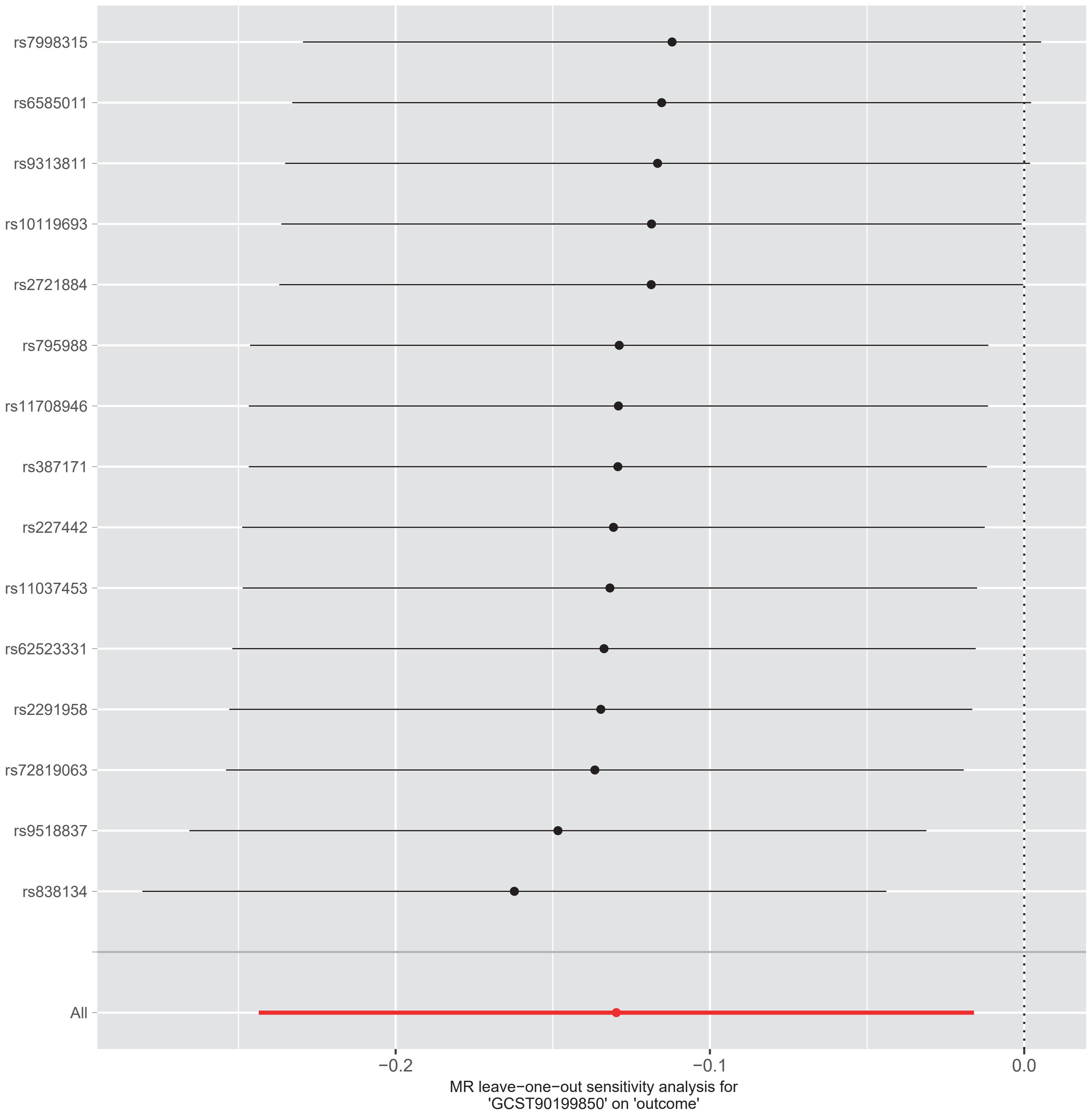
rs7998315
rs6585011
rs9313811
rs10119693
rs2721884
rs795988
rs11708946
rs387171
rs227442
rs11037453
rs62523331
rs2291958
rs72819063
rs9518837
rs838134
All
−0.2
−0.1
0.0
MR leave−one−out sensitivity analysis for
'GCST90199850' on 'outcome'

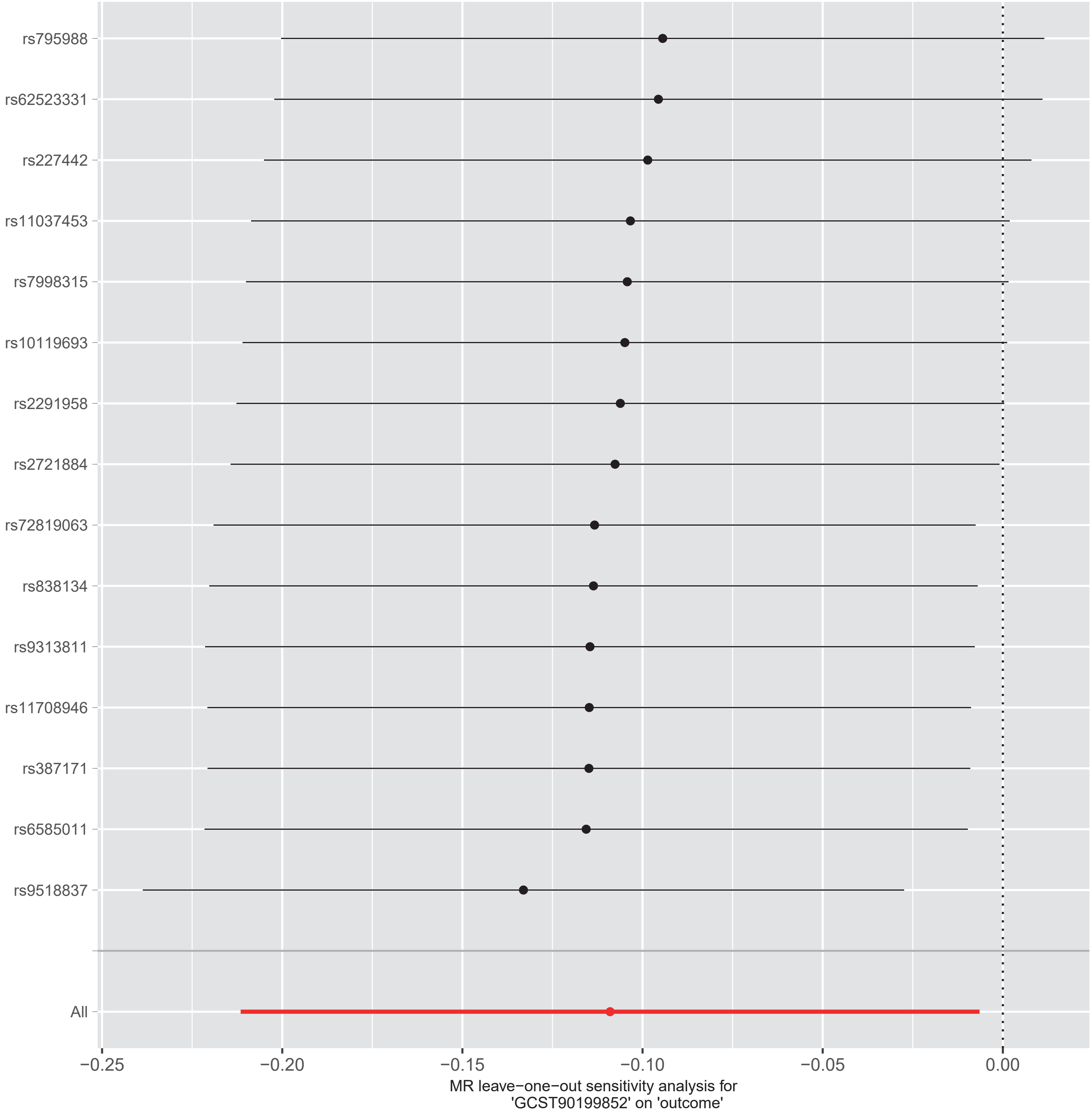
rs795988
rs62523331
rs227442
rs11037453
rs7998315
rs10119693
rs2291958
rs2721884
rs72819063
rs838134
rs9313811
rs11708946
rs387171
rs6585011
rs9518837
All
−0.25
−0.20
−0.15
−0.10
−0.05
0.00
MR leave−one−out sensitivity analysis for
'GCST90199852' on 'outcome'

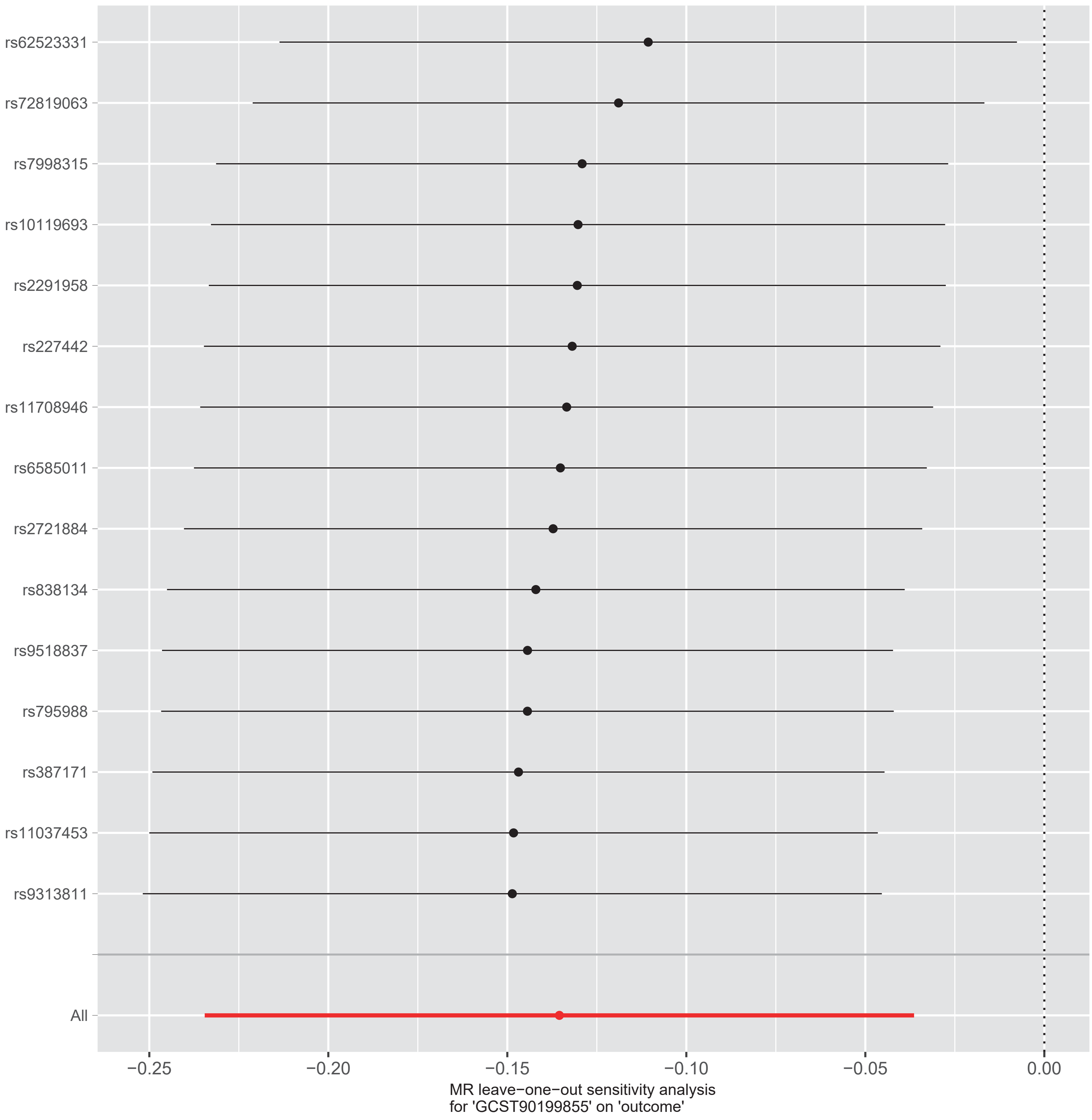
rs62523331
rs72819063
rs7998315
rs10119693
rs2291958
rs227442
rs11708946
rs6585011
rs2721884
rs838134
rs9518837
rs795988
rs387171
rs11037453
rs9313811
All
−0.25
−0.20
−0.15
−0.10
−0.05
0.00
MR leave−one−out sensitivity analysis
for 'GCST90199855' on 'outcome'

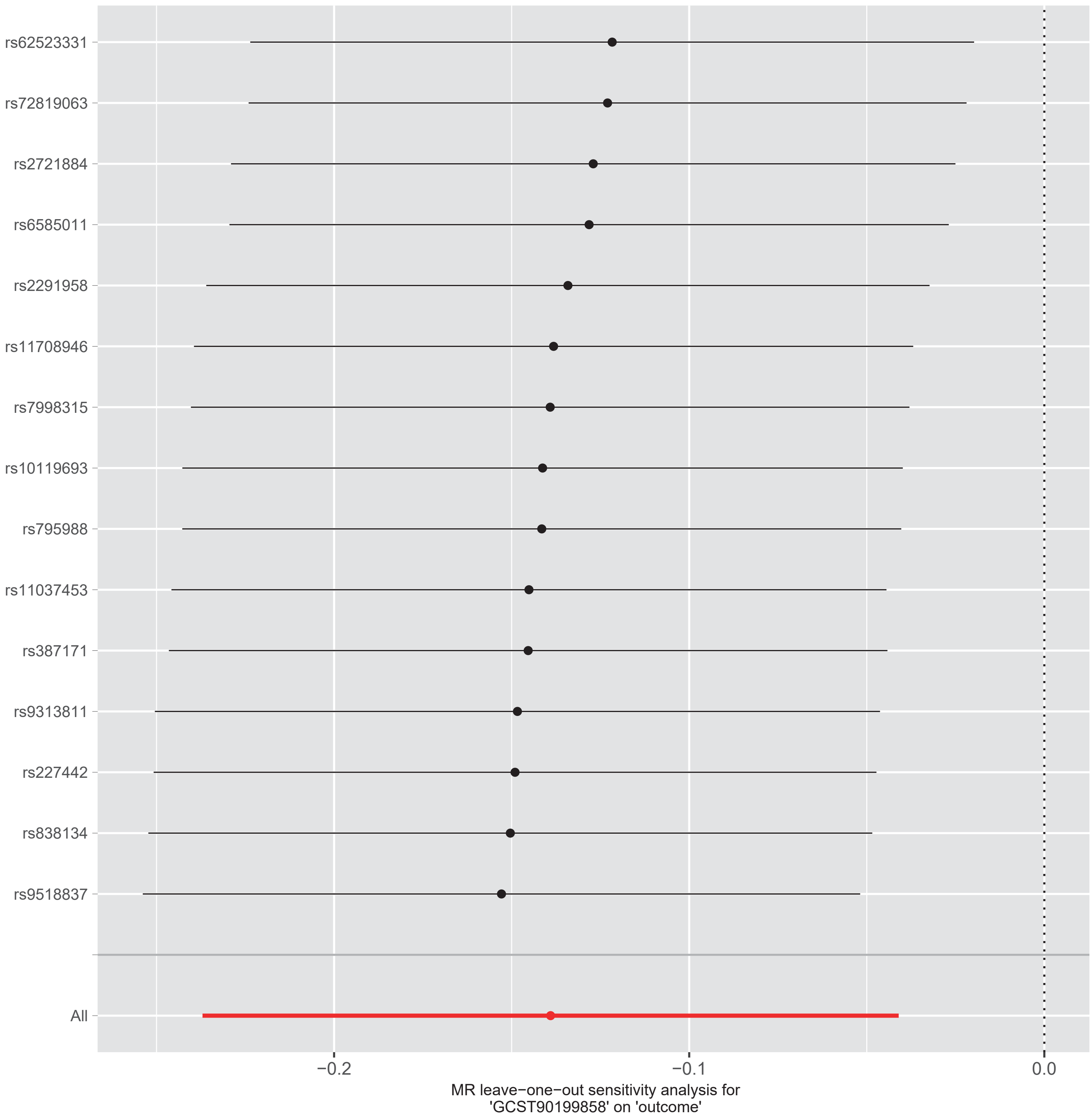
rs62523331
rs72819063
rs2721884
rs6585011
rs2291958
rs11708946
rs7998315
rs10119693
rs795988
rs11037453
rs387171
rs9313811
rs227442
rs838134
rs9518837
All
−0.2
−0.1
0.0
MR leave−one−out sensitivity analysis for
'GCST90199858' on 'outcome'

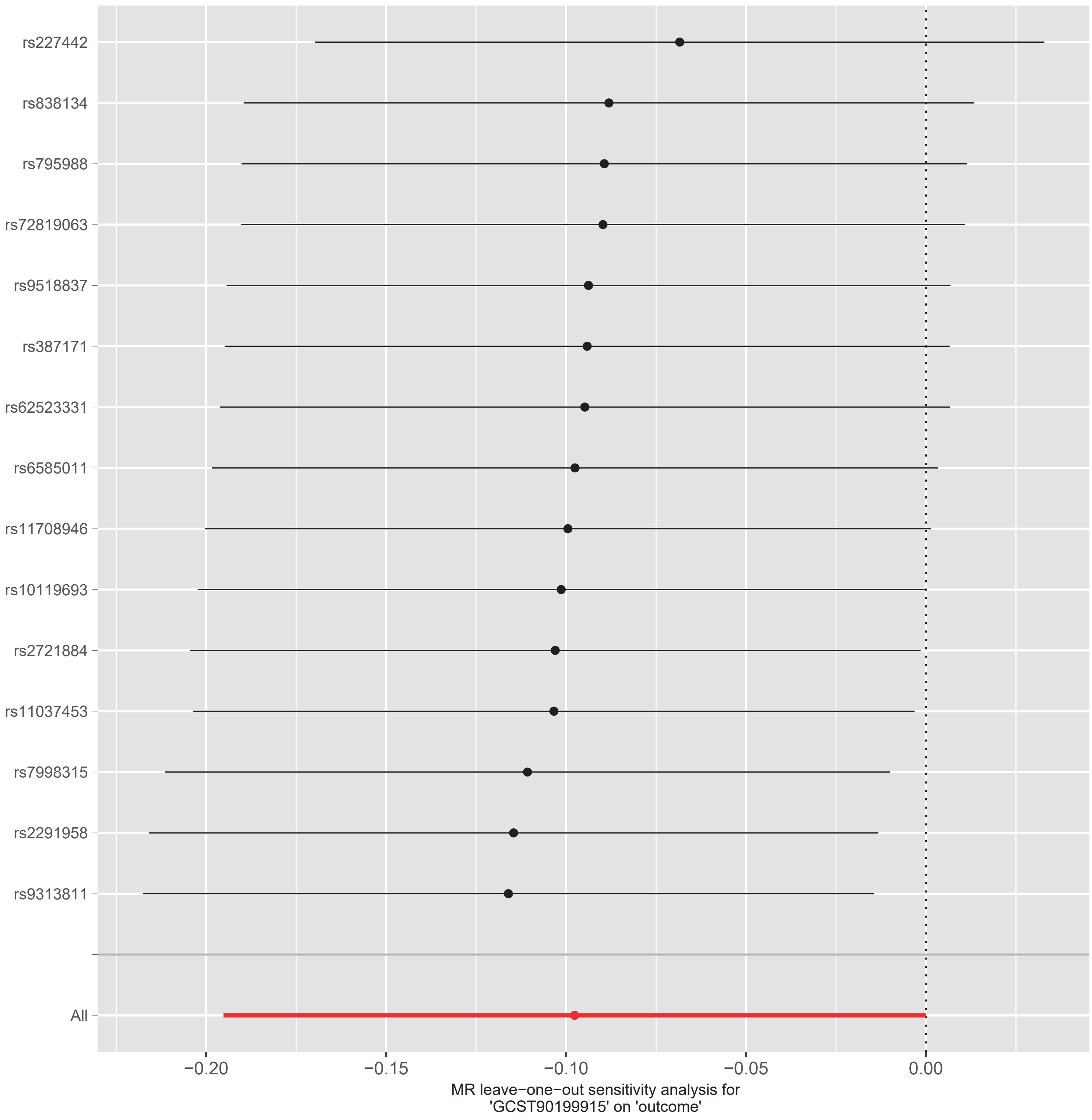
rs227442
rs838134
rs795988
rs72819063
rs9518837
rs387171
rs62523331
rs6585011
rs11708946
rs10119693
rs2721884
rs11037453
rs7998315
rs2291958
rs9313811
All
−0.20
−0.15
−0.10
−0.05
0.00
MR leave−one−out sensitivity analysis for
'GCST90199915' on 'outcome'

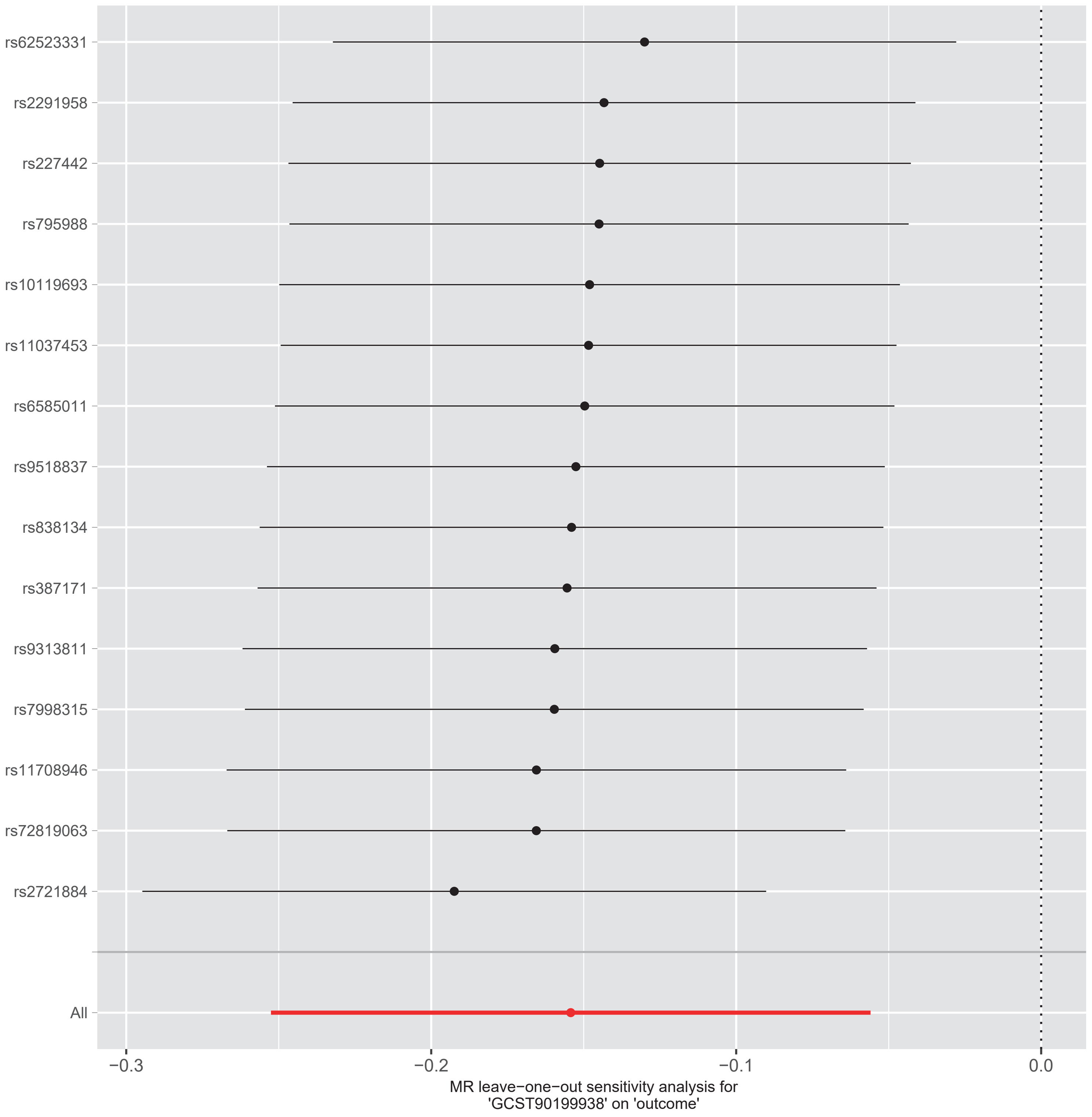

rs62523331
rs2291958
rs227442
rs795988
rs10119693
rs11037453
rs6585011
rs9518837
rs838134
rs387171
rs9313811
rs7998315
rs11708946
rs72819063
rs2721884
All
−0.3
−0.2
−0.1
0.0
MR leave−one−out sensitivity analysis for
'GCST90199938' on 'outcome'

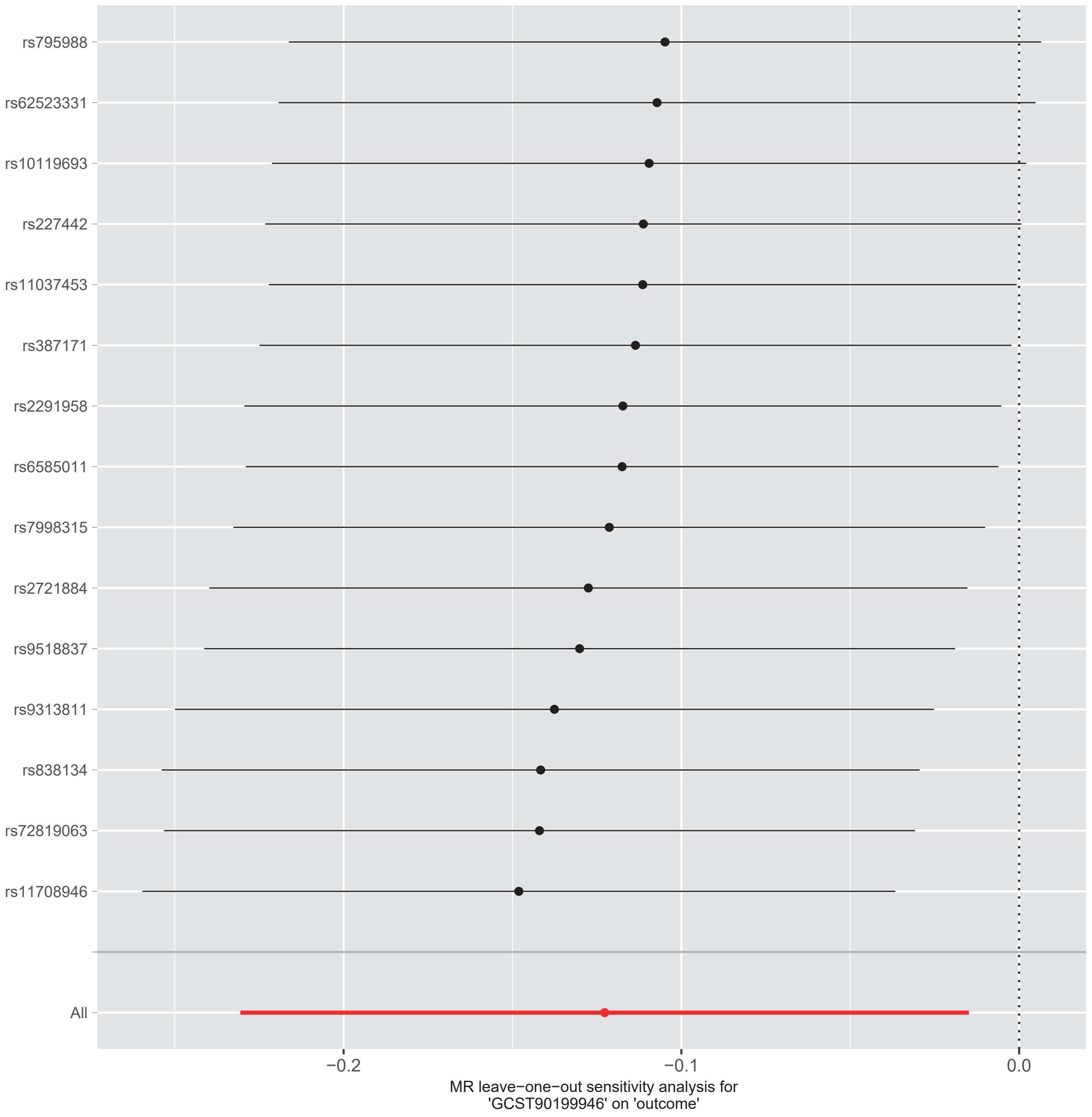
rs795988
rs62523331
rs10119693
rs227442
rs11037453
rs387171
rs2291958
rs6585011
rs7998315
rs2721884
rs9518837
rs9313811
rs838134
rs72819063
rs11708946
All
−0.2
−0.1
0.0
MR leave−one−out sensitivity analysis for
'GCST90199946' on 'outcome'

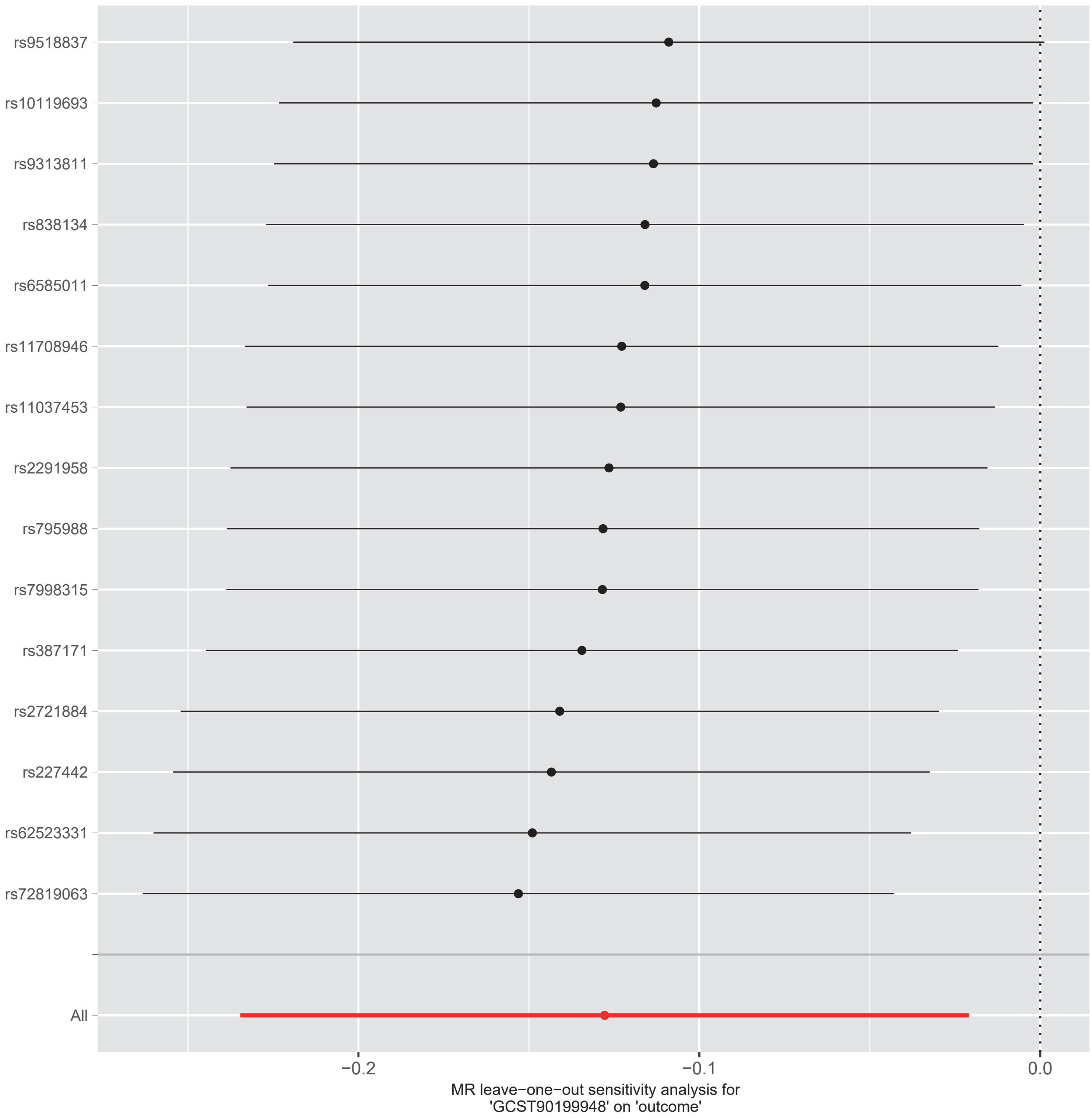
rs9518837
rs10119693
rs9313811
rs838134
rs6585011
rs11708946
rs11037453
rs2291958
rs795988
rs7998315
rs387171
rs2721884
rs227442
rs62523331
rs72819063
All
−0.2
−0.1
0.0
MR leave−one−out sensitivity analysis for
'GCST90199948' on 'outcome'

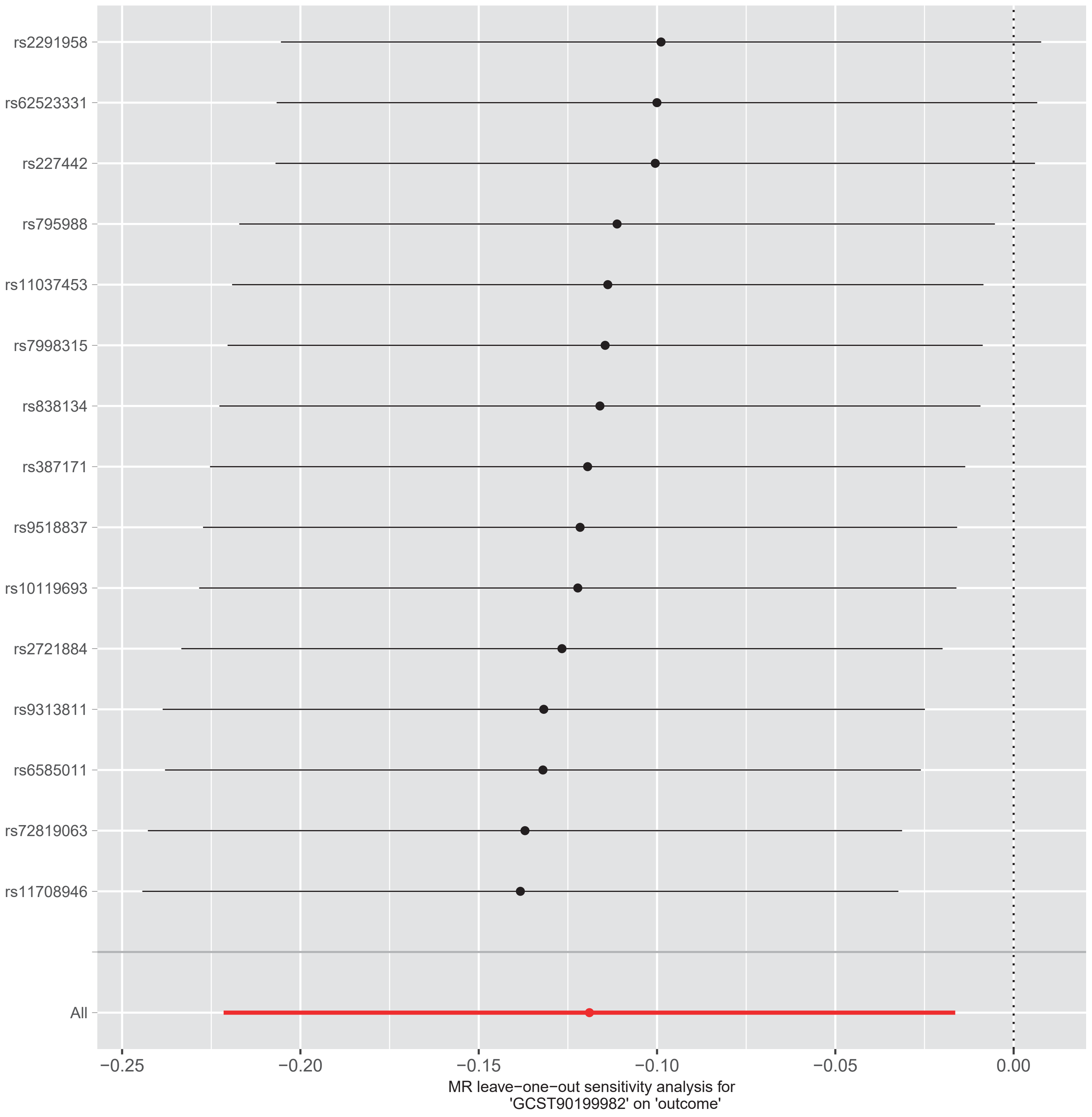
rs2291958
rs62523331
rs227442
rs795988
rs11037453
rs7998315
rs838134
rs387171
rs9518837
rs10119693
rs2721884
rs9313811
rs6585011
rs72819063
rs11708946
All
−0.25
−0.20
−0.15
−0.10
−0.05
0.00
MR leave−one−out sensitivity analysis for
'GCST90199982' on 'outcome'

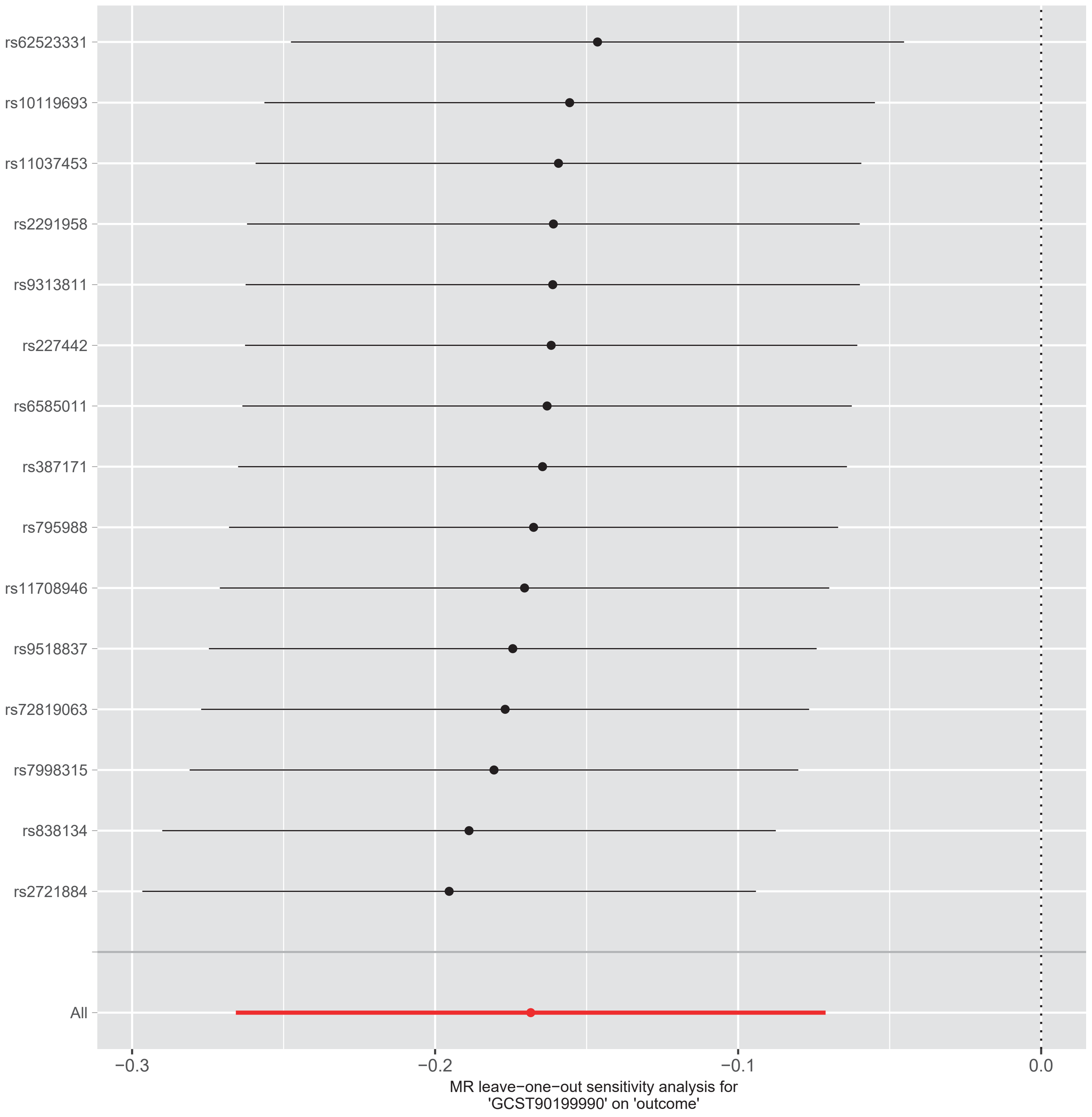
rs62523331
rs10119693
rs11037453
rs2291958
rs9313811
rs227442
rs6585011
rs387171
rs795988
rs11708946
rs9518837
rs72819063
rs7998315
rs838134
rs2721884
All
−0.3
−0.2
−0.1
0.0
MR leave−one−out sensitivity analysis for
'GCST90199990' on 'outcome'

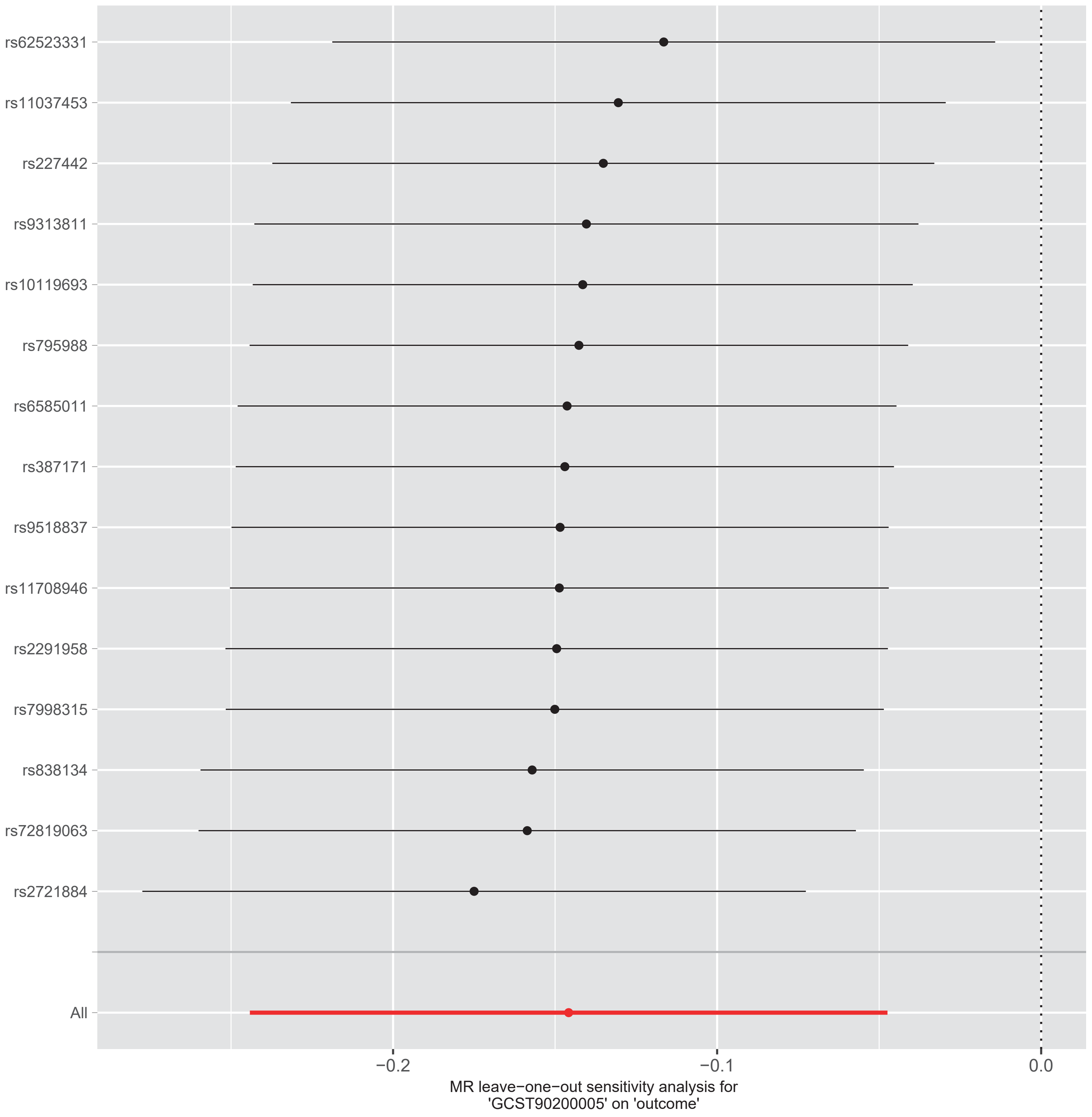
rs62523331
rs11037453
rs227442
rs9313811
rs10119693
rs795988
rs6585011
rs387171
rs9518837
rs11708946
rs2291958
rs7998315
rs838134
rs72819063
rs2721884
All
−0.2
−0.1
0.0
MR leave−one−out sensitivity analysis for
'GCST90200005' on 'outcome'

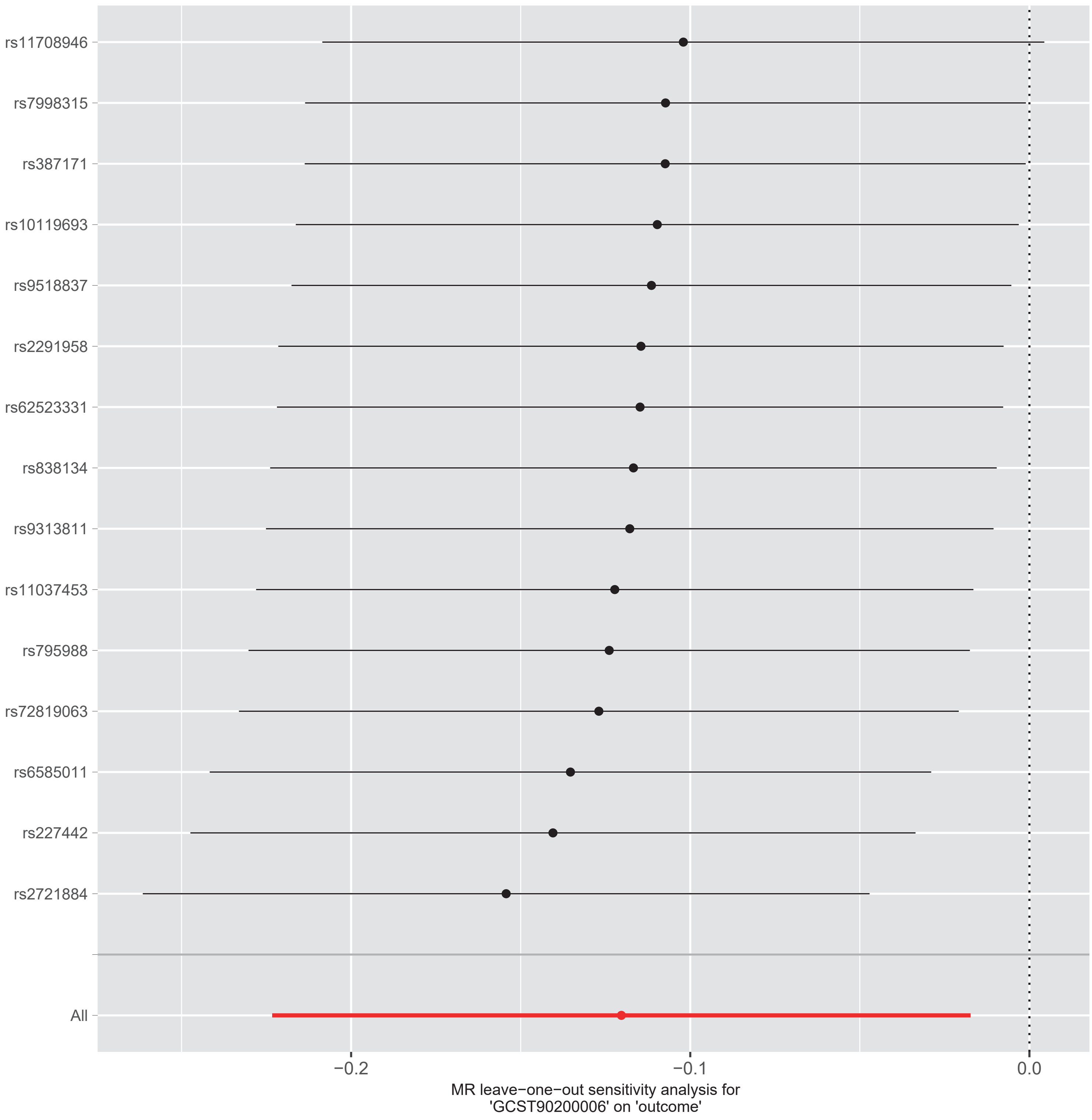
rs11708946
rs7998315
rs387171
rs10119693
rs9518837
rs2291958
rs62523331
rs838134
rs9313811
rs11037453
rs795988
rs72819063
rs6585011
rs227442
rs2721884
All
−0.2
−0.1
0.0
MR leave−one−out sensitivity analysis for
'GCST90200006' on 'outcome'

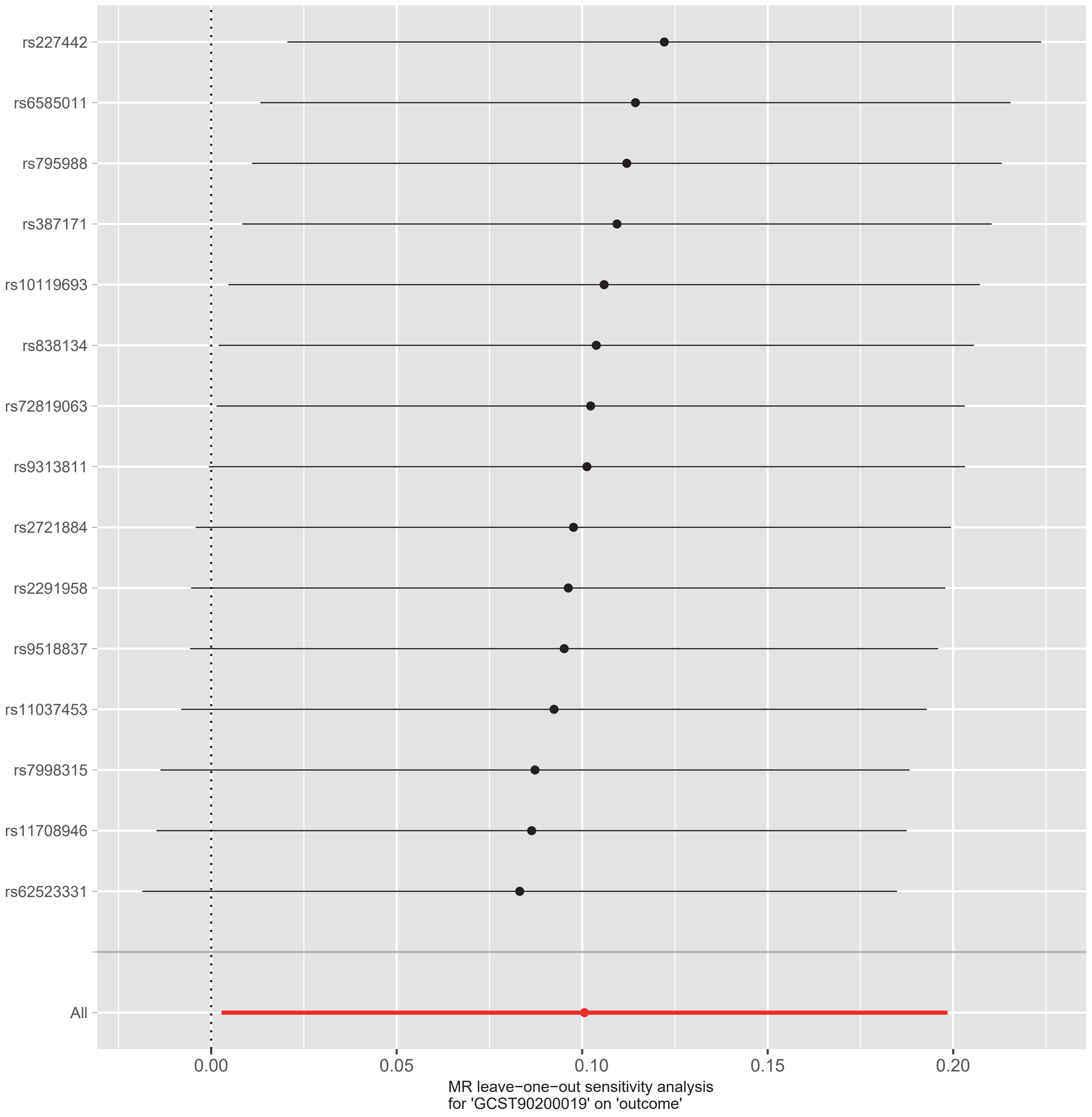

rs227442
rs6585011
rs795988
rs387171
rs10119693
rs838134
rs72819063
rs9313811
rs2721884
rs2291958
rs9518837
rs11037453
rs7998315
rs11708946
rs62523331
All
0.00
0.05
0.10
0.15
0.20
MR leave−one−out sensitivity analysis
for 'GCST90200019' on 'outcome'

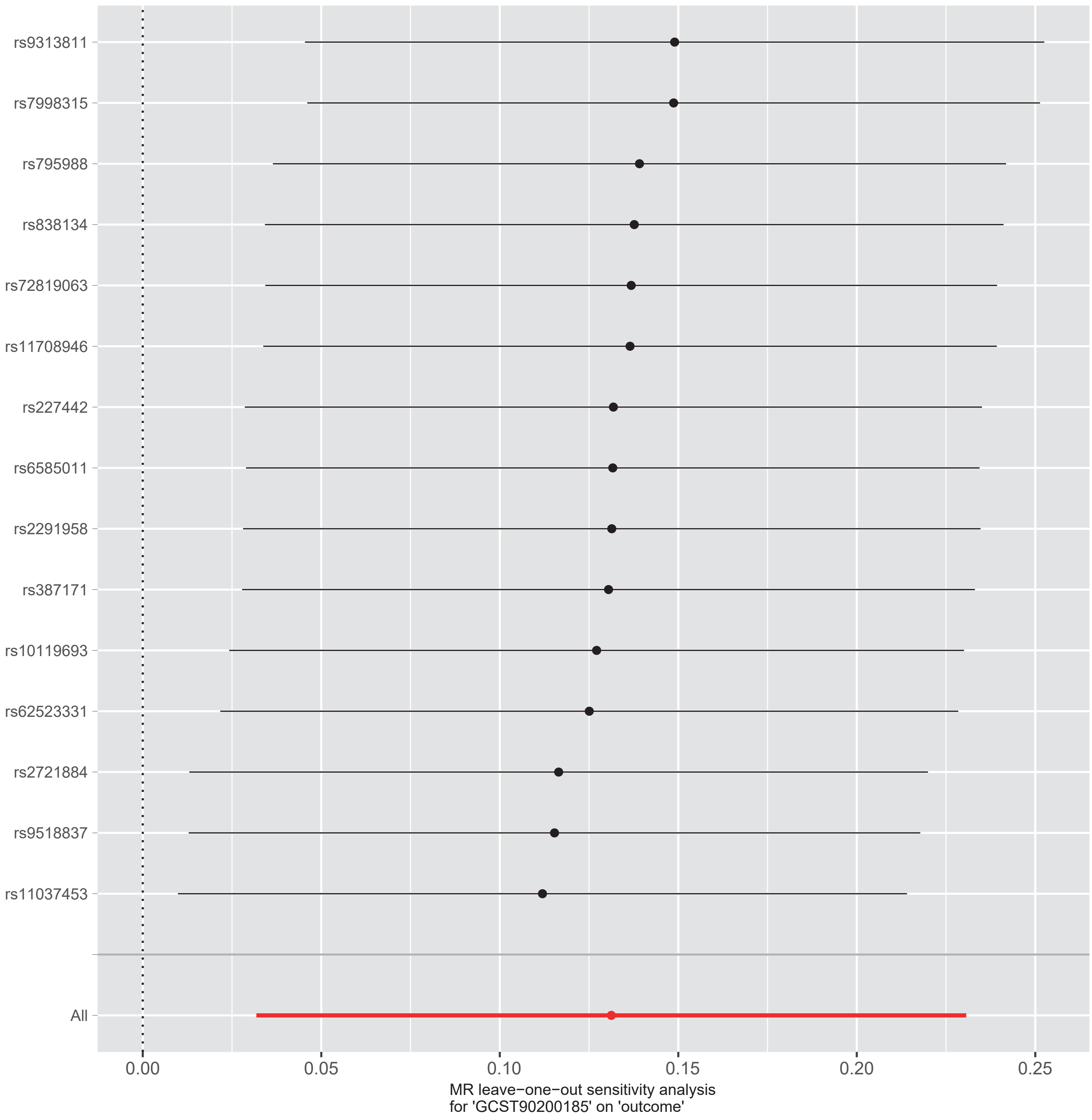

rs9313811
rs7998315
rs795988
rs838134
rs72819063
rs11708946
rs227442
rs6585011
rs2291958
rs387171
rs10119693
rs62523331
rs2721884
rs9518837
rs11037453
All
0.00
0.05
0.10
0.15
0.20
0.25
MR leave−one−out sensitivity analysis
for 'GCST90200185' on 'outcome'

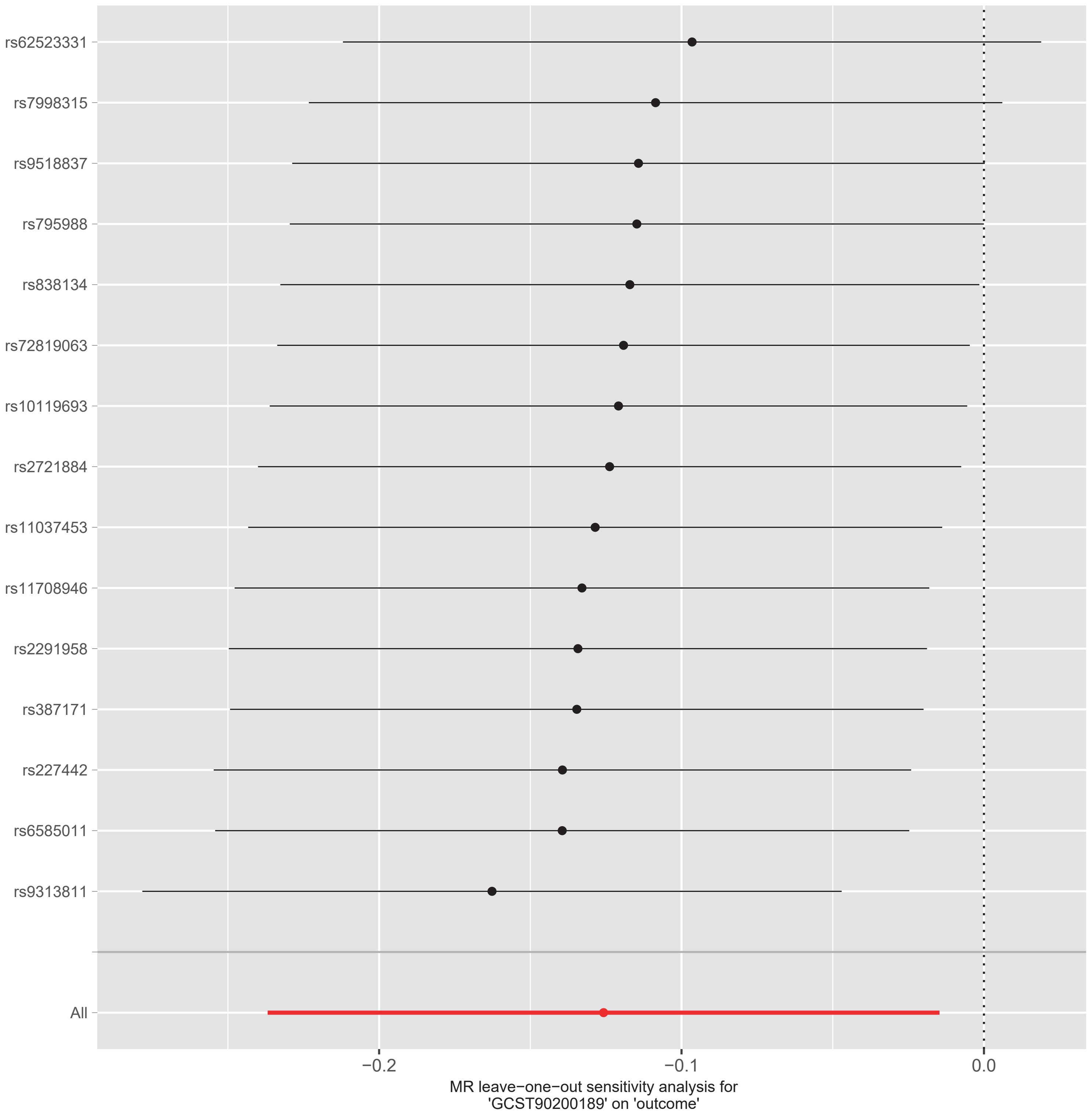

rs62523331
rs7998315
rs9518837
rs795988
rs838134
rs72819063
rs10119693
rs2721884
rs11037453
rs11708946
rs2291958
rs387171
rs227442
rs6585011
rs9313811
All
−0.2
−0.1
0.0
MR leave−one−out sensitivity analysis for
'GCST90200189' on 'outcome'

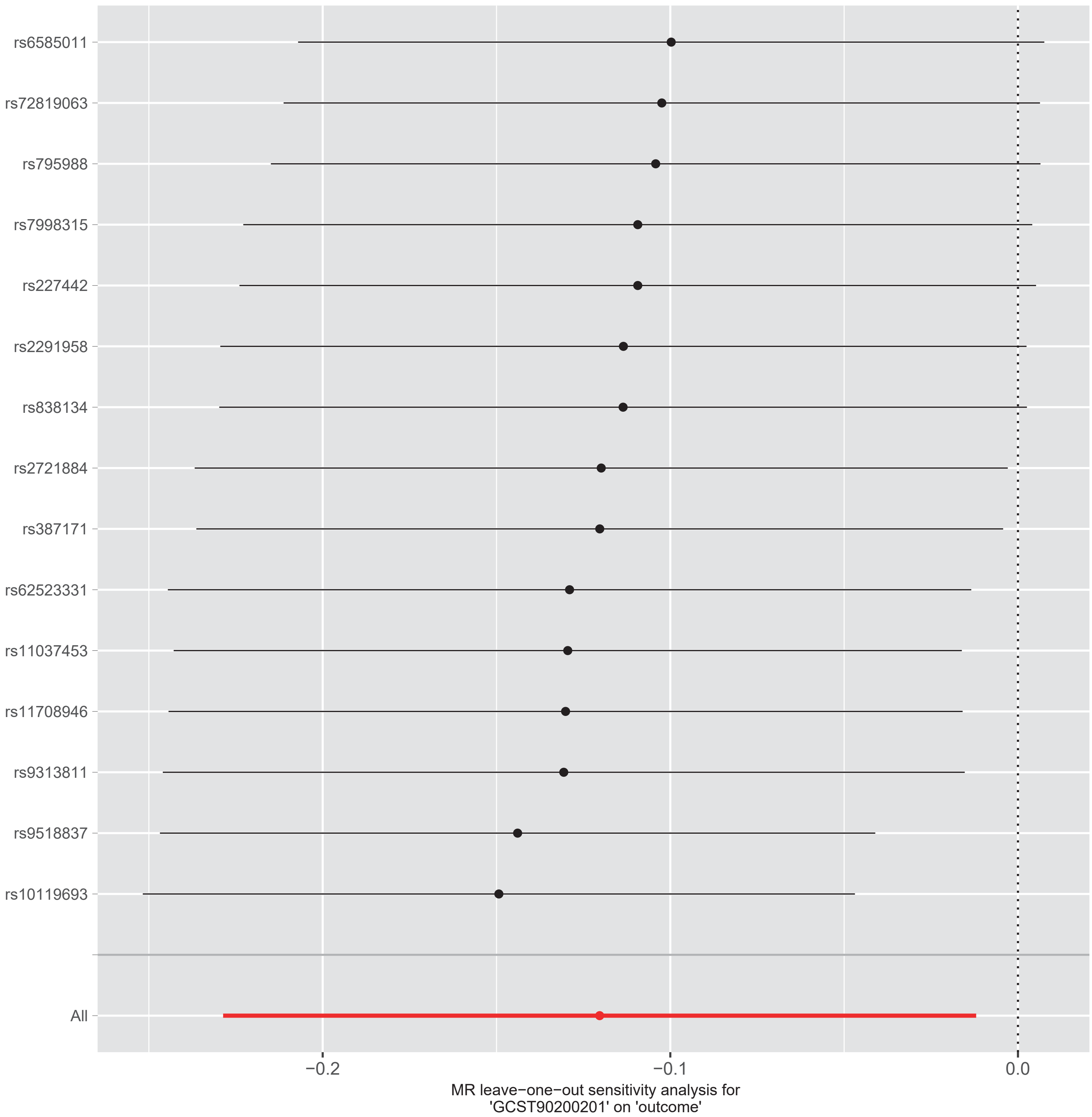
rs6585011
rs72819063
rs795988
rs7998315
rs227442
rs2291958
rs838134
rs2721884
rs387171
rs62523331
rs11037453
rs11708946
rs9313811
rs9518837
rs10119693
All
−0.2
−0.1
0.0
MR leave−one−out sensitivity analysis for
'GCST90200201' on 'outcome'

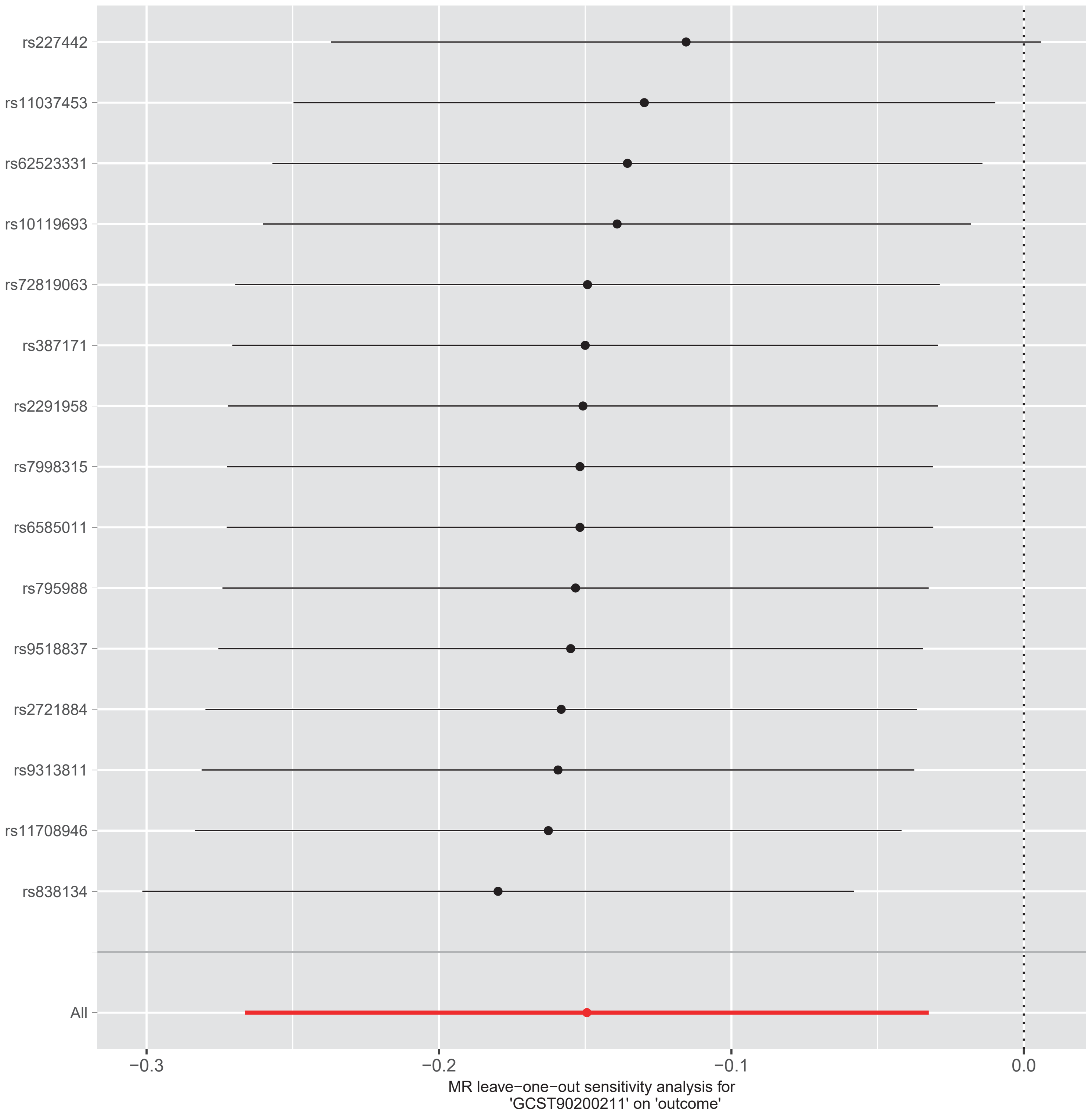
rs227442
rs11037453
rs62523331
rs10119693
rs72819063
rs387171
rs2291958
rs7998315
rs6585011
rs795988
rs9518837
rs2721884
rs9313811
rs11708946
rs838134
All
−0.3
−0.2
−0.1
0.0
MR leave−one−out sensitivity analysis for
'GCST90200211' on 'outcome'

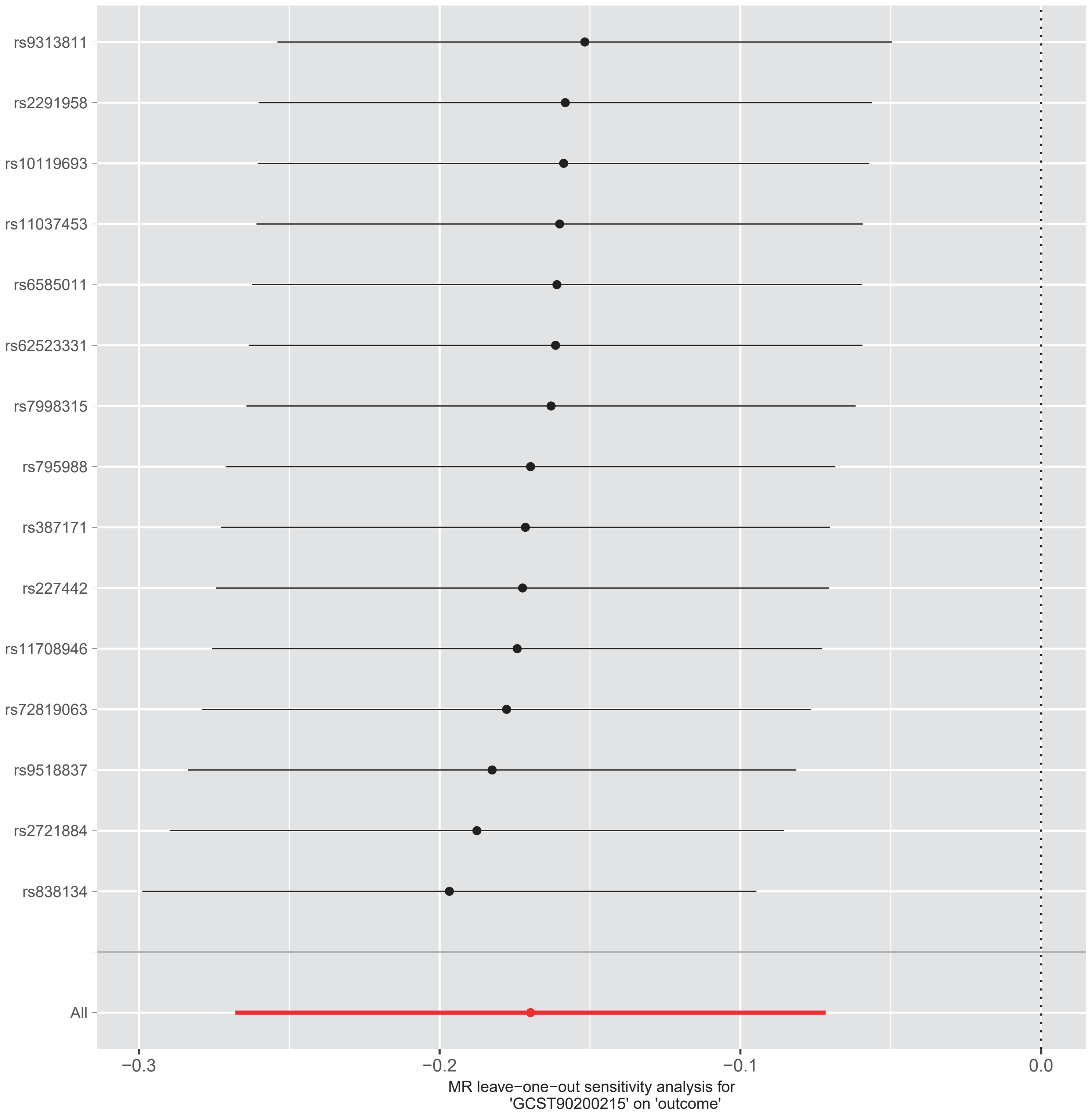
rs9313811
rs2291958
rs10119693
rs11037453
rs6585011
rs62523331
rs7998315
rs795988
rs387171
rs227442
rs11708946
rs72819063
rs9518837
rs2721884
rs838134
All
−0.3
−0.2
−0.1
0.0
MR leave−one−out sensitivity analysis for
'GCST90200215' on 'outcome'

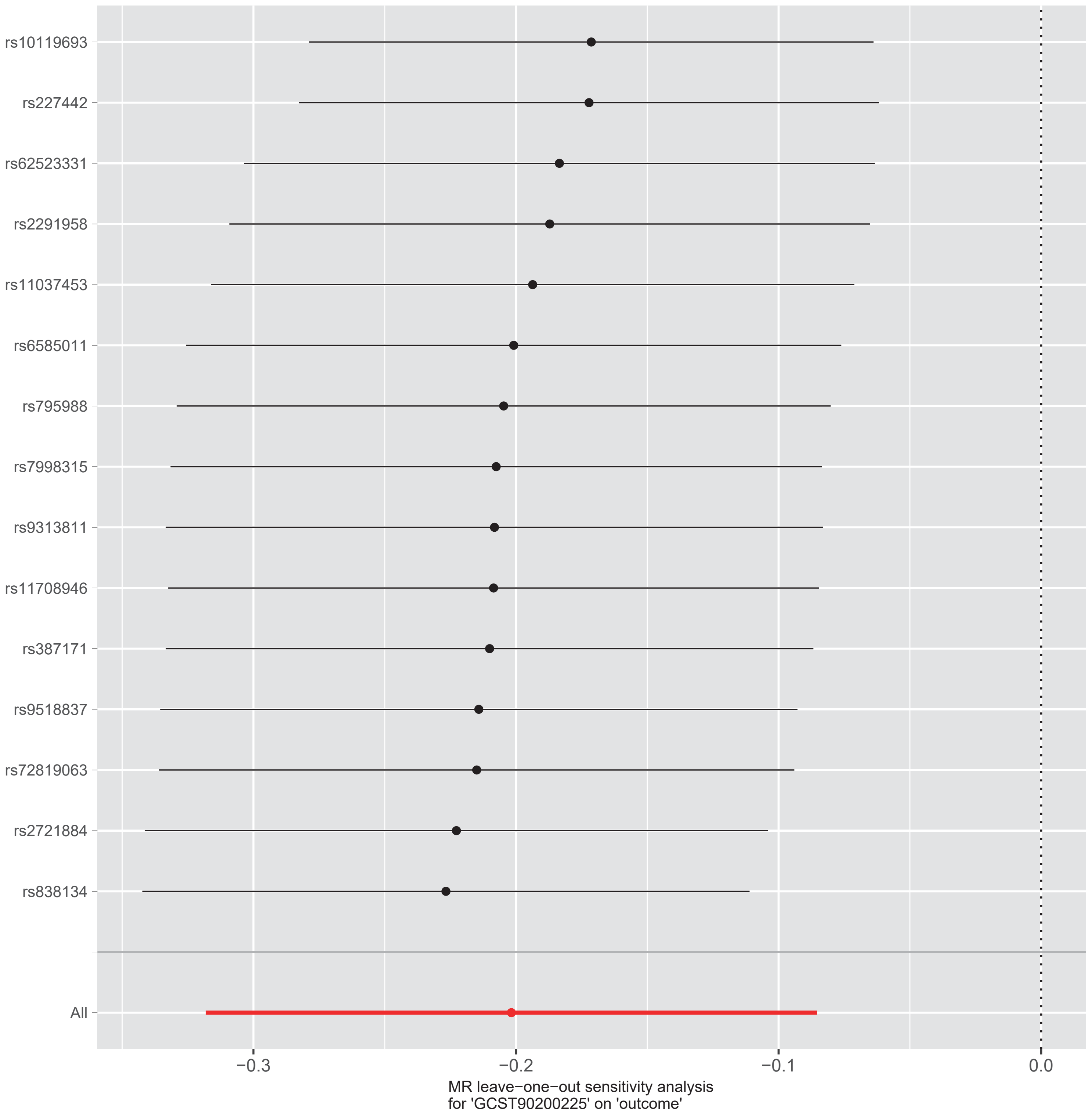
rs10119693
rs227442
rs62523331
rs2291958
rs11037453
rs6585011
rs795988
rs7998315
rs9313811
rs11708946
rs387171
rs9518837
rs72819063
rs2721884
rs838134
All
−0.3
−0.2
−0.1
0.0
MR leave−one−out sensitivity analysis
for 'GCST90200225' on 'outcome'

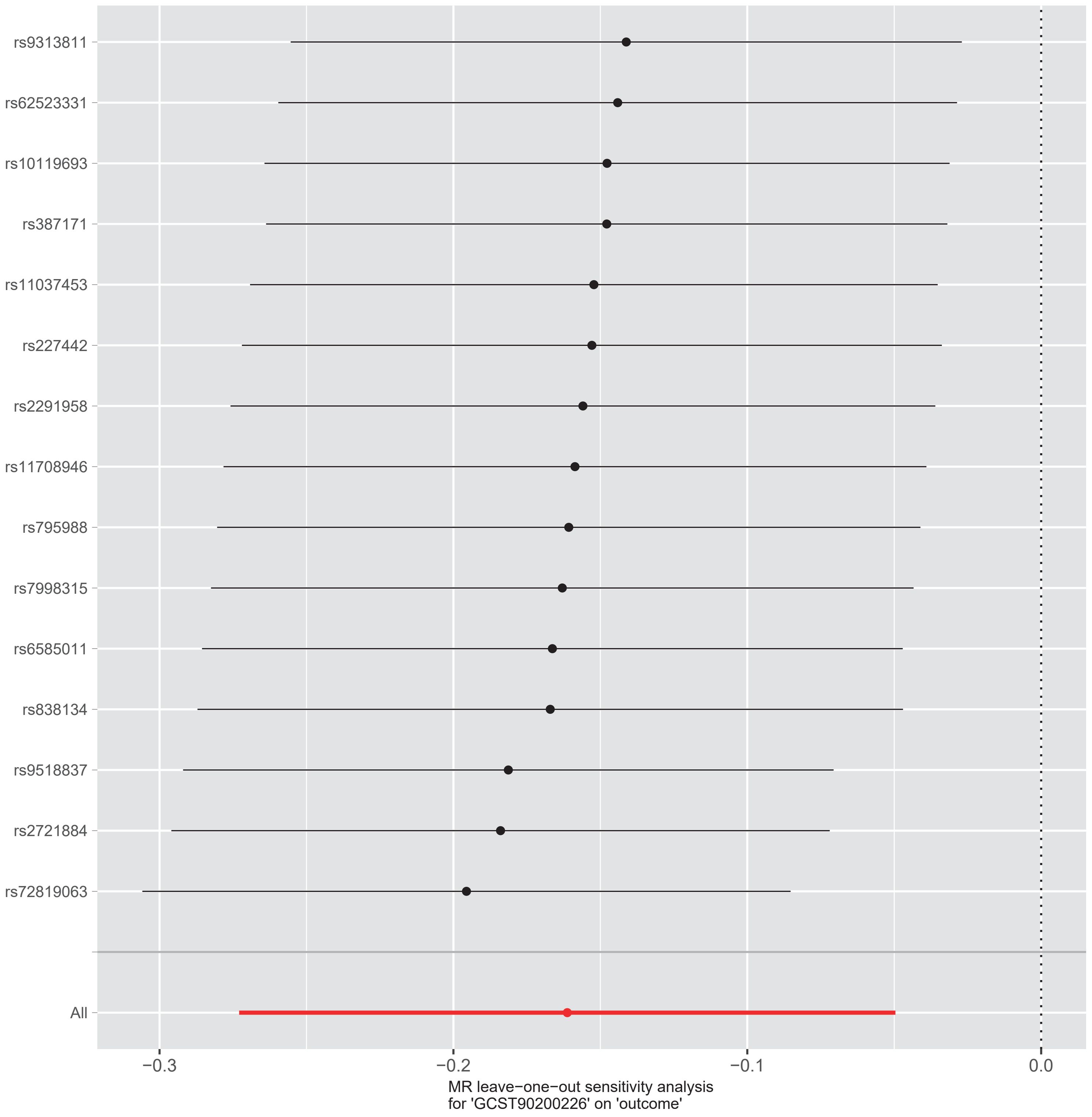
rs9313811
rs62523331
rs10119693
rs387171
rs11037453
rs227442
rs2291958
rs11708946
rs795988
rs7998315
rs6585011
rs838134
rs9518837
rs2721884
rs72819063
All
−0.3
−0.2
−0.1
0.0
MR leave−one−out sensitivity analysis
for 'GCST90200226' on 'outcome'

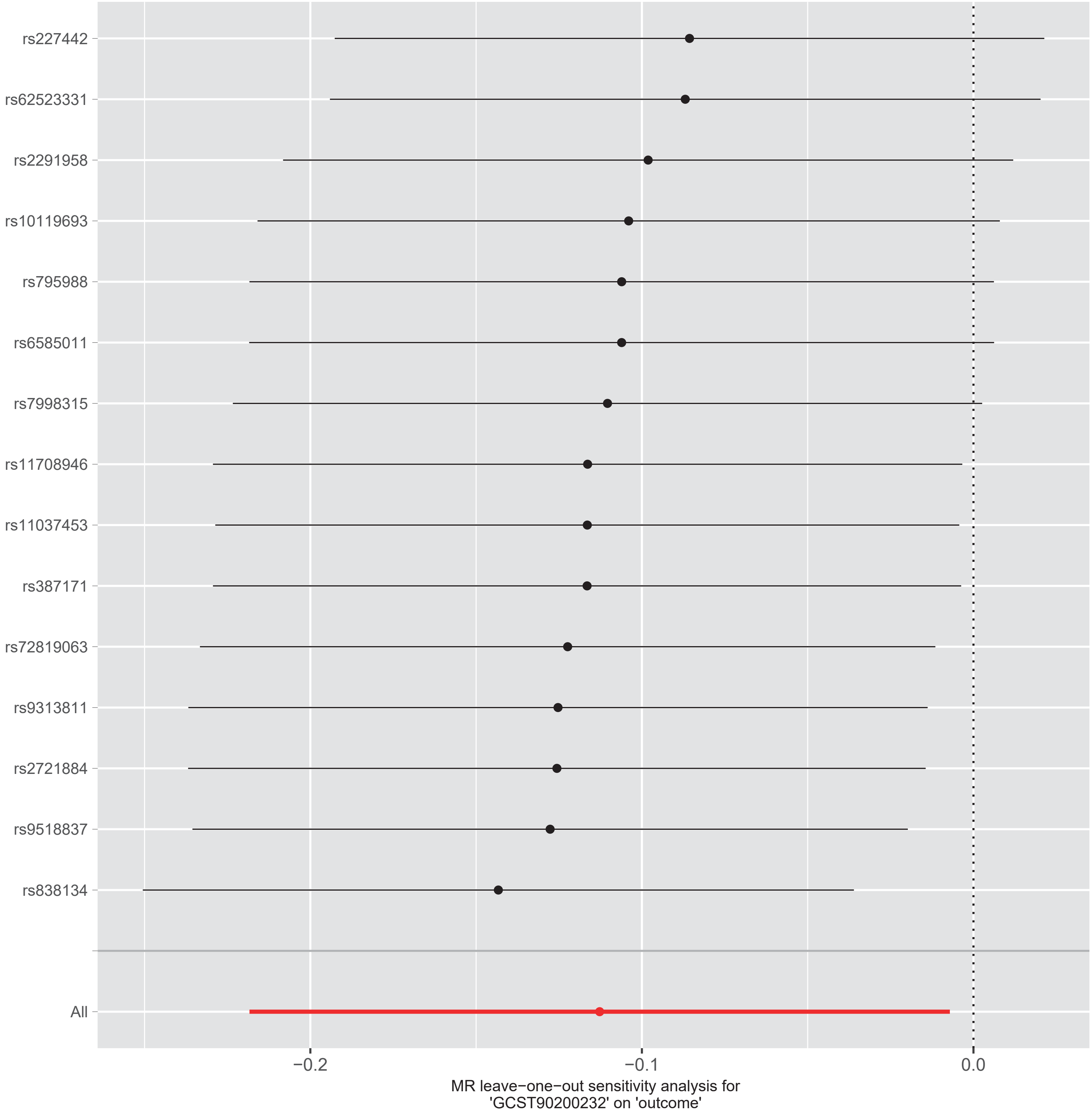
rs227442
rs62523331
rs2291958
rs10119693
rs795988
rs6585011
rs7998315
rs11708946
rs11037453
rs387171
rs72819063
rs9313811
rs2721884
rs9518837
rs838134
All
−0.2
−0.1
0.0
MR leave−one−out sensitivity analysis for
'GCST90200232' on 'outcome'

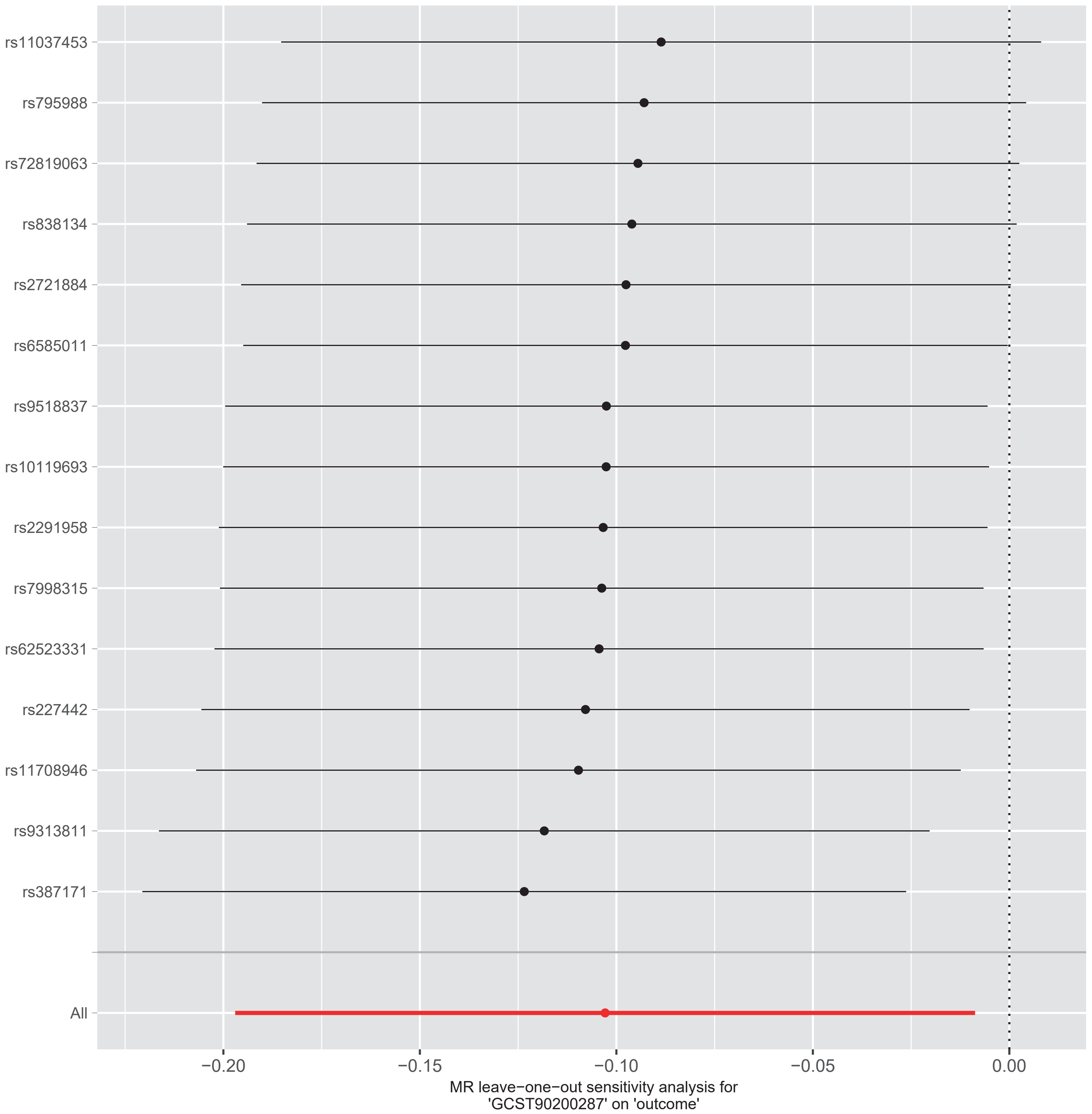
rs11037453
rs795988
rs72819063
rs838134
rs2721884
rs6585011
rs9518837
rs10119693
rs2291958
rs7998315
rs62523331
rs227442
rs11708946
rs9313811
rs387171
All
−0.20
−0.15
−0.10
−0.05
0.00
MR leave−one−out sensitivity analysis for
'GCST90200287' on 'outcome'

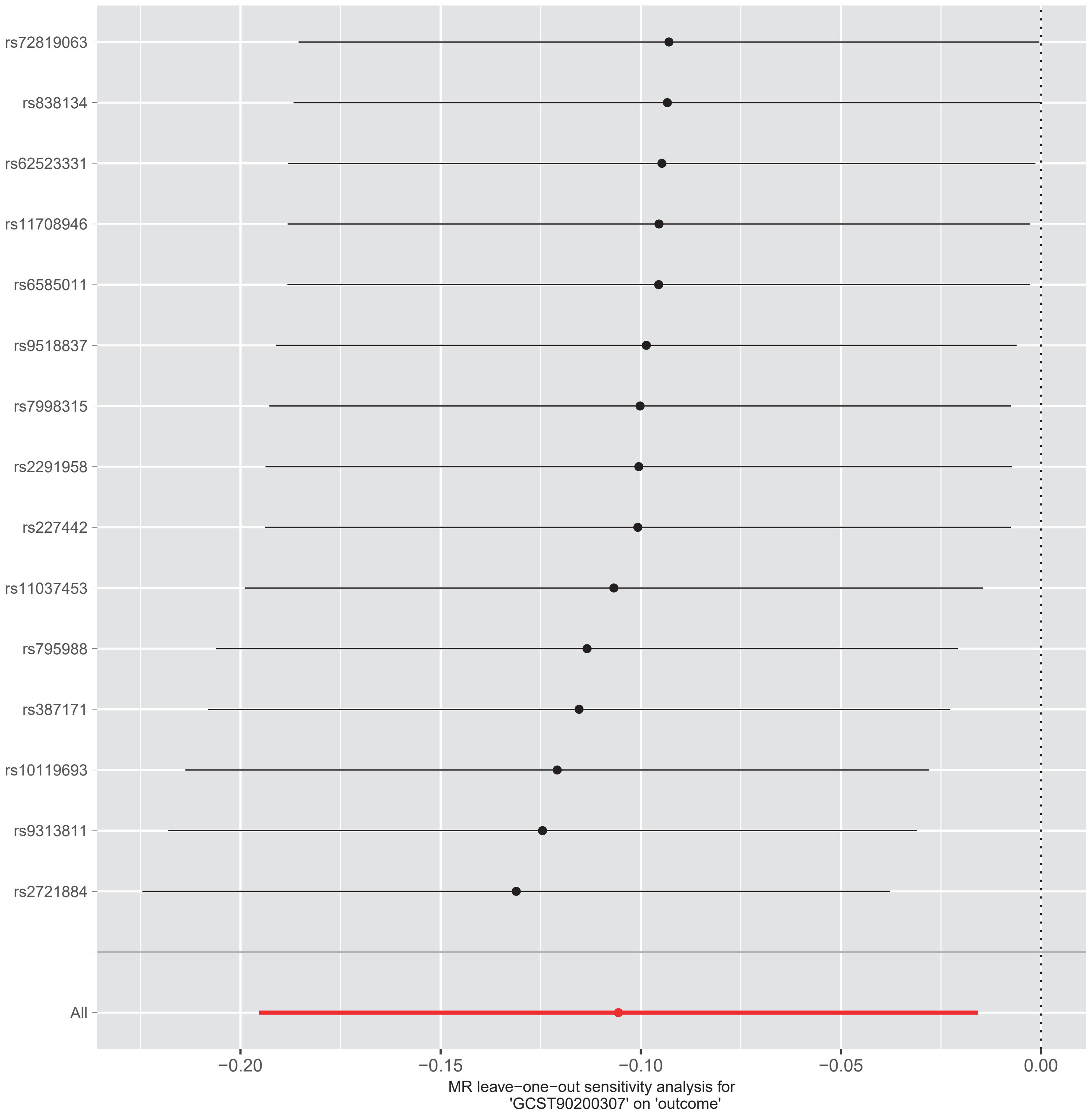
rs72819063
rs838134
rs62523331
rs11708946
rs6585011
rs9518837
rs7998315
rs2291958
rs227442
rs11037453
rs795988
rs387171
rs10119693
rs9313811
rs2721884
All
−0.20
−0.15
−0.10
−0.05
0.00
MR leave−one−out sensitivity analysis for
'GCST90200307' on 'outcome'

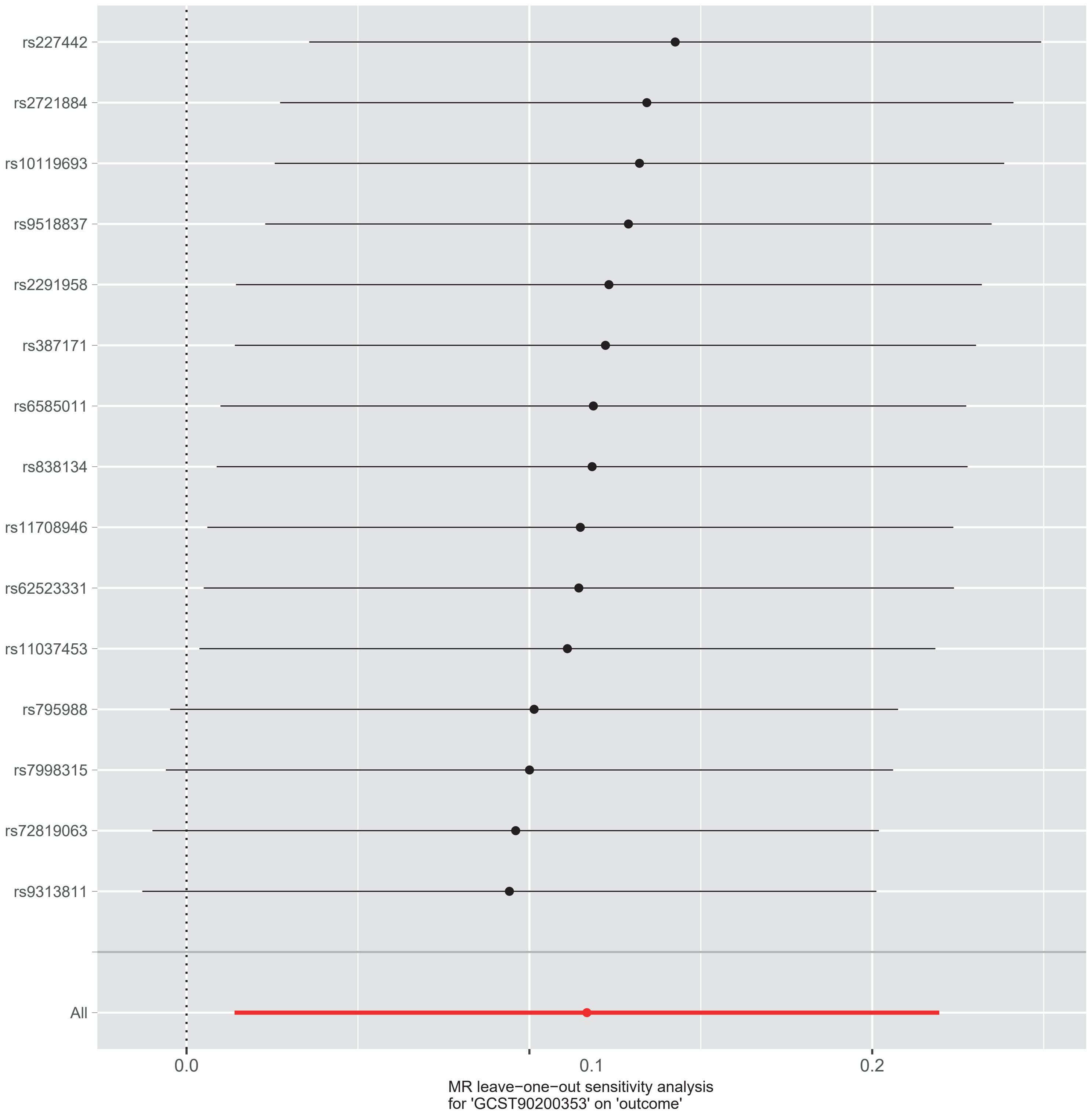
rs227442
rs2721884
rs10119693
rs9518837
rs2291958
rs387171
rs6585011
rs838134
rs11708946
rs62523331
rs11037453
rs795988
rs7998315
rs72819063
rs9313811
All
0.0
0.1
0.2
MR leave−one−out sensitivity analysis
for 'GCST90200353' on 'outcome'

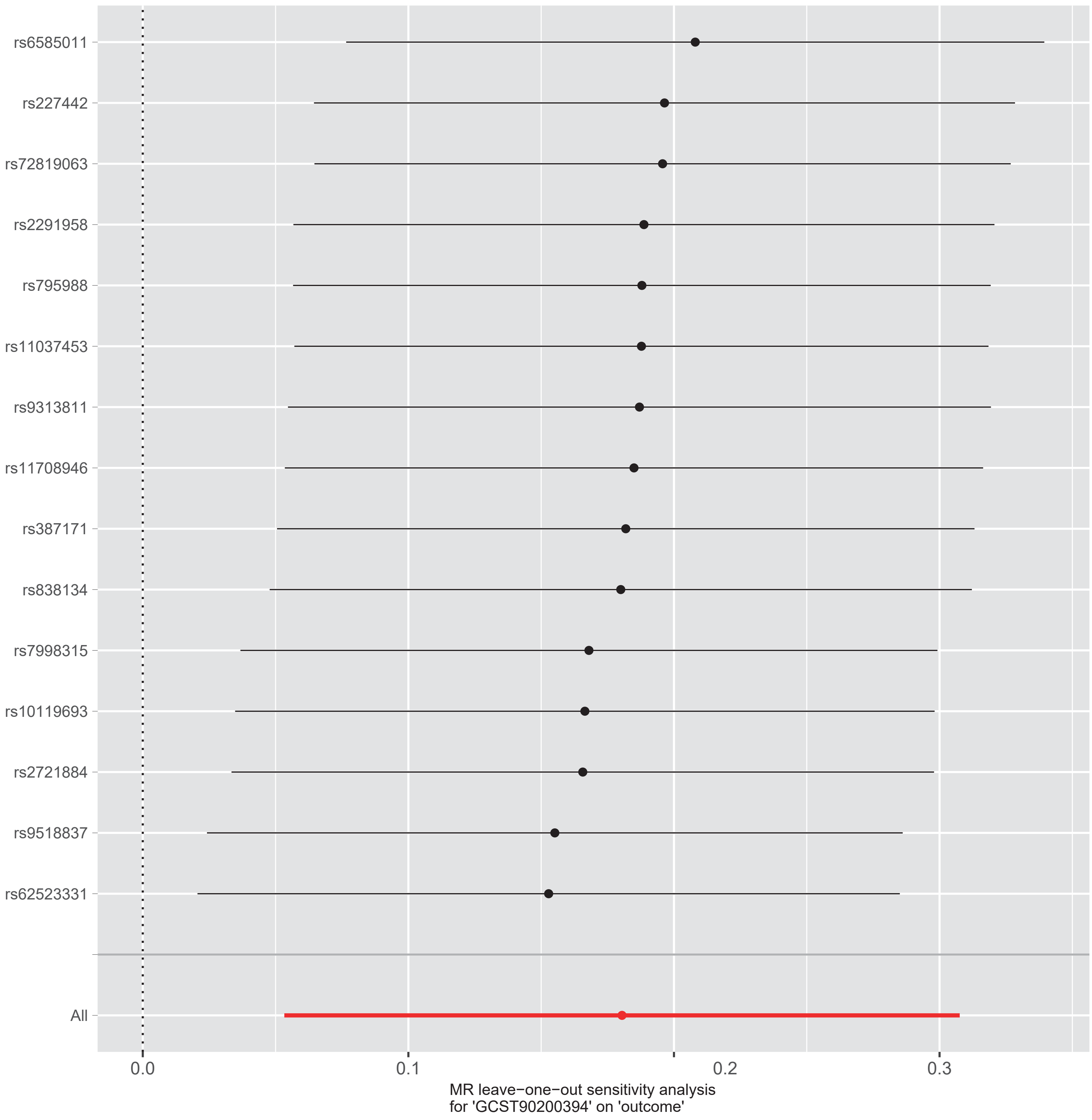
rs6585011
rs227442
rs72819063
rs2291958
rs795988
rs11037453
rs9313811
rs11708946
rs387171
rs838134
rs7998315
rs10119693
rs2721884
rs9518837
rs62523331
All
0.0
0.1
0.2
0.3
MR leave−one−out sensitivity analysis
for 'GCST90200394' on 'outcome'

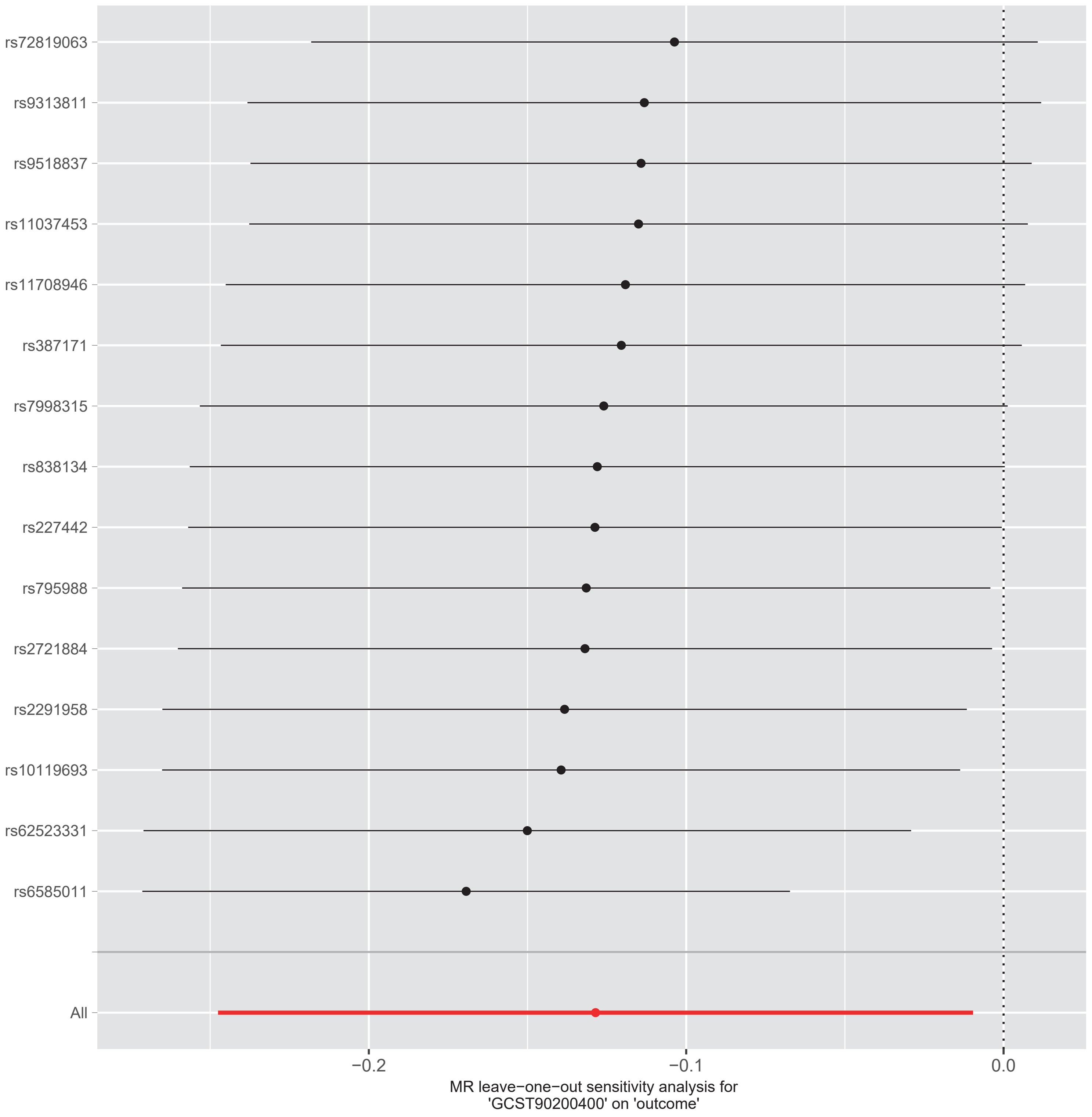
rs72819063
rs9313811
rs9518837
rs11037453
rs11708946
rs387171
rs7998315
rs838134
rs227442
rs795988
rs2721884
rs2291958
rs10119693
rs62523331
rs6585011
All
−0.2
−0.1
0.0
MR leave−one−out sensitivity analysis for
'GCST90200400' on 'outcome'

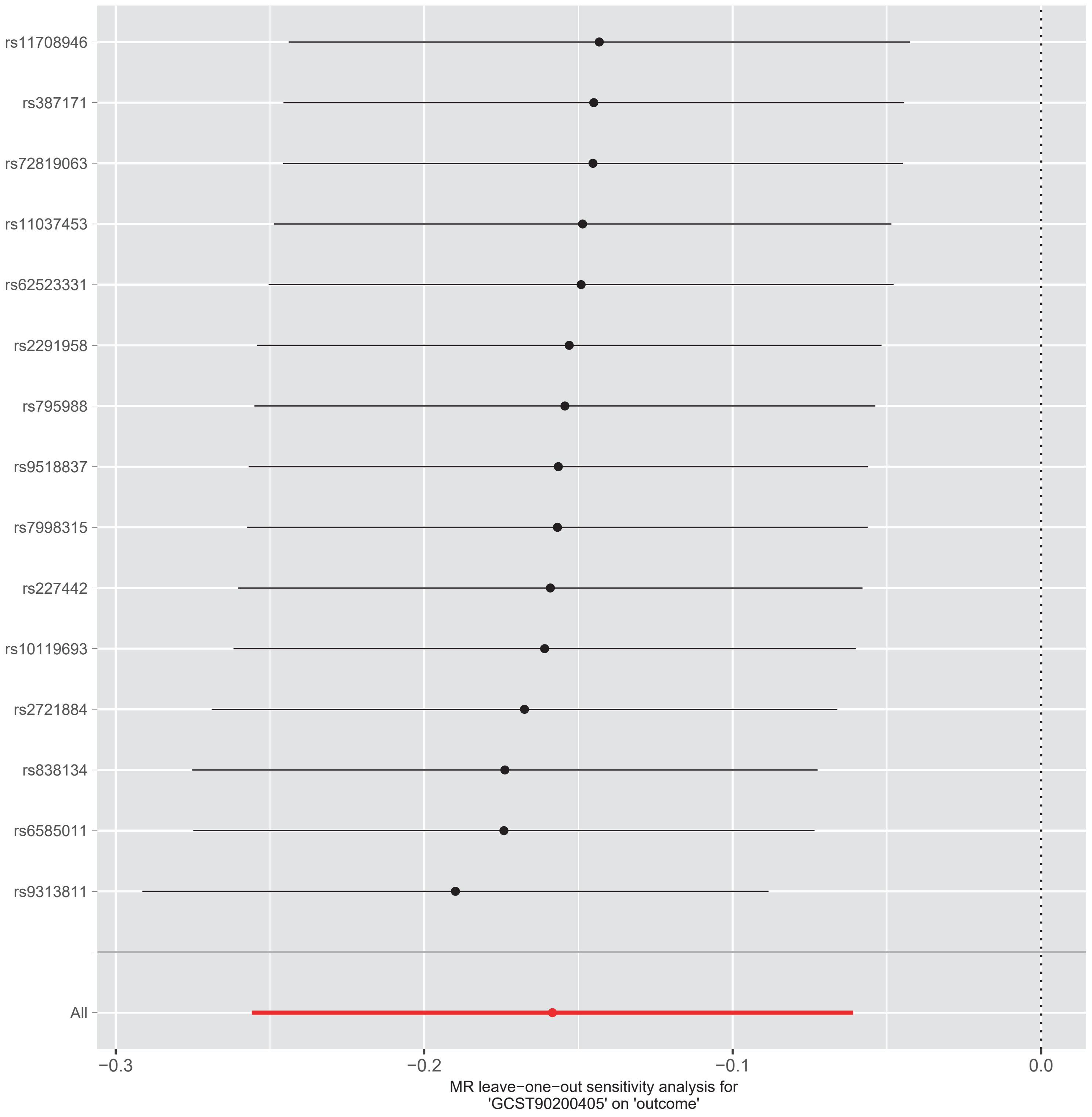
rs11708946
rs387171
rs72819063
rs11037453
rs62523331
rs2291958
rs795988
rs9518837
rs7998315
rs227442
rs10119693
rs2721884
rs838134
rs6585011
rs9313811
All
−0.3
−0.2
−0.1
0.0
MR leave−one−out sensitivity analysis for
'GCST90200405' on 'outcome'

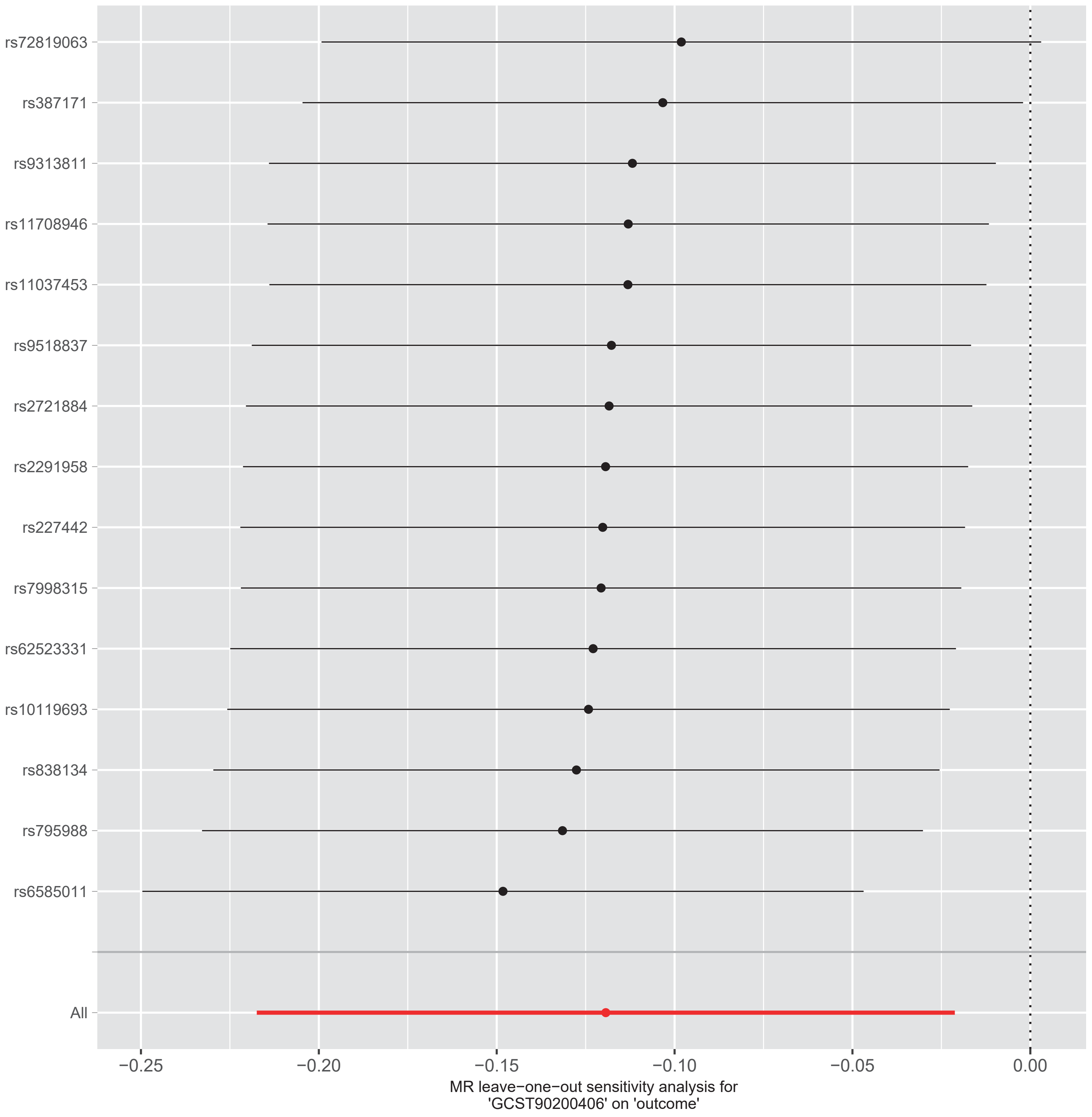

rs72819063
rs387171
rs9313811
rs11708946
rs11037453
rs9518837
rs2721884
rs2291958
rs227442
rs7998315
rs62523331
rs10119693
rs838134
rs795988
rs6585011
All
−0.25
−0.20
−0.15
−0.10
−0.05
0.00
MR leave−one−out sensitivity analysis for
'GCST90200406' on 'outcome'

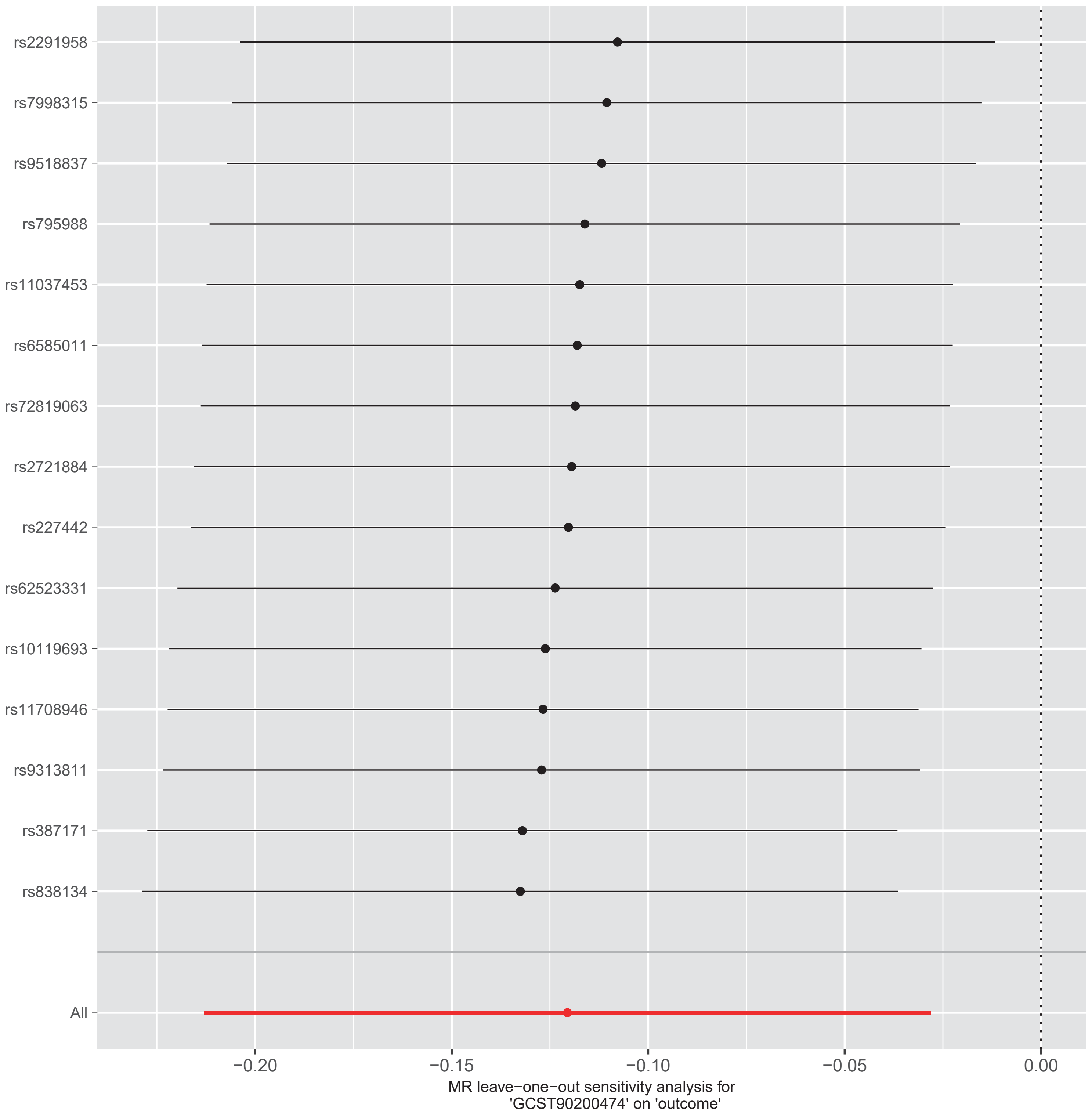
rs2291958
rs7998315
rs9518837
rs795988
rs11037453
rs6585011
rs72819063
rs2721884
rs227442
rs62523331
rs10119693
rs11708946
rs9313811
rs387171
rs838134
All
−0.20
−0.15
−0.10
−0.05
0.00
MR leave−one−out sensitivity analysis for
'GCST90200474' on 'outcome'

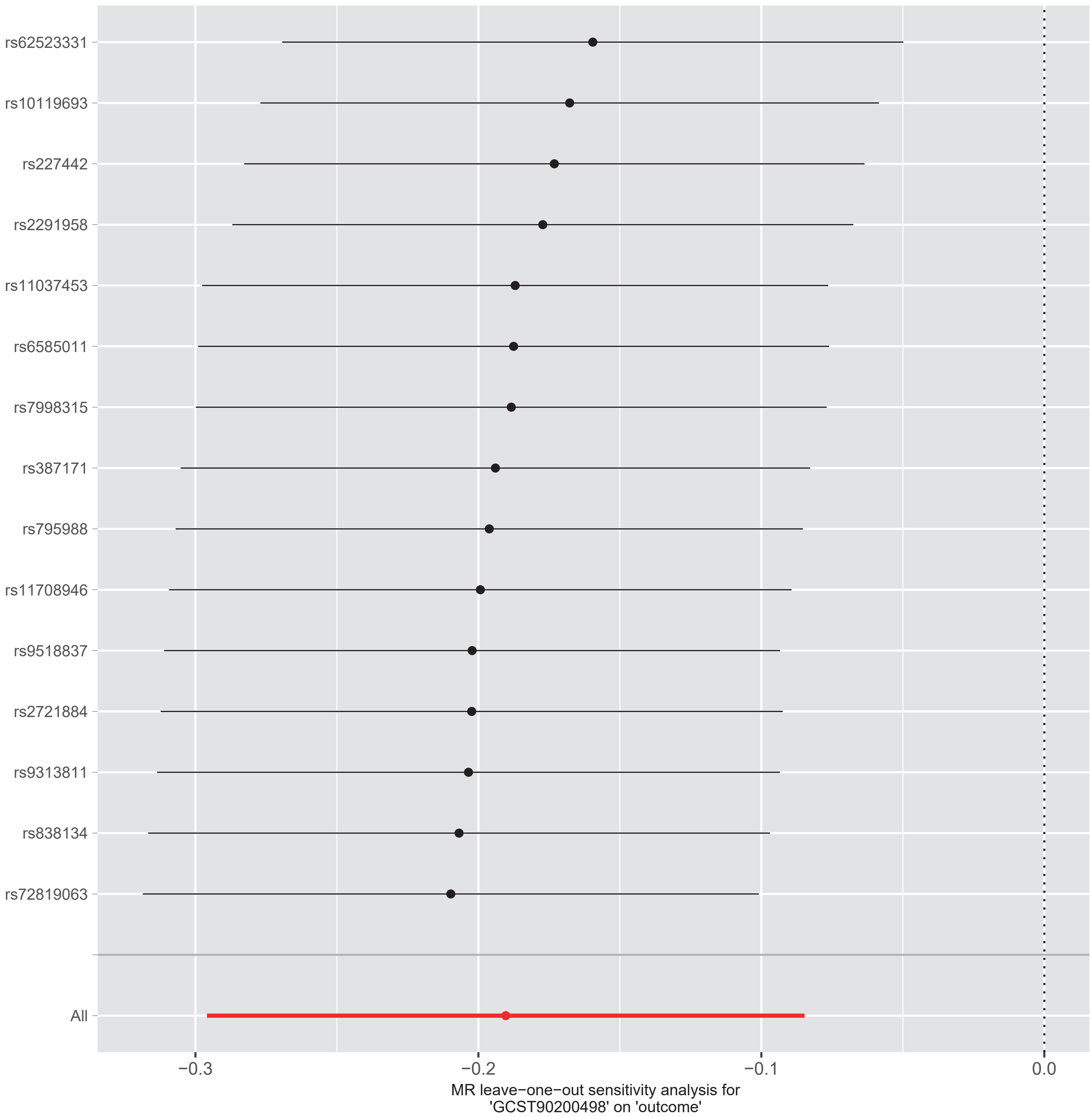

rs62523331
rs10119693
rs227442
rs2291958
rs11037453
rs6585011
rs7998315
rs387171
rs795988
rs11708946
rs9518837
rs2721884
rs9313811
rs838134
rs72819063
All
−0.3
−0.2
−0.1
0.0
MR leave−one−out sensitivity analysis for
'GCST90200498' on 'outcome'

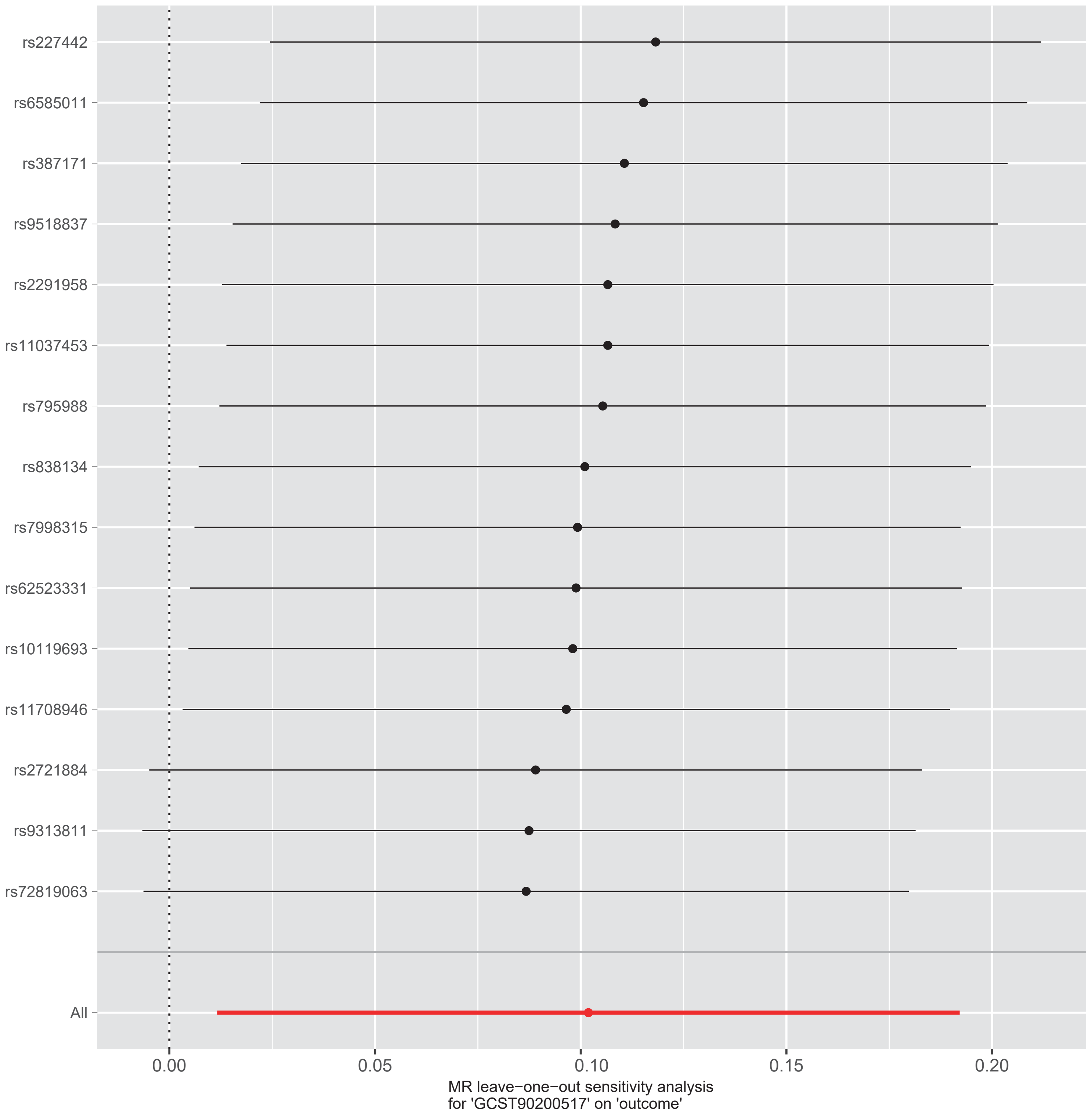
rs227442
rs6585011
rs387171
rs9518837
rs2291958
rs11037453
rs795988
rs838134
rs7998315
rs62523331
rs10119693
rs11708946
rs2721884
rs9313811
rs72819063
All
0.00
0.05
0.10
0.15
0.20
MR leave−one−out sensitivity analysis
for 'GCST90200517' on 'outcome'

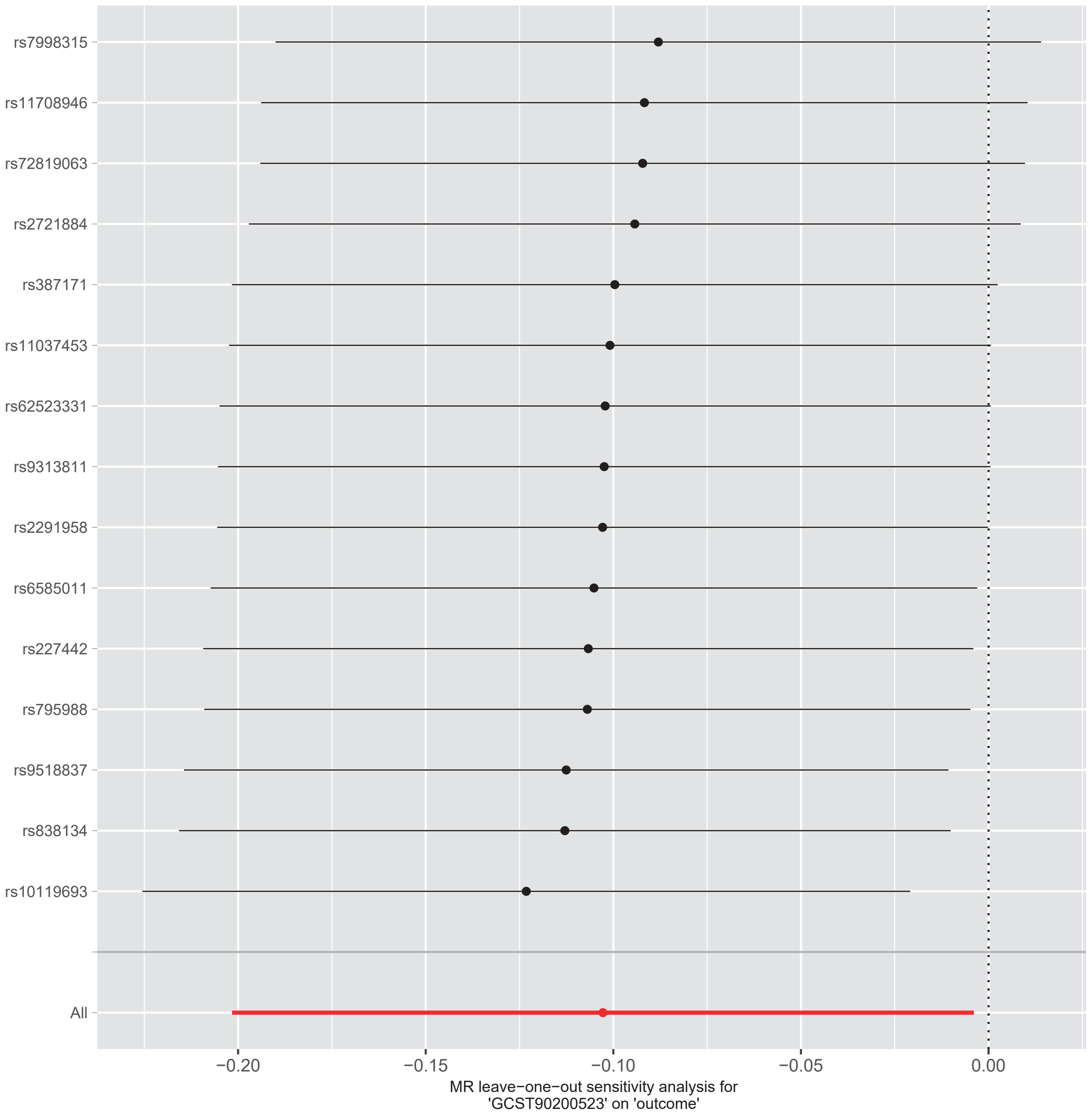
rs7998315
rs11708946
rs72819063
rs2721884
rs387171
rs11037453
rs62523331
rs9313811
rs2291958
rs6585011
rs227442
rs795988
rs9518837
rs838134
rs10119693
All
−0.20
−0.15
−0.10
−0.05
0.00
MR leave−one−out sensitivity analysis for
'GCST90200523' on 'outcome'

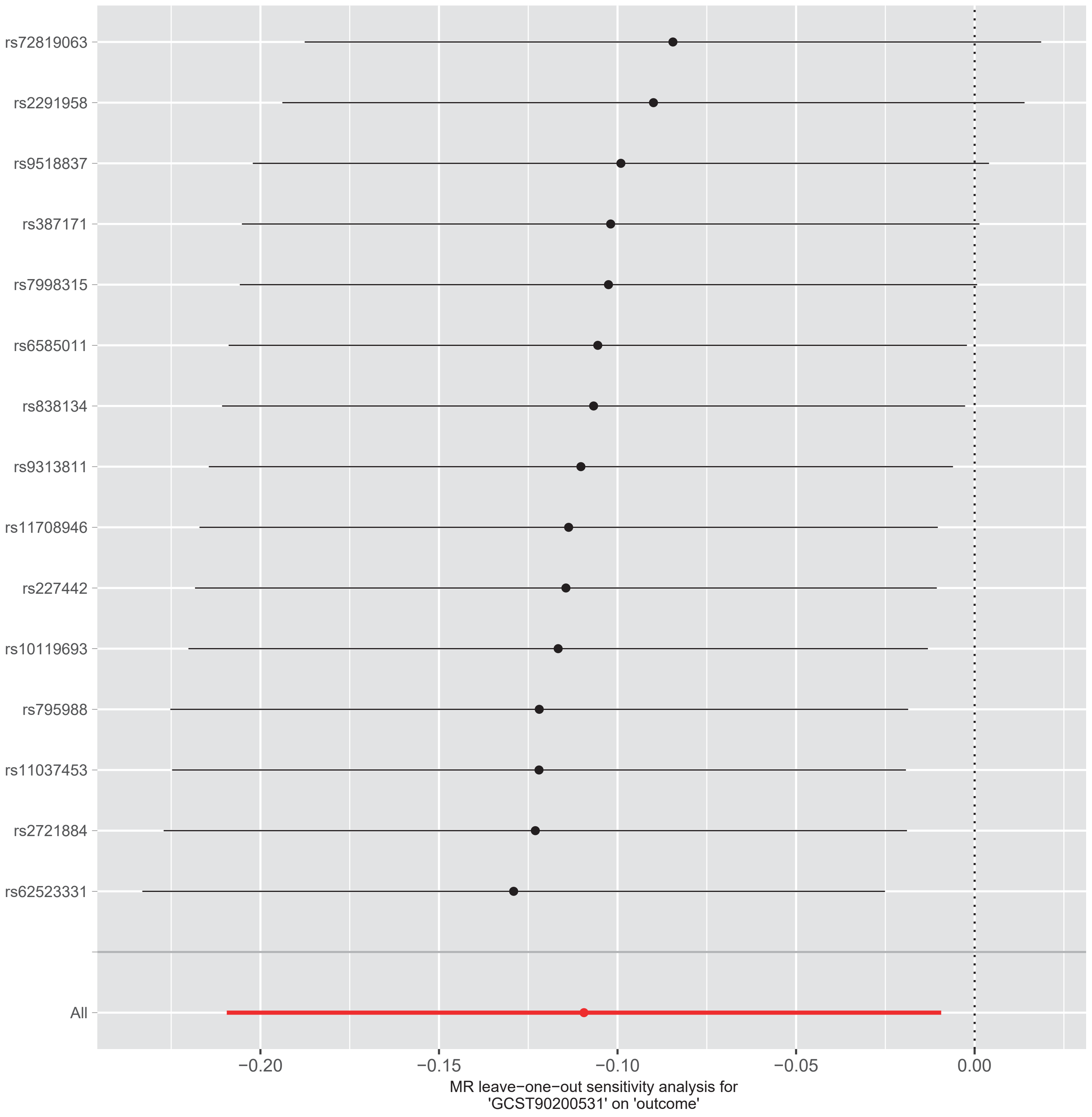
rs72819063
rs2291958
rs9518837
rs387171
rs7998315
rs6585011
rs838134
rs9313811
rs11708946
rs227442
rs10119693
rs795988
rs11037453
rs2721884
rs62523331
All
−0.20
−0.15
−0.10
−0.05
0.00
MR leave−one−out sensitivity analysis for
'GCST90200531' on 'outcome'

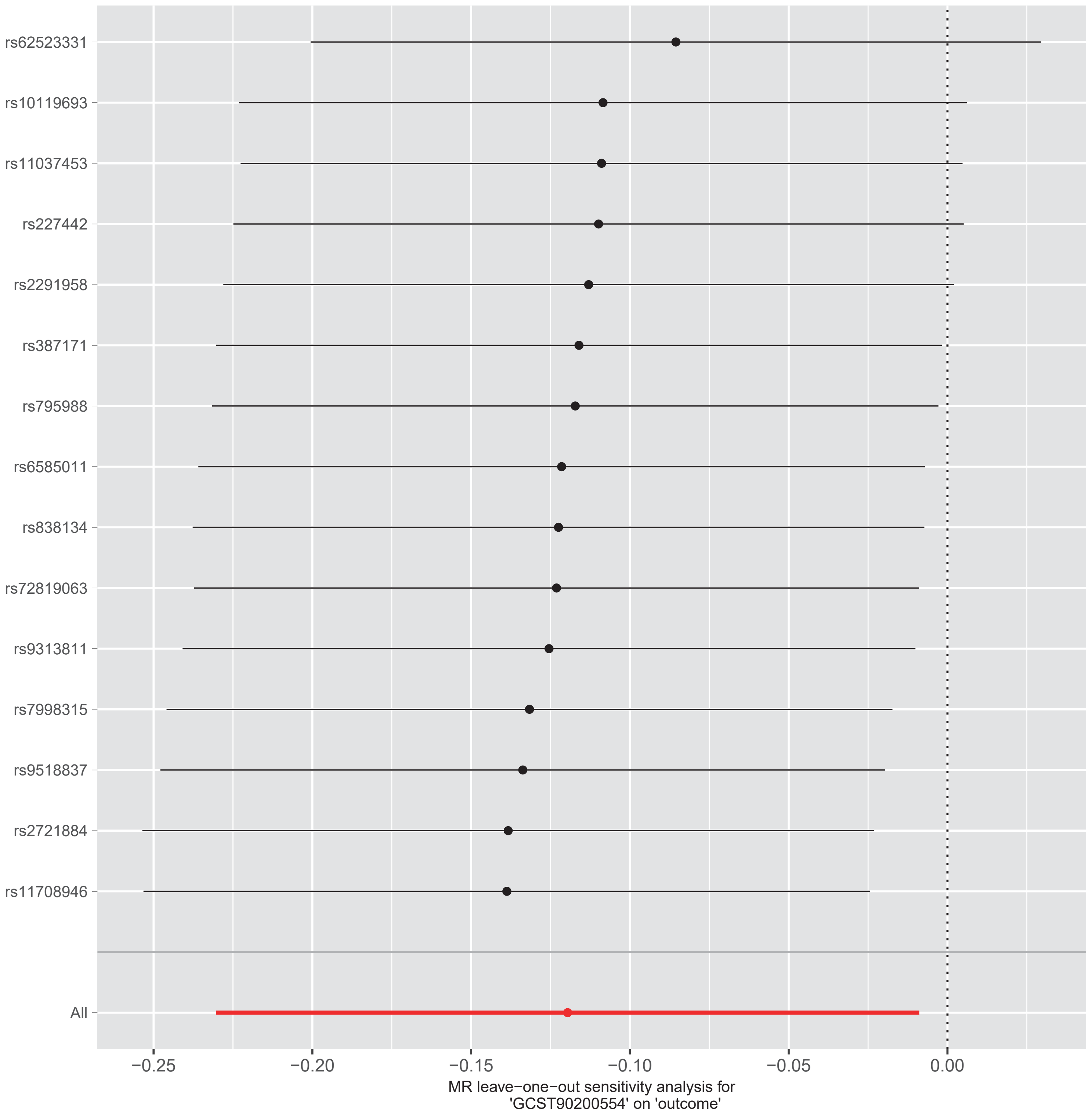

rs62523331
rs10119693
rs11037453
rs227442
rs2291958
rs387171
rs795988
rs6585011
rs838134
rs72819063
rs9313811
rs7998315
rs9518837
rs2721884
rs11708946
All
−0.25
−0.20
−0.15
−0.10
−0.05
0.00
MR leave−one−out sensitivity analysis for
'GCST90200554' on 'outcome'

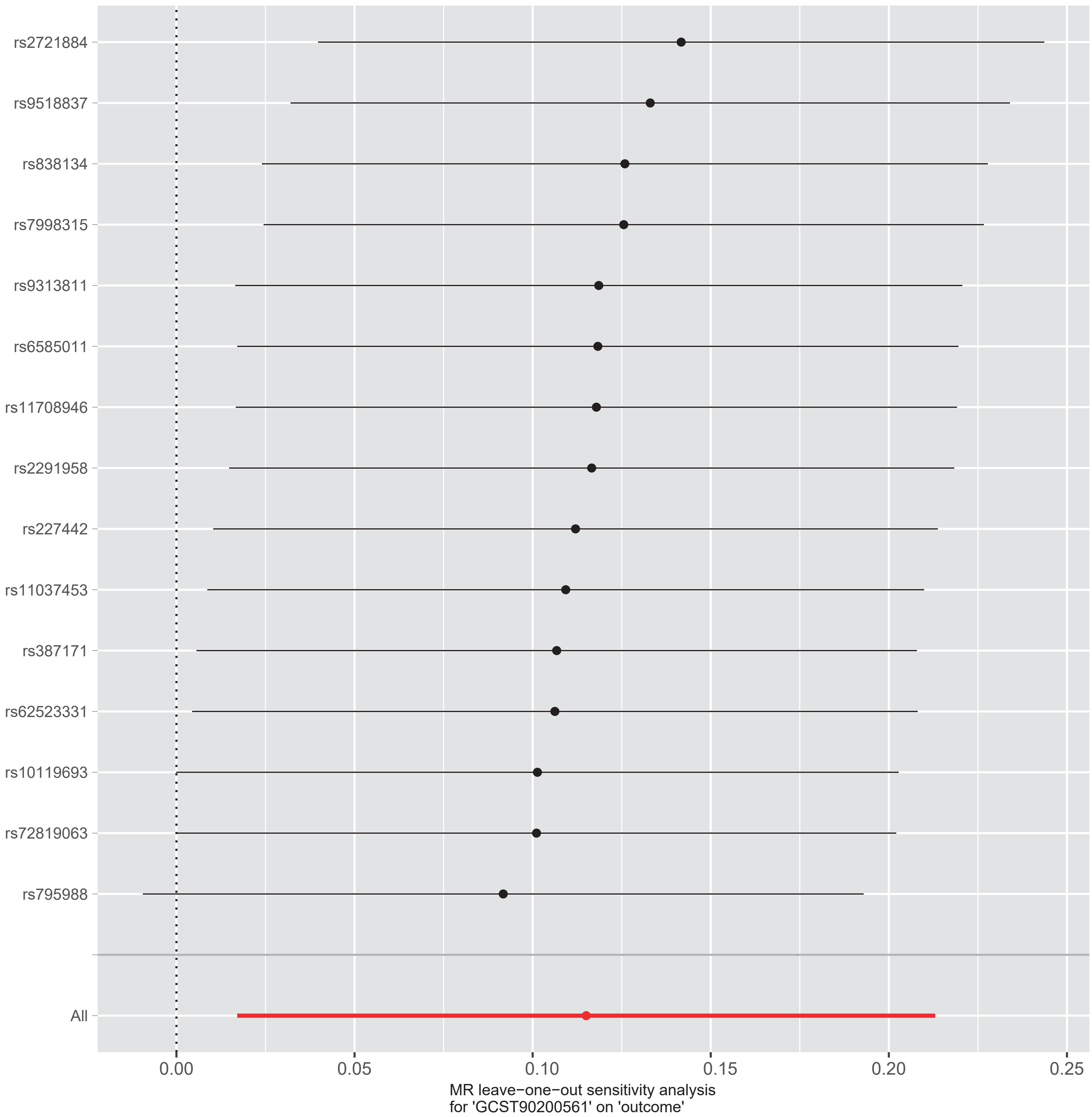
rs2721884
rs9518837
rs838134
rs7998315
rs9313811
rs6585011
rs11708946
rs2291958
rs227442
rs11037453
rs387171
rs62523331
rs10119693
rs72819063
rs795988
All
0.00
0.05
0.10
0.15
0.20
0.25
MR leave−one−out sensitivity analysis
for 'GCST90200561' on 'outcome'

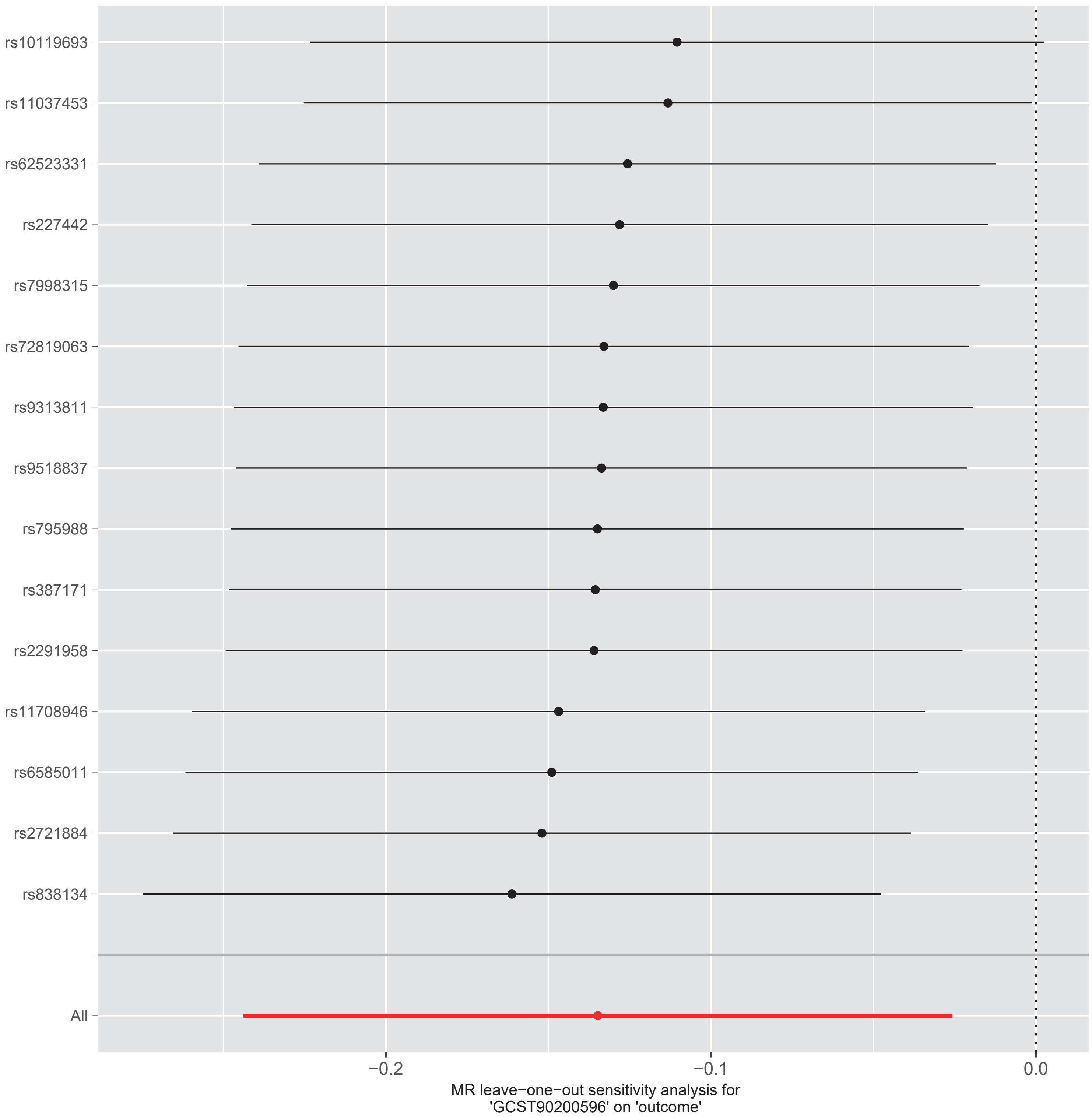
rs10119693
rs11037453
rs62523331
rs227442
rs7998315
rs72819063
rs9313811
rs9518837
rs795988
rs387171
rs2291958
rs11708946
rs6585011
rs2721884
rs838134
All
−0.2
−0.1
0.0
MR leave−one−out sensitivity analysis for
'GCST90200596' on 'outcome'

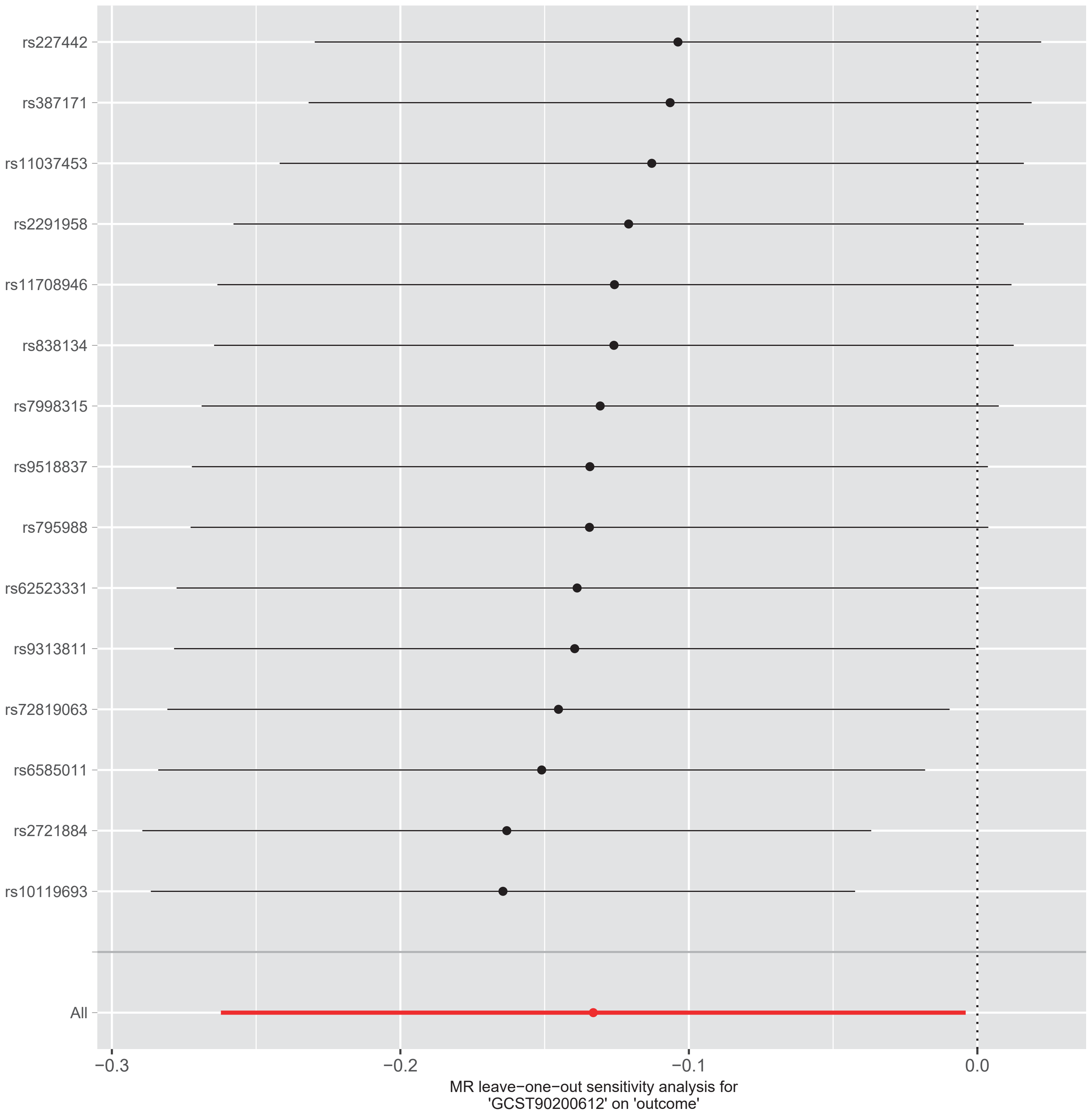

rs227442
rs387171
rs11037453
rs2291958
rs11708946
rs838134
rs7998315
rs9518837
rs795988
rs62523331
rs9313811
rs72819063
rs6585011
rs2721884
rs10119693
All
−0.3
−0.2
−0.1
0.0
MR leave−one−out sensitivity analysis for
'GCST90200612' on 'outcome'

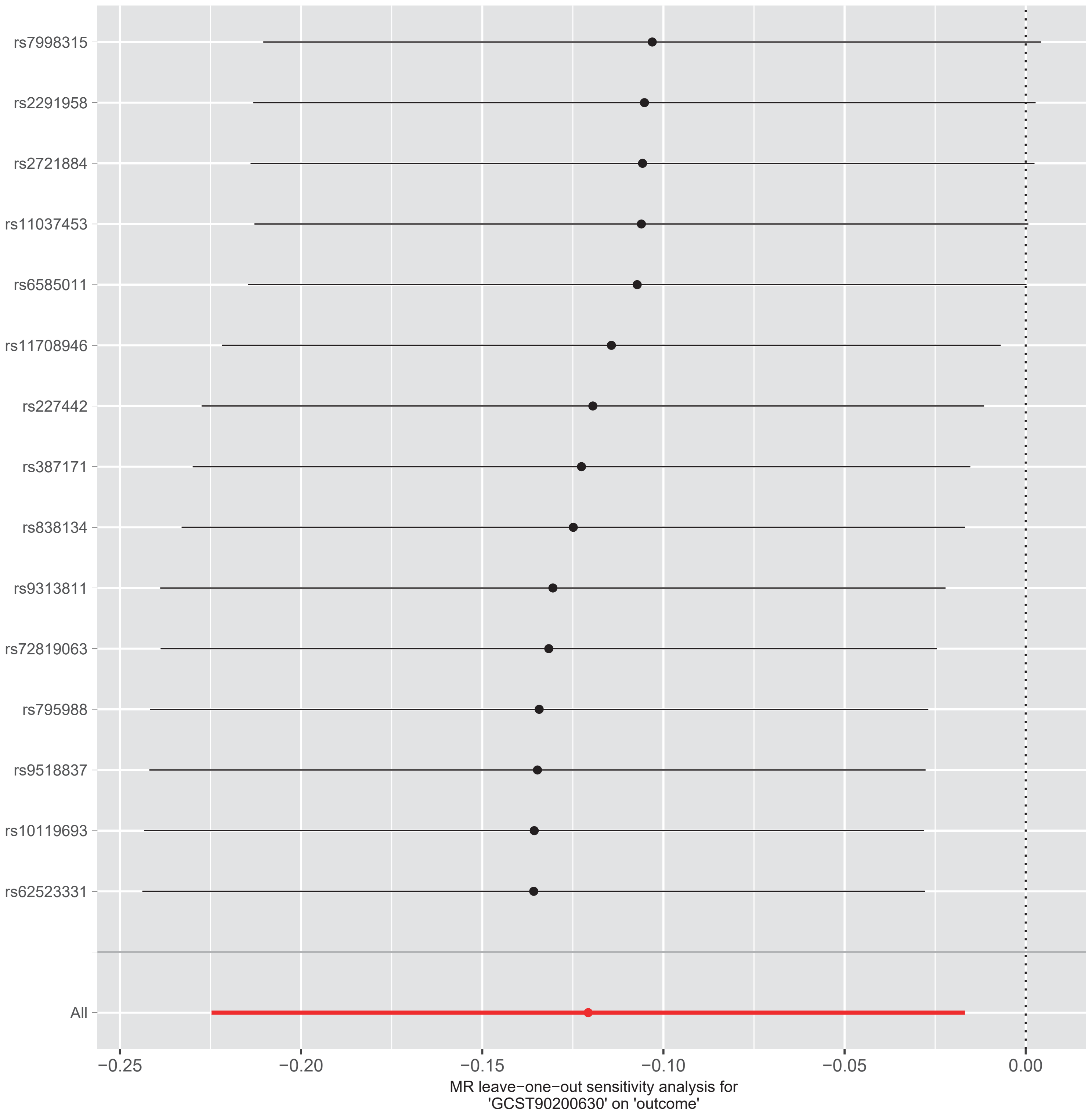
rs7998315
rs2291958
rs2721884
rs11037453
rs6585011
rs11708946
rs227442
rs387171
rs838134
rs9313811
rs72819063
rs795988
rs9518837
rs10119693
rs62523331
All
−0.25
−0.20
−0.15
−0.10
−0.05
0.00
MR leave−one−out sensitivity analysis for
'GCST90200630' on 'outcome'

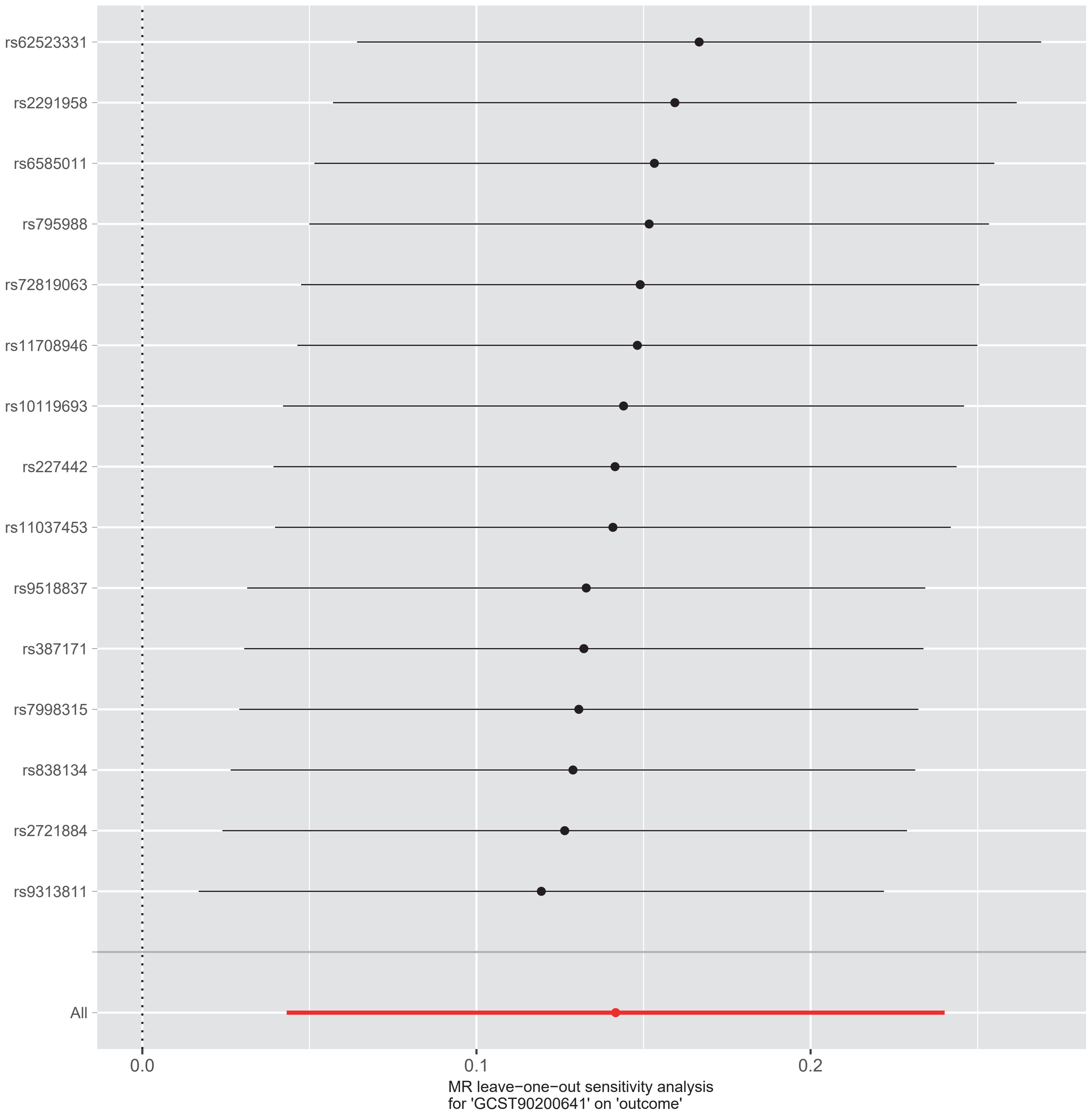
rs62523331
rs2291958
rs6585011
rs795988
rs72819063
rs11708946
rs10119693
rs227442
rs11037453
rs9518837
rs387171
rs7998315
rs838134
rs2721884
rs9313811
All
0.0
0.1
0.2
MR leave−one−out sensitivity analysis
for 'GCST90200641' on 'outcome'

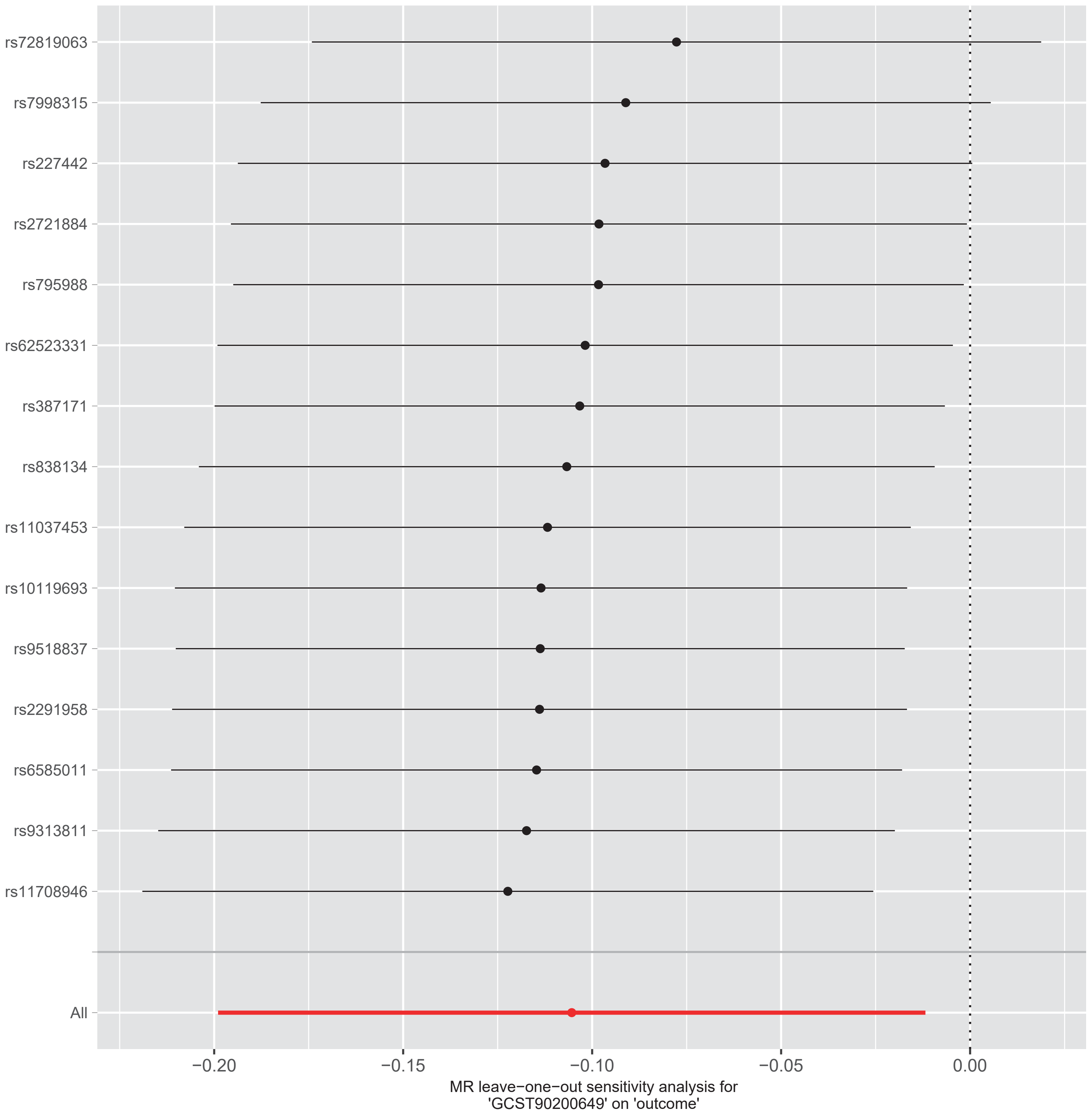
rs72819063
rs7998315
rs227442
rs2721884
rs795988
rs62523331
rs387171
rs838134
rs11037453
rs10119693
rs9518837
rs2291958
rs6585011
rs9313811
rs11708946
All
−0.20
−0.15
−0.10
−0.05
0.00
MR leave−one−out sensitivity analysis for
'GCST90200649' on 'outcome'

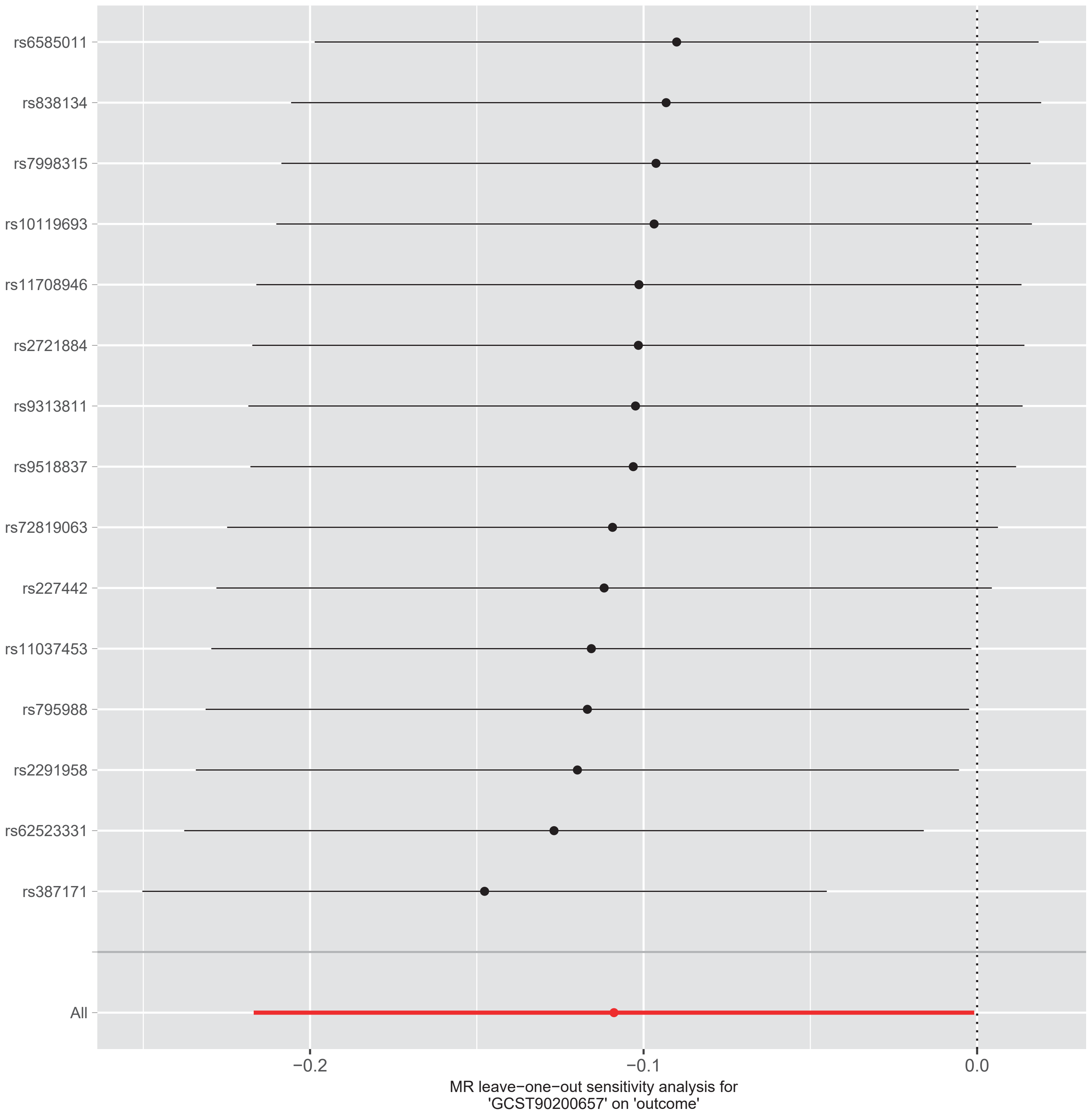
rs6585011
rs838134
rs7998315
rs10119693
rs11708946
rs2721884
rs9313811
rs9518837
rs72819063
rs227442
rs11037453
rs795988
rs2291958
rs62523331
rs387171
All
−0.2
−0.1
0.0
MR leave−one−out sensitivity analysis for
'GCST90200657' on 'outcome'

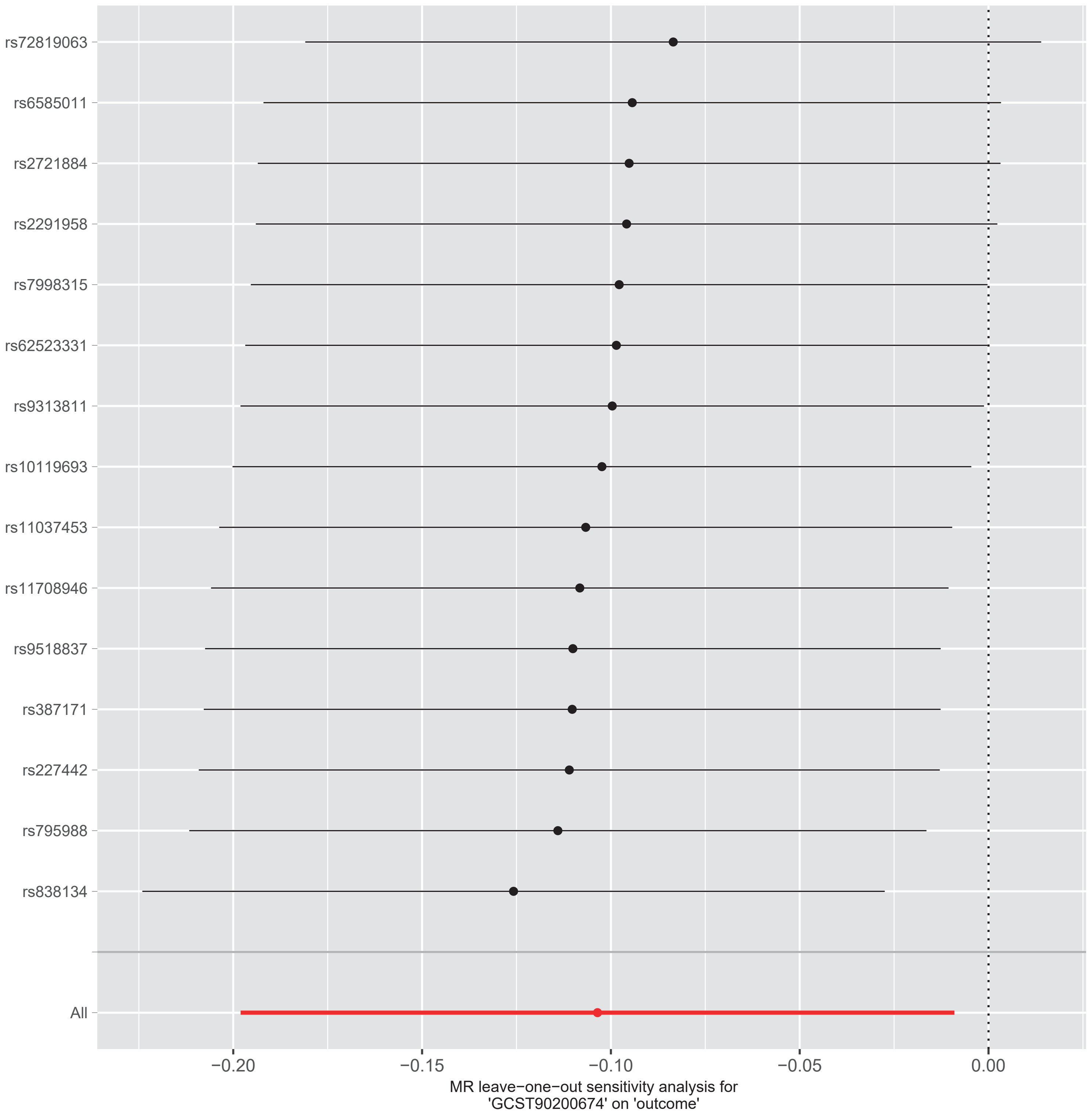
rs72819063
rs6585011
rs2721884
rs2291958
rs7998315
rs62523331
rs9313811
rs10119693
rs11037453
rs11708946
rs9518837
rs387171
rs227442
rs795988
rs838134
All
−0.20
−0.15
−0.10
−0.05
0.00
MR leave−one−out sensitivity analysis for
'GCST90200674' on 'outcome'

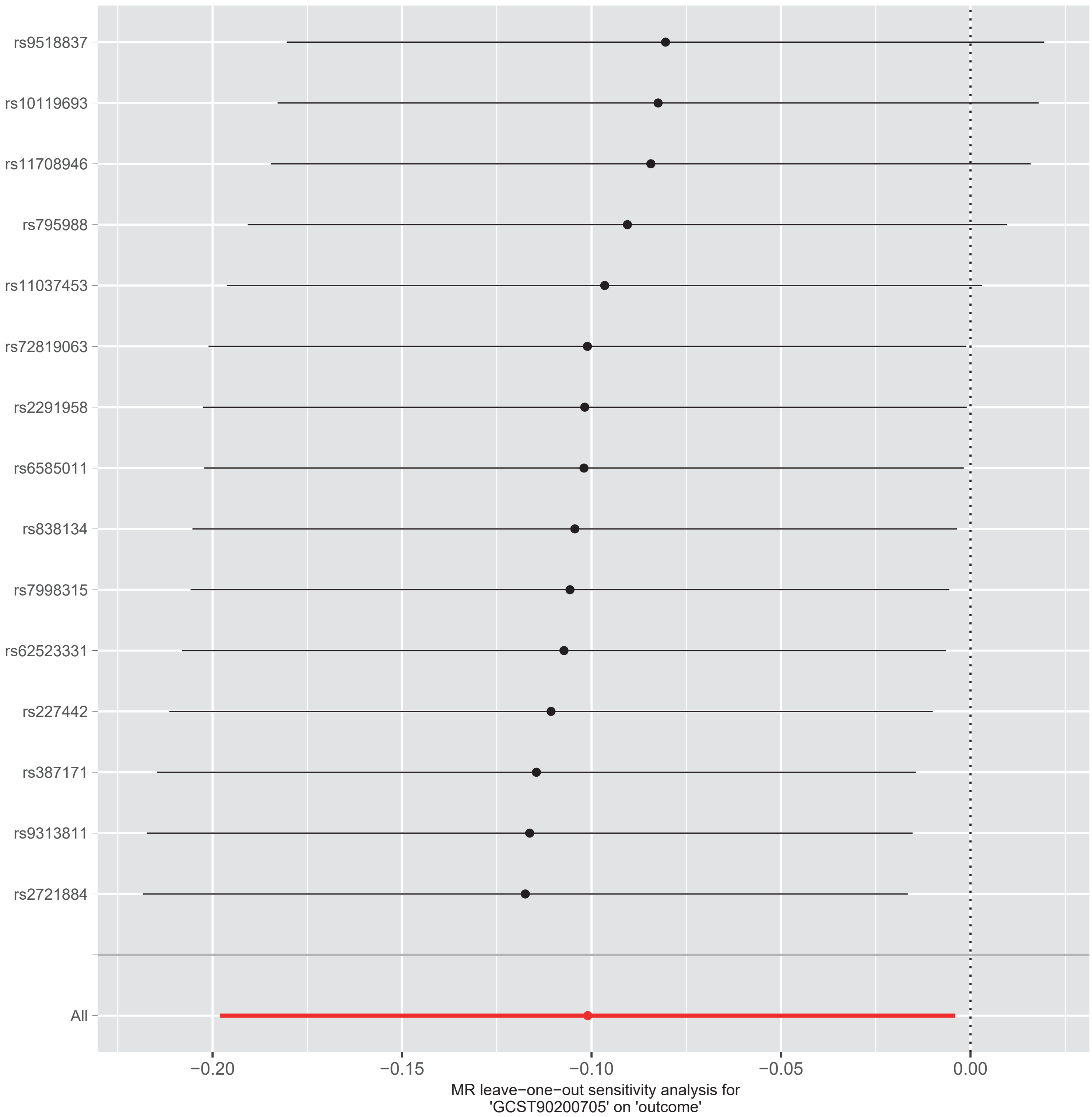
rs9518837
rs10119693
rs11708946
rs795988
rs11037453
rs72819063
rs2291958
rs6585011
rs838134
rs7998315
rs62523331
rs227442
rs387171
rs9313811
rs2721884
All
−0.20
−0.15
−0.10
−0.05
0.00
MR leave−one−out sensitivity analysis for
'GCST90200705' on 'outcome'
