# Supplementary material for: Dissecting Causal Relationships Between Gut Microbiota, Plasma Metabolites and Bladder Cancer: A Two‐Step Mendelian Randomization Study
Source: Health Sci Rep. 2025 Sep 9;8(9):e71206. doi: 10.1002/hsr2.71206 (PMC12420358; doi:10.1002/hsr2.71206)
Supplement: Supplementary file 7 — Figure S7: Funnel plots for MR causal effects of plasma metabolites on s_Bacteroides_salyersiae. [file HSR2-8-e71206-s005.pdf]

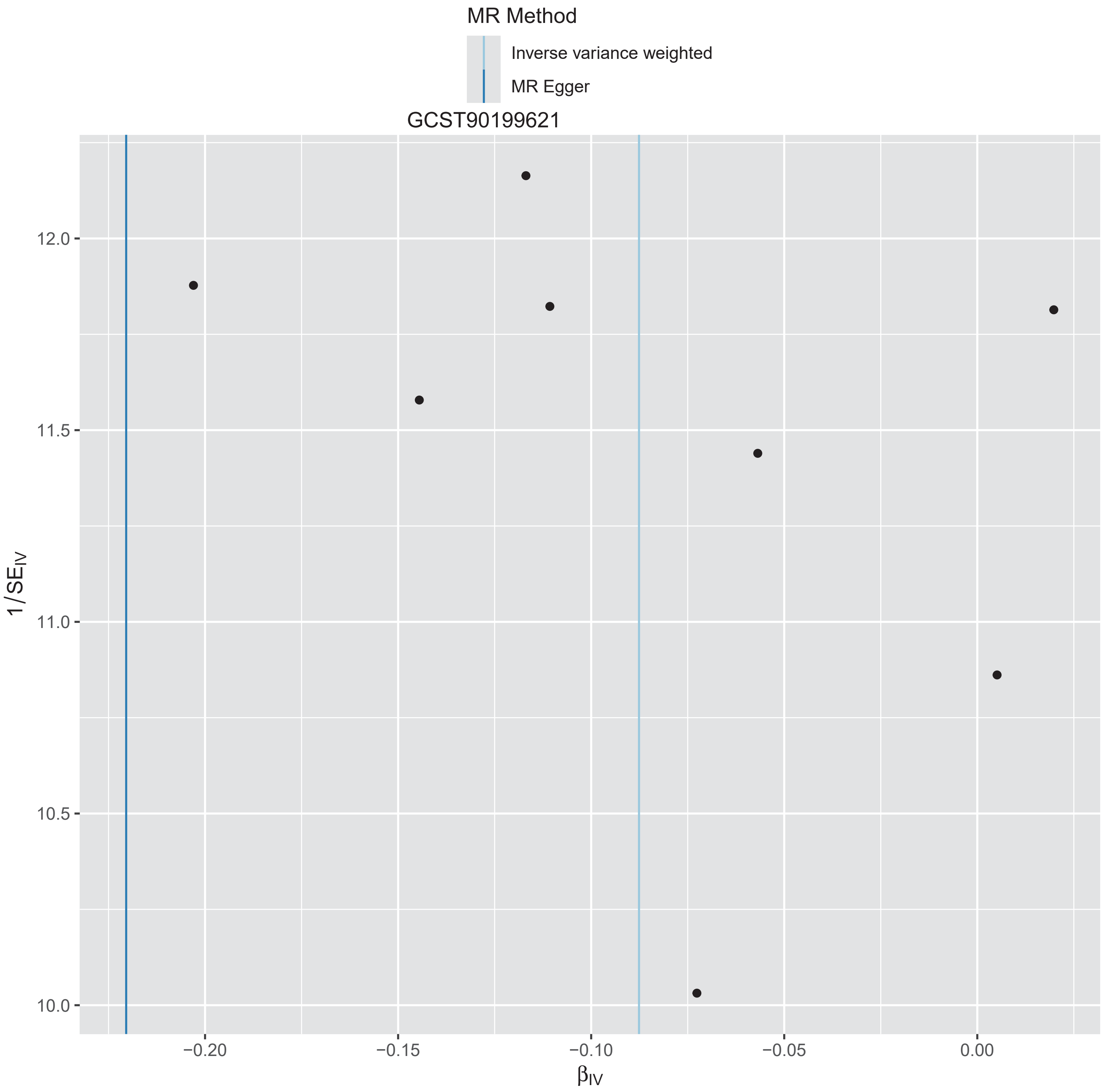
MR Method
Inverse variance weighted
MR Egger
GCST90199621
12.0
11.5
11.0
10.5
10.0
1/SE$_{IV}$
−0.20
−0.15
−0.10
−0.05
0.00
β_{IV}

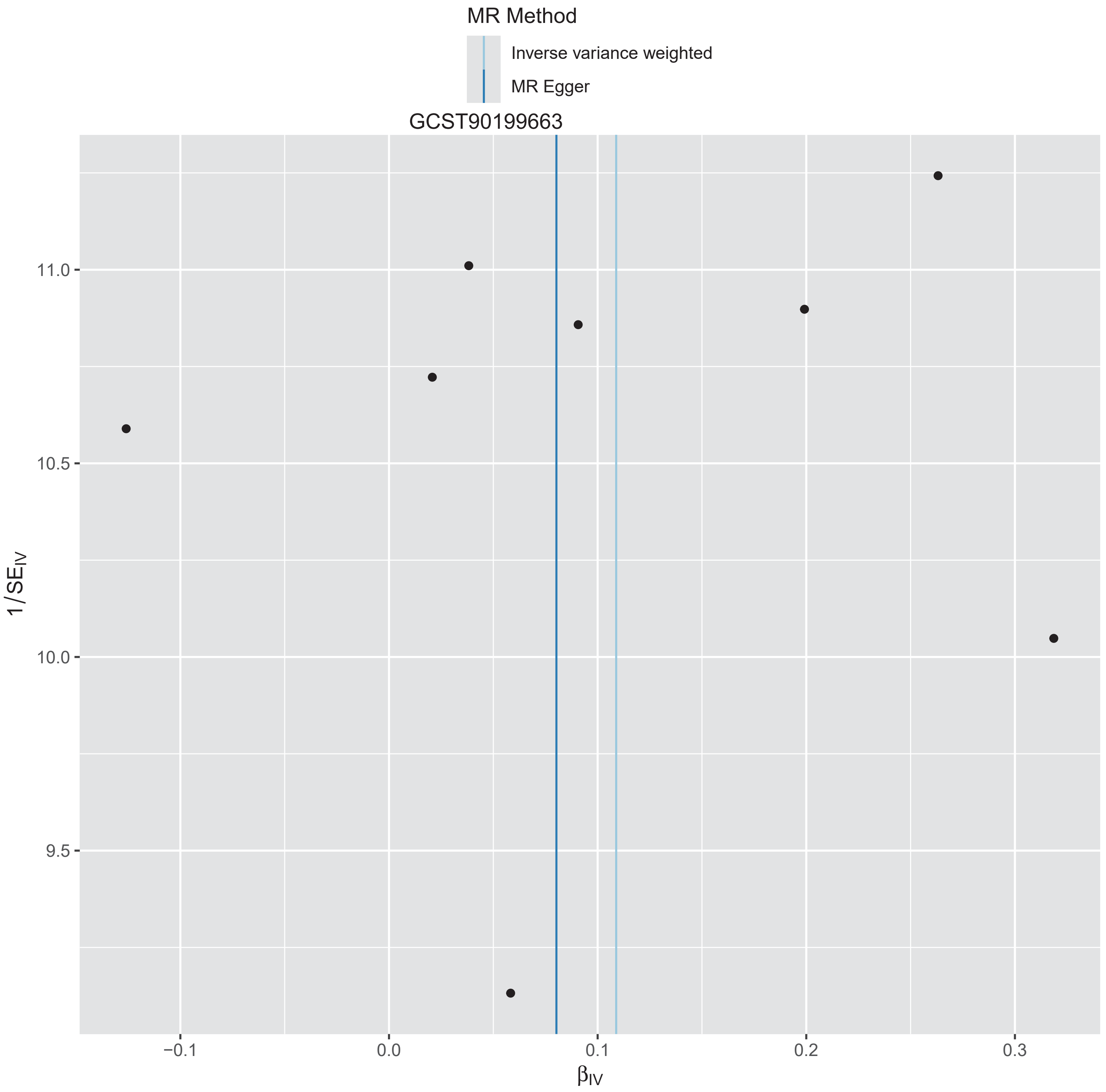
MR Method
Inverse variance weighted
MR Egger
GCST90199663
1/SE$_{IV}$
11.0
10.5
10.0
9.5
−0.1
0.0
0.1
0.2
0.3
β$_{IV}$

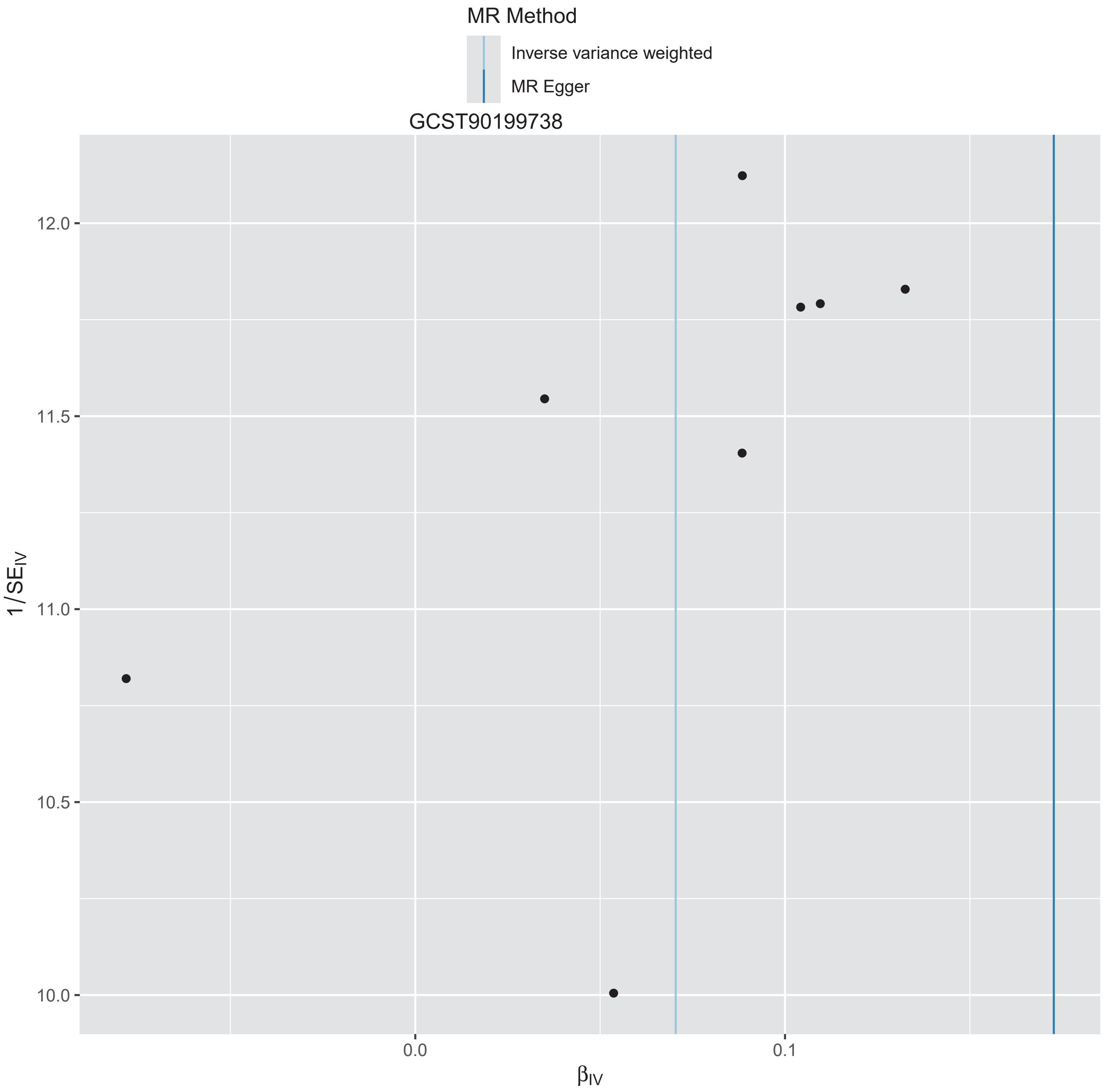

MR Method
Inverse variance weighted
MR Egger
GCST90199738
12.0
11.5
11.0
10.5
10.0
1/SE_IV
0.0
0.1
β_IV

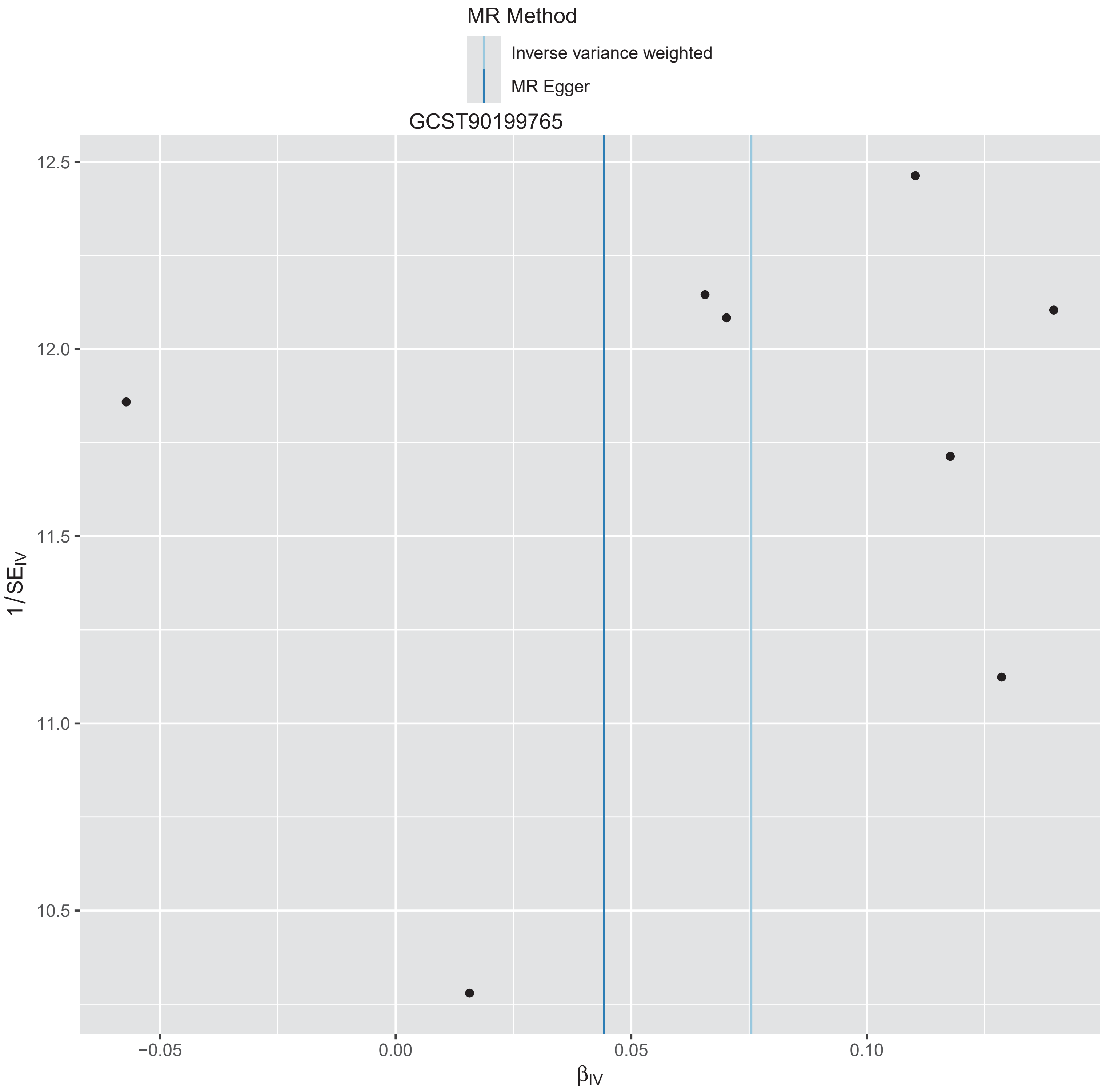
MR Method
Inverse variance weighted
MR Egger
GCST90199765
12.5
12.0
11.5
11.0
10.5
1/SE_IV
−0.05
0.00
0.05
0.10
β_IV

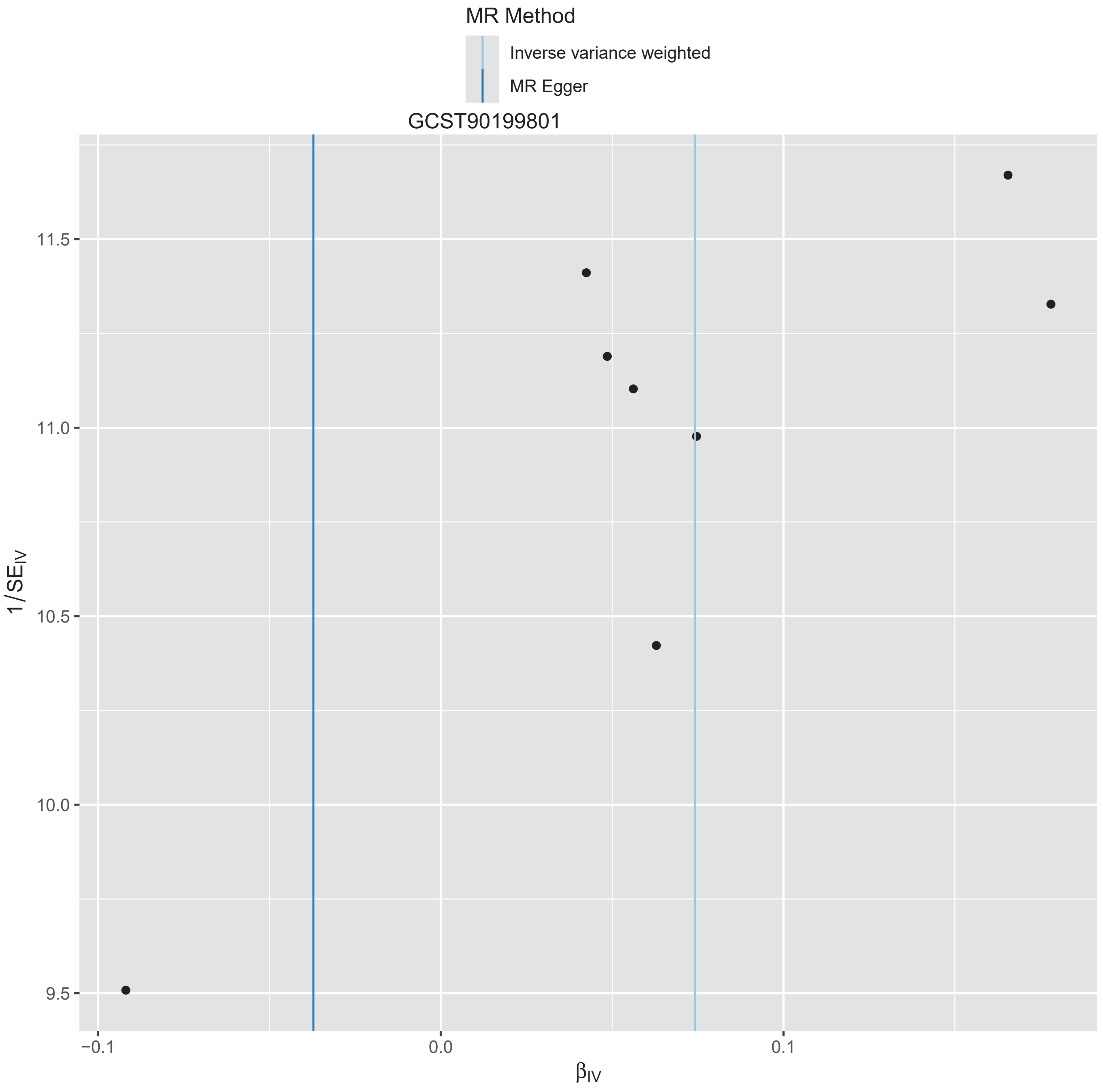
MR Method
Inverse variance weighted
MR Egger
GCST90199801
11.5
11.0
10.5
10.0
9.5
1/SE_IV
−0.1
0.0
0.1
β_IV

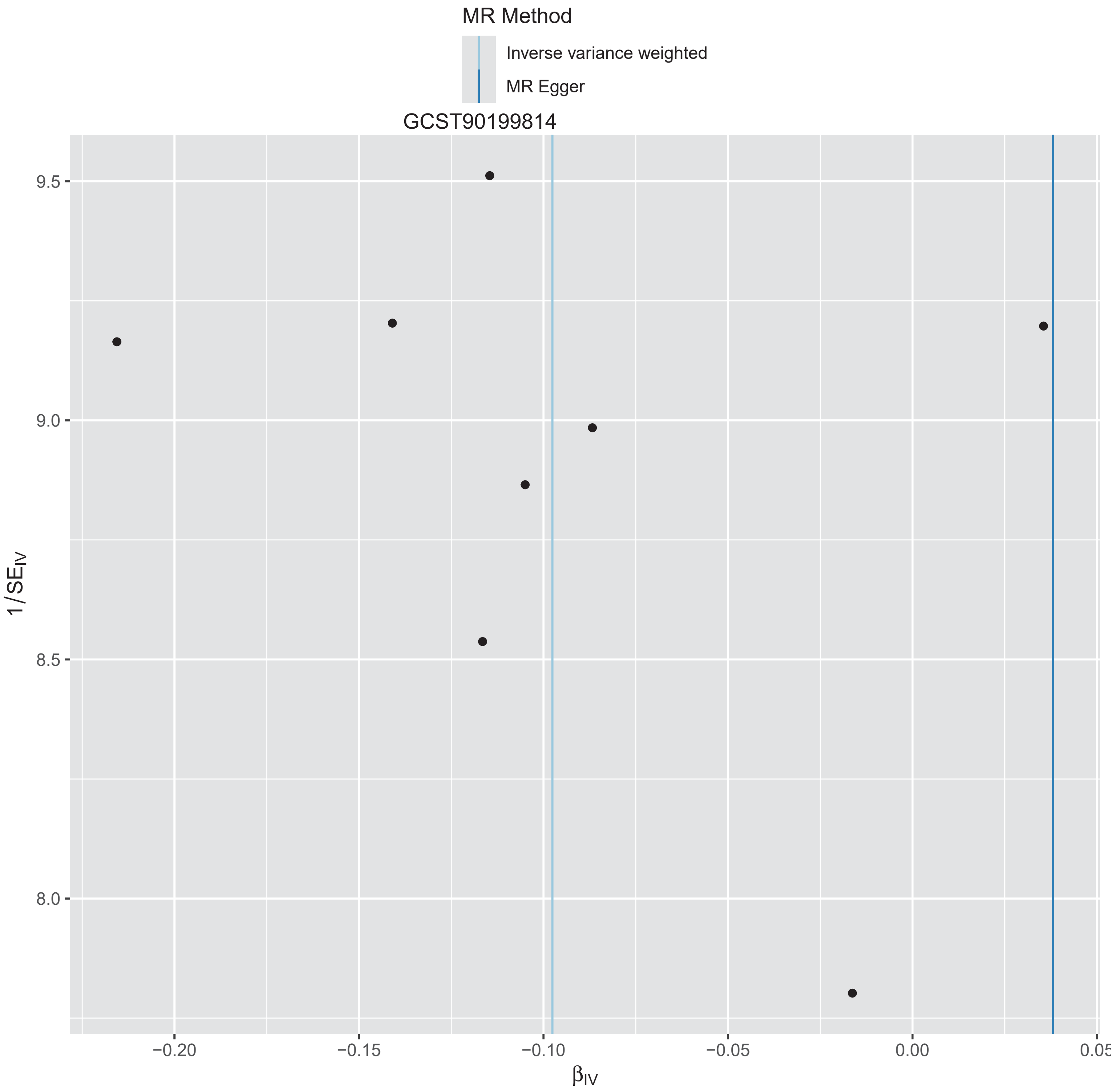

MR Method
Inverse variance weighted
MR Egger
GCST90199814
9.5
9.0
8.5
8.0
1/SE_IV
−0.20
−0.15
−0.10
−0.05
0.00
0.05
β_IV

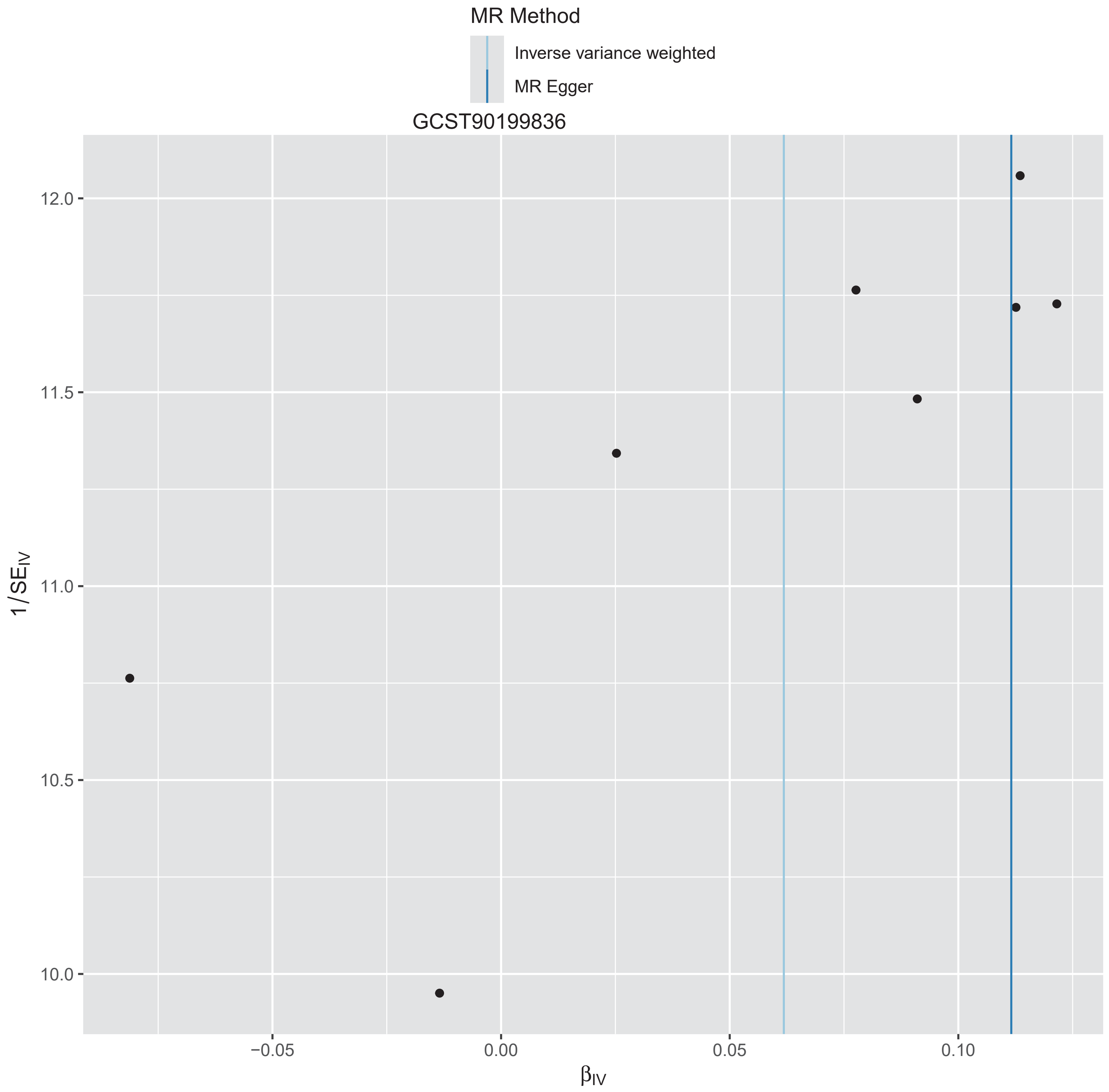

MR Method
Inverse variance weighted
MR Egger
GCST90199836
12.0
11.5
11.0
10.5
10.0
1/SE$_{IV}$
−0.05
0.00
0.05
0.10
β$_{IV}$

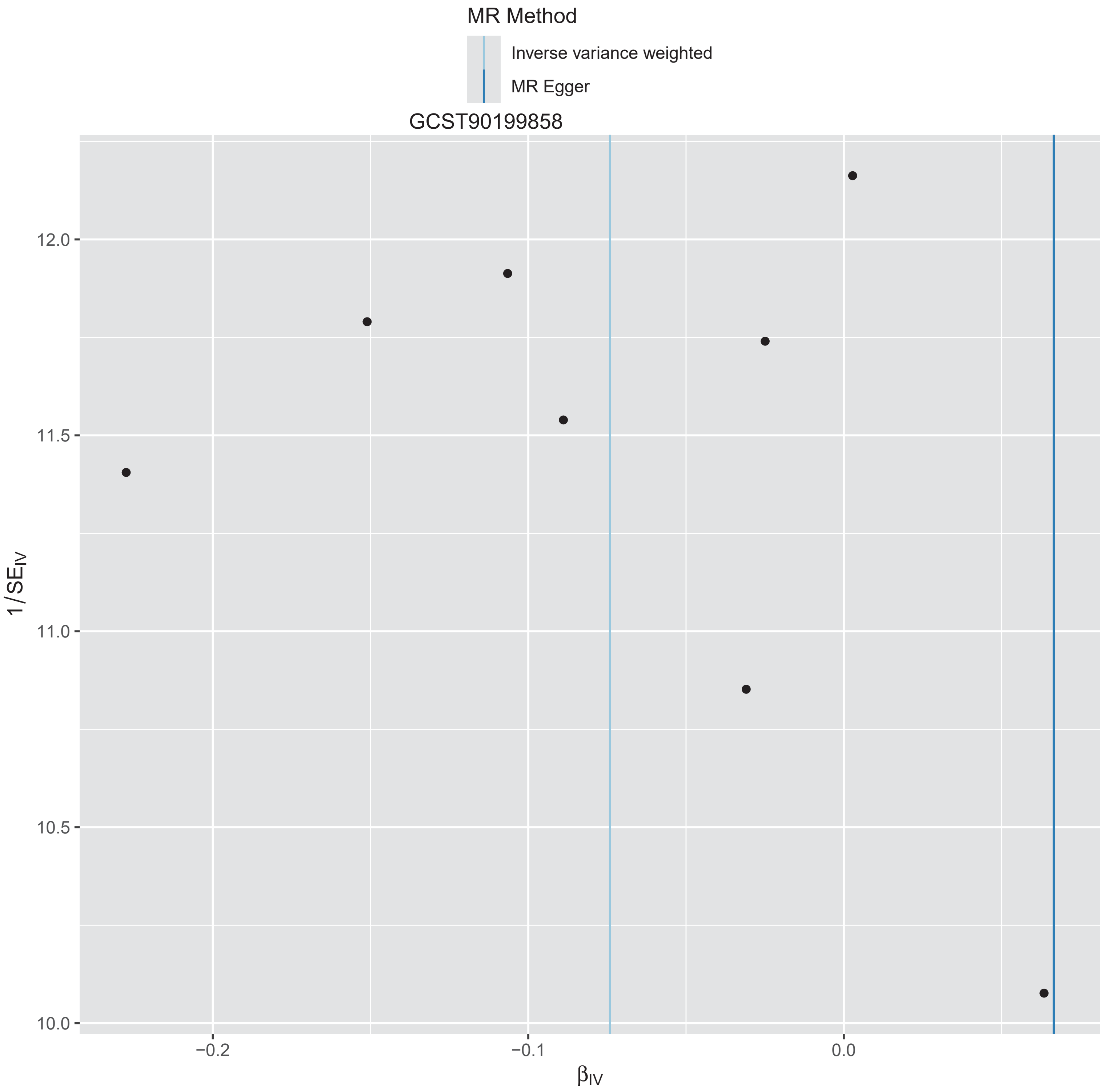
MR Method
Inverse variance weighted
MR Egger
GCST90199858
12.0
11.5
11.0
10.5
10.0
1/SE$_{IV}$
−0.2
−0.1
0.0
β_{IV}

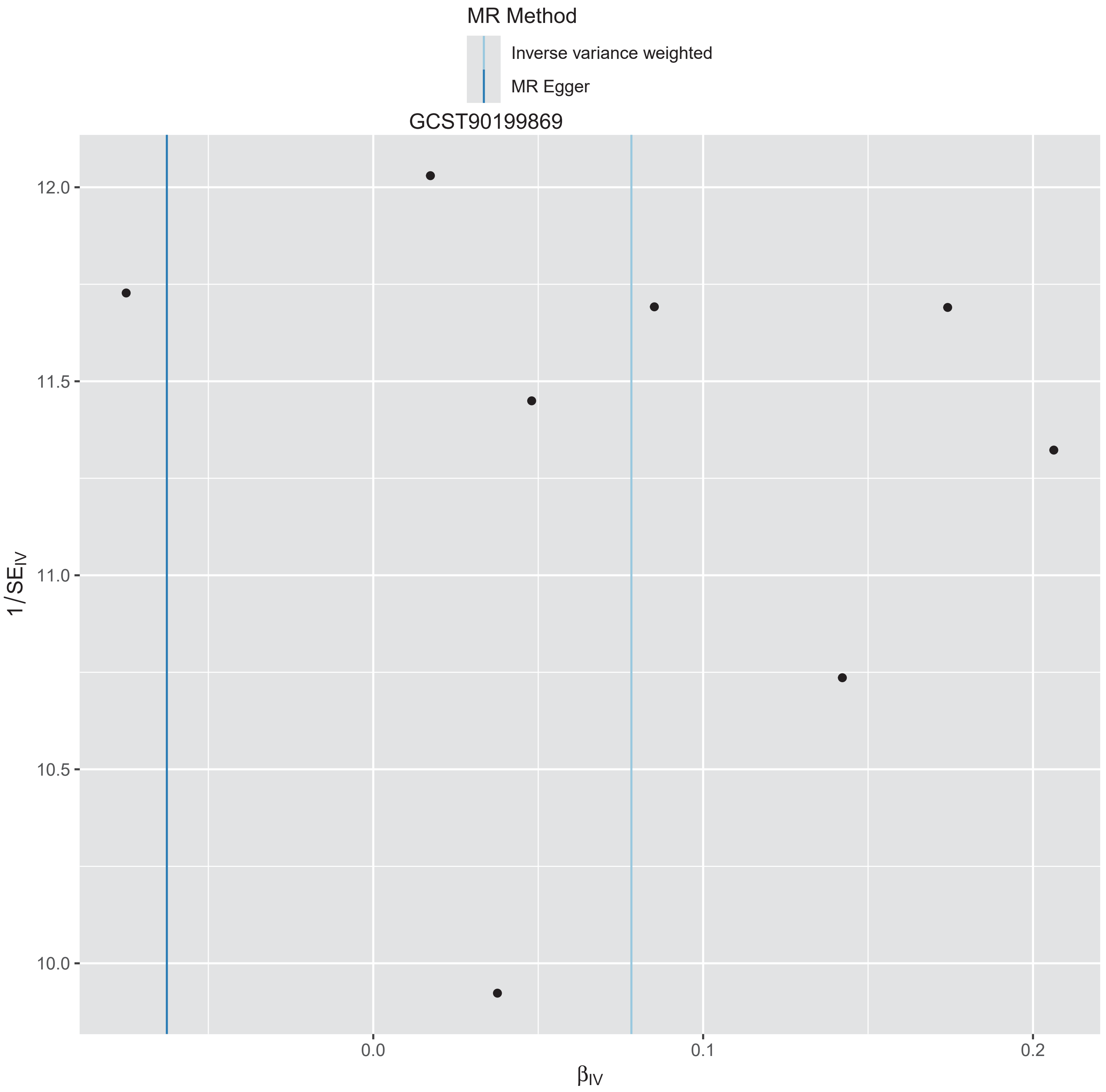

MR Method
Inverse variance weighted
MR Egger
GCST90199869
12.0
11.5
11.0
10.5
10.0
1/SE$_{IV}$
0.0
0.1
0.2
β$_{IV}$

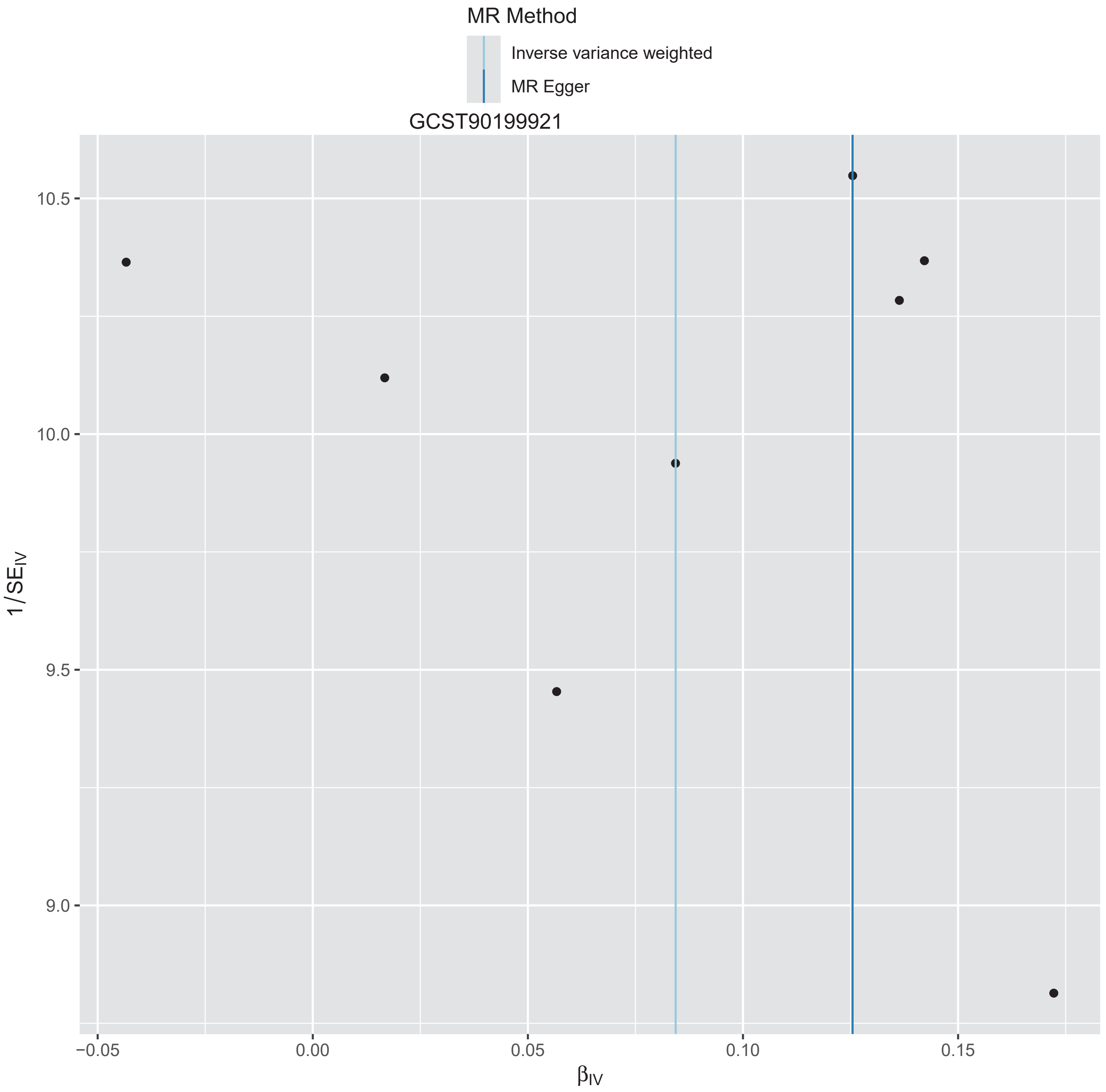

MR Method
Inverse variance weighted
MR Egger
GCST90199921
10.5
10.0
9.5
9.0
1/SE_IV
−0.05
0.00
0.05
0.10
0.15
β_IV

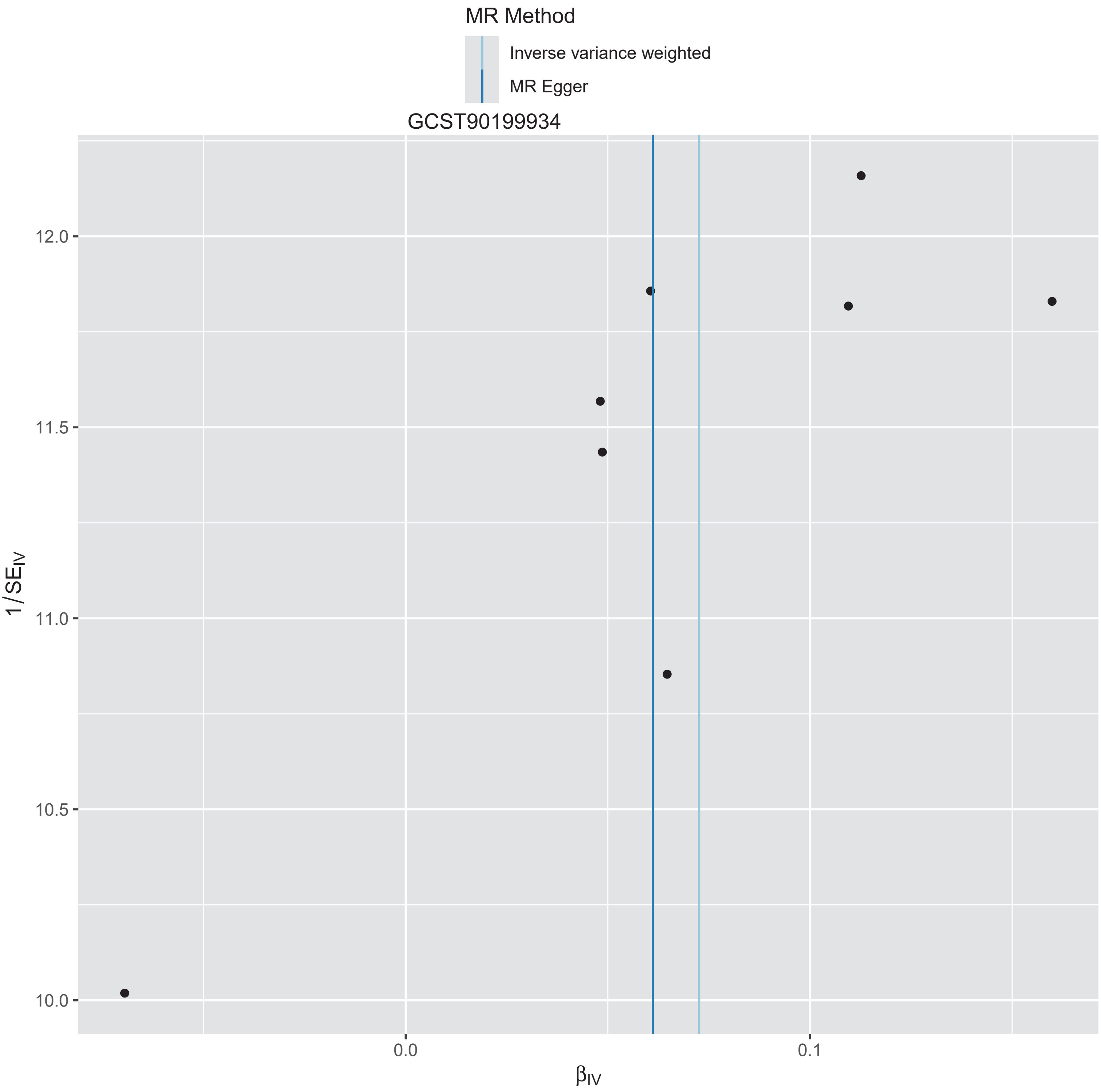

MR Method
Inverse variance weighted
MR Egger
GCST90199934
12.0
11.5
11.0
10.5
10.0
1/SE$_{IV}$
0.0
0.1
β$_{IV}$

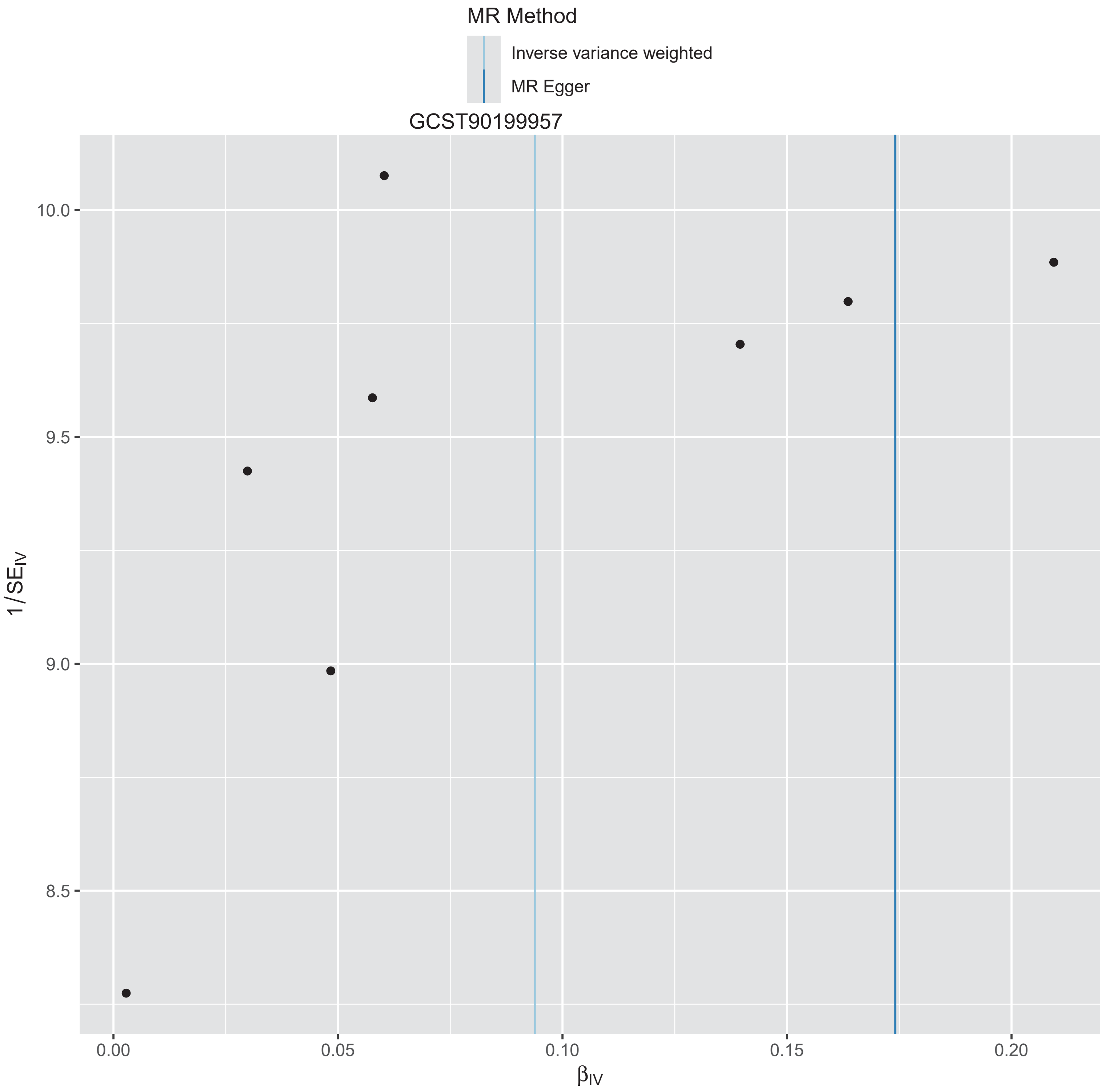
MR Method
Inverse variance weighted
MR Egger
GCST90199957
10.0
9.5
9.0
8.5
1/SE_IV
0.00
0.05
0.10
0.15
0.20
β_IV

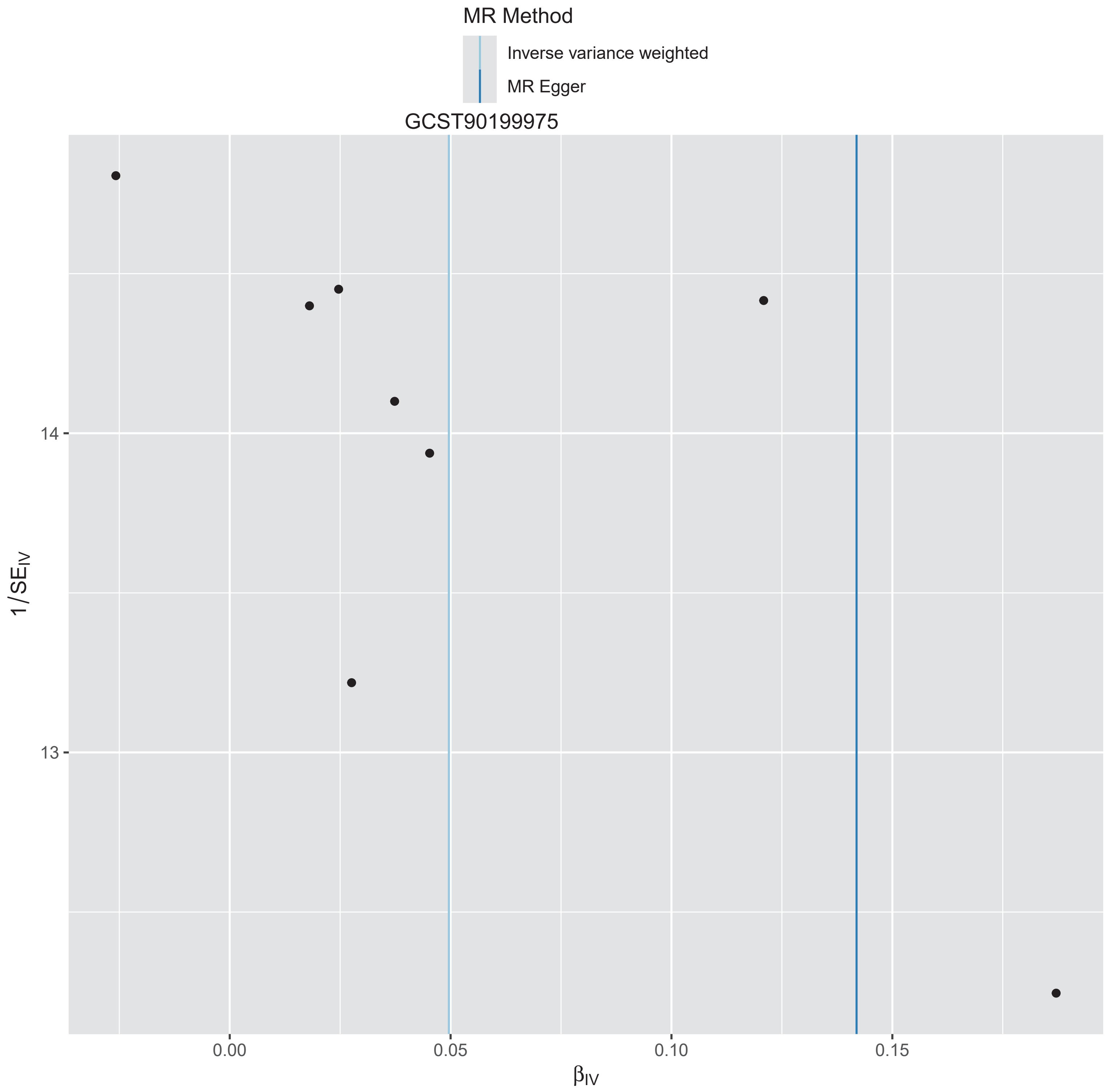

MR Method
Inverse variance weighted
MR Egger
GCST90199975
14
13
1/SE$_{IV}$
0.00
0.05
0.10
0.15
β$_{IV}$

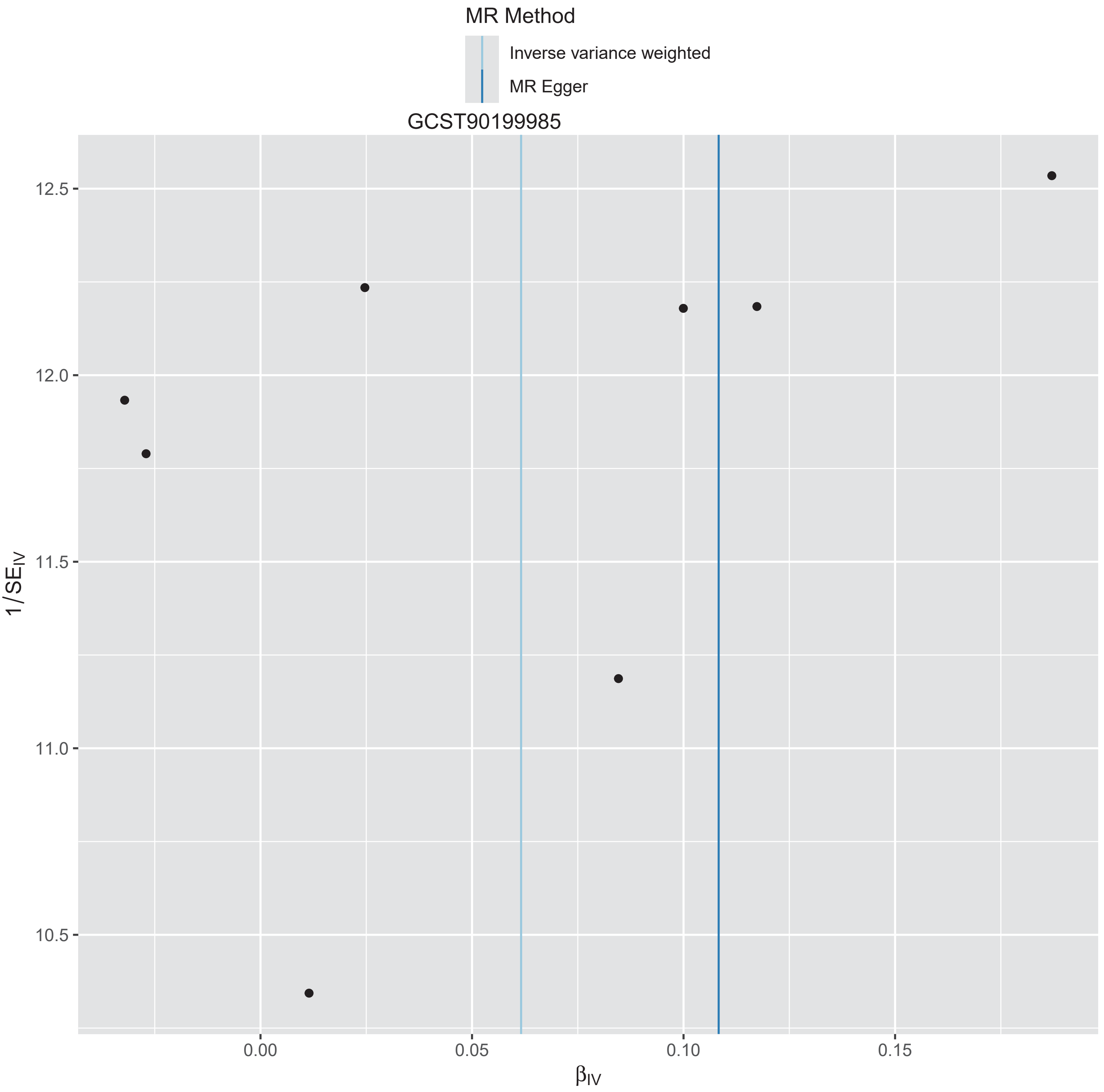

MR Method
Inverse variance weighted
MR Egger
GCST90199985
12.5
12.0
11.5
11.0
10.5
1/SE$_{IV}$
0.00
0.05
0.10
0.15
β_{IV}

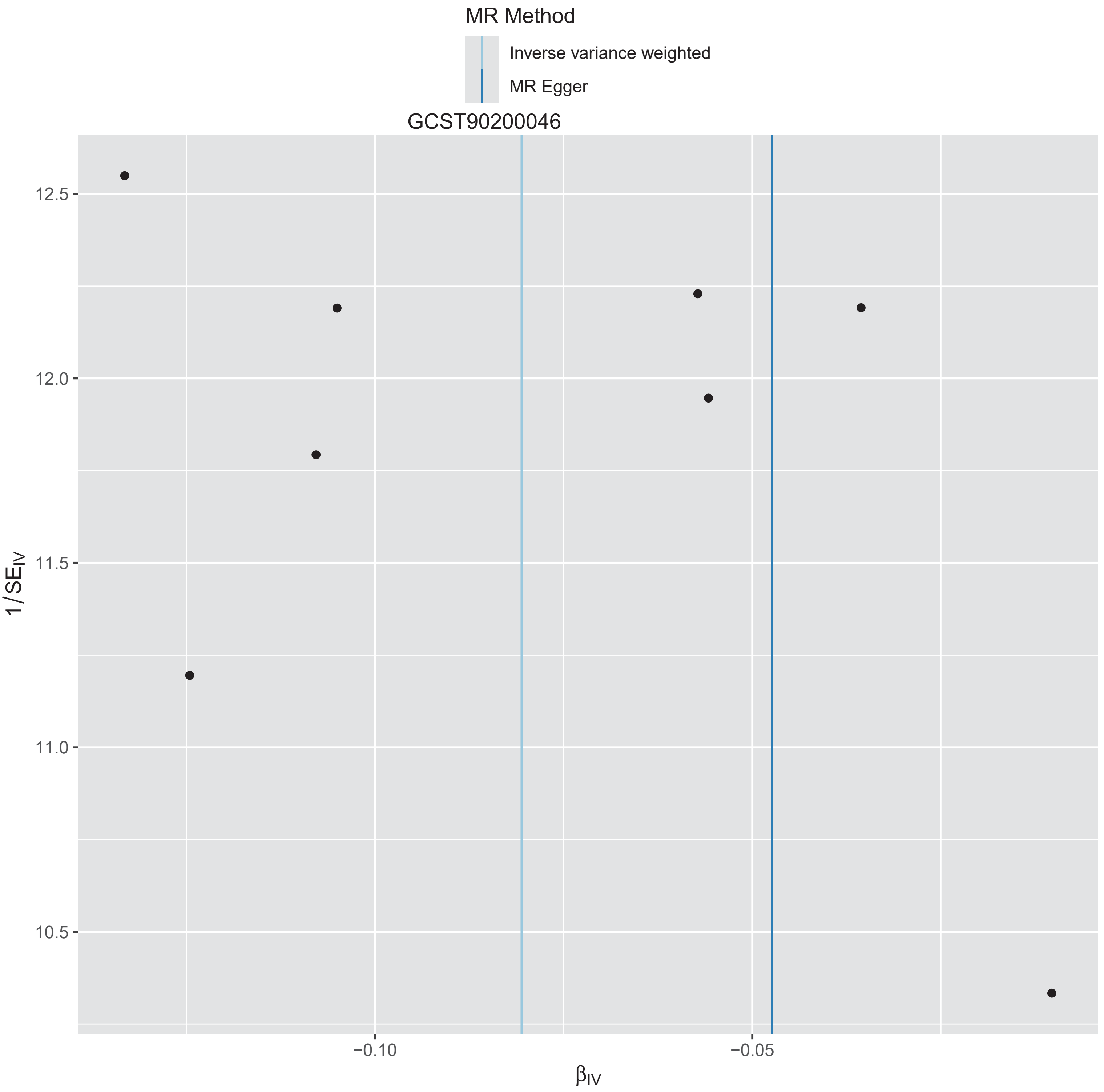
MR Method
Inverse variance weighted
MR Egger
GCST90200046
12.5
12.0
11.5
11.0
10.5
1/SE_IV
−0.10
−0.05
β_IV

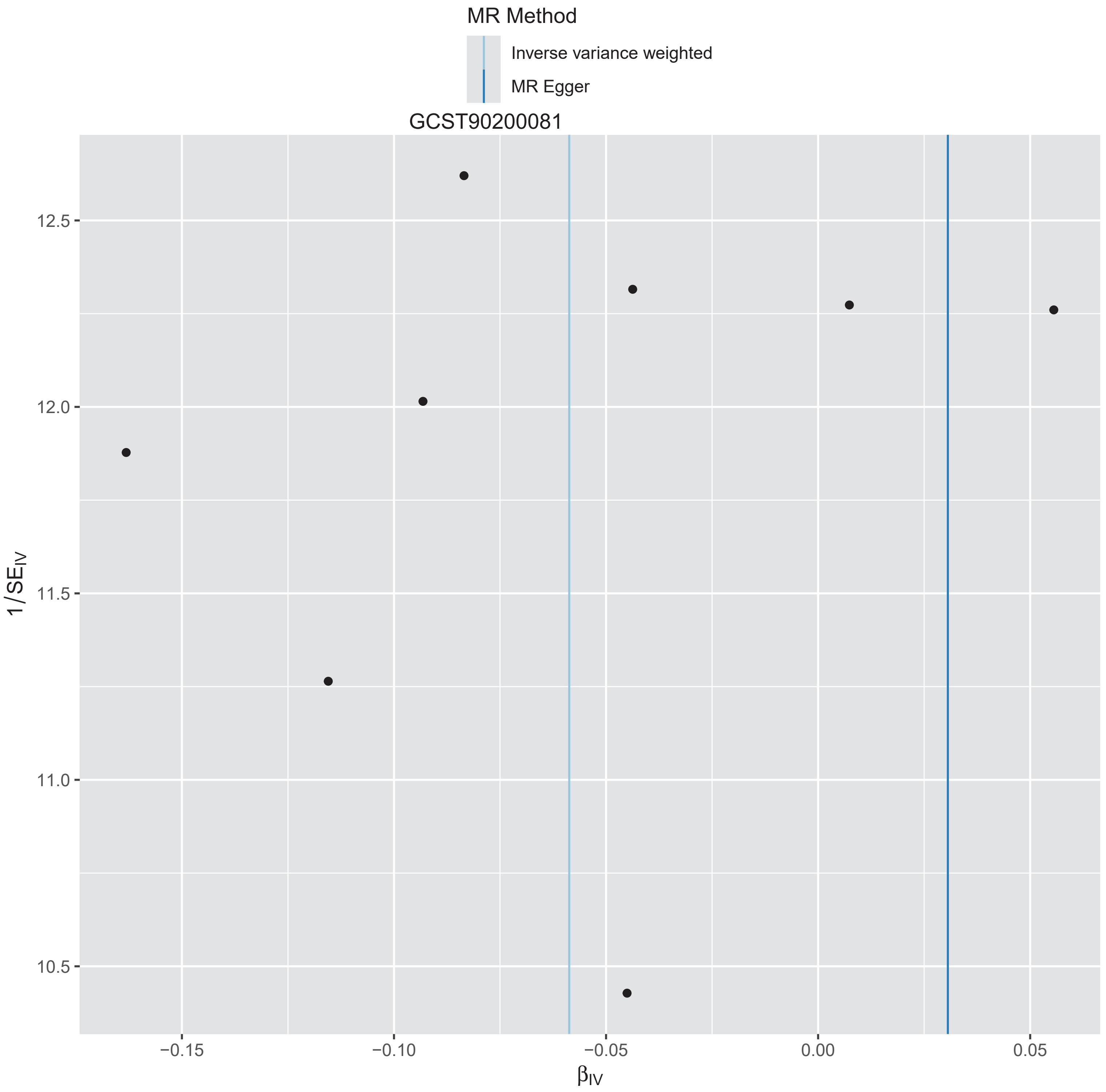
MR Method
Inverse variance weighted
MR Egger
GCST90200081
12.5
12.0
11.5
11.0
10.5
1/SE$_{IV}$
−0.15
−0.10
−0.05
0.00
0.05
β$_{IV}$

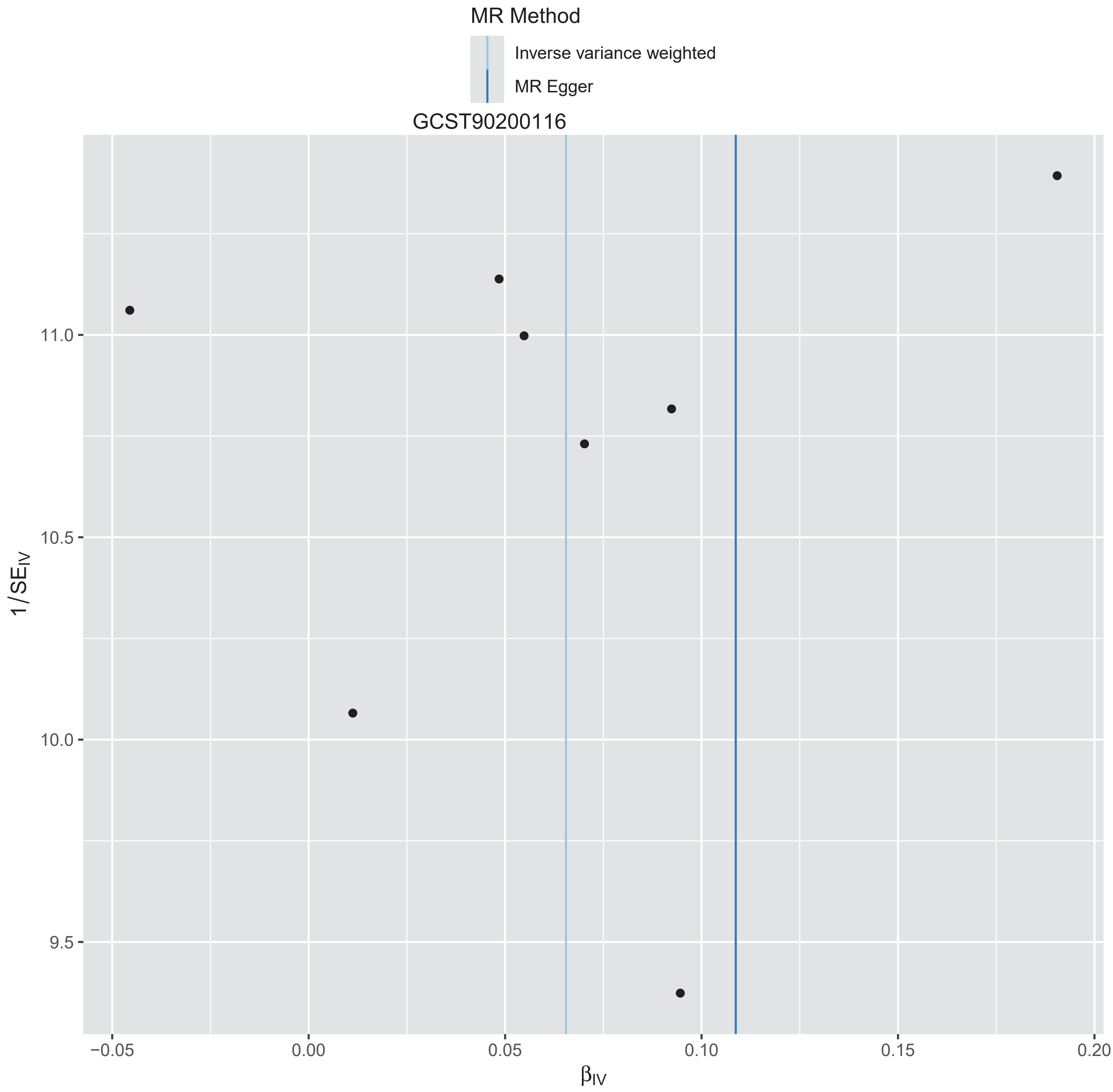

MR Method
Inverse variance weighted
MR Egger
GCST90200116
11.0
10.5
10.0
9.5
1/SE_IV
−0.05
0.00
0.05
0.10
0.15
0.20
β_IV

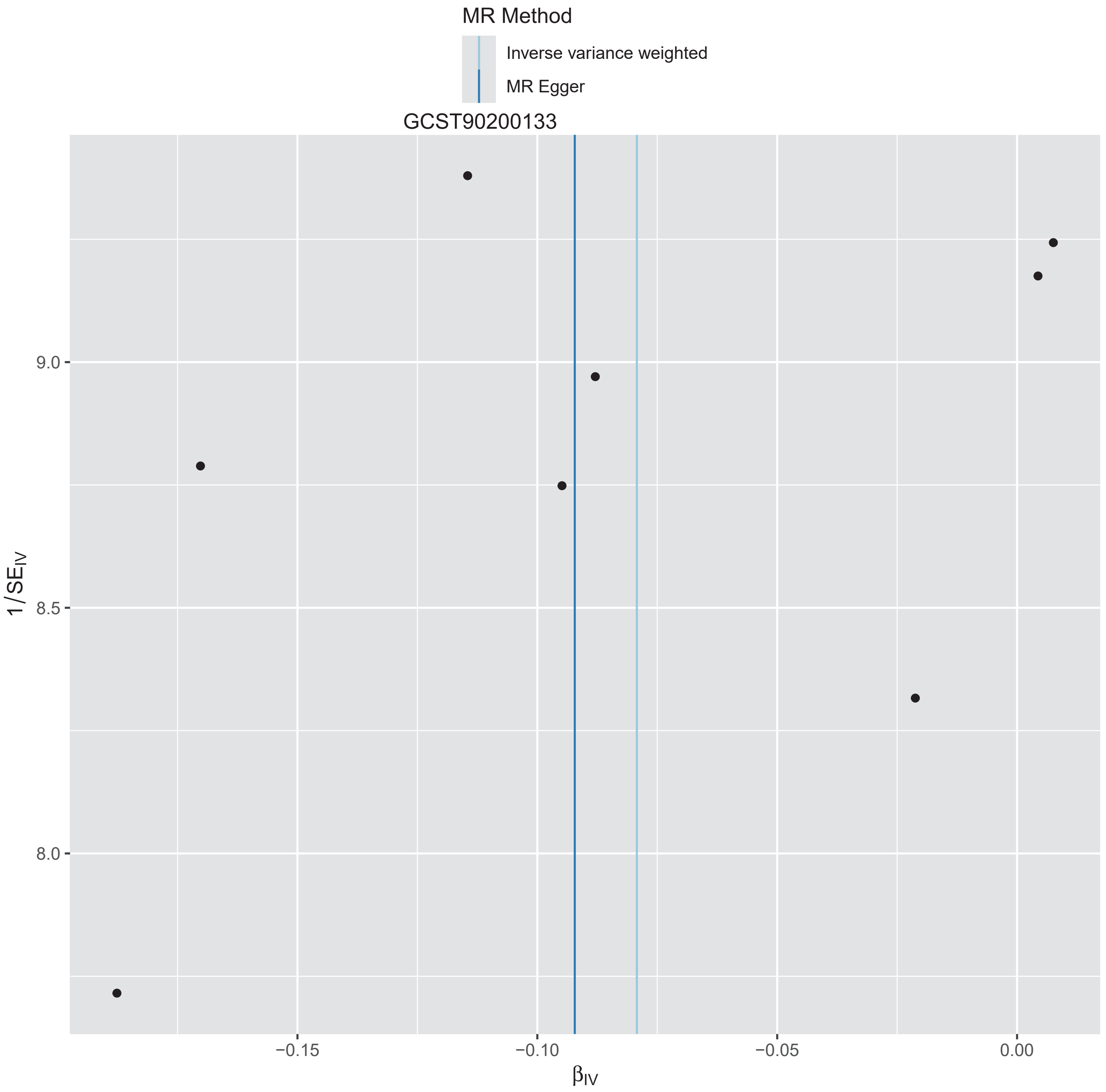

MR Method
Inverse variance weighted
MR Egger
GCST90200133
9.0
8.5
8.0
1/SE_IV
−0.15
−0.10
−0.05
0.00
β_IV

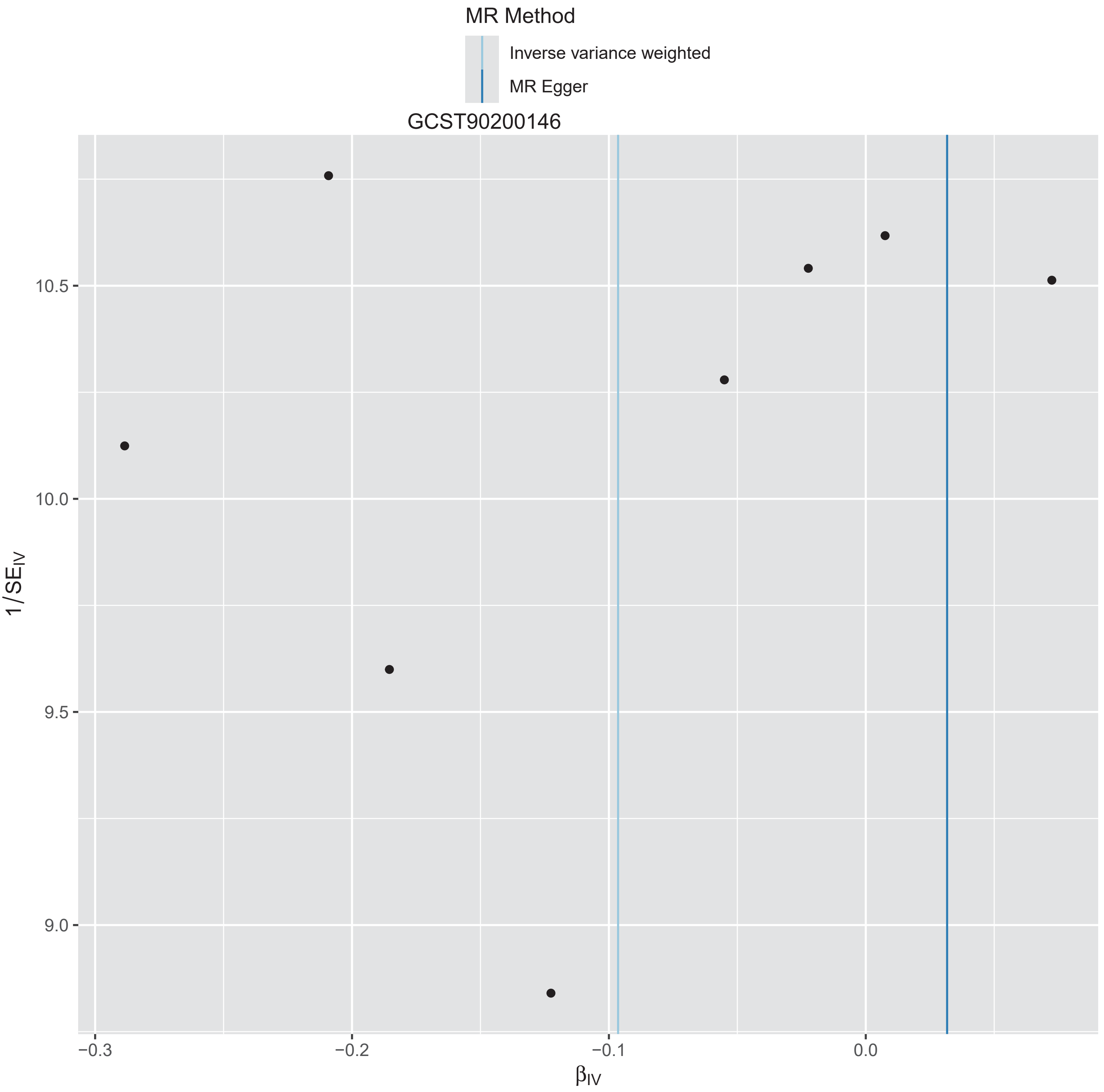
MR Method
Inverse variance weighted
MR Egger
GCST90200146
1/SE_IV
β_IV
10.5
10.0
9.5
9.0
−0.3
−0.2
−0.1
0.0

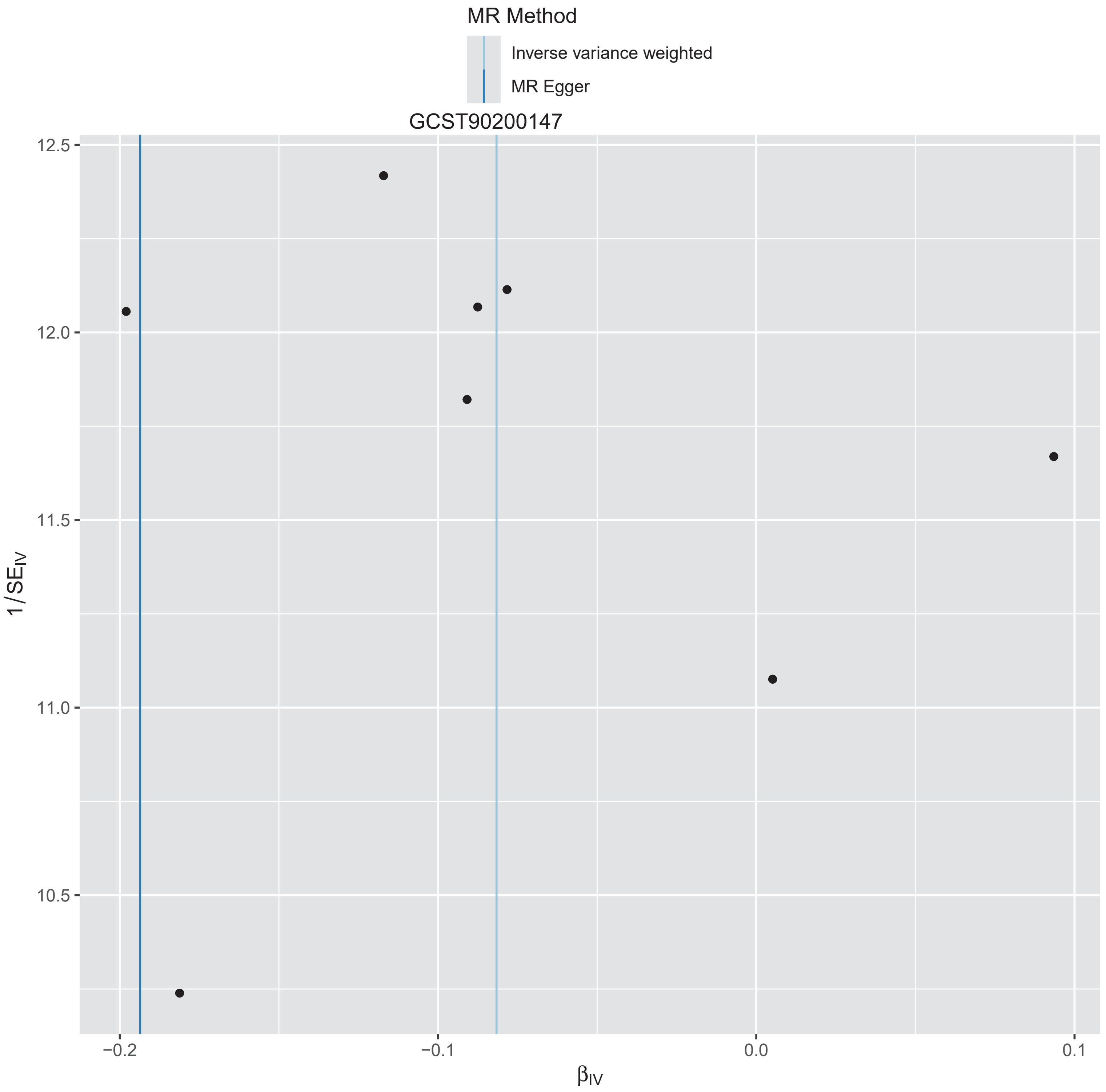

MR Method
Inverse variance weighted
MR Egger
GCST90200147
12.5
12.0
11.5
11.0
10.5
1/SE_IV
−0.2
−0.1
0.0
0.1
β_IV

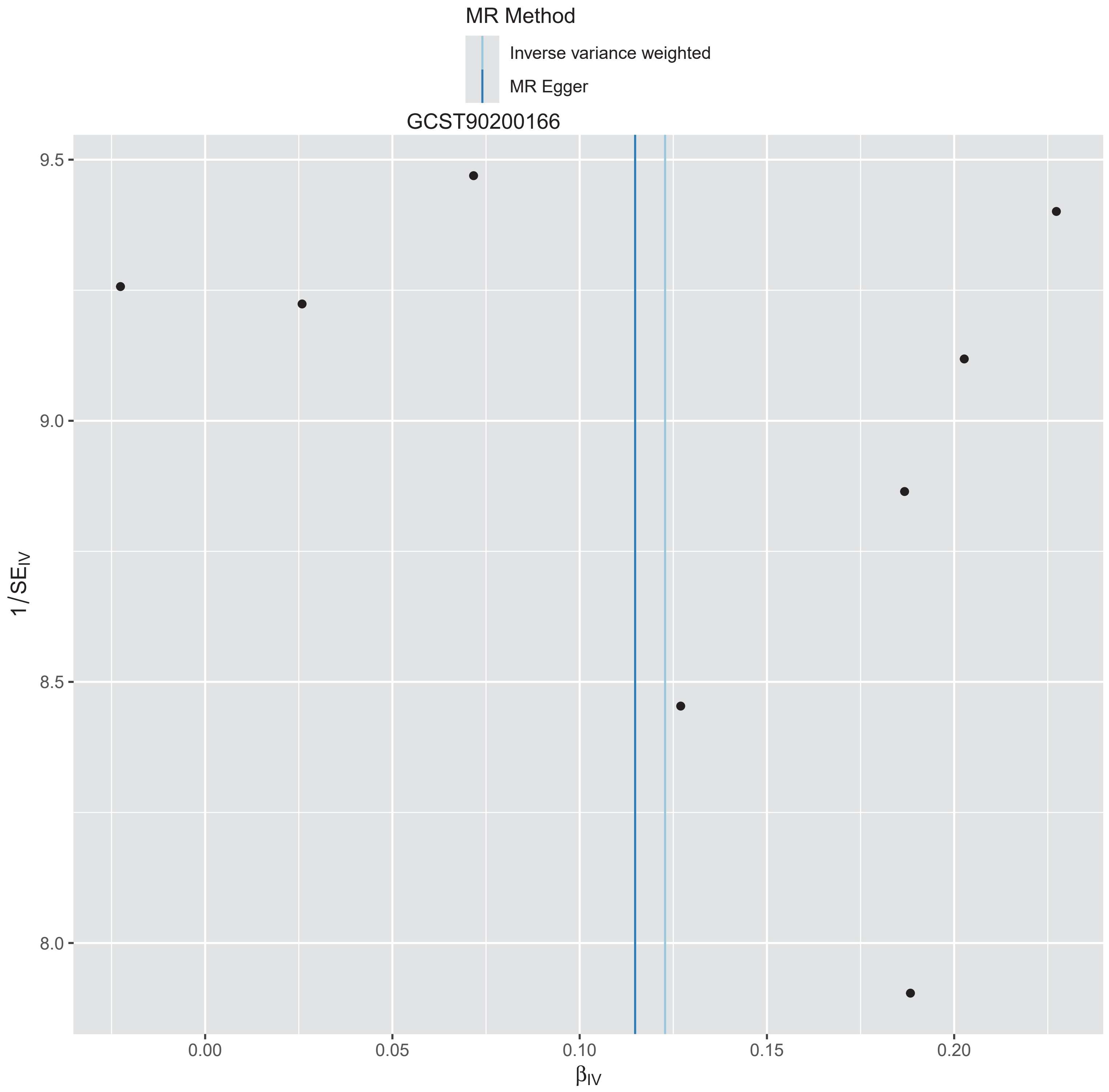

MR Method
Inverse variance weighted
MR Egger
GCST90200166
9.5
9.0
8.5
8.0
1/SE_IV
0.00
0.05
0.10
0.15
0.20
β_IV

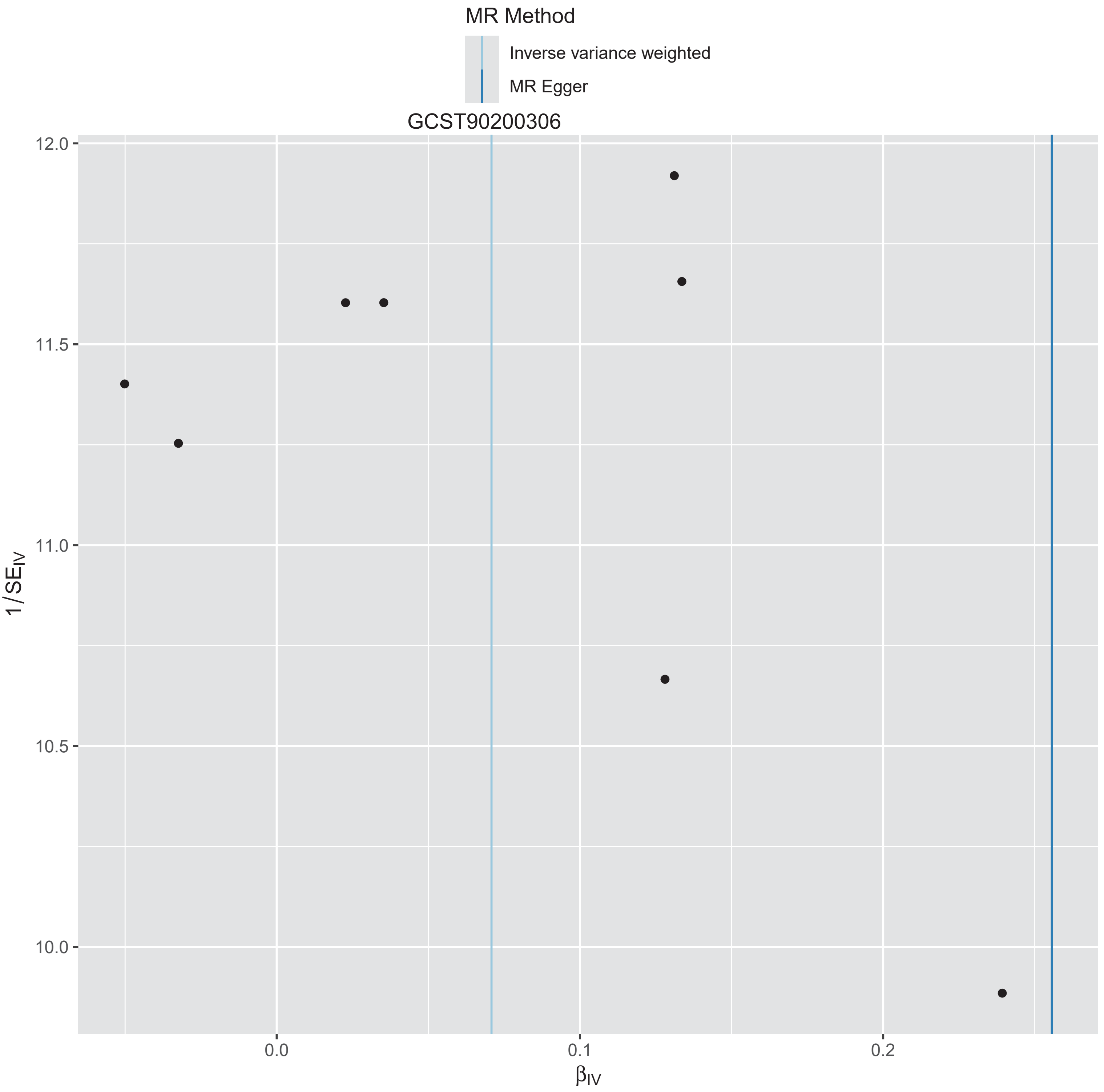
MR Method
Inverse variance weighted
MR Egger
GCST90200306
12.0
11.5
11.0
10.5
10.0
1/SE_IV
0.0
0.1
0.2
β_IV

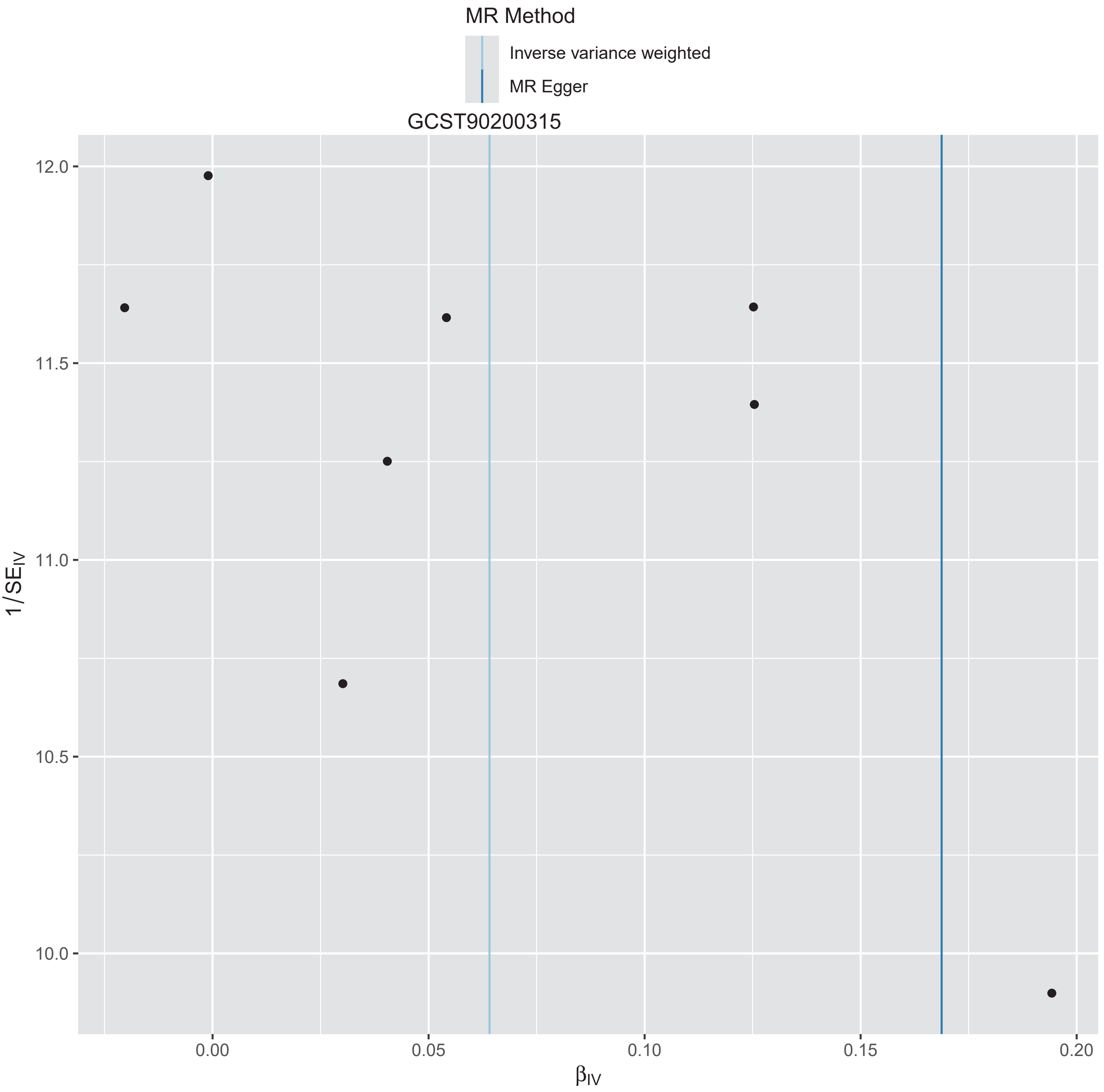
MR Method
Inverse variance weighted
MR Egger
GCST90200315
12.0
11.5
11.0
10.5
10.0
1/SE$_{IV}$
0.00
0.05
0.10
0.15
0.20
β_{IV}

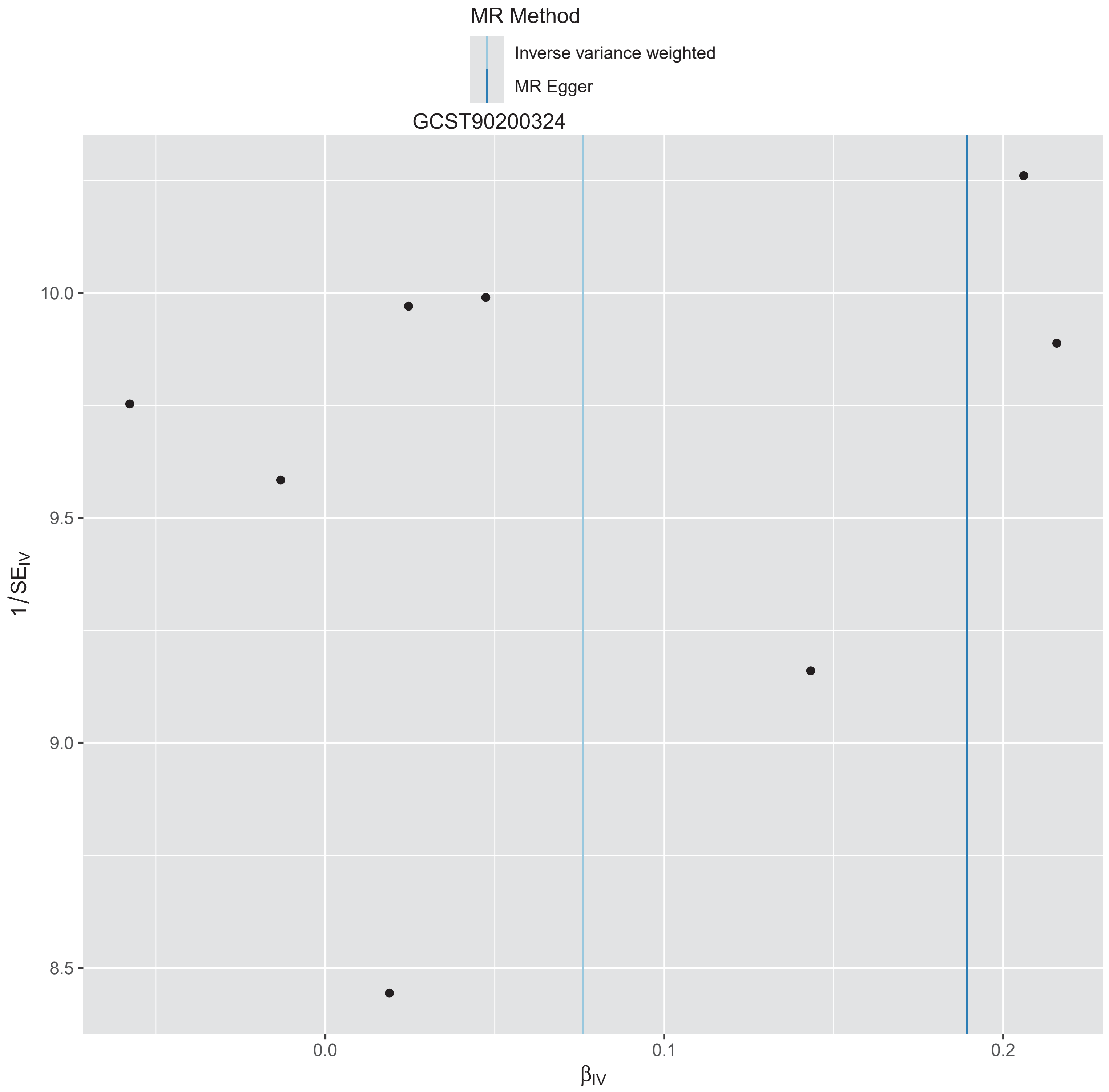

MR Method
Inverse variance weighted
MR Egger
GCST90200324
10.0
9.5
9.0
8.5
1/SE$_{IV}$
0.0
0.1
0.2
β_{IV}

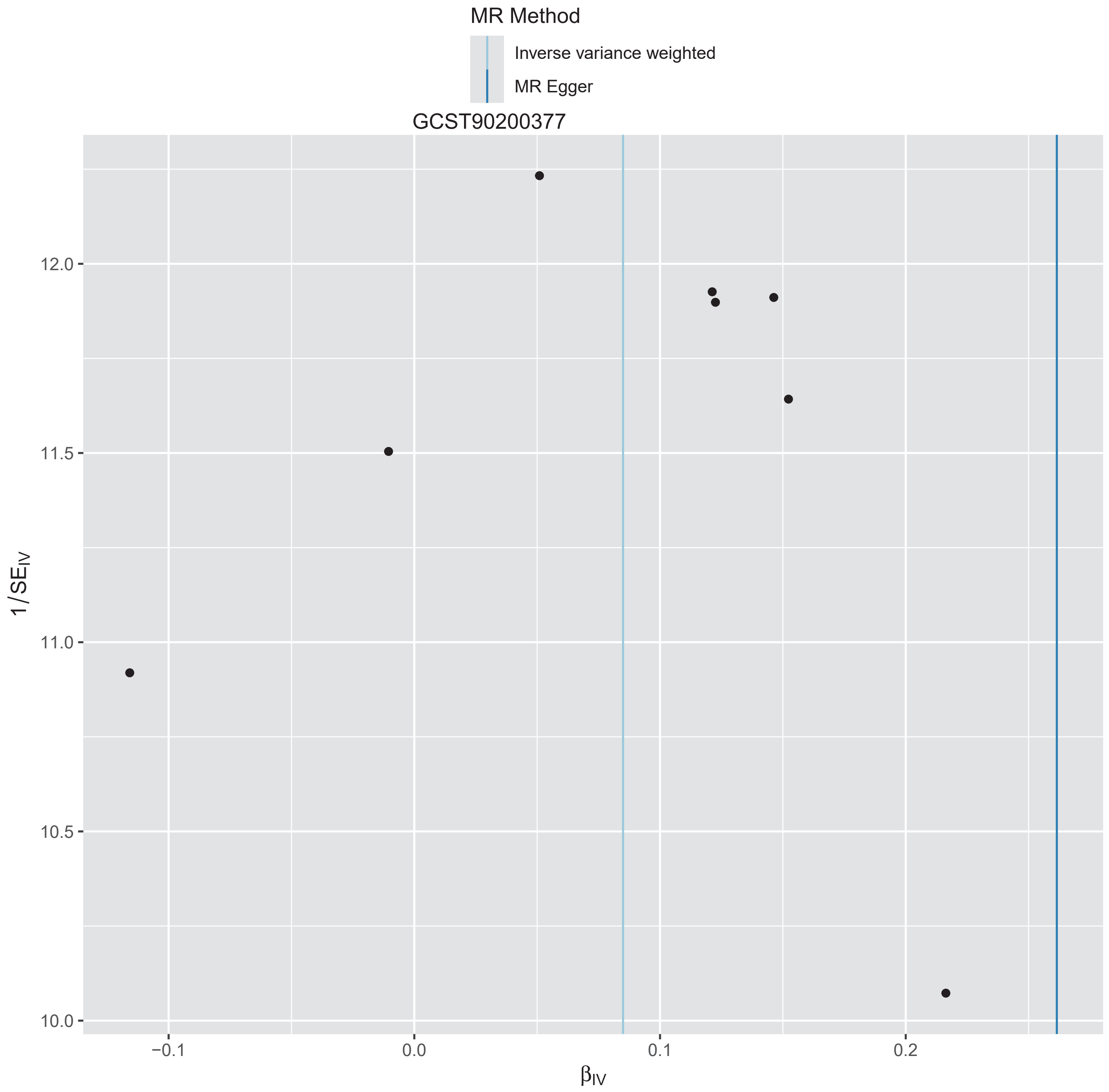
MR Method
Inverse variance weighted
MR Egger
GCST90200377
12.0
11.5
11.0
10.5
10.0
1/SE_IV
−0.1
0.0
0.1
0.2
β_IV

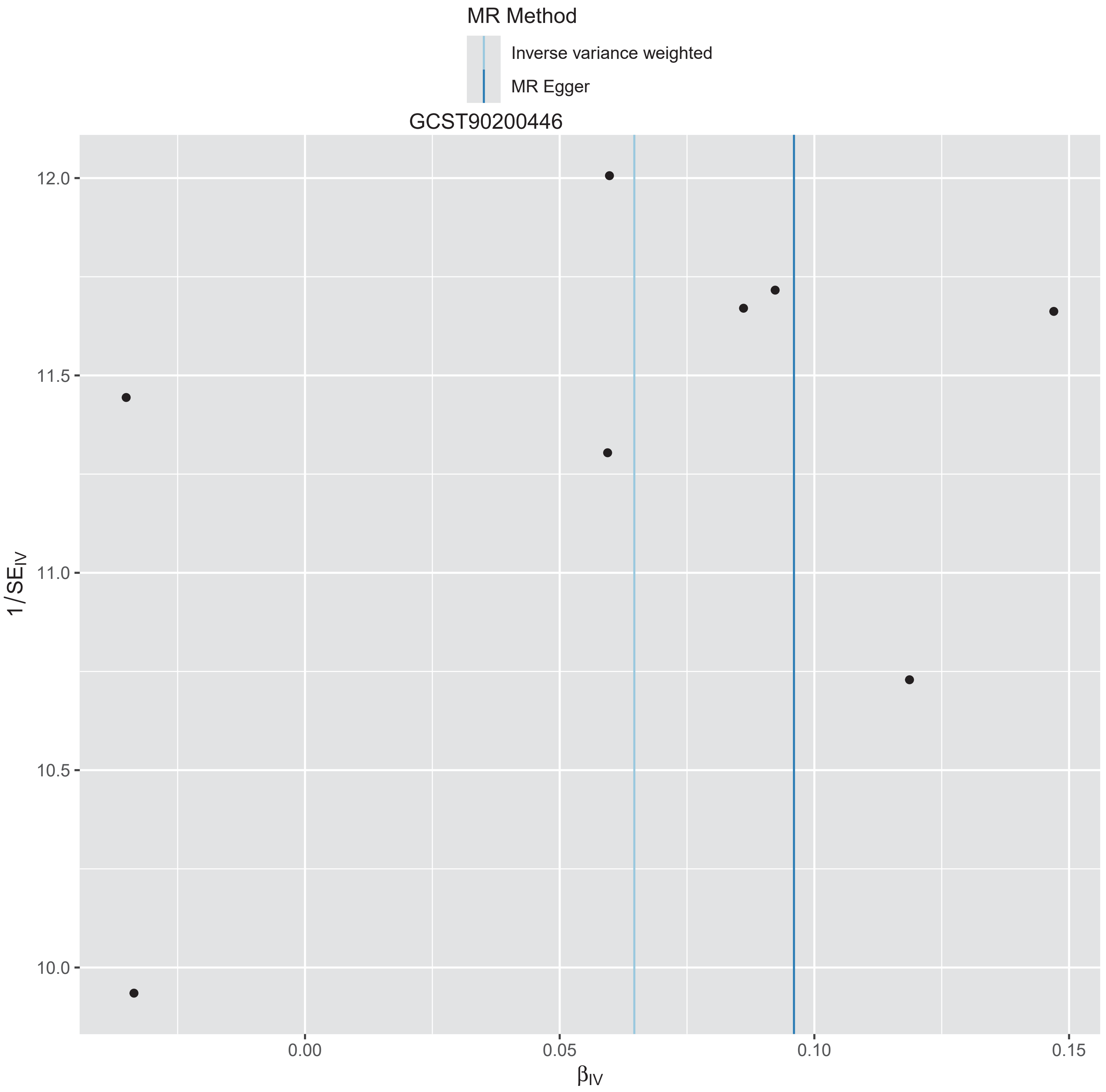
MR Method
Inverse variance weighted
MR Egger
GCST90200446
12.0
11.5
11.0
10.5
10.0
1/SE_IV
0.00
0.05
0.10
0.15
β_IV

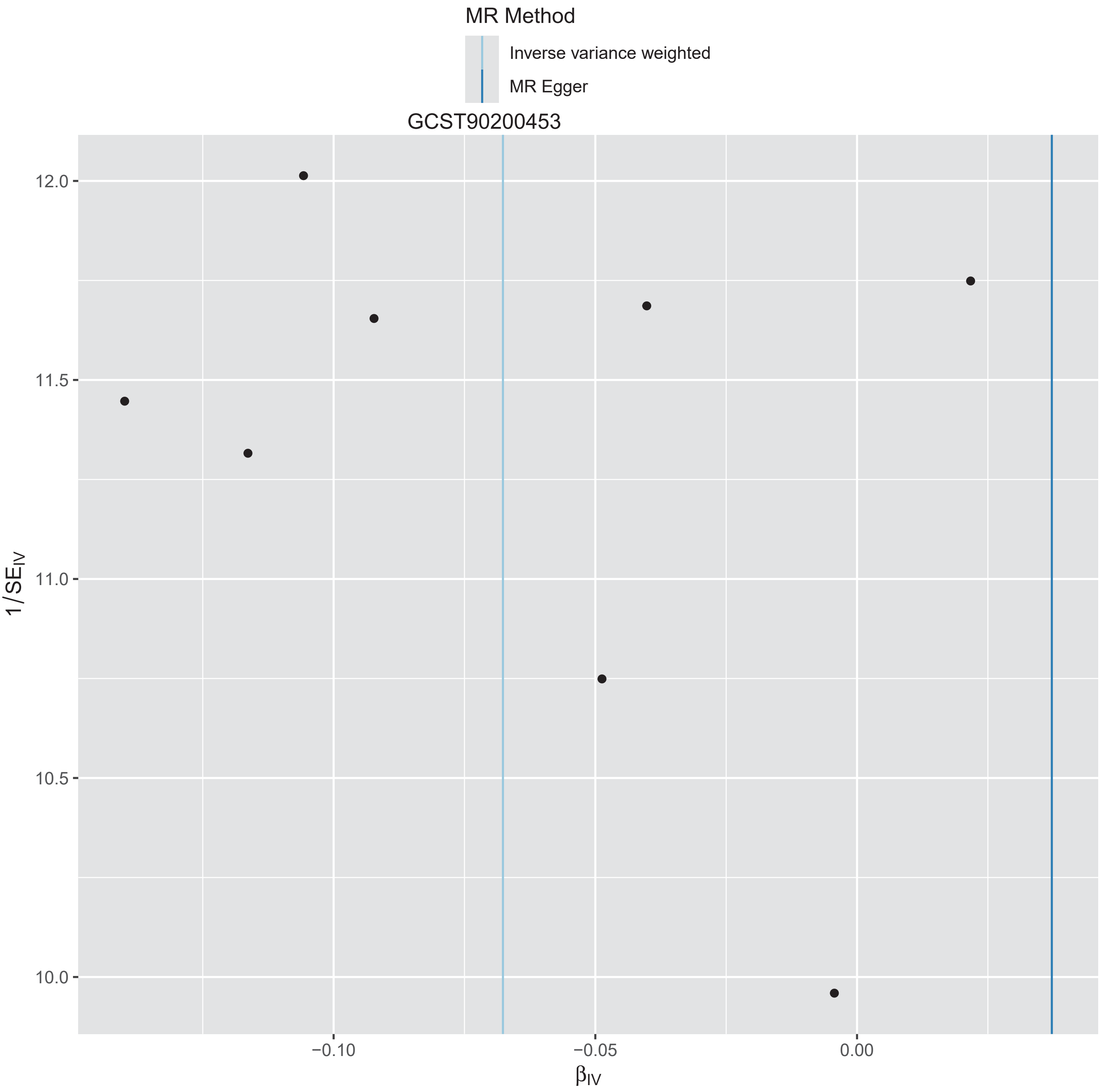

MR Method
Inverse variance weighted
MR Egger
GCST90200453
12.0
11.5
11.0
10.5
10.0
1/SE_IV
−0.10
−0.05
0.00
β_IV

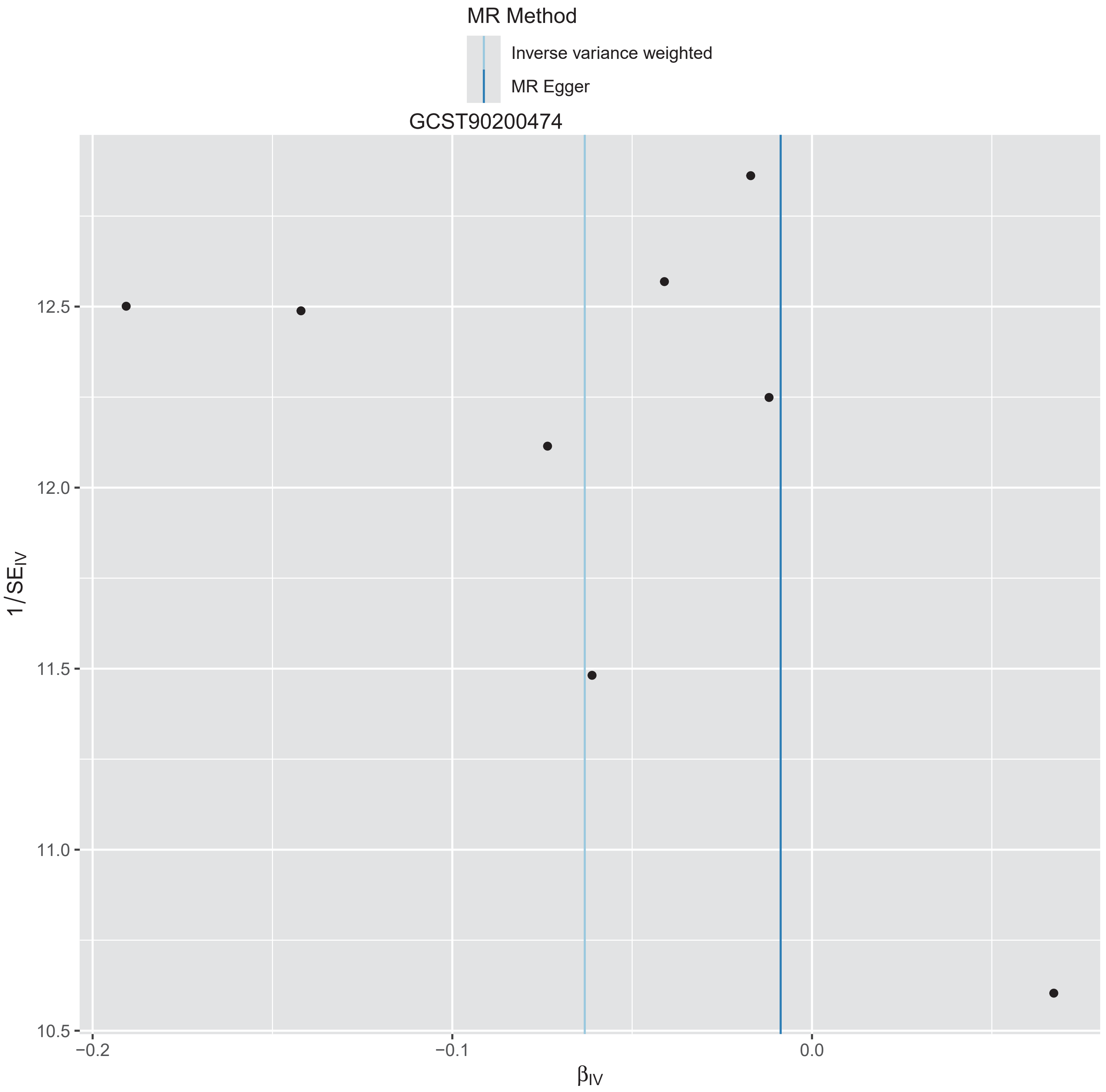

MR Method
Inverse variance weighted
MR Egger
GCST90200474
12.5
12.0
11.5
11.0
10.5
1/SE$_{IV}$
−0.2
−0.1
0.0
β$_{IV}$

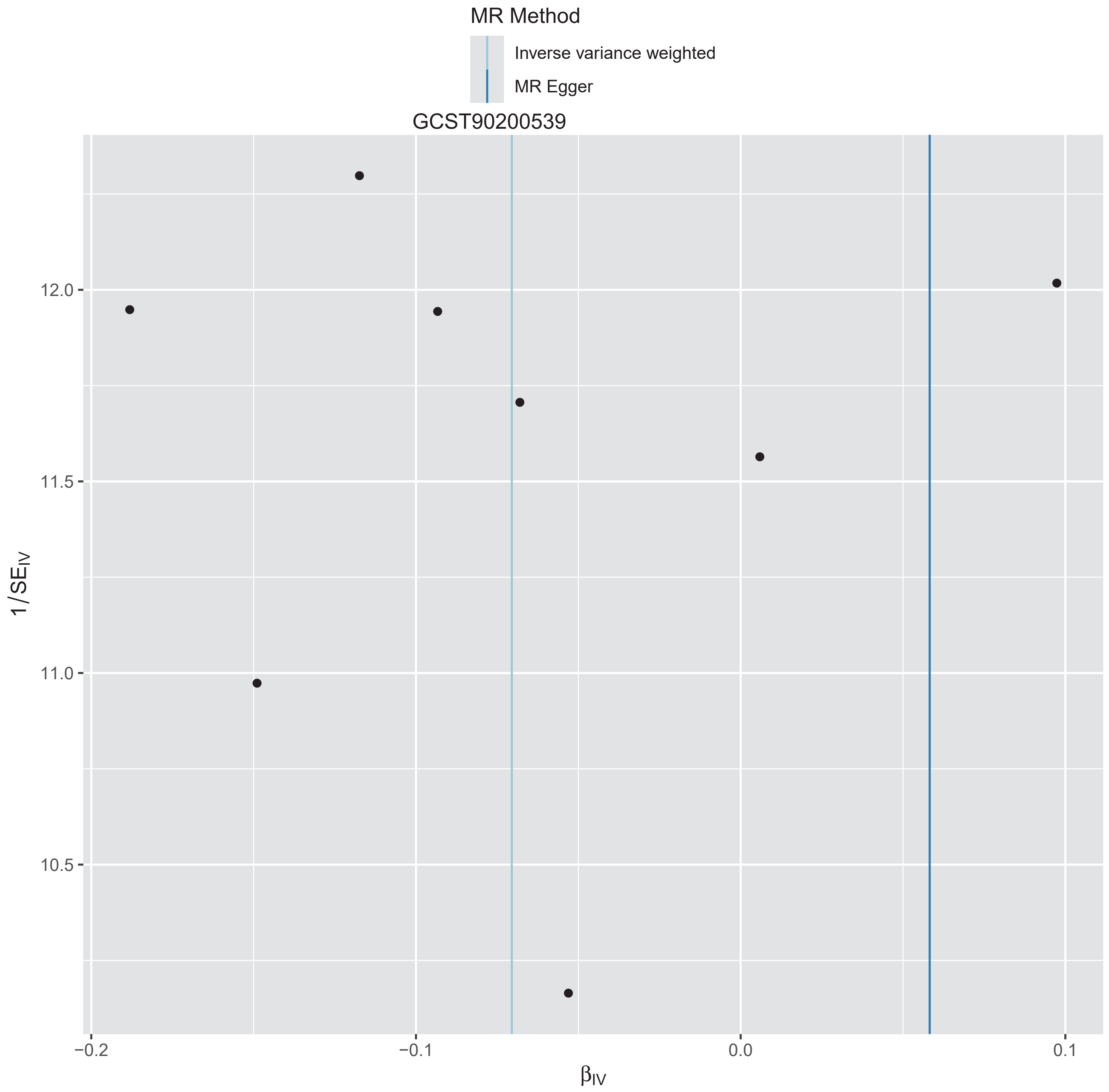

MR Method
Inverse variance weighted
MR Egger
GCST90200539
1/SE_IV
12.0
11.5
11.0
10.5
−0.2
−0.1
0.0
0.1
β_IV

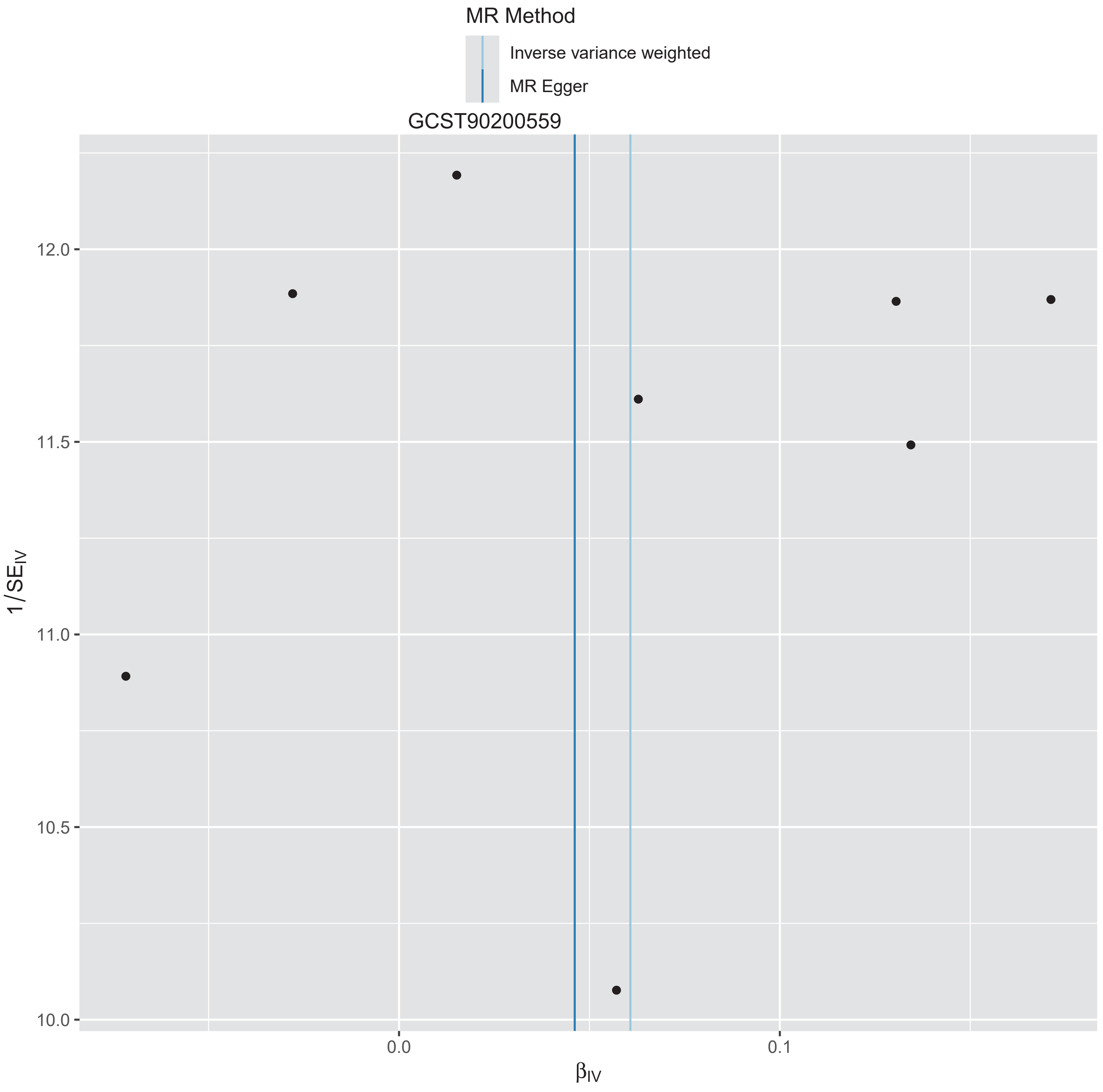

MR Method
Inverse variance weighted
MR Egger
GCST90200559
12.0
11.5
11.0
10.5
10.0
1/SE_IV
0.0
0.1
β_IV

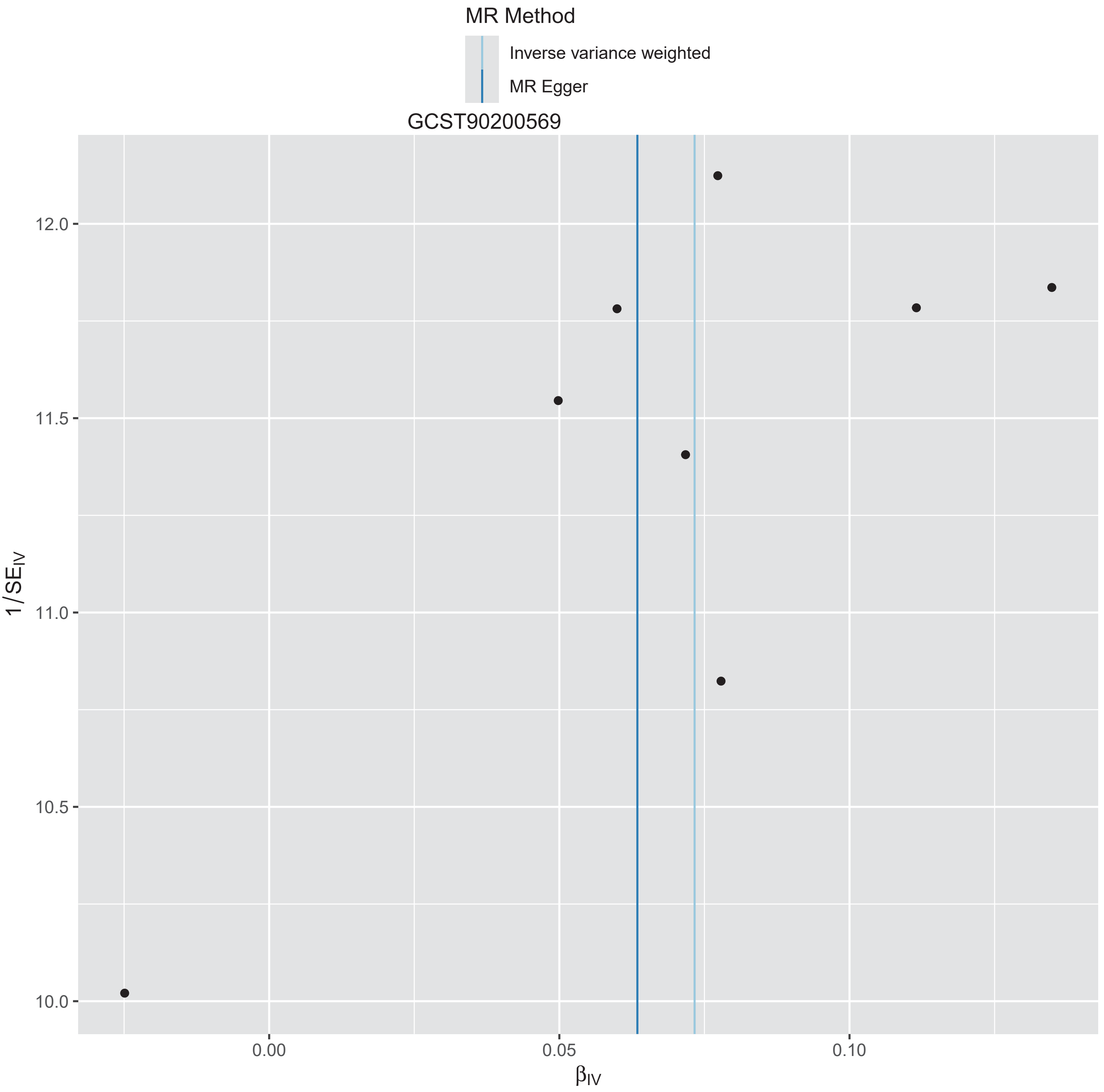

MR Method
Inverse variance weighted
MR Egger
GCST90200569
12.0
11.5
11.0
10.5
10.0
1/SE_IV
0.00
0.05
0.10
β_IV

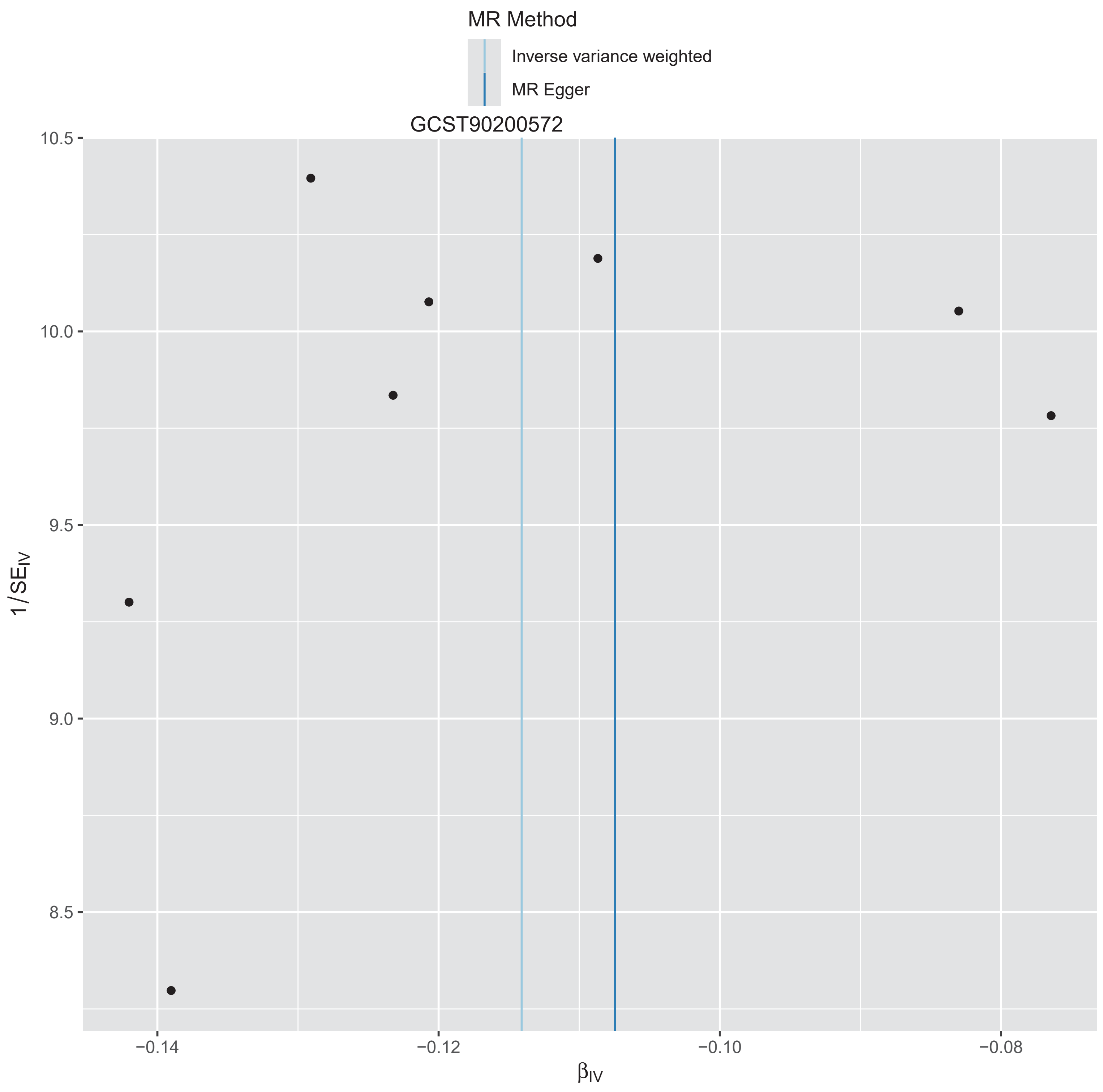
MR Method
Inverse variance weighted
MR Egger
GCST90200572
10.5
10.0
9.5
9.0
8.5
1/SE$_{IV}$
−0.14
−0.12
−0.10
−0.08
β$_{IV}$

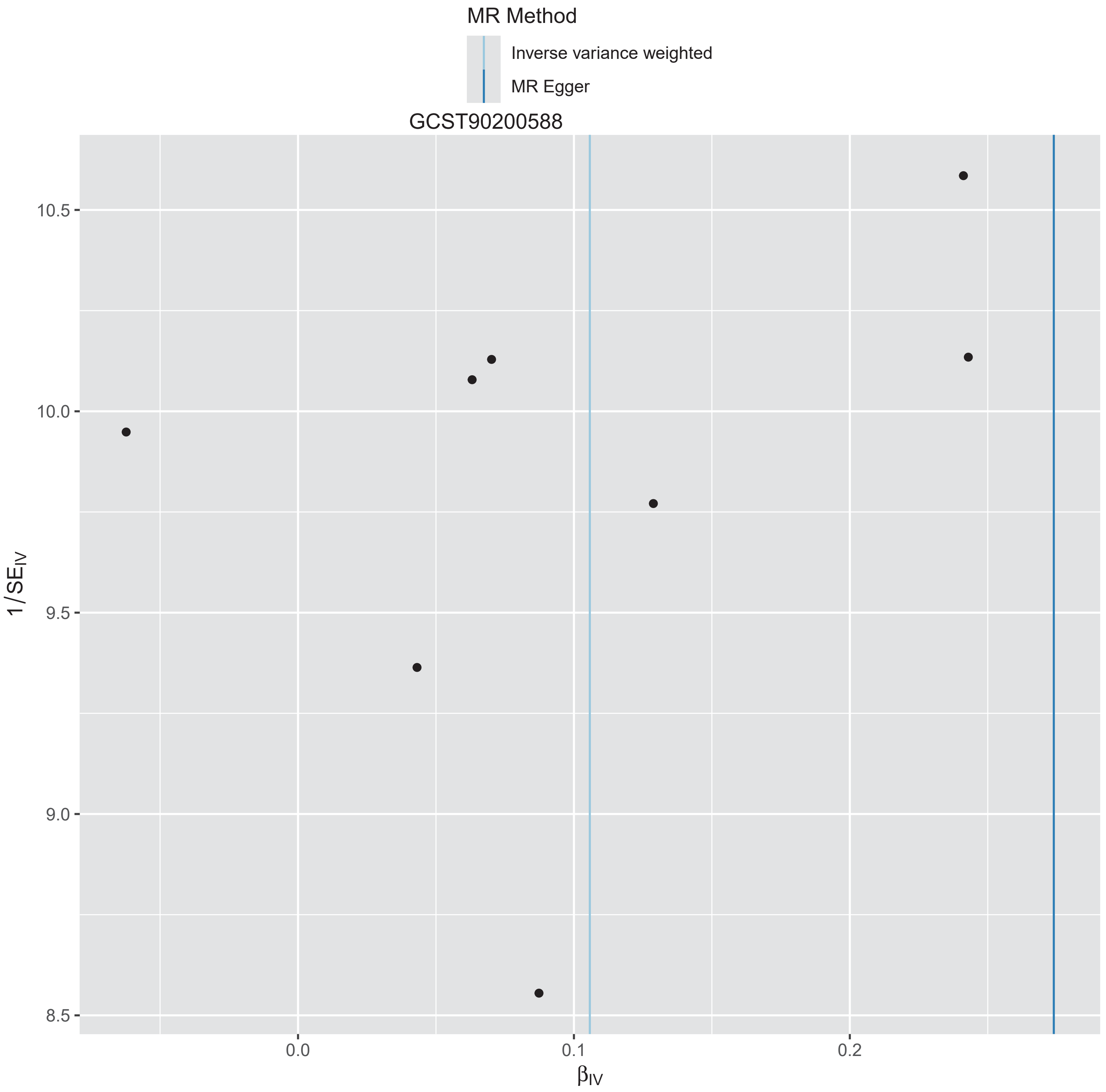
MR Method
Inverse variance weighted
MR Egger
GCST90200588
10.5
10.0
9.5
9.0
8.5
1/SE_IV
0.0
0.1
0.2
β_IV

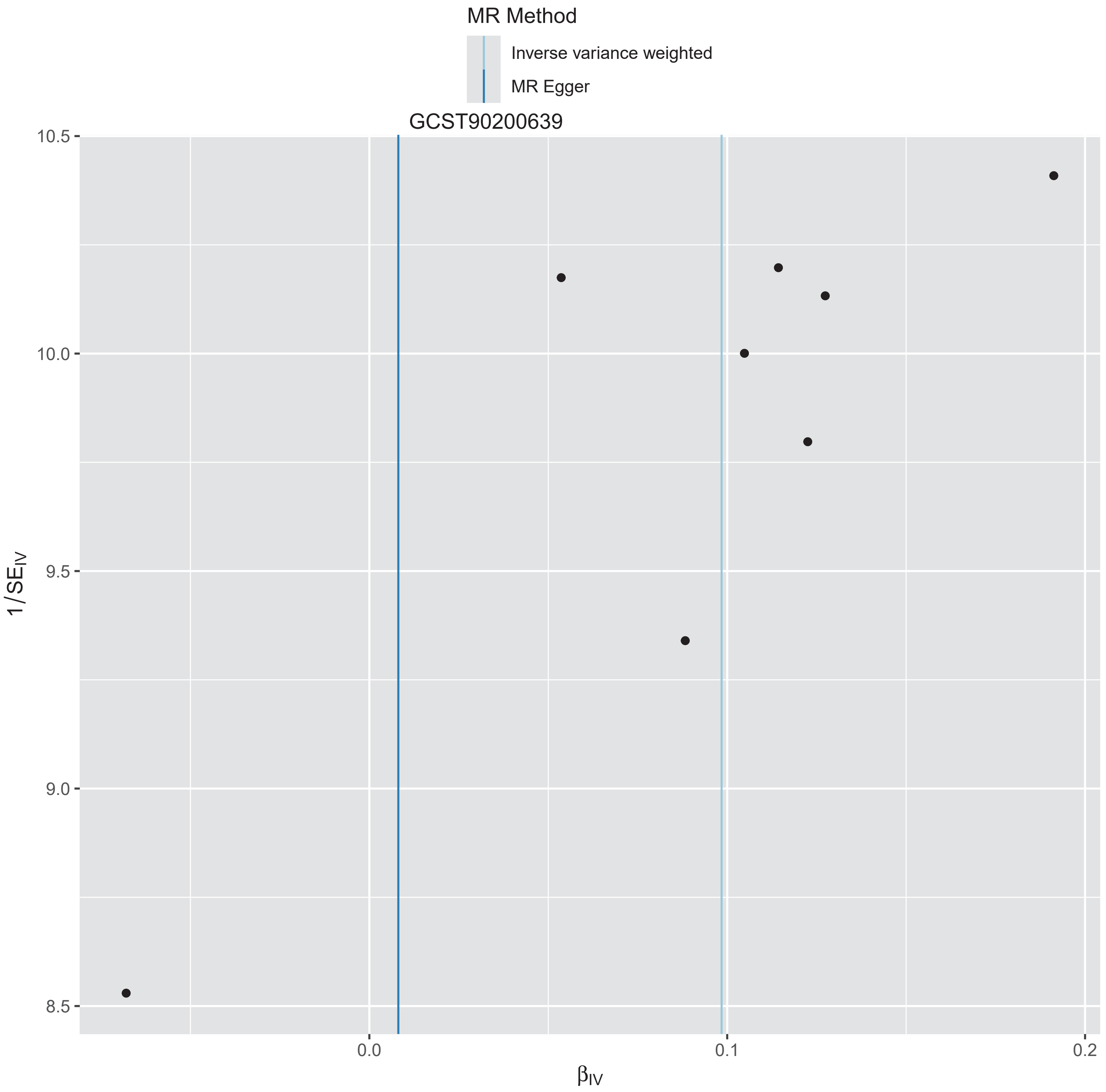
MR Method
Inverse variance weighted
MR Egger
GCST90200639
10.5
10.0
9.5
9.0
8.5
1/SE_IV
0.0
0.1
0.2
β_IV

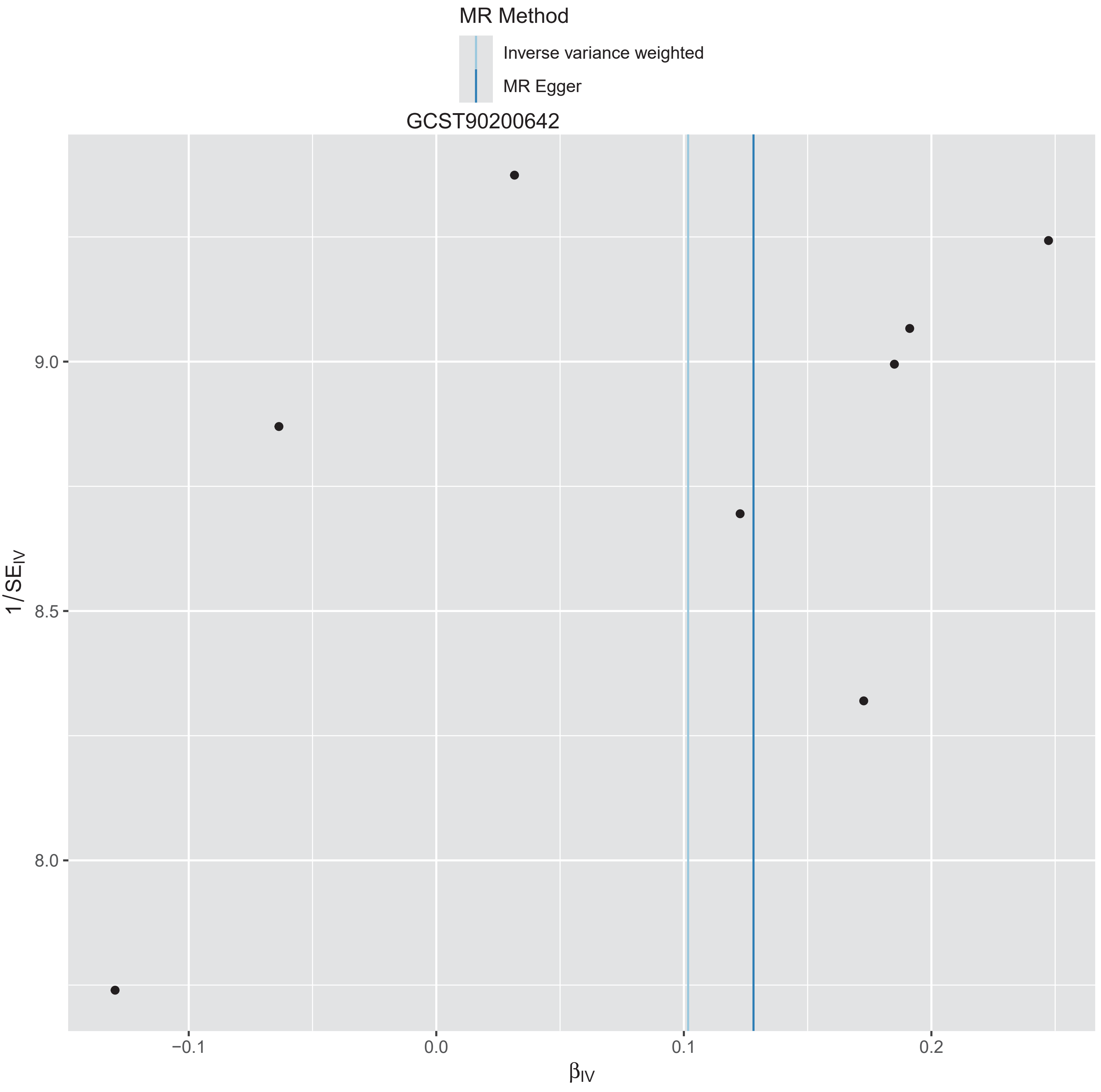

MR Method
Inverse variance weighted
MR Egger
GCST90200642
9.0
8.5
8.0
1/SE_IV
−0.1
0.0
0.1
0.2
β_IV

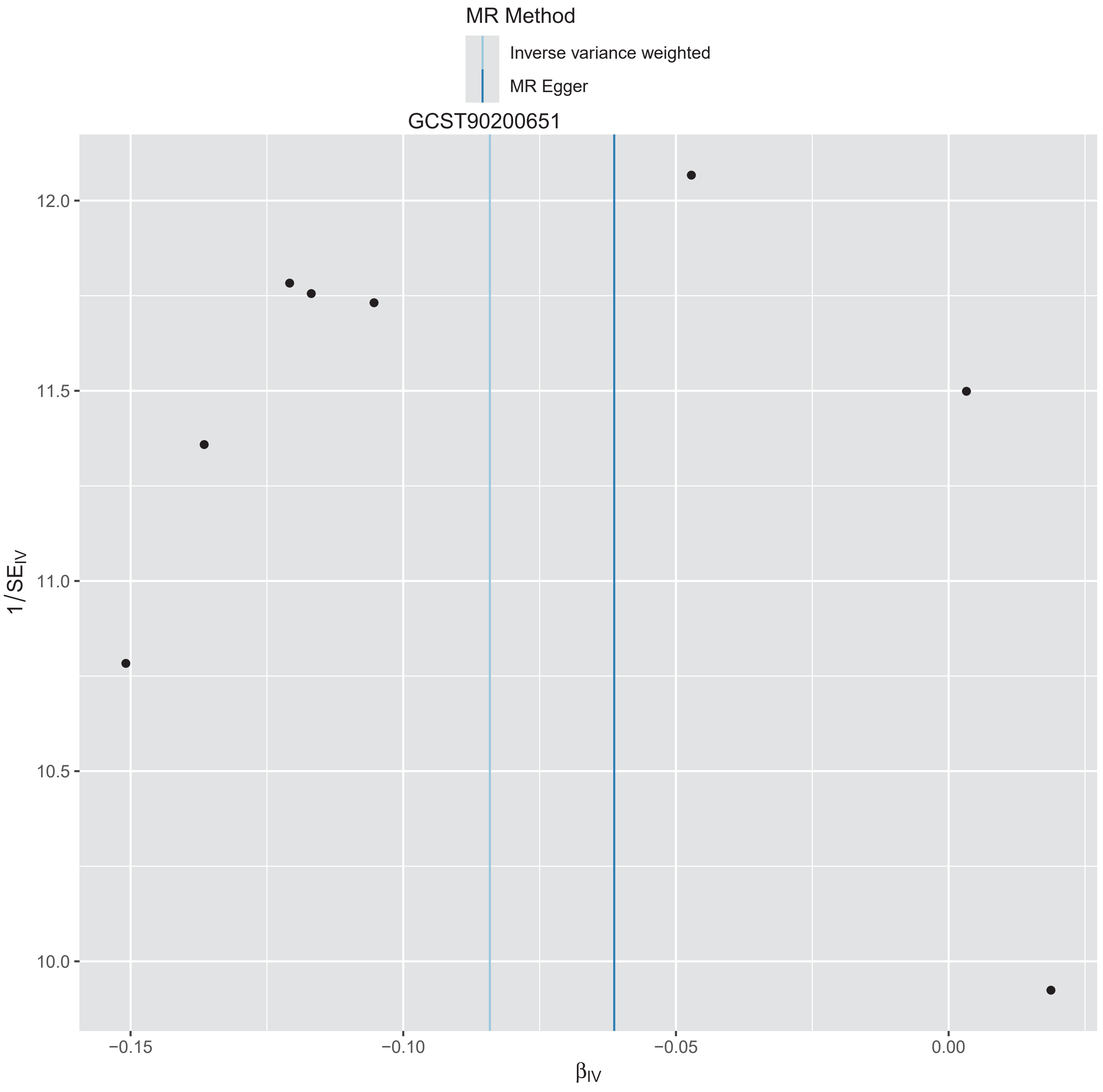
MR Method
Inverse variance weighted
MR Egger
GCST90200651
12.0
11.5
11.0
10.5
10.0
1/SE_IV
−0.15
−0.10
−0.05
0.00
β_IV

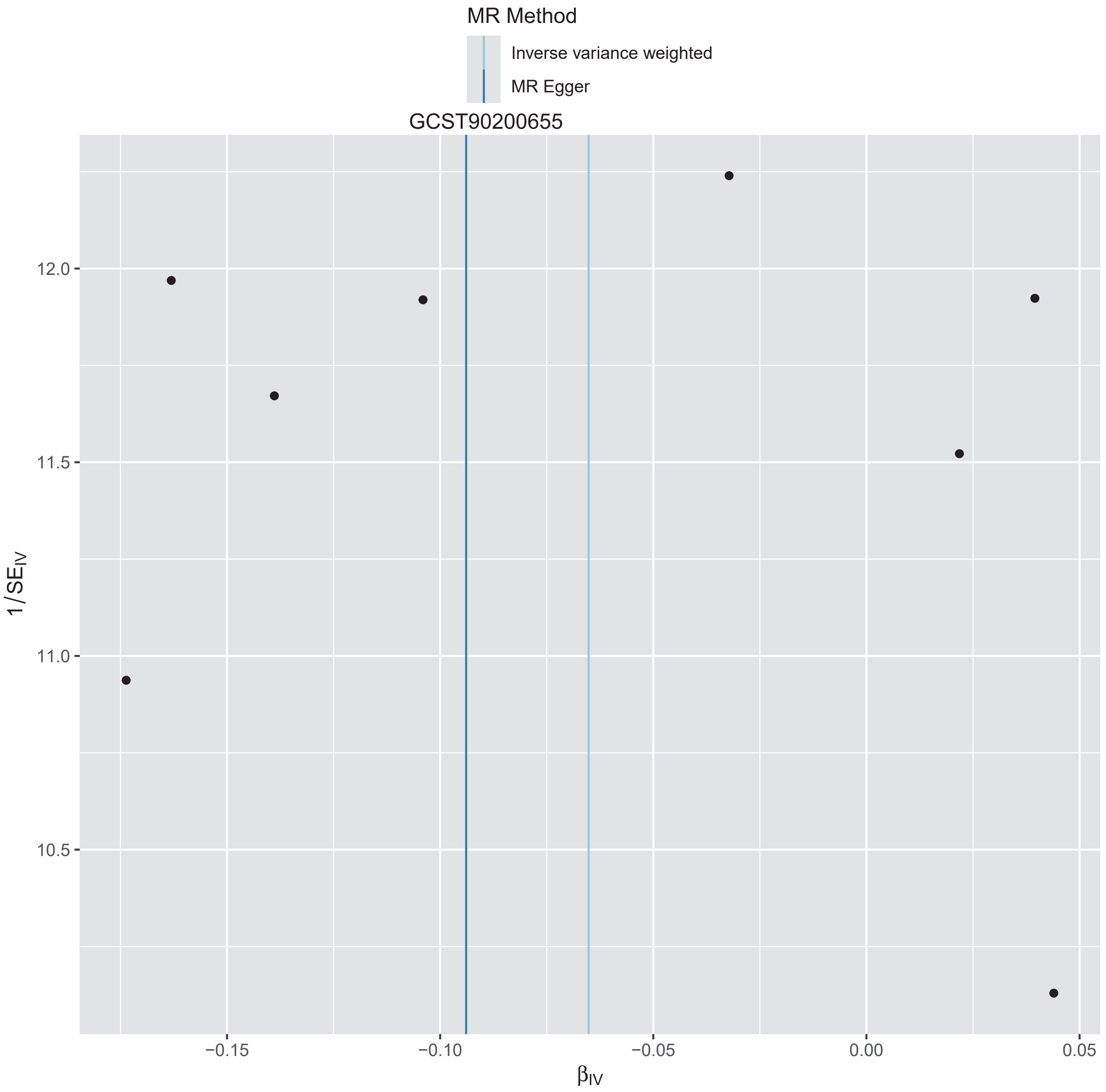
MR Method
Inverse variance weighted
MR Egger
GCST90200655
12.0
11.5
11.0
10.5
1/SE_IV
−0.15
−0.10
−0.05
0.00
0.05
β_IV

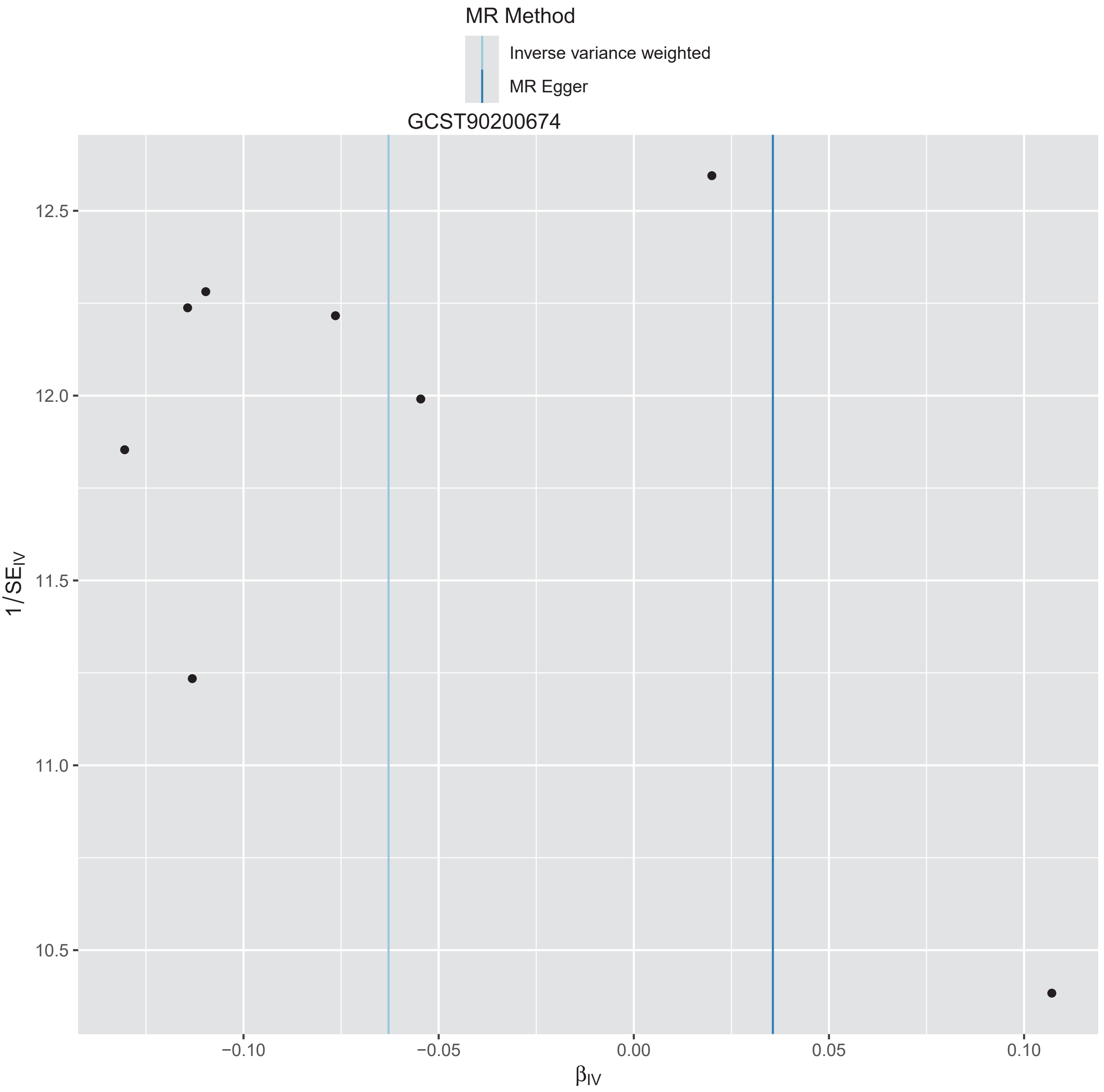

MR Method
Inverse variance weighted
MR Egger
GCST90200674
12.5
12.0
11.5
11.0
10.5
1/SE_IV
−0.10
−0.05
0.00
0.05
0.10
β_IV
