# Supplementary material for: Dissecting Causal Relationships Between Gut Microbiota, Plasma Metabolites and Bladder Cancer: A Two‐Step Mendelian Randomization Study
Source: Health Sci Rep. 2025 Sep 9;8(9):e71206. doi: 10.1002/hsr2.71206 (PMC12420358; doi:10.1002/hsr2.71206)
Supplement: Supplementary file 8 — Figure S8: Leave‐one‐out analysis for MR causal effects of plasma metabolites on s_Bacteroides_salyersiae. [file HSR2-8-e71206-s001.pdf]

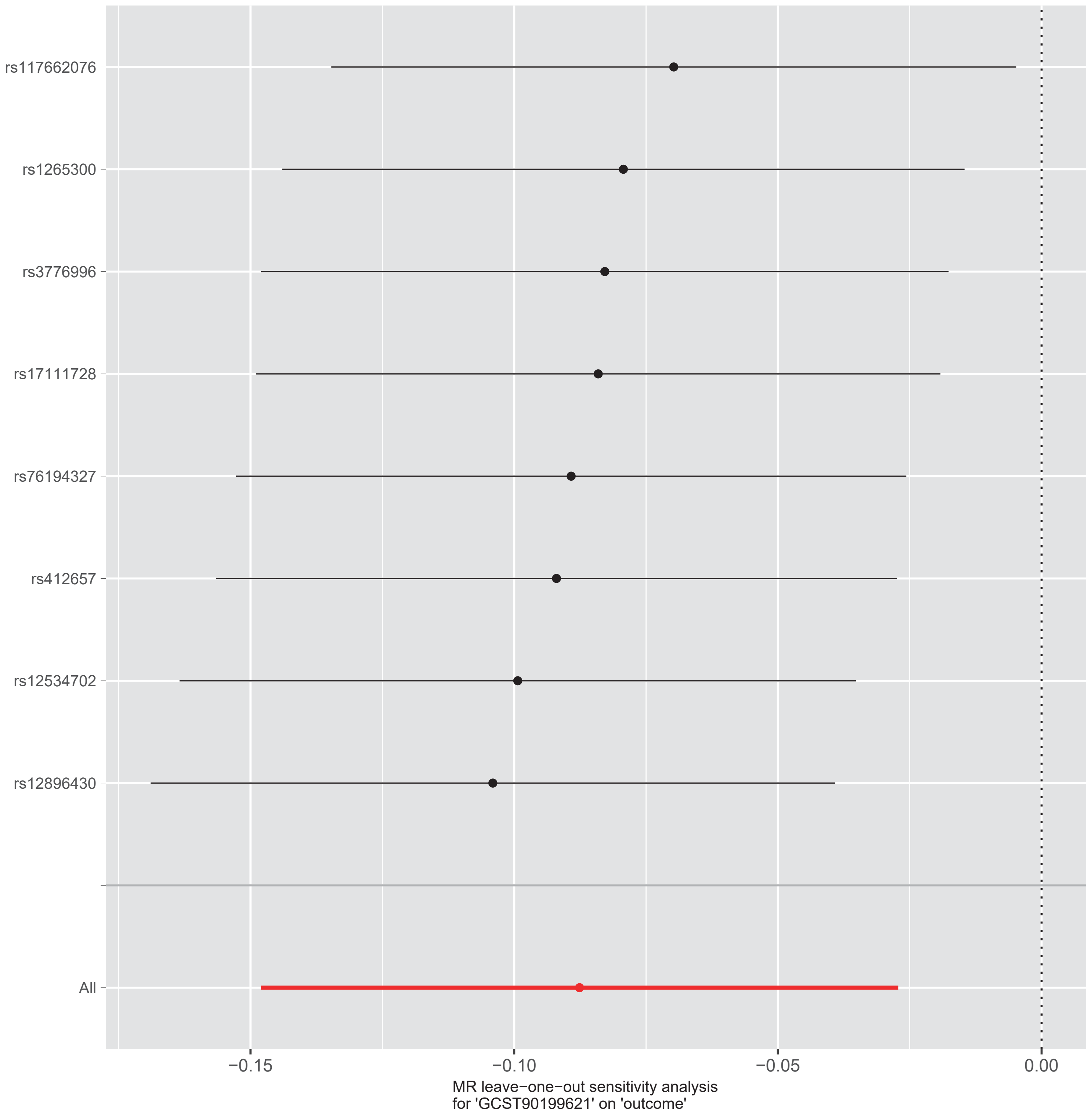
rs117662076
rs1265300
rs3776996
rs17111728
rs76194327
rs412657
rs12534702
rs12896430
All
−0.15
−0.10
−0.05
0.00
MR leave−one−out sensitivity analysis
for 'GCST90199621' on 'outcome'

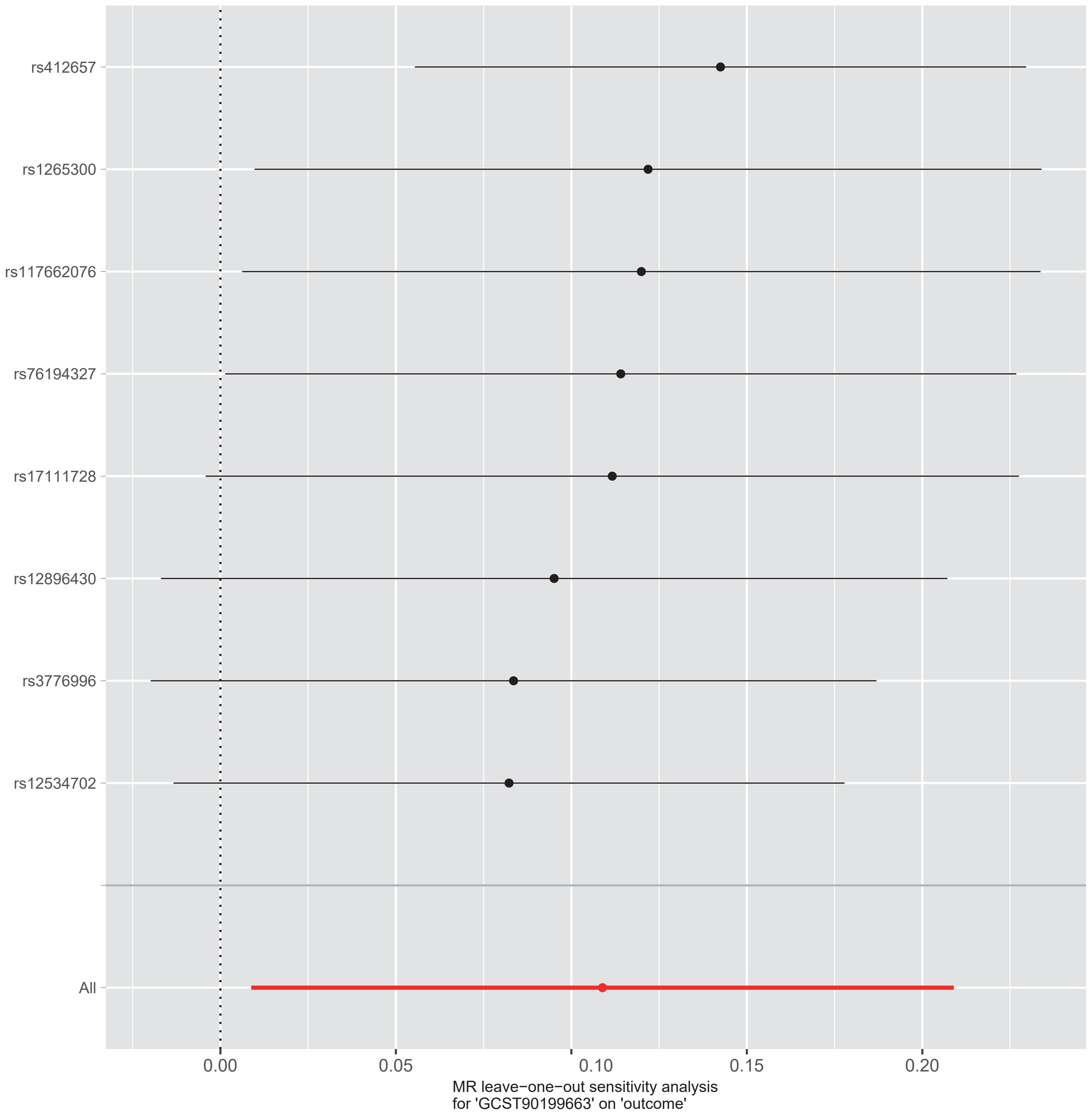

rs412657
rs1265300
rs117662076
rs76194327
rs17111728
rs12896430
rs3776996
rs12534702
All
0.00
0.05
0.10
0.15
0.20
MR leave−one−out sensitivity analysis
for 'GCST90199663' on 'outcome'

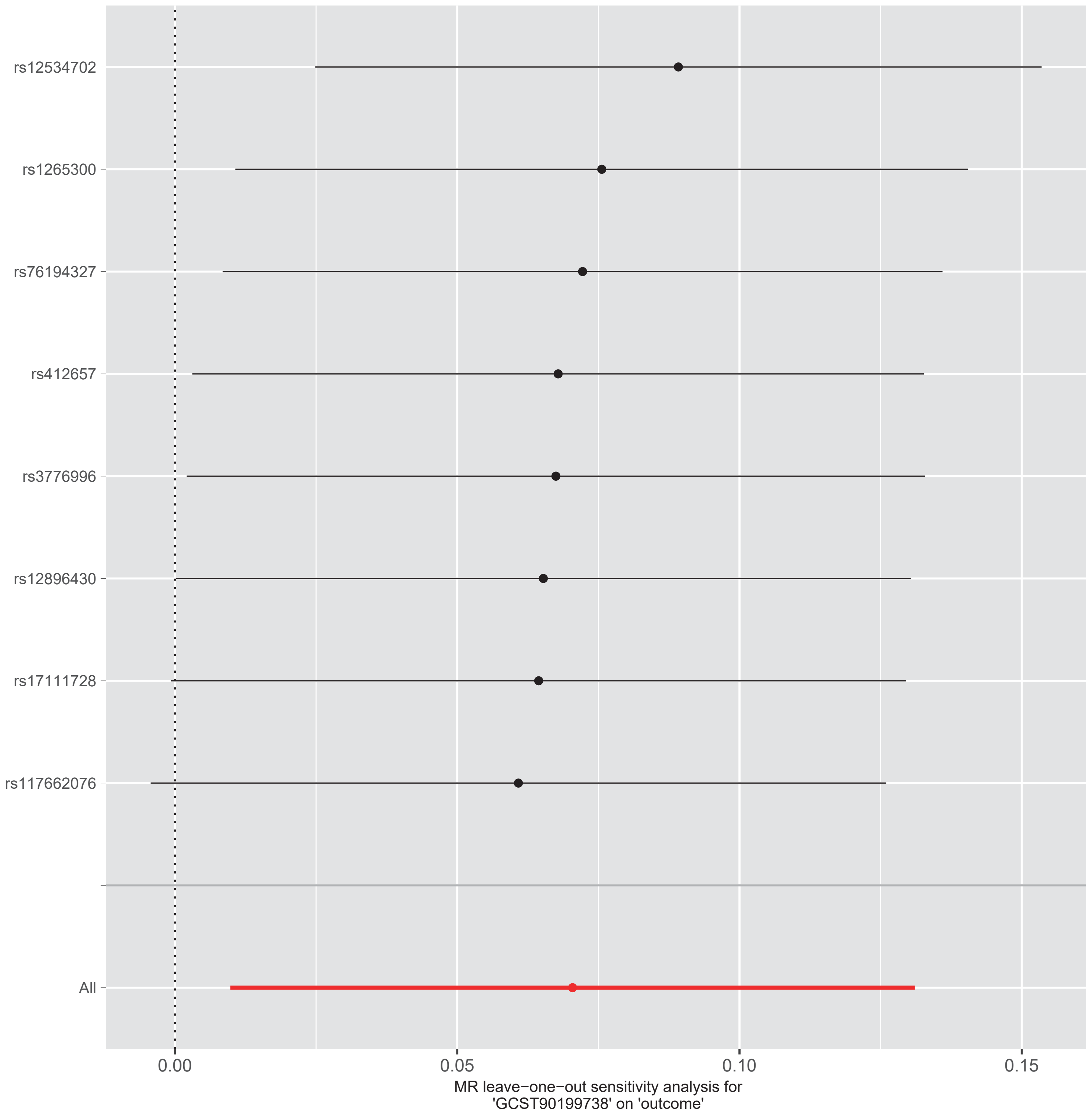

rs12534702
rs1265300
rs76194327
rs412657
rs3776996
rs12896430
rs17111728
rs117662076
All
0.00
0.05
0.10
0.15
MR leave−one−out sensitivity analysis for
'GCST90199738' on 'outcome'

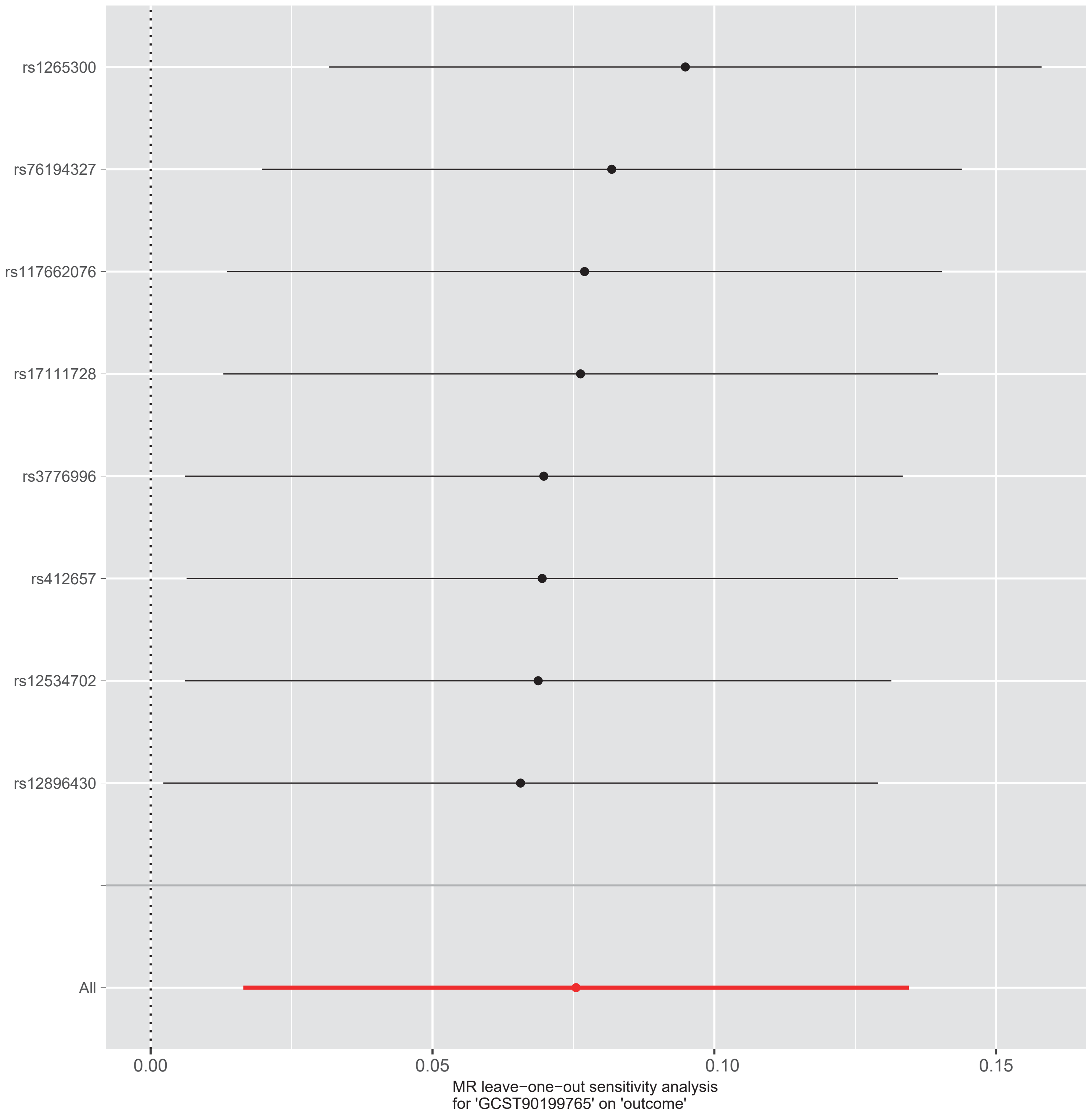
rs1265300
rs76194327
rs117662076
rs17111728
rs3776996
rs412657
rs12534702
rs12896430
All
0.00
0.05
0.10
0.15
MR leave−one−out sensitivity analysis
for 'GCST90199765' on 'outcome'

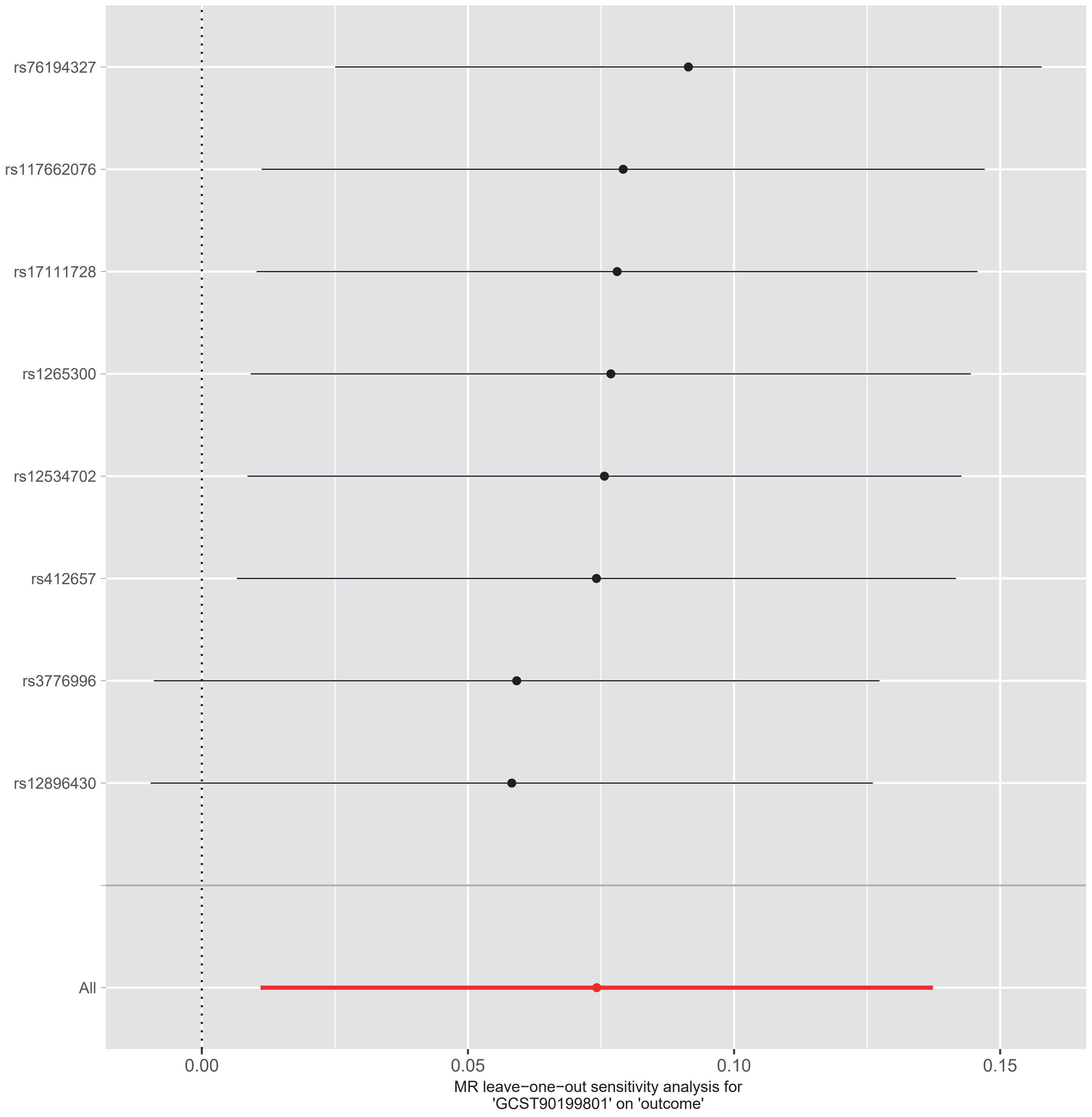

rs76194327
rs117662076
rs17111728
rs1265300
rs12534702
rs412657
rs3776996
rs12896430
All
0.00
0.05
0.10
0.15
MR leave−one−out sensitivity analysis for
'GCST90199801' on 'outcome'

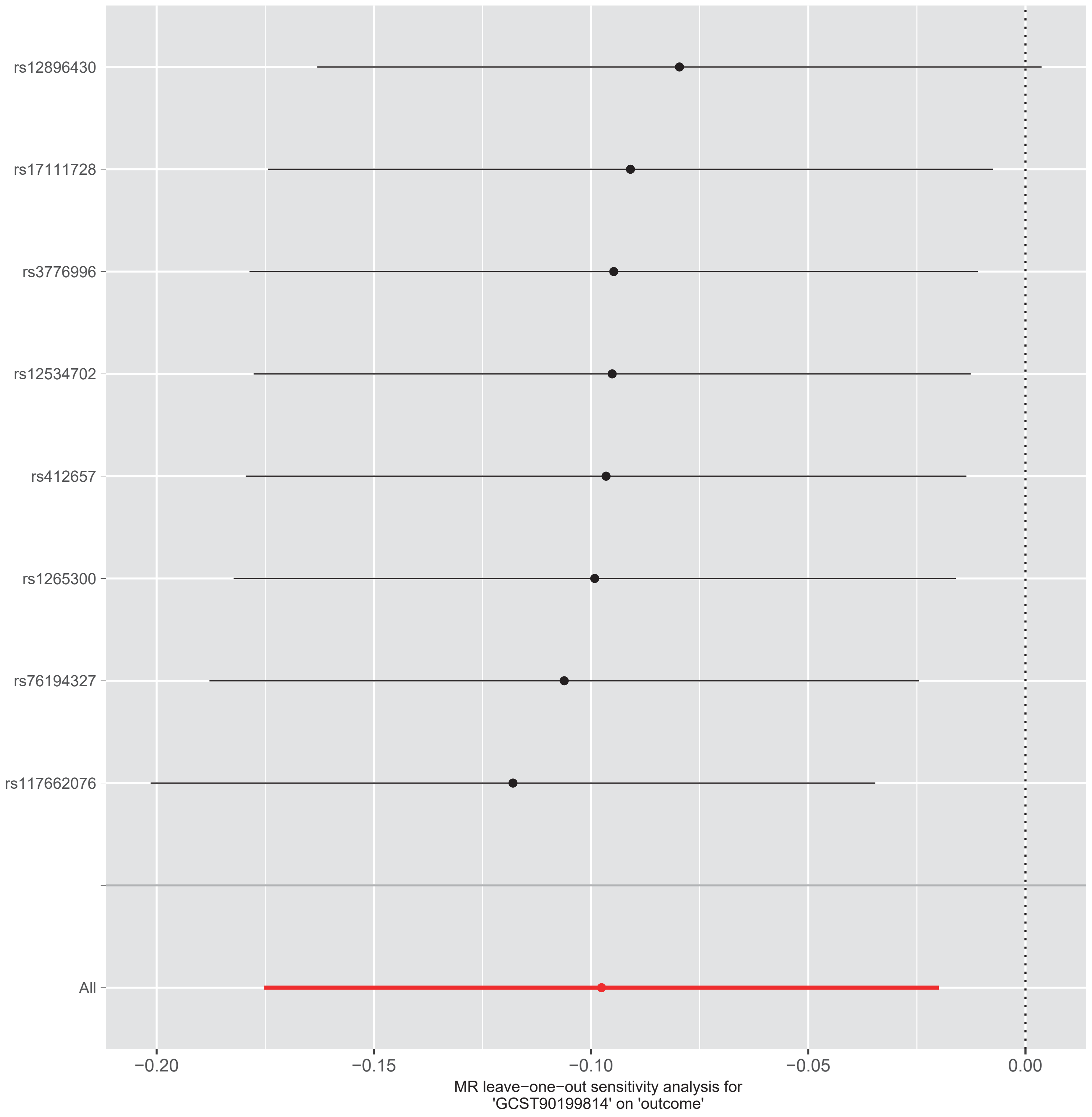
rs12896430
rs17111728
rs3776996
rs12534702
rs412657
rs1265300
rs76194327
rs117662076
All
−0.20
−0.15
−0.10
−0.05
0.00
MR leave−one−out sensitivity analysis for
'GCST90199814' on 'outcome'

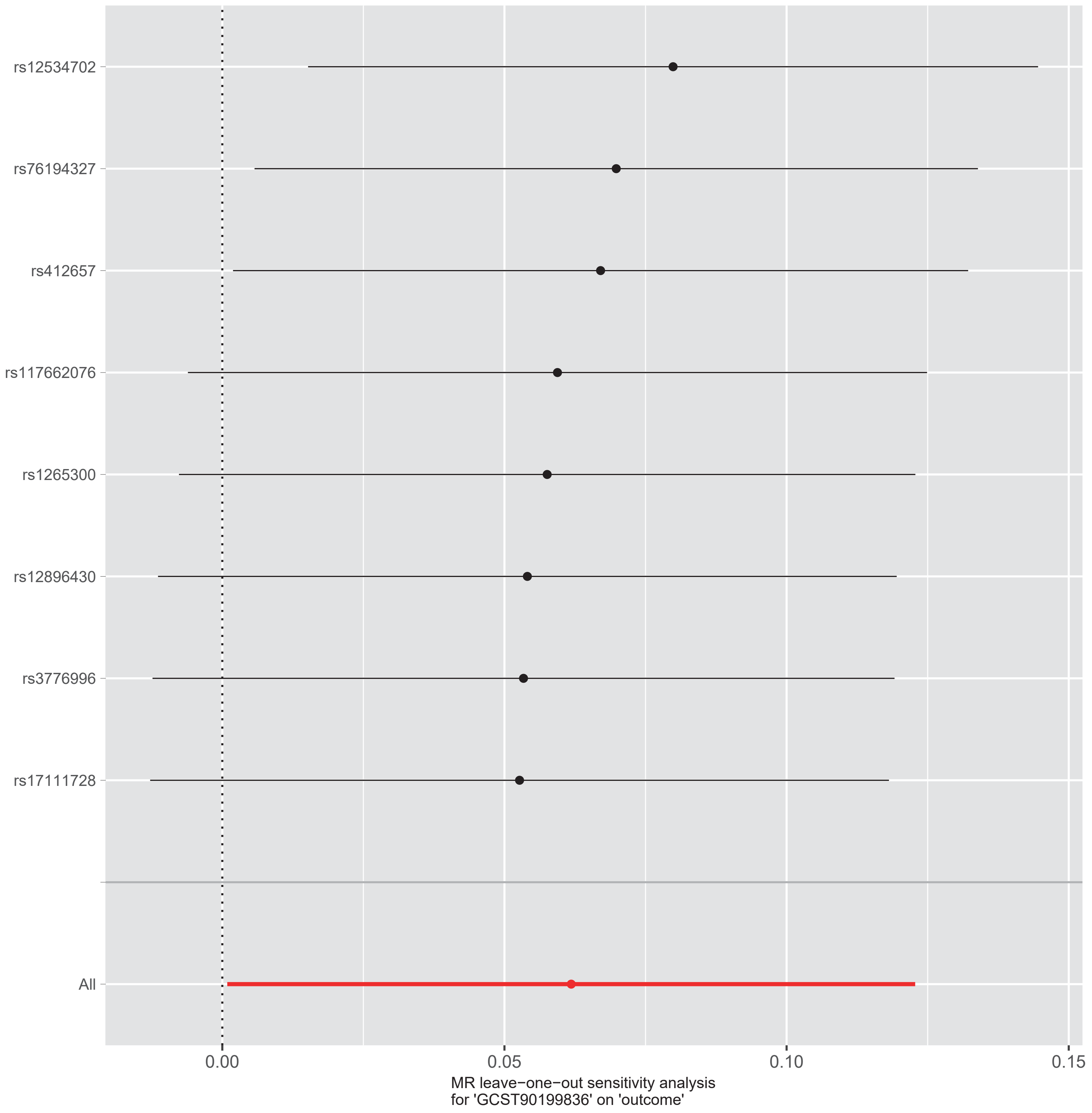

rs12534702
rs76194327
rs412657
rs117662076
rs1265300
rs12896430
rs3776996
rs17111728
All
0.00
0.05
0.10
0.15
MR leave−one−out sensitivity analysis
for 'GCST90199836' on 'outcome'

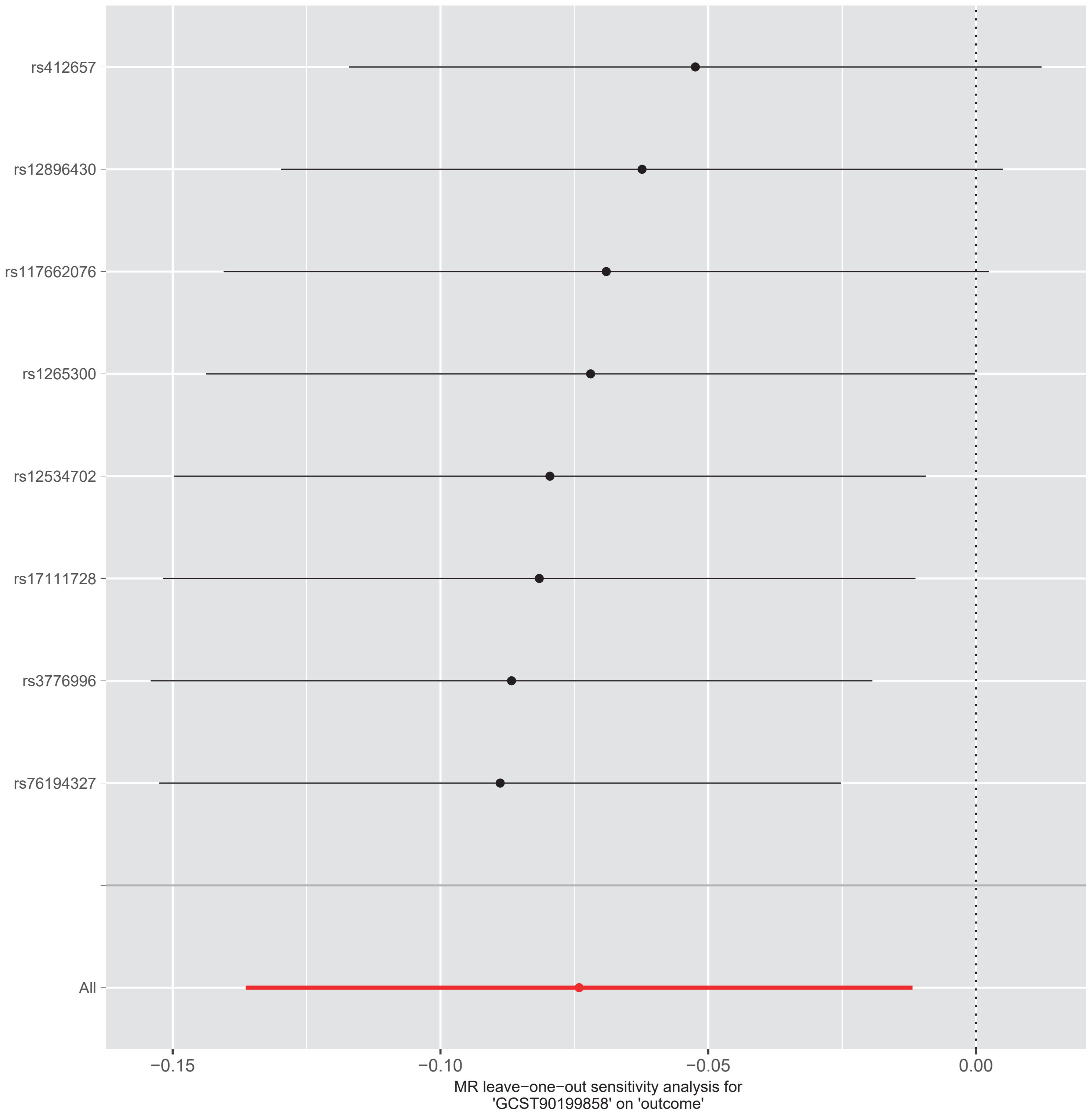

rs412657
rs12896430
rs117662076
rs1265300
rs12534702
rs17111728
rs3776996
rs76194327
All
−0.15
−0.10
−0.05
0.00
MR leave−one−out sensitivity analysis for
'GCST90199858' on 'outcome'

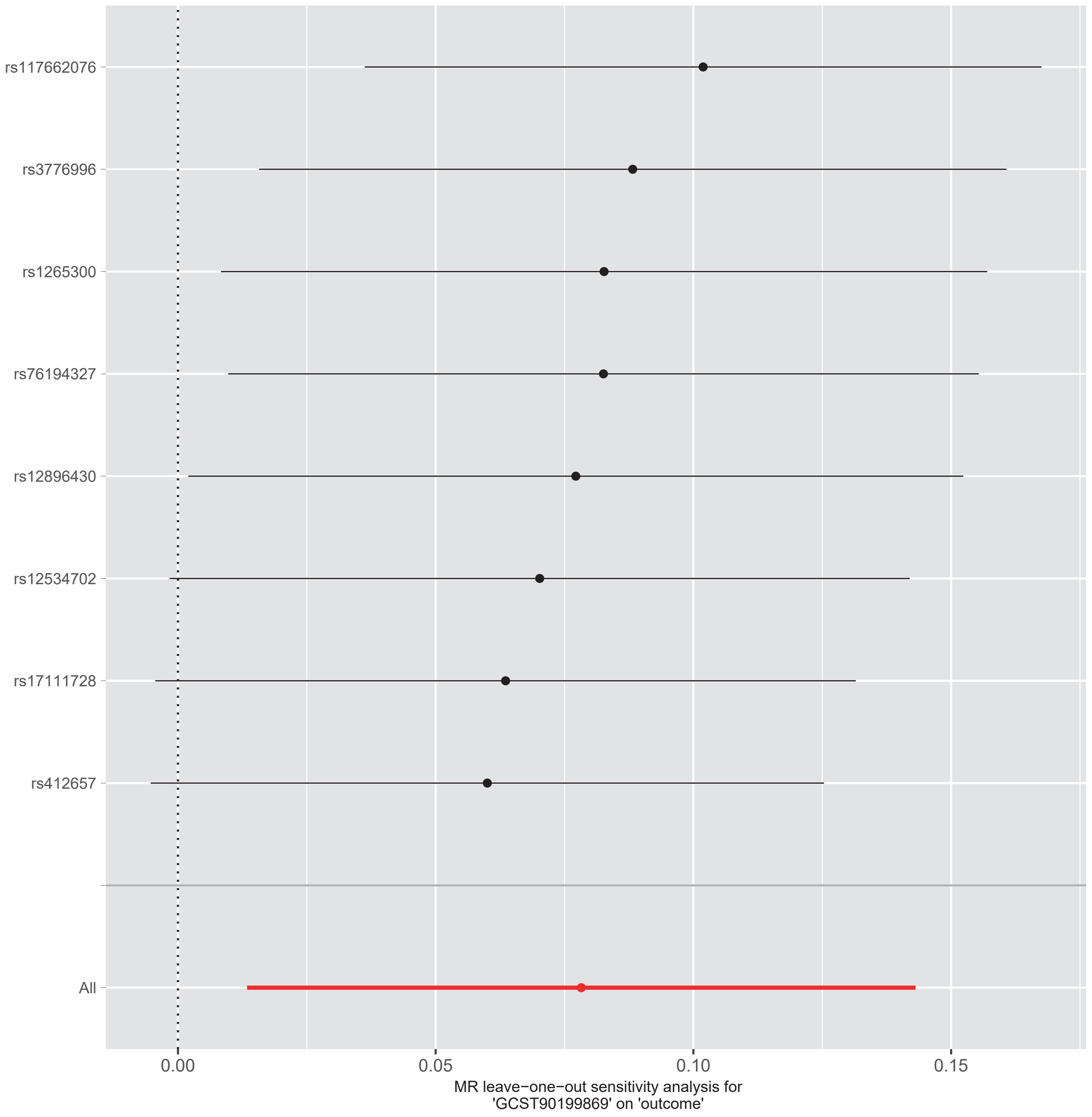
rs117662076
rs3776996
rs1265300
rs76194327
rs12896430
rs12534702
rs17111728
rs412657
All
0.00
0.05
0.10
0.15
MR leave−one−out sensitivity analysis for
'GCST90199869' on 'outcome'

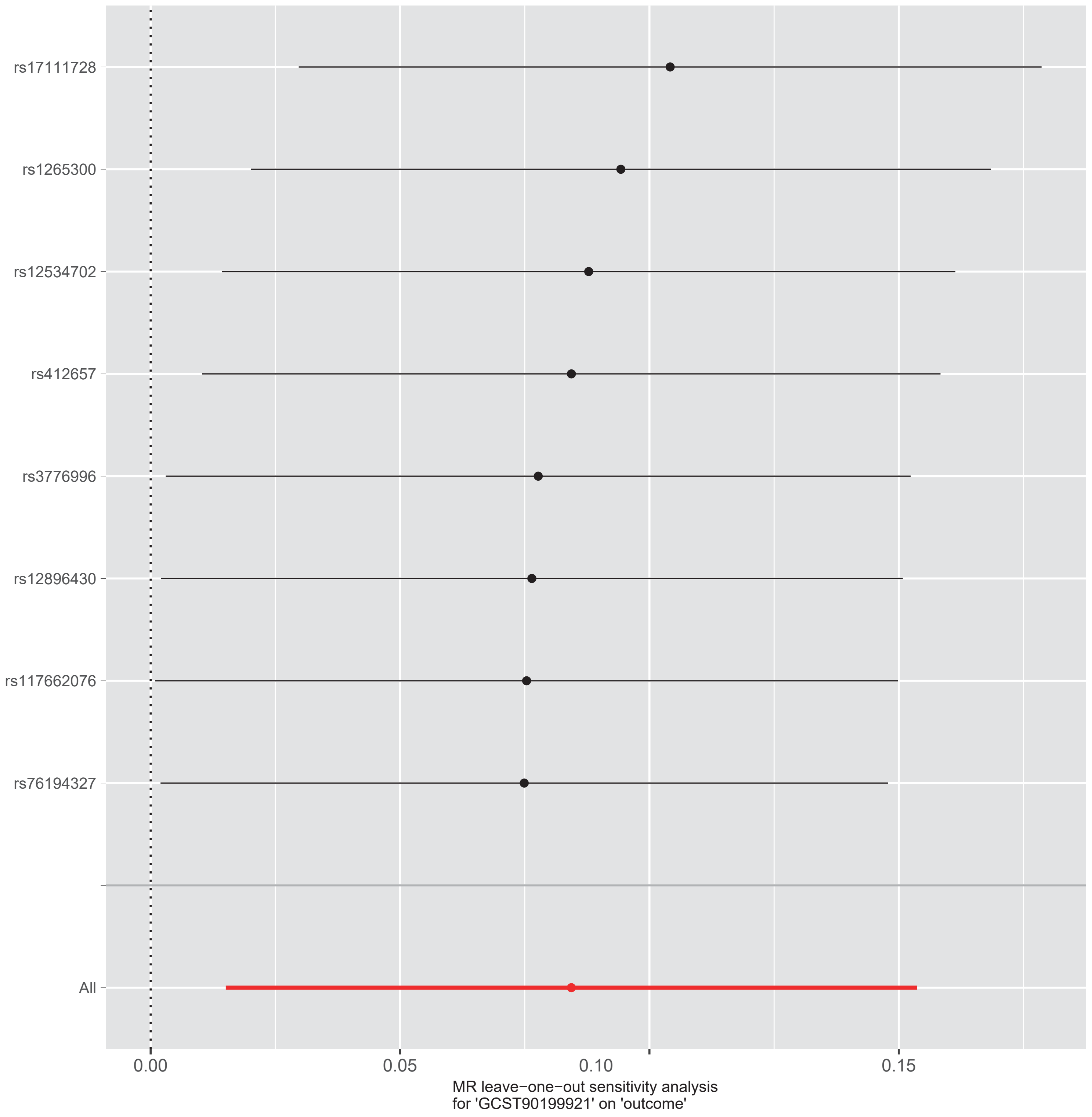
rs17111728
rs1265300
rs12534702
rs412657
rs3776996
rs12896430
rs117662076
rs76194327
All
0.00
0.05
0.10
0.15
MR leave−one−out sensitivity analysis
for 'GCST90199921' on 'outcome'

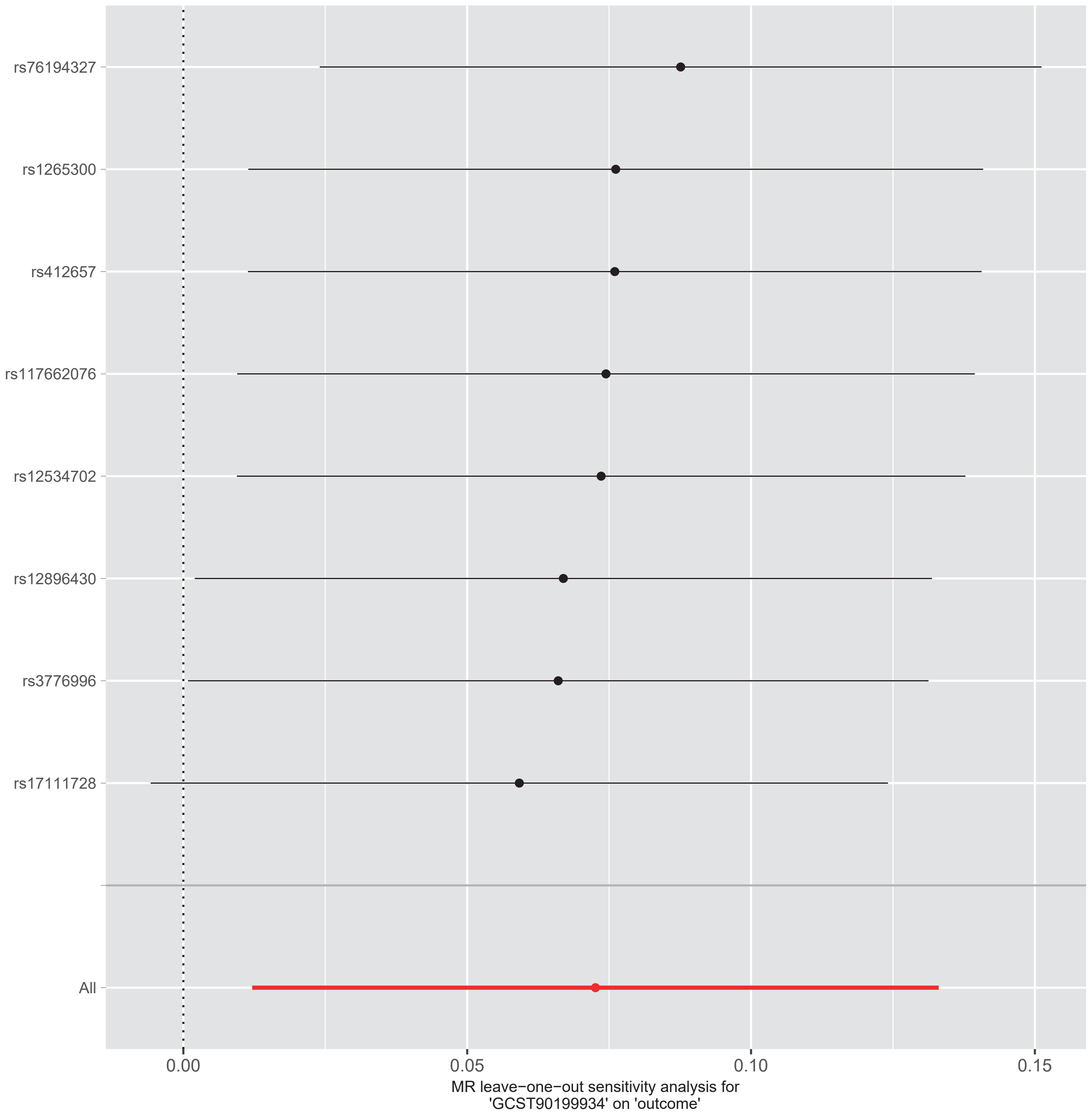
rs76194327
rs1265300
rs412657
rs117662076
rs12534702
rs12896430
rs3776996
rs17111728
All
0.00
0.05
0.10
0.15
MR leave−one−out sensitivity analysis for
'GCST90199934' on 'outcome'

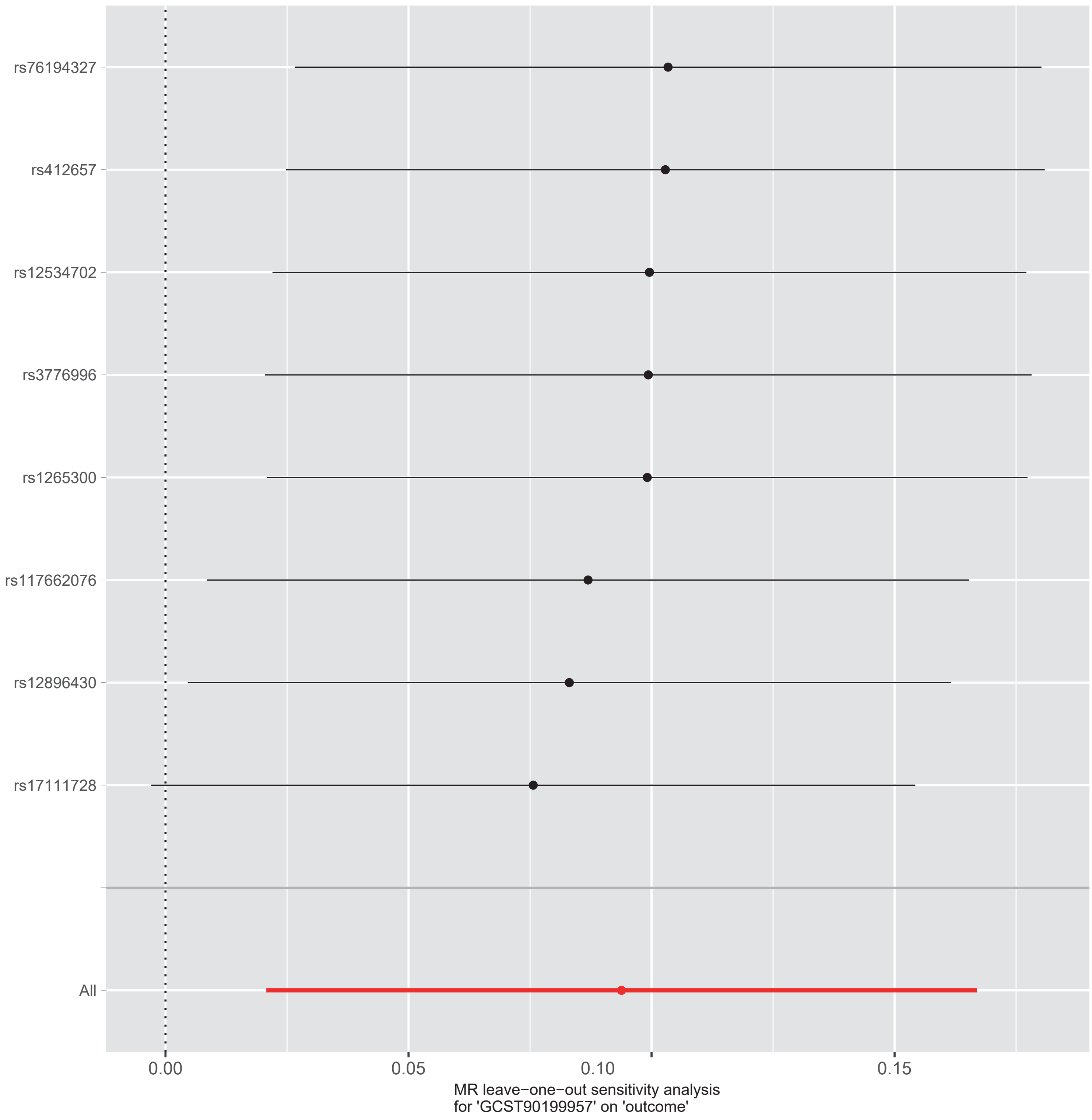
rs76194327
rs412657
rs12534702
rs3776996
rs1265300
rs117662076
rs12896430
rs17111728
All
0.00
0.05
0.10
0.15
MR leave−one−out sensitivity analysis
for 'GCST90199957' on 'outcome'

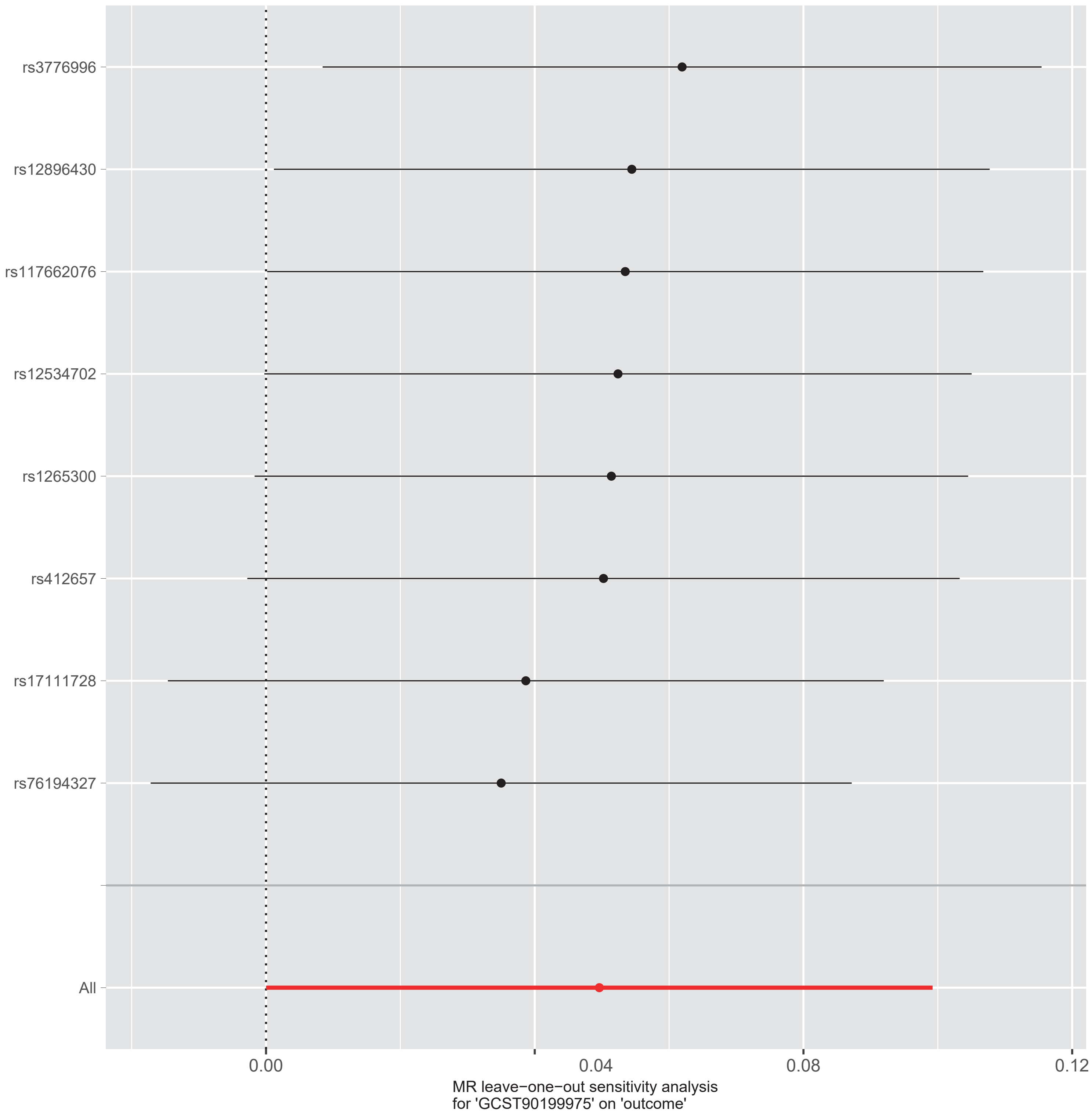

rs3776996
rs12896430
rs117662076
rs12534702
rs1265300
rs412657
rs17111728
rs76194327
All
0.00
0.04
0.08
0.12
MR leave−one−out sensitivity analysis
for 'GCST90199975' on 'outcome'

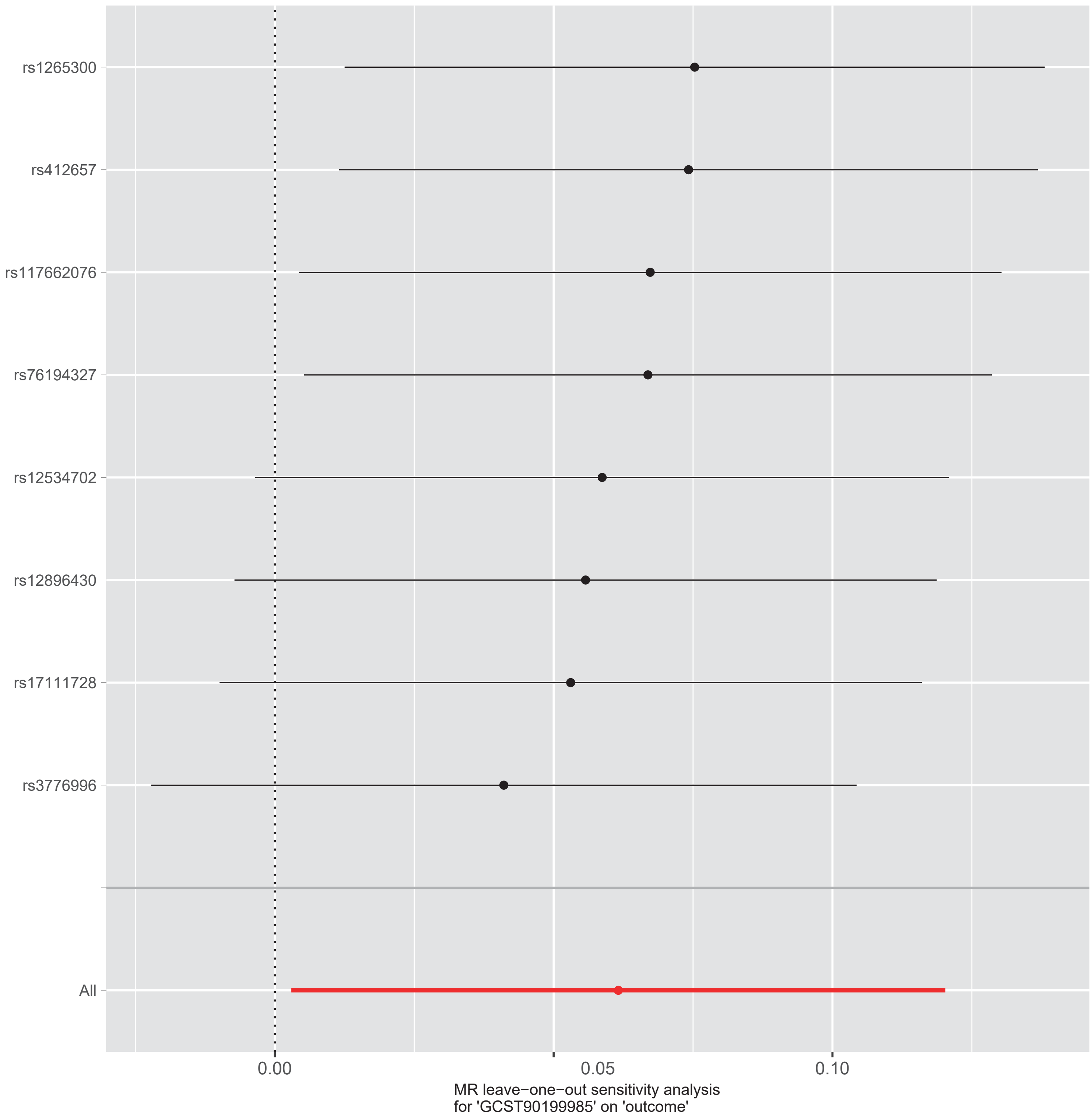
rs1265300
rs412657
rs117662076
rs76194327
rs12534702
rs12896430
rs17111728
rs3776996
All
0.00
0.05
0.10
MR leave−one−out sensitivity analysis
for 'GCST90199985' on 'outcome'

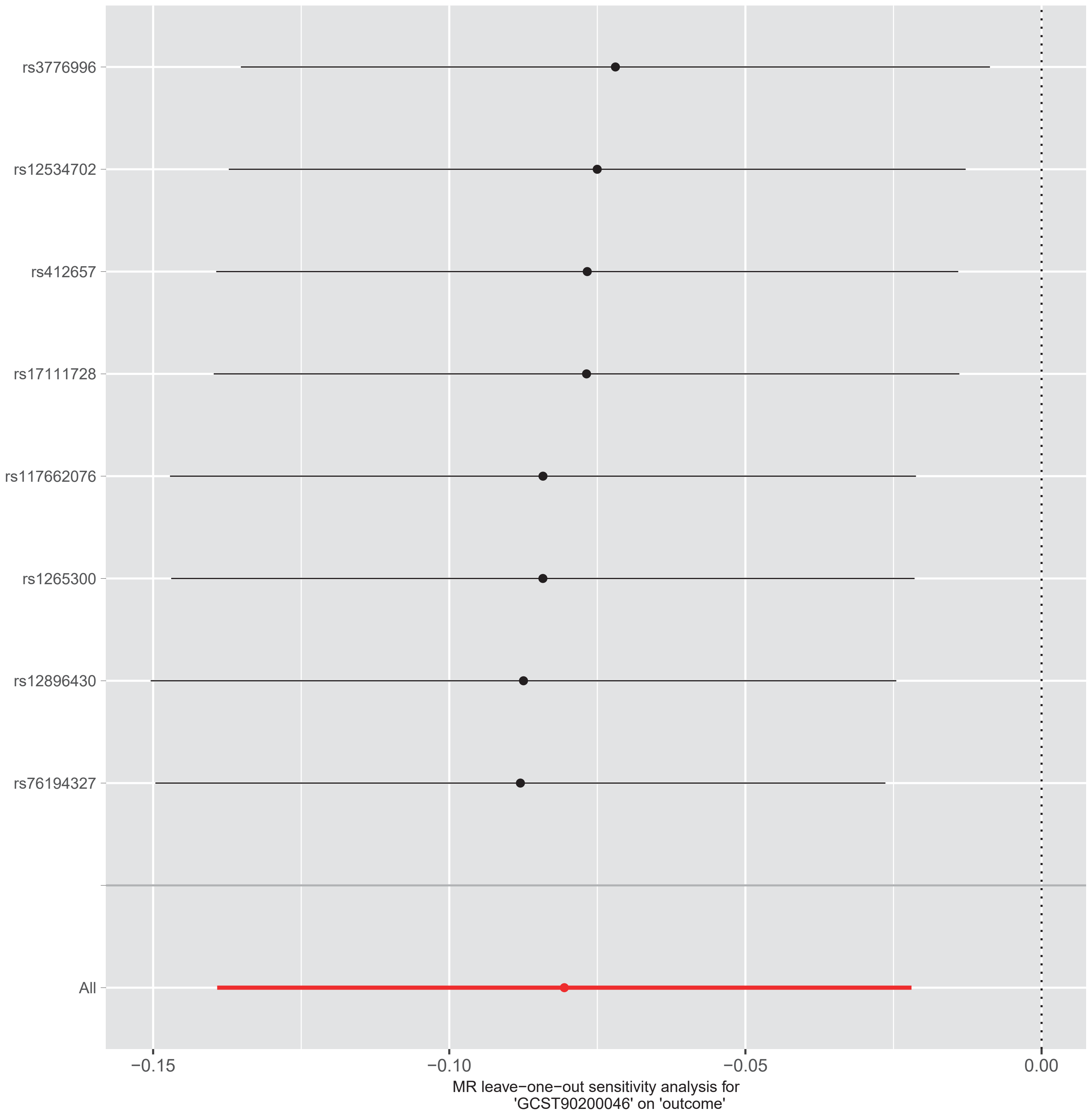

rs3776996
rs12534702
rs412657
rs17111728
rs117662076
rs1265300
rs12896430
rs76194327
All
−0.15
−0.10
−0.05
0.00
MR leave−one−out sensitivity analysis for
'GCST90200046' on 'outcome'

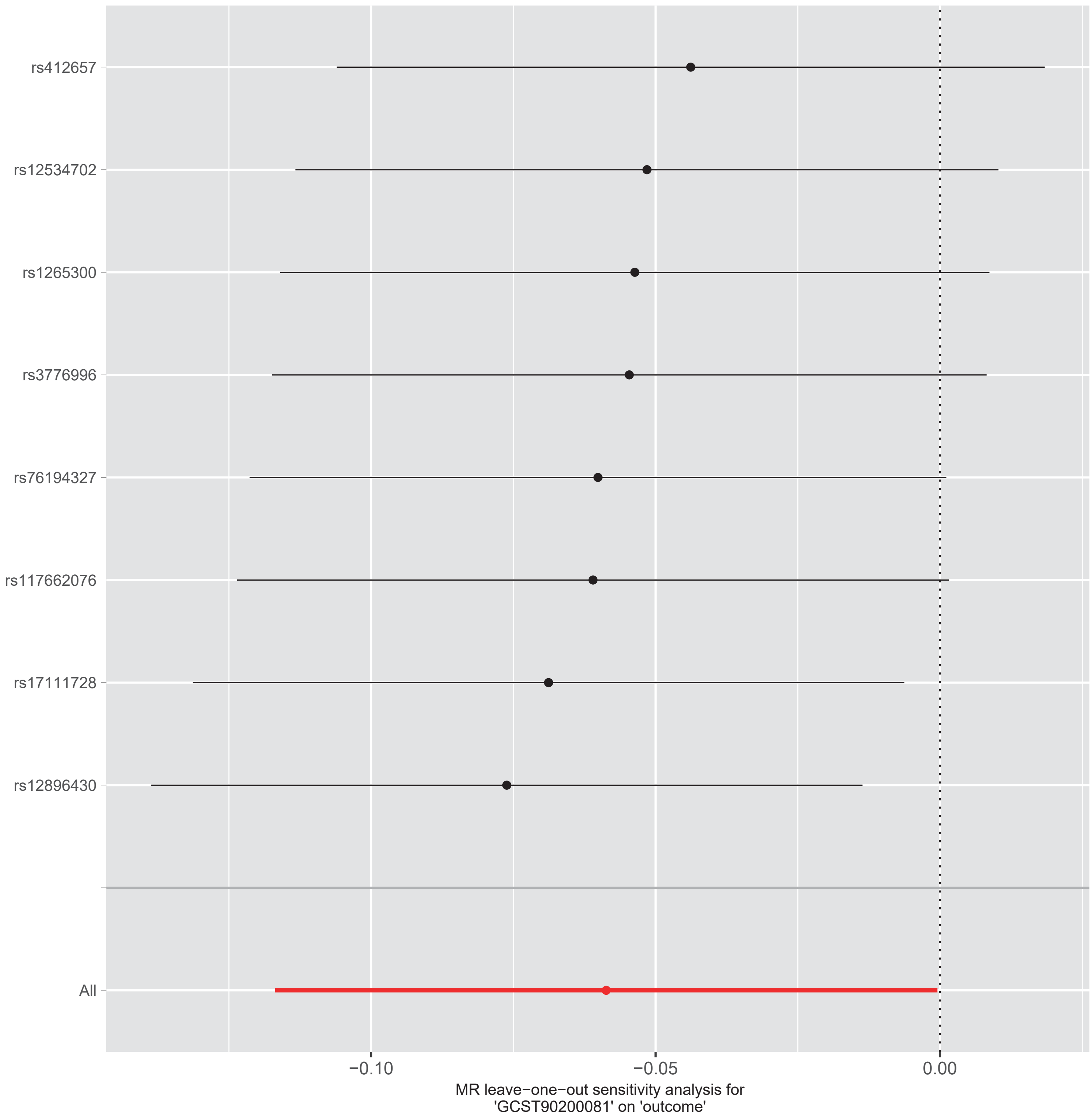
rs412657
rs12534702
rs1265300
rs3776996
rs76194327
rs117662076
rs17111728
rs12896430
All
−0.10
−0.05
0.00
MR leave−one−out sensitivity analysis for
'GCST90200081' on 'outcome'

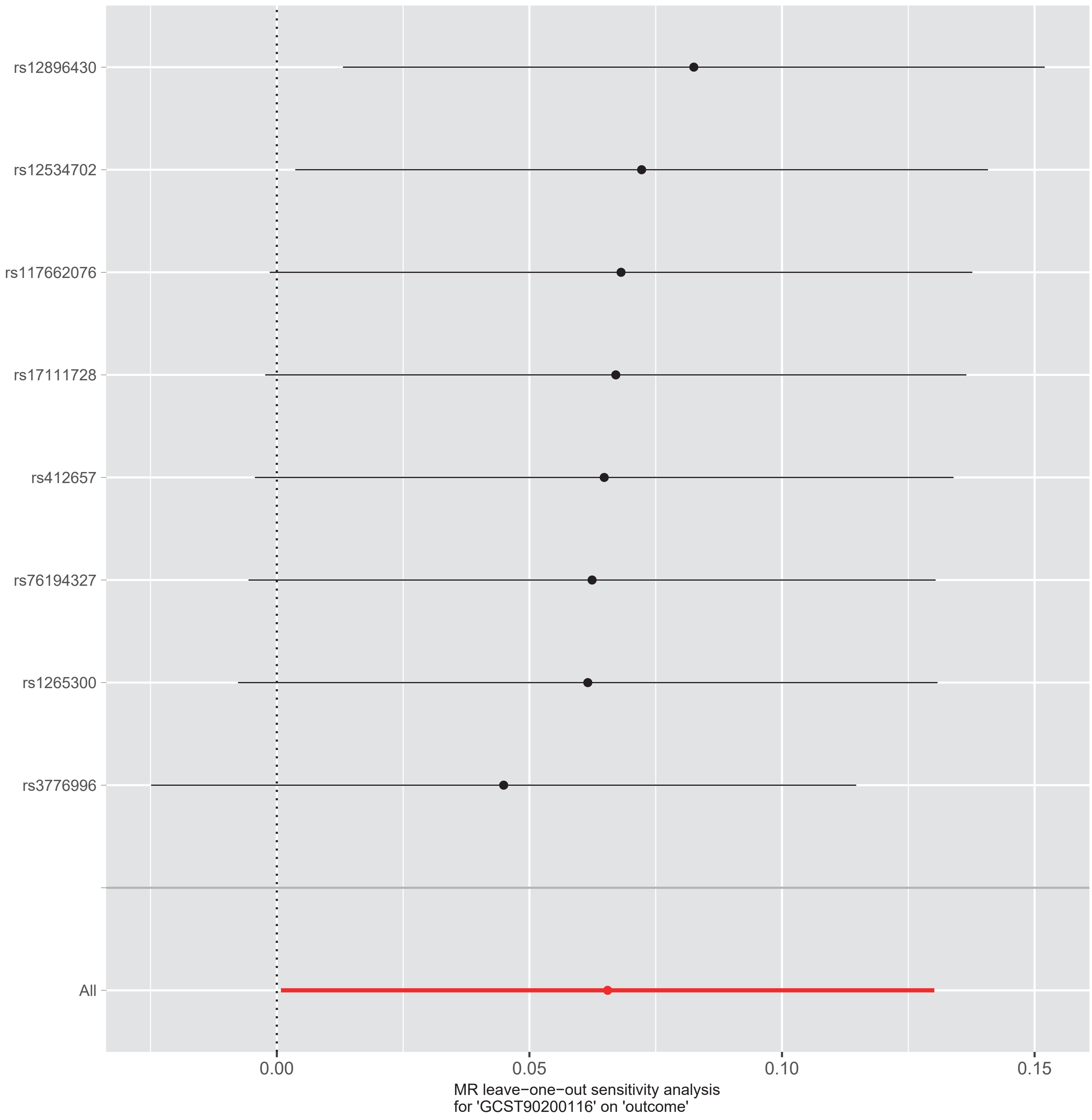

rs12896430
rs12534702
rs117662076
rs17111728
rs412657
rs76194327
rs1265300
rs3776996
All
0.00
0.05
0.10
0.15
MR leave−one−out sensitivity analysis
for 'GCST90200116' on 'outcome'

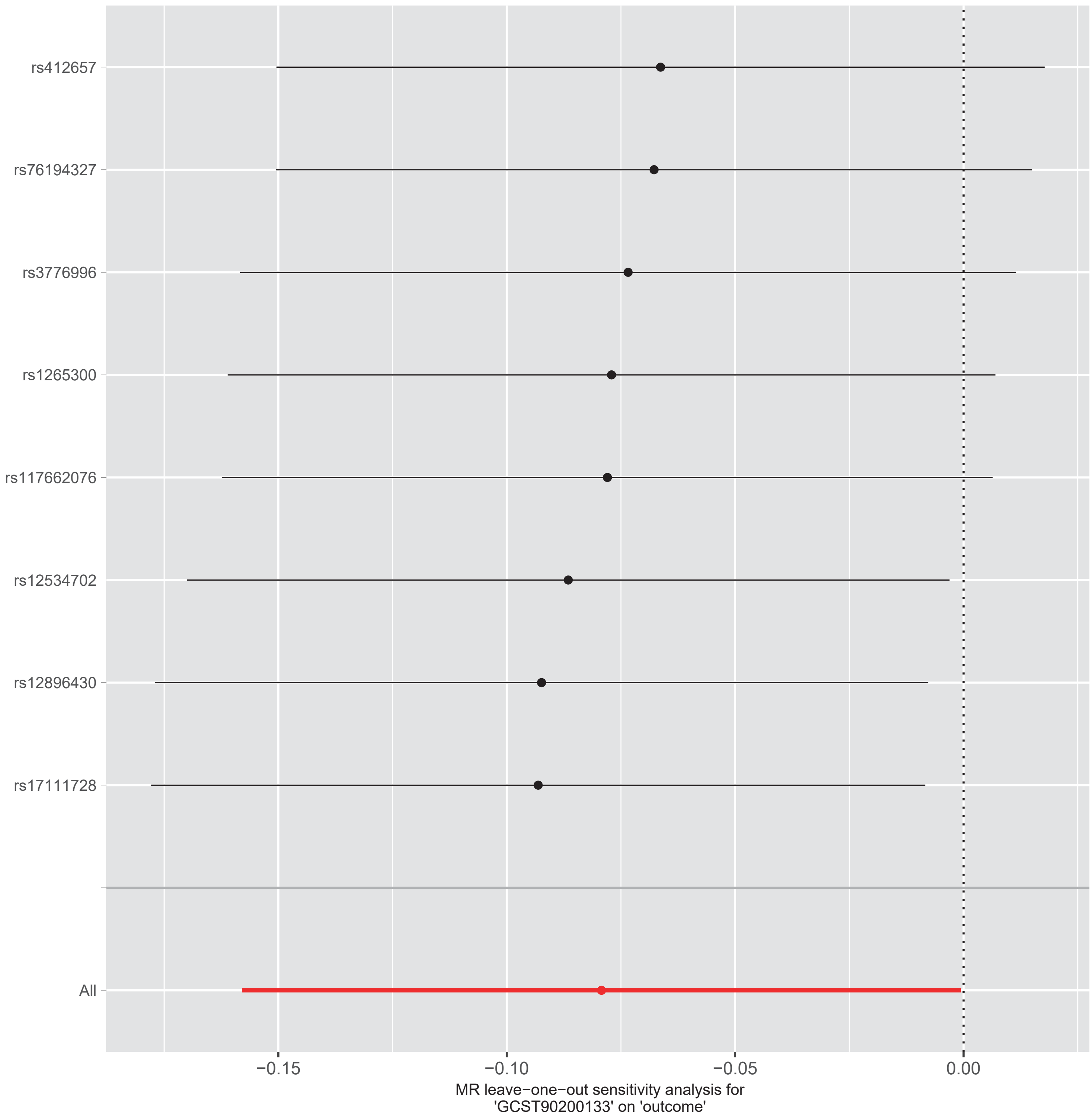

rs412657
rs76194327
rs3776996
rs1265300
rs117662076
rs12534702
rs12896430
rs17111728
All
−0.15
−0.10
−0.05
0.00
MR leave−one−out sensitivity analysis for
'GCST90200133' on 'outcome'

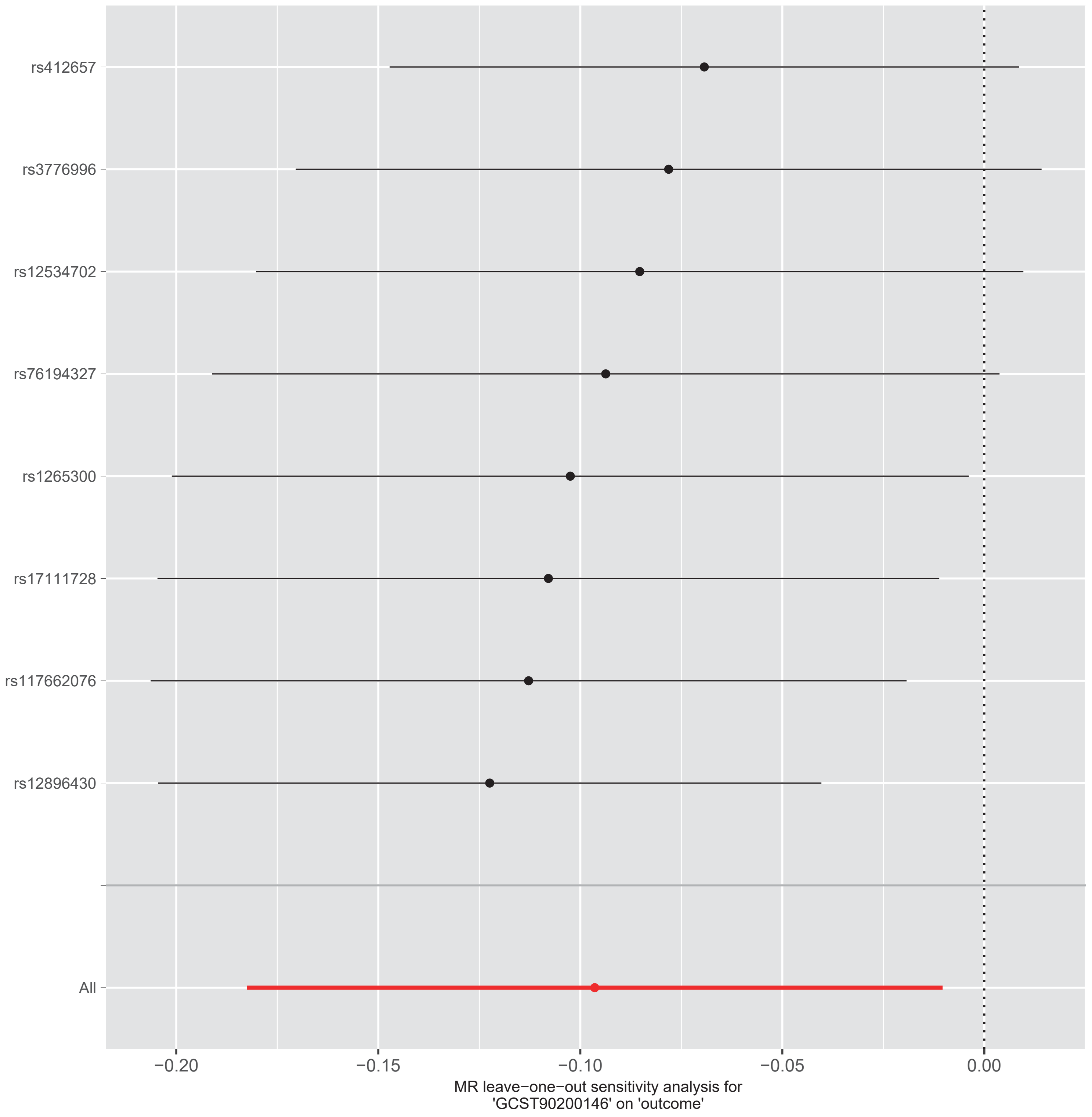
rs412657
rs3776996
rs12534702
rs76194327
rs1265300
rs17111728
rs117662076
rs12896430
All
−0.20
−0.15
−0.10
−0.05
0.00
MR leave−one−out sensitivity analysis for
'GCST90200146' on 'outcome'

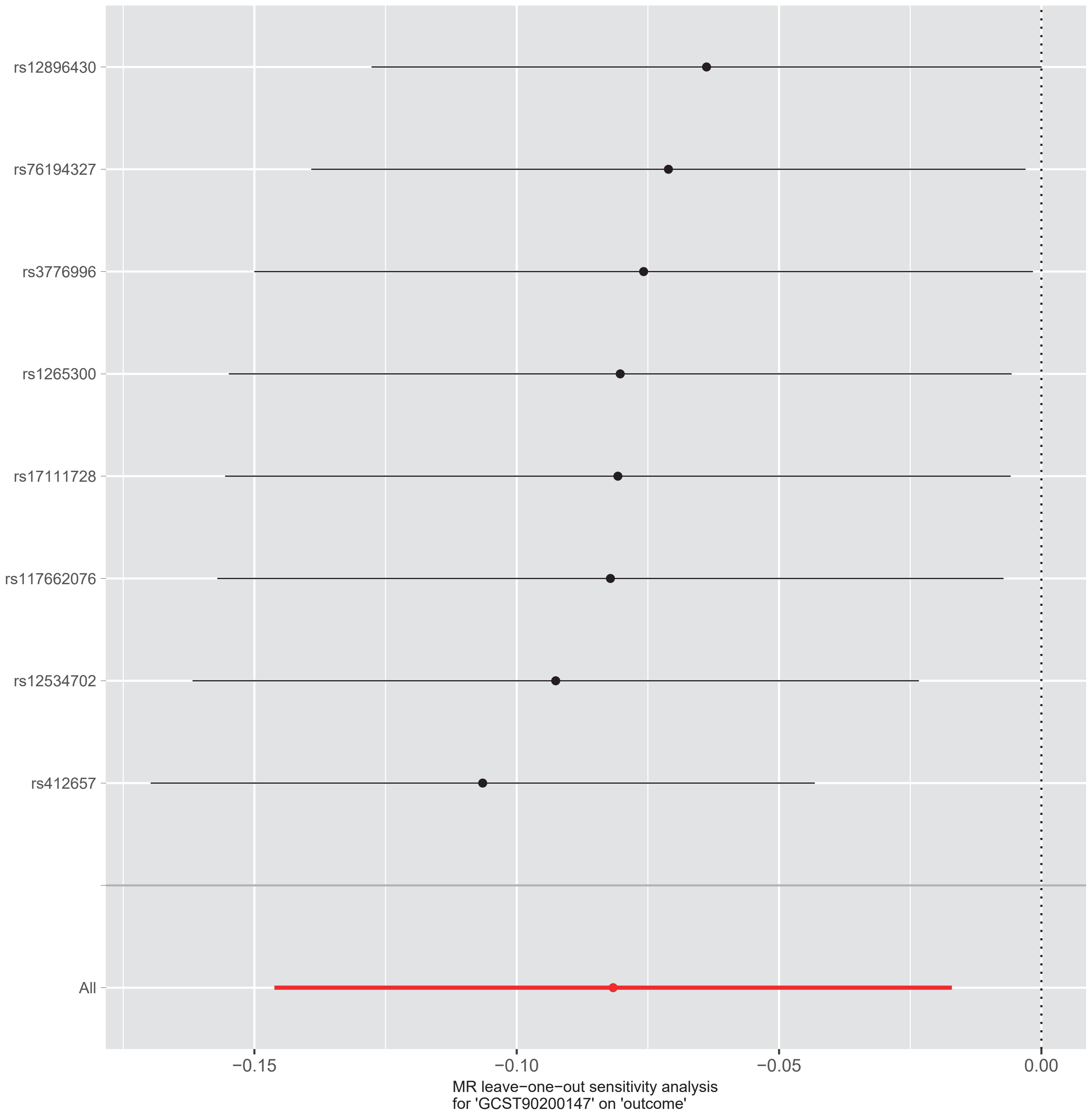

rs12896430
rs76194327
rs3776996
rs1265300
rs17111728
rs117662076
rs12534702
rs412657
All
−0.15
−0.10
−0.05
0.00
MR leave−one−out sensitivity analysis
for 'GCST90200147' on 'outcome'

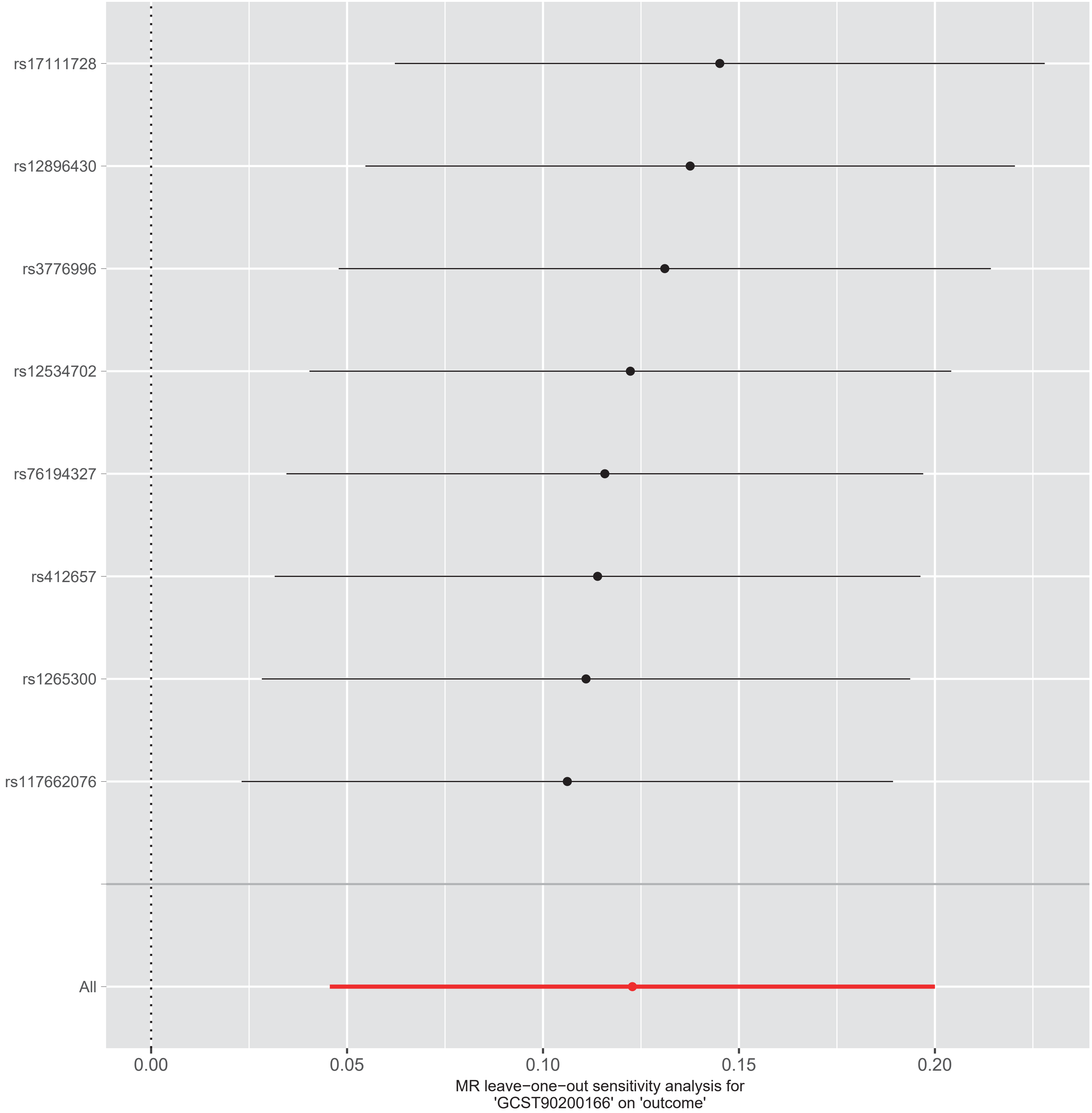

rs17111728
rs12896430
rs3776996
rs12534702
rs76194327
rs412657
rs1265300
rs117662076
All
0.00
0.05
0.10
0.15
0.20
MR leave−one−out sensitivity analysis for
'GCST90200166' on 'outcome'

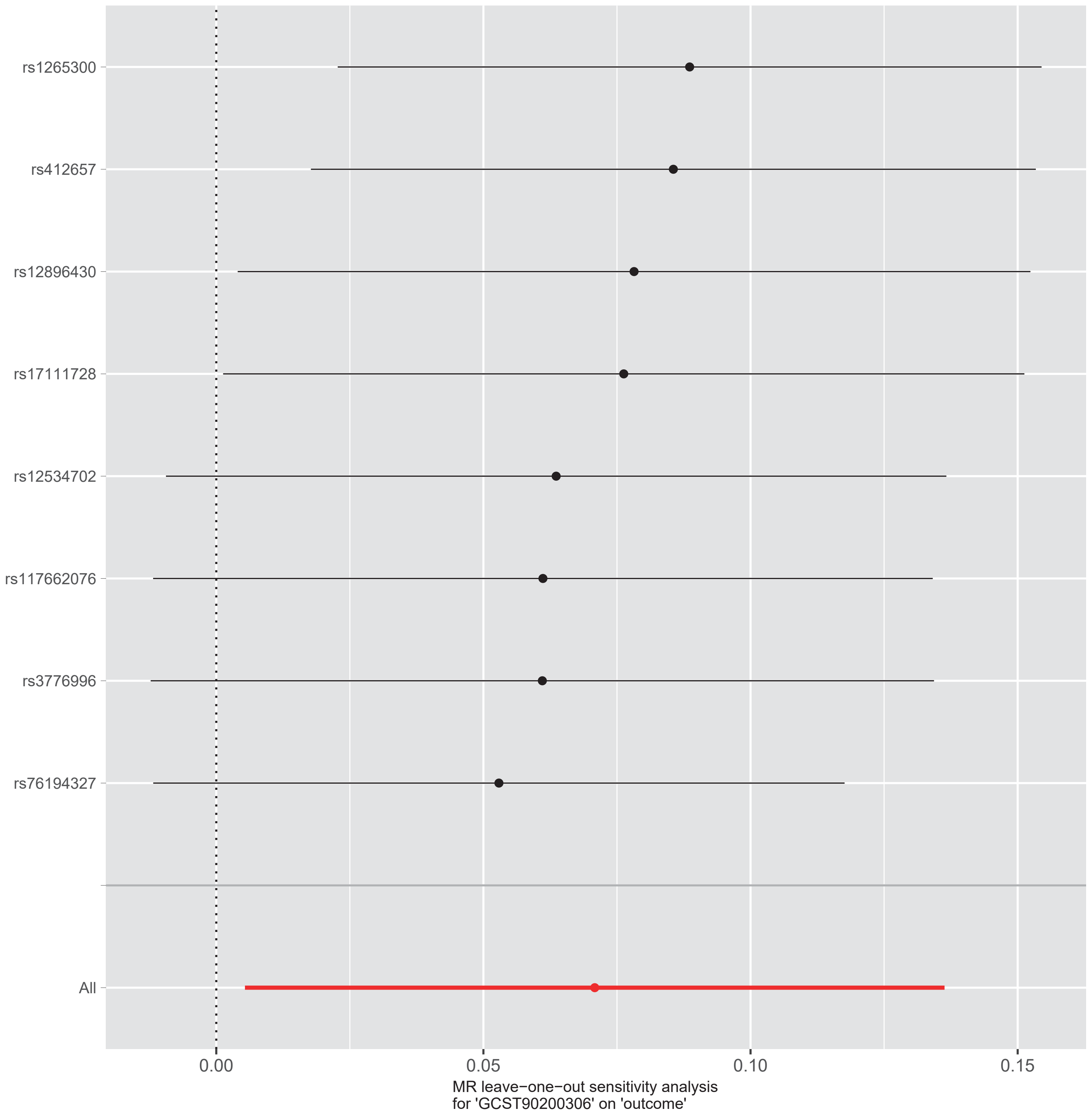

rs1265300
rs412657
rs12896430
rs17111728
rs12534702
rs117662076
rs3776996
rs76194327
All
0.00
0.05
0.10
0.15
MR leave−one−out sensitivity analysis
for 'GCST90200306' on 'outcome'

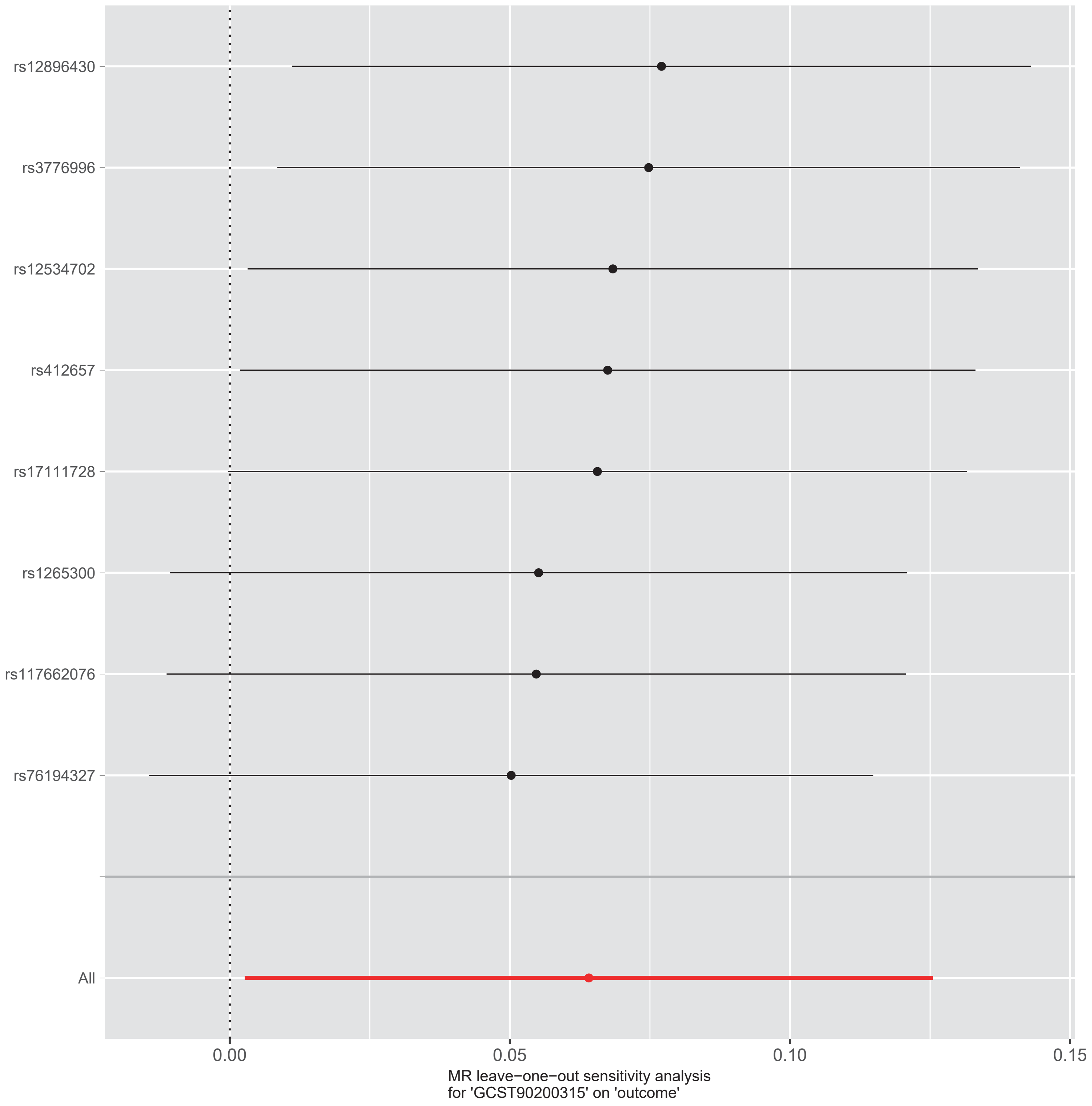
rs12896430
rs3776996
rs12534702
rs412657
rs17111728
rs1265300
rs117662076
rs76194327
All
0.00
0.05
0.10
0.15
MR leave−one−out sensitivity analysis
for 'GCST90200315' on 'outcome'

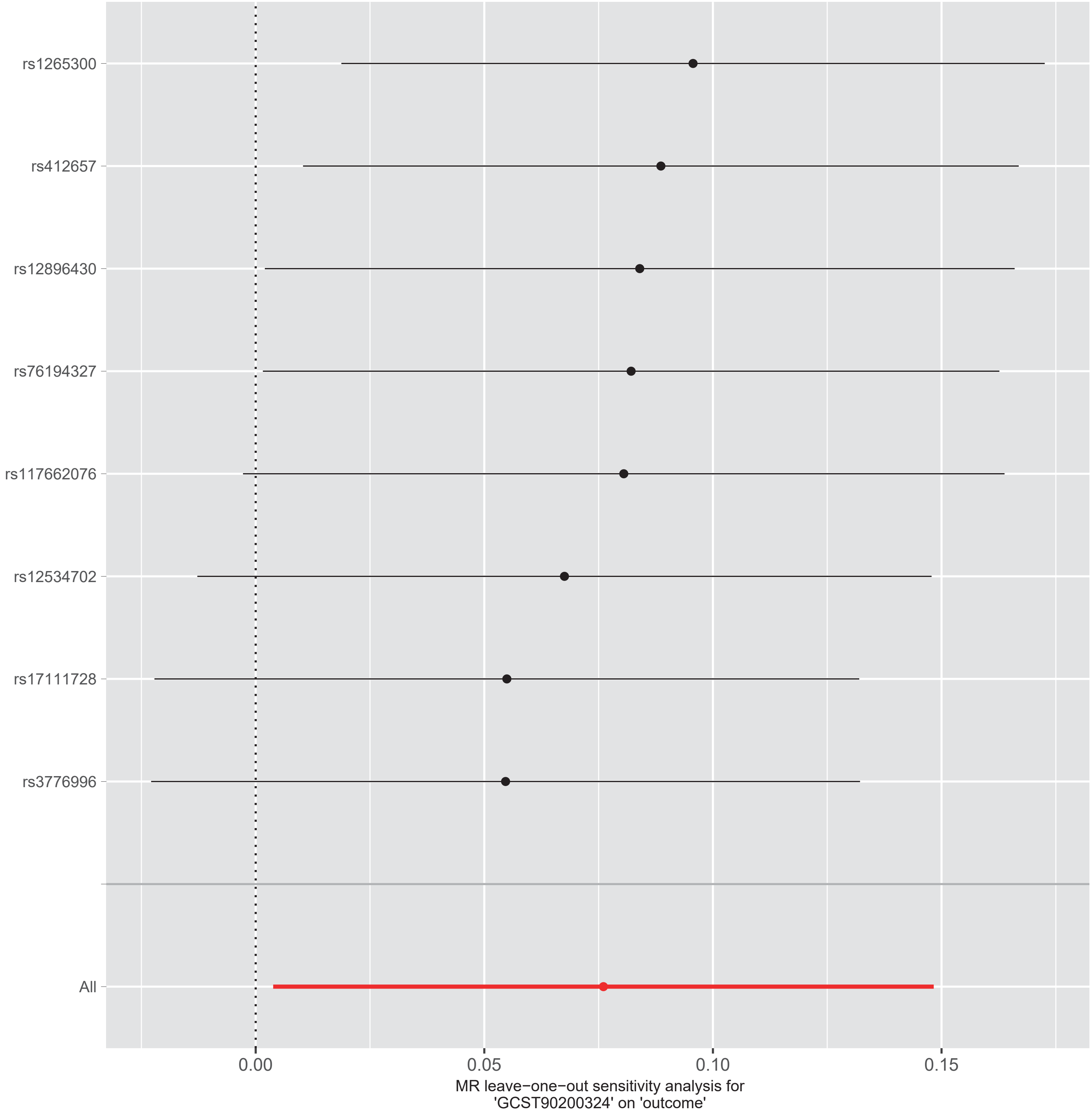
rs1265300
rs412657
rs12896430
rs76194327
rs117662076
rs12534702
rs17111728
rs3776996
All
0.00
0.05
0.10
0.15
MR leave−one−out sensitivity analysis for
'GCST90200324' on 'outcome'

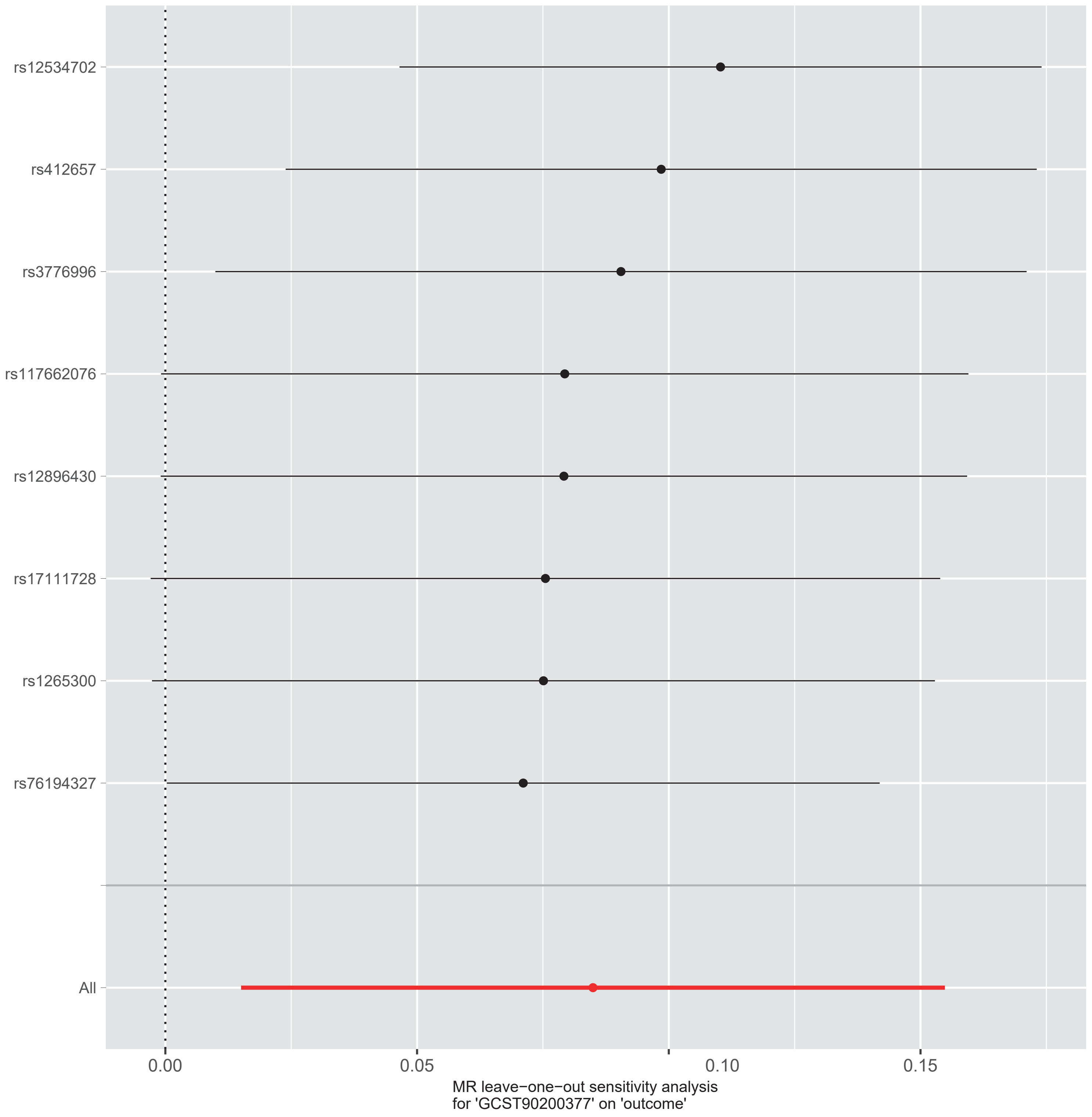
rs12534702
rs412657
rs3776996
rs117662076
rs12896430
rs17111728
rs1265300
rs76194327
All
0.00
0.05
0.10
0.15
MR leave−one−out sensitivity analysis
for 'GCST90200377' on 'outcome'

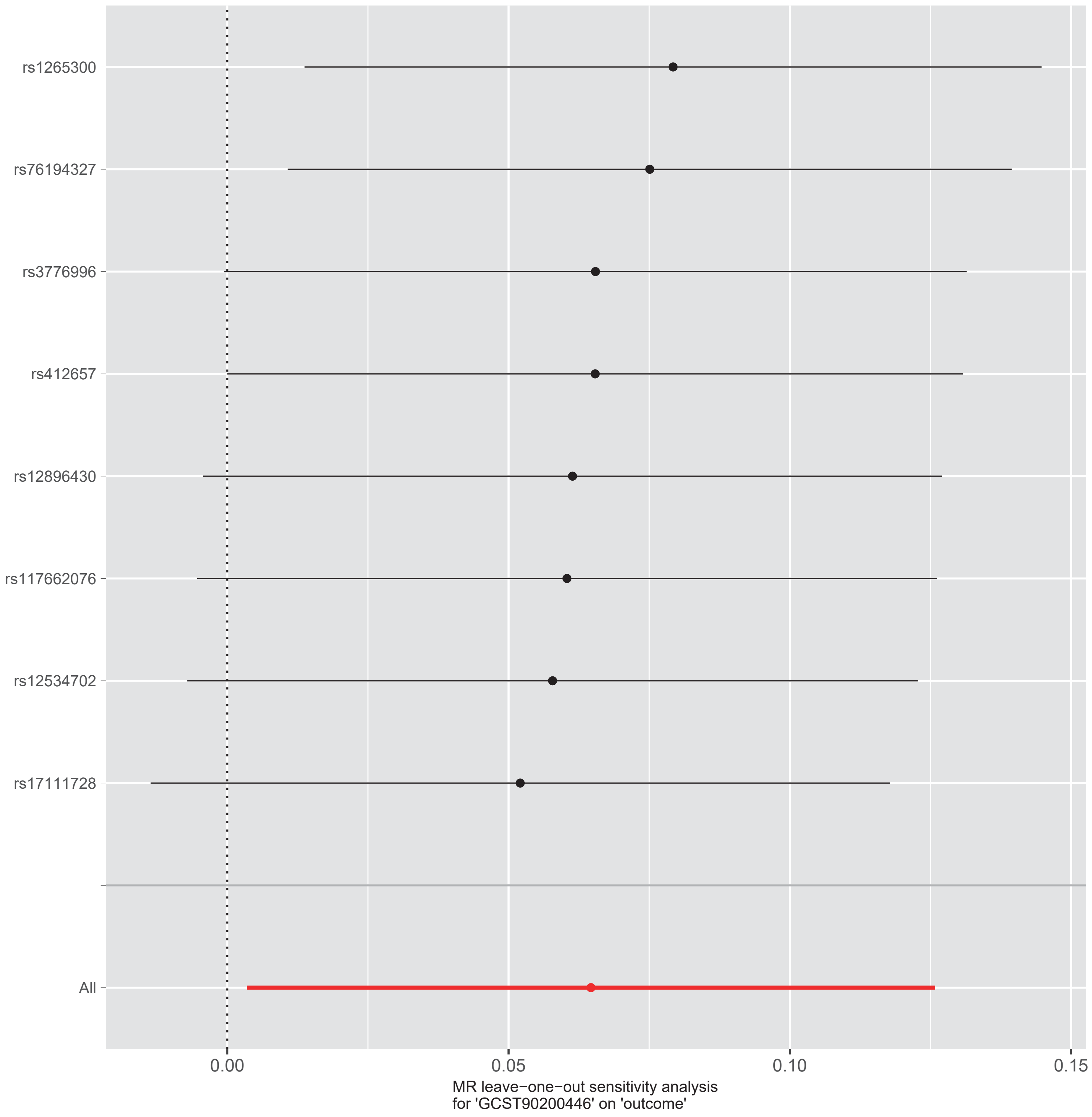
rs1265300
rs76194327
rs3776996
rs412657
rs12896430
rs117662076
rs12534702
rs17111728
All
0.00
0.05
0.10
0.15
MR leave−one−out sensitivity analysis
for 'GCST90200446' on 'outcome'

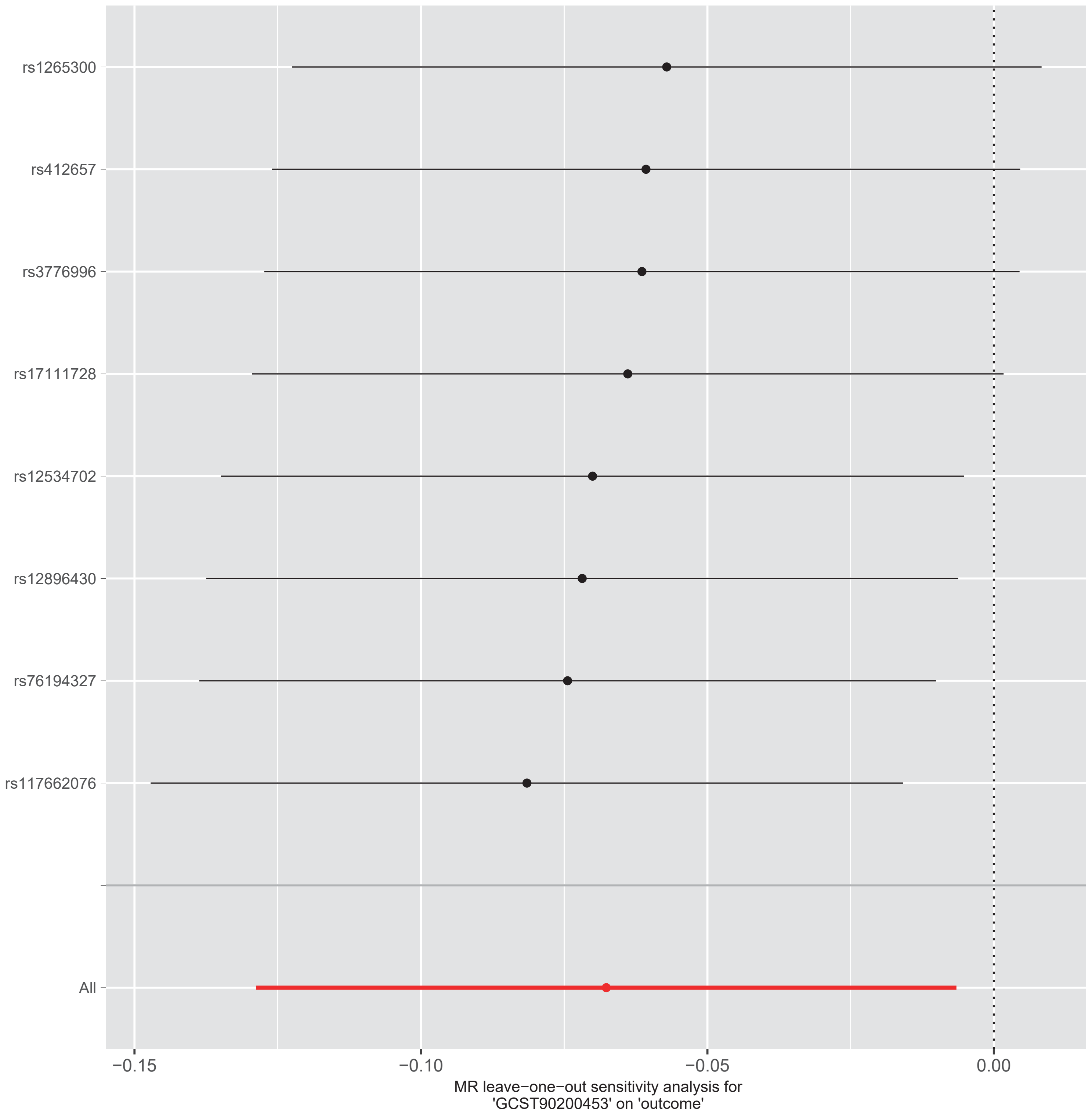
rs1265300
rs412657
rs3776996
rs17111728
rs12534702
rs12896430
rs76194327
rs117662076
All
−0.15
−0.10
−0.05
0.00
MR leave−one−out sensitivity analysis for
'GCST90200453' on 'outcome'

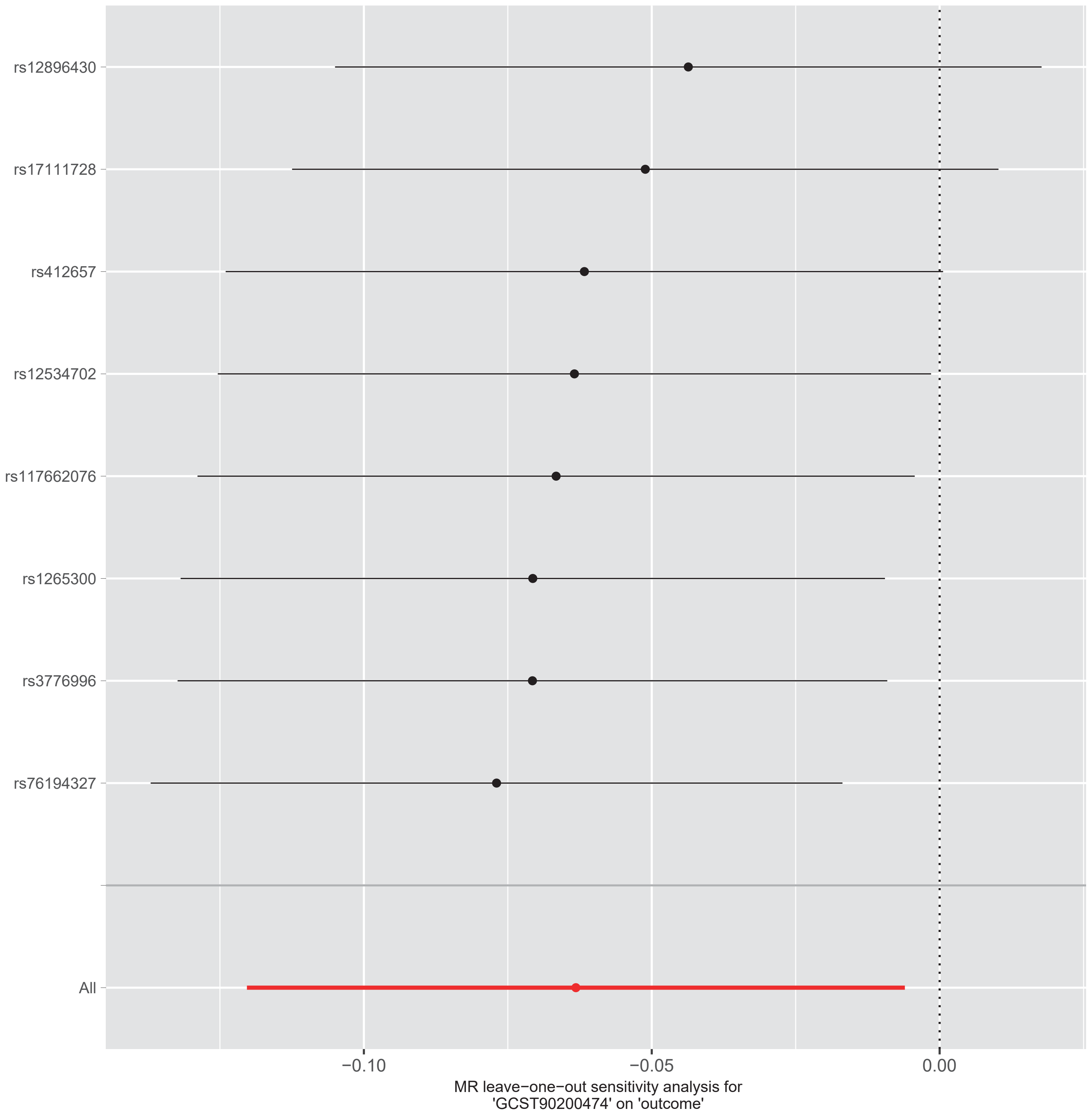
rs12896430
rs17111728
rs412657
rs12534702
rs117662076
rs1265300
rs3776996
rs76194327
All
−0.10
−0.05
0.00
MR leave−one−out sensitivity analysis for
'GCST90200474' on 'outcome'

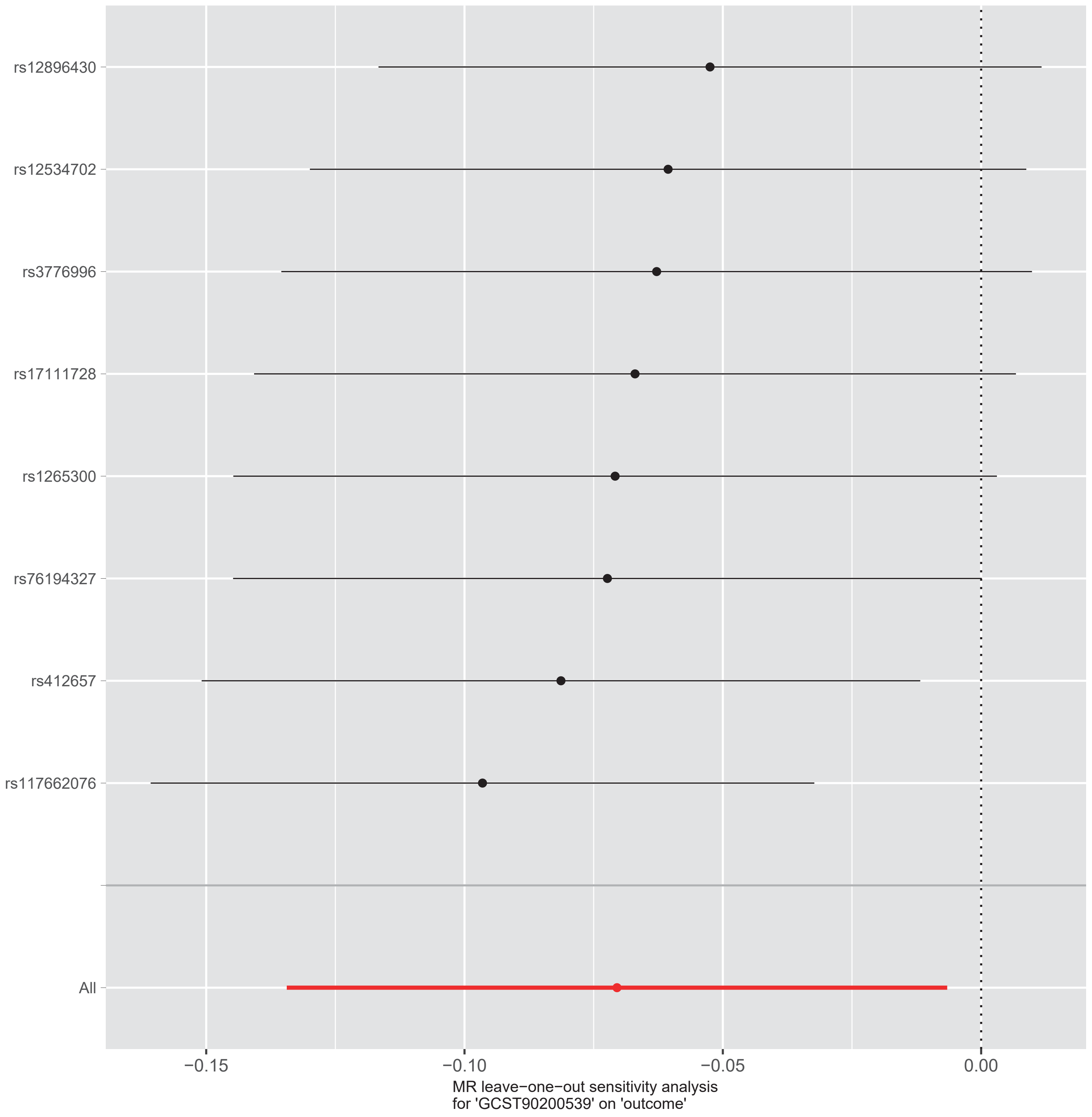
rs12896430
rs12534702
rs3776996
rs17111728
rs1265300
rs76194327
rs412657
rs117662076
All
−0.15
−0.10
−0.05
0.00
MR leave−one−out sensitivity analysis
for 'GCST90200539' on 'outcome'

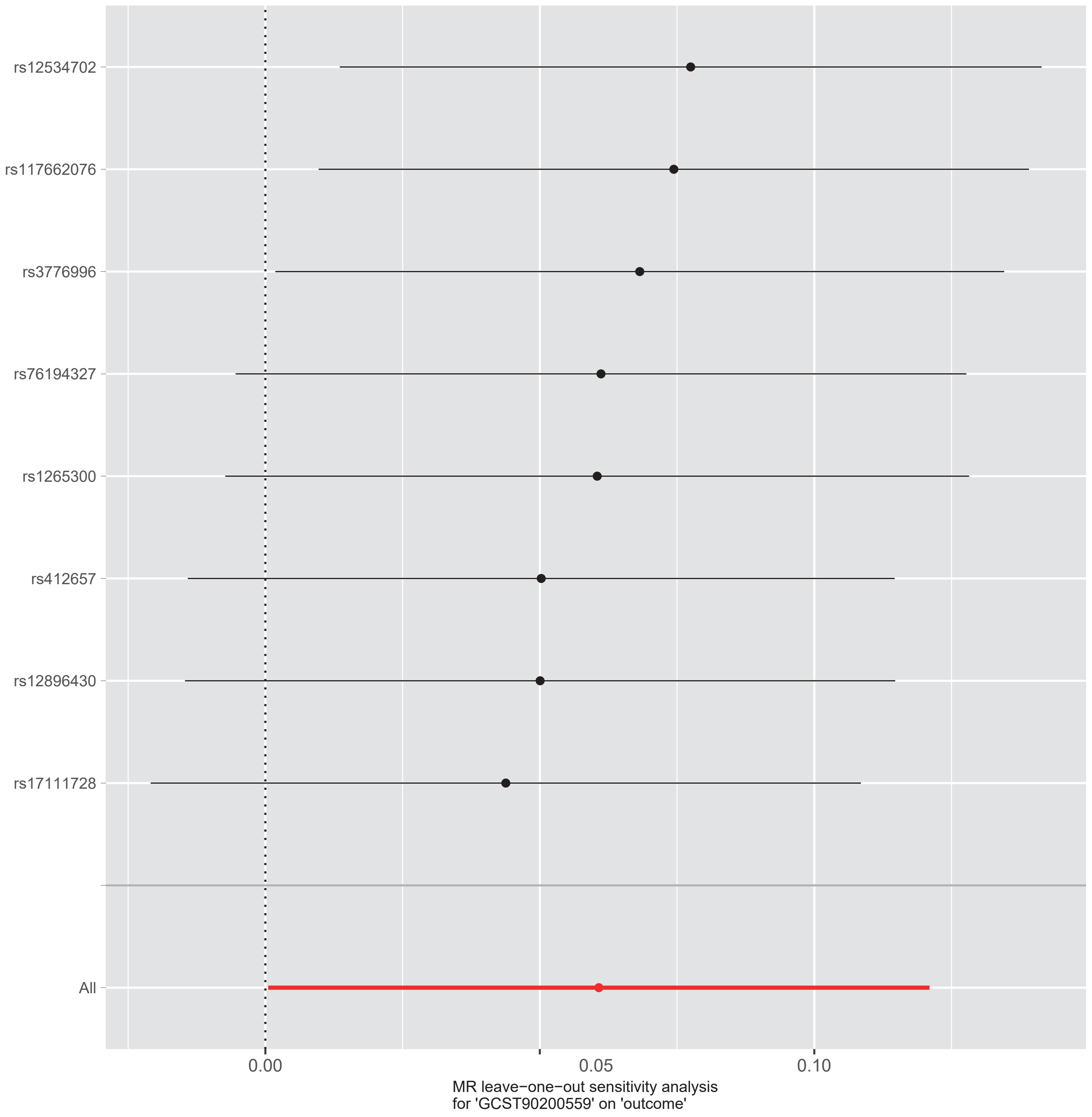
rs12534702
rs117662076
rs3776996
rs76194327
rs1265300
rs412657
rs12896430
rs17111728
All
0.00
0.05
0.10
MR leave−one−out sensitivity analysis
for 'GCST90200559' on 'outcome'

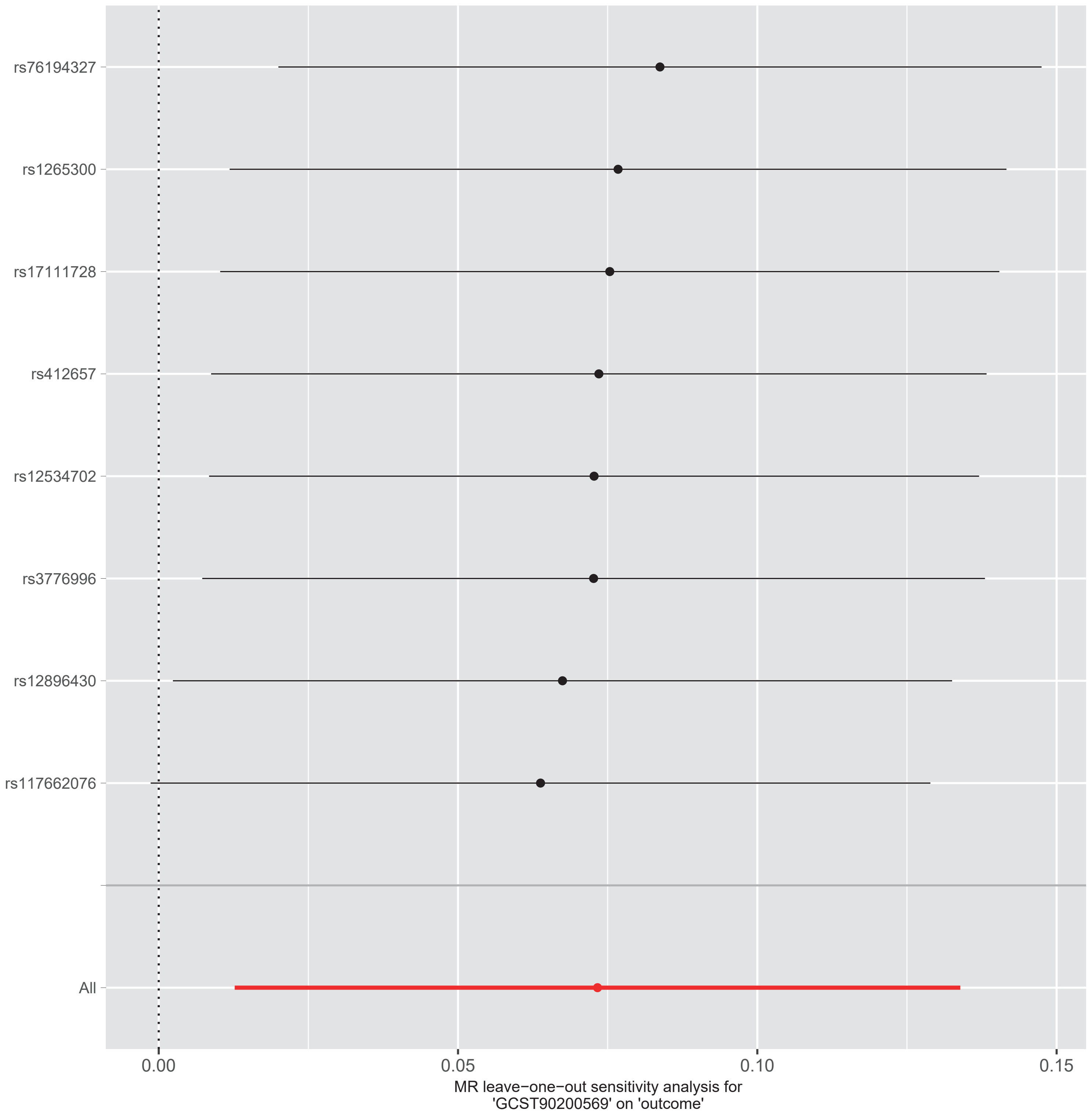
rs76194327
rs1265300
rs17111728
rs412657
rs12534702
rs3776996
rs12896430
rs117662076
All
0.00
0.05
0.10
0.15
MR leave−one−out sensitivity analysis for
'GCST90200569' on 'outcome'

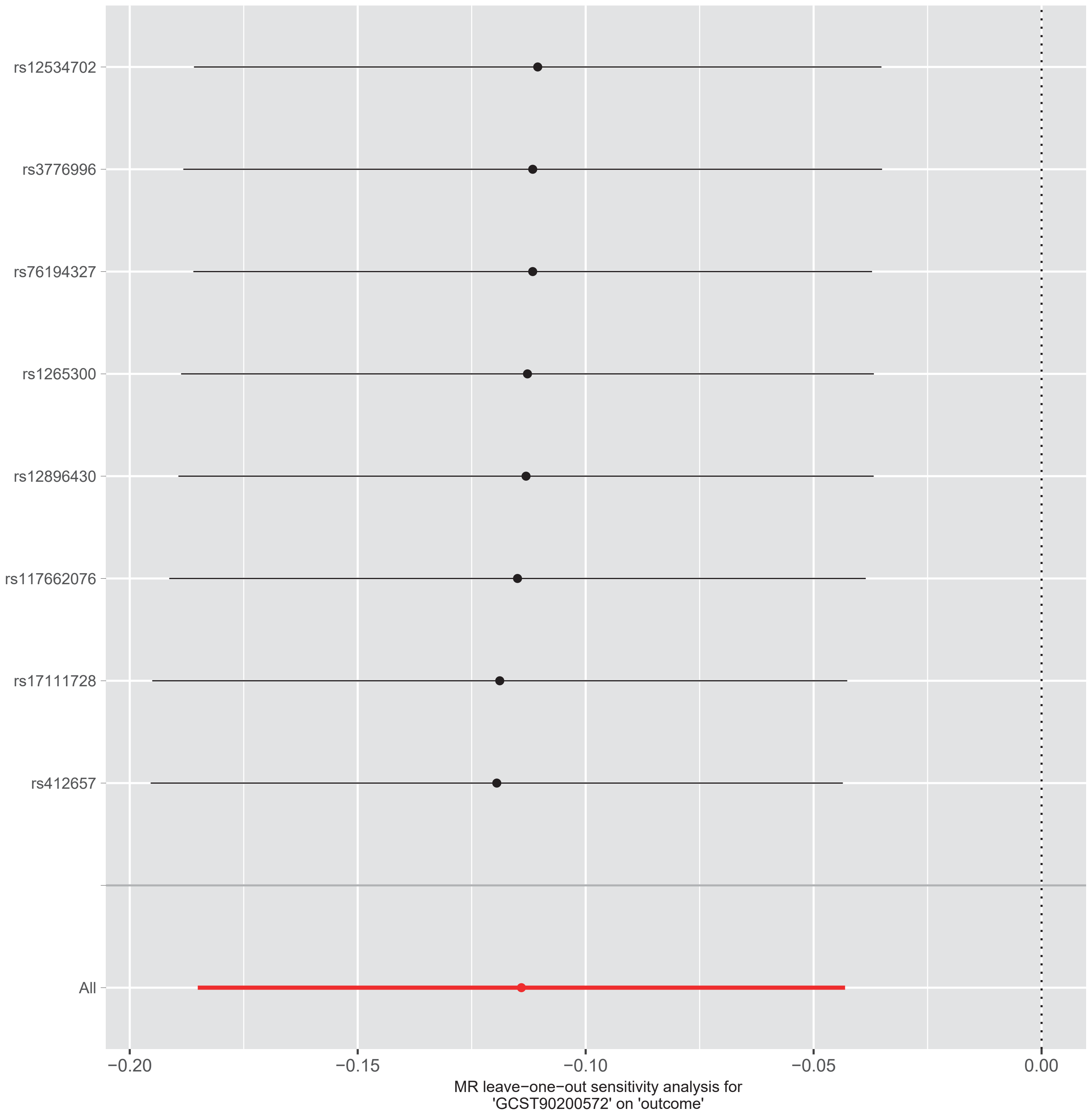
rs12534702
rs3776996
rs76194327
rs1265300
rs12896430
rs117662076
rs17111728
rs412657
All
−0.20
−0.15
−0.10
−0.05
0.00
MR leave−one−out sensitivity analysis for
'GCST90200572' on 'outcome'

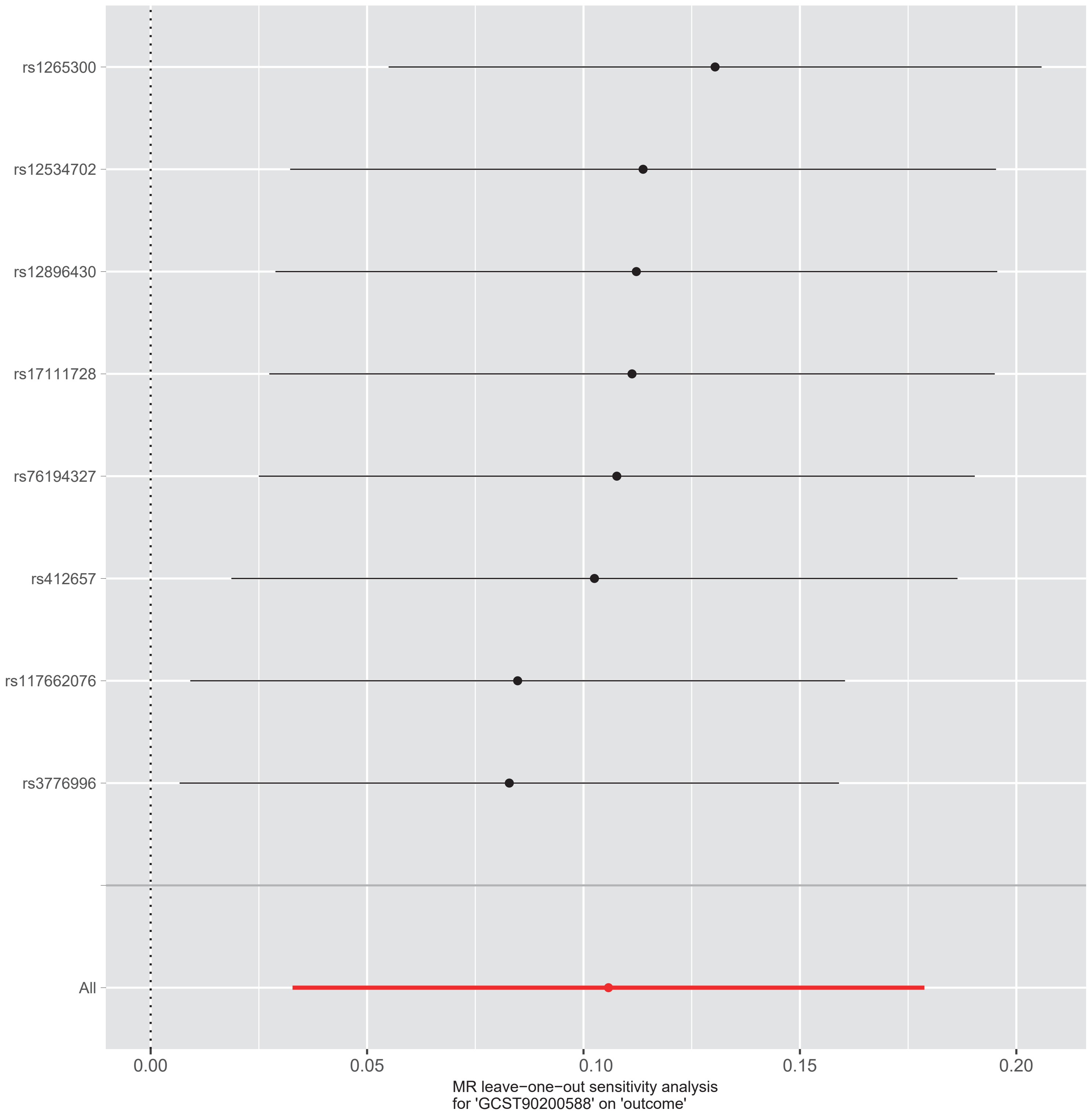
rs1265300
rs12534702
rs12896430
rs17111728
rs76194327
rs412657
rs117662076
rs3776996
All
0.00
0.05
0.10
0.15
0.20
MR leave−one−out sensitivity analysis
for 'GCST90200588' on 'outcome'

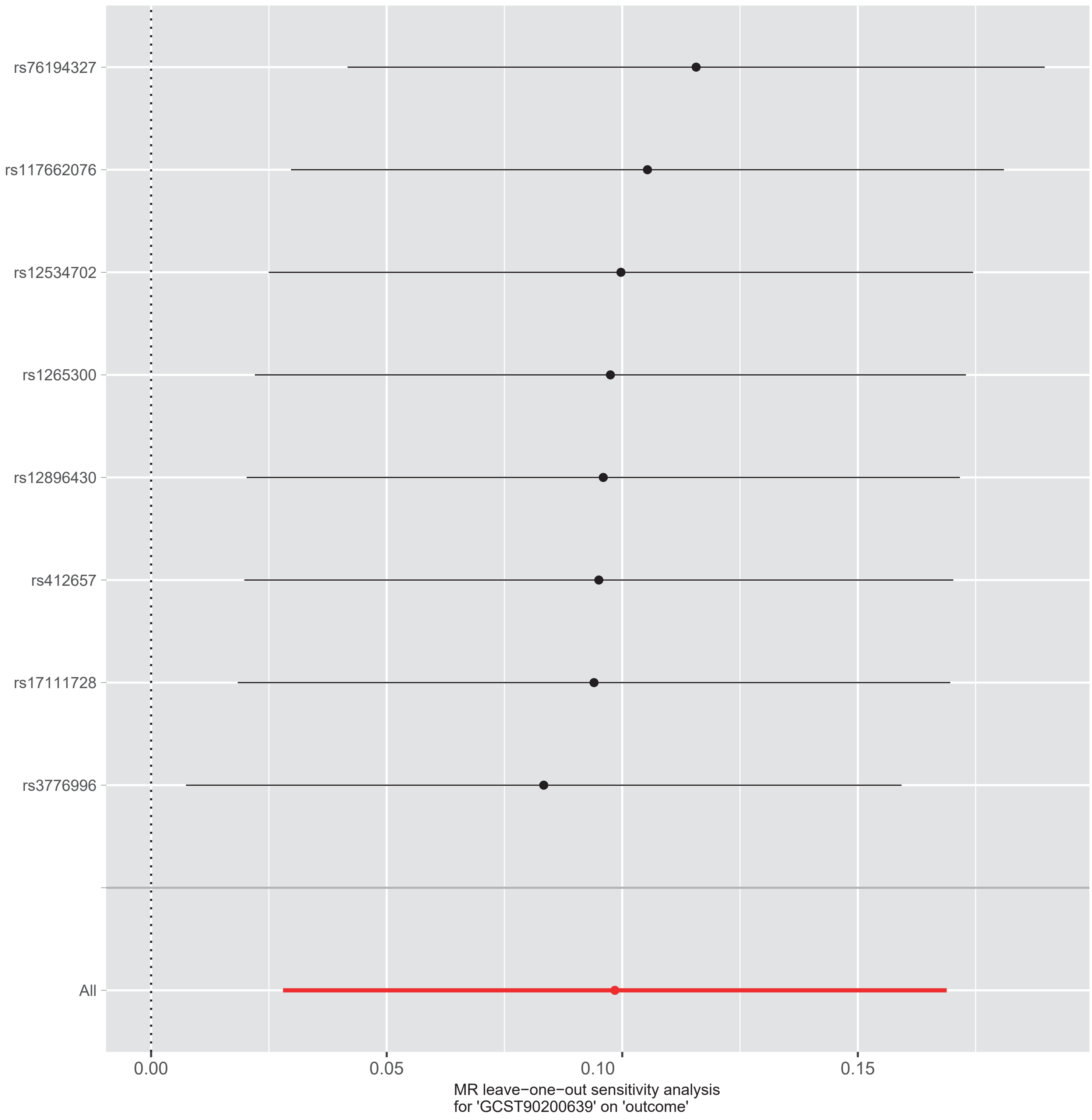

rs76194327
rs117662076
rs12534702
rs1265300
rs12896430
rs412657
rs17111728
rs3776996
All
0.00
0.05
0.10
0.15
MR leave−one−out sensitivity analysis
for 'GCST90200639' on 'outcome'

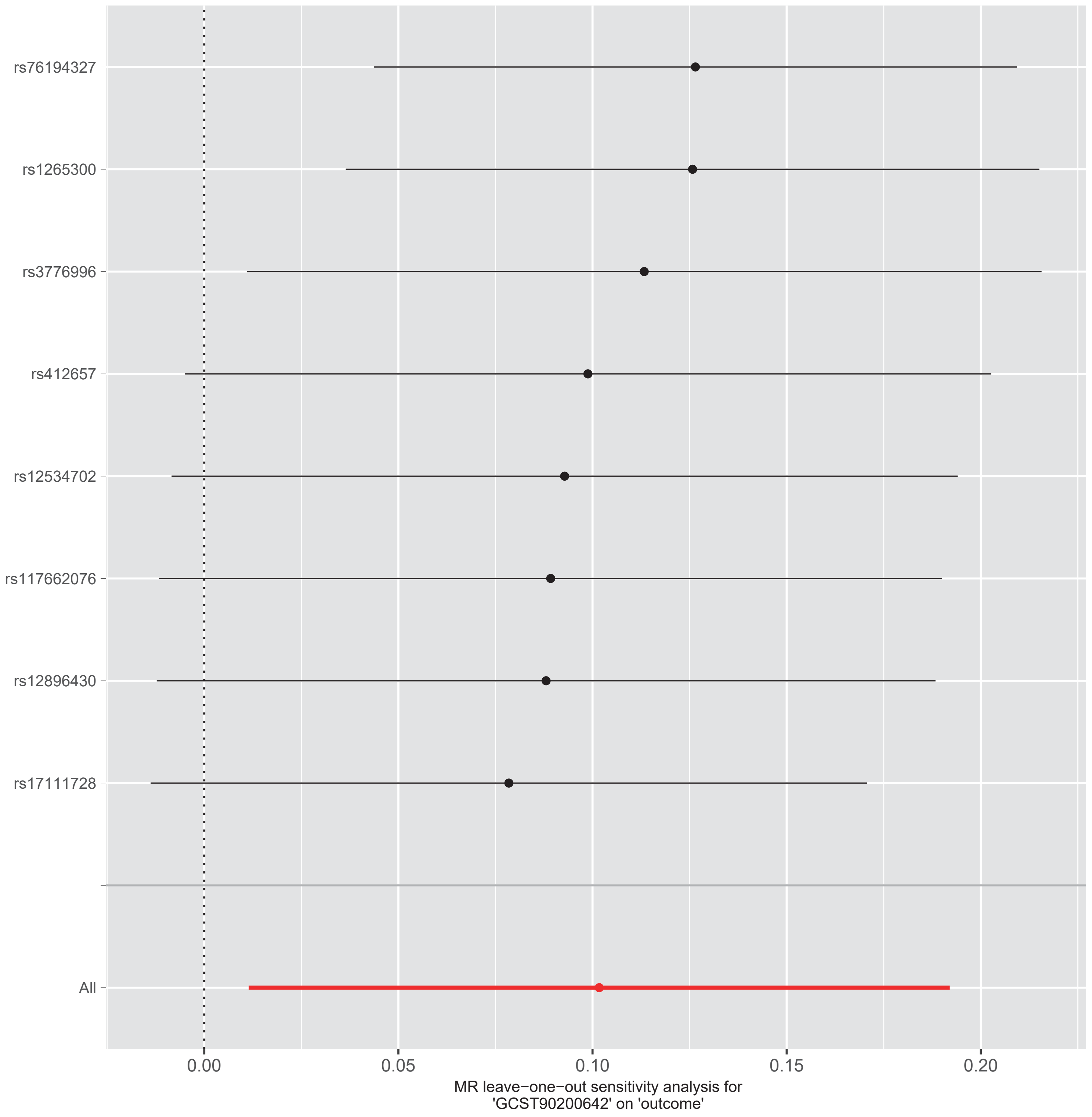
rs76194327
rs1265300
rs3776996
rs412657
rs12534702
rs117662076
rs12896430
rs17111728
All
0.00
0.05
0.10
0.15
0.20
MR leave−one−out sensitivity analysis for
'GCST90200642' on 'outcome'

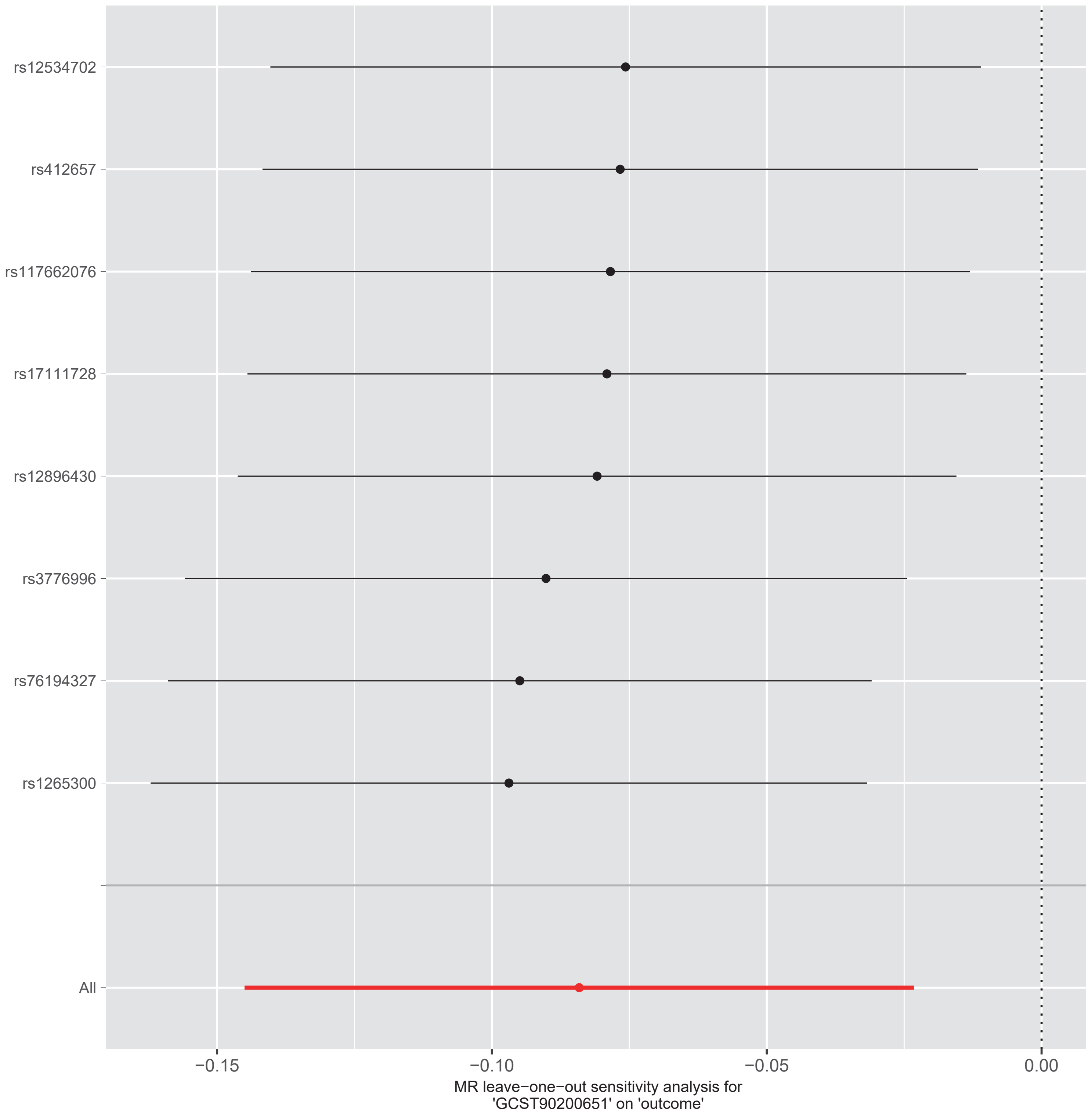

rs12534702
rs412657
rs117662076
rs17111728
rs12896430
rs3776996
rs76194327
rs1265300
All
−0.15
−0.10
−0.05
0.00
MR leave−one−out sensitivity analysis for
'GCST90200651' on 'outcome'

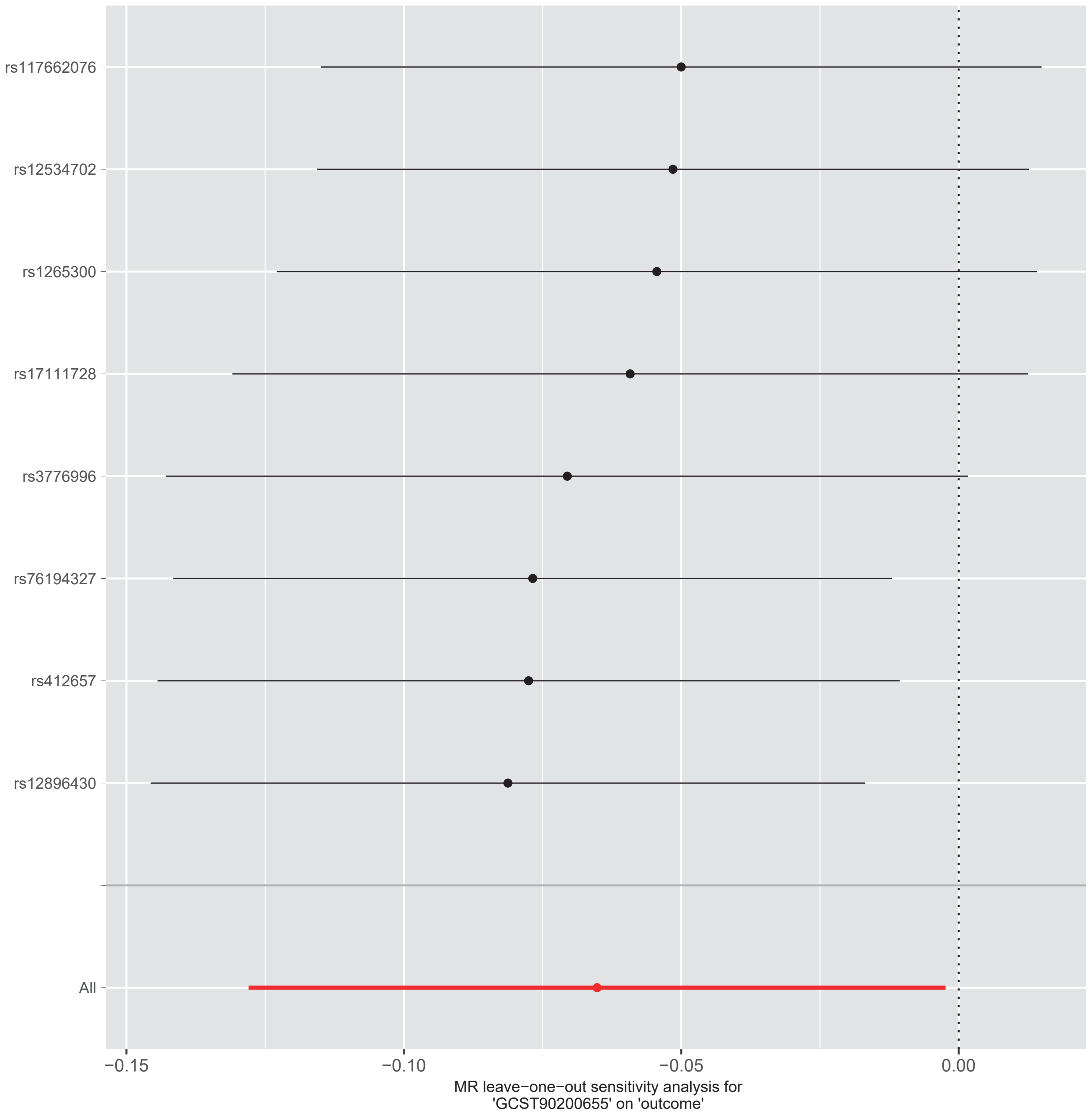
rs117662076
rs12534702
rs1265300
rs17111728
rs3776996
rs76194327
rs412657
rs12896430
All
−0.15
−0.10
−0.05
0.00
MR leave−one−out sensitivity analysis for
'GCST90200655' on 'outcome'

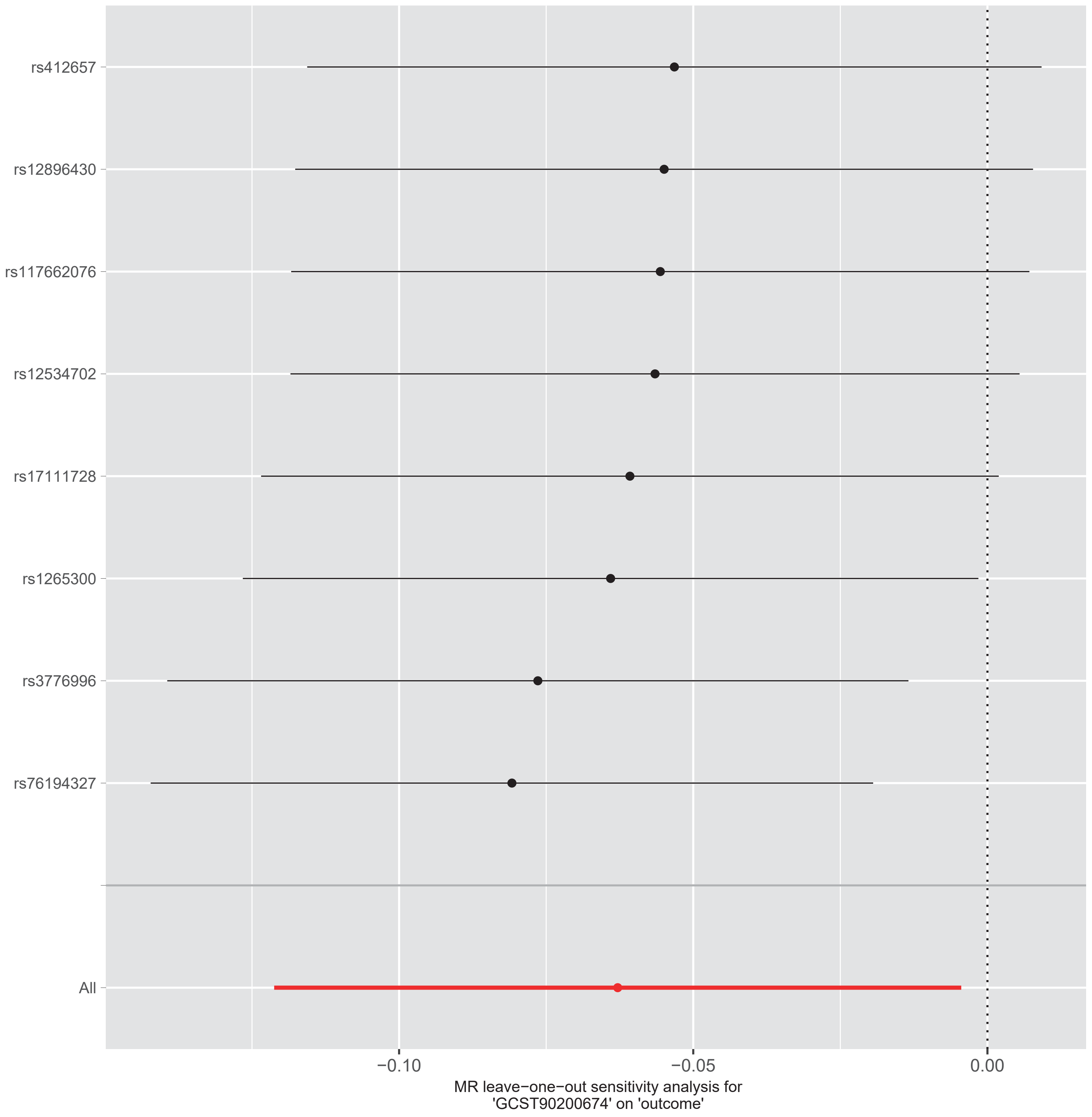
rs412657
rs12896430
rs117662076
rs12534702
rs17111728
rs1265300
rs3776996
rs76194327
All
−0.10
−0.05
0.00
MR leave−one−out sensitivity analysis for
'GCST90200674' on 'outcome'
