# Supplementary material for: Dissecting Causal Relationships Between Gut Microbiota, Plasma Metabolites and Bladder Cancer: A Two‐Step Mendelian Randomization Study
Source: Health Sci Rep. 2025 Sep 9;8(9):e71206. doi: 10.1002/hsr2.71206 (PMC12420358; doi:10.1002/hsr2.71206)
Supplement: Supplementary file 9 — Figure S9: Funnel plots for MR causal effects of significantly plasma metabolites on Bca. [file HSR2-8-e71206-s006.pdf]

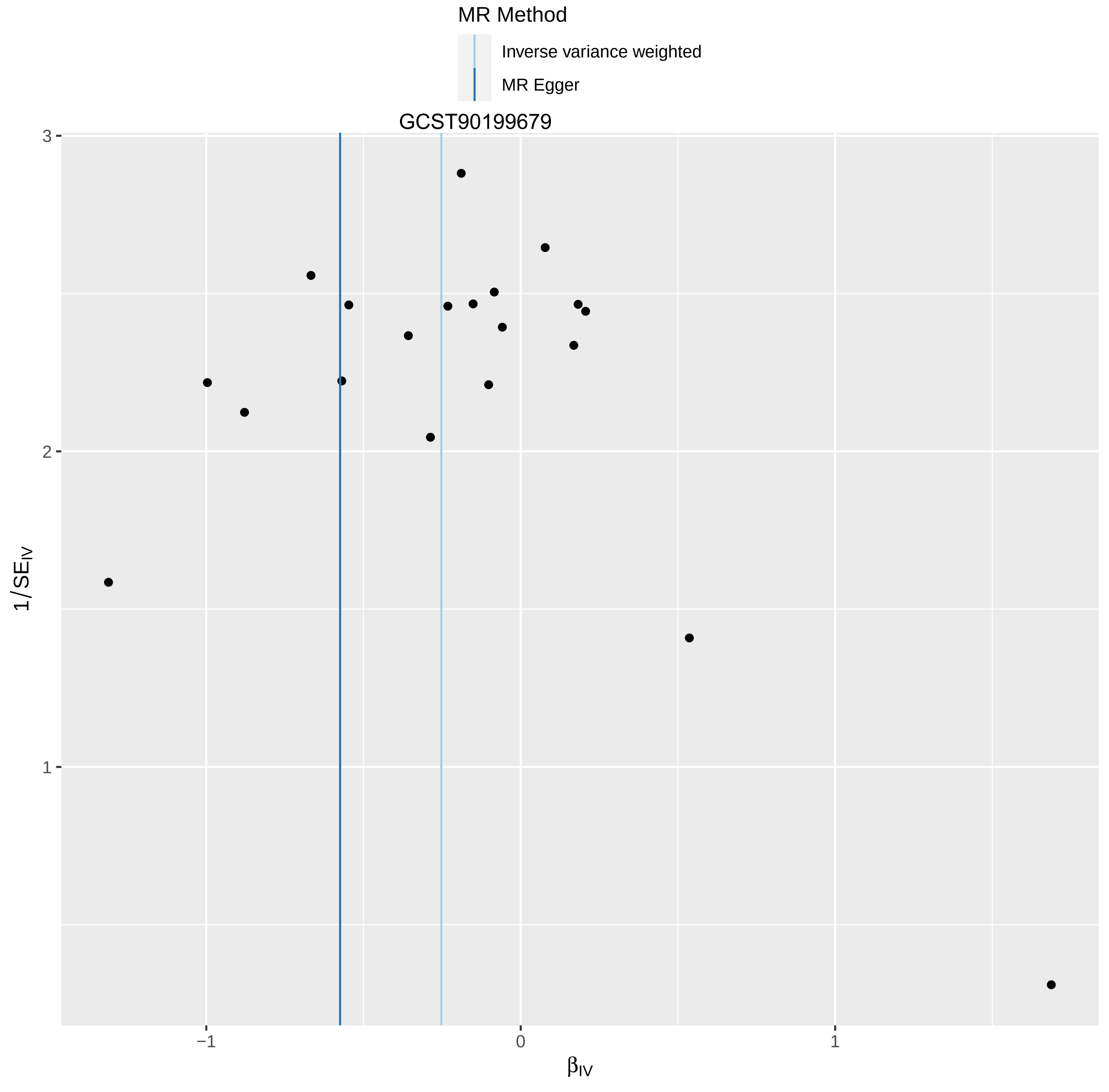
MR Method
Inverse variance weighted
MR Egger
GCST90199679
1/SE_IV
β_IV
3
2
1
−1
0
1

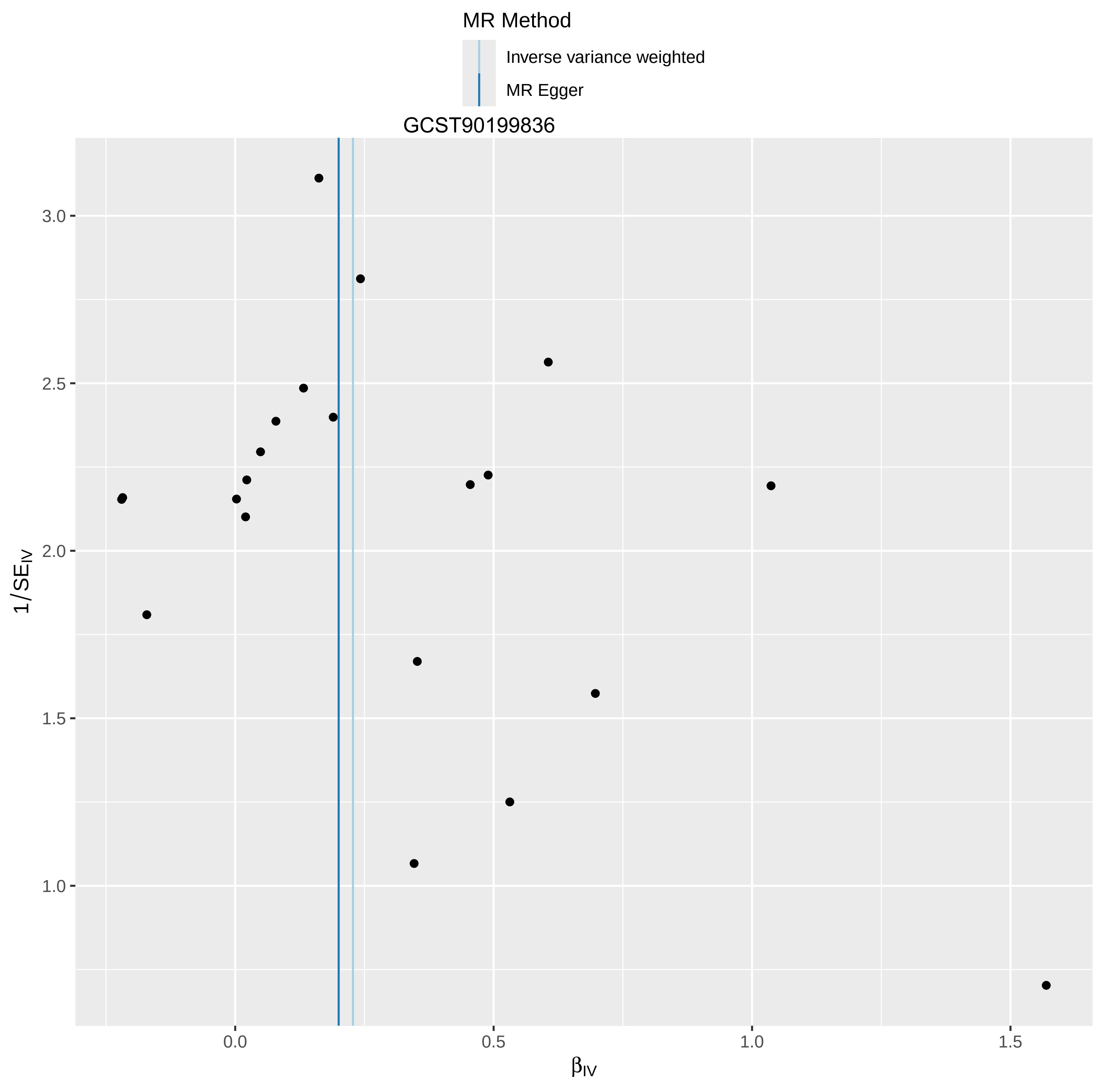
MR Method
Inverse variance weighted
MR Egger
GCST90199836
3.0
2.5
2.0
1.5
1.0
0.0
0.5
1.0
1.5
1/SE_IV
β_IV

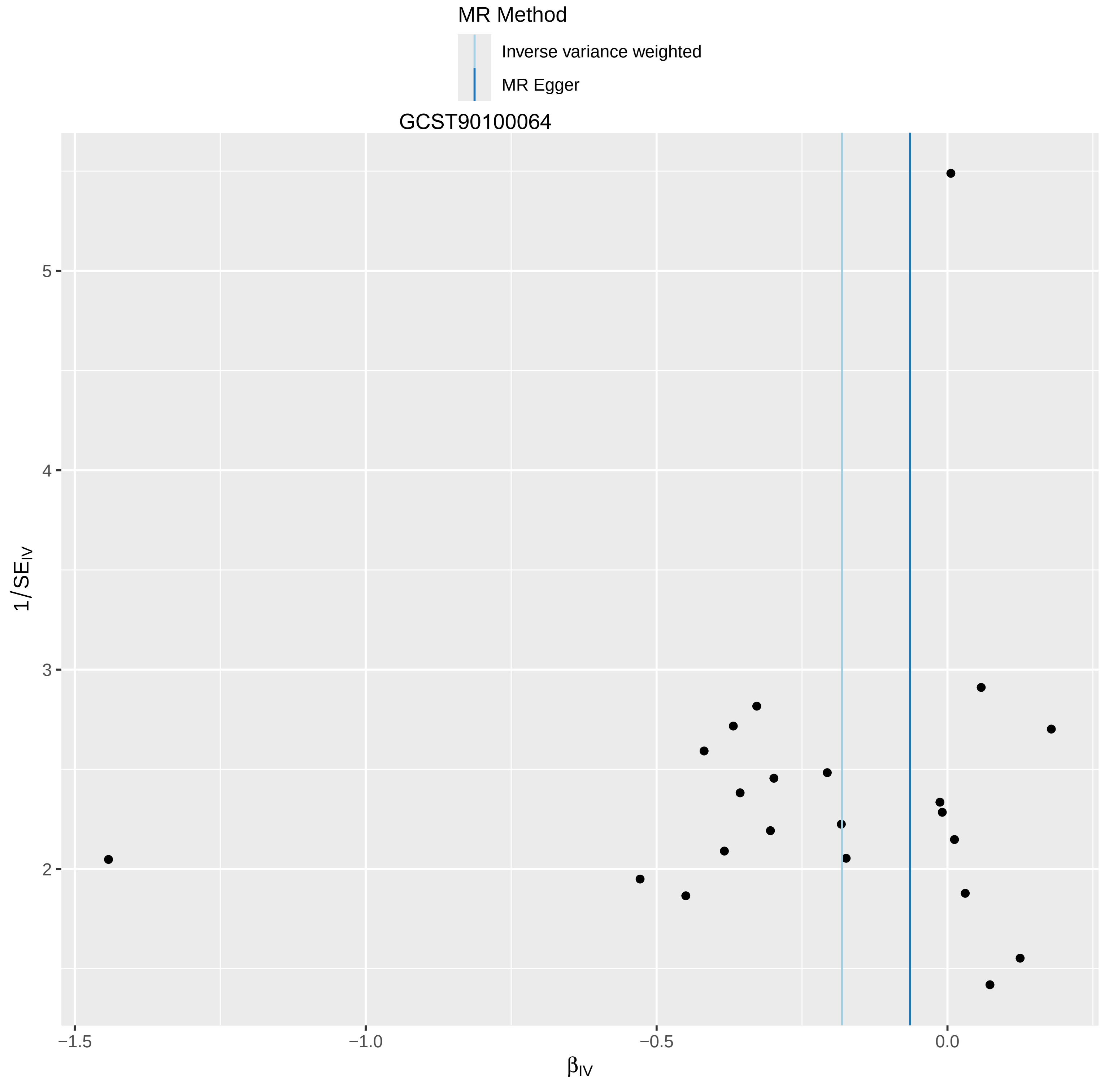
MR Method
Inverse variance weighted
MR Egger
GCST90100064
1/SE_IV
β_IV
5
4
3
2
−1.5
−1.0
−0.5
0.0

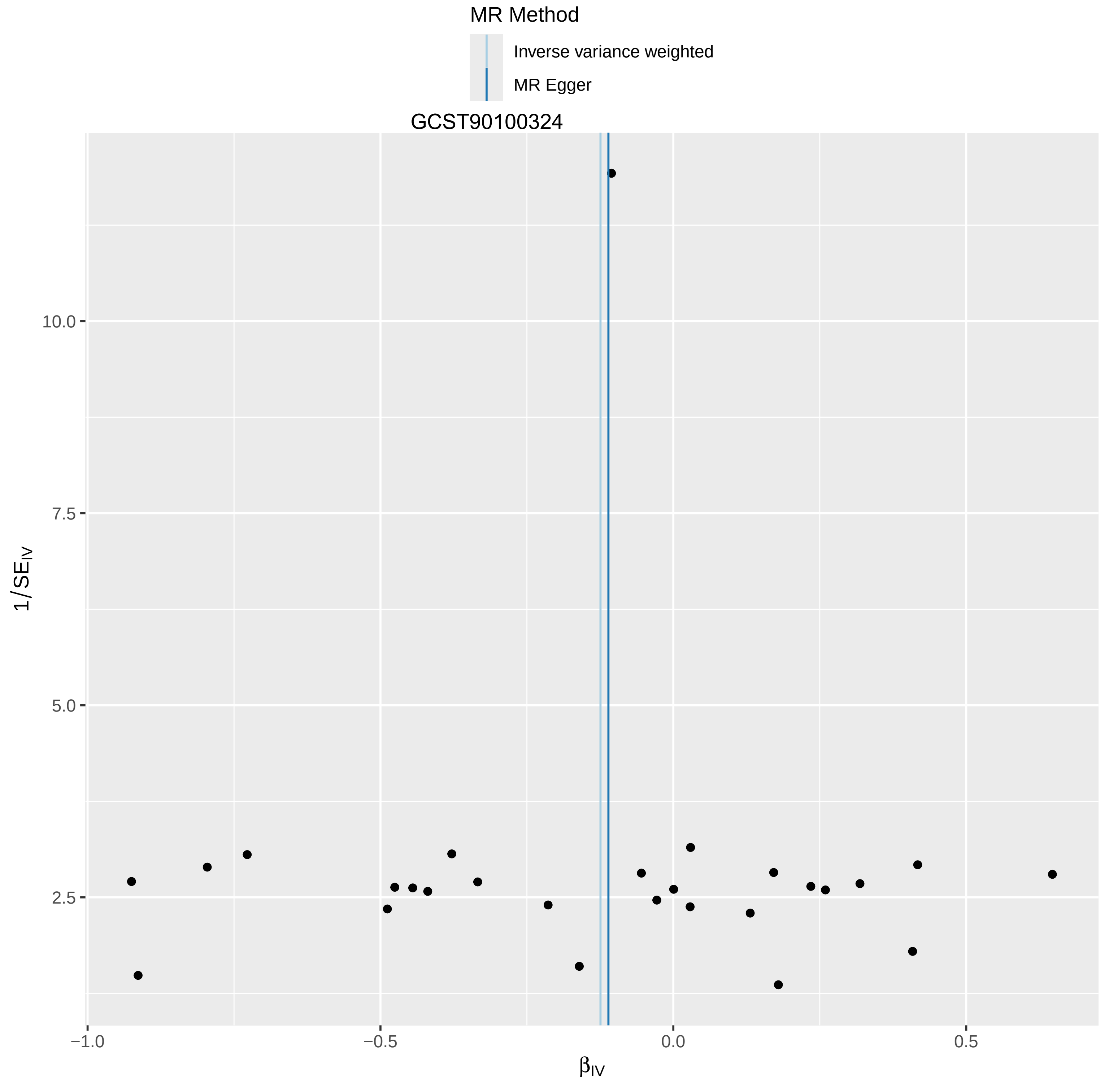

MR Method
Inverse variance weighted
MR Egger
GCST90100324
10.0
7.5
5.0
2.5
1/SE_IV
−1.0
−0.5
0.0
0.5
β_IV

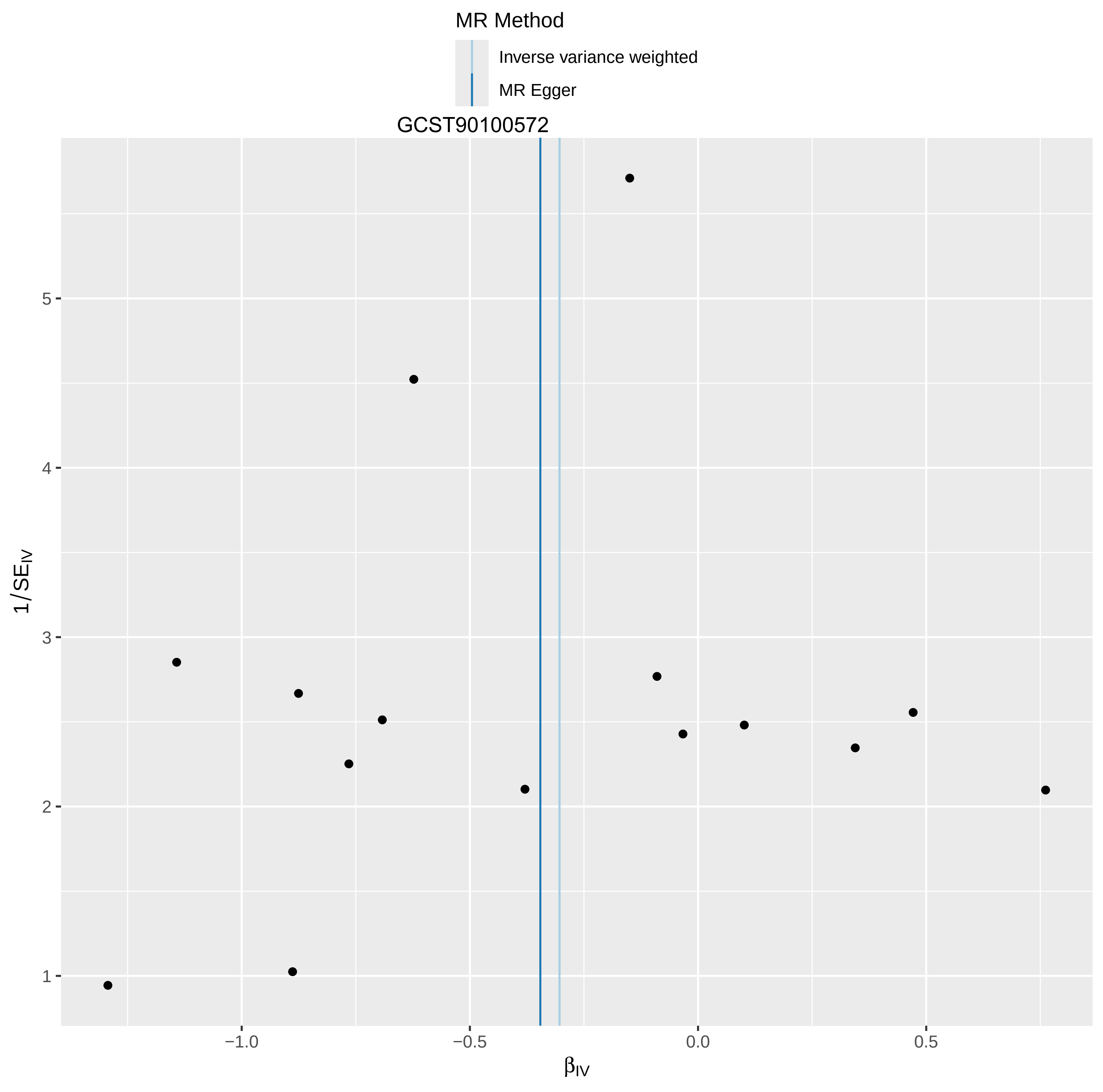

MR Method
Inverse variance weighted
MR Egger
GCST90100572
1/SE_IV
β_IV
−1.0
−0.5
0.0
0.5
1
2
3
4
5

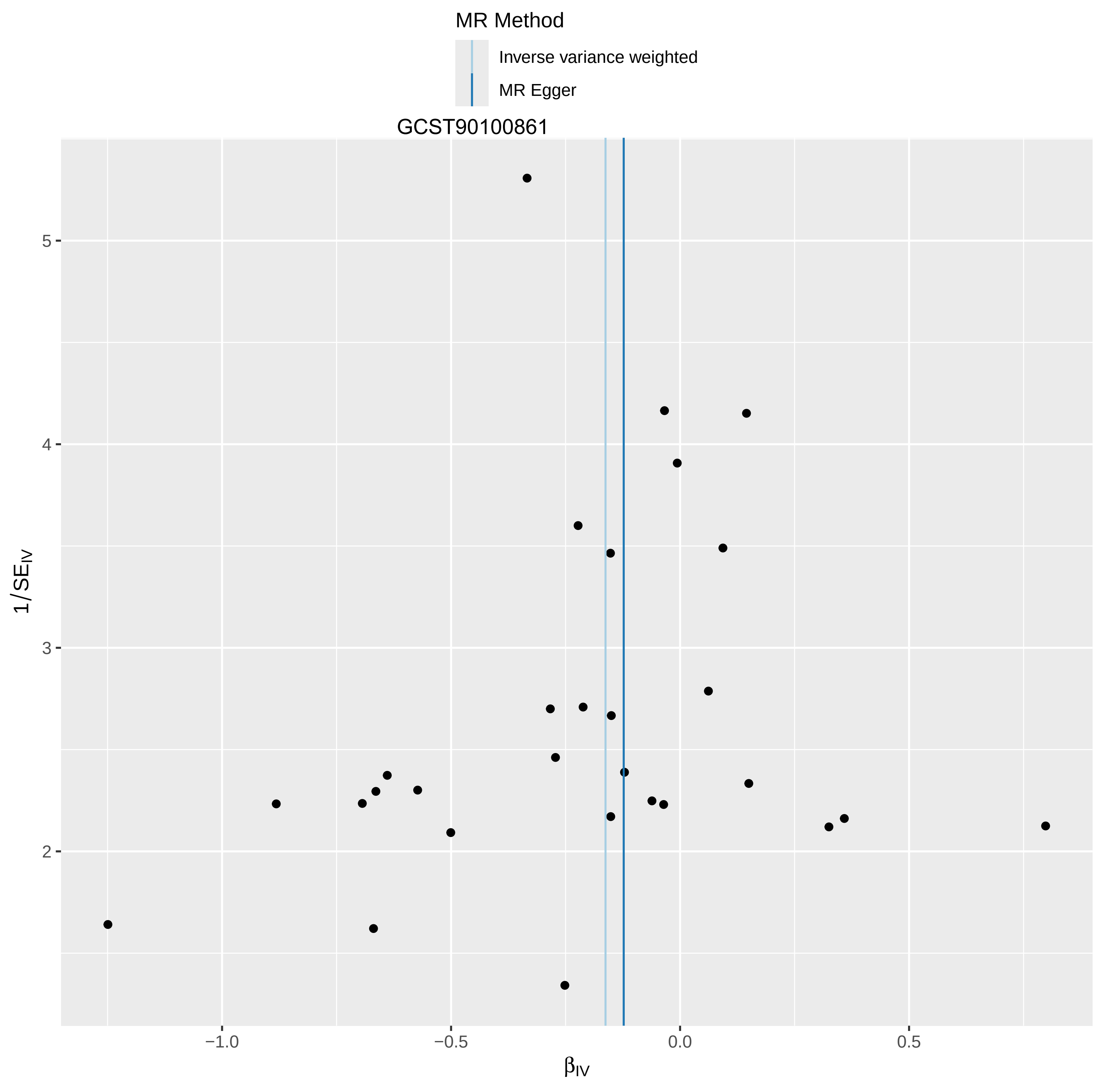
MR Method
Inverse variance weighted
MR Egger
GCST90100861
1/SE_IV
β_IV
5
4
3
2
−1.0
−0.5
0.0
0.5
