# Supplementary material for: Dissecting Causal Relationships Between Gut Microbiota, Plasma Metabolites and Bladder Cancer: A Two‐Step Mendelian Randomization Study
Source: Health Sci Rep. 2025 Sep 9;8(9):e71206. doi: 10.1002/hsr2.71206 (PMC12420358; doi:10.1002/hsr2.71206)
Supplement: Supplementary file 12 — supmat. [file HSR2-8-e71206-s003.docx]

Figure S1 Funnel plots for MR causal effects of gut microbiota on Bca

Figure S2 Leave-one-out analysis for MR causal effects of gut microbiota on Bca

Figure S3 Funnel plots for MR causal effects of plasma metabolites on g_Streptococcus

Figure S4 Leave-one-out analysis for MR causal effects of plasma metabolite on g_Streptococcus

Figure S5 Funnel plots for MR causal effects of plasma metabolites on s_Bacteroides_dorei

Figure S6 Leave-one-out analysis for MR causal effects of plasma metabolites on s_Bacteroides_dorei

Figure S7 Funnel plots for MR causal effects of plasma metabolites on s_Bacteroides_salyersiae

Figure S8 Leave-one-out analysis for MR causal effects of plasma metabolites on s_Bacteroides_salyersiae

Figure S9 Funnel plots for MR causal effects of significantly plasma metabolites on Bca

Figure S10 Leave-one-out analysis for MR causal effects of significantly plasma metabolites on Bca
